# Global, Regional, and National Burden of Cardiovascular Diseases for 10 Causes, 1990 to 2015

**DOI:** 10.1016/j.jacc.2017.04.052

**Published:** 2017-07-04

**Authors:** Gregory A. Roth, Catherine Johnson, Amanuel Abajobir, Foad Abd-Allah, Semaw Ferede Abera, Gebre Abyu, Muktar Ahmed, Baran Aksut, Tahiya Alam, Khurshid Alam, François Alla, Nelson Alvis-Guzman, Stephen Amrock, Hossein Ansari, Johan Ärnlöv, Hamid Asayesh, Tesfay Mehari Atey, Leticia Avila-Burgos, Ashish Awasthi, Amitava Banerjee, Aleksandra Barac, Till Bärnighausen, Lars Barregard, Neeraj Bedi, Ezra Belay Ketema, Derrick Bennett, Gebremedhin Berhe, Zulfiqar Bhutta, Shimelash Bitew, Jonathan Carapetis, Juan Jesus Carrero, Deborah Carvalho Malta, Carlos Andres Castañeda-Orjuela, Jacqueline Castillo-Rivas, Ferrán Catalá-López, Jee-Young Choi, Hanne Christensen, Massimo Cirillo, Leslie Cooper, Michael Criqui, David Cundiff, Albertino Damasceno, Lalit Dandona, Rakhi Dandona, Kairat Davletov, Samath Dharmaratne, Prabhakaran Dorairaj, Manisha Dubey, Rebecca Ehrenkranz, Maysaa El Sayed Zaki, Emerito Jose A. Faraon, Alireza Esteghamati, Talha Farid, Maryam Farvid, Valery Feigin, Eric L. Ding, Gerry Fowkes, Tsegaye Gebrehiwot, Richard Gillum, Audra Gold, Philimon Gona, Rajeev Gupta, Tesfa Dejenie Habtewold, Nima Hafezi-Nejad, Tesfaye Hailu, Gessessew Bugssa Hailu, Graeme Hankey, Hamid Yimam Hassen, Kalkidan Hassen Abate, Rasmus Havmoeller, Simon I. Hay, Masako Horino, Peter J. Hotez, Kathryn Jacobsen, Spencer James, Mehdi Javanbakht, Panniyammakal Jeemon, Denny John, Jost Jonas, Yogeshwar Kalkonde, Chante Karimkhani, Amir Kasaeian, Yousef Khader, Abdur Khan, Young-Ho Khang, Sahil Khera, Abdullah T. Khoja, Jagdish Khubchandani, Daniel Kim, Dhaval Kolte, Soewarta Kosen, Kristopher J. Krohn, G. Anil Kumar, Gene F. Kwan, Dharmesh Kumar Lal, Anders Larsson, Shai Linn, Alan Lopez, Paulo A. Lotufo, Hassan Magdy Abd El Razek, Reza Malekzadeh, Mohsen Mazidi, Toni Meier, Kidanu Gebremariam Meles, George Mensah, Atte Meretoja, Haftay Mezgebe, Ted Miller, Erkin Mirrakhimov, Shafiu Mohammed, Andrew E. Moran, Kamarul Imran Musa, Jagat Narula, Bruce Neal, Frida Ngalesoni, Grant Nguyen, Carla Makhlouf Obermeyer, Mayowa Owolabi, George Patton, João Pedro, Dima Qato, Mostafa Qorbani, Kazem Rahimi, Rajesh Kumar Rai, Salman Rawaf, Antônio Ribeiro, Saeid Safiri, Joshua A. Salomon, Itamar Santos, Milena Santric Milicevic, Benn Sartorius, Aletta Schutte, Sadaf Sepanlou, Masood Ali Shaikh, Min-Jeong Shin, Mehdi Shishehbor, Hirbo Shore, Diego Augusto Santos Silva, Eugene Sobngwi, Saverio Stranges, Soumya Swaminathan, Rafael Tabarés-Seisdedos, Niguse Tadele Atnafu, Fisaha Tesfay, J.S. Thakur, Amanda Thrift, Roman Topor-Madry, Thomas Truelsen, Stefanos Tyrovolas, Kingsley Nnanna Ukwaja, Olalekan Uthman, Tommi Vasankari, Vasiliy Vlassov, Stein Emil Vollset, Tolassa Wakayo, David Watkins, Robert Weintraub, Andrea Werdecker, Ronny Westerman, Charles Shey Wiysonge, Charles Wolfe, Abdulhalik Workicho, Gelin Xu, Yuichiro Yano, Paul Yip, Naohiro Yonemoto, Mustafa Younis, Chuanhua Yu, Theo Vos, Mohsen Naghavi, Christopher Murray

**Affiliations:** a University of Washington, Seattle, Washington; b University of Queensland, Brisbane, Queensland, Australia; c Cairo University, Cairo, Egypt; d Mekelle University, Addis Ababa, Ethiopia; e Jimma University, Jimma, Ethiopia; f Cleveland Clinic, Cleveland, Ohio; g University of Melbourne, Melbourne, Victoria, Australia; h University of Lorraine, Nancy, France; i Universidad de Cartagena, Cartagena, Colombia; j Oregon Health & Science University, Portland, Oregon; k Zahedan University of Medical Sciences, Zahedan, Iran; l Uppsala University, Uppsala, Sweden; m Qom University of Medical Sciences, Qom, Iran; n National Institute of Public Health, Cuernavaca, Mexico; o Sanjay Gandhi Postgraduate Institute of Medical Sciences, Lucknow, India; p University College London, London, United Kingdom; q University of Belgrade, Belgrade, Serbia; r Harvard University, Boston, Massachusetts; s University of Gothenburg, Gothenburg, Sweden; t College of Public Health and Tropical Medicine, Jazan University, Jazan, Saudi Arabia; u University of Oxford, Oxford, United Kingdom; v Aga Khan University, Karachi, Pakistan; w Wolaita Sodo University, Wolaita Sodo, Ethiopia; x The University of Western Australia, Perth, Western Australia, Australia; y Karolinska Institutet, Stockholm, Sweden; z Universidade Federal de Minas Gerais, Belo Horizonte, Brazil; aa Instituto Nacional de Salud, Bogotá, Colombia; bb Caja Costarricense de Seguro Social, San José, Costa Rica; cc University of València/INCLIVA Health Research Institute and CIBERSAM, València, Spain; dd Seoul National University Hospital, Seoul, South Korea; ee Bispebjerg University Hospital, Copenhagen, Denmark; ff University of Salerno, Salerno, Italy; gg Mayo Clinic, Rochester, Minnesota; hh University of California, San Diego, California; ii Long Beach, California; jj Eduardo Mondlane University, Maputo, Mozambique; kk Public Health Foundation of India, New Delhi, India; ll Republican Institute of Cardiology and Internal Diseases, Almaty, Kazakhstan; mm University of Peradeniya, Peradeniya, Sri Lanka; nn Centre for Chronic Disease Control, Gurgaon, India; oo International Institute for Population Sciences, Mumbai, India; pp Mansoura Faculty of Medicine, Mansoura University, Mansoura, Egypt; qq University of Philippines Manila, Manila, Philippines; rr Tehran University of Medical Sciences, Tehran, Iran; ss University of Louisville, Louisville, Kentucky; tt Auckland University of Technology, Auckland, New Zealand; uu University of Edinburgh, Edinburgh United Kingdom; vv University of Massachusetts Boston, Boston, Massachusetts; ww Eternal Heart Care Center and Research Institute, Jaipur, India; xx University of Groningen, Groningen, the Netherlands; yy Mizan-Tepi University, Mizan Teferi, Ethiopia; zz Nevada Division of Public and Behavioral Health, Carson City, Nevada; aaa Baylor College of Medicine, Houston, Texas; bbb George Mason University, Fairfax, Virginia; ccc Denver Health/University of Colorado, Denver, Colorado; ddd University of Aberdeen, Aberdeen, United Kingdom; eee International Center for Research on Women, New Delhi, India; fff Ruprecht-Karls Universitaet Heidelberg, Heidelberg, Germany; ggg Society for Education, Action and Research in Community Health, Gadchiroli, India; hhh Case Western University Hospitals, Cleveland, Ohio; iii Jordan University of Science and Technology, Irbid, Jordan; jjj University of Louisville, Louisville, Kentucky; kkk Seoul National University, Seoul, South Korea; lll New York Medical College, Valhalla, New York; mmm Al-Imam Muhammad Ibn Saud Islamic University (IMSIU), Riyadh, Saudi Arabia; nnn Ball State University, Muncie, Indiana; ooo Northeastern University, Boston, Massachusetts; ppp Brown University, Providence, Rhode Island; qqq Health Policy and Humanities, National Institute of Health Research and Development, Jakarta, Indonesia; rrr Boston University School of Medicine, Boston, Massachusetts; sss University of Haifa, Haifa, Israel; ttt University of São Paulo, São Paulo, Brazil; uuu Chinese Academy of Sciences, Beijing, China; vvv Martin Luther University Halle-Wittenberg, Halle, Germany; www National Institutes of Health, Bethesda, Maryland; xxx Pacific Institute for Research & Evaluation, Beltsville, Maryland; yyy Kyrgyz State Medical Academy, Bishkek, Kyrgyzstan; zzz Ahmadu Bello University, Zaria, Nigeria; aaaa Columbia University, New York, New York; bbbb University of Science Malaysia, Penang, Malaysia; cccc The Mount Sinai Hospital, New York, New York; dddd The George Institute for Global Health, Newtown, New South Wales, Australia; eeee Ministry of Health and Social Welfare, Dar es Salaam, Tanzania; ffff American University of Beirut, Beirut, Lebanon; gggg College of Medicine, University of Ibadan, Ibadan, Nigeria; hhhh University of Porto, Porto, Portugal; iiii University of Illinois at Chicago, Chicago, Illinois; jjjj Alborz University of Medical Sciences, Karaj, Iran; kkkk Society for Health and Demographic Surveillance, Birbhum, India; llll Imperial College London, London, United Kingdom; mmmm Maragheh University of Medical Sciences, Maragheh, Iran; nnnn University of KwaZulu-Natal, Durban, South Africa; oooo North-West University, Potchefstroom, South Africa; pppp Independent Consultant, Islamabad, Pakistan; qqqq Korea University, Seoul, South Korea; rrrr Haramaya University, Dire Dawa, Ethiopia; ssss Federal University of Santa Catarina, Florianópolis, Brazil; tttt University of Yaoundé, Yaoundé, Cameroon; uuuu Luxembourg Institute of Health, Strassen, Luxembourg; vvvv Indian Council of Medical Research, New Delhi, India; wwww Postgraduate Institute of Medical Education and Research, Chandigarh, India; xxxx Monash University, Melbourne, Victoria, Australia; yyyy Jagiellonian University Medical College, Kraków, Poland; zzzz University of Copenhagen, Copenhagen, Denmark; aaaaa Universitat de Barcelona, CIBERSAM, Barcelona, Spain; bbbbb Federal Teaching Hospital, Abakaliki, Nigeria; ccccc University of Warwick, Coventry, United Kingdom; ddddd UKK Institute for Health Promotion Research, Tampere, Finland; eeeee National Research University Higher School of Economics, Moscow, Russia; fffff Norwegian Institute of Public Health, Oslo, Norway; ggggg Royal Children’s Hospital, Melbourne, Victoria, Australia; hhhhh Federal Institute for Population Research, Wiesbaden, Germany; iiiii Cochrane South Africa, Tygerberg, South Africa; jjjjj King’s College London, London, United Kingdom; kkkkk Nanjing University School of Medicine, Nanjing, China; lllll Northwestern University, Chicago, Illinois; mmmmm University of Hong Kong, Pokfulam, Hong Kong; nnnnn Kyoto University, Kyoto, Japan; ooooo Jackson State University, Jackson, Mississippi; ppppp Wuhan University, Wuhan, China

**Keywords:** cause of death, epidemiology, global health, CVD, cardiovascular disease, DALY, disability-adjusted life-year, IHD, ischemic heart disease, PAD, peripheral arterial disease, RHD, rheumatic heart disease, SDI, sociodemographic index, UI, uncertainty interval, YLD, years lived with disability, YLL, years of life lost

## Abstract

**Background:**

The burden of cardiovascular diseases (CVDs) remains unclear in many regions of the world.

**Objectives:**

The GBD (Global Burden of Disease) 2015 study integrated data on disease incidence, prevalence, and mortality to produce consistent, up-to-date estimates for cardiovascular burden.

**Methods:**

CVD mortality was estimated from vital registration and verbal autopsy data. CVD prevalence was estimated using modeling software and data from health surveys, prospective cohorts, health system administrative data, and registries. Years lived with disability (YLD) were estimated by multiplying prevalence by disability weights. Years of life lost (YLL) were estimated by multiplying age-specific CVD deaths by a reference life expectancy. A sociodemographic index (SDI) was created for each location based on income per capita, educational attainment, and fertility.

**Results:**

In 2015, there were an estimated 422.7 million cases of CVD (95% uncertainty interval: 415.53 to 427.87 million cases) and 17.92 million CVD deaths (95% uncertainty interval: 17.59 to 18.28 million CVD deaths). Declines in the age-standardized CVD death rate occurred between 1990 and 2015 in all high-income and some middle-income countries. Ischemic heart disease was the leading cause of CVD health lost globally, as well as in each world region, followed by stroke. As SDI increased beyond 0.25, the highest CVD mortality shifted from women to men. CVD mortality decreased sharply for both sexes in countries with an SDI >0.75.

**Conclusions:**

CVDs remain a major cause of health loss for all regions of the world. Sociodemographic change over the past 25 years has been associated with dramatic declines in CVD in regions with very high SDI, but only a gradual decrease or no change in most regions. Future updates of the GBD study can be used to guide policymakers who are focused on reducing the overall burden of noncommunicable disease and achieving specific global health targets for CVD.

Cardiovascular diseases (CVDs) are a leading cause of death in the world and a major barrier to sustainable human development (1). In 2011, the United Nations formally recognized noncommunicable diseases, including CVDs, as a major concern for global health and set out an ambitious plan to dramatically reduce the effect of these diseases in all regions (2). An increased awareness of these global noncommunicable disease goals has expanded attempts to track and benchmark national efforts at reducing CVD and other noncommunicable diseases 3, 4.

The third Sustainable Development Goal recognized the importance of CVD by targeting a one-third reduction in premature mortality due to noncommunicable diseases (5). Countries that take the SDG goals seriously will have to contend with a wide range of barriers limiting their ability to improve health care and reduce CVD risks. In many regions of the world, the relative position of CVD as a health problem remains unclear or is based on limited data. Many low- and middle-income countries have implemented health examination surveys that have improved measurement of CVD and its associated risk factors (6).

Systematic evaluation of data collected in death certificates, verbal autopsy, health surveys, prospective cohort studies, health system administrative data, and disease registries is needed to appropriately guide efforts to reduce the health burden of CVD. The GBD (Global Burden of Disease) study is an effort to continuously improve our understanding of the burden of diseases by integrating the available data on disease incidence, prevalence, and mortality to produce consistent, transparent, and up-to-date global, regional, and national estimates (7).

The global number of CVD deaths and regional patterns of total CVD mortality were previously reported from the GBD 2013 study (8). The GBD 2015 study results provide a completely new mortality time-series estimated from 1990 forward and updated through 2015. We now also report national estimates of mortality to provide results relevant to specific countries at the level of each underlying CVD condition. In addition, the study addresses the nonfatal burden of CVD by reporting global, regional, and national estimates of prevalence, years lived with disability, and disability-adjusted life years.

## Methods

### GBD estimation framework

The Global Burden of Diseases, Injuries, and Risk Factors 2015 study is a multinational collaborative research project with the goal of producing consistent estimates of health loss due to over 310 diseases and injuries. A wide range of data sources and methods were used to produce age-, sex-, and country-specific results for the years 1990 to 2015. Results are updated annually for the entire time series, and these results supersede previous versions of the GBD study. Methods have been reported in detail previously and are summarized here and in the Online Appendix 9, 10, 11.

### Changes since the GBD 2013 study

There have been numerous changes to data collection and methods in the current study to update and improve upon the results of the GBD 2013 study. Mortality data has been updated through 2014 using newly-identified data sources released or collected since GBD 2013. Because of their available vital registration data, 7 territories have been added: American Samoa, Bermuda, Greenland, Guam, the Northern Mariana Islands, Puerto Rico, and the Virgin Islands. Disease burden from these territories are now included in the national totals for the United States, United Kingdom, and Denmark. A new approach to estimating uncertainty for countries with long (>95% complete) time series of vital registration data has been used uniformly for all CVD causes so that results are not affected by uncertainty in regions with less complete vital registration. Models of disease incidence and prevalence now uniformly include estimates of excess mortality and, for stroke, cause-specific mortality, so that they are better informed by the available mortality data. For each incidence or prevalence data point, we matched the age-sex-location-year-cause–specific mortality rate to produce a ratio conceptually equivalent to an excess mortality rate. Because of implausibly rapid increases in deaths reported due to atrial fibrillation, we have developed a unified model of atrial fibrillation that makes use of prevalence, case fatality, and mortality data to estimate both the nonfatal and fatal burden due to this condition.

### Defining disease categories

CVD was estimated overall and separately for the 10 most common global causes of CVD-related death. These causes were ischemic heart disease (IHD), ischemic stroke, hemorrhagic and other stroke, atrial fibrillation, peripheral arterial disease (PAD), aortic aneurysm, cardiomyopathy and myocarditis, hypertensive heart disease, endocarditis, rheumatic heart disease (RHD), and a category for other CVD conditions. The GBD cause list is a hierarchical, mutually-exclusive, and collectively exhaustive list of causes of death. The 3 level 1 GBD causes consist of communicable, maternal, neonatal, and nutritional disorders; noncommunicable diseases; and injuries. Level 2 causes consist of 21 cause groups, such as neoplasms and CVD. Levels 3 and 4 consist of disaggregated subcauses (Online Methods Appendix Table 1).

Cause of death was defined by international standards governing the reporting of death certificates, in which a single underlying cause is assigned by a physician. For example, IHD was defined as an underlying cause of death across International Classification of Diseases (ICD) revisions (most recently ICD-10 I20 to I25, ICD-9 410 to 414) (12). The leading causes included as “other cardiovascular and circulatory diseases” were nonrheumatic valvular disorders and pulmonary embolism. A proportion of deaths that were assigned on death certificates to nonfatal, undefined, or intermediate causes (e.g., cardiac arrest, heart failure, or hypertension) were redistributed using statistical regression methods or fixed proportions (9). Redistribution of deaths coded to heart failure was accomplished using a regression model that accounted for the variable use of these codes by age, sex, and location. This approach improves upon methods that either exclude deaths coded to an intermediate cause or reassign them using a fixed proportion that ignores variation by age, sex, or location. Deaths due to unspecified types of stroke (ICD-10 I64) were distributed using the ratio of ischemic to hemorrhagic stroke deaths in a country’s region or, for South Asia, the global ratio, stratified by age. A Bayesian noise reduction algorithm was applied to death data to improve estimation of the underlying mortality rate (see the Online Appendix for details). This noise reduction algorithm was adopted to improve upon prior methods in which 0 counts were excluded, an approach that leads to an upward bias in estimates. Verbal autopsy, a method in which a standardized interview collects information from household members on symptoms preceding death, was included as a data input only for total CVD, ischemic heart disease, and stroke deaths, and was excluded for other CVD causes of death.

Disease prevalence was estimated at a more granular level of specific disease sequelae, using input data from systematic reviews of the published scientific reports, unpublished registry data, and health system administrative data. A regression equation was used to adjust data in the direction of the gold-standard case definition. Detailed nonfatal modeling methods are included in the Online Appendix. IHD was the summation of 4 distinct disease sequelae: acute myocardial infarction, chronic stable angina, chronic IHD, and heart failure due to IHD. Myocardial infarction was defined according to the Third Universal Definition of Myocardial Infarction and the case-finding approach from the MONICA (Multinational MONItoring of trends and determinants in CArdiovascular disease) studies, which accounts for out-of-hospital sudden cardiac death 13, 14. Adjustments were made for the advent of troponin-testing technology for diagnosis of acute coronary syndromes during the years covered by the study using meta-analysis of its increased sensitivity (compared with prior markers) to adjust pre-2000 incidence rates upward by 56%. Stable angina was defined according to the Rose Angina Questionnaire, which was adjusted to account for the observed differences in survey and administrative data found in the United States. Cerebrovascular disease relied on a case definition developed by the World Health Organization and was estimated separately for 2 subcategories: 1) ischemic stroke; and 2) hemorrhagic or other nonischemic stroke (15). Stroke data was adjusted to match our case definition of subtype-specific first-ever incident events, and was used to separately estimate acute and chronic stroke. PAD was defined by an ankle brachial index (ABI) <0.9, and symptomatic PAD was defined as self-report of claudicatory symptoms among those with ABI <0.9 (16). Atrial fibrillation was defined by electrocardiogram and included atrial flutter. The prevalence of symptomatic heart failure was estimated using both health system administrative and population-based registry data, and was then attributed to specific underlying heart failure etiologies (some of which were not CVD). Hypertensive heart disease was defined as symptomatic heart failure due to the direct and long-term effects of hypertension, with its nonfatal burden derived from the model of heart failure. Cardiomyopathy was defined as symptomatic heart failure due to primary myocardial disease or toxic exposures, such as alcohol, with its nonfatal burden derived from the model of heart failure (17). Acute myocarditis was estimated as an acute and time-limited condition due to myocardial inflammation using health system administrative data. Endocarditis and RHD were defined by their clinical diagnosis. Estimates of RHD include cases identified by clinical history and physical examination, including auscultation or standard echocardiographic criteria for definite disease.

### Data sources and analytic methods

A map of data availability for each country are included in Online Figures 1A and 1B (9). The GBD 2015 study used country-level surveillance data, verbal autopsy, vital registration, published and unpublished disease registries, and published scientific reports. Table 1 summarizes data sources used to estimate CVD burden. Table 1 also shows the data representativeness index for nonfatal estimates, which is the proportion of age-sex-location strata with available data for nonfatal modeling shown by cause and over time. Online Methods Appendix Tables 2 and 3 are tables of all data sources. Data sources for models are also available online from the Global Health Data Exchange (18). National income, metabolic and nutritional risk factors, and other country-level covariates were estimated from surveys and published systematic reviews. Analysis of mortality used Cause of Death Ensemble modeling (CODEm), an approach that incorporates country-level covariates, including age-sex-country-year–specific estimates of CVD risk factors, national income, and other causal factors (Online Appendix). CODEm borrows strength across space, time, and age groups using a variety of geospatial model types, and weighs the results using tests of out-of-sample predictive validity. Analysis of disease prevalence used epidemiological state-transition–based disease modeling software, DisMod-MR, which accounts for study-level differences in measurement method (9). Disease-specific incidence, prevalence, case fatality, and mortality rates were integrated to produce consistent estimates of prevalence of all geographies in the study (19). Estimates were considered significantly different if there was no overlap in their 95% uncertainty intervals (UIs). The cause-specific mortality rate for atrial fibrillation was also estimated using DisMod because of implausible increases in the rate when derived only from death certificates. Prevalent cases of each disease’s sequelae are assigned specific levels of severity based on the U.S. Medical Expenditure Panel Survey 2000 to 2011, a population-based survey with data on functional health that also provides linkage to respondent medical records. DisMod-MR models were run separately by sex, country, and year.

**Table 1:** Data Representativeness in GBD 2015 Fatal and Nonfatal Modeling by CVD Cause

Cause	Number of Site-Years of Mortality Data		Percentage of Geographies With Data for Nonfatal Modeling		
	Vital Registration	Verbal Autopsy	Before 2005	2005–2015	Total
Cardiovascular diseases	10,446	964	81	74	85
Rheumatic heart disease	10,417	0	29	27	37
Ischemic heart disease	10,652	734	47	30	51
Cerebrovascular disease	10,660	692	64	67	74
Ischemic stroke	9,207	0	63	61	68
Hemorrhagic stroke	9,211	0	63	61	68
Hypertensive heart disease	10,039	0	13	6	16
Cardiomyopathy and myocarditis	10,020	0	25	22	31
Atrial fibrillation and flutter	8,104	0	22	24	27
Aortic aneurysm∗	9,215	0	N/A	N/A	N/A
Peripheral vascular disease	8,087	0	19	20	23
Endocarditis	9,274	0	18	19	21
Other cardiovascular and circulatory diseases	10,340	0	1	1	1

CVD = cardiovascular disease; GBD = Global Burden of Disease; N/A = not available.

∗ Nonfatal estimates are not produced for aortic aneurysm.

### Disability-adjusted life years

Disability-adjusted life-years (DALYs) combine information regarding premature death (years of life lost [YLL]) and disability caused by the condition (years lived with disability [YLD]) to provide a summary measure of health lost due to that condition. YLL was calculated by multiplying observed deaths for a specific age in the year of interest by the age-specific reference life expectancy estimated using life table methods. The normative standard life expectancy at birth is 86.59 years, based on the lowest observed death rates for each 5-year age group in populations larger than 5 million. YLD was calculated by multiplying disease prevalence (in number of cases) by a health-state–specific disability weight representing a degree of lost functional capacity. A detailed explanation of the process of disability weight estimation has been reported separately 10, 11. Briefly, disability weights were developed using household surveys in multiple countries that asked members of the general public to choose between lay descriptions of health states 20, 21. Adjustment was made for comorbidity by simulating 40,000 individuals in each age-sex-country-year stratum exposed to the independent probability of acquiring each condition based on disease prevalence.

The 95% UIs reported for each estimate used 1,000 samples from the posterior distribution from the respective step in the modeling process, reported as the 2.5th and 97.5th values of the distribution. Age standardization was via the direct method, applying a global age structure.

### Sociodemographic index

Instead of using the categories of national socioeconomic status developed for the GBD 2013 study, we have produced a new continuous measure of sociodemographic status. The sociodemographic index (SDI) was estimated to examine changes in CVD burden as a function of the global epidemiological transition (9). Similar to the method used to compute the human development index, SDI was calculated for each country or territory in each year from 1990 to 2015. SDI was the equally-weighted geometric mean of income per capita, educational attainment, and total fertility rate. Least squares regression of death rates on SDI was used with a smoothing spline and dummy variables for outlier regions that skewed fit to capture the average relationship for each age-sex-cause group.

## Results

All results of the GBD 2015 study, including prevalence, mortality, YLL, YLD, and DALYs, for all country-years are available for download from the GBD results tool webpage (22) and can be explored visually at the GBD Compare visualization hub (23).

### Regional variation in CVD

#### Prevalence

**Figure 1:**
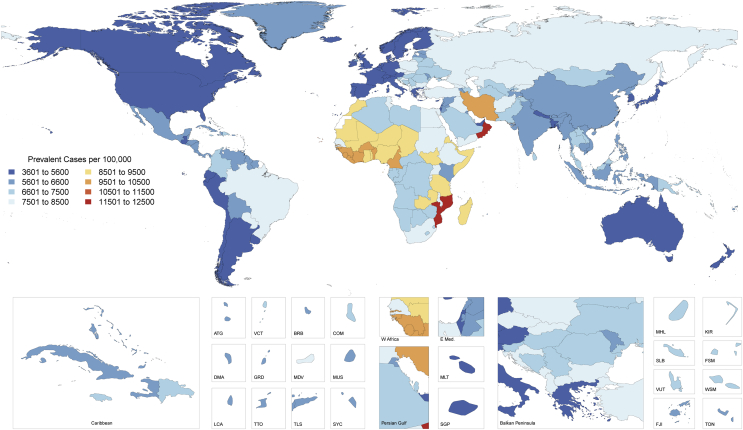
Global Map, Age-Standardized Prevalence of CVD in 2015 Choropleth map showing the estimated age-standardized prevalence of total CVD in 2015 for each country. ATG = Antigua and Barbuda; BRB = Barbados; COM = Comoros; CVD = cardiovascular diseases; DMA = Dominica; E Med = Eastern Mediterranean; FJI = Fiji; FSM = Federated States of Micronesia; GRD = Grenada; KIR = Kiribati; KS = Kaposi sarcoma; LCA = Saint Lucia; MDV = Maldives; MHL = Marshall Islands; MLT = Malta; MUS = Mauritius; NMSC = nonmelanoma skin cancer; SGP = Singapore; SLB = Solomon Islands; SYC = Seychelles; TLS = Timor-Leste; TON = Tonga; TTO = Trinidad and Tobago; VCT = Saint Vincent and the Grenadines; VUT = Vanuatu; W Africa = West Africa; WSM = Samoa.

Globally, there were an estimated 422.7 million prevalent cases of CVD (95% UI: 415.53 to 427.87 million cases) in 2015 (Table 2). The age-standardized prevalence of CVD varied significantly by country (Figure 1). Countries with the lowest age-standardized prevalence in 2015, all with <5,000 cases per 100,000 individuals, included Singapore, Japan, South Korea, Chile, Argentina, Uruguay, Canada, Australia, New Zealand, Ireland, Cyprus, Malta, Italy, Greece, and Israel. Countries in Western Europe, as well as the United States, the United Arab Emirates, and Nepal, all had only slightly higher prevalence. Countries with the highest age-standardized prevalence in 2015, all >9,000 cases per 100,000 persons, included most countries in West Africa, Morocco, Iran, Oman, Zambia, Mozambique, and Madagascar.

**Table 2:** Global and Regional All-Age Deaths and Age-Standardized Death Rates in 2015, by Sex, for Selected Causes of CVD Mortality∗

	Death					
	All Ages			Age-Standardized (per 100,000)		
	Total	Female	Male	Total	Female	Male
Cardiovascular diseases						
Global	17,921,047 (17,590,537–18,276,848)	8,501,409 (8,301,355–8,722,665)	9,419,637 (9,199,720–9,648,088)	286 (280–291)	242 (236–248)	335 (327–342)
Andean Latin America	63,861 (59,748–68,356)	32,495 (29,222–35,927)	31,366 (28,782–34,204)	157 (146–168)	144 (130–160)	170 (156–185)
Australasia	67,481 (65,263–69,507)	34,620 (33,129–36,147)	32,861 (31,754–34,076)	147 (143–151)	127 (122–132)	168 (163–175)
Caribbean	126,769 (121,035–132,439)	65,947 (61,476–70,829)	60,822 (57,446–64,113)	293 (280–306)	274 (254–294)	314 (298–331)
Central Asia	304,212 (296,495–311,855)	148,071 (142,407–153,246)	156,141 (151,207–161,193)	545 (532–558)	451 (433–466)	674 (654–693)
Central Europe	666,173 (654,844–676,711)	355,129 (347,583–362,138)	311,044 (305,556–317,045)	338 (333–344)	278 (272–283)	419 (411–427)
Central Latin America	337,507 (328,984–345,456)	167,760 (162,327–173,252)	169,747 (164,317–175,158)	198 (193–203)	176 (171–182)	223 (216–229)
Central sub-Saharan Africa	147,629 (100,125–205,190)	85,290 (54,094–123,452)	62,339 (40,084–92,009)	418 (291–560)	455 (298–633)	366 (244–519)
East Asia	3,953,300 (3,805,196–4,117,647)	1,651,066 (1,566,414–1,740,137)	2,302,234 (2,183,097–2,432,661)	295 (284–307)	237 (225–249)	359 (341–377)
Eastern Europe	1,774,861 (1,740,489–1,811,091)	993,829 (969,164–1,020,806)	781,033 (761,821–801,141)	532 (522–543)	423 (413–435)	701 (685–718)
Eastern sub-Saharan Africa	424,364 (353,978–507,026)	218,704 (169,208–277,600)	205,661 (163,706–260,525)	349 (295–414)	346 (272–433)	352 (285–437)
High-income Asia Pacific	498,622 (485,719–511,659)	270,969 (261,880–280,189)	227,653 (222,117–233,411)	112 (110–115)	93 (90–96)	135 (131–138)
High-income North America	946,416 (924,685–967,818)	474,764 (460,316–489,759)	471,652 (461,763–481,077)	171 (168–175)	143 (139–147)	204 (200–208)
North Africa and Middle East	1,079,493 (1,028,619–1,134,703)	508,366 (475,397–543,603)	571,127 (537,220–607,652)	361 (344–376)	326 (306–347)	398 (376–421)
Oceania	27,503 (20,884–36,700)	13,649 (10,261–18,281)	13,854 (10,540–18,619)	525 (416–664)	506 (392–648)	540 (432–677)
South Asia	3,610,666 (3,473,581–3,755,833)	1,509,355 (1,420,049–1,599,458)	2,101,312 (1,993,733–2,221,549)	369 (355–383)	314 (296–332)	424 (404–447)
Southeast Asia	1,351,557 (1,238,336–1,455,239)	632,078 (565,558–694,348)	719,479 (633,386–798,803)	321 (296–344)	274 (247–300)	377 (335–411)
Southern Latin America	164,667 (160,162–169,048)	87,224 (83,804–90,842)	77,443 (74,888–79,792)	218 (212–224)	178 (172–186)	269 (260–277)
Southern sub-Saharan Africa	136,002 (123,737–150,420)	78,333 (69,335–88,846)	57,669 (51,859–64,908)	338 (309–372)	321 (285–363)	349 (317–387)
Tropical Latin America	435,272 (418,494–455,826)	205,462 (195,338–218,739)	229,811 (218,571–242,203)	256 (247–269)	211 (201–225)	316 (301–332)
Western Europe	1,483,792 (1,444,804–1,521,399)	798,509 (772,187–825,482)	685,283 (668,957–701,669)	157 (154–161)	132 (128–135)	187 (183–192)
Western sub-Saharan Africa	320,897 (274,658–384,354)	169,791 (136,039–224,498)	151,106 (125,014–187,897)	285 (247–335)	298 (244–386)	266 (226–324)
Ischemic heart disease						
Global	8,916,964 (8,751,617–9,108,850)	4,035,936 (3,941,319–4,146,339)	4,881,028 (4,747,381–5,022,975)	142 (140–145)	115 (112–118)	173 (168–178)
Andean Latin America	34,041 (31,629–36,640)	16,786 (14,973–18,783)	17,255 (15,646–18,940)	84 (78–91)	75 (67–84)	94 (86–103)
Australasia	38,507 (36,922–40,107)	18,382 (17,258–19,556)	20,125 (19,203–21,078)	84 (81–88)	67 (63–71)	103 (99–108)
Caribbean	65,422 (62,394–68,337)	31,991 (29,892–34,192)	33,431 (31,703–35,282)	151 (144–158)	132 (123–142)	173 (164–182)
Central Asia	185,521 (179,788–191,429)	88,069 (84,158–91,666)	97,452 (93,405–101,577)	336 (326–347)	271 (259–282)	425 (409–442)
Central Europe	357,073 (350,311–364,381)	182,915 (177,942–187,778)	174,158 (170,286–178,258)	181 (177–184)	141 (138–145)	234 (229–240)
Central Latin America	202,329 (196,619–207,546)	95,669 (92,132–99,212)	106,660 (103,143–110,376)	119 (116–122)	101 (97–105)	140 (135–144)
Central sub-Saharan Africa	47,589 (31,139–67,216)	24,686 (15,044–36,067)	22,902 (14,369–35,115)	143 (94–196)	143 (89–205)	139 (91–204)
East Asia	1,507,596 (1,443,996–1,579,379)	636,714 (599,802–674,258)	870,883 (817,224–928,402)	114 (109–119)	92 (87–98)	137 (129–145)
Eastern Europe	1,093,600 (1,070,126–1,117,719)	599,344 (580,595–618,307)	494,256 (480,975–507,720)	326 (319–333)	252 (245–260)	445 (433–456)
Eastern sub-Saharan Africa	143,019 (113,946–175,641)	64,860 (46,988–86,834)	78,159 (60,498–102,024)	122 (98–147)	108 (79–142)	137 (109–174)
High-income Asia Pacific	197,492 (190,870–203,632)	103,780 (98,289–108,644)	93,712 (90,345–97,136)	45 (44–46)	35 (34–37)	56 (54–58)
High-income North America	583,761 (565,503–600,239)	276,513 (265,351–286,639)	307,247 (297,884–315,053)	106 (102–108)	83 (79–86)	133 (129–136)
North Africa and Middle East	599,360 (565,847–631,996)	257,621 (239,786–276,947)	341,738 (319,275–366,768)	201 (190–210)	168 (157–179)	236 (222–252)
Oceania	12,707 (9,738–16,864)	5,309 (3,976–7,101)	7,399 (5,637–9,955)	240 (192–304)	205 (160–262)	275 (219–345)
South Asia	2,073,496 (1,985,218–2,169,575)	805,483 (754,450–868,996)	1,268,013 (1,197,852–1,346,206)	212 (204–221)	171 (160–184)	254 (240–268)
Southeast Asia	558,700 (510,069–601,308)	236,125 (213,084–261,064)	322,575 (284,187–356,407)	131 (121–140)	103 (93–113)	166 (148–182)
Southern Latin America	83,253 (80,419–86,198)	41,921 (39,779–44,220)	41,332 (39,592–42,881)	110 (107–114)	85 (81–89)	143 (137–148)
Southern sub-Saharan Africa	56,521 (51,368–62,508)	31,275 (27,621–35,669)	25,246 (22,593–28,281)	142 (130–157)	130 (115–148)	154 (140–171)
Tropical Latin America	201,510 (193,188–211,658)	90,837 (85,863–97,235)	110,673 (103,939–117,172)	118 (113–124)	94 (89–100)	149 (140–158)
Western Europe	745,878 (722,801–767,056)	365,207 (350,185–380,330)	380,670 (369,601–391,158)	80 (78–82)	60 (58–62)	105 (102–108)
Western sub-Saharan Africa	129,590 (111,345–153,647)	62,449 (50,092–82,307)	67,141 (54,806–84,115)	123 (107–144)	119 (98–151)	127 (106–155)
Ischemic strokes						
Global	2,977,980 (2,880,779–3,068,756)	1,550,557 (1,477,734–1,619,514)	1,427,423 (1,369,627–1,484,115)	49 (47–50)	44 (42–46)	54 (52–56)
Andean Latin America	9,701 (8,824–10,726)	5,215 (4,501–5,941)	4,485 (3,970–5,121)	24 (22–27)	23 (20–27)	25 (23–29)
Australasia	8,726 (8,048–9,498)	5,110 (4,560–5,752)	3,616 (3,264–3,997)	18 (17–20)	18 (16–20)	18 (16–20)
Caribbean	20,504 (19,174–22,125)	11,679 (10,509–13,181)	8,824 (8,159–9,610)	48 (45–51)	48 (43–54)	47 (43–51)
Central Asia	39,172 (37,193–41,353)	20,028 (18,461–21,835)	19,144 (17,907–20,352)	73 (69–77)	62 (57–68)	89 (83–95)
Central Europe	125,872 (122,372–129,393)	74,220 (71,301–77,020)	51,652 (49,753–53,800)	63 (61–64)	57 (55–59)	70 (67–73)
Central Latin America	37,869 (36,541–39,267)	20,762 (19,776–21,833)	17,107 (16,369–17,959)	23 (22–24)	22 (21–23)	24 (23–26)
Central sub-Saharan Africa	22,654 (14,109–32,102)	14,551 (8,419–21,794)	8,103 (4,927–12,473)	77 (49–108)	89 (53–133)	60 (37–89)
East Asia	785,226 (703,812–827,827)	330,404 (271,544–357,625)	454,821 (418,276–485,389)	61 (54–64)	48 (40–52)	74 (68–79)
Eastern Europe	385,151 (372,405–399,825)	245,598 (234,439–257,431)	139,553 (132,831–146,054)	113 (109–117)	102 (97–107)	131 (124–137)
Eastern sub-Saharan Africa	60,759 (46,770–76,049)	34,978 (22,707–49,063)	25,781 (19,130–34,120)	57 (43–70)	61 (40–83)	52 (39–67)
High-income Asia Pacific	132,454 (127,229–137,646)	74,185 (70,362–77,931)	58,269 (55,682–60,833)	28 (27–29)	24 (22–25)	33 (31–34)
High-income North America	123,894 (118,687–129,215)	73,295 (69,314–77,624)	50,599 (48,173–53,465)	21 (21–22)	21 (20–22)	22 (21–23)
North Africa and Middle East	149,264 (136,741–159,778)	80,022 (71,508–88,363)	69,242 (62,597–75,881)	55 (51–59)	55 (49–60)	56 (51–61)
Oceania	3,364 (2,427–4,652)	1,843 (1,269–2,891)	1,521 (1,088–2,178)	77 (58–103)	78 (55–118)	76 (57–102)
South Asia	500,203 (457,812–552,667)	229,925 (198,486–274,331)	270,278 (245,928–297,888)	57 (53–63)	52 (45–62)	63 (57–69)
Southeast Asia	215,754 (192,311–239,386)	116,663 (100,381–133,400)	99,091 (83,978–113,695)	58 (52–64)	55 (47–62)	62 (53–70)
Southern Latin America	23,765 (22,597–25,049)	13,896 (12,952–14,931)	9,869 (9,256–10,486)	31 (29–32)	27 (25–29)	35 (33–38)
Southern sub-Saharan Africa	21,684 (19,588–23,974)	14,081 (12,432–15,964)	7,603 (6,822–8,508)	58 (53–65)	60 (53–68)	54 (48–60)
Tropical Latin America	48,553 (46,004–53,803)	23,527 (21,698–27,136)	25,026 (23,488–26,988)	31 (29–34)	25 (23–29)	39 (37–43)
Western Europe	208,929 (199,938–219,001)	126,842 (119,343–134,411)	82,087 (78,376–86,524)	21 (20–22)	20 (19–21)	22 (21–23)
Western sub-Saharan Africa	54,484 (45,545–66,759)	33,733 (26,361–44,685)	20,752 (16,778–26,538)	60 (51–72)	70 (56–91)	47 (39–58)
Hemorrhagic and other strokes						
Global	3,348,155 (3,240,908–3,500,094)	1,544,379 (1,472,186–1,644,306)	1,803,776 (1,728,916–1,888,569)	52 (50–54)	44 (42–47)	62 (59–65)
Andean Latin America	9,090 (8,336–10,212)	4,756 (4,169–5,506)	4,334 (3,827–4,957)	21 (19–24)	21 (18–24)	22 (19–25)
Australasia	9,007 (8,338–9,723)	5,345 (4,818–5,960)	3,661 (3,329–4,043)	20 (18–21)	20 (18–22)	19 (17–21)
Caribbean	19,121 (17,586–20,601)	10,401 (9,105–11,825)	8,720 (7,952–9,529)	44 (41–47)	44 (38–50)	44 (40–48)
Central Asia	46,416 (44,225–49,783)	24,013 (22,074–27,101)	22,403 (21,174–23,800)	80 (76–86)	71 (66–80)	92 (86–99)
Central Europe	75,563 (73,070–78,708)	40,535 (38,502–42,524)	35,028 (33,720–37,208)	40 (38–41)	34 (32–36)	47 (45–50)
Central Latin America	47,484 (45,863–49,166)	24,888 (23,802–26,120)	22,596 (21,627–23,654)	27 (26–28)	26 (24–27)	28 (27–29)
Central sub-Saharan Africa	38,990 (25,248–55,651)	22,364 (12,823–33,985)	16,626 (10,509–25,575)	99 (62–139)	107 (59–159)	89 (57–135)
East Asia	1,168,477 (1,109,258–1,263,491)	457,939 (424,166–529,581)	710,537 (662,234–763,372)	83 (79–91)	63 (59–74)	105 (98–112)
Eastern Europe	135,182 (127,552–143,922)	74,236 (68,208–80,933)	60,946 (56,905–65,141)	41 (39–44)	34 (31–37)	53 (49–56)
Eastern sub-Saharan Africa	93,683 (73,892–115,030)	49,175 (33,474–65,910)	44,509 (33,637–57,836)	69 (54–85)	69 (45–92)	69 (53–89)
High-income Asia Pacific	84,648 (81,230–88,822)	42,987 (40,471–45,896)	41,661 (39,815–43,536)	21 (20–22)	17 (16–18)	26 (25–27)
High-income North America	58,576 (56,053–61,567)	31,967 (30,125–34,035)	26,609 (25,175–28,227)	11 (11–12)	11 (10–11)	12 (11–12)
North Africa and Middle East	153,741 (141,926–171,810)	77,195 (68,269–88,362)	76,546 (70,712–87,959)	48 (44–53)	46 (41–53)	50 (46–57)
Oceania	6,272 (4,551–8,805)	3,664 (2,538–5,333)	2,608 (1,822–3,673)	114 (82–152)	127 (87–180)	96 (70–126)
South Asia	594,675 (542,901–645,401)	269,859 (223,569–308,772)	324,816 (290,521–360,005)	56 (51–62)	52 (42–60)	61 (54–68)
Southeast Asia	409,791 (366,137–451,121)	192,227 (165,661–219,609)	217,564 (188,214–247,832)	93 (84–102)	80 (69–92)	107 (93–121)
Southern Latin America	18,630 (17,766–19,510)	9,436 (8,777–10,113)	9,194 (8,677–9,734)	26 (24–27)	21 (20–23)	31 (29–33)
Southern sub-Saharan Africa	25,307 (22,691–28,908)	14,474 (12,620–16,999)	10,833 (9,475–12,614)	60 (54–69)	58 (51–68)	61 (54–71)
Tropical Latin America	99,629 (94,975–106,647)	49,108 (46,136–52,691)	50,521 (47,601–56,066)	57 (55–61)	50 (47–53)	67 (63–75)
Western Europe	174,106 (166,453–182,116)	98,706 (92,998–104,421)	75,400 (71,667–79,634)	19 (18–20)	17 (16–18)	21 (20–22)
Western sub-Saharan Africa	79,764 (67,506–97,152)	41,101 (32,116–55,046)	38,662 (31,506–49,231)	59 (50–71)	60 (48–80)	57 (47–71)

Values are n (95% uncertainty intervals).

Abbreviations as in Table 1.

∗ Results for all causes are available online via the GBD Compare web visualization tool (23).

Many countries had no significant change in the estimated age-standardized prevalence of CVD from 1990 to 2015, often reflecting low data availability (Online Figures 1B and 2A). A number of countries showed a significant decline in the age-standardized prevalence of CVD, including the United States, Canada, Western Europe, Brazil, Australia, New Zealand, Japan, South Korea, Kenya, Cambodia, Laos, and India. There was a significant but small (<1%) increase in the age-standardized prevalence of CVD in Mexico, Venezuela, Saudi Arabia, and Mongolia.

#### Deaths

There were 12.59 million deaths (95% UI: 12.38 to 12.80 million deaths) due to CVD in 1990, increasing to 17.92 million deaths (95% UI: 17.59 to 18.28 million deaths) in 2015. There was broad variation in the age-standardized CVD mortality rate among countries (Central Illustration). Significant declines in the age-standardized death rate due to CVD occurred between 1990 and 2015 in all high-income and some middle-income countries, but no significant changes were detected over this time period for most of sub-Saharan Africa and multiple countries in Oceania and Southeast Asia, as well as Pakistan, Afghanistan, Kyrgyzstan, and Mongolia (Online Figure 2B). Bangladesh and the Philippines had significant increases in the age-standardized death rate due to CVD.

**Central Illustration:**
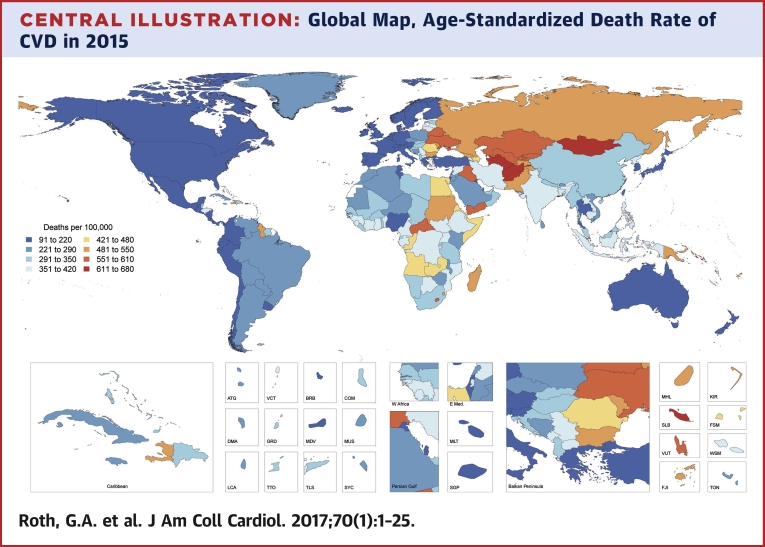
Global Map, Age-Standardized Death Rate of CVD in 2015 Choropleth map showing the estimated age-standardized mortality rate of total CVD in 2015 for each country. ATG = Antigua and Barbuda; BRB = Barbados; COM = Comoros; CVD = cardiovascular diseases; DMA = Dominica; E Med = Eastern Mediterranean; FJI = Fiji; FSM = Federated States of Micronesia; GRD = Grenada; KIR = Kiribati; KS = Kaposi sarcoma; LCA = Saint Lucia; MDV = Maldives; MHL = Marshall Islands; MLT = Malta; MUS = Mauritius; NMSC = nonmelanoma skin cancer; SGP = Singapore; SLB = Solomon Islands; SYC = Seychelles; TLS = Timor-Leste; TON = Tonga; TTO = Trinidad and Tobago; VCT = Saint Vincent and the Grenadines; VUT = Vanuatu; W Africa = West Africa; WSM = Samoa.

### CVD deaths and sociodemographic transition

**Figure 2:**
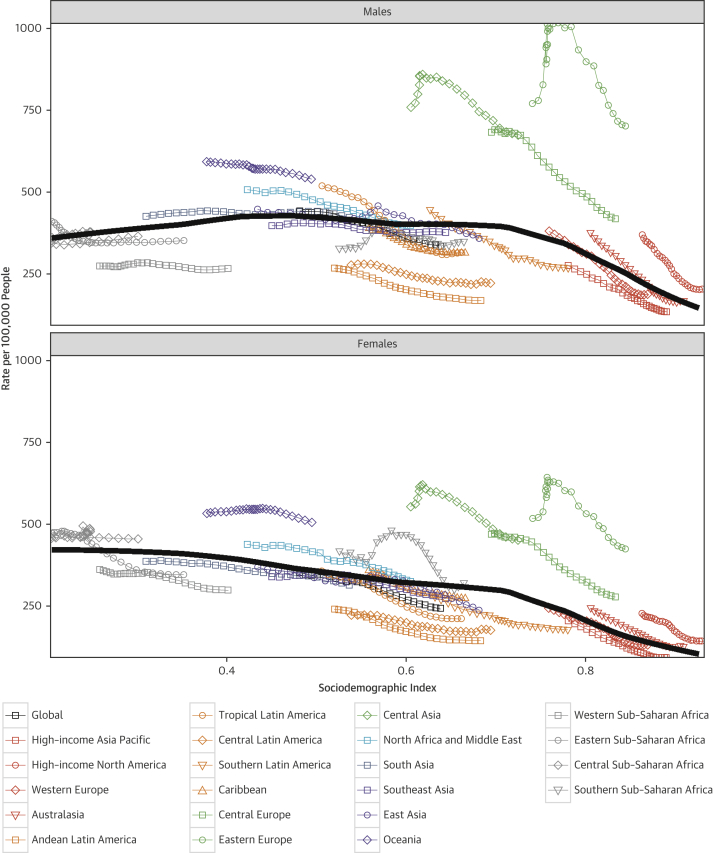
Age-Standardized CVD Mortality Rate, From 1990 to 2015, of 21 GBD World Regions by SDI Relationship between age-standardized mortality rate for CVD and SDI over time. Each **colored line** represents a time trend of the relationship for the specified region. Each **point** represents a specific year for that region. The **black line** represents the overall global trend for age-standardized death rate of CVD in relation to SDI. CVD = cardiovascular diseases; GBD = Global Burden of Disease; SDI = sociodemographic index.

When each world region’s age-standardized CVD death rate for each year from 1990 to 2015 is plotted against an index of that region’s sociodemographic status in the same year, distinct patterns of epidemiological change are observed (Figure 2). Both an increase in SDI and a decline in cardiovascular mortality occurred in many regions. Despite marked improvement in SDI, CVD mortality did not decrease for men in South Asia and for both sexes in much of sub-Saharan Africa. A smaller rise and fall were seen for women in Oceania and men in East Asia. CVD mortality declined among high-income regions, but has plateaued in recent years. The lack of further decline in CVD mortality is particularly notable in high-income North America, Australasia, Western Europe, and the Caribbean. The maximum likelihood estimate relationship between SDI and CVD mortality, shown as a black line in Figure 2, is a gradual decline for women as SDI increases, with a more rapid decline at the highest levels of SDI. For men, CVD mortality increases as regions move from the lowest to middle levels of the SDI, with a significant decrease in mortality only observed for Central and Eastern Europe, high-income regions, and in Central Asia during the past decade.

**Figure 3:**
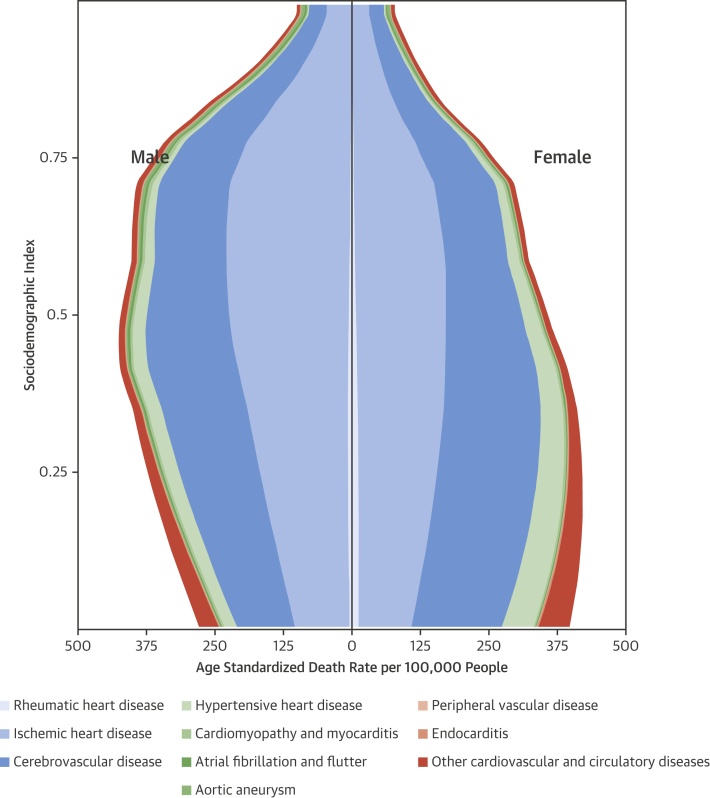
Relationship Between Age-Standardized Mortality Rate, CVD Cause, and SDI, by Sex This figure displays the distribution of age-standardized death rate by causes of CVD mortality by SDI. Abbreviations as in Figure 1.

The relationship between SDI and the age-standardized CVD death rate at the global level is shown in Figure 3. As SDI increases beyond 0.25, the highest CVD mortality rates shift from women to men. Cerebrovascular mortality rates begin to decline among women above an SDI of 0.3, although still increasing for men. IHD and cerebrovascular mortality rates continue to increase with greater SDI among men, peaking at an SDI of 0.5 to 0.75 (compared with 0.25 among women). CVD mortality decreases sharply for both sexes in countries with an SDI >0.75.

### Prevalence and mortality for CVD by cause groups

#### Ischemic heart disease

In 2015, IHD was the leading cause of all health loss globally, as well as in each world region (Online Figure 3). There were an estimated 7.29 million acute myocardial infarctions (95% UI: 6.80 to 7.81 million acute myocardial infarctions) and 110.55 million prevalent cases of IHD (95% UI: 100.68 to 121.80 million cases) in 2015. Prevalent cases of IHD began accounting for a large proportion of prevalent cases of CVD after 40 years of age, and the prevalence rose steeply with older age categories (Online Figure 4A). There were an estimated 10.88 million prevalent cases of IHD (95% UI: 8.82 to 13.25 million cases) among persons 50 to 54 years of age, which is more than 3-fold the number of cases for persons 40 to 44 years of age. The IHD prevalence rose from an estimated 290 cases per 100,000 (95% UI: 255 to 328 cases per 100,000) for those 40 to 44 years of age to 11,203 cases per 100,000 (95% UI: 9,610 to 13,178 cases per 100,000) for those 75 to 79 years of age, declining slightly for those 80 years of age and over to a rate of 9,700 cases per 100,000 (95% UI: 8,773 to 10,738 cases per 100,000).

**Figure 4:**
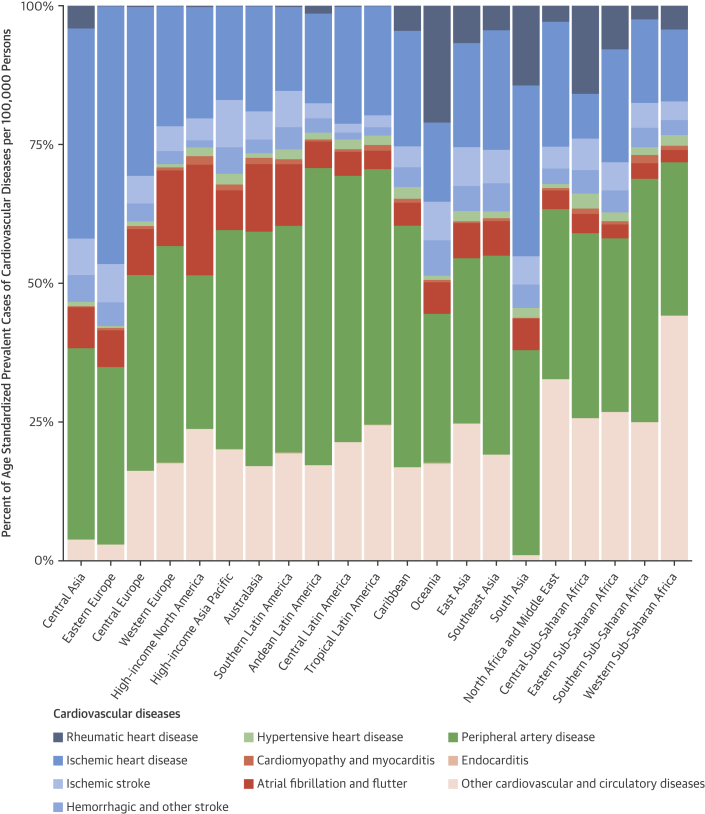
Percent of Age-Standardized Prevalent Cases per 100,000 for CVD Causes, 2015 This figure displays the relative distribution of age-standardized prevalence by CVD cause for 21 GBD world regions. Abbreviations as in Figure 1.

Eastern Europe had the highest estimated age-standardized prevalence of IHD in 2015 (4,140 cases per 100,000; 95% UI: 3,811 to 4,499 cases per 100,000), followed by Central Asia and then Central Europe (Online Figure 5A). IHD accounted for almost one-half of all CVD cases in Central Asia and Eastern Europe, but a smaller proportion in Central Europe, where other cardiovascular and circulatory diseases made up a larger proportion of total cases (Figure 4). Eastern sub-Saharan Africa, the Middle East/North Africa region, and South Asia all had similar estimated rates of just over 2,000 prevalent cases per 100,000. More than one-quarter of cases in sub-Saharan Africa were due to other cardiovascular and circulatory diseases. The lowest estimated age-standardized prevalence of IHD was in Central sub-Saharan Africa (622 per 100,000; 95% UI: 578 to 677 per 100,000), with similar low rates estimated in the southern Latin America and high-income Asia Pacific regions.

There were an estimated 8.92 million deaths (95% UI: 8.75 to 9.12 million deaths) due to IHD in 2015, making IHD the leading cause of death in the world. The death rate due to IHD rose steeply above age 40 years, increasing from an estimated 33 deaths per 100,000 (95% UI: 32 to 35 per 100,000) for those 40 to 44 years of age to 1,050 per 100,000 (95% UI: 1,025 to 1,076 per 100,000) by ages 75 to 79 years (Online Figure 4B). Above 80 years of age, the IHD death rate was estimated to be more than twice that rate (2,671 per 100,000; 95% UI: 2,600 to 2,738 per 100,000) and was by far the leading global cause of death.

**Figure 5:**
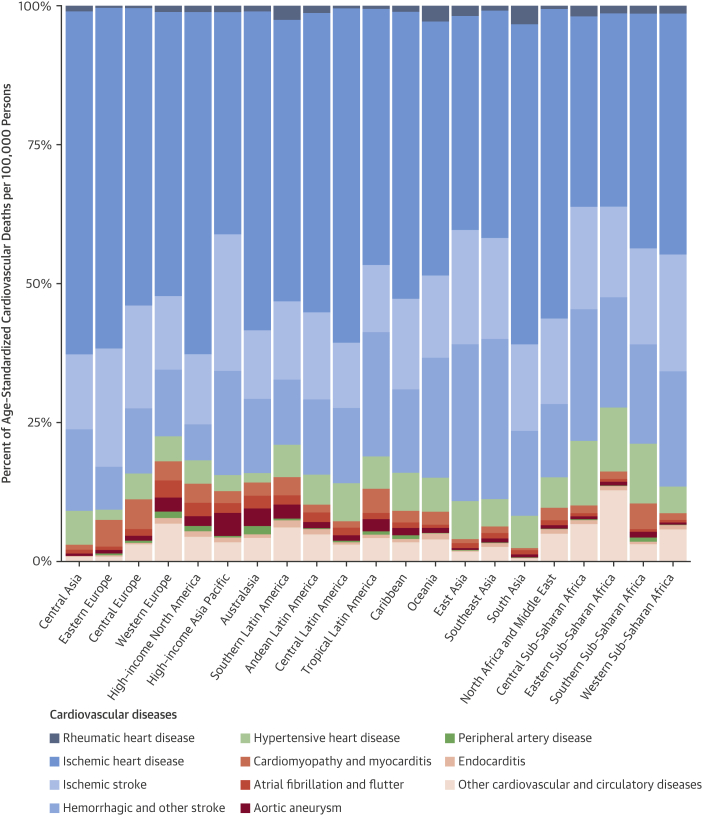
Percent of Age-Standardized Deaths per 100,000 for CVD Causes, 2015 This figure displays the relative distribution of age-standardized prevalence by CVD cause for 21 GBD world regions. Abbreviations as in Figure 1.

The estimated age-standardized IHD death rate was highest in Central Asia (336 per 100,000; 95% UI: 326 to 347 per 100,000) and Eastern Europe (326 per 100,000; 95% UI: 319 to 333 per 100,000), followed by Oceania, South Asia, and the Middle East/North Africa (Table 3). These regions, as well as high-income North America and Latin America, had particularly high proportions of total CVD deaths that were due to IHD (Figure 5). The estimated age-standardized IHD death rate was similar, from 100 to 150 deaths per 100,000, across a wide range of regions including high-income North America, Southeast and East Asia, and the Caribbean. The high-income Asia Pacific region had a much lower estimated age-standardized IHD death rate than any other region (45 per 100,000; 95% UI: 44 to 46 per 100,000) (Online Figure 6A).

**Table 3:** Global and Regional All-Age Prevalence and Age-Standardized Prevalence Rates in 2015, by Sex, for Selected Causes of CVD Mortality∗

	All-Age Prevalence			Age-Standardized Rate (per 100,000)		
	Total	Female	Male	Total	Female	Male
Cardiovascular diseases						
Global	422,738,396 (415,534,458–427,870,820)	205,821,777 (202,134,194–208,585,279)	216,916,608 (213,418,653–219,875,383)	6,304 (6,196–6,383)	5,812 (5,711–5,892)	6,833 (6,717–6,913)
Andean Latin America	2,527,168 (2,440,089–2,632,508)	1,172,826 (1,114,050–1,250,584)	1,354,342 (1,295,192–1,433,551)	5,666 (5,472–5,913)	5,049 (4,798–5,397)	6,330 (6,052–6,719)
Australasia	1,761,024 (1,697,613–1,854,157)	806,618 (770,407–861,873)	954,406 (911,026–1,015,916)	4,472 (4,305–4,705)	3,816 (3,637–4,093)	5,171 (4,938–5,496)
Caribbean	2,822,711 (2,751,853–2,904,703)	1,374,060 (1,325,923–1,427,497)	1,448,651 (1,404,659–1,502,391)	6,477 (6,314–6,664)	5,943 (5,731–6,178)	7,071 (6,859–7,335)
Central Asia	4,555,309 (4,461,547–4,654,438)	2,356,480 (2,299,260–2,433,326)	2,198,828 (2,137,653–2,268,905)	7,147 (6,989–7,309)	6,576 (6,413–6,787)	7,880 (7,675–8,107)
Central Europe	13,560,336 (13,304,071–13,830,352)	6,901,529 (6,747,945–7,104,379)	6,658,807 (6,512,843–6,843,977)	7,552 (7,418–7,704)	6,610 (6,465–6,798)	8,656 (8,480–8,886)
Central Latin America	11,826,535 (11,582,577–12,074,503)	5,897,220 (5,737,257–6,055,563)	5,929,315 (5,785,197–6,072,577)	6,225 (6,095–6,359)	5,842 (5,685–6,007)	6,653 (6,502–6,813)
Central sub-Saharan Africa	3,570,609 (3,420,593–3,723,098)	1,923,654 (1,820,220–2,025,073)	1,646,955 (1,545,442–1,747,781)	6,852 (6,590–7,144)	7,070 (6,672–7,494)	6,621 (6,321–7,010)
East Asia	93,345,590 (91,674,478–95,227,641)	43,135,337 (42,131,579–44,032,727)	50,210,253 (49,254,116–51,093,487)	6,146 (6,019–6,259)	5,543 (5,409–5,684)	6,725 (6,594–6,848)
Eastern Europe	23,898,622 (23,231,343–24,726,919)	13,142,602 (12,664,583–13,765,752)	10,756,019 (10,338,916–11,263,598)	7,482 (7,270–7,739)	6,441 (6,192–6,736)	8,982 (8,648–9,382)
Eastern sub-Saharan Africa	13,924,061 (13,569,314–14,266,069)	7,357,728 (7,135,795–7,570,055)	6,566,333 (6,356,066–6,795,777)	8,444 (8,237–8,654)	8,542 (8,290–8,806)	8,317 (8,084–8,624)
High-income Asia Pacific	13,619,192 (13,383,086–13,853,346)	6,987,560 (6,835,073–7,141,021)	6,631,632 (6,506,293–6,750,103)	3,831 (3,756–3,900)	3,433 (3,355–3,515)	4,268 (4,182–4,344)
High-income North America	26,512,238 (26,323,905–26,683,623)	12,993,521 (12,883,697–13,104,758)	13,518,717 (13,407,429–13,637,389)	5,302 (5,268–5,338)	4,732 (4,693–4,776)	5,953 (5,906–6,003)
North Africa and Middle East	31,044,596 (30,460,856–31,635,075)	14,248,387 (13,908,987–14,665,663)	16,796,211 (16,442,389–17,219,725)	8,017 (7,880–8,150)	7,425 (7,263–7,609)	8,627 (8,461–8,820)
Oceania	515,811 (499,389–532,290)	256,344 (245,973–265,385)	259,468 (248,274–271,865)	7,057 (6,866–7,253)	6,908 (6,646–7,118)	7,243 (7,006–7,539)
South Asia	71,649,779 (69,838,392–73,308,893)	33,572,668 (32,670,754–34,519,966)	38,077,110 (37,050,891–39,261,090)	6,006 (5,854–6,120)	5,567 (5,420–5,700)	6,460 (6,313–6,605)
Southeast Asia	31,811,965 (31,108,918–32,506,116)	15,689,372 (15,295,087–16,147,415)	16,122,592 (15,672,125–16,657,696)	6,293 (6,153–6,427)	5,860 (5,714–6,040)	6,772 (6,595–6,992)
Southern Latin America	2,907,935 (2,789,462–3,052,480)	1,514,205 (1,432,185–1,625,489)	1,393,730 (1,326,067–1,486,534)	4,092 (3,921–4,305)	3,668 (3,459–3,962)	4,631 (4,409–4,935)
Southern sub-Saharan Africa	3,451,145 (3,361,155–3,547,451)	1,891,089 (1,841,419–1,949,982)	1,560,056 (1,508,904–1,619,296)	7,668 (7,495–7,844)	7,386 (7,205–7,600)	7,936 (7,698–8,188)
Tropical Latin America	15,369,478 (15,061,429–15,652,622)	7,474,127 (7,279,837–7,700,746)	7,895,350 (7,725,078–8,091,713)	8,102 (7,946–8,252)	7,233 (7,038–7,445)	9,110 (8,922–9,328)
Western Europe	38,123,584 (37,400,667–38,901,652)	18,947,709 (18,414,908–19,464,239)	19,175,875 (18,772,625–19,677,426)	5,106 (5,005–5,214)	4,479 (4,355–4,607)	5,778 (5,649–5,927)
Western sub-Saharan Africa	15,940,704 (15,547,008–16,301,023)	8,178,745 (7,950,866–8,396,914)	7,761,959 (7,528,316–8,000,790)	9,475 (9,269–9,679)	9,753 (9,489–9,996)	9,179 (8,938–9,423)
Ischemic heart disease						
Global	110,550,305 (100,675,890–121,798,660)	46,102,255 (42,134,035–50,800,684)	64,448,047 (58,685,079–71,017,092)	1,663 (1,519–1,828)	1,312 (1,198–1,447)	2,045 (1,864–2,251)
Andean Latin America	433,378 (392,679–480,935)	191,058 (172,479–211,737)	242,320 (218,848–270,674)	999 (904–1,110)	836 (755–928)	1,179 (1,064–1,318)
Australasia	374,391 (356,154–394,637)	141,494 (133,557–150,318)	232,897 (220,558–245,473)	951 (903–1,003)	659 (620–703)	1,261 (1,193–1,328)
Caribbean	649,913 (594,603–712,186)	291,676 (266,641–320,463)	358,237 (326,370–393,695)	1,499 (1,367–1,645)	1,265 (1,155–1,391)	1,763 (1,609–1,933)
Central Asia	1,959,856 (1,792,534–2,148,360)	938,381 (850,809–1,037,029)	1,021,475 (934,886–1,115,021)	3,158 (2,886–3,464)	2,683 (2,432–2,970)	3,778 (3,473–4,102)
Central Europe	4,887,250 (4,421,084–5,396,339)	2,054,737 (1,855,428–2,273,925)	2,832,513 (2,556,599–3,130,360)	2,723 (2,462–3,006)	1,977 (1,786–2,192)	3,634 (3,292–4,009)
Central Latin America	2,743,458 (2,506,477–3,022,604)	1,314,991 (1,201,625–1,444,890)	1,428,466 (1,302,990–1,579,648)	1,465 (1,341–1,617)	1,313 (1,200–1,449)	1,640 (1,499–1,811)
Central sub-Saharan Africa	262,008 (242,179–285,446)	116,600 (107,838–126,833)	145,408 (133,789–159,226)	622 (577–677)	533 (492–577)	721 (666–787)
East Asia	20,261,326 (18,299,731–22,520,859)	8,319,850 (7,520,800–9,229,590)	11,941,475 (10,714,137–13,265,553)	1,276 (1,158–1,414)	1,046 (945–1,158)	1,511 (1,368–1,674)
Eastern Europe	13,228,694 (12,169,855–14,388,371)	6,092,839 (5,547,898–6,669,667)	7,135,855 (6,530,196–7,789,228)	4,140 (3,812–4,499)	2,973 (2,710–3,261)	5,903 (5,419–6,416)
Eastern sub-Saharan Africa	3,098,872 (2,735,642–3,532,901)	1,264,989 (1,131,992–1,434,792)	1,833,883 (1,598,281–2,126,672)	2,060 (1,812–2,348)	1,607 (1,431–1,821)	2,578 (2,229–2,961)
High-income Asia Pacific	2,596,645 (2,457,585–2,752,645)	1,216,369 (1,151,727–1,286,526)	1,380,276 (1,300,678–1,469,956)	708 (669–752)	562 (531–598)	873 (824–928)
High-income North America	6,278,342 (6,098,359–6,483,078)	2,789,029 (2,708,288–2,870,653)	3,489,312 (3,371,951–3,618,277)	1,226 (1,191–1,267)	972 (945–1,000)	1,517 (1,466–1,572)
North Africa and Middle East	7,072,468 (6,265,905–8,007,140)	2,975,936 (2,637,853–3,378,580)	4,096,532 (3,629,716–4,639,947)	2,055 (1,825–2,315)	1,705 (1,505–1,931)	2,424 (2,156–2,723)
Oceania	62,813 (56,704–69,835)	27,472 (24,802–30,653)	35,341 (31,670–39,554)	1,103 (995–1,226)	947 (858–1,057)	1,269 (1,141–1,411)
South Asia	23,148,018 (20,930,975–25,698,774)	8,781,644 (7,924,913–9,746,863)	14,366,374 (12,946,829–16,030,805)	2,052 (1,861–2,271)	1,576 (1,423–1,750)	2,533 (2,290–2,808)
Southeast Asia	7,471,104 (6,551,421–8,655,836)	3,246,055 (2,871,795–3,703,755)	4,225,049 (3,637,737–4,990,540)	1,520 (1,339–1,742)	1,261 (1,116–1,442)	1,801 (1,571–2,089)
Southern Latin America	488,329 (458,416–516,912)	178,843 (167,464–189,854)	309,486 (289,061–330,028)	690 (647–731)	425 (398–451)	1,021 (954–1,088)
Southern sub-Saharan Africa	653,589 (584,539–736,284)	283,992 (255,828–318,332)	369,597 (328,378–419,248)	1,415 (1,264–1,595)	1,091 (981–1,230)	1,826 (1,627–2,065)
Tropical Latin America	3,480,148 (3,159,912–3,841,271)	1,414,323 (1,296,129–1,549,721)	2,065,825 (1,858,218–2,300,500)	1,830 (1,666–2,018)	1,370 (1,258–1,507)	2,384 (2,155–2,641)
Western Europe	9,054,333 (8,208,626–10,087,602)	3,670,540 (3,286,094–4,154,807)	5,383,793 (4,897,589–5,957,280)	1,244 (1,125–1,388)	892 (793–1,012)	1,634 (1,485–1,809)
Western sub-Saharan Africa	2,345,370 (2,057,647–2,677,442)	791,437 (707,199–894,197)	1,553,933 (1,341,625–1,795,108)	1,513 (1,327–1,724)	1,038 (928–1,172)	2,024 (1,759–2,321)
Ischemic strokes						
Global	24,929,026 (24,362,226–25,609,959)	11,902,196 (11,625,159–12,233,046)	13,026,829 (12,718,240–13,382,922)	376 (367–386)	340 (331–349)	416 (406–428)
Andean Latin America	72,045 (69,967–74,245)	37,542 (36,413–38,744)	34,503 (33,492–35,599)	165 (160–170)	163 (158–168)	167 (162–173)
Australasia	97,732 (95,064–100,797)	48,401 (46,706–50,165)	49,331 (47,986–50,972)	252 (245–260)	235 (227–243)	271 (263–280)
Caribbean	117,888 (114,875–121,193)	59,348 (57,746–61,088)	58,539 (56,879–60,294)	274 (267–282)	259 (252–267)	290 (281–299)
Central Asia	344,977 (337,341–353,209)	189,937 (185,553–194,749)	155,040 (151,404–159,070)	552 (539–566)	535 (522–549)	575 (560–590)
Central Europe	796,434 (775,591–820,957)	424,906 (412,679–439,291)	371,527 (361,686–382,321)	445 (434–458)	415 (404–429)	483 (470–497)
Central Latin America	194,157 (187,314–201,435)	99,012 (95,566–102,898)	95,145 (91,835–98,817)	105 (102–110)	100 (97–104)	111 (107–115)
Central sub-Saharan Africa	187,635 (182,346–192,989)	107,722 (104,532–111,025)	79,912 (77,566–82,174)	440 (427–453)	476 (461–492)	394 (382–407)
East Asia	7,423,862 (7,228,513–7,652,139)	3,161,924 (3,077,657–3,259,358)	4,261,938 (4,146,738–4,395,442)	474 (461–490)	403 (392–416)	548 (532–566)
Eastern Europe	1,939,626 (1,886,897–1,993,437)	1,201,817 (1,169,100–1,236,376)	737,809 (713,336–763,066)	614 (597–630)	603 (588–620)	625 (604–647)
Eastern sub-Saharan Africa	772,966 (750,967–795,839)	418,379 (406,070–431,313)	354,587 (344,928–364,964)	506 (490–523)	520 (503–538)	489 (473–505)
High-income Asia Pacific	1,193,488 (1,162,144–1,229,286)	530,971 (516,893–546,687)	662,517 (645,074–683,051)	358 (350–369)	294 (287–303)	434 (423–447)
High-income North America	1,167,328 (1,133,548–1,205,267)	581,089 (563,548–599,833)	586,239 (569,088–605,367)	236 (229–244)	216 (209–223)	259 (252–268)
North Africa and Middle East	1,312,093 (1,285,007–1,341,997)	672,113 (658,117–688,072)	639,980 (626,008–654,639)	355 (347–364)	357 (349–366)	353 (344–362)
Oceania	32,854 (31,947–33,759)	16,173 (15,656–16,722)	16,681 (16,256–17,125)	535 (519–551)	504 (488–523)	570 (554–587)
South Asia	3,932,151 (3,831,984–4,047,704)	1,714,307 (1,667,742–1,765,753)	2,217,843 (2,160,750–2,281,985)	333 (323–343)	289 (280–298)	377 (367–390)
Southeast Asia	2,106,975 (2,059,929–2,157,906)	1,011,731 (986,323–1,038,279)	1,095,244 (1,068,060–1,121,515)	424 (413–435)	383 (373–394)	470 (457–482)
Southern Latin America	206,767 (200,751–213,651)	107,942 (104,638–111,837)	98,824 (95,589–102,483)	296 (287–306)	275 (266–285)	324 (313–336)
Southern sub-Saharan Africa	190,018 (185,419–195,150)	103,090 (100,211–106,287)	86,928 (84,803–89,122)	419 (408–431)	396 (384–410)	444 (432–456)
Tropical Latin America	356,275 (338,561–375,658)	172,946 (164,866–182,222)	183,329 (173,927–194,059)	193 (183–203)	169 (161–179)	221 (210–235)
Western Europe	1,865,822 (1,817,012–1,917,658)	924,111 (899,640–951,652)	941,712 (915,999–969,970)	255 (248–262)	227 (222–234)	286 (278–294)
Western sub-Saharan Africa	617,935 (603,712–633,755)	318,735 (310,971–327,448)	299,200 (292,051–306,700)	388 (378–399)	397 (386–410)	377 (367–387)
Hemorrhagic and other strokes						
Global	18,669,622 (18,258,729–19,124,482)	8,900,797 (8,700,081–9,121,277)	9,768,824 (9,543,517–10,012,685)	268 (262–275)	249 (243–255)	288 (282–296)
Andean Latin America	82,248 (79,877–84,631)	44,824 (43,468–46,162)	37,424 (36,298–38,588)	164 (160–169)	174 (169–179)	155 (150–159)
Australasia	46,455 (45,253–47,851)	25,097 (24,270–25,923)	21,358 (20,810–22,010)	124 (121–128)	129 (125–133)	119 (116–123)
Caribbean	114,404 (111,412–117,288)	60,894 (59,271–62,556)	53,511 (52,084–54,945)	258 (251–264)	266 (258–273)	250 (243–257)
Central Asia	285,031 (279,091–291,260)	155,973 (152,515–159,672)	129,058 (126,281–131,955)	404 (395–413)	402 (393–412)	407 (398–416)
Central Europe	482,420 (470,995–495,973)	278,486 (271,057–287,367)	203,934 (199,058–209,297)	299 (292–307)	319 (310–329)	279 (272–286)
Central Latin America	180,268 (173,792–187,229)	93,592 (90,155–97,387)	86,676 (83,670–90,019)	86 (83–89)	85 (82–89)	87 (84–90)
Central sub-Saharan Africa	181,821 (177,249–186,526)	105,226 (102,578–108,171)	76,596 (74,630–78,729)	332 (323–340)	372 (362–382)	285 (278–293)
East Asia	4,933,114 (4,809,787–5,071,809)	2,009,705 (1,958,387–2,067,784)	2,923,409 (2,850,909–3,004,011)	307 (299–316)	254 (248–262)	360 (350–370)
Eastern Europe	1,129,261 (1,103,084–1,156,455)	683,094 (666,554–701,155)	446,168 (433,515–459,717)	377 (369–386)	379 (370–389)	377 (367–389)
Eastern sub-Saharan Africa	840,950 (816,966–866,461)	461,124 (448,039–475,939)	379,825 (368,337–392,136)	405 (395–417)	433 (422–446)	373 (363–383)
High-income Asia Pacific	587,292 (572,980–603,859)	294,104 (286,904–302,836)	293,188 (285,828–301,761)	202 (197–208)	194 (189–200)	213 (207–219)
High-income North America	403,245 (391,490–416,047)	208,999 (202,897–215,686)	194,246 (188,361–200,619)	85 (82–88)	82 (79–85)	88 (85–91)
North Africa and Middle East	1,093,535 (1,069,924–1,119,497)	594,477 (581,671–608,840)	499,059 (487,623–511,343)	253 (248–259)	275 (269–281)	232 (227–237)
Oceania	38,961 (38,070–39,939)	20,324 (19,764–20,923)	18,637 (18,175–19,116)	493 (481–505)	507 (492–522)	479 (468–492)
South Asia	3,970,983 (3,865,439–4,086,075)	1,734,618 (1,687,573–1,786,688)	2,236,364 (2,178,258–2,300,244)	286 (279–294)	254 (247–261)	318 (310–327)
Southeast Asia	2,040,033 (1,995,560–2,086,665)	973,642 (950,221–998,350)	1,066,391 (1,041,873–1,090,836)	357 (350–365)	331 (323–339)	385 (376–394)
Southern Latin America	126,534 (122,934–130,607)	68,749 (66,763–71,058)	57,786 (56,064–59,706)	185 (180–191)	186 (181–192)	186 (180–192)
Southern sub-Saharan Africa	186,863 (182,132–192,225)	95,226 (92,741–98,041)	91,637 (89,262–94,206)	332 (324–341)	316 (308–325)	349 (341–358)
Tropical Latin America	292,725 (279,182–307,749)	149,531 (142,631–157,232)	143,194 (136,362–150,570)	146 (139–153)	138 (132–146)	156 (149–164)
Western Europe	926,435 (903,480–950,683)	482,432 (470,375–495,455)	444,003 (432,460–456,481)	141 (137–145)	138 (135–142)	145 (141–149)
Western sub-Saharan Africa	727,043 (709,295–746,947)	360,683 (352,133–370,683)	366,360 (356,969–377,288)	323 (316–331)	332 (324–340)	312 (305–320)

Values are n (95% uncertainty intervals).

∗ Results for all causes are available online via the GBD Compare web visualization tool (23).

#### Stroke

Globally, hemorrhagic and other strokes, and ischemic strokes were the second- and third-largest CVD causes of DALYs in 2015, and the 4th- and 13th-largest overall, respectively. Ischemic stroke DALY outranked hemorrhagic and other strokes only in Central and Eastern Europe and high-income North America. There were an estimated 5.39 million acute first-ever ischemic strokes (95% UI: 5.02 to 5.73 million), 3.58 million acute first-ever hemorrhagic and other strokes (95% UI: 3.34 to 3.82 million), and 42.43 million prevalent cases of cerebrovascular disease (95% UI: 42.07 to 42.77 million) overall in 2015. The prevalence of stroke began increasing above 40 years of age, reaching the highest rate for any 5-year age group for those 74 to 79 years of age (4,201 cases per 100,000; 95% UI: 4,140 to 4,258 per 100,000), with the rate declining by one-half this amount among those over 80 years of age.

Oceania was the region with the highest prevalence of stroke (1,003 per 100,000; 95% UI: 985 to 1,025 per 100,000), followed by Eastern Europe, Central Asia, and Southeast Asia. The lowest stroke prevalence in the world, less than one-fifth that of the highest regions, was in Central Latin America (177 per 100,000; 95% UI: 174 to 180 per 100,000). The estimated age-standardized prevalence of ischemic stroke was greater than that of hemorrhagic stroke in most regions, but was the same for Andean Latin America.

There were 6.33 million deaths due to stroke in 2015 (95% UI: 6.18 to 6.49 million deaths), with 57% of these stroke deaths due to ischemic stroke. The stroke mortality rate began rising above 50 years of age, increasing for each older age group until it reached 1,812 per 100,000 (95% UI: 1,764 to 1,864 per 100,000) among those over age 80 years.

The estimated stroke age-standardized mortality rate in 2015 was also greatest in Oceania (191 per 100,000; 95% UI: 148 to 248 per 100,000), followed by Central sub-Saharan Africa. The lowest estimated rates were in high-income North America, Australasia, Western Europe, Andean Latin America, high-income Asia Pacific, Central Latin America, and southern Latin America. The ischemic stroke death rate dominated the hemorrhagic stroke death rate in Eastern and Central Europe, Middle East/North Africa, the Caribbean, and Southern Latin America, with similar rates found in Western sub-Saharan Africa, high-income North America, Australasia, South Asia, and Western Europe.

#### Hypertensive heart disease

Hypertensive heart disease was the fourth-highest ranked CVD cause for DALYs in 2015 globally; however, it ranked lower in high-income regions such as Australasia, high-income Asia Pacific, and Western Europe. There were 6.09 million (95% UI: 5.73 to 6.43 million) prevalent cases of hypertensive heart disease in 2015. The prevalence rose continuously for each age group, from 2.0 per 100,000 (95% UI: 1.7 to 2.1 per 100,000) at ages 20 to 24 years until reaching 1,360 per 100,000 (95% UI: 1,248 to 1,502 per 100,000) for those >80 years of age. The prevalence was highest in Western sub-Saharan Africa, followed by Central and Eastern sub-Saharan Africa, tropical Latin America, and the Caribbean. The lowest rates were estimated for Western and Eastern Europe.

There were 962,400 deaths (95% UI: 873,600 to 1,024,500 deaths) due to hypertensive heart disease in 2015. The mortality rate rose for ages >60 years, peaking at 296 per 100,000 (95% UI: 257 to 315 per 100,000) for age >80 years. Death rates due to hypertensive heart disease followed a similar pattern as the condition's prevalence.

#### Cardiomyopathy

Cardiomyopathies and acute myocarditis were a higher-ranked cause of CVD DALYs in the regions of Central and Eastern Europe than in other world regions. There were an estimated 2.54 million (95% UI: 2.41 to 2.66 million) prevalent cases of cardiomyopathy and myocarditis in 2015. There was a slightly higher prevalence among children 1 to 4 years of age (29 per 100,000; 95% UI: 26 to 32 per 100,000), which then decreased for older children, increasing slowly throughout adulthood. A very large increase in cases of cardiomyopathy and acute myocarditis was estimated above 80 years of age (628 per 100,000; 95% UI: 569 to 692 per 100,000), more than 6-fold higher than for the next youngest age group of 75 to 79 years of age. This condition accounted for a relatively small proportion of CVD cases overall, with the greatest age-standardized prevalence estimated for Southern sub-Saharan Africa, followed by tropical Latin America, high-income North America, and other regions of sub-Saharan Africa.

There were 353,700 (95% UI: 339,500 to 370,600) deaths due to cardiomyopathy and myocarditis in 2015. The mortality rate was as high as 47 per 100,000 persons (95% UI: 33 to 59 per 100,000) within the first week of life. The mortality rate decreased by 5 years of age, and then increased steadily throughout adulthood before increasing more than 300% after 80 years of age, to a peak rate of 90 per 100,000 (95% UI: 85 to 94 per 100,000). CVD deaths due to cardiomyopathy and myocarditis were most common in regions with high prevalence, including Southern sub-Saharan Africa and tropical Latin America.

#### Aortic aneurysm

Aortic aneurysm was the fifth leading cause of CVD DALYs in the high-income Asia Pacific region, but ranked lower in other world regions. Globally, there were an estimated 168,200 deaths (95% UI: 163,500 to 172,800 deaths) due to aortic aneurysm in 2015. The age-specific mortality rate due to aortic aneurysm rose most quickly after 60 years of age, reaching a peak of 50 per 100,000 (95% UI: 48 to 53 per 100,000) above 80 years of age. Tropical Latin America had the highest death rate due to aortic aneurysm, followed by Southern Latin America, high-income Asia Pacific, Australasia, and Oceania. Death rates due to aortic aneurysm were lowest in Western sub-Saharan Africa and East Asia.

#### Atrial fibrillation

Atrial fibrillation ranked higher as a CVD cause of DALYs for Western Europe (fifth), Australasia (sixth), and South Asia (sixth). Globally there were an estimated 33.3 million (95% UI: 30.0 to 37.2 million) prevalent cases of atrial fibrillation in 2015. The age-specific prevalence of atrial fibrillation increased steadily for each 5-year age group above 30 years of age, reaching a rate of 5,544 per 100,000 (95% UI: 4,863 to 6,307 per 100,000) above 80 years of age. Age-standardized prevalence was highest in high-income North America (1,224 per 100,000; 95% UI: 1,157 to 1,297 per 100,000), followed by Western and Central Europe.

There were an estimated 195,300 (95% UI: 159,519 to 236,176) deaths due to atrial fibrillation in 2015. This death rate was almost 5× higher, 116 per 100,000 (95% UI: 90 to 145 per 100,000) above 80 years of age than below that age. Atrial fibrillation mortality rates were highest in Western and Central Europe and high-income North America, and lowest in sub-Saharan Africa.

#### Rheumatic heart disease

RHD was the fifth highest-ranked CVD cause of DALYs for South Asia and Central Asia and the sixth highest-ranked globally, although it was among the lowest-ranked causes in high-income regions. It accounted for substantially larger proportions of prevalent CVD and mortality in Oceania, sub-Saharan Africa, South Asia, the Caribbean, and Central Asia compared with other regions.

#### Endocarditis

Although a relatively less-common cause of CVD, endocarditis DALYs were greater in sub-Saharan Africa compared with other regions. There were an estimated 115,700 prevalent cases of endocarditis in 2015 (95% UI: 108,000 to 125,000 cases). There was an increase and then decline in the mortality rate among children, which peaked among children 28 days to 1 year of age (0.22 cases per 100,000; 95% UI: 0.18 to 0.26 per 100,000). The prevalence rose quickly above 60 years of age, reaching 28 per 100,000 (95% UI: 24 to 34 per 100,000) above 80 years of age. The highest prevalence of endocarditis was estimated for Oceania, 14 per 100,000 (95% UI: 13 to 15 per 100,000), which was twice the rate of the next-highest region, Southern Latin America. The lowest rates were estimated for East Asia and Andean Latin America.

There were 84,900 deaths due to endocarditis in 2015. Mortality rates were highest at the extremes of the age range: 7 per 100,000 (95% UI: 4 to 9 per 100,000) among those in the first week of life; 8 per 100,000 (95% UI: 7 to 9 per 100,000) among those 75 to 79 years of age; and 22 per 100,000 (95% UI 21 to 24 per 100,000) older than 80 years of age. Age-standardized mortality rates were greatest in Oceania, 6 per 100,000 (95% UI: 4 to 8 per 100,000), more than twice that of the next 2 regions, Central sub-Saharan Africa and Middle East/North Africa.

#### Peripheral arterial disease

PAD was among the lowest-ranked CVD cause of DALYs in most world regions, but accounted for the largest proportion of cases of prevalent CVD in most world regions, reflecting high estimates of prevalence (based on surveys using ABI) and low estimates of symptomatic PAD (based on self-reported claudication among those with reduced ABI) and PAD death. There were an estimated 154.7 million (95% UI: 136.3 to 176.2 million) cases of PAD in 2015. The age-specific prevalence increased steadily after 40 years of age, reaching 23,913 per 100,000 (95% UI: 20,555 to 27,853 per 100,000) after 80 years of age. Age-standardized PAD prevalence was as high as 4,286 per 100,000 (95% UI: 3,773 to 4,897 per 100,000) in tropical Latin America, followed by Southern sub-Saharan Africa. The lowest prevalence of PAD, less than one-half of that rate, was estimated for high-income Asia Pacific and high-income North America.

There were 52,500 deaths (95% UI: 49,700 to 55,700 deaths) due to PAD in 2015. Death rates were highest above 80 years of age (24 per 100,000; 95% UI: 22 to 26 per 100,000). The Southern sub-Saharan region had the greatest age-standardized mortality rate, 3 per 100,000 (95% UI: 2 to 3 per 100,000), followed by Australasia, Eastern Europe, and the Caribbean, whereas the lowest mortality rates were estimated for Western sub-Saharan Africa, Andean Latin America, and Southeast Asia.

## Discussion

CVD accounted for one-third of all deaths in 2015, and there were an estimated 422 million prevalent cases. The prevalence of CVD varied widely among countries, and when age-standardized, was declining in many high-income countries. Our analysis of mortality and sociodemographic change demonstrates a global disease gradient dominated by atherosclerotic vascular diseases, such as IHD and stroke, and with the most rapid decline occurring only at the highest levels of development. An alarming finding is that trends in CVD mortality have plateaued and are no longer declining for high-income regions. Overall, these results demonstrate the importance of increased investment in prevention and treatment of CVD for all regions of the world.

It is notable that very high age-standardized death rates due to CVD were not limited to any single region of the world, occurring among a subset of countries throughout Eastern Europe, Central Asia, the Middle East, South America, sub-Saharan Africa, and Oceania. There were also extremely large differences in estimated country-level age-standardized prevalence of CVD, ranging from <4,000 to >11,000 prevalent cases per 100,000 persons in 2015.

### Sociodemographic transition

Our regional analysis of SDI over time shows that Oceania, Central and Eastern Europe, Central Asia, and high-income North America have experienced levels of CVD far higher than would be expected given global patterns of disease. Over the same time period, tropical Latin America and the Middle East/North Africa have experienced steady declines during periods of continuous socioeconomic development. Western sub-Saharan Africa and other areas of Latin America have maintained levels well below regions with similar SDI levels. No decline at all is estimated for South and Southeast Asia. Recent continuous and rapid decline in CVD mortality remains a phenomenon limited to only a subset of high-SDI regions. Regional differences in CVD are likely a result of variation in exposure to modifiable risk factors, as well as access to effective health care interventions 24, 25, 26, 27, 28.

### Changes in the decline of CVD mortality

Of particular concern is that CVD age-standardized mortality shows less decline in the past 5 years than over the past 25 years. This trend, which is most obvious for IHD and aortic aneurysm, is observed not only in high-income countries, but also in Central Latin America for men. Regions with very high rates of CVD that have declined rapidly, such as Central Asia and Eastern Europe, also see moderation in that decline. Our use of the most recently available mortality data (through 2013 in many high-income countries) may explain why our findings differ from a recent analysis of CVD trends (29). Although an explanation of stagnation in declining CVD mortality is beyond the scope of this analysis, several possibilities can be considered. Rising rates of obesity may be increasing CVD risk over a short period of time (30). Interventions that reduce CVD mortality rates may have maximally diffused to the population able to access them, whereas interventions to address obesity are more challenging to implement. Some CVD risk factors, in particular air pollution or changes in average temperature, may account for larger increases of CVD mortality than previously suspected 31, 32. Improving methods for estimating the most likely future trajectories for CVD is an important area for further research.

### Discontinuities in cardiovascular mortality

Any broad conclusions on the global influence of socioeconomic development must be tempered by the fact that rapid increases in CVD burden have occurred due to a diverse and evolving set of health risks. Economic crises in Eastern Europe in the 1990s and the resulting rapid changes in CVD mortality rates have only been partially described and require more investigation. There is strong evidence that the hazardous use of alcohol was a major contributor to this pattern 33, 34. South Africa experienced a similar mortality crisis, peaking around the year 2000 due to “colliding” epidemics of human immunodeficiency virus/acquired immunodeficiency syndrome and noncommunicable diseases (35). These striking examples of abrupt discontinuities in the number of deaths due to CVD (“fatal discontinuities”) demonstrate how political and social unrest may lead not only to outbreaks of communicable disease, but also to dramatic changes in cardiovascular health. Further attention is needed to understand how CVD is influenced by and would be best treated during rapid changes in material living conditions caused by war, natural disasters, and mass migration from lower to higher SDI regions. Patterns of CVD during times of large-scale migration require particular attention, due to the potential unfavorable effects of immigration on atherosclerotic risk factors (when moving to higher-SDI regions) as well as in reducing access to basic health care (36).

### Cause-specific variation in the epidemiological transition

IHD and stroke mortality rates increase and then fall as SDI increases, supporting the theory of an epidemiological transition for CVD 37, 38. The initial increases in IHD and stroke mortality at low levels of SDI reflect the particularly high mortality rates estimated for Oceania, tropical Latin America, and the Middle East/North Africa region in the 1990s. Almost all other CVD burdens decrease continuously at higher levels of SDI. Declines in IHD and stroke mortality lag for men compared with women as SDI increases. Differences in CVD risk exposures may explain this differential by sex, such as exposure to tobacco smoking (39). The decline in stroke mortality among women at lower levels of SDI ranging from 0.3 to 0.6, driven by trends in South Asia, North Africa/Middle East, and Oceania, suggests that countries need not achieve the highest levels of development to reduce the burden of stroke. Results of the Prospective Urban Rural Epidemiological study show that women have lower use of the most common medications used to treat CVD compared with men, suggesting that this differential outcome is not the result of higher use of secondary medications among women (40).

### Peripheral arterial disease

We estimated that PAD is the most prevalent cardiovascular condition globally, although low estimated rates of claudication and mortality made it a minor contributor to DALYs. The high prevalence of PAD in comparison to IHD is a notable finding that may reflect the ease of its diagnosis using ABI, compared with more complex diagnostic testing required for IHD. Further attention should be paid to the use of ABI or palpation of foot pulses as a screening tool for overall vascular risk in low-income settings 41, 42. There is some evidence that even those with asymptomatic PAD would benefit significantly from inexpensive medications, such as an angiotensin-converting enzyme inhibitor or antiplatelet agents 43, 44.

### Stroke

We estimated that hemorrhagic and other stroke accounted for more CVD DALYs than ischemic stroke globally and for almost all regions. Although the predominance of hemorrhagic and other strokes over ischemic subtypes may reflect variable use of unspecified stroke ICD-10 codes in some regions, this ranking also results from the predominance of hemorrhagic and other stroke deaths over ischemic stroke deaths for all ages <75 years, even among high-income countries. Both stroke subtypes share a common set of modifiable risk factors, suggesting that public health and health system investments in their shared risks could reduce overall burden due to all stroke (45).

The GBD 2015 study offers improved access to its data sources, research methods, and results. Citations for all data used in the study can be found via the publicly available Global Health Data Exchange (18). The new GATHER (Guidelines for Accurate and Transparent Health Estimates Reporting) guidelines for reporting estimates of public health have been fully implemented (Online Appendix). Intermediate model results from DisMod and all final results have been made publicly available using web-based data visualization tools (46). Finally, software code and data tables have been posted online and are freely available for download (47).

### Study limitations

CVD estimates of the GBD 2015 study have several important limitations. Misclassification bias due to miscoding of death certificates remains likely 48, 49. The GBD study takes several steps to improve the reliability and comparability of vital registration data, including redistribution of garbage codes, but some systematic bias due to regional patterns in the use of diagnosis codes may remain. For example, the relatively large number of deaths coded to cardiomyopathy in Balkan countries may lead to an underestimate of the true number of IHD deaths. Many stroke deaths are coded to non–subtype-specific stroke codes, which, when combined with lack of access to computed tomography scanners to aid in the acute management of stroke, adds additional uncertainty to the breakdown of stroke into ischemic and hemorrhagic subtypes. Uncertainty regarding stroke subtype is of particular concern in South Asia, where no subtype-specific mortality data were found, and the global proportion of ischemic and hemorrhagic stroke was used instead. The rapid rise, globally, in death certificates coded to atrial fibrillation is implausible in the face of cohort data showing stable age-standardized prevalence and case-fatality rates, and led to our adopting a new natural history method for estimating its global burden that incorporates this cohort data. Estimation of the burden of atrial fibrillation is limited by variable coding of this condition as an underlying cause of death, especially in the setting of stroke, and very little data on its prevalence in low- and middle-income countries. Similar increases in PAD may also reflect changes in coding practice among physicians.

Health data on CVD remains extremely limited for some regions of the world, such as India and sub-Saharan Africa. In sub-Saharan Africa, a much larger proportion of prevalent cases of CVD were estimated to come from other cardiovascular and circulatory diseases. This category of other causes includes pericardial disease, nonrheumatic valvular diseases, unspecified lymphatic and vascular conditions, and pulmonary embolism, as well as deaths coded to left and right heart failure and other nonunderlying or nonspecific causes of death. Lack of data is 1 reason why no significant change in prevalence could be detected in many regions, for example, Western sub-Saharan Africa. There is only limited data available in some countries with both high all-cause mortality and high estimated CVD mortality. For example, estimates of CVD mortality in Afghanistan are based on a single verbal autopsy survey performed in 2008 that reported CVD as the cause of 14.4% of all male deaths and 20.7% of female deaths.

Data on the severity distribution of CVD is also particularly limited, and future iterations of GBD will benefit from additional sources from more regions. Limited data presents a special challenge in India, where GBD 2015 relied on 3 major sample vital registration sources for estimating mortality: Medical Certification of Causes of Death, primarily an urban-area program; the Survey of Causes of Death and Survey of Causes of Death-Rural; and the Sample Registration System. The latter source, in particular, has not been released in detail for specific causes of CVD. Expanded access to this kind of data in India and other countries that have invested in sample death registration systems could significantly improve our ability to forecast trends in atherosclerotic vascular diseases.

The GBD study has reported subnational estimates for a growing number of countries; however, the current report is limited to country-level estimates 50, 51, 52. There is substantial small-area variation in CVD burden within countries, and our national estimates represent only an average level for an entire country. Even these national estimates are important starting points for improving evidence available to policymakers.

The GBD study accounts for comorbidity using a simulation method that assumes an independent probability of having any disease state. A sensitivity analysis found that taking dependent and independent comorbidity into account changed overall estimated YLDs by a small amount that increased with age, ranging from 0.6% to 3.4%. Because CVD is more common at older ages, the assumption of independence may have a larger effect on this group of causes. Unfortunately, data on the full correlation structure of prevalent CVD conditions remains limited.

GBD includes an estimate of measurement error, reported as a 95% UI, for each result. Our ability to detect significant trends over time is limited for those regions where UIs are wide, as seen on our global map of change in prevalence over time. Some countries may have experienced a rise or fall in CVD burden that we cannot detect because of limited data.

Although inclusion of measurement error is a strength of the GBD study, nonsampling error has not been quantified. GBD uses a wide range of validation methods, but relies on in-sample and out-of-sample validity testing to guide model selection. Additional sources of error in the GBD study may include regional patterns of clinical diagnosis, death code redistribution, selection of data sources, covariate selection for selected models, and measurement of the SDI. For example, measures of wealth, rather than income per capita, could potentially capture additional aspects of the epidemiological transition for some countries. Limited data on PAD with increased ABI due to noncompressible arteries may lead to underestimates of its true burden. The burden due to Chagas cardiomyopathy has not been included, but is estimated by the GBD study as a sequelae of Chagas disease. Chronic kidney disease and congenital heart disease are also estimated by the GBD study and have been reported separately. Overall, the GBD study is most likely to underestimate uncertainty for those geographies where few data sources are available.

## Conclusions

CVD remain a major cause of premature death and chronic disability for all regions of the world. IHD and stroke account for the majority of health lost to CVD. Sociodemographic change over the past 25 years has been associated with dramatic declines in age-standardized rates of CVD mortality in regions with very high SDI, but only a gradual decrease or no change at all in most regions. It is concerning that large reductions in atherosclerotic vascular disease mortality, a crowning achievement for public health, are no longer apparent in many world regions, despite impressive advances in technical capacity for preventing and treating CVD.

The GBD study offers a unique platform for tracking rapidly evolving patterns in CVD epidemiology and their relationship to demographic and socioeconomic change. Specific causes of CVD can be examined within the broader context of global health. Countries should consider further investment in CVD surveillance and population-based registries to benchmark their efforts toward reducing the burden of CVD. Future updates of the GBD study can be used to guide policymakers who are focused on reducing the overall burden of noncommunicable disease and achieving specific global health targets.

Perspectives **COMPETENCY IN SYSTEMS-BASED PRACTICE:** Ischemic heart disease and stroke account for most of the global burden of cardiovascular disease, but there is wide regional variation. Improvements in mortality related to cardiovascular disease appear to be slowing for regions of the world characterized by a high sociodemographic index based on per capita income, educational attainment, and fertility.

**TRANSLATIONAL OUTLOOK:** Development of evidence-based public health policies requires incorporation of comparative health status and burden of disease estimates between and within countries.
