# Supplementary material for: Global, Regional, and National Burden of Cardiovascular Diseases for 10 Causes, 1990 to 2015
Source: J Am Coll Cardiol. 2017 Jul 4;70(1):1–25. doi: 10.1016/j.jacc.2017.04.052 (PMC5491406; doi:10.1016/j.jacc.2017.04.052)

Supplementary Results to Global, Regional, and National Burden of Cardiovascular Diseases for 10 Causes, 1990–2015

This document provides supplementary results for incidence, prevalence, and years of life lived with disability. Further methodological details can be found in the appendix document provided in a separate on-line file.

Supplementary Results: Tables & Figures

Tables:

eTable 1. Global and Regional YLLs for all CVD causes, total number and age-standardized rate, by sex and total, for 2015

eTable 2. Global and Regional YLDs for all CVD causes, total number and age-standardized rate, by sex and total, for 2015

eTable 3a. Prevalence of CVD Causes for Years 1990-2000, with Upper and Lower Uncertainty Bounds

eTable 3b. Prevalence of CVD Causes for Years 2005-2015, with Upper and Lower Uncertainty Bounds

eTable 4a. All-ages Prevalence for men and women separately and combined, for all CVD level 3 and 4 subcauses, by country in 1990 and 2015

eTable 4b. All-ages Mortality rate for men and women separately and combined, for all CVD level 3 and 4 subcauses, by country in 1990 and 2015

eTable 4c. All-ages DALY rate for men and women separately and combined, for all CVD level 3 and 4 subcauses, by country in 1990 and 2015

Figures:

eFigure 1a. Global country map, CVD data availability in GBD modeling, by sources for fatal modelling

eFigure 1b. Global country map, CVD data availability in GBD modeling, by sources for nonfatal modelling

eFigure 2a. Global country map, percentage change in age-standardized prevalence of CVD, 1990-2015

eFigure 2b. Global country map, percentage change in age-standardized death rate of CVD, 1990-2015

eFigure 3. Ranking of age-standardized DALYs per 100,000, both sexes combined, for CVD level 3 and 4 causes globally and for 21 GDB world regions.

eFigure 4a. Prevalence of CVD level 3 and 4 causes in 2015, by age categories

eFigure 4b. Death rate for CVD level 3 and 4 causes in 2015, by age categories.

eFigure 5a. Age-Standardized Prevalence of IHD per 100,000 persons in 2015, Both Sexes

eFigure 5b. Age-Standardized Prevalence of HHD per 100,000 persons in 2015, Both Sexes

eFigure 5c. Age-Standardized Prevalence of Ischemic Stroke per 100,000 persons in 2015, Both Sexes

eFigure 5d. Age-Standardized Prevalence of Hemorrhagic Stroke per 100,000 persons in 2015, Both Sexes

eFigure 6a. Age-Standardized Deaths due to IHD per 100,000 persons in 2015, Both Sexes

eFigure 6b. Age-Standardized Deaths due to HHD per 100,000 persons in 2015, Both Sexes

eFigure 6c. Age-Standardized Deaths due to Ischemic Stroke per 100,000 persons in 2015, Both Sexes
eFigure 6d. Age-Standardized Deaths due to Hemorrhagic Stroke per 100,000 persons in 2015, Both Sexes
eFigure 7a. Trends in Age-Standarized Incidence Rates for MI, 1990 – 2015
eFigure 7b. Trends in Age-Standarized Incidence Rates for Stroke, 1990 - 2015
eFigure 8a. Age-Standardized Prevelance of All Cardiovascular Disease per 100,000 persons in 2015, Female
eFigure 8b. Age-Standardized All Cardiovascular Disease, Deaths per 100,000 persons in 2015, Female
eFigure 9a. Age-Standardized Prevelance of All Cardiovascular Disease per 100,000 persons in 2015, Male
eFigure 9b. Age-Standardized All Cardiovascular Disease, Deaths per 100,000 persons in 2015, Male

eTable 1: 2015 Global and Regional YLLs for all CVD level 3 and 4 subcauses including 95% uncertainty, total number and age-standardized rate, by sex and total for 2015

	Years of Life Lost (YLLs)					
	All Ages			Age-standardized (per 100,000)		
	Total	Female	Male	Total	Female	Male
**Cardiovascular diseases**						
Global	321,908,774<br>(314,611,139 to 330,169,998)	129,774,606<br>(126,143,935 to 134,198,007)	192,134,169<br>(186,574,029 to 197,861,686)	4,799<br>(4,697 to 4,917)	3,670<br>(3,568 to 3,793)	6,022<br>(5,858 to 6,191)
Andean Latin America	1,098,336<br>(1,025,287 to 1,181,538)	491,812<br>(441,350 to 545,379)	606,524<br>(548,444 to 669,754)	2,433<br>(2,275 to 2,617)	2,078<br>(1,863 to 2,309)	2,815<br>(2,558 to 3,096)
Australasia	716,222<br>(699,515 to 733,145)	293,208<br>(283,498 to 303,378)	423,013<br>(410,813 to 437,411)	1,759<br>(1,719 to 1,802)	1,265<br>(1,225 to 1,306)	2,288<br>(2,223 to 2,365)
Caribbean	2,080,491<br>(1,947,121 to 2,212,360)	982,547<br>(882,084 to 1,094,590)	1,097,944<br>(1,019,913 to 1,179,207)	4,766<br>(4,464 to 5,064)	4,225<br>(3,788 to 4,713)	5,359<br>(4,990 to 5,736)
Central Asia	5,641,225<br>(5,469,201 to 5,812,266)	2,282,527<br>(2,181,564 to 2,375,248)	3,358,698<br>(3,232,703 to 3,491,623)	8,846<br>(8,601 to 9,100)	6,482<br>(6,209 to 6,740)	11,842<br>(11,448 to 12,256)
Central Europe	8,866,985<br>(8,703,497 to 9,010,743)	3,824,322<br>(3,744,969 to 3,903,144)	5,042,663<br>(4,935,331 to 5,147,908)	4,851<br>(4,755 to 4,932)	3,389<br>(3,317 to 3,461)	6,637<br>(6,494 to 6,775)
Central Latin America	5,565,138<br>(5,415,548 to 5,703,801)	2,376,855<br>(2,292,221 to 2,454,390)	3,188,282<br>(3,075,014 to 3,304,211)	2,934<br>(2,856 to 3,005)	2,368<br>(2,285 to 2,443)	3,567<br>(3,446 to 3,692)
Central Sub-Saharan Africa	3,585,086<br>(2,408,690 to 5,156,965)	1,921,944<br>(1,197,528 to 2,931,182)	1,663,141<br>(1,069,309 to 2,533,605)	7,479<br>(4,998 to 10,525)	7,822<br>(4,828 to 11,636)	6,985<br>(4,449 to 10,415)
East Asia	67,779,410<br>(64,663,651 to 71,288,604)	24,453,325<br>(22,974,498 to 26,051,655)	43,326,085<br>(40,763,048 to 46,205,250)	4,469<br>(4,276 to 4,687)	3,212<br>(3,029 to 3,413)	5,775<br>(5,458 to 6,126)
Eastern Europe	27,990,267<br>(27,373,275 to 28,673,806)	12,378,113<br>(12,039,991 to 12,765,749)	15,612,154<br>(15,146,776 to 16,089,527)	8,919<br>(8,708 to 9,145)	5,931<br>(5,762 to 6,129)	13,155<br>(12,774 to 13,541)
Eastern Sub-Saharan Africa	9,746,534<br>(8,042,783 to 11,771,553)	4,588,583<br>(3,483,341 to 5,924,471)	5,157,951<br>(3,996,260 to 6,604,100)	6,169<br>(5,103 to 7,435)	5,734<br>(4,375 to 7,356)	6,623<br>(5,209 to 8,486)
High-income Asia Pacific	5,300,784<br>(5,189,637 to 5,413,472)	2,205,736<br>(2,145,129 to 2,267,540)	3,095,048<br>(3,022,068 to 3,172,413)	1,490<br>(1,460 to 1,520)	995<br>(969 to 1,022)	2,043<br>(1,992 to 2,093)
High-income North America	12,862,962<br>(12,673,756 to 13,058,807)	5,229,519<br>(5,111,188 to 5,356,040)	7,633,442<br>(7,492,675 to 7,767,166)	2,577<br>(2,540 to 2,614)	1,875<br>(1,834 to 1,917)	3,359<br>(3,296 to 3,416)
North Africa and Middle East	23,806,196<br>(22,485,264 to 25,346,872)	10,308,648<br>(9,479,239 to 11,264,681)	13,497,548<br>(12,629,180 to 14,504,293)	6,401<br>(6,072 to 6,772)	5,516<br>(5,098 to 5,981)	7,314<br>(6,859 to 7,807)
Oceania	785,332<br>(575,442 to 1,096,141)	362,751<br>(259,582 to 508,644)	422,581<br>(311,116 to 595,000)	11,279<br>(8,480 to 15,249)	10,371<br>(7,651 to 14,160)	12,143<br>(9,238 to 16,350)
South Asia	80,871,510<br>(77,305,676 to 84,772,737)	30,214,506<br>(28,284,664 to 32,444,411)	50,657,004<br>(47,808,373 to 53,989,680)	6,797<br>(6,518 to 7,093)	5,258<br>(4,938 to 5,608)	8,320<br>(7,877 to 8,834)
Southeast Asia	28,413,937<br>(25,627,169 to 31,070,862)	11,535,433<br>(10,102,974 to 13,047,334)	16,878,504<br>(14,733,209 to 19,149,637)	5,593<br>(5,080 to 6,084)	4,368<br>(3,856 to 4,883)	6,972<br>(6,116 to 7,797)
Southern Latin America	2,296,134<br>(2,240,465 to 2,353,203)	968,043<br>(932,315 to 1,004,349)	1,328,091<br>(1,283,544 to 1,370,949)	3,226<br>(3,147 to 3,305)	2,285<br>(2,197 to 2,371)	4,374<br>(4,230 to 4,514)
Southern Sub-Saharan Africa	2,637,407<br>(2,366,098 to 2,987,332)	1,310,401<br>(1,132,658 to 1,538,681)	1,327,006<br>(1,167,803 to 1,523,246)	5,634<br>(5,081 to 6,337)	4,945<br>(4,291 to 5,799)	6,404<br>(5,720 to 7,259)
Tropical Latin America	8,017,664<br>(7,677,663 to 8,425,560)	3,345,056<br>(3,170,109 to 3,568,099)	4,672,608<br>(4,415,883 to 4,953,414)	4,213<br>(4,037 to 4,424)	3,230<br>(3,064 to 3,443)	5,414<br>(5,129 to 5,732)
Western Europe	15,658,936<br>(15,351,118 to 15,939,637)	6,709,801<br>(6,536,136 to 6,875,476)	8,949,135<br>(8,765,448 to 9,142,915)	1,983<br>(1,945 to 2,017)	1,373<br>(1,342 to 1,403)	2,666<br>(2,612 to 2,722)
Western Sub-Saharan Africa	8,188,218<br>(6,999,015 to 9,892,755)	3,991,473<br>(3,159,494 to 5,308,391)	4,196,745<br>(3,460,006 to 5,292,931)	4,895<br>(4,186 to 5,870)	4,880<br>(3,895 to 6,478)	4,869<br>(4,028 to 6,059)
**Aortic Aneurysm**						
Global	2,930,176<br>(2,846,030 to 3,007,029)	899,228<br>(871,473 to 932,501)	2,030,948<br>(1,964,364 to 2,090,492)	44<br>(43 to 45)	25<br>(25 to 26)	65<br>(63 to 67)
Andean Latin America	12,585<br>(11,138 to 14,097)	4,567<br>(3,987 to 5,224)	8,019<br>(6,758 to 9,246)	28<br>(25 to 32)	19<br>(17 to 22)	39<br>(33 to 45)
Australasia	24,211<br>(22,595 to 25,889)	8,606<br>(7,746 to 9,504)	15,605<br>(14,243 to 17,134)	61<br>(57 to 65)	40<br>(36 to 43)	84<br>(77 to 92)
Caribbean	25,781<br>(24,215 to 27,552)	7,959<br>(7,315 to 8,788)	17,822<br>(16,443 to 19,509)	60<br>(56 to 64)	34<br>(32 to 38)	89<br>(82 to 97)

Central Asia	27,560<br>(23,304 to 30,780)	7,298<br>(5,800 to 9,309)	20,262<br>(17,307 to 22,103)	42<br>(35 to 47)	20<br>(16 to 25)	71<br>(61 to 77)
Central Europe	101,420<br>(95,749 to 105,432)	30,241<br>(28,502 to 32,015)	71,179<br>(65,585 to 75,091)	59<br>(55 to 61)	31<br>(29 to 33)	93<br>(86 to 98)
Central Latin America	60,176<br>(56,890 to 63,077)	16,386<br>(15,337 to 17,464)	43,790<br>(40,970 to 46,351)	32<br>(30 to 33)	16<br>(15 to 17)	51<br>(47 to 54)
Central Sub-Saharan Africa	23,735<br>(15,005 to 37,729)	7,730<br>(4,356 to 12,639)	16,005<br>(9,326 to 26,370)	50<br>(33 to 77)	33<br>(19 to 52)	71<br>(44 to 111)
East Asia	302,404<br>(280,757 to 324,400)	78,292<br>(70,994 to 86,861)	224,112<br>(202,559 to 245,617)	19<br>(18 to 20)	9.8<br>(8.9 to 10.8)	28<br>(25 to 31)
Eastern Europe	203,891<br>(190,811 to 216,157)	61,110<br>(56,478 to 65,684)	142,780<br>(130,820 to 153,768)	67<br>(63 to 71)	32<br>(30 to 35)	119<br>(109 to 127)
Eastern Sub-Saharan Africa	78,000<br>(58,006 to 108,049)	19,954<br>(13,486 to 29,011)	58,046<br>(40,346 to 84,426)	47<br>(35 to 65)	24<br>(16 to 37)	73<br>(51 to 105)
High-income Asia Pacific	228,801<br>(221,791 to 236,082)	91,092<br>(87,233 to 94,993)	137,708<br>(131,515 to 144,342)	63<br>(61 to 65)	41<br>(39 to 43)	89<br>(85 to 93)
High-income North America	234,435<br>(225,221 to 244,889)	77,722<br>(73,380 to 82,343)	156,712<br>(148,839 to 166,186)	48<br>(46 to 50)	29<br>(27 to 31)	70<br>(66 to 74)
North Africa and Middle East	152,629<br>(143,208 to 162,809)	47,728<br>(43,607 to 52,284)	104,901<br>(96,892 to 113,880)	41<br>(38 to 43)	26<br>(24 to 29)	55<br>(52 to 60)
Oceania	5,830<br>(3,945 to 9,217)	1,995<br>(1,438 to 3,057)	3,835<br>(2,343 to 6,728)	90<br>(64 to 137)	64<br>(48 to 94)	119<br>(77 to 195)
South Asia	480,181<br>(448,513 to 519,337)	130,414<br>(119,937 to 143,263)	349,766<br>(321,139 to 387,382)	39<br>(36 to 42)	20<br>(19 to 22)	58<br>(53 to 64)
Southeast Asia	197,924<br>(164,414 to 232,693)	67,736<br>(54,227 to 82,227)	130,188<br>(106,201 to 155,334)	41<br>(34 to 47)	25<br>(21 to 30)	60<br>(49 to 71)
Southern Latin America	61,616<br>(58,094 to 65,134)	19,777<br>(18,197 to 21,499)	41,839<br>(38,534 to 44,961)	87<br>(82 to 92)	47<br>(44 to 51)	138<br>(127 to 148)
Southern Sub-Saharan Africa	31,069<br>(27,606 to 35,300)	8,326<br>(7,002 to 9,865)	22,743<br>(19,548 to 26,254)	66<br>(59 to 74)	30<br>(25 to 35)	117<br>(102 to 134)
Tropical Latin America	202,654<br>(187,210 to 219,084)	71,091<br>(65,279 to 77,825)	131,564<br>(118,115 to 145,091)	105<br>(97 to 113)	67<br>(62 to 74)	151<br>(136 to 166)
Western Europe	437,348<br>(420,085 to 456,680)	129,120<br>(123,072 to 135,654)	308,229<br>(292,626 to 325,772)	58<br>(56 to 61)	29<br>(28 to 31)	92<br>(87 to 97)
Western Sub-Saharan Africa	37,925<br>(30,751 to 49,272)	12,085<br>(8,305 to 17,995)	25,841<br>(20,253 to 35,181)	23<br>(19 to 29)	15<br>(11 to 21)	32<br>(26 to 43)
**Atrial fibrillation and flutter**						
Global	1,799,111<br>(1,518,144 to 2,126,610)	913,787<br>(769,001 to 1,078,533)	885,324<br>(745,353 to 1,050,012)	30<br>(25 to 35)	26<br>(22 to 31)	35<br>(29 to 41)
Andean Latin America	9,338<br>(7,169 to 11,948)	4,081<br>(3,147 to 5,250)	5,257<br>(3,968 to 6,816)	24<br>(18 to 31)	18<br>(14 to 24)	31<br>(23 to 40)
Australasia	13,483<br>(10,976 to 16,227)	6,362<br>(5,175 to 7,808)	7,121<br>(5,771 to 8,682)	31<br>(26 to 37)	26<br>(21 to 31)	37<br>(30 to 45)
Caribbean	10,997<br>(8,460 to 13,970)	4,886<br>(3,729 to 6,174)	6,111<br>(4,670 to 7,826)	26<br>(20 to 32)	20<br>(15 to 25)	33<br>(25 to 42)
Central Asia	20,418<br>(16,234 to 25,569)	11,442<br>(8,995 to 14,469)	8,976<br>(7,258 to 11,206)	38<br>(30 to 48)	36<br>(28 to 45)	42<br>(33 to 53)
Central Europe	85,592<br>(72,765 to 99,702)	49,270<br>(41,382 to 58,106)	36,323<br>(31,054 to 41,903)	44<br>(37 to 51)	40<br>(34 to 48)	49<br>(42 to 56)
Central Latin America	39,938<br>(30,985 to 50,146)	17,715<br>(13,876 to 22,240)	22,223<br>(17,260 to 27,911)	24<br>(19 to 31)	19<br>(15 to 24)	32<br>(24 to 41)
Central Sub-Saharan Africa	6,308<br>(3,980 to 9,111)	2,909<br>(1,609 to 4,626)	3,399<br>(2,067 to 5,221)	23<br>(14 to 33)	19<br>(11 to 31)	29<br>(18 to 44)
East Asia	316,838<br>(254,057 to 383,833)	148,661<br>(119,216 to 181,534)	168,177<br>(134,852 to 204,575)	25<br>(20 to 31)	22<br>(17 to 27)	29<br>(23 to 36)
Eastern Europe	118,820<br>(93,185 to 150,727)	77,975<br>(60,664 to 98,874)	40,845<br>(32,589 to 51,921)	35<br>(28 to 44)	33<br>(26 to 42)	38<br>(30 to 48)
Eastern Sub-Saharan Africa	17,168<br>(12,943 to 22,070)	6,658<br>(4,535 to 9,196)	10,510<br>(7,809 to 14,030)	18<br>(13 to 23)	12.6<br>(8.5 to 17.4)	24<br>(17 to 32)
High-income Asia Pacific	73,873<br>(56,652 to 93,235)	28,788<br>(22,066 to 37,117)	45,085<br>(34,840 to 56,712)	17<br>(13 to 20)	10.2<br>(7.9 to 12.9)	26<br>(20 to 32)

High-income North America	210,113<br>(183,645 to 237,601)	115,890<br>(100,469 to 133,281)	94,222<br>(83,192 to 106,477)	38<br>(34 to 43)	36<br>(31 to 40)	41<br>(36 to 47)
North Africa and Middle East	79,793<br>(63,912 to 98,670)	41,875<br>(32,905 to 52,259)	37,918<br>(30,381 to 47,280)	31<br>(24 to 39)	30<br>(23 to 37)	33<br>(26 to 43)
Oceania	1,127<br>(828 to 1,498)	574<br>(420 to 778)	553<br>(400 to 732)	32<br>(23 to 42)	28<br>(21 to 39)	37<br>(27 to 50)
South Asia	218,996<br>(174,949 to 268,245)	82,607<br>(65,554 to 102,418)	136,390<br>(107,802 to 167,033)	27<br>(21 to 34)	20<br>(15 to 25)	36<br>(28 to 45)
Southeast Asia	107,962<br>(84,033 to 134,451)	56,687<br>(44,069 to 71,820)	51,274<br>(39,430 to 64,782)	30<br>(23 to 38)	26<br>(20 to 34)	35<br>(26 to 45)
Southern Latin America	26,620<br>(20,778 to 33,386)	13,280<br>(10,143 to 17,042)	13,340<br>(10,491 to 16,784)	35<br>(28 to 44)	28<br>(21 to 35)	47<br>(37 to 59)
Southern Sub-Saharan Africa	5,254<br>(4,108 to 6,630)	2,654<br>(2,028 to 3,419)	2,600<br>(2,023 to 3,295)	14<br>(11 to 18)	11.1<br>(8.5 to 14.4)	20<br>(15 to 25)
Tropical Latin America	38,944<br>(30,713 to 48,220)	18,219<br>(14,431 to 22,811)	20,725<br>(16,477 to 26,003)	25<br>(19 to 31)	19<br>(15 to 24)	33<br>(26 to 42)
Western Europe	387,163<br>(324,622 to 461,813)	218,805<br>(182,237 to 262,646)	168,358<br>(141,089 to 200,261)	41<br>(35 to 49)	38<br>(32 to 45)	46<br>(38 to 54)
Western Sub-Saharan Africa	10,366<br>(7,903 to 13,689)	4,449<br>(3,261 to 6,216)	5,918<br>(4,362 to 7,971)	13<br>(10 to 18)	10.8<br>(7.8 to 15.0)	16<br>(12 to 22)
**Cardiomyopathy and myocarditis**						
Global	8,945,221<br>(8,513,808 to 9,431,718)	3,257,555<br>(3,128,826 to 3,424,308)	5,687,666<br>(5,196,369 to 6,090,516)	126<br>(120 to 133)	90<br>(86 to 95)	163<br>(149 to 174)
Andean Latin America	28,905<br>(25,139 to 35,424)	12,393<br>(10,622 to 14,563)	16,512<br>(13,762 to 22,138)	55<br>(48 to 68)	46<br>(40 to 54)	64<br>(54 to 86)
Australasia	27,013<br>(25,382 to 28,728)	7,505<br>(6,928 to 8,219)	19,507<br>(18,011 to 21,225)	76<br>(72 to 81)	40<br>(37 to 44)	114<br>(105 to 123)
Caribbean	68,656<br>(61,156 to 78,232)	30,684<br>(26,080 to 36,975)	37,972<br>(33,242 to 44,462)	155<br>(138 to 176)	135<br>(114 to 163)	175<br>(153 to 205)
Central Asia	113,722<br>(104,734 to 125,763)	32,243<br>(29,678 to 34,897)	81,479<br>(73,353 to 91,926)	141<br>(130 to 155)	78<br>(71 to 84)	211<br>(190 to 239)
Central Europe	502,878<br>(479,080 to 523,721)	196,440<br>(185,438 to 208,694)	306,438<br>(286,071 to 324,727)	292<br>(278 to 304)	185<br>(175 to 195)	415<br>(387 to 440)
Central Latin America	130,025<br>(122,942 to 140,554)	48,751<br>(46,205 to 51,535)	81,273<br>(75,312 to 90,191)	58<br>(55 to 62)	42<br>(40 to 45)	74<br>(68 to 81)
Central Sub-Saharan Africa	138,730<br>(97,245 to 192,462)	67,041<br>(40,671 to 98,823)	71,689<br>(46,680 to 105,953)	159<br>(108 to 233)	139<br>(87 to 212)	179<br>(110 to 282)
East Asia	833,920<br>(694,140 to 900,536)	310,369<br>(283,580 to 342,986)	523,551<br>(379,608 to 581,839)	60<br>(50 to 65)	48<br>(44 to 54)	72<br>(52 to 81)
Eastern Europe	2,244,117<br>(2,107,170 to 2,392,986)	619,352<br>(574,113 to 665,586)	1,624,766<br>(1,502,119 to 1,775,718)	833<br>(780 to 889)	403<br>(374 to 433)	1,336<br>(1,230 to 1,455)
Eastern Sub-Saharan Africa	227,532<br>(165,422 to 300,844)	85,437<br>(61,728 to 114,202)	142,095<br>(96,556 to 202,246)	99<br>(70 to 131)	71<br>(48 to 100)	129<br>(84 to 184)
High-income Asia Pacific	132,101<br>(126,822 to 137,518)	56,560<br>(53,859 to 59,499)	75,542<br>(71,619 to 79,766)	50<br>(48 to 52)	36<br>(34 to 38)	63<br>(60 to 67)
High-income North America	591,718<br>(568,474 to 617,154)	202,873<br>(192,314 to 213,801)	388,845<br>(368,036 to 412,690)	135<br>(130 to 141)	89<br>(84 to 93)	186<br>(176 to 197)
North Africa and Middle East	1,028,757<br>(922,601 to 1,125,627)	429,606<br>(383,298 to 477,938)	599,151<br>(488,814 to 670,406)	211<br>(191 to 230)	174<br>(158 to 192)	247<br>(209 to 281)
Oceania	29,229<br>(19,630 to 43,044)	15,335<br>(9,789 to 22,689)	13,894<br>(8,973 to 20,847)	333<br>(229 to 477)	338<br>(224 to 494)	331<br>(219 to 484)
South Asia	781,345<br>(663,199 to 854,374)	353,498<br>(310,503 to 401,368)	427,847<br>(292,553 to 492,764)	48<br>(42 to 52)	43<br>(38 to 49)	52<br>(39 to 60)
Southeast Asia	529,838<br>(456,906 to 635,823)	203,076<br>(171,541 to 241,610)	326,761<br>(266,653 to 421,538)	91<br>(79 to 108)	69<br>(58 to 81)	114<br>(94 to 148)
Southern Latin America	95,805<br>(90,526 to 101,345)	31,500<br>(29,173 to 34,180)	64,305<br>(59,500 to 69,482)	139<br>(132 to 147)	81<br>(75 to 87)	208<br>(193 to 225)
Southern Sub-Saharan Africa	198,514<br>(172,227 to 231,334)	88,156<br>(73,678 to 106,308)	110,358<br>(91,517 to 132,099)	344<br>(300 to 398)	279<br>(235 to 334)	424<br>(355 to 508)
Tropical Latin America	450,303<br>(396,102 to 494,200)	160,611<br>(148,364 to 175,409)	289,692<br>(231,173 to 325,195)	228<br>(201 to 251)	156<br>(144 to 170)	311<br>(250 to 350)

Western Europe	589,598<br>(564,719 to 615,490)	228,541<br>(215,054 to 243,072)	361,057<br>(341,636 to 382,881)	84<br>(80 to 87)	52<br>(49 to 54)	117<br>(111 to 124)
Western Sub-Saharan Africa	202,515<br>(164,610 to 255,554)	77,583<br>(56,273 to 105,659)	124,932<br>(97,179 to 167,947)	77<br>(62 to 99)	55<br>(41 to 77)	100<br>(77 to 140)
**Endocarditis**						
Global	2,197,934<br>(1,824,900 to 2,410,635)	1,055,438<br>(809,456 to 1,203,411)	1,142,496<br>(903,571 to 1,337,854)	31<br>(26 to 34)	29<br>(22 to 33)	33<br>(26 to 38)
Andean Latin America	21,466<br>(17,390 to 26,563)	9,746<br>(7,468 to 13,501)	11,720<br>(8,319 to 15,832)	40<br>(32 to 50)	36<br>(27 to 49)	45<br>(31 to 59)
Australasia	5,905<br>(5,421 to 6,382)	2,602<br>(2,320 to 2,931)	3,303<br>(2,958 to 3,665)	16<br>(15 to 17)	13<br>(12 to 15)	19<br>(17 to 21)
Caribbean	18,046<br>(15,544 to 21,224)	9,628<br>(7,580 to 12,672)	8,418<br>(6,782 to 10,229)	41<br>(35 to 48)	42<br>(33 to 56)	39<br>(31 to 47)
Central Asia	12,617<br>(10,675 to 16,333)	4,668<br>(3,867 to 6,349)	7,949<br>(6,514 to 10,765)	16<br>(13 to 20)	11.2<br>(9.4 to 15.1)	21<br>(17 to 28)
Central Europe	25,130<br>(23,350 to 26,812)	9,085<br>(8,488 to 9,848)	16,045<br>(14,566 to 17,404)	16<br>(15 to 17)	10.8<br>(10.0 to 11.7)	23<br>(20 to 24)
Central Latin America	55,150<br>(52,090 to 58,173)	23,379<br>(21,866 to 25,066)	31,771<br>(29,485 to 34,257)	24<br>(23 to 25)	20<br>(19 to 21)	28<br>(26 to 30)
Central Sub-Saharan Africa	57,028<br>(32,829 to 85,084)	31,081<br>(12,671 to 50,869)	25,947<br>(13,827 to 43,391)	72<br>(40 to 113)	78<br>(34 to 131)	65<br>(34 to 106)
East Asia	151,602<br>(123,199 to 191,764)	56,337<br>(40,947 to 76,126)	95,265<br>(70,957 to 129,472)	10.8<br>(8.8 to 13.5)	8.3<br>(6.0 to 11.1)	13.4<br>(9.9 to 18.2)
Eastern Europe	79,745<br>(72,607 to 88,459)	25,169<br>(22,987 to 28,700)	54,576<br>(48,178 to 61,888)	31<br>(28 to 34)	17<br>(16 to 20)	46<br>(41 to 53)
Eastern Sub-Saharan Africa	179,407<br>(111,753 to 236,339)	86,146<br>(43,294 to 118,584)	93,262<br>(55,045 to 134,080)	68<br>(38 to 98)	64<br>(32 to 96)	71<br>(39 to 114)
High-income Asia Pacific	44,572<br>(42,462 to 46,916)	22,660<br>(21,363 to 24,106)	21,912<br>(20,485 to 23,366)	14<br>(14 to 15)	12<br>(12 to 13)	16<br>(15 to 17)
High-income North America	145,474<br>(139,378 to 151,601)	66,944<br>(63,280 to 70,578)	78,530<br>(74,147 to 82,779)	31<br>(30 to 33)	27<br>(25 to 28)	36<br>(34 to 38)
North Africa and Middle East	309,311<br>(267,590 to 412,108)	189,936<br>(153,274 to 267,814)	119,374<br>(93,988 to 161,678)	67<br>(59 to 93)	85<br>(70 to 125)	49<br>(40 to 70)
Oceania	11,814<br>(8,226 to 16,360)	7,371<br>(4,582 to 11,490)	4,443<br>(2,875 to 6,579)	146<br>(103 to 199)	181<br>(116 to 271)	107<br>(72 to 156)
South Asia	244,962<br>(126,882 to 304,495)	116,656<br>(48,199 to 145,880)	128,306<br>(65,594 to 162,941)	15.5<br>(8.8 to 18.9)	14.7<br>(6.7 to 18.0)	16.4<br>(9.1 to 20.4)
Southeast Asia	354,598<br>(297,066 to 430,244)	157,459<br>(118,576 to 204,623)	197,139<br>(153,715 to 249,816)	59<br>(49 to 70)	51<br>(38 to 66)	67<br>(51 to 84)
Southern Latin America	31,751<br>(29,525 to 33,893)	18,375<br>(16,661 to 20,228)	13,376<br>(12,224 to 14,530)	46<br>(43 to 49)	45<br>(42 to 49)	43<br>(39 to 47)
Southern Sub-Saharan Africa	25,945<br>(21,819 to 35,104)	11,940<br>(9,358 to 18,031)	14,005<br>(10,732 to 20,600)	39<br>(33 to 52)	34<br>(27 to 52)	43<br>(33 to 64)
Tropical Latin America	76,188<br>(54,869 to 89,728)	32,834<br>(21,203 to 40,896)	43,353<br>(28,793 to 54,434)	37<br>(26 to 44)	31<br>(20 to 38)	45<br>(29 to 55)
Western Europe	169,852<br>(161,333 to 178,512)	86,759<br>(81,148 to 92,943)	83,093<br>(76,371 to 89,352)	24<br>(23 to 25)	20<br>(19 to 22)	27<br>(25 to 29)
Western Sub-Saharan Africa	177,372<br>(135,982 to 229,570)	86,664<br>(56,600 to 129,984)	90,708<br>(65,051 to 124,011)	56<br>(44 to 74)	54<br>(38 to 81)	57<br>(41 to 81)
**Hemorrhagic and other stroke**						
Global	70,623,033<br>(68,220,445 to 73,564,496)	29,333,303<br>(27,872,920 to 31,381,178)	41,289,730<br>(39,438,614 to 43,279,057)	1,030<br>(995 to 1,073)	822<br>(781 to 880)	1,256<br>(1,201 to 1,316)
Andean Latin America	214,782<br>(197,475 to 238,715)	103,252<br>(91,125 to 117,780)	111,529<br>(99,125 to 126,400)	445<br>(408 to 495)	417<br>(368 to 475)	473<br>(418 to 536)
Australasia	95,308<br>(90,279 to 101,208)	49,889<br>(46,038 to 53,951)	45,419<br>(42,117 to 49,348)	236<br>(224 to 249)	224<br>(209 to 241)	247<br>(230 to 268)
Caribbean	381,939<br>(347,208 to 418,563)	197,711<br>(168,620 to 231,752)	184,228<br>(166,386 to 204,621)	868<br>(790 to 951)	856<br>(729 to 1,005)	882<br>(800 to 977)
Central Asia	959,392<br>(918,469 to 1,029,525)	438,219<br>(403,243 to 495,596)	521,172<br>(491,394 to 553,774)	1,453<br>(1,389 to 1,557)	1,201<br>(1,107 to 1,353)	1,766<br>(1,666 to 1,875)

Central Europe	1,189,331<br>(1,149,019 to 1,240,077)	549,281<br>(524,745 to 572,179)	640,050<br>(616,256 to 681,064)	678<br>(655 to 706)	536<br>(513 to 559)	847<br>(816 to 900)
Central Latin America	986,403<br>(951,738 to 1,019,951)	471,384<br>(451,161 to 492,019)	515,019<br>(490,422 to 540,519)	490<br>(474 to 507)	447<br>(428 to 466)	538<br>(513 to 564)
Central Sub-Saharan Africa	1,052,294<br>(692,972 to 1,562,499)	575,238<br>(337,458 to 906,792)	477,056<br>(300,991 to 737,478)	2,036<br>(1,303 to 2,939)	2,167<br>(1,231 to 3,381)	1,878<br>(1,165 to 2,928)
East Asia	22,492,144<br>(21,251,873 to 24,214,858)	7,806,002<br>(7,210,706 to 8,792,281)	14,686,142<br>(13,609,724 to 15,875,148)	1,426<br>(1,348 to 1,540)	988<br>(915 to 1,123)	1,877<br>(1,742 to 2,023)
Eastern Europe	2,586,833<br>(2,451,007 to 2,734,949)	1,185,669<br>(1,096,487 to 1,281,855)	1,401,164<br>(1,302,843 to 1,499,165)	844<br>(801 to 888)	618<br>(574 to 666)	1,151<br>(1,073 to 1,226)
Eastern Sub-Saharan Africa	2,424,699<br>(1,911,450 to 2,981,447)	1,197,385<br>(854,693 to 1,609,404)	1,227,314<br>(931,460 to 1,609,105)	1,443<br>(1,136 to 1,776)	1,396<br>(969 to 1,881)	1,488<br>(1,122 to 1,946)
High-income Asia Pacific	1,164,266<br>(1,125,832 to 1,207,312)	470,383<br>(449,837 to 494,538)	693,883<br>(666,432 to 722,292)	366<br>(355 to 379)	257<br>(247 to 269)	485<br>(466 to 505)
High-income North America	1,028,038<br>(991,249 to 1,070,851)	503,752<br>(478,738 to 530,568)	524,285<br>(498,217 to 555,044)	217<br>(210 to 226)	200<br>(190 to 210)	236<br>(225 to 250)
North Africa and Middle East	4,035,257<br>(3,710,082 to 4,492,444)	1,929,068<br>(1,685,675 to 2,263,172)	2,106,189<br>(1,944,553 to 2,341,315)	987<br>(909 to 1,099)	946<br>(829 to 1,102)	1,033<br>(955 to 1,168)
Oceania	192,578<br>(137,190 to 279,717)	106,304<br>(71,616 to 156,302)	86,274<br>(60,406 to 123,402)	2,615<br>(1,891 to 3,705)	2,877<br>(1,970 to 4,220)	2,310<br>(1,615 to 3,243)
South Asia	15,173,456<br>(13,987,019 to 16,304,292)	6,373,524<br>(5,478,720 to 7,146,032)	8,799,931<br>(7,924,700 to 9,701,190)	1,199<br>(1,101 to 1,293)	1,038<br>(881 to 1,172)	1,355<br>(1,217 to 1,499)
Southeast Asia	9,352,614<br>(8,283,051 to 10,389,562)	3,900,517<br>(3,294,651 to 4,504,850)	5,452,097<br>(4,690,434 to 6,317,434)	1,778<br>(1,585 to 1,961)	1,431<br>(1,217 to 1,644)	2,162<br>(1,864 to 2,472)
Southern Latin America	346,556<br>(331,744 to 359,967)	153,474<br>(144,731 to 162,465)	193,083<br>(182,455 to 204,214)	500<br>(479 to 519)	400<br>(377 to 423)	621<br>(587 to 657)
Southern Sub-Saharan Africa	564,469<br>(497,815 to 647,673)	281,156<br>(238,450 to 341,019)	283,313<br>(241,605 to 333,248)	1,133<br>(1,006 to 1,290)	1,020<br>(871 to 1,229)	1,250<br>(1,085 to 1,471)
Tropical Latin America	2,007,630<br>(1,913,492 to 2,162,505)	924,520<br>(870,402 to 991,401)	1,083,109<br>(1,017,696 to 1,208,866)	1,033<br>(985 to 1,111)	873<br>(822 to 935)	1,231<br>(1,157 to 1,371)
Western Europe	1,967,477<br>(1,899,948 to 2,045,706)	957,561<br>(914,165 to 1,002,173)	1,009,916<br>(967,534 to 1,059,399)	260<br>(252 to 269)	219<br>(210 to 229)	306<br>(293 to 320)
Western Sub-Saharan Africa	2,407,569<br>(2,045,106 to 2,912,238)	1,159,014<br>(910,331 to 1,559,330)	1,248,556<br>(1,012,004 to 1,570,157)	1,223<br>(1,032 to 1,497)	1,210<br>(942 to 1,631)	1,231<br>(1,000 to 1,572)
**Hypertensive heart disease**						
Global	16,814,565<br>(15,609,949 to 17,888,838)	8,063,747<br>(6,798,970 to 8,865,627)	8,750,818<br>(8,234,861 to 9,288,021)	252<br>(233 to 268)	228<br>(192 to 250)	277<br>(261 to 294)
Andean Latin America	52,361<br>(45,814 to 57,702)	27,080<br>(20,462 to 31,437)	25,281<br>(21,946 to 28,978)	120<br>(104 to 132)	116<br>(89 to 135)	122<br>(106 to 141)
Australasia	10,918<br>(9,921 to 12,058)	6,115<br>(5,334 to 7,048)	4,803<br>(4,265 to 5,523)	26<br>(24 to 29)	25<br>(22 to 28)	26<br>(23 to 30)
Caribbean	136,877<br>(120,545 to 167,282)	72,415<br>(58,549 to 99,191)	64,462<br>(55,724 to 78,587)	314<br>(276 to 384)	311<br>(250 to 425)	316<br>(274 to 383)
Central Asia	358,990<br>(289,688 to 399,475)	165,871<br>(94,945 to 195,839)	193,119<br>(167,365 to 219,523)	564<br>(448 to 628)	463<br>(261 to 547)	696<br>(601 to 792)
Central Europe	395,808<br>(363,338 to 416,565)	202,033<br>(190,096 to 214,129)	193,775<br>(157,544 to 209,638)	212<br>(194 to 223)	177<br>(167 to 187)	254<br>(206 to 273)
Central Latin America	299,235<br>(284,985 to 314,748)	162,055<br>(150,932 to 172,682)	137,180<br>(128,123 to 147,100)	167<br>(159 to 176)	166<br>(155 to 177)	166<br>(156 to 178)
Central Sub-Saharan Africa	357,525<br>(221,756 to 553,121)	232,478<br>(120,470 to 395,293)	125,047<br>(71,022 to 216,870)	830<br>(513 to 1,271)	1,039<br>(554 to 1,766)	573<br>(334 to 983)
East Asia	3,882,813<br>(3,322,545 to 4,201,381)	1,555,035<br>(1,011,618 to 1,747,733)	2,327,778<br>(2,106,805 to 2,552,881)	266<br>(224 to 287)	208<br>(135 to 234)	328<br>(297 to 359)
Eastern Europe	524,454<br>(492,696 to 564,914)	267,828<br>(246,438 to 292,303)	256,626<br>(231,922 to 286,362)	166<br>(156 to 179)	130<br>(120 to 141)	214<br>(194 to 238)
Eastern Sub-Saharan Africa	997,925<br>(694,186 to 1,582,141)	590,341<br>(341,986 to 1,040,848)	407,584<br>(290,895 to 706,075)	673<br>(457 to 1,117)	779<br>(444 to 1,415)	543<br>(390 to 998)
High-income Asia Pacific	132,924<br>(125,168 to 142,713)	77,849<br>(72,649 to 83,979)	55,076<br>(51,077 to 61,146)	34<br>(32 to 36)	31<br>(29 to 34)	36<br>(33 to 40)
High-income North America	660,907<br>(581,842 to 690,950)	272,863<br>(254,982 to 289,316)	388,044<br>(318,548 to 413,817)	136<br>(119 to 143)	102<br>(95 to 108)	172<br>(141 to 183)

North Africa and Middle East	1,024,654 (922,916 to 1,160,280)	588,228 (503,271 to 734,622)	436,426 (387,434 to 489,517)	300 (270 to 336)	332 (285 to 412)	266 (236 to 296)
Oceania	38,212 (26,210 to 57,435)	19,471 (11,923 to 31,315)	18,741 (12,834 to 28,270)	615 (434 to 926)	606 (385 to 978)	623 (440 to 966)
South Asia	4,787,266 (4,295,242 to 5,415,984)	2,061,601 (1,765,209 to 2,539,856)	2,725,665 (2,322,364 to 3,106,398)	398 (356 to 448)	351 (299 to 433)	443 (376 to 507)
Southeast Asia	1,356,173 (1,197,263 to 1,540,060)	693,301 (573,973 to 842,199)	662,872 (567,775 to 772,172)	269 (238 to 304)	261 (217 to 315)	274 (237 to 317)
Southern Latin America	112,978 (106,006 to 122,155)	54,619 (49,559 to 59,949)	58,359 (53,666 to 64,010)	155 (146 to 167)	122 (112 to 134)	196 (180 to 216)
Southern Sub-Saharan Africa	266,048 (233,140 to 308,018)	161,944 (134,859 to 195,055)	104,104 (90,667 to 119,721)	591 (517 to 678)	623 (520 to 747)	531 (469 to 603)
Tropical Latin America	406,673 (366,598 to 431,793)	205,353 (171,508 to 226,692)	201,320 (185,696 to 215,729)	220 (196 to 233)	200 (165 to 221)	245 (226 to 263)
Western Europe	605,258 (571,025 to 641,977)	359,930 (331,887 to 389,894)	245,329 (228,280 to 264,742)	69 (65 to 73)	66 (61 to 70)	70 (65 to 75)
Western Sub-Saharan Africa	406,566 (321,147 to 552,740)	287,337 (197,966 to 420,884)	119,230 (90,767 to 161,623)	250 (196 to 336)	352 (250 to 503)	137 (105 to 186)
**Ischemic heart disease**						
Global	156,745,658 (152,797,770 to 160,957,100)	57,580,629 (55,973,775 to 59,632,960)	99,165,029 (95,982,020 to 102,734,676)	2,343 (2,285 to 2,403)	1,634 (1,589 to 1,692)	3,106 (3,011 to 3,209)
Andean Latin America	534,930 (495,318 to 576,593)	222,256 (198,037 to 250,438)	312,674 (279,934 to 347,926)	1,222 (1,132 to 1,319)	966 (859 to 1,090)	1,498 (1,345 to 1,653)
Australasia	420,533 (406,499 to 433,350)	151,984 (144,416 to 159,491)	268,549 (258,273 to 278,728)	1,033 (999 to 1,063)	645 (613 to 676)	1,450 (1,395 to 1,504)
Caribbean	1,026,879 (969,210 to 1,085,389)	437,889 (398,421 to 482,253)	588,990 (550,368 to 631,009)	2,355 (2,223 to 2,490)	1,876 (1,705 to 2,068)	2,882 (2,696 to 3,077)
Central Asia	3,296,465 (3,172,848 to 3,424,808)	1,250,126 (1,185,851 to 1,310,872)	2,046,339 (1,947,373 to 2,147,913)	5,251 (5,071 to 5,443)	3,631 (3,449 to 3,800)	7,304 (6,974 to 7,624)
Central Europe	4,704,390 (4,605,636 to 4,795,481)	1,881,457 (1,836,768 to 1,924,439)	2,822,934 (2,744,936 to 2,894,107)	2,556 (2,498 to 2,607)	1,626 (1,585 to 1,664)	3,695 (3,595 to 3,789)
Central Latin America	3,245,807 (3,149,886 to 3,330,968)	1,267,416 (1,218,980 to 1,311,814)	1,978,391 (1,904,045 to 2,055,287)	1,733 (1,683 to 1,777)	1,286 (1,237 to 1,330)	2,234 (2,155 to 2,317)
Central Sub-Saharan Africa	1,026,771 (655,805 to 1,542,636)	462,960 (271,126 to 716,457)	563,811 (342,986 to 897,808)	2,403 (1,563 to 3,431)	2,193 (1,323 to 3,282)	2,595 (1,617 to 3,993)
East Asia	25,207,835 (23,858,923 to 26,611,608)	8,828,763 (8,244,901 to 9,441,690)	16,379,073 (15,219,087 to 17,665,323)	1,662 (1,580 to 1,750)	1,167 (1,093 to 1,246)	2,172 (2,028 to 2,331)
Eastern Europe	16,663,181 (16,261,727 to 17,054,359)	7,158,632 (6,933,221 to 7,384,622)	9,504,549 (9,203,439 to 9,813,611)	5,223 (5,096 to 5,348)	3,325 (3,221 to 3,436)	8,002 (7,762 to 8,241)
Eastern Sub-Saharan Africa	3,084,083 (2,431,134 to 3,837,577)	1,227,141 (870,859 to 1,693,777)	1,856,942 (1,391,307 to 2,474,449)	2,070 (1,643 to 2,548)	1,660 (1,191 to 2,247)	2,525 (1,931 to 3,315)
High-income Asia Pacific	2,166,000 (2,112,127 to 2,220,663)	826,918 (792,151 to 859,266)	1,339,082 (1,294,239 to 1,383,200)	612 (597 to 628)	358 (345 to 372)	890 (859 to 919)
High-income North America	7,918,311 (7,718,471 to 8,076,801)	2,953,356 (2,845,620 to 3,050,230)	4,964,955 (4,831,780 to 5,076,783)	1,562 (1,524 to 1,593)	1,032 (996 to 1,063)	2,156 (2,099 to 2,204)
North Africa and Middle East	12,698,714 (11,872,505 to 13,616,871)	4,823,324 (4,420,181 to 5,316,216)	7,875,390 (7,289,692 to 8,561,362)	3,533 (3,323 to 3,760)	2,719 (2,508 to 2,964)	4,367 (4,070 to 4,700)
Oceania	350,880 (256,876 to 487,590)	127,099 (90,486 to 181,046)	223,782 (163,725 to 315,236)	5,183 (3,928 to 6,966)	3,922 (2,882 to 5,369)	6,446 (4,906 to 8,688)
South Asia	45,499,549 (43,342,793 to 48,060,954)	15,084,312 (14,049,374 to 16,366,510)	30,415,237 (28,610,810 to 32,603,576)	3,873 (3,698 to 4,071)	2,713 (2,532 to 2,937)	5,020 (4,734 to 5,345)
Southeast Asia	11,854,851 (10,632,446 to 13,036,603)	4,217,467 (3,707,708 to 4,751,282)	7,637,384 (6,666,846 to 8,655,077)	2,317 (2,093 to 2,520)	1,605 (1,425 to 1,792)	3,123 (2,743 to 3,473)
Southern Latin America	1,152,236 (1,115,850 to 1,186,836)	440,901 (421,076 to 461,994)	711,335 (682,644 to 736,991)	1,614 (1,563 to 1,663)	1,018 (974 to 1,067)	2,337 (2,243 to 2,421)
Southern Sub-Saharan Africa	1,024,791 (916,632 to 1,156,002)	474,488 (406,290 to 557,223)	550,304 (482,639 to 630,405)	2,302 (2,074 to 2,583)	1,876 (1,613 to 2,195)	2,799 (2,483 to 3,168)
Tropical Latin America	3,718,598 (3,545,957 to 3,904,185)	1,422,468 (1,341,224 to 1,530,940)	2,296,129 (2,142,073 to 2,445,214)	1,946 (1,857 to 2,043)	1,378 (1,299 to 1,480)	2,625 (2,458 to 2,789)
Western Europe	8,263,885 (8,068,828 to 8,435,307)	3,067,414 (2,966,414 to 3,169,367)	5,196,472 (5,072,530 to 5,324,522)	1,058 (1,036 to 1,079)	619 (600 to 638)	1,553 (1,517 to 1,590)

Western Sub-Saharan Africa	2,886,968 (2,439,007 to 3,475,099)	1,254,257 (981,436 to 1,698,642)	1,632,710 (1,309,843 to 2,093,666)	1,987 (1,706 to 2,356)	1,783 (1,426 to 2,358)	2,193 (1,794 to 2,745)
**Ischemic stroke**						
Global	41,548,546 (39,843,949 to 43,182,109)	18,890,267 (17,796,906 to 20,065,480)	22,658,279 (21,549,208 to 23,734,429)	651 (625 to 676)	543 (512 to 577)	773 (738 to 807)
Andean Latin America	534,930 (495,318 to 576,593)	222,256 (198,037 to 250,438)	312,674 (279,934 to 347,926)	1,222 (1,132 to 1,319)	966 (859 to 1,090)	1,498 (1,345 to 1,653)
Australasia	420,533 (406,499 to 433,350)	151,984 (144,416 to 159,491)	268,549 (258,273 to 278,728)	1,033 (999 to 1,063)	645 (613 to 676)	1,450 (1,395 to 1,504)
Caribbean	1,026,879 (969,210 to 1,085,389)	437,889 (398,421 to 482,253)	588,990 (550,368 to 631,009)	2,355 (2,223 to 2,490)	1,876 (1,705 to 2,068)	2,882 (2,696 to 3,077)
Central Asia	3,296,465 (3,172,848 to 3,424,808)	1,250,126 (1,185,851 to 1,310,872)	2,046,339 (1,947,373 to 2,147,913)	5,251 (5,071 to 5,443)	3,631 (3,449 to 3,800)	7,304 (6,974 to 7,624)
Central Europe	4,704,390 (4,605,636 to 4,795,481)	1,881,457 (1,836,768 to 1,924,439)	2,822,934 (2,744,936 to 2,894,107)	2,556 (2,498 to 2,607)	1,626 (1,585 to 1,664)	3,695 (3,595 to 3,789)
Central Latin America	3,245,807 (3,149,886 to 3,330,968)	1,267,416 (1,218,980 to 1,311,814)	1,978,391 (1,904,045 to 2,055,287)	1,733 (1,683 to 1,777)	1,286 (1,237 to 1,330)	2,234 (2,155 to 2,317)
Central Sub-Saharan Africa	1,026,771 (655,805 to 1,542,636)	462,960 (271,126 to 716,457)	563,811 (342,986 to 897,808)	2,403 (1,563 to 3,431)	2,193 (1,323 to 3,282)	2,595 (1,617 to 3,993)
East Asia	25,207,835 (23,858,923 to 26,611,608)	8,828,763 (8,244,901 to 9,441,690)	16,379,073 (15,219,087 to 17,665,323)	1,662 (1,580 to 1,750)	1,167 (1,093 to 1,246)	2,172 (2,028 to 2,331)
Eastern Europe	16,663,181 (16,261,727 to 17,054,359)	7,158,632 (6,933,221 to 7,384,622)	9,504,549 (9,203,439 to 9,813,611)	5,223 (5,096 to 5,348)	3,325 (3,221 to 3,436)	8,002 (7,762 to 8,241)
Eastern Sub-Saharan Africa	3,084,083 (2,431,134 to 3,837,577)	1,227,141 (870,859 to 1,693,777)	1,856,942 (1,391,307 to 2,474,449)	2,070 (1,643 to 2,548)	1,660 (1,191 to 2,247)	2,525 (1,931 to 3,315)
High-income Asia Pacific	2,166,000 (2,112,127 to 2,220,663)	826,918 (792,151 to 859,266)	1,339,082 (1,294,239 to 1,383,200)	612 (597 to 628)	358 (345 to 372)	890 (859 to 919)
High-income North America	7,918,311 (7,718,471 to 8,076,801)	2,953,356 (2,845,620 to 3,050,230)	4,964,955 (4,831,780 to 5,076,783)	1,562 (1,524 to 1,593)	1,032 (996 to 1,063)	2,156 (2,099 to 2,204)
North Africa and Middle East	12,698,714 (11,872,505 to 13,616,871)	4,823,324 (4,420,181 to 5,316,216)	7,875,390 (7,289,692 to 8,561,362)	3,533 (3,323 to 3,760)	2,719 (2,508 to 2,964)	4,367 (4,070 to 4,700)
Oceania	350,880 (256,876 to 487,590)	127,099 (90,486 to 181,046)	223,782 (163,725 to 315,236)	5,183 (3,928 to 6,966)	3,922 (2,882 to 5,369)	6,446 (4,906 to 8,688)
South Asia	45,499,549 (43,342,793 to 48,060,954)	15,084,312 (14,049,374 to 16,366,510)	30,415,237 (28,610,810 to 32,603,576)	3,873 (3,698 to 4,071)	2,713 (2,532 to 2,937)	5,020 (4,734 to 5,345)
Southeast Asia	11,854,851 (10,632,446 to 13,036,603)	4,217,467 (3,707,708 to 4,751,282)	7,637,384 (6,666,846 to 8,655,077)	2,317 (2,093 to 2,520)	1,605 (1,425 to 1,792)	3,123 (2,743 to 3,473)
Southern Latin America	1,152,236 (1,115,850 to 1,186,836)	440,901 (421,076 to 461,994)	711,335 (682,644 to 736,991)	1,614 (1,563 to 1,663)	1,018 (974 to 1,067)	2,337 (2,243 to 2,421)
Southern Sub-Saharan Africa	1,024,791 (916,632 to 1,156,002)	474,488 (406,290 to 557,223)	550,304 (482,639 to 630,405)	2,302 (2,074 to 2,583)	1,876 (1,613 to 2,195)	2,799 (2,483 to 3,168)
Tropical Latin America	3,718,598 (3,545,957 to 3,904,185)	1,422,468 (1,341,224 to 1,530,940)	2,296,129 (2,142,073 to 2,445,214)	1,946 (1,857 to 2,043)	1,378 (1,299 to 1,480)	2,625 (2,458 to 2,789)
Western Europe	8,263,885 (8,068,828 to 8,435,307)	3,067,414 (2,966,414 to 3,169,367)	5,196,472 (5,072,530 to 5,324,522)	1,058 (1,036 to 1,079)	619 (600 to 638)	1,553 (1,517 to 1,590)
Western Sub-Saharan Africa	2,886,968 (2,439,007 to 3,475,099)	1,254,257 (981,436 to 1,698,642)	1,632,710 (1,309,843 to 2,093,666)	1,987 (1,706 to 2,356)	1,783 (1,426 to 2,358)	2,193 (1,794 to 2,745)
**Other cardiovascular and circulatory diseases**						
Global	10,820,011 (10,339,766 to 11,347,688)	5,025,055 (4,743,043 to 5,345,762)	5,794,956 (5,478,813 to 6,142,325)	158 (152 to 166)	141 (133 to 150)	177 (168 to 188)
Andean Latin America	70,695 (63,472 to 77,530)	33,083 (28,656 to 37,641)	37,612 (32,367 to 42,547)	144 (131 to 158)	131 (113 to 150)	157 (137 to 178)
Australasia	32,189 (30,057 to 34,409)	15,384 (13,879 to 16,964)	16,805 (15,353 to 18,389)	81 (76 to 86)	71 (65 to 78)	92 (84 to 100)
Caribbean	93,038 (84,676 to 103,541)	47,888 (41,333 to 56,080)	45,150 (40,601 to 50,558)	211 (192 to 235)	208 (179 to 244)	215 (195 to 241)
Central Asia	90,875 (83,169 to 99,891)	30,973 (27,225 to 34,593)	59,903 (52,863 to 67,580)	122 (112 to 134)	78 (69 to 87)	176 (156 to 197)
Central Europe	329,409 (318,480 to 340,682)	133,277 (127,613 to 138,841)	196,133 (187,735 to 204,836)	189 (182 to 195)	131 (126 to 136)	259 (248 to 270)

Central Latin America	236,472 (228,807 to 244,357)	114,066 (109,031 to 119,662)	122,406 (117,299 to 128,084)	115 (111 to 119)	106 (101 to 111)	124 (119 to 130)
Central Sub-Saharan Africa	381,688 (272,739 to 541,473)	211,509 (134,830 to 320,916)	170,180 (114,381 to 252,921)	606 (403 to 884)	654 (392 to 1,037)	549 (347 to 853)
East Asia	1,371,477 (1,280,115 to 1,473,235)	530,634 (479,950 to 582,205)	840,843 (770,600 to 924,024)	99 (92 to 106)	76 (69 to 84)	122 (112 to 133)
Eastern Europe	357,549 (336,157 to 381,511)	157,233 (145,303 to 171,238)	200,316 (184,535 to 218,457)	123 (116 to 131)	90 (84 to 98)	166 (153 to 181)
Eastern Sub-Saharan Africa	1,436,902 (1,182,575 to 1,735,426)	660,711 (496,403 to 861,319)	776,191 (614,449 to 966,437)	809 (656 to 978)	738 (544 to 979)	885 (695 to 1,122)
High-income Asia Pacific	183,553 (176,817 to 191,142)	94,868 (90,316 to 100,289)	88,684 (84,590 to 93,087)	53 (51 to 55)	47 (45 to 49)	60 (57 to 63)
High-income North America	615,692 (592,239 to 643,959)	304,221 (288,736 to 321,339)	311,471 (295,626 to 330,497)	130 (125 to 136)	119 (113 to 126)	142 (135 to 151)
North Africa and Middle East	1,642,969 (1,536,648 to 1,773,343)	792,241 (720,176 to 877,772)	850,728 (781,589 to 932,621)	384 (360 to 409)	366 (333 to 404)	402 (370 to 439)
Oceania	39,142 (29,530 to 53,671)	21,080 (14,807 to 29,742)	18,061 (13,700 to 24,242)	493 (377 to 657)	519 (371 to 719)	468 (366 to 612)
South Asia	434,826 (400,352 to 476,093)	186,423 (167,051 to 212,212)	248,403 (223,661 to 280,457)	39 (36 to 42)	33 (30 to 38)	44 (40 to 50)
Southeast Asia	889,987 (794,766 to 991,111)	395,211 (342,020 to 457,020)	494,776 (428,900 to 567,592)	166 (149 to 185)	142 (123 to 164)	194 (169 to 222)
Southern Latin America	150,330 (142,329 to 158,393)	76,745 (71,273 to 82,854)	73,584 (68,486 to 78,997)	214 (203 to 225)	189 (177 to 203)	242 (225 to 260)
Southern Sub-Saharan Africa	126,405 (110,332 to 145,063)	68,476 (57,546 to 81,874)	57,928 (48,593 to 69,511)	222 (196 to 252)	219 (186 to 257)	226 (194 to 264)
Tropical Latin America	397,618 (376,008 to 421,535)	193,508 (180,120 to 209,694)	204,110 (188,762 to 220,645)	206 (195 to 218)	186 (173 to 201)	229 (213 to 247)
Western Europe	1,104,516 (1,062,413 to 1,147,835)	554,600 (524,900 to 584,722)	549,916 (523,973 to 580,438)	145 (140 to 151)	125 (119 to 131)	167 (159 to 176)
Western Sub-Saharan Africa	834,680 (712,722 to 1,008,888)	402,925 (328,287 to 525,558)	431,755 (350,225 to 556,109)	353 (297 to 442)	355 (276 to 481)	347 (275 to 459)
**Peripheral artery disease**						
Global	626,004 (597,995 to 658,714)	261,357 (242,226 to 282,354)	364,647 (346,037 to 385,291)	9.9 (9.5 to 10.4)	7.5 (6.9 to 8.1)	13 (12 to 13)
Andean Latin America	582 (498 to 682)	266 (212 to 340)	316 (264 to 380)	1.4 (1.2 to 1.7)	1.2 (0.9 to 1.5)	1.7 (1.4 to 2.1)
Australasia	8,731 (7,375 to 10,528)	3,993 (3,019 to 5,201)	4,739 (3,897 to 5,881)	20 (17 to 23)	16 (12 to 20)	25 (20 to 30)
Caribbean	10,548 (9,489 to 11,693)	4,891 (4,125 to 5,679)	5,657 (5,008 to 6,382)	24 (22 to 27)	20 (17 to 24)	29 (26 to 33)
Central Asia	2,352 (2,045 to 2,669)	734 (587 to 911)	1,618 (1,384 to 1,851)	4.0 (3.5 to 4.5)	2.2 (1.7 to 2.7)	6.5 (5.6 to 7.3)
Central Europe	41,745 (38,127 to 45,288)	14,163 (12,314 to 16,512)	27,581 (24,787 to 30,767)	22 (20 to 24)	12 (11 to 14)	35 (32 to 39)
Central Latin America	8,986 (8,261 to 9,790)	4,706 (4,183 to 5,258)	4,280 (3,886 to 4,728)	5.2 (4.8 to 5.6)	4.9 (4.3 to 5.4)	5.5 (5.0 to 6.1)
Central Sub-Saharan Africa	1,493 (938 to 2,366)	852 (423 to 1,679)	641 (392 to 1,057)	4.3 (2.7 to 6.8)	4.7 (2.4 to 9.0)	3.7 (2.3 to 5.8)
East Asia	24,257 (22,309 to 26,544)	8,230 (7,065 to 9,273)	16,027 (14,373 to 18,026)	1.7 (1.6 to 1.9)	1.1 (1.0 to 1.3)	2.4 (2.1 to 2.7)
Eastern Europe	108,354 (97,268 to 120,217)	42,023 (34,753 to 49,456)	66,331 (58,807 to 74,917)	33 (30 to 37)	19 (16 to 22)	56 (50 to 63)
Eastern Sub-Saharan Africa	4,437 (3,227 to 6,382)	2,159 (1,173 to 3,753)	2,277 (1,657 to 3,123)	3.7 (2.6 to 5.5)	3.5 (1.9 to 6.2)	3.9 (2.8 to 5.4)
High-income Asia Pacific	11,762 (10,818 to 12,788)	4,890 (4,228 to 5,719)	6,872 (6,323 to 7,457)	2.8 (2.6 to 3.1)	1.8 (1.6 to 2.1)	4.1 (3.8 to 4.4)
High-income North America	110,369 (102,125 to 119,198)	52,803 (45,799 to 59,660)	57,567 (53,087 to 62,640)	21 (20 to 23)	18 (16 to 20)	25 (23 to 27)
North Africa and Middle East	11,237 (10,155 to 12,551)	3,538 (3,034 to 4,074)	7,698 (6,775 to 8,766)	3.6 (3.3 to 4.1)	2.2 (1.9 to 2.6)	5.2 (4.6 to 6.0)

Oceania	263<br>(157 to 375)	69<br>(41 to 133)	194<br>(103 to 283)	5.0<br>(3.1 to 6.9)	2.8<br>(1.6 to 5.2)	7.7<br>(4.3 to 10.7)
South Asia	31,843<br>(26,717 to 38,861)	10,093<br>(7,208 to 17,011)	21,750<br>(15,361 to 28,499)	3.2<br>(2.7 to 3.9)	2.1<br>(1.5 to 3.6)	4.4<br>(3.1 to 5.7)
Southeast Asia	7,626<br>(6,544 to 9,234)	2,719<br>(2,128 to 3,451)	4,907<br>(4,059 to 6,082)	1.7<br>(1.5 to 2.1)	1.2<br>(0.9 to 1.5)	2.4<br>(2.0 to 3.0)
Southern Latin America	6,099<br>(5,458 to 6,789)	2,601<br>(2,141 to 3,107)	3,497<br>(3,051 to 4,003)	8.3<br>(7.4 to 9.1)	5.6<br>(4.7 to 6.7)	12<br>(11 to 14)
Southern Sub-Saharan Africa	18,222<br>(16,052 to 20,916)	6,482<br>(5,306 to 7,946)	11,741<br>(9,979 to 13,755)	42<br>(37 to 48)	26<br>(21 to 32)	63<br>(54 to 74)
Tropical Latin America	37,829<br>(34,608 to 42,055)	16,480<br>(14,398 to 19,030)	21,350<br>(19,082 to 24,113)	21<br>(19 to 24)	16<br>(14 to 19)	28<br>(25 to 31)
Western Europe	177,394<br>(164,238 to 191,151)	78,337<br>(69,479 to 88,718)	99,057<br>(90,671 to 107,661)	21<br>(20 to 23)	15<br>(13 to 17)	28<br>(26 to 30)
Western Sub-Saharan Africa	1,873<br>(1,376 to 2,752)	1,328<br>(891 to 2,217)	545<br>(375 to 843)	1.6<br>(1.2 to 2.2)	2.1<br>(1.5 to 3.4)	1.0<br>(0.7 to 1.6)

eTable 2: 2015 Global and Regional of YLDs for all CVD level 3 and 4 subcauses including 95% uncertainty, total number and age-standardized rate, by sex and total for 2015

	Years Lived with Disability (YLDs)					
	All Ages			Age-standardized (per 100,000)		
	Total	Female	Male	Total	Female	Male
**Cardiovascular diseases**						
Global	25,620,138<br>(18,401,575 to 33,656,619)	12,153,354<br>(8,787,238 to 15,872,403)	13,466,784<br>(9,616,125 to 17,652,994)	380<br>(274 to 498)	343<br>(248 to 448)	421<br>(301 to 551)
Andean Latin America	113,341<br>(80,268 to 150,399)	49,023<br>(35,166 to 64,580)	64,317<br>(44,822 to 85,583)	241<br>(172 to 318)	203<br>(146 to 267)	282<br>(199 to 373)
Australasia	97,598<br>(71,177 to 126,915)	45,809<br>(33,415 to 59,895)	51,789<br>(37,564 to 67,460)	249<br>(181 to 325)	218<br>(159 to 286)	283<br>(205 to 370)
Caribbean	152,639<br>(108,578 to 200,538)	70,718<br>(50,464 to 92,924)	81,921<br>(58,121 to 108,304)	349<br>(248 to 458)	307<br>(219 to 403)	396<br>(282 to 524)
Central Asia	272,967<br>(193,757 to 357,169)	144,891<br>(103,304 to 189,886)	128,076<br>(90,996 to 167,811)	422<br>(300 to 551)	400<br>(285 to 524)	450<br>(318 to 588)
Central Europe	812,093<br>(581,858 to 1,068,991)	400,755<br>(288,756 to 524,641)	411,339<br>(291,367 to 543,231)	458<br>(328 to 605)	392<br>(281 to 515)	538<br>(382 to 710)
Central Latin America	573,193<br>(405,075 to 766,177)	275,143<br>(194,421 to 366,154)	298,050<br>(207,978 to 399,985)	292<br>(207 to 389)	265<br>(188 to 352)	322<br>(226 to 430)
Central Sub-Saharan Africa	207,146<br>(147,540 to 273,236)	113,444<br>(81,275 to 150,304)	93,702<br>(66,934 to 124,291)	392<br>(283 to 513)	414<br>(299 to 549)	367<br>(265 to 479)
East Asia	6,157,341<br>(4,415,232 to 8,052,645)	2,729,922<br>(1,960,100 to 3,548,586)	3,427,419<br>(2,436,685 to 4,478,459)	405<br>(290 to 532)	352<br>(252 to 461)	456<br>(324 to 598)
Eastern Europe	1,309,704<br>(936,467 to 1,711,540)	739,882<br>(530,940 to 960,383)	569,822<br>(405,985 to 752,052)	415<br>(296 to 543)	371<br>(266 to 483)	480<br>(342 to 632)
Eastern Sub-Saharan Africa	898,612<br>(644,379 to 1,192,276)	482,802<br>(346,269 to 632,138)	415,810<br>(292,368 to 559,993)	535<br>(382 to 708)	560<br>(404 to 737)	504<br>(357 to 672)
High-income Asia Pacific	863,666<br>(629,465 to 1,126,415)	411,522<br>(299,280 to 538,977)	452,144<br>(329,225 to 591,515)	251<br>(182 to 328)	211<br>(153 to 274)	297<br>(215 to 389)
High-income North America	1,873,765<br>(1,366,064 to 2,456,041)	907,568<br>(664,458 to 1,188,183)	966,197<br>(703,723 to 1,265,372)	375<br>(273 to 491)	328<br>(240 to 430)	429<br>(312 to 562)
North Africa and Middle East	1,972,018<br>(1,392,330 to 2,623,101)	898,283<br>(635,004 to 1,188,618)	1,073,735<br>(753,225 to 1,435,632)	489<br>(349 to 647)	452<br>(322 to 596)	528<br>(377 to 698)
Oceania	33,684<br>(24,068 to 44,243)	16,164<br>(11,612 to 21,192)	17,520<br>(12,468 to 23,140)	450<br>(325 to 585)	430<br>(313 to 559)	473<br>(337 to 615)
South Asia	4,014,086<br>(2,888,776 to 5,274,493)	1,806,938<br>(1,306,206 to 2,369,385)	2,207,148<br>(1,575,314 to 2,921,608)	325<br>(234 to 425)	290<br>(210 to 379)	360<br>(258 to 472)
Southeast Asia	2,020,718<br>(1,437,149 to 2,637,793)	974,026<br>(699,464 to 1,275,889)	1,046,692<br>(743,565 to 1,375,268)	391<br>(279 to 510)	358<br>(258 to 469)	425<br>(302 to 556)
Southern Latin America	166,762<br>(119,609 to 218,472)	87,348<br>(62,550 to 114,535)	79,414<br>(57,120 to 104,265)	235<br>(168 to 308)	213<br>(152 to 280)	265<br>(191 to 347)
Southern Sub-Saharan Africa	193,053<br>(139,524 to 251,231)	106,683<br>(77,528 to 138,529)	86,370<br>(61,669 to 111,854)	422<br>(307 to 547)	413<br>(302 to 537)	423<br>(304 to 548)
Tropical Latin America	780,085<br>(554,095 to 1,038,496)	346,108<br>(247,942 to 460,233)	433,977<br>(304,877 to 579,732)	406<br>(290 to 538)	332<br>(238 to 441)	494<br>(350 to 659)
Western Europe	2,050,932<br>(1,478,472 to 2,709,850)	995,368<br>(721,161 to 1,309,241)	1,055,564<br>(762,279 to 1,401,311)	277<br>(199 to 366)	237<br>(170 to 313)	320<br>(231 to 426)
Western Sub-Saharan Africa	1,056,737<br>(753,667 to 1,393,913)	550,957<br>(393,878 to 728,083)	505,779<br>(356,982 to 666,514)	617<br>(443 to 815)	651<br>(469 to 863)	580<br>(416 to 761)
**Atrial fibrillation and flutter**						
Global	2,634,581<br>(1,782,603 to 3,637,262)	1,120,738<br>(756,014 to 1,548,898)	1,513,842<br>(1,022,789 to 2,089,929)	41<br>(28 to 57)	32<br>(22 to 44)	51<br>(35 to 71)
Andean Latin America	9,774<br>(6,557 to 13,541)	3,833<br>(2,575 to 5,335)	5,941<br>(3,963 to 8,311)	23<br>(16 to 33)	17<br>(12 to 24)	31<br>(21 to 43)
Australasia	19,039<br>(12,998 to 26,069)	7,104<br>(4,860 to 9,800)	11,935<br>(8,060 to 16,207)	49<br>(33 to 67)	33<br>(23 to 46)	65<br>(44 to 89)
Caribbean	10,074<br>(6,725 to 13,886)	3,961<br>(2,653 to 5,518)	6,114<br>(4,123 to 8,432)	24<br>(16 to 32)	17<br>(11 to 24)	31<br>(21 to 43)
Central Asia	30,050<br>(19,886 to 41,529)	13,566<br>(9,054 to 18,723)	16,484<br>(10,957 to 22,998)	49<br>(33 to 68)	40<br>(27 to 55)	60<br>(40 to 84)
Central Europe	107,768<br>(74,873 to 146,496)	53,808<br>(37,114 to 73,499)	53,960<br>(37,312 to 73,711)	59<br>(41 to 80)	50<br>(34 to 68)	69<br>(48 to 95)
Central Latin America	41,885<br>(27,850 to 57,647)	16,531<br>(11,100 to 22,801)	25,353<br>(16,971 to 34,985)	24<br>(16 to 32)	17<br>(12 to 24)	31<br>(21 to 43)
Central Sub-Saharan Africa	7,848<br>(5,315 to 10,774)	3,072<br>(2,050 to 4,291)	4,776<br>(3,204 to 6,623)	21<br>(14 to 28)	15<br>(10 to 21)	28<br>(19 to 38)
East Asia	529,184<br>(350,483 to 742,168)	225,110<br>(149,020 to 315,950)	304,074<br>(201,520 to 428,833)	35<br>(23 to 49)	29<br>(19 to 40)	41<br>(27 to 58)
Eastern Europe	148,831<br>(100,509 to 205,437)	79,923<br>(53,786 to 110,616)	68,909<br>(45,974 to 95,428)	47<br>(32 to 65)	38<br>(26 to 53)	58<br>(39 to 81)
Eastern Sub-Saharan Africa	26,549<br>(17,813 to 36,766)	10,311<br>(6,902 to 14,527)	16,238<br>(10,897 to 22,538)	20<br>(14 to 28)	14.8<br>(10.0 to 20.6)	27<br>(18 to 37)
High-income Asia Pacific	89,723<br>(59,705 to 124,296)	26,744<br>(17,951 to 36,926)	62,979<br>(41,849 to 88,022)	24<br>(16 to 34)	11.7<br>(7.8 to 16.3)	39<br>(26 to 55)
High-income North America	491,194<br>(345,118 to 668,254)	215,350<br>(151,456 to 292,996)	275,844<br>(192,995 to 374,608)	96<br>(67 to 130)	73<br>(51 to 100)	121<br>(85 to 165)
North Africa and Middle East	74,542<br>(49,217 to 103,048)	29,308<br>(19,635 to 40,569)	45,235<br>(29,844 to 62,434)	24<br>(16 to 33)	18<br>(12 to 25)	30<br>(20 to 42)
Oceania	1,766<br>(1,184 to 2,456)	727<br>(483 to 1,005)	1,038<br>(699 to 1,463)	34<br>(23 to 47)	27<br>(18 to 37)	44<br>(29 to 60)
South Asia	305,607<br>(206,039 to 422,781)	112,003<br>(75,844 to 153,441)	193,605<br>(129,288 to 267,828)	30<br>(20 to 41)	21<br>(14 to 29)	39<br>(26 to 53)
Southeast Asia	159,857<br>(106,421 to 222,052)	68,310<br>(45,775 to 94,938)	91,547<br>(60,545 to 127,229)	35<br>(23 to 48)	27<br>(18 to 38)	44<br>(30 to 61)
Southern Latin America	29,094<br>(19,312 to 40,154)	10,711<br>(7,143 to 14,883)	18,383<br>(12,142 to 25,479)	41<br>(27 to 56)	25<br>(17 to 35)	61<br>(41 to 85)
Southern Sub-Saharan Africa	8,678<br>(5,840 to 11,917)	3,850<br>(2,587 to 5,364)	4,828<br>(3,254 to 6,608)	21<br>(14 to 28)	15<br>(10 to 22)	28<br>(19 to 39)
Tropical Latin America	43,148<br>(28,992 to 60,021)	18,011<br>(12,194 to 25,143)	25,137<br>(16,958 to 34,897)	24<br>(16 to 33)	18<br>(12 to 25)	32<br>(22 to 45)
Western Europe	472,856<br>(319,760 to 653,087)	208,312<br>(141,652 to 285,839)	264,543<br>(177,781 to 362,892)	63<br>(42 to 87)	47<br>(31 to 64)	80<br>(54 to 110)
Western Sub-Saharan Africa	27,115<br>(18,155 to 38,150)	10,193<br>(6,819 to 14,362)	16,922<br>(11,220 to 23,438)	21<br>(14 to 29)	15<br>(10 to 22)	28<br>(19 to 39)
**Cardiomyopathy and myocarditis**						
Global	275,097<br>(190,348 to 377,894)	149,814<br>(103,872 to 205,621)	125,283<br>(87,201 to 172,831)	4.1<br>(2.9 to 5.7)	4.2<br>(2.9 to 5.7)	3.9<br>(2.7 to 5.4)
Andean Latin America	1,256<br>(847 to 1,741)	691<br>(464 to 964)	566<br>(384 to 796)	2.6<br>(1.8 to 3.6)	2.7<br>(1.8 to 3.8)	2.4<br>(1.7 to 3.4)
Australasia	2,554<br>(1,749 to 3,526)	1,401<br>(950 to 1,946)	1,154<br>(785 to 1,591)	6.5<br>(4.4 to 9.0)	6.2<br>(4.2 to 8.6)	6.7<br>(4.5 to 9.2)
Caribbean	2,552<br>(1,766 to 3,530)	1,324<br>(902 to 1,843)	1,227<br>(844 to 1,705)	5.7<br>(4.0 to 8.0)	5.6<br>(3.8 to 7.8)	5.8<br>(4.0 to 8.1)
Central Asia	1,147<br>(783 to 1,581)	540<br>(369 to 745)	607<br>(417 to 842)	1.5<br>(1.1 to 2.1)	1.4<br>(1.0 to 1.9)	1.6<br>(1.1 to 2.3)

Central Europe	9,167 (6,263 to 12,540)	5,281 (3,650 to 7,233)	3,886 (2,655 to 5,303)	5.3 (3.6 to 7.3)	4.9 (3.4 to 6.7)	5.5 (3.8 to 7.6)
Central Latin America	9,093 (6,226 to 12,476)	4,841 (3,363 to 6,627)	4,252 (2,870 to 5,912)	4.2 (2.9 to 5.8)	4.3 (3.0 to 5.9)	4.1 (2.8 to 5.6)
Central Sub-Saharan Africa	4,374 (2,968 to 6,066)	2,096 (1,437 to 2,933)	2,278 (1,533 to 3,183)	8.0 (5.5 to 11.1)	7.5 (5.2 to 10.3)	8.6 (5.9 to 12.0)
East Asia	28,495 (19,802 to 39,340)	16,044 (11,058 to 22,088)	12,451 (8,638 to 17,188)	2.2 (1.5 to 3.1)	2.5 (1.7 to 3.4)	1.8 (1.3 to 2.5)
Eastern Europe	12,038 (8,110 to 16,764)	6,240 (4,204 to 8,768)	5,798 (3,881 to 8,134)	4.2 (2.8 to 5.9)	3.4 (2.3 to 4.8)	5.0 (3.4 to 7.0)
Eastern Sub-Saharan Africa	11,303 (7,566 to 15,865)	5,366 (3,590 to 7,536)	5,937 (3,982 to 8,429)	6.0 (4.1 to 8.5)	5.4 (3.7 to 7.6)	6.7 (4.5 to 9.4)
High-income Asia Pacific	16,345 (11,264 to 22,596)	10,532 (7,230 to 14,535)	5,813 (4,019 to 8,022)	4.9 (3.4 to 6.8)	5.0 (3.5 to 7.0)	4.5 (3.1 to 6.1)
High-income North America	49,397 (34,706 to 67,877)	27,657 (19,091 to 37,610)	21,740 (15,202 to 29,834)	10.0 (7.0 to 13.8)	9.5 (6.6 to 12.9)	10.2 (7.1 to 14.0)
North Africa and Middle East	18,191 (12,722 to 25,105)	8,933 (6,223 to 12,247)	9,258 (6,409 to 12,832)	4.5 (3.2 to 6.2)	4.5 (3.2 to 6.2)	4.4 (3.1 to 6.1)
Oceania	202 (139 to 278)	113 (78 to 155)	89 (61 to 124)	3.4 (2.4 to 4.6)	3.9 (2.7 to 5.4)	2.6 (1.8 to 3.5)
South Asia	15,560 (10,818 to 21,566)	8,508 (5,858 to 11,850)	7,053 (4,885 to 9,782)	1.2 (0.8 to 1.6)	1.4 (1.0 to 1.9)	0.9 (0.6 to 1.3)
Southeast Asia	17,717 (12,295 to 24,476)	9,273 (6,326 to 12,836)	8,443 (5,841 to 11,697)	3.6 (2.5 to 4.9)	3.6 (2.5 to 4.9)	3.5 (2.4 to 4.8)
Southern Latin America	3,330 (2,218 to 4,609)	2,243 (1,505 to 3,090)	1,086 (698 to 1,551)	4.5 (3.0 to 6.2)	4.8 (3.2 to 6.8)	3.7 (2.4 to 5.2)
Southern Sub-Saharan Africa	6,993 (4,914 to 9,595)	3,566 (2,497 to 4,871)	3,427 (2,379 to 4,704)	15 (10 to 20)	13.1 (9.2 to 17.8)	17 (12 to 24)
Tropical Latin America	20,263 (14,017 to 27,919)	10,064 (6,980 to 13,717)	10,199 (7,034 to 14,043)	10.9 (7.6 to 15.0)	10.0 (6.9 to 13.6)	11.9 (8.3 to 16.5)
Western Europe	27,870 (19,348 to 38,356)	17,650 (12,274 to 23,967)	10,220 (6,989 to 14,239)	3.6 (2.5 to 4.9)	3.5 (2.4 to 4.8)	3.4 (2.3 to 4.7)
Western Sub-Saharan Africa	17,248 (12,021 to 23,890)	7,451 (5,189 to 10,415)	9,797 (6,799 to 13,596)	9.5 (6.6 to 13.0)	8.2 (5.7 to 11.2)	10.9 (7.6 to 15.1)
**Endocarditis**						
Global	12,325 (8,509 to 17,114)	9,076 (6,284 to 12,591)	3,248 (2,202 to 4,525)	0.2 (0.1 to 0.3)	0.3 (0.2 to 0.4)	0.1 (0.1 to 0.1)
Andean Latin America	17 (11 to 23)	12.1 (8.3 to 17.1)	4.5 (2.9 to 6.5)	0.0 (0.0 to 0.1)	0.0 (0.0 to 0.1)	0.0 (0.0 to 0.0)
Australasia	62 (41 to 88)	29 (19 to 43)	32 (21 to 46)	0.2 (0.1 to 0.2)	0.2 (0.1 to 0.2)	0.2 (0.1 to 0.3)
Caribbean	131 (91 to 181)	125 (86 to 172)	6.0 (4.1 to 8.5)	0.3 (0.2 to 0.4)	0.5 (0.4 to 0.7)	0.0 (0.0 to 0.0)
Central Asia	69 (47 to 98)	36 (25 to 51)	33 (22 to 46)	0.1 (0.1 to 0.2)	0.1 (0.1 to 0.1)	0.1 (0.1 to 0.2)
Central Europe	123 (85 to 172)	32 (21 to 45)	91 (62 to 129)	0.1 (0.1 to 0.1)	0.1 (0.0 to 0.1)	0.1 (0.1 to 0.2)
Central Latin America	223 (150 to 310)	147 (99 to 204)	76 (50 to 107)	0.1 (0.1 to 0.1)	0.1 (0.1 to 0.2)	0.1 (0.1 to 0.1)
Central Sub-Saharan Africa	60 (40 to 83)	35 (24 to 49)	25 (17 to 34)	0.1 (0.1 to 0.1)	0.1 (0.1 to 0.1)	0.1 (0.1 to 0.1)
East Asia	714 (475 to 1,035)	319 (207 to 463)	394 (261 to 570)	0.1 (0.0 to 0.1)	0.0 (0.0 to 0.1)	0.1 (0.0 to 0.1)
Eastern Europe	248 (170 to 351)	150 (101 to 212)	99 (67 to 138)	0.1 (0.1 to 0.1)	0.1 (0.1 to 0.1)	0.1 (0.1 to 0.1)
Eastern Sub-Saharan Africa	212 (142 to 295)	120 (80 to 167)	92 (62 to 128)	0.1 (0.1 to 0.1)	0.1 (0.1 to 0.1)	0.1 (0.1 to 0.1)
High-income Asia Pacific	440 (294 to 625)	264 (173 to 380)	175 (117 to 253)	0.1 (0.1 to 0.2)	0.2 (0.1 to 0.2)	0.1 (0.1 to 0.2)
High-income North America	1,313 (885 to 1,863)	664 (438 to 935)	649 (440 to 932)	0.3 (0.2 to 0.4)	0.3 (0.2 to 0.4)	0.3 (0.2 to 0.4)
North Africa and Middle East	1,921 (1,328 to 2,640)	1,804 (1,247 to 2,479)	117 (79 to 163)	0.6 (0.4 to 0.8)	1.1 (0.7 to 1.4)	0.0 (0.0 to 0.1)
Oceania	65 (46 to 89)	48 (34 to 66)	17 (11 to 23)	1.4 (1.0 to 2.0)	1.9 (1.4 to 2.6)	0.8 (0.6 to 1.1)
South Asia	1,302 (858 to 1,837)	715 (473 to 1,023)	586 (387 to 822)	0.1 (0.1 to 0.1)	0.1 (0.1 to 0.2)	0.1 (0.1 to 0.1)
Southeast Asia	507 (339 to 704)	292 (193 to 407)	215 (145 to 297)	0.1 (0.1 to 0.1)	0.1 (0.1 to 0.1)	0.1 (0.1 to 0.1)
Southern Latin America	594 (414 to 836)	590 (410 to 831)	4.2 (1.7 to 7.2)	0.8 (0.5 to 1.1)	1.3 (0.9 to 1.8)	0.0 (0.0 to 0.0)
Southern Sub-Saharan Africa	41 (27 to 59)	22 (15 to 32)	19 (12 to 27)	0.1 (0.0 to 0.1)	0.1 (0.1 to 0.1)	0.0 (0.0 to 0.1)
Tropical Latin America	226 (149 to 329)	105 (67 to 156)	120 (80 to 174)	0.1 (0.1 to 0.2)	0.1 (0.1 to 0.2)	0.1 (0.1 to 0.2)
Western Europe	3,811 (2,663 to 5,249)	3,428 (2,395 to 4,737)	383 (262 to 535)	0.4 (0.3 to 0.6)	0.7 (0.5 to 0.9)	0.1 (0.1 to 0.2)
Western Sub-Saharan Africa	248 (168 to 345)	139 (94 to 193)	109 (73 to 151)	0.1 (0.1 to 0.2)	0.1 (0.1 to 0.2)	0.1 (0.1 to 0.1)
**Hemorrhagic and other stroke**						
Global	2,795,183 (1,945,790 to 3,749,593)	1,326,256 (915,041 to 1,777,544)	1,468,927 (1,023,737 to 1,971,159)	40 (28 to 53)	37 (26 to 50)	43 (30 to 58)
Andean Latin America	12,480 (8,643 to 16,708)	6,795 (4,719 to 9,197)	5,684 (3,899 to 7,665)	25 (17 to 33)	26 (18 to 35)	23 (16 to 31)
Australasia	6,827 (4,740 to 9,090)	3,689 (2,580 to 4,949)	3,138 (2,189 to 4,209)	18 (13 to 25)	19 (14 to 26)	18 (12 to 24)
Caribbean	17,206 (11,825 to 23,152)	9,134 (6,333 to 12,198)	8,072 (5,607 to 10,904)	39 (27 to 52)	40 (28 to 53)	38 (26 to 51)
Central Asia	42,683 (29,769 to 57,046)	23,313 (16,235 to 31,231)	19,370 (13,457 to 25,908)	60 (42 to 80)	60 (41 to 80)	60 (42 to 80)
Central Europe	71,096 (50,000 to 94,550)	41,129 (28,909 to 54,890)	29,967 (20,831 to 39,778)	44 (31 to 60)	48 (33 to 64)	41 (29 to 55)
Central Latin America	27,363 (18,914 to 36,967)	14,161 (9,737 to 19,242)	13,202 (9,146 to 17,852)	12.9 (8.9 to 17.4)	12.8 (8.8 to 17.3)	13.1 (9.1 to 17.6)
Central Sub-Saharan Africa	26,845 (18,654 to 36,206)	15,448 (10,807 to 20,634)	11,397 (7,900 to 15,498)	48 (33 to 64)	53 (37 to 71)	41 (29 to 56)
East Asia	745,389 (515,228 to 1,006,704)	302,927 (207,379 to 409,209)	442,462 (307,566 to 598,057)	46 (32 to 63)	38 (26 to 52)	54 (38 to 73)
Eastern Europe	164,286 (114,259 to 217,043)	99,163 (68,732 to 131,764)	65,123 (44,530 to 86,459)	55 (38 to 73)	56 (38 to 74)	55 (38 to 73)
Eastern Sub-Saharan Africa	126,816 (88,331 to 171,716)	69,355 (48,428 to 94,228)	57,461 (39,908 to 77,248)	60 (42 to 80)	64 (45 to 86)	55 (38 to 74)
High-income Asia Pacific	87,483 (60,892 to 116,811)	43,860 (30,538 to 58,761)	43,623 (30,220 to 58,187)	31 (21 to 41)	29 (21 to 40)	32 (22 to 43)
High-income North America	58,908 (41,249 to 79,190)	30,442 (21,326 to 40,893)	28,466 (19,926 to 38,188)	12.5 (8.7 to 16.8)	12.1 (8.4 to 16.3)	13.0 (9.0 to 17.4)
North Africa and Middle East	163,831 (114,512 to 219,253)	88,627 (62,101 to 118,545)	75,204 (52,379 to 100,980)	37 (26 to 50)	40 (28 to 54)	34 (24 to 46)

Oceania	5,809<br>(4,030 to 7,805)	2,995<br>(2,076 to 4,019)	2,814<br>(1,944 to 3,802)	72<br>(50 to 96)	74<br>(51 to 98)	71<br>(49 to 95)
South Asia	594,221<br>(417,010 to 801,637)	257,852<br>(179,580 to 347,807)	336,368<br>(235,042 to 456,845)	42<br>(30 to 57)	37<br>(26 to 50)	47<br>(33 to 64)
Southeast Asia	307,742<br>(214,972 to 412,393)	146,119<br>(100,407 to 194,620)	161,623<br>(112,302 to 217,256)	53<br>(37 to 71)	49<br>(34 to 65)	58<br>(40 to 77)
Southern Latin America	19,019<br>(13,053 to 25,430)	10,315<br>(7,065 to 13,870)	8,704<br>(6,030 to 11,644)	28<br>(19 to 37)	28<br>(19 to 38)	28<br>(19 to 37)
Southern Sub-Saharan Africa	27,637<br>(19,233 to 37,166)	13,957<br>(9,608 to 18,718)	13,680<br>(9,447 to 18,372)	48<br>(34 to 64)	46<br>(32 to 61)	51<br>(35 to 68)
Tropical Latin America	43,505<br>(30,275 to 58,093)	22,150<br>(15,390 to 29,770)	21,355<br>(14,729 to 28,547)	22<br>(15 to 29)	20<br>(14 to 27)	23<br>(16 to 31)
Western Europe	136,363<br>(95,256 to 182,659)	70,885<br>(49,813 to 94,985)	65,478<br>(45,561 to 87,685)	21<br>(15 to 28)	21<br>(14 to 28)	22<br>(15 to 29)
Western Sub-Saharan Africa	109,674<br>(76,219 to 148,294)	53,940<br>(37,364 to 73,128)	55,734<br>(38,855 to 75,521)	48<br>(33 to 64)	48<br>(34 to 65)	46<br>(32 to 62)
**Hypertensive heart disease**						
Global	669,988<br>(467,103 to 917,055)	369,694<br>(258,706 to 507,247)	300,295<br>(209,826 to 411,504)	10.5<br>(7.3 to 14.4)	10.5<br>(7.4 to 14.4)	10.5<br>(7.3 to 14.3)
Andean Latin America	3,220<br>(2,205 to 4,438)	1,899<br>(1,290 to 2,624)	1,321<br>(901 to 1,820)	7.8<br>(5.4 to 10.8)	8.4<br>(5.7 to 11.7)	7.2<br>(5.0 to 9.9)
Australasia	1,826<br>(1,238 to 2,528)	954<br>(648 to 1,339)	872<br>(586 to 1,219)	4.3<br>(2.9 to 6.0)	4.1<br>(2.7 to 5.7)	4.6<br>(3.1 to 6.4)
Caribbean	7,084<br>(4,952 to 9,724)	3,691<br>(2,560 to 5,024)	3,393<br>(2,340 to 4,625)	16<br>(12 to 23)	16<br>(11 to 22)	17<br>(12 to 24)
Central Asia	3,884<br>(2,723 to 5,382)	2,343<br>(1,626 to 3,223)	1,541<br>(1,073 to 2,146)	7.0<br>(4.9 to 9.7)	7.1<br>(4.9 to 9.8)	6.7<br>(4.6 to 9.2)
Central Europe	14,573<br>(10,164 to 19,944)	9,711<br>(6,773 to 13,343)	4,862<br>(3,395 to 6,662)	7.5<br>(5.2 to 10.2)	7.9<br>(5.5 to 10.9)	6.5<br>(4.5 to 8.9)
Central Latin America	22,986<br>(16,024 to 31,517)	13,941<br>(9,711 to 19,091)	9,045<br>(6,307 to 12,380)	13.2<br>(9.2 to 18.0)	14<br>(10 to 20)	11.8<br>(8.3 to 16.2)
Central Sub-Saharan Africa	7,464<br>(5,158 to 10,273)	3,448<br>(2,395 to 4,727)	4,015<br>(2,759 to 5,462)	22<br>(15 to 30)	19<br>(13 to 26)	26<br>(18 to 35)
East Asia	203,246<br>(142,742 to 279,570)	104,183<br>(72,881 to 143,753)	99,063<br>(68,882 to 136,401)	14.1<br>(9.9 to 19.4)	13.9<br>(9.7 to 19.2)	14.3<br>(10.0 to 19.7)
Eastern Europe	10,371<br>(7,114 to 14,111)	7,678<br>(5,283 to 10,450)	2,693<br>(1,849 to 3,760)	3.1<br>(2.1 to 4.2)	3.4<br>(2.3 to 4.5)	2.4<br>(1.7 to 3.4)
Eastern Sub-Saharan Africa	20,678<br>(14,286 to 28,401)	9,383<br>(6,426 to 12,845)	11,294<br>(7,868 to 15,564)	17<br>(12 to 24)	14<br>(10 to 20)	21<br>(15 to 29)
High-income Asia Pacific	36,570<br>(25,529 to 50,242)	24,322<br>(16,949 to 33,423)	12,248<br>(8,533 to 16,844)	8.7<br>(6.1 to 12.0)	9.7<br>(6.7 to 13.3)	7.3<br>(5.1 to 10.0)
High-income North America	53,323<br>(37,119 to 72,955)	31,462<br>(21,978 to 43,024)	21,861<br>(15,140 to 29,809)	10.3<br>(7.2 to 14.0)	10.6<br>(7.4 to 14.6)	9.6<br>(6.6 to 13.0)
North Africa and Middle East	22,341<br>(15,471 to 30,586)	14,882<br>(10,369 to 20,367)	7,459<br>(5,106 to 10,222)	7.3<br>(5.1 to 10.0)	9.1<br>(6.4 to 12.5)	5.3<br>(3.7 to 7.3)
Oceania	273<br>(189 to 371)	157<br>(110 to 215)	116<br>(80 to 160)	6.1<br>(4.3 to 8.4)	6.4<br>(4.5 to 8.7)	5.8<br>(4.0 to 8.0)
South Asia	124,727<br>(87,802 to 170,494)	63,801<br>(44,936 to 86,940)	60,926<br>(42,716 to 83,241)	12.4<br>(8.7 to 17.0)	12.3<br>(8.7 to 16.8)	12.5<br>(8.7 to 17.2)
Southeast Asia	41,151<br>(28,784 to 56,981)	25,131<br>(17,600 to 34,679)	16,020<br>(10,985 to 22,306)	9.5<br>(6.7 to 13.3)	10.4<br>(7.3 to 14.5)	8.4<br>(5.8 to 11.6)
Southern Latin America	6,312<br>(4,206 to 8,743)	4,085<br>(2,769 to 5,724)	2,227<br>(1,472 to 3,090)	8.5<br>(5.7 to 11.7)	8.9<br>(6.0 to 12.5)	7.8<br>(5.2 to 10.8)
Southern Sub-Saharan Africa	5,658<br>(4,022 to 7,738)	2,844<br>(2,006 to 3,870)	2,814<br>(1,978 to 3,845)	14<br>(10 to 19)	11.7<br>(8.3 to 15.9)	19<br>(13 to 25)
Tropical Latin America	29,291<br>(20,295 to 40,427)	16,732<br>(11,655 to 23,127)	12,559<br>(8,708 to 17,300)	17<br>(12 to 23)	17<br>(12 to 23)	17<br>(12 to 23)
Western Europe	28,859<br>(19,875 to 39,949)	18,081<br>(12,423 to 24,931)	10,778<br>(7,346 to 14,847)	3.1<br>(2.2 to 4.4)	3.2<br>(2.2 to 4.5)	2.9<br>(2.0 to 4.1)
Western Sub-Saharan Africa	26,153<br>(18,503 to 35,824)	10,966<br>(7,650 to 15,010)	15,187<br>(10,688 to 20,717)	23<br>(16 to 32)	19<br>(13 to 26)	28<br>(20 to 38)
**Ischemic heart disease**						
Global	7,274,736<br>(4,958,633 to 9,940,309)	3,262,511<br>(2,231,014 to 4,442,745)	4,012,225<br>(2,730,805 to 5,530,231)	110<br>(75 to 150)	93<br>(63 to 126)	128<br>(88 to 176)
Andean Latin America	33,174<br>(22,449 to 45,219)	14,810<br>(10,078 to 20,292)	18,365<br>(12,402 to 25,013)	77<br>(52 to 105)	65<br>(44 to 89)	90<br>(61 to 122)
Australasia	26,747<br>(18,634 to 36,530)	12,209<br>(8,564 to 16,573)	14,538<br>(10,032 to 19,860)	67<br>(46 to 91)	56<br>(39 to 76)	78<br>(54 to 107)
Caribbean	52,881<br>(36,330 to 72,745)	23,581<br>(16,233 to 32,401)	29,299<br>(20,291 to 40,180)	122<br>(84 to 168)	102<br>(70 to 140)	145<br>(100 to 198)
Central Asia	109,789<br>(74,893 to 151,471)	60,092<br>(40,906 to 82,556)	49,696<br>(33,913 to 68,975)	176<br>(120 to 242)	170<br>(115 to 232)	184<br>(125 to 254)
Central Europe	304,630<br>(208,034 to 418,598)	138,164<br>(94,571 to 189,739)	166,466<br>(113,074 to 228,394)	169<br>(115 to 232)	132<br>(90 to 181)	215<br>(147 to 295)
Central Latin America	218,215<br>(149,390 to 300,906)	103,468<br>(70,889 to 141,561)	114,747<br>(78,298 to 158,682)	117<br>(80 to 160)	103<br>(71 to 141)	132<br>(90 to 182)
Central Sub-Saharan Africa	14,685<br>(10,076 to 20,241)	6,562<br>(4,493 to 9,003)	8,123<br>(5,577 to 11,174)	34<br>(24 to 47)	29<br>(20 to 40)	40<br>(28 to 55)
East Asia	1,407,888<br>(949,403 to 1,940,296)	611,494<br>(410,795 to 839,671)	796,394<br>(539,574 to 1,101,829)	88<br>(59 to 121)	77<br>(52 to 105)	100<br>(67 to 137)
Eastern Europe	607,365<br>(407,796 to 832,928)	319,771<br>(215,200 to 438,104)	287,594<br>(193,398 to 397,727)	190<br>(127 to 260)	156<br>(105 to 214)	241<br>(162 to 332)
Eastern Sub-Saharan Africa	201,488<br>(132,469 to 280,440)	82,465<br>(54,720 to 114,426)	119,023<br>(78,138 to 168,580)	133<br>(88 to 183)	104<br>(69 to 144)	166<br>(109 to 233)
High-income Asia Pacific	235,301<br>(164,174 to 321,827)	115,922<br>(81,117 to 157,760)	119,379<br>(83,081 to 164,015)	63<br>(44 to 87)	52<br>(36 to 71)	75<br>(52 to 103)
High-income North America	533,547<br>(376,872 to 722,963)	253,083<br>(178,292 to 343,085)	280,464<br>(197,645 to 381,053)	103<br>(73 to 140)	87<br>(61 to 118)	123<br>(86 to 167)
North Africa and Middle East	485,724<br>(332,330 to 671,890)	214,792<br>(147,136 to 294,531)	270,932<br>(184,709 to 378,172)	142<br>(98 to 195)	123<br>(85 to 168)	161<br>(111 to 223)
Oceania	4,475<br>(3,077 to 6,116)	2,053<br>(1,411 to 2,792)	2,422<br>(1,664 to 3,340)	79<br>(55 to 108)	72<br>(49 to 97)	87<br>(60 to 119)
South Asia	1,489,690<br>(1,007,634 to 2,052,674)	609,190<br>(414,322 to 828,525)	880,500<br>(593,027 to 1,216,961)	132<br>(90 to 181)	110<br>(75 to 150)	154<br>(104 to 212)
Southeast Asia	527,905<br>(356,289 to 729,512)	246,027<br>(166,897 to 336,237)	281,878<br>(187,196 to 392,703)	108<br>(73 to 147)	96<br>(65 to 130)	120<br>(81 to 167)
Southern Latin America	28,475<br>(19,290 to 38,354)	14,194<br>(9,742 to 18,987)	14,281<br>(9,693 to 19,338)	39<br>(27 to 53)	33<br>(22 to 44)	48<br>(33 to 65)
Southern Sub-Saharan Africa	40,815<br>(27,344 to 56,358)	17,393<br>(11,701 to 23,867)	23,421<br>(15,618 to 32,727)	88<br>(59 to 121)	66<br>(45 to 91)	116<br>(78 to 160)
Tropical Latin America	273,909<br>(187,077 to 376,880)	114,628<br>(78,748 to 156,305)	159,281<br>(108,358 to 220,704)	145<br>(99 to 198)	111<br>(76 to 151)	185<br>(126 to 254)
Western Europe	542,221<br>(369,725 to 742,222)	258,514<br>(176,961 to 350,841)	283,707<br>(192,859 to 386,405)	72<br>(48 to 98)	60<br>(41 to 83)	85<br>(57 to 116)
Western Sub-Saharan Africa	135,813<br>(88,567 to 191,105)	44,097<br>(29,149 to 61,692)	91,716<br>(60,469 to 130,760)	84<br>(55 to 117)	55<br>(37 to 77)	115<br>(75 to 160)
**Ischemic stroke**						

Global	3,659,982 (2,559,278 to 4,874,934)	1,741,480 (1,221,066 to 2,321,574)	1,918,502 (1,339,942 to 2,554,020)	55 (38 to 73)	50 (35 to 66)	61 (43 to 81)
Andean Latin America	33,174 (22,449 to 45,219)	14,810 (10,078 to 20,292)	18,365 (12,402 to 25,013)	77 (52 to 105)	65 (44 to 89)	90 (61 to 122)
Australasia	26,747 (18,634 to 36,530)	12,209 (8,564 to 16,573)	14,538 (10,032 to 19,860)	67 (46 to 91)	56 (39 to 76)	78 (54 to 107)
Caribbean	52,881 (36,330 to 72,745)	23,581 (16,233 to 32,401)	29,299 (20,291 to 40,180)	122 (84 to 168)	102 (70 to 140)	145 (100 to 198)
Central Asia	109,789 (74,893 to 151,471)	60,092 (40,906 to 82,556)	49,696 (33,913 to 68,975)	176 (120 to 242)	170 (115 to 232)	184 (125 to 254)
Central Europe	304,630 (208,034 to 418,598)	138,164 (94,571 to 189,739)	166,466 (113,074 to 228,394)	169 (115 to 232)	132 (90 to 181)	215 (147 to 295)
Central Latin America	218,215 (149,390 to 300,906)	103,468 (70,889 to 141,561)	114,747 (78,298 to 158,682)	117 (80 to 160)	103 (71 to 141)	132 (90 to 182)
Central Sub-Saharan Africa	14,685 (10,076 to 20,241)	6,562 (4,493 to 9,003)	8,123 (5,577 to 11,174)	34 (24 to 47)	29 (20 to 40)	40 (28 to 55)
East Asia	1,407,888 (949,403 to 1,940,296)	611,494 (410,795 to 839,671)	796,394 (539,574 to 1,101,829)	88 (59 to 121)	77 (52 to 105)	100 (67 to 137)
Eastern Europe	607,365 (407,796 to 832,928)	319,771 (215,200 to 438,104)	287,594 (193,398 to 397,727)	190 (127 to 260)	156 (105 to 214)	241 (162 to 332)
Eastern Sub-Saharan Africa	201,488 (132,469 to 280,440)	82,465 (54,720 to 114,426)	119,023 (78,138 to 168,580)	133 (88 to 183)	104 (69 to 144)	166 (109 to 233)
High-income Asia Pacific	235,301 (164,174 to 321,827)	115,922 (81,117 to 157,760)	119,379 (83,081 to 164,015)	63 (44 to 87)	52 (36 to 71)	75 (52 to 103)
High-income North America	533,547 (376,872 to 722,963)	253,083 (178,292 to 343,085)	280,464 (197,645 to 381,053)	103 (73 to 140)	87 (61 to 118)	123 (86 to 167)
North Africa and Middle East	485,724 (332,330 to 671,890)	214,792 (147,136 to 294,531)	270,932 (184,709 to 378,172)	142 (98 to 195)	123 (85 to 168)	161 (111 to 223)
Oceania	4,475 (3,077 to 6,116)	2,053 (1,411 to 2,792)	2,422 (1,664 to 3,340)	79 (55 to 108)	72 (49 to 97)	87 (60 to 119)
South Asia	1,489,690 (1,007,634 to 2,052,674)	609,190 (414,322 to 828,525)	880,500 (593,027 to 1,216,961)	132 (90 to 181)	110 (75 to 150)	154 (104 to 212)
Southeast Asia	527,905 (356,289 to 729,512)	246,027 (166,897 to 336,237)	281,878 (187,196 to 392,703)	108 (73 to 147)	96 (65 to 130)	120 (81 to 167)
Southern Latin America	28,475 (19,290 to 38,354)	14,194 (9,742 to 18,987)	14,281 (9,693 to 19,338)	39 (27 to 53)	33 (22 to 44)	48 (33 to 65)
Southern Sub-Saharan Africa	40,815 (27,344 to 56,358)	17,393 (11,701 to 23,867)	23,421 (15,618 to 32,727)	88 (59 to 121)	66 (45 to 91)	116 (78 to 160)
Tropical Latin America	273,909 (187,077 to 376,880)	114,628 (78,748 to 156,305)	159,281 (108,358 to 220,704)	145 (99 to 198)	111 (76 to 151)	185 (126 to 254)
Western Europe	542,221 (369,725 to 742,222)	258,514 (176,961 to 350,841)	283,707 (192,859 to 386,405)	72 (48 to 98)	60 (41 to 83)	85 (57 to 116)
Western Sub-Saharan Africa	135,813 (88,567 to 191,105)	44,097 (29,149 to 61,692)	91,716 (60,469 to 130,760)	84 (55 to 117)	55 (37 to 77)	115 (75 to 160)
**Other cardiovascular and circulatory diseases**						
Global	6,070,697 (4,149,988 to 8,291,725)	2,904,069 (1,985,114 to 3,981,270)	3,166,627 (2,142,626 to 4,323,897)	89 (61 to 121)	82 (56 to 112)	95 (65 to 130)
Andean Latin America	144 (131 to 158)	131 (113 to 150)	157 (137 to 178)	73 (49 to 100)	50 (34 to 69)	96 (63 to 131)
Australasia	81 (76 to 86)	71 (65 to 78)	92 (84 to 100)	57 (39 to 78)	56 (38 to 76)	58 (40 to 80)
Caribbean	211 (192 to 235)	208 (179 to 244)	215 (195 to 241)	82 (56 to 113)	63 (43 to 86)	103 (69 to 141)
Central Asia	122 (112 to 134)	78 (69 to 87)	176 (156 to 197)	21 (15 to 30)	15 (10 to 21)	29 (20 to 41)
Central Europe	189 (182 to 195)	131 (126 to 136)	259 (248 to 270)	97 (66 to 133)	78 (53 to 107)	119 (81 to 163)
Central Latin America	115 (111 to 119)	106 (101 to 111)	124 (119 to 130)	100 (68 to 138)	93 (63 to 127)	108 (73 to 147)
Central Sub-Saharan Africa	606 (403 to 884)	654 (392 to 1,037)	549 (347 to 853)	129 (83 to 187)	148 (94 to 222)	107 (71 to 152)
East Asia	99 (92 to 106)	76 (69 to 84)	122 (112 to 133)	115 (78 to 159)	94 (64 to 131)	133 (89 to 184)
Eastern Europe	123 (116 to 131)	90 (84 to 98)	166 (153 to 181)	17 (12 to 24)	17 (11 to 23)	18 (12 to 25)
Eastern Sub-Saharan Africa	809 (656 to 978)	738 (544 to 979)	885 (695 to 1,122)	178 (116 to 262)	225 (147 to 323)	120 (74 to 186)
High-income Asia Pacific	53 (51 to 55)	47 (45 to 49)	60 (57 to 63)	57 (39 to 79)	49 (34 to 68)	65 (45 to 90)
High-income North America	130 (125 to 136)	119 (113 to 126)	142 (135 to 151)	96 (66 to 131)	93 (64 to 127)	101 (70 to 139)
North Africa and Middle East	384 (360 to 409)	366 (333 to 404)	402 (370 to 439)	201 (137 to 273)	180 (123 to 247)	221 (150 to 300)
Oceania	493 (377 to 657)	519 (371 to 719)	468 (366 to 612)	90 (61 to 125)	70 (48 to 96)	111 (75 to 153)
South Asia	39 (36 to 42)	33 (30 to 38)	44 (40 to 50)	4.7 (3.3 to 6.5)	3.8 (2.6 to 5.2)	5.8 (4.0 to 7.9)
Southeast Asia	166 (149 to 185)	142 (123 to 164)	194 (169 to 222)	91 (62 to 125)	85 (58 to 117)	97 (66 to 133)
Southern Latin America	214 (203 to 225)	189 (177 to 203)	242 (225 to 260)	59 (39 to 83)	61 (40 to 88)	57 (37 to 80)
Southern Sub-Saharan Africa	222 (196 to 252)	219 (186 to 257)	226 (194 to 264)	151 (105 to 203)	179 (124 to 240)	103 (71 to 138)
Tropical Latin America	206 (195 to 218)	186 (173 to 201)	229 (213 to 247)	154 (105 to 209)	125 (85 to 170)	186 (126 to 254)
Western Europe	145 (140 to 151)	125 (119 to 131)	167 (159 to 176)	68 (47 to 93)	60 (41 to 82)	76 (53 to 105)
Western Sub-Saharan Africa	353 (297 to 442)	355 (276 to 481)	347 (275 to 459)	339 (230 to 459)	405 (274 to 553)	267 (182 to 363)
**Peripheral artery disease**						
Global	572,829 (272,090 to 1,056,179)	309,381 (146,230 to 572,188)	263,448 (125,664 to 486,823)	9.2 (4.3 to 16.9)	9.0 (4.3 to 16.8)	9.3 (4.4 to 17.1)
Andean Latin America	1,837 (839 to 3,475)	965 (440 to 1,841)	872 (397 to 1,634)	4.6 (2.1 to 8.7)	4.5 (2.0 to 8.6)	4.7 (2.2 to 8.9)
Australasia	4,372 (2,021 to 8,255)	2,044 (945 to 3,896)	2,328 (1,048 to 4,319)	10.9 (5.0 to 20.4)	9.4 (4.3 to 17.8)	12.4 (5.6 to 23.1)
Caribbean	1,829 (840 to 3,472)	964 (436 to 1,803)	865 (397 to 1,627)	4.4 (2.0 to 8.3)	4.2 (1.9 to 7.9)	4.5 (2.1 to 8.5)
Central Asia	5,150 (2,317 to 9,676)	3,017 (1,370 to 5,804)	2,133 (971 to 4,045)	9.5 (4.3 to 18.0)	9.3 (4.3 to 18.1)	9.6 (4.4 to 18.1)
Central Europe	19,814 (9,101 to 37,661)	11,772 (5,449 to 22,702)	8,042 (3,725 to 14,957)	10.4 (4.8 to 19.8)	10.3 (4.8 to 19.9)	10.5 (4.8 to 19.6)
Central Latin America	7,723 (3,633 to 14,270)	4,123 (1,948 to 7,555)	3,601 (1,686 to 6,637)	4.6 (2.2 to 8.5)	4.5 (2.1 to 8.3)	4.7 (2.2 to 8.7)
Central Sub-Saharan Africa	2,831 (1,288 to 5,251)	1,531 (703 to 2,905)	1,300 (591 to 2,426)	8.2 (3.8 to 15.5)	8.1 (3.7 to 15.5)	8.3 (3.8 to 15.2)
East Asia	143,225 (68,744 to 265,548)	75,013 (35,667 to 139,377)	68,212 (32,705 to 124,072)	10.1 (4.8 to 18.6)	10.2 (4.8 to 18.8)	9.9 (4.7 to 18.2)

Eastern Europe	30,873<br>(14,162 to 58,765)	20,516<br>(9,458 to 39,553)	10,357<br>(4,614 to 19,524)	9.4<br>(4.3 to 17.9)	9.3<br>(4.3 to 18.0)	9.4<br>(4.2 to 17.9)
Eastern Sub-Saharan Africa	12,161<br>(5,619 to 22,838)	6,306<br>(2,927 to 12,053)	5,855<br>(2,696 to 10,805)	10.2<br>(4.7 to 19.3)	9.7<br>(4.5 to 18.5)	10.8<br>(4.9 to 20.1)
High-income Asia Pacific	33,829<br>(16,096 to 63,452)	19,506<br>(9,131 to 36,307)	14,324<br>(6,842 to 27,094)	8.6<br>(4.1 to 16.1)	8.6<br>(4.0 to 15.9)	8.5<br>(4.1 to 16.1)
High-income North America	54,324<br>(26,982 to 99,754)	27,726<br>(13,725 to 51,152)	26,598<br>(13,334 to 47,931)	10.6<br>(5.2 to 19.4)	9.6<br>(4.8 to 17.8)	11.6<br>(5.8 to 21.0)
North Africa and Middle East	26,163<br>(11,961 to 48,613)	13,496<br>(6,169 to 26,247)	12,667<br>(5,789 to 23,657)	9.1<br>(4.2 to 17.2)	8.8<br>(4.1 to 17.0)	9.4<br>(4.3 to 17.7)
Oceania	456<br>(208 to 832)	246<br>(114 to 456)	210<br>(97 to 389)	10.3<br>(4.8 to 18.9)	10.2<br>(4.7 to 18.8)	10.4<br>(4.8 to 19.1)
South Asia	75,183<br>(35,185 to 139,639)	38,122<br>(18,003 to 70,799)	37,061<br>(17,355 to 68,903)	7.9<br>(3.7 to 14.6)	7.8<br>(3.7 to 14.3)	8.0<br>(3.8 to 15.0)
Southeast Asia	52,972<br>(24,294 to 97,402)	29,354<br>(13,531 to 54,595)	23,619<br>(10,946 to 43,973)	12.7<br>(5.8 to 23.8)	12.6<br>(5.8 to 23.3)	12.8<br>(5.9 to 23.7)
Southern Latin America	6,881<br>(3,174 to 12,843)	3,852<br>(1,753 to 7,062)	3,029<br>(1,397 to 5,620)	9.6<br>(4.4 to 18.0)	9.0<br>(4.1 to 17.0)	10.3<br>(4.7 to 19.2)
Southern Sub-Saharan Africa	5,127<br>(2,413 to 9,433)	2,987<br>(1,425 to 5,492)	2,139<br>(1,019 to 3,971)	13.1<br>(6.2 to 24.1)	12.7<br>(6.0 to 23.4)	13.7<br>(6.4 to 25.6)
Tropical Latin America	10,468<br>(4,956 to 19,385)	5,880<br>(2,750 to 10,824)	4,588<br>(2,185 to 8,560)	6.1<br>(2.9 to 11.3)	6.0<br>(2.8 to 11.2)	6.2<br>(2.9 to 11.4)
Western Europe	65,721<br>(30,247 to 121,459)	36,044<br>(16,600 to 67,147)	29,678<br>(13,817 to 54,999)	8.2<br>(3.8 to 15.1)	7.9<br>(3.6 to 14.7)	8.5<br>(4.0 to 15.5)
Western Sub-Saharan Africa	11,889<br>(5,426 to 22,427)	5,917<br>(2,693 to 11,221)	5,972<br>(2,733 to 11,116)	10.3<br>(4.7 to 19.5)	9.7<br>(4.5 to 18.5)	11.0<br>(5.0 to 20.6)

eTable 3a. Prevalent Cases of Cardiovascular Causes for Years 1990-2000 with Upper and Lower Uncertainty Bounds

	1990			1995			2000		
	Both	Female	Male	Both	Female	Male	Both	Female	Male
**Global**									
Atrial fibrillation and flutter	18,306,523.6<br>(16,333,861.3 to 20,612,184.1)	8,158,848.4<br>(7,213,772.1 to 9,255,376.1)	10,147,675.4<br>(9,023,327.1 to 11,410,687.0)	20,109,829.6<br>(17,972,993.1 to 22,583,065.9)	8,898,973.5<br>(7,956,377.0 to 10,016,613.5)	11,210,856.9<br>(9,986,521.5 to 12,572,269.6)	22,654,056.7<br>(20,402,018.6 to 25,283,350.5)	9,950,661.8<br>(8,925,936.3 to 11,148,010.3)	12,703,394.5<br>(11,403,246.3 to 14,195,003.6)
Cardiomyopathy and myocarditis	1,486,813.9<br>(1,416,292.0 to 1,558,092.0)	795,463.9<br>(755,198.4 to 834,365.3)	691,350.0<br>(658,317.6 to 726,362.1)	1,626,750.2<br>(1,549,698.4 to 1,699,801.9)	877,982.5<br>(833,908.6 to 919,264.6)	748,767.8<br>(712,037.7 to 785,399.0)	1,796,594.4<br>(1,710,814.3 to 1,877,343.4)	966,009.0<br>(917,771.5 to 1,012,432.4)	830,585.4<br>(790,916.9 to 871,607.6)
Endocarditis	70,896.2<br>(66,466.6 to 76,197.5)	52,522.6<br>(49,213.5 to 56,493.2)	18,373.6<br>(17,033.5 to 19,887.7)	78,837.8<br>(74,186.5 to 84,249.7)	58,263.4<br>(54,875.4 to 62,157.3)	20,574.5<br>(19,139.5 to 22,242.6)	85,700.2<br>(80,543.2 to 91,497.0)	63,116.2<br>(59,365.5 to 67,356.4)	22,584.0<br>(21,011.5 to 24,371.9)
Hemorrhagic and other stroke	11,979,011.4<br>(11,708,997.2 to 12,282,796.8)	6,037,032.7<br>(5,898,188.2 to 6,195,624.8)	5,941,978.7<br>(5,805,758.2 to 6,096,621.0)	13,362,158.6<br>(13,081,655.5 to 13,666,935.1)	6,666,962.2<br>(6,528,639.3 to 6,819,070.4)	6,695,196.9<br>(6,552,193.3 to 6,855,543.6)	14,623,804.5<br>(14,316,630.9 to 14,964,973.3)	7,221,754.4<br>(7,069,739.4 to 7,383,947.7)	7,402,050.0<br>(7,245,062.5 to 7,581,592.2)
Hypertensive heart disease	2,817,450.0<br>(2,646,882.4 to 2,988,502.8)	1,596,626.0<br>(1,497,435.8 to 1,694,434.8)	1,220,824.0<br>(1,148,244.7 to 1,294,319.3)	3,239,274.3<br>(3,048,481.9 to 3,427,595.8)	1,835,747.6<br>(1,727,290.5 to 1,943,328.5)	1,403,526.9<br>(1,319,743.1 to 1,485,505.9)	3,778,742.5<br>(3,552,599.4 to 4,002,184.1)	2,136,191.1<br>(2,006,179.4 to 2,261,441.1)	1,642,551.3<br>(1,547,710.7 to 1,740,299.2)
Ischemic heart disease	63,806,340.4<br>(58,303,508.1 to 70,233,654.3)	27,178,072.6<br>(24,886,003.0 to 29,923,255.1)	36,628,268.2<br>(33,451,192.0 to 40,356,791.8)	70,345,817.9<br>(64,413,003.5 to 77,379,782.7)	29,720,085.8<br>(27,207,227.9 to 32,683,172.8)	40,625,734.7<br>(37,172,291.0 to 44,669,179.0)	78,107,980.6<br>(71,552,691.9 to 85,844,870.2)	32,830,407.6<br>(30,172,608.6 to 35,981,757.3)	45,277,571.7<br>(41,384,645.6 to 49,636,625.7)
Ischemic stroke	15,556,614.7<br>(15,196,647.7 to 15,962,819.1)	7,654,364.2<br>(7,477,839.8 to 7,858,608.9)	7,902,250.6<br>(7,707,554.8 to 8,116,855.9)	17,286,526.7<br>(16,915,820.0 to 17,703,810.6)	8,416,352.0<br>(8,240,062.3 to 8,609,023.5)	8,870,175.3<br>(8,669,600.5 to 9,092,604.5)	18,893,146.5<br>(18,476,815.3 to 19,359,444.3)	9,135,018.1<br>(8,934,001.4 to 9,355,625.7)	9,758,128.2<br>(9,535,725.6 to 10,004,509.7)
Other cardiovascular and circulatory diseases	55,546,972.4<br>(51,620,912.2 to 60,088,660.9)	25,170,966.4<br>(23,326,696.3 to 27,391,445.1)	30,376,006.2<br>(27,521,698.3 to 33,638,001.7)	61,629,586.0<br>(57,249,065.3 to 66,504,063.9)	27,952,563.4<br>(25,979,128.1 to 30,319,895.3)	33,677,025.0<br>(30,511,042.2 to 37,285,024.3)	66,821,099.7<br>(62,397,472.4 to 71,767,804.7)	31,020,920.5<br>(29,012,674.8 to 33,470,177.4)	35,800,178.8<br>(32,707,024.9 to 39,445,852.4)
Peripheral artery disease	79,862,464.7<br>(69,985,759.2 to 91,806,610.4)	43,057,757.5<br>(37,865,954.0 to 49,321,127.7)	36,804,708.1<br>(32,079,495.3 to 42,455,444.5)	89,484,901.3<br>(78,776,520.9 to 102,346,987.8)	48,048,840.4<br>(42,440,852.5 to 54,867,357.4)	41,436,064.0<br>(36,326,642.0 to 47,600,295.5)	100,465,797.4<br>(88,286,961.1 to 114,852,864.0)	53,732,913.0<br>(47,361,126.7 to 61,312,893.3)	46,732,883.1<br>(40,935,949.4 to 53,572,060.0)
**Central Asia**									
Atrial fibrillation and flutter	248,692.7<br>(216,238.7 to 283,683.8)	121,987.6<br>(104,787.0 to 142,678.9)	126,705.1<br>(109,186.7 to 145,435.7)	265,877.2<br>(231,222.7 to 305,010.2)	128,036.7<br>(109,402.2 to 149,147.4)	137,840.5<br>(118,464.5 to 158,154.7)	281,842.5<br>(244,195.1 to 323,112.4)	134,066.9<br>(115,297.6 to 156,376.5)	147,775.7<br>(128,059.5 to 169,623.3)
Cardiomyopathy and myocarditis	7,793.3<br>(7,303.5 to 8,309.2)	3,771.9<br>(3,527.7 to 4,016.9)	4,021.3<br>(3,765.2 to 4,312.5)	8,088.4<br>(7,607.9 to 8,609.5)	3,904.2<br>(3,664.5 to 4,162.6)	4,184.3<br>(3,909.7 to 4,478.9)	8,077.2<br>(7,575.3 to 8,592.5)	3,770.1<br>(3,535.2 to 4,003.9)	4,307.2<br>(4,024.3 to 4,618.4)
Endocarditis	400.9<br>(382.8 to 418.7)	221.6<br>(211.6 to 232.2)	179.2<br>(171.5 to 186.8)	427.5<br>(411.5 to 442.3)	233.9<br>(224.5 to 242.8)	193.6<br>(186.6 to 200.6)	456.2<br>(438.9 to 472.7)	245.6<br>(235.5 to 255.0)	210.6<br>(203.3 to 217.5)
Hemorrhagic and other stroke	205,236.7<br>(200,967.5 to 209,737.7)	117,089.1<br>(114,540.6 to 119,656.2)	88,147.6<br>(86,174.1 to 90,144.5)	216,233.5<br>(212,078.4 to 220,570.9)	121,111.4<br>(118,806.0 to 123,598.4)	95,122.1<br>(93,253.0 to 97,211.7)	228,958.5<br>(224,748.4 to 233,636.9)	126,634.5<br>(124,241.5 to 129,115.1)	102,324.0<br>(100,274.6 to 104,508.5)
Hypertensive heart disease	21,676.3<br>(19,804.4 to 23,548.6)	13,927.3<br>(12,668.6 to 15,131.4)	7,749.1<br>(7,050.7 to 8,487.0)	23,232.7<br>(21,211.4 to 25,442.7)	14,749.3<br>(13,429.8 to 16,132.3)	8,483.4<br>(7,697.3 to 9,323.1)	24,990.2<br>(22,835.3 to 27,492.8)	15,628.2<br>(14,223.1 to 17,209.8)	9,362.0<br>(8,468.6 to 10,334.7)
Ischemic heart disease	1,340,359.4<br>(1,221,879.6 to 1,465,622.5)	691,597.4<br>(626,900.0 to 762,056.6)	648,762.1<br>(590,226.3 to 711,571.4)	1,454,356.3<br>(1,328,809.8 to 1,588,368.5)	727,011.7<br>(660,494.5 to 802,773.2)	727,344.5<br>(665,744.6 to 793,230.7)	1,520,052.1<br>(1,397,294.0 to 1,657,280.7)	749,048.9<br>(683,029.8 to 824,116.4)	771,003.2<br>(708,506.5 to 842,918.8)
Ischemic stroke	253,271.3<br>(247,482.8 to 259,069.2)	146,083.7<br>(142,660.1 to 149,657.4)	107,187.6<br>(104,587.3 to 109,638.9)	266,654.8<br>(260,878.5 to 272,530.9)	148,789.5<br>(145,370.4 to 152,057.1)	117,865.3<br>(115,287.9 to 120,655.6)	277,720.0<br>(271,547.6 to 283,973.5)	151,352.0<br>(147,997.8 to 154,833.2)	126,367.9<br>(123,357.7 to 129,434.5)
Other cardiovascular and circulatory diseases	156,115.5<br>(143,795.9 to 168,230.8)	61,403.9<br>(56,155.7 to 66,705.0)	94,711.5<br>(86,082.1 to 103,504.8)	161,528.9<br>(148,865.3 to 175,537.9)	62,763.4<br>(57,737.7 to 68,198.8)	98,765.5<br>(89,917.1 to 107,866.7)	164,868.9<br>(151,936.5 to 178,942.5)	63,892.1<br>(58,531.6 to 69,247.4)	100,976.8<br>(92,054.9 to 110,338.3)
Peripheral artery disease	1,074,952.6<br>(942,114.5 to 1,239,965.3)	639,140.2<br>(558,725.6 to 734,689.7)	435,812.4<br>(380,240.4 to 505,842.6)	1,146,009.2<br>(1,000,362.5 to 1,328,075.5)	667,702.8<br>(584,062.7 to 767,630.4)	478,306.4<br>(417,632.5 to 556,408.2)	1,190,537.5<br>(1,038,937.2 to 1,376,355.8)	680,939.2<br>(594,343.7 to 787,213.1)	509,598.3<br>(443,671.5 to 590,475.5)
**Eastern Europe**									
Atrial fibrillation and flutter	1,471,580.1<br>(1,282,424.1 to 1,695,006.8)	811,853.4<br>(694,663.7 to 956,244.7)	659,726.8<br>(565,802.5 to 766,206.8)	1,535,300.1<br>(1,330,304.7 to 1,761,313.3)	835,222.5<br>(713,670.7 to 971,565.8)	700,077.6<br>(601,673.6 to 811,391.6)	1,596,380.6<br>(1,383,546.9 to 1,838,215.9)	863,042.3<br>(733,816.0 to 1,006,016.9)	733,338.2<br>(630,579.7 to 852,005.2)
Cardiomyopathy and myocarditis	79,836.5<br>(73,202.9 to 86,624.0)	39,542.0<br>(36,029.0 to 43,094.2)	40,294.6<br>(36,381.1 to 44,646.5)	81,242.4<br>(75,115.2 to 87,663.1)	42,029.5<br>(38,709.5 to 45,484.3)	39,212.9<br>(35,777.2 to 42,970.9)	84,065.5<br>(77,168.3 to 91,085.3)	42,191.8<br>(38,678.4 to 45,876.3)	41,873.7<br>(37,823.6 to 46,159.3)
Endocarditis	1,676.8<br>(1,591.1 to 1,768.0)	1,029.0<br>(968.0 to 1,099.1)	647.8<br>(617.1 to 678.0)	1,739.0<br>(1,659.8 to 1,821.8)	1,046.7<br>(990.9 to 1,106.5)	692.4<br>(662.3 to 721.1)	1,854.6<br>(1,775.4 to 1,934.9)	1,090.4<br>(1,035.3 to 1,146.2)	764.2<br>(729.6 to 795.2)
Hemorrhagic and other stroke	950,867.0<br>(928,818.3 to 973,004.4)	591,616.5<br>(576,846.4 to 606,900.8)	359,250.6<br>(349,630.3 to 368,176.8)	1,009,976.4<br>(987,785.9 to 1,033,288.6)	614,755.9<br>(600,516.9 to 628,882.2)	395,220.5<br>(384,870.5 to 406,120.0)	1,094,936.2<br>(1,068,950.1 to 1,120,045.6)	657,855.9<br>(642,125.6 to 674,135.1)	437,080.3<br>(424,532.8 to 448,322.1)
Hypertensive heart disease	69,112.5<br>(62,040.5 to 76,640.0)	52,180.3<br>(46,661.5 to 58,437.2)	16,932.2<br>(15,102.0 to 19,011.8)	71,603.0<br>(64,654.0 to 79,063.2)	54,968.5<br>(49,140.3 to 61,219.2)	16,634.4<br>(14,883.4 to 18,556.1)	74,372.0<br>(66,567.7 to 82,834.4)	56,648.5<br>(50,316.7 to 63,536.4)	17,723.5<br>(15,734.0 to 20,066.9)
Ischemic heart disease	11,178,685.4<br>(10,297,033.9 to 12,129,848.3)	5,283,843.9<br>(4,806,776.0 to 5,756,784.1)	5,894,842.2<br>(5,385,227.0 to 6,453,150.6)	11,617,668.3<br>(10,722,056.3 to 12,643,313.8)	5,364,062.5<br>(4,920,353.6 to 5,861,553.5)	6,253,605.9<br>(5,702,177.8 to 6,907,014.3)	11,846,932.1<br>(10,949,689.4 to 12,824,514.6)	5,426,336.6<br>(4,962,377.2 to 5,915,045.9)	6,420,595.5<br>(5,885,457.9 to 7,032,918.4)
Ischemic stroke	1,683,081.6<br>(1,636,598.2 to 1,728,797.3)	1,077,721.5<br>(1,046,316.9 to 1,110,790.0)	605,360.2<br>(586,392.0 to 622,524.4)	1,779,207.2<br>(1,734,318.2 to 1,825,361.8)	1,109,360.5<br>(1,080,568.5 to 1,138,435.3)	669,846.7<br>(649,646.2 to 691,531.3)	1,841,014.4<br>(1,792,071.1 to 1,889,758.4)	1,136,643.1<br>(1,105,730.1 to 1,168,616.6)	704,371.3<br>(680,717.3 to 726,662.9)
Other cardiovascular and circulatory diseases	648,604.4<br>(580,199.3 to 725,191.2)	390,714.8<br>(349,126.1 to 436,507.9)	257,889.6<br>(227,302.3 to 294,218.4)	649,061.6<br>(584,397.5 to 718,124.9)	404,823.5<br>(362,464.2 to 447,903.5)	244,238.2<br>(215,211.6 to 276,146.2)	690,778.6<br>(616,354.6 to 772,063.1)	426,220.8<br>(378,526.8 to 477,323.1)	264,557.8<br>(234,275.5 to 299,732.9)
Peripheral artery disease	6,785,364.2<br>(5,926,328.6 to 7,804,919.5)	4,464,032.1<br>(3,915,566.8 to 5,135,047.7)	2,321,332.6<br>(2,009,610.1 to 2,688,843.9)	7,131,137.6<br>(6,220,557.7 to 8,217,312.7)	4,581,912.1<br>(3,985,245.1 to 5,308,927.7)	2,549,225.7<br>(2,220,301.7 to 2,972,750.9)	7,209,914.6<br>(6,275,566.1 to 8,302,520.3)	4,620,713.2<br>(4,028,040.1 to 5,330,003.5)	2,589,201.4<br>(2,249,165.2 to 3,003,750.5)
**Central Europe**									
Atrial fibrillation and flutter	907,786.0<br>(786,249.9 to 1,044,252.7)	448,554.0<br>(383,958.6 to 522,081.6)	459,232.0<br>(396,640.9 to 530,626.5)	1,023,090.1<br>(922,660.8 to 1,134,990.6)	515,299.0<br>(458,716.9 to 577,287.6)	507,791.1<br>(460,061.7 to 559,289.9)	1,096,860.3<br>(1,008,374.6 to 1,198,533.5)	553,864.9<br>(503,154.0 to 613,553.0)	542,995.4<br>(500,916.2 to 589,464.9)
Cardiomyopathy and myocarditis	54,440.7<br>(50,861.2 to 58,137.1)	28,539.7<br>(26,432.5 to 30,704.3)	25,900.9<br>(24,238.1 to 27,737.7)	59,790.4<br>(55,887.5 to 63,771.5)	32,711.9<br>(30,408.3 to 35,014.1)	27,078.5<br>(25,263.3 to 28,912.9)	60,853.1<br>(56,856.4 to 64,853.1)	33,021.5<br>(30,719.8 to 35,356.2)	27,831.6<br>(25,926.5 to 29,677.0)
Endocarditis	1,007.0<br>(957.7 to 1,053.5)	345.0<br>(321.8 to 369.7)	662.0<br>(631.2 to 691.4)	984.7<br>(943.1 to 1,023.4)	348.8<br>(329.3 to 369.6)	635.9<br>(609.8 to 660.8)	990.2<br>(951.6 to 1,029.3)	322.9<br>(304.5 to 341.5)	667.3<br>(639.5 to 694.6)
Hemorrhagic and other stroke	443,976.8<br>(434,514.1 to 454,436.8)	254,422.4<br>(249,343.1 to 260,163.9)	189,554.4<br>(184,872.8 to 194,293.7)	461,813.9<br>(452,833.4 to 471,251.5)	263,073.9<br>(257,897.9 to 268,199.1)	198,740.0<br>(194,389.4 to 203,168.9)	479,799.7<br>(469,838.2 to 490,220.9)	273,750.8<br>(268,027.5 to 279,951.4)	206,048.9<br>(201,374.0 to 210,923.1)
Hypertensive heart disease	79,713.9<br>(72,375.9 to 86,979.9)	51,315.3<br>(46,333.5 to 56,305.7)	28,398.6<br>(25,837.1 to 30,992.9)	89,263.5<br>(81,720.6 to 97,215.2)	59,306.4<br>(54,216.4 to 64,675.8)	29,957.0<br>(27,274.8 to 32,664.3)	95,899.0<br>(87,102.7 to 104,558.4)	64,481.5<br>(58,460.3 to 70,369.9)	31,417.5<br>(28,658.9 to 34,435.4)
Ischemic heart disease	4,275,036.5<br>(3,889,440.8 to 4,712,429.0)	1,784,946.6<br>(1,617,948.6 to 1,977,888.9)	2,490,089.9<br>(2,256,675.3 to 2,746,926.4)	4,234,406.0<br>(3,854,654.5 to 4,699,711.4)	1,784,771.0<br>(1,615,668.3 to 1,979,497.1)	2,449,635.0<br>(2,222,504.7 to 2,705,748.4)	4,386,000.7<br>(3,982,964.0 to 4,836,534.0)	1,843,272.0<br>(1,669,564.6 to 2,041,836.7)	2,542,728.9<br>(2,310,870.8 to 2,804,694.2)
Ischemic stroke	682,661.6<br>(665,883.7 to 700,966.6)	363,313.7<br>(354,882.5 to 372,839.6)	319,347.9<br>(310,162.2 to 328,388.8)	705,088.8<br>(688,338.9 to 722,003.8)	373,285.5<br>(364,715.5 to 382,257.2)	331,803.3<br>(323,491.7 to 341,004.3)	734,487.6<br>(717,239.8 to 753,521.8)	389,848.5<br>(380,289.3 to 400,338.2)	344,639.1<br>(335,738.1 to 354,687.5)
Other cardiovascular and circulatory diseases	1,779,533.8<br>(1,636,200.8 to 1,927,826.9)	803,746.8<br>(741,041.8 to 873,691.8)	975,787.0<br>(898,339.1 to 1,057,416.8)	1,926,637.3<br>(1,775,819.7 to 2,084,456.2)	906,266.6<br>(833,421.1 to 983,716.1)	1,020,370.7<br>(936,953.9 to 1,107,232.8)	2,026,391.9<br>(1,874,255.3 to 2,181,883.7)	963,860.0<br>(891,045.7 to 1,040,279.7)	1,062,532.0<br>(979,760.2 to 1,150,468.4)
Peripheral artery disease	4,049,714.9<br>(3,546,660.7 to 4,651,426.5)	2,306,537.2<br>(2,018,352.9 to 2,638,490.5)	1,743,177.5<br>(1,525,226.3 to 2,011,942.1)	4,302,335.1<br>(3,786,297.9 to 4,928,692.7)	2,459,635.2<br>(2,155,807.4 to 2,816,255.3)	1,842,699.9<br>(1,612,223.8 to 2,120,036.4)	4,581,375.2<br>(4,010,767.9 to 5,275,197.9)	2,625,286.3<br>(2,299,684.9 to 3,034,301.0)	1,956,089.1<br>(1,701,954.6 to 2,254,711.0)
**Western Europe**									
Atrial fibrillation and flutter	3,878,423.3<br>(3,426,903.1 to 4,444,517.5)	1,819,030.0<br>(1,582,339.1 to 2,116,625.0)	2,059,393.3<br>(1,812,610.4 to 2,341,968.3)	4,184,127.7<br>(3,753,821.5 to 4,711,437.8)	1,953,377.4<br>(1,736,000.8 to 2,213,970.5)	2,230,750.4<br>(1,995,969.6 to 2,493,274.4)	4,560,496.2<br>(4,105,740.2 to 5,096,788.7)	2,100,350.2<br>(1,864,444.8 to 2,381,009.4)	2,460,146.1<br>(2,215,567.0 to 2,748,709.6)
Cardiomyopathy and myocarditis	194,648.2<br>(182,393.3 to 207,219.4)	120,493.6<br>(111,599.3 to 129,617.4)	74,154.7<br>(69,674.2 to 79,041.3)	209,047.8<br>(196,742.9 to 221,655.6)	131,427.1<br>(122,246.5 to 140,419.9)	77,620.7<br>(72,874.7 to 82,256.7)	212,681.1<br>(200,810.3 to 224,895.5)	132,915.4<br>(124,240.1 to 141,528.1)	79,765.7<br>(75,003.9 to 84,555.1)
Endocarditis	28,066.3<br>(26,045.9 to 30,385.5)	25,302.9<br>(23,379.4 to 27,521.3)	2,763.4<br>(2,632.0 to 2,905.0)	29,948.6<br>(28,016.8 to 32,074.1)	26,981.9<br>(25,140.3 to 28,986.2)	2,966.7<br>(2,844.4 to 3,098.3)	30,911.4<br>(28,865.7 to 33,210.1)	27,772.2<br>(25,836.4 to 29,933.2)	3,139.2<br>(3,003.1 to 3,281.9)
Hemorrhagic and other stroke	727,407.1<br>(709,737.3 to 748,650.6)	397,979.4<br>(388,662.6 to 409,056.9)	329,427.7<br>(320,755.8 to 339,294.6)	765,286.2<br>(747,303.6 to 785,921.8)	413,470.9<br>(404,222.7 to 424,118.9)	351,815.3<br>(342,579.2 to 362,067.2)	802,219.3<br>(783,267.0 to 822,741.8)	428,860.9<br>(419,498.4 to 439,532.7)	373,358.5<br>(363,971.0 to 383,870.7)
Hypertensive heart disease	194,786.4<br>(178,281.2 to 212,314.7)	126,900.4<br>(115,775.9 to 138,360.7)	67,886.1<br>(62,059.3 to 74,051.0)	219,142.2<br>(202,311.0 to 236,548.0)	144,547.7<br>(132,966.5 to 156,905.7)	74,594.5<br>(69,071.8 to 80,560.1)	227,962.7<br>(210,604.4 to 246,238.8)	150,013.0<br>(138,259.5 to 162,287.9)	77,949.7<br>(71,857.6 to 84,358.4)
Ischemic heart disease	8,648,991.1<br>(7,767,100.1 to 9,764,727.7)	3,704,070.1<br>(3,274,951.3 to 4,256,020.9)	4,944,920.9<br>(4,472,558.3 to 5,483,639.4)	8,723,513.0<br>(7,877,083.3 to 9,789,482.2)	3,669,069.8<br>(3,265,273.1 to 4,178,500.4)	5,054,443.2<br>(4,592,558.9 to 5,630,611.2)	8,547,814.7<br>(7,734,643.8 to 9,552,605.4)	3,592,837.2<br>(3,209,114.1 to 4,075,204.3)	4,954,977.8<br>(4,519,282.5 to 5,479,351.8)
Ischemic stroke	1,415,788.4<br>(1,380,548.6 to 1,458,682.5)	740,054.4<br>(721,534.0 to 762,806.6)	675,734.0<br>(656,601.1 to 697,472.4)	1,499,453.2<br>(1,463,297.7 to 1,542,773.3)	774,023.6<br>(755,575.6 to 795,785.5)	725,429.6<br>(704,924.0 to 748,570.1)	1,550,208.9<br>(1,512,368.8 to 1,594,383.0)	787,897.8<br>(769,114.9 to 809,469.0)	762,311.1<br>(741,768.1 to 785,504.7)
Other cardiovascular and circulatory diseases	6,151,155.1<br>(5,714,129.5 to 6,633,779.9)	3,204,001.4<br>(2,976,069.2 to 3,446,345.5)	2,947,153.7<br>(2,717,840.3 to 3,206,882.2)	6,399,752.0<br>(5,989,992.5 to 6,851,386.0)	3,385,079.0<br>(3,166,192.9 to 3,623,115.0)	3,014,673.1<br>(2,791,348.0 to 3,252,683.0)	6,707,250.3<br>(6,286,522.5 to 7,190,699.2)	3,543,389.6<br>(3,311,928.2 to 3,790,466.3)	3,163,861.0<br>(2,938,073.7 to 3,422,030.4)
Peripheral artery disease	12,902,300.6<br>(11,374,600.5 to 14,712,271.2)	7,423,786.4<br>(6,542,359.9 to 8,424,848.0)	5,478,514.1<br>(4,790,992.6 to 6,341,209.4)	13,622,443.8<br>(12,103,840.4 to 15,422,864.9)	7,793,334.5<br>(6,895,625.1 to 8,832,709.5)	5,829,109.3<br>(5,159,084.9 to 6,627,744.8)	14,378,321.4<br>(12,771,386.5 to 16,265,059.2)	8,151,423.6<br>(7,228,868.8 to 9,240,535.7)	6,226,898.4<br>(5,496,983.5 to 7,070,845.1)
**High-income North America**									
Atrial fibrillation and flutter	3,785,460.9<br>(3,533,832.3 to 4,069,641.4)	1,779,539.7<br>(1,656,244.9 to 1,919,849.9)	2,005,921.2<br>(1,870,393.6 to 2,163,703.3)	3,833,012.2<br>(3,564,244.7 to 4,140,420.4)	1,808,172.2<br>(1,684,995.3 to 1,955,879.2)	2,024,839.9<br>(1,874,406.2 to 2,197,613.2)	4,316,791.5<br>(4,126,610.5 to 4,530,073.9)	2,016,327.7<br>(1,926,966.7 to 2,116,208.5)	2,300,463.8<br>(2,189,192.0 to 2,423,945.7)
Cardiomyopathy and myocarditis	315,715.1<br>(298,816.0 to 333,259.2)	178,836.2<br>(168,576.3 to 189,913.6)	136,878.9<br>(129,502.0 to 144,108.1)	304,370.4<br>(288,113.5 to 321,184.0)	174,671.7<br>(164,286.9 to 185,137.4)	129,698.7<br>(122,932.1 to 136,966.3)	337,824.8<br>(319,687.8 to 357,978.2)	191,499.7<br>(180,119.1 to 203,254.8)	146,325.0<br>(138,488.2 to 154,670.4)
Endocarditis	8,696.9<br>(7,518.4 to 10,029.3)	4,622.5<br>(3,977.2 to 5,359.0)	4,074.3<br>(3,479.6 to 4,749.5)	9,288.3<br>(8,060.9 to 10,705.9)	4,910.3<br>(4,251.5 to 5,718.7)	4,378.0<br>(3,738.7 to 5,092.0)	9,605.4<br>(8,377.8 to 11,025.1)	5,015.6<br>(4,327.6 to 5,816.5)	4,589.8<br>(3,949.8 to 5,279.8)
Hemorrhagic and other stroke	325,087.9<br>(315,879.6 to 335,404.4)	177,175.6<br>(172,179.6 to 182,736.0)	147,912.3<br>(143,541.9 to 152,917.6)	347,480.0<br>(337,307.1 to 358,228.6)	187,033.2<br>(181,713.8 to 192,831.5)	160,446.8<br>(155,504.5 to 165,802.7)	333,014.7<br>(322,083.0 to 344,993.5)	177,941.2<br>(172,062.5 to 184,152.4)	155,073.5<br>(149,933.9 to 160,993.3)
Hypertensive heart disease	333,371.0<br>(312,141.6 to 357,250.3)	207,648.2<br>(194,419.3 to 222,878.0)	125,722.7<br>(117,254.5 to 134,583.9)	321,763.8<br>(301,105.3 to 344,657.6)	200,643.7<br>(187,549.2 to 214,456.2)	121,120.1<br>(112,987.8 to 129,907.6)	357,861.0<br>(335,074.6 to 382,553.8)	221,188.1<br>(206,920.6 to 237,122.2)	136,672.9<br>(127,946.5 to 146,518.3)
Ischemic heart disease	5,631,919.4<br>(5,323,999.1 to 5,965,836.5)	2,459,516.4<br>(2,329,953.4 to 2,604,526.6)	3,172,402.9<br>(2,983,798.8 to 3,373,042.9)	5,988,135.6<br>(5,712,354.7 to 6,269,974.6)	2,582,570.2<br>(2,474,216.6 to 2,696,258.8)	3,405,565.4<br>(3,229,096.9 to 3,580,402.9)	6,157,957.1<br>(5,900,951.4 to 6,425,326.9)	2,692,442.4<br>(2,586,461.8 to 2,802,048.3)	3,465,514.6<br>(3,306,039.8 to 3,628,194.0)
Ischemic stroke	852,704.4<br>(829,406.4 to 878,952.9)	449,062.1<br>(436,484.4 to 462,639.3)	403,642.3<br>(392,420.2 to 416,227.3)	887,092.6<br>(861,502.9 to 915,113.0)	464,367.3<br>(450,726.5 to 478,014.9)	422,725.3<br>(410,200.7 to 437,264.3)	977,068.9<br>(945,910.0 to 1,011,873.2)	508,488.1<br>(491,772.3 to 526,067.4)	468,580.7<br>(453,243.0 to 486,082.4)
Other cardiovascular and circulatory diseases	4,946,180.5<br>(4,667,010.8 to 5,265,840.0)	2,665,355.7<br>(2,514,506.7 to 2,837,603.6)	2,280,824.8<br>(2,135,682.7 to 2,448,068.6)	4,780,374.3<br>(4,502,372.7 to 5,098,164.2)	2,594,433.6<br>(2,448,920.9 to 2,763,167.6)	2,185,940.7<br>(2,045,308.3 to 2,350,497.9)	5,234,057.0<br>(4,929,600.2 to 5,583,101.5)	2,800,802.9<br>(2,636,010.4 to 2,980,190.0)	2,433,254.0<br>(2,280,919.9 to 2,608,178.3)
Peripheral artery disease	5,118,912.3<br>(4,535,267.0 to 5,821,907.8)	2,738,462.4<br>(2,430,682.8 to 3,089,210.8)	2,380,449.9<br>(2,088,005.1 to 2,739,362.5)	5,567,260.8<br>(5,165,209.3 to 6,014,085.8)	2,956,970.9<br>(2,732,884.9 to 3,199,386.7)	2,610,289.9<br>(2,416,277.3 to 2,819,567.3)	5,893,800.3<br>(5,463,831.4 to 6,402,561.2)	3,087,963.5<br>(2,840,757.5 to 3,386,600.5)	2,805,836.7<br>(2,612,689.6 to 3,025,212.3)
**High-income Asia Pacific**									
Atrial fibrillation and flutter	515,626.6<br>(449,314.2 to 591,536.0)	142,234.8<br>(122,923.2 to 166,124.0)	373,391.9<br>(324,791.2 to 426,463.2)	616,638.5<br>(536,533.6 to 704,782.9)	172,703.7<br>(149,391.6 to 201,753.7)	443,934.8<br>(386,701.1 to 507,289.9)	723,441.7<br>(630,627.7 to 827,930.8)	212,701.8<br>(184,127.4 to 248,891.3)	510,739.9<br>(443,741.6 to 584,220.0)
Cardiomyopathy and myocarditis	94,736.6<br>(89,813.0 to 99,974.9)	53,276.7<br>(50,386.0 to 56,364.0)	41,459.9<br>(39,196.5 to 43,832.8)	104,137.0<br>(98,496.7 to 110,004.5)	60,692.1<br>(57,105.2 to 64,478.3)	43,444.8<br>(41,069.7 to 46,039.5)	112,896.7<br>(106,769.3 to 119,450.1)	67,875.9<br>(63,778.3 to 72,204.9)	45,020.8<br>(42,370.1 to 47,705.8)
Endocarditis	3,024.8<br>(2,709.7 to 3,403.9)	1,830.8<br>(1,627.1 to 2,075.5)	1,194.0<br>(1,080.9 to 1,328.8)	3,157.4<br>(2,844.5 to 3,527.4)	1,913.6<br>(1,706.1 to 2,164.6)	1,243.8<br>(1,128.1 to 1,376.2)	3,287.1<br>(2,951.0 to 3,683.9)	2,003.4<br>(1,791.5 to 2,262.3)	1,283.7<br>(1,159.1 to 1,438.8)
Hemorrhagic and other stroke	447,971.3<br>(437,128.2 to 459,907.9)	235,535.8<br>(229,715.3 to 242,268.3)	212,435.5<br>(206,948.2 to 218,154.1)	497,246.3<br>(486,208.5 to 509,383.2)	259,966.4<br>(253,766.2 to 266,715.8)	237,280.0<br>(232,147.7 to 242,868.6)	519,614.8<br>(507,036.4 to 533,496.4)	266,987.3<br>(260,150.1 to 274,542.9)	252,627.5<br>(246,609.1 to 259,174.9)
Hypertensive heart disease	160,841.3<br>(149,753.0 to 172,497.9)	105,549.0<br>(98,061.1 to 113,624.2)	55,292.3<br>(51,593.5 to 59,525.0)	187,567.0<br>(174,284.9 to 202,068.2)	125,059.9<br>(115,920.4 to 134,731.1)	62,507.2<br>(58,375.8 to 66,940.8)	217,262.5<br>(201,963.8 to 234,172.1)	146,388.9<br>(135,788.3 to 157,953.8)	70,873.6<br>(65,892.8 to 76,312.4)
Ischemic heart disease	1,460,831.9<br>(1,378,441.5 to 1,558,580.3)	688,633.6<br>(650,393.0 to 734,242.7)	772,198.3<br>(725,951.5 to 827,340.1)	1,682,303.9<br>(1,587,140.0 to 1,792,600.2)	792,657.1<br>(749,035.0 to 844,792.5)	889,646.8<br>(835,501.3 to 952,080.4)	1,881,552.5<br>(1,778,746.8 to 2,003,198.0)	888,984.7<br>(840,739.7 to 944,346.7)	992,567.8<br>(934,835.4 to 1,060,789.8)
Ischemic stroke	836,553.5<br>(815,643.5 to 858,870.2)	377,555.8<br>(368,153.8 to 387,896.9)	458,997.8<br>(447,069.6 to 471,792.9)	938,486.0<br>(916,490.0 to 962,431.1)	418,958.6<br>(408,099.0 to 429,838.6)	519,527.4<br>(507,056.2 to 533,014.3)	1,014,143.6<br>(988,253.5 to 1,043,641.8)	450,790.9<br>(438,714.9 to 464,808.4)	563,352.7<br>(548,709.9 to 579,251.6)

Other cardiovascular and circulatory diseases	1,910,905.9 (1,789,940.5 to 2,051,049.7)	901,662.8 (844,547.8 to 970,230.0)	1,009,243.2 (940,542.6 to 1,086,906.4)	2,048,465.6 (1,916,993.6 to 2,199,166.1)	980,601.6 (920,216.7 to 1,049,292.9)	1,067,864.0 (994,294.2 to 1,147,203.3)	2,228,603.4 (2,084,138.3 to 2,391,198.4)	1,076,131.7 (1,007,355.2 to 1,152,604.8)	1,152,471.7 (1,072,119.5 to 1,242,943.4)
Peripheral artery disease	3,242,272.5 (2,849,888.2 to 3,716,612.8)	1,830,029.6 (1,616,233.7 to 2,091,285.5)	1,412,243.0 (1,235,903.1 to 1,627,017.1)	3,714,547.0 (3,265,397.4 to 4,246,211.6)	2,132,840.0 (1,882,630.1 to 2,436,451.7)	1,581,707.0 (1,382,588.0 to 1,819,247.2)	4,388,953.5 (3,863,190.8 to 5,024,906.8)	2,545,975.2 (2,246,123.8 to 2,893,857.9)	1,842,978.2 (1,612,135.1 to 2,127,001.1)
**Australasia**									
Atrial fibrillation and flutter	132,923.7 (119,445.4 to 148,238.6)	53,086.8 (48,024.9 to 59,102.9)	79,836.9 (70,838.6 to 89,877.9)	144,480.0 (131,312.7 to 160,084.4)	56,495.9 (51,152.0 to 62,888.5)	87,984.0 (79,273.0 to 97,859.8)	165,959.7 (150,509.4 to 184,115.4)	64,578.9 (58,493.2 to 71,884.6)	101,380.8 (91,447.7 to 113,635.7)
Cardiomyopathy and myocarditis	12,988.6 (12,131.8 to 13,853.9)	6,880.0 (6,346.1 to 7,429.0)	6,108.6 (5,680.5 to 6,573.5)	14,852.1 (13,845.6 to 15,905.9)	8,065.4 (7,415.5 to 8,741.8)	6,786.6 (6,300.4 to 7,293.5)	16,507.6 (15,350.1 to 17,656.8)	9,018.9 (8,267.5 to 9,825.9)	7,488.7 (6,978.5 to 8,071.6)
Endocarditis	490.1 (470.5 to 510.1)	246.4 (235.0 to 259.4)	243.7 (233.9 to 253.1)	511.0 (492.4 to 529.3)	259.0 (247.8 to 270.5)	252.0 (243.3 to 261.1)	514.3 (495.8 to 532.9)	258.9 (247.8 to 270.4)	255.3 (246.0 to 264.2)
Hemorrhagic and other stroke	31,513.7 (30,865.2 to 32,267.4)	17,316.7 (16,914.9 to 17,747.1)	14,197.0 (13,842.2 to 14,597.7)	34,596.7 (33,922.1 to 35,365.4)	19,011.5 (18,610.3 to 19,448.2)	15,585.3 (15,210.2 to 15,972.2)	37,423.1 (36,594.1 to 38,369.2)	20,494.8 (20,005.3 to 21,036.6)	16,928.3 (16,505.1 to 17,411.1)
Hypertensive heart disease	8,811.7 (8,032.1 to 9,676.5)	4,934.7 (4,458.4 to 5,469.1)	3,877.0 (3,503.7 to 4,301.2)	9,642.5 (8,772.1 to 10,567.2)	5,166.0 (4,663.5 to 5,679.8)	4,476.5 (4,043.8 to 4,941.7)	10,990.3 (10,046.7 to 12,073.2)	5,826.3 (5,273.8 to 6,449.3)	5,164.0 (4,670.6 to 5,682.9)
Ischemic heart disease	301,175.4 (282,765.7 to 320,947.3)	116,103.3 (109,126.7 to 123,677.0)	185,072.1 (172,684.8 to 198,725.0)	307,244.2 (291,443.1 to 323,719.9)	120,038.0 (112,731.3 to 127,644.9)	187,206.3 (177,018.2 to 198,079.5)	328,865.7 (311,737.6 to 347,230.5)	127,879.7 (120,431.6 to 136,147.6)	200,986.0 (189,393.1 to 213,119.5)
Ischemic stroke	63,642.4 (62,243.0 to 65,325.2)	32,055.2 (31,243.3 to 32,895.0)	31,587.2 (30,781.3 to 32,518.4)	70,521.4 (69,086.2 to 72,180.1)	35,410.7 (34,628.5 to 36,283.7)	35,110.6 (34,251.2 to 36,050.8)	75,847.6 (74,109.3 to 77,867.1)	37,968.7 (37,015.1 to 39,019.3)	37,879.0 (36,900.3 to 38,963.8)
Other cardiovascular and circulatory diseases	195,325.1 (179,427.0 to 212,627.1)	104,634.4 (96,129.8 to 113,780.4)	90,690.7 (82,667.4 to 99,959.2)	216,403.2 (199,020.9 to 234,961.5)	116,147.0 (106,562.9 to 127,197.3)	100,256.1 (91,380.2 to 110,292.0)	243,720.9 (223,840.7 to 265,590.7)	129,734.1 (118,188.3 to 140,959.2)	113,986.8 (103,356.5 to 125,443.7)
Peripheral artery disease	489,928.0 (428,448.0 to 562,300.5)	239,138.1 (209,500.2 to 273,799.4)	250,790.0 (217,937.5 to 290,833.0)	540,041.7 (477,863.2 to 615,242.7)	269,549.0 (236,248.0 to 308,752.0)	270,492.7 (238,810.1 to 308,625.9)	617,278.0 (547,810.4 to 703,145.1)	305,601.2 (269,117.4 to 348,310.5)	311,676.8 (276,366.9 to 358,051.4)
**Southern Latin America**									
Atrial fibrillation and flutter	209,883.5 (181,915.7 to 241,680.4)	72,222.2 (62,294.9 to 84,200.5)	137,661.3 (119,082.9 to 158,928.1)	235,320.6 (204,840.0 to 271,386.4)	82,114.2 (70,966.6 to 96,548.8)	153,206.3 (133,086.8 to 176,421.4)	263,775.3 (230,200.3 to 303,816.5)	94,784.2 (82,208.5 to 109,996.3)	168,991.1 (147,205.5 to 194,758.6)
Cardiomyopathy and myocarditis	17,022.2 (14,312.9 to 20,166.9)	10,976.3 (9,207.7 to 12,840.8)	6,046.0 (4,982.1 to 7,426.4)	19,929.8 (16,845.9 to 23,467.7)	13,156.2 (11,043.0 to 15,245.1)	6,773.6 (5,567.4 to 8,314.8)	22,021.1 (18,570.1 to 25,848.6)	14,817.2 (12,457.0 to 17,106.2)	7,203.9 (5,944.5 to 8,874.0)
Endocarditis	2,811.6 (2,618.0 to 3,021.8)	2,833.7 (2,647.0 to 3,036.2)	-22.1 (-38.1 to -7.0)	3,315.0 (3,130.2 to 3,534.3)	3,333.3 (3,155.3 to 3,547.6)	-18.4 (-32.3 to -5.2)	3,760.1 (3,546.2 to 4,017.2)	3,758.0 (3,545.4 to 4,009.8)	2.1 (-11.6 to 14.7)
Hemorrhagic and other stroke	91,746.8 (89,459.1 to 94,118.7)	50,222.6 (48,874.4 to 51,510.4)	41,524.2 (40,331.8 to 42,770.9)	98,967.6 (96,643.0 to 101,473.8)	53,745.2 (52,478.5 to 55,007.4)	45,222.5 (43,894.8 to 46,544.2)	104,411.6 (102,065.5 to 107,020.0)	56,750.5 (55,426.2 to 58,108.0)	47,661.1 (46,309.7 to 49,033.6)
Hypertensive heart disease	35,853.3 (30,286.3 to 41,571.1)	22,411.3 (18,923.6 to 25,793.6)	13,442.0 (10,940.2 to 16,249.9)	41,076.3 (35,171.8 to 47,503.6)	26,161.5 (22,633.3 to 29,677.4)	14,914.8 (12,167.0 to 17,860.9)	44,881.1 (38,288.2 to 51,659.3)	28,971.4 (24,914.8 to 32,852.8)	15,909.7 (13,018.4 to 18,958.4)
Ischemic heart disease	327,712.0 (309,413.7 to 347,347.3)	112,763.7 (106,015.9 to 119,575.5)	214,948.3 (201,704.9 to 228,729.5)	358,416.0 (339,950.3 to 378,098.6)	125,452.2 (118,234.6 to 133,119.9)	232,963.8 (220,615.3 to 245,929.7)	393,045.8 (371,133.6 to 416,340.6)	138,858.9 (130,474.0 to 147,324.2)	254,186.9 (238,840.7 to 269,725.3)
Ischemic stroke	142,228.4 (138,314.9 to 146,441.6)	72,743.8 (70,741.5 to 74,773.9)	69,484.6 (67,126.0 to 71,839.7)	157,217.0 (153,281.8 to 161,512.5)	80,266.3 (78,347.4 to 82,370.4)	76,950.7 (74,538.6 to 79,427.1)	166,641.9 (162,518.8 to 170,947.3)	85,397.9 (83,366.5 to 87,595.0)	81,244.0 (78,877.9 to 83,873.7)
Other cardiovascular and circulatory diseases	404,921.8 (305,428.7 to 514,984.5)	243,081.7 (178,029.4 to 307,341.8)	161,840.1 (125,039.2 to 208,778.6)	448,279.9 (346,800.8 to 560,392.0)	275,459.8 (206,718.2 to 340,675.2)	172,820.0 (135,829.1 to 220,063.1)	479,065.8 (369,077.0 to 595,898.3)	298,588.9 (223,976.8 to 370,461.1)	180,476.9 (142,163.2 to 232,024.8)
Peripheral artery disease	798,383.7 (702,196.7 to 918,667.4)	428,450.6 (374,486.7 to 493,481.8)	369,933.1 (322,294.7 to 429,199.2)	889,855.0 (780,278.9 to 1,019,079.4)	489,727.7 (428,862.5 to 563,541.3)	400,127.3 (348,731.3 to 462,660.0)	1,001,167.0 (879,145.4 to 1,146,480.1)	557,428.5 (488,799.8 to 639,667.1)	443,738.6 (385,752.5 to 511,652.1)
**Andean Latin America**									
Atrial fibrillation and flutter	51,216.9 (44,146.9 to 59,314.9)	19,768.6 (16,959.8 to 23,007.6)	31,448.3 (27,212.0 to 36,384.3)	61,048.9 (52,522.1 to 70,896.8)	23,516.2 (20,238.4 to 27,433.8)	37,532.7 (32,408.5 to 43,740.6)	73,026.8 (62,966.3 to 84,466.8)	28,059.9 (24,137.9 to 32,733.4)	44,966.8 (38,497.1 to 52,195.7)
Cardiomyopathy and myocarditis	5,098.5 (4,743.9 to 5,451.5)	2,717.8 (2,536.3 to 2,914.4)	2,380.7 (2,199.8 to 2,559.6)	5,827.0 (5,407.9 to 6,210.6)	3,138.1 (2,920.8 to 3,344.2)	2,688.9 (2,479.1 to 2,881.8)	6,790.2 (6,318.1 to 7,221.6)	3,657.9 (3,415.1 to 3,895.8)	3,132.3 (2,905.2 to 3,359.1)
Endocarditis	83.0 (73.6 to 92.8)	67.4 (62.3 to 72.3)	15.6 (9.6 to 21.7)	84.4 (75.3 to 93.1)	74.5 (69.9 to 79.0)	9.9 (3.3 to 16.1)	97.6 (88.2 to 107.4)	83.9 (79.2 to 88.5)	13.8 (6.9 to 20.7)
Hemorrhagic and other stroke	46,978.6 (45,585.2 to 48,559.7)	25,505.6 (24,681.1 to 26,432.1)	21,473.0 (20,745.3 to 22,181.7)	53,211.4 (51,660.0 to 54,858.3)	28,846.9 (27,921.9 to 29,846.8)	24,364.5 (23,634.8 to 25,143.7)	58,394.1 (56,806.0 to 60,170.7)	31,720.7 (30,751.9 to 32,735.9)	26,673.4 (25,875.4 to 27,531.7)
Hypertensive heart disease	9,948.8 (9,035.4 to 10,833.1)	5,831.1 (5,279.5 to 6,366.9)	4,117.7 (3,727.5 to 4,498.9)	12,074.8 (11,006.8 to 13,159.2)	7,088.6 (6,449.6 to 7,736.7)	4,986.2 (4,524.0 to 5,444.3)	15,306.9 (13,976.6 to 16,605.3)	8,961.7 (8,161.5 to 9,767.3)	6,345.2 (5,763.0 to 6,904.2)
Ischemic heart disease	208,234.1 (188,089.4 to 232,402.6)	90,985.0 (82,372.8 to 101,317.1)	117,249.1 (105,489.1 to 130,822.6)	235,859.2 (212,772.1 to 262,131.2)	102,854.3 (92,629.7 to 114,251.6)	133,004.9 (119,169.7 to 148,454.0)	264,215.0 (238,688.3 to 293,618.9)	114,151.2 (103,211.6 to 126,291.1)	150,063.8 (134,266.4 to 168,153.7)
Ischemic stroke	39,337.6 (38,174.6 to 40,594.4)	20,082.3 (19,417.0 to 20,748.2)	19,255.3 (18,630.5 to 19,857.0)	45,395.5 (44,138.9 to 46,749.9)	23,157.9 (22,471.6 to 23,932.4)	22,237.5 (21,638.6 to 22,879.7)	49,832.6 (48,483.8 to 51,245.9)	25,457.9 (24,711.0 to 26,186.2)	24,374.7 (23,667.3 to 25,105.7)
Other cardiovascular and circulatory diseases	296,065.6 (253,420.1 to 345,119.9)	93,218.2 (78,280.4 to 117,590.4)	202,847.5 (168,431.7 to 243,225.5)	329,523.2 (282,072.3 to 383,866.5)	106,040.5 (89,843.9 to 130,955.3)	223,482.7 (186,655.9 to 266,601.0)	382,810.3 (333,096.7 to 440,123.4)	124,502.4 (106,693.1 to 151,120.8)	258,307.9 (216,880.8 to 304,225.8)
Peripheral artery disease	564,512.2 (495,732.0 to 650,572.1)	284,901.0 (250,025.6 to 326,703.4)	279,611.3 (244,624.8 to 323,945.1)	672,696.3 (588,730.3 to 774,338.4)	339,836.5 (297,475.8 to 392,791.3)	332,859.8 (290,653.9 to 384,559.1)	808,178.9 (709,955.1 to 930,482.0)	406,059.8 (356,617.4 to 468,015.0)	402,119.1 (351,211.6 to 464,932.1)
**Central Latin America**									
Atrial fibrillation and flutter	220,396.3 (190,061.0 to 256,227.9)	87,845.6 (75,202.1 to 102,913.0)	132,550.8 (114,569.1 to 153,196.3)	259,948.9 (224,157.2 to 299,529.7)	104,314.6 (89,418.1 to 120,743.0)	155,634.3 (134,268.7 to 179,113.0)	308,719.0 (266,124.3 to 358,279.3)	123,526.6 (106,215.8 to 143,430.8)	185,192.4 (159,332.9 to 214,681.0)
Cardiomyopathy and myocarditis	40,065.2 (37,868.8 to 42,192.7)	20,946.4 (19,864.2 to 22,003.7)	19,118.8 (17,935.7 to 20,335.5)	46,249.4 (43,611.5 to 48,745.4)	24,414.3 (23,073.8 to 25,633.8)	21,835.1 (20,539.5 to 23,221.3)	52,117.5 (49,170.6 to 54,939.1)	27,507.5 (25,986.8 to 28,878.3)	24,610.0 (23,121.7 to 26,177.1)
Endocarditis	969.1 (885.9 to 1,072.8)	667.3 (606.5 to 742.7)	301.9 (273.7 to 332.5)	1,104.6 (1,008.3 to 1,214.4)	758.0 (691.9 to 840.8)	346.6 (313.5 to 382.1)	1,229.4 (1,121.3 to 1,357.9)	846.3 (770.1 to 938.9)	383.1 (347.9 to 420.7)
Hemorrhagic and other stroke	106,109.4 (102,328.2 to 110,157.8)	57,264.0 (55,253.5 to 59,409.5)	48,845.4 (47,019.7 to 50,813.8)	117,959.8 (113,728.4 to 122,286.4)	63,575.4 (61,292.5 to 65,940.8)	54,384.4 (52,365.8 to 56,415.5)	127,858.4 (123,287.3 to 132,747.5)	68,090.7 (65,613.2 to 70,755.2)	59,767.6 (57,522.6 to 62,115.1)
Hypertensive heart disease	75,612.3 (69,530.4 to 81,659.4)	46,561.8 (42,893.7 to 50,222.4)	29,050.5 (26,446.4 to 31,552.5)	92,177.4 (84,794.0 to 99,561.6)	57,020.7 (52,625.7 to 61,225.5)	35,156.7 (32,216.8 to 38,196.8)	110,391.3 (101,644.9 to 118,827.4)	68,203.2 (62,880.0 to 73,345.4)	42,188.1 (38,704.5 to 45,823.0)
Ischemic heart disease	1,150,768.4 (1,047,851.9 to 1,271,618.7)	558,829.1 (510,134.4 to 616,470.9)	591,939.3 (538,803.8 to 655,111.2)	1,356,038.2 (1,235,656.4 to 1,492,004.6)	658,824.4 (601,418.8 to 724,481.1)	697,213.7 (635,084.7 to 769,003.0)	1,591,691.3 (1,452,920.6 to 1,754,469.4)	760,487.6 (693,622.2 to 839,442.1)	831,203.7 (757,955.8 to 917,944.4)
Ischemic stroke	109,180.5 (106,038.0 to 112,592.8)	56,621.2 (55,045.4 to 58,417.3)	52,559.3 (50,994.1 to 54,254.9)	123,217.4 (119,313.0 to 127,462.3)	63,545.4 (61,596.7 to 65,751.1)	59,672.1 (57,816.9 to 61,739.8)	134,570.2 (130,390.2 to 139,495.3)	68,836.6 (66,750.1 to 71,353.8)	65,733.6 (63,615.1 to 68,115.6)
Other cardiovascular and circulatory diseases	1,481,820.7 (1,349,149.5 to 1,628,901.9)	661,279.3 (600,103.6 to 738,117.0)	820,541.4 (726,166.5 to 921,458.2)	1,716,738.2 (1,565,585.4 to 1,879,574.5)	777,110.3 (705,414.9 to 859,247.7)	939,627.9 (837,849.3 to 1,048,505.4)	1,960,078.2 (1,796,447.9 to 2,141,562.5)	897,615.2 (817,086.6 to 984,944.1)	1,062,463.1 (953,053.6 to 1,179,496.9)
Peripheral artery disease	2,387,148.7 (2,099,727.2 to 2,741,989.7)	1,254,942.3 (1,102,650.0 to 1,443,050.3)	1,132,206.4 (996,016.0 to 1,303,129.6)	2,828,411.1 (2,484,961.6 to 3,252,420.3)	1,491,914.8 (1,314,635.2 to 1,716,687.9)	1,336,496.2 (1,171,495.7 to 1,539,019.9)	3,388,131.3 (2,980,943.3 to 3,888,887.8)	1,774,083.9 (1,560,665.0 to 2,028,744.5)	1,614,047.5 (1,419,216.1 to 1,852,905.2)
**Tropical Latin America**									
Atrial fibrillation and flutter	208,482.3 (179,881.8 to 243,015.6)	86,989.4 (74,993.6 to 102,062.8)	121,492.9 (104,328.7 to 141,597.8)	248,682.8 (214,653.8 to 287,851.7)	104,615.1 (90,326.9 to 121,601.3)	144,067.6 (123,596.8 to 167,920.7)	303,867.4 (262,971.2 to 352,520.5)	127,564.2 (109,464.0 to 148,237.5)	176,303.2 (151,890.5 to 204,941.3)
Cardiomyopathy and myocarditis	95,098.4 (87,799.1 to 101,491.6)	47,064.3 (44,533.7 to 49,461.6)	48,034.1 (43,401.6 to 52,339.8)	121,226.5 (112,961.4 to 128,855.2)	60,010.9 (56,914.4 to 63,073.1)	61,215.5 (55,952.9 to 66,218.7)	141,761.2 (132,388.3 to 150,220.3)	69,478.9 (65,727.8 to 73,133.3)	72,282.3 (66,220.0 to 77,783.2)
Endocarditis	1,069.5 (926.0 to 1,240.8)	490.8 (414.2 to 587.1)	578.7 (506.0 to 658.7)	1,619.9 (1,378.7 to 1,904.9)	738.5 (608.0 to 895.4)	881.4 (759.7 to 1,032.2)	1,847.0 (1,571.0 to 2,163.5)	845.7 (698.0 to 1,023.2)	1,001.3 (863.2 to 1,156.1)
Hemorrhagic and other stroke	207,241.3 (200,662.3 to 214,726.1)	106,833.5 (103,468.4 to 110,690.8)	100,407.7 (96,919.1 to 104,130.8)	221,406.0 (213,497.5 to 230,372.2)	115,214.4 (111,163.1 to 120,205.9)	106,191.6 (102,260.7 to 110,581.8)	228,257.9 (219,232.3 to 238,450.4)	118,819.6 (114,013.8 to 124,010.9)	109,438.3 (105,072.6 to 114,606.7)
Hypertensive heart disease	118,547.4 (109,294.0 to 127,184.8)	68,465.9 (63,602.5 to 73,195.3)	50,081.5 (45,614.0 to 54,178.8)	160,633.1 (149,128.0 to 172,138.1)	93,573.1 (87,244.6 to 99,911.0)	67,060.1 (61,762.8 to 72,418.6)	195,753.7 (181,673.2 to 209,580.4)	112,583.6 (105,382.7 to 120,184.4)	83,170.0 (76,685.1 to 89,553.9)
Ischemic heart disease	1,511,687.1 (1,376,914.7 to 1,668,104.7)	609,545.5 (559,998.4 to 667,137.4)	902,141.5 (815,821.8 to 1,005,549.5)	1,799,261.1 (1,639,470.6 to 1,979,165.1)	743,078.2 (685,477.2 to 809,192.8)	1,056,183.0 (954,804.7 to 1,169,228.7)	2,163,224.9 (1,980,104.8 to 2,370,740.8)	878,331.2 (811,201.2 to 955,437.0)	1,284,893.7 (1,165,072.2 to 1,423,944.0)
Ischemic stroke	247,117.0 (238,920.2 to 256,133.5)	118,503.0 (114,842.2 to 122,522.0)	128,614.0 (123,739.6 to 133,569.4)	265,009.2 (254,837.4 to 276,708.9)	128,871.2 (123,963.5 to 134,526.7)	136,138.0 (130,560.0 to 142,247.9)	271,227.0 (260,042.8 to 284,575.9)	131,827.6 (126,333.8 to 138,228.5)	139,399.4 (133,357.5 to 146,555.6)
Other cardiovascular and circulatory diseases	3,003,101.1 (2,724,319.0 to 3,302,566.4)	1,268,995.2 (1,147,810.6 to 1,439,513.8)	1,734,105.8 (1,523,586.1 to 1,946,339.2)	3,666,223.8 (3,366,712.2 to 3,990,270.1)	1,603,415.9 (1,465,415.6 to 1,776,177.8)	2,062,807.8 (1,834,604.9 to 2,296,702.3)	4,107,938.9 (3,787,093.3 to 4,454,138.5)	1,802,591.3 (1,661,439.1 to 1,977,094.0)	2,305,347.6 (2,061,755.6 to 2,544,673.8)
Peripheral artery disease	3,001,333.5 (2,626,494.0 to 3,469,190.1)	1,608,386.6 (1,410,100.7 to 1,855,735.3)	1,392,946.7 (1,218,782.0 to 1,616,006.4)	3,489,511.8 (3,053,002.4 to 4,022,888.6)	1,884,783.9 (1,649,815.1 to 2,171,849.1)	1,604,727.9 (1,401,824.9 to 1,860,368.8)	4,295,997.8 (3,764,088.0 to 4,947,995.8)	2,324,629.5 (2,037,912.2 to 2,665,235.3)	1,971,368.3 (1,717,187.0 to 2,281,421.8)
**Caribbean**									
Atrial fibrillation and flutter	70,657.8 (60,960.5 to 82,548.7)	27,079.9 (23,274.8 to 31,576.1)	43,577.9 (37,800.3 to 50,570.0)	79,047.3 (68,422.8 to 91,601.6)	30,703.0 (26,394.4 to 35,687.8)	48,344.3 (41,785.4 to 56,170.6)	88,723.5 (76,918.7 to 103,059.5)	34,605.8 (29,978.6 to 40,295.0)	54,117.8 (46,717.6 to 63,069.1)
Cardiomyopathy and myocarditis	13,948.7 (13,214.5 to 14,713.4)	6,978.8 (6,597.2 to 7,357.7)	6,969.9 (6,586.9 to 7,377.8)	15,784.7 (14,932.4 to 16,653.7)	7,979.9 (7,514.5 to 8,408.8)	7,804.8 (7,363.9 to 8,252.4)	17,349.0 (16,393.4 to 18,316.3)	8,858.3 (8,366.5 to 9,353.8)	8,490.8 (8,013.7 to 8,985.6)
Endocarditis	653.7 (620.0 to 694.0)	595.3 (561.7 to 634.8)	58.5 (54.6 to 62.7)	749.1 (713.3 to 788.0)	687.5 (651.8 to 726.0)	61.6 (58.0 to 65.4)	839.6 (795.9 to 887.1)	774.6 (732.8 to 821.2)	65.0 (61.1 to 69.0)
Hemorrhagic and other stroke	77,531.1 (75,510.9 to 79,722.2)	41,360.7 (40,258.0 to 42,562.6)	36,170.4 (35,136.9 to 37,227.3)	82,828.1 (81,024.2 to 85,028.4)	44,041.2 (42,979.5 to 45,262.5)	38,786.8 (37,866.1 to 39,824.6)	90,253.3 (88,033.3 to 92,533.8)	48,147.1 (46,877.6 to 49,434.1)	42,106.2 (41,025.2 to 43,194.6)
Hypertensive heart disease	35,094.0 (32,341.9 to 37,907.6)	17,695.7 (16,289.5 to 19,102.3)	17,398.3 (15,959.3 to 18,823.1)	40,794.9 (37,798.9 to 44,023.4)	20,794.7 (19,193.1 to 22,416.4)	20,000.3 (18,376.2 to 21,691.9)	45,031.7 (41,657.0 to 48,820.5)	23,110.8 (21,294.3 to 24,966.8)	21,920.9 (20,182.9 to 23,778.4)
Ischemic heart disease	363,391.1 (332,958.4 to 399,661.6)	157,722.1 (143,984.5 to 173,143.8)	205,669.0 (187,355.8 to 225,854.6)	395,026.0 (361,538.6 to 433,399.4)	172,872.9 (158,109.4 to 189,562.1)	222,153.2 (203,200.6 to 244,158.4)	446,120.1 (409,841.9 to 489,644.0)	197,191.2 (181,242.0 to 216,144.6)	248,928.9 (228,116.5 to 274,023.1)
Ischemic stroke	75,483.2 (73,638.7 to 77,592.2)	37,208.1 (36,261.9 to 38,234.8)	38,275.1 (37,283.3 to 39,350.9)	80,832.9 (79,076.4 to 82,727.9)	40,036.6 (39,109.7 to 41,029.6)	40,796.2 (39,813.8 to 41,778.8)	87,667.2 (85,557.6 to 89,833.0)	43,540.2 (42,501.9 to 44,635.8)	44,127.1 (42,983.3 to 45,323.3)
Other cardiovascular and circulatory diseases	354,368.9 (325,044.1 to 385,101.0)	131,027.2 (119,409.2 to 145,086.8)	223,341.7 (201,722.0 to 247,006.3)	394,860.0 (363,849.7 to 428,498.9)	148,337.1 (135,844.8 to 163,606.6)	246,522.9 (223,063.8 to 271,667.8)	423,863.9 (391,587.2 to 459,183.6)	162,089.3 (148,455.7 to 177,545.6)	261,774.6 (237,639.3 to 288,468.0)
Peripheral artery disease	721,143.0 (633,994.7 to 828,344.5)	360,067.4 (315,445.2 to 412,220.0)	361,075.6 (317,844.7 to 417,170.6)	801,637.0 (705,294.9 to 920,781.1)	403,830.0 (354,329.2 to 463,436.1)	397,807.0 (348,989.5 to 459,851.2)	908,120.3 (797,378.1 to 1,039,456.5)	459,564.4 (402,775.2 to 525,496.8)	448,555.8 (395,649.3 to 516,444.6)
**Oceania**									
Atrial fibrillation and flutter	9,975.6 (8,577.2 to 11,461.6)	4,029.0 (3,454.8 to 4,679.6)	5,946.6 (5,115.4 to 6,817.9)	11,574.5 (10,034.5 to 13,327.9)	4,731.7 (4,060.5 to 5,495.1)	6,842.8 (5,916.6 to 7,877.5)	13,474.8 (11,628.4 to 15,473.5)	5,558.6 (4,755.7 to 6,478.6)	7,916.1 (6,832.8 to 9,089.1)
Cardiomyopathy and myocarditis	945.7 (890.4 to 1,000.3)	505.4 (475.2 to 534.1)	440.3 (413.3 to 467.3)	1,088.4 (1,028.8 to 1,150.9)	587.1 (553.3 to 620.4)	501.3 (471.0 to 531.6)	1,254.0 (1,183.1 to 1,321.7)	685.3 (645.6 to 724.5)	568.7 (535.6 to 602.1)
Endocarditis	254.0 (243.3 to 266.9)	185.3 (176.8 to 195.0)	68.7 (65.8 to 72.0)	298.7 (286.7 to 311.4)	219.0 (210.1 to 228.4)	79.7 (76.4 to 83.0)	356.4 (343.0 to 370.6)	262.7 (251.7 to 273.7)	93.8 (89.9 to 97.6)
Hemorrhagic and other stroke	18,922.9 (18,436.3 to 19,440.2)	9,489.2 (9,208.2 to 9,783.3)	9,433.7 (9,173.1 to 9,697.6)	21,958.0 (21,444.2 to 22,506.2)	11,075.3 (10,732.3 to 11,422.3)	10,882.8 (10,641.1 to 11,133.5)	25,504.9 (24,880.5 to 26,109.3)	13,013.7 (12,597.7 to 13,417.2)	12,491.1 (12,206.4 to 12,780.0)
Hypertensive heart disease	1,136.6 (1,049.1 to 1,230.6)	648.8 (596.0 to 703.7)	487.8 (448.7 to 529.2)	1,321.3 (1,218.0 to 1,424.4)	761.7 (698.0 to 825.2)	559.6 (513.8 to 608.0)	1,523.8 (1,404.8 to 1,647.6)	883.5 (813.1 to 958.5)	640.3 (587.4 to 693.5)
Ischemic heart disease	24,580.3 (22,143.5 to 27,295.3)	10,679.0 (9,644.5 to 11,801.0)	13,901.3 (12,439.6 to 15,603.3)	29,241.7 (26,440.9 to 32,536.9)	12,755.2 (11,568.2 to 14,124.7)	16,486.6 (14,783.0 to 18,484.5)	35,270.9 (31,809.1 to 39,361.7)	15,302.0 (13,846.5 to 17,017.8)	19,968.8 (17,877.2 to 22,398.3)
Ischemic stroke	15,539.4 (15,105.2 to 15,971.0)	7,194.2 (6,957.7 to 7,442.7)	8,345.1 (8,115.0 to 8,572.5)	18,182.7 (17,727.3 to 18,633.7)	8,550.4 (8,262.1 to 8,829.5)	9,632.4 (9,405.6 to 9,859.7)	20,950.7 (20,415.6 to 21,515.8)	10,022.8 (9,698.9 to 10,385.4)	10,927.9 (10,666.2 to 11,198.7)
Other cardiovascular and circulatory diseases	64,180.7 (55,700.2 to 74,116.8)	20,036.6 (17,505.7 to 23,976.7)	44,144.2 (37,353.4 to 52,147.6)	72,292.3 (63,035.1 to 83,460.2)	23,002.8 (20,121.2 to 27,397.3)	49,289.6 (41,810.2 to 58,256.9)	81,593.5 (71,434.5 to 93,026.4)	26,446.5 (23,318.3 to 31,346.9)	55,147.0 (46,816.4 to 64,301.4)

Peripheral artery disease	42,079.3 (36,541.2 to 48,988.7)	21,385.1 (18,595.7 to 24,849.0)	20,694.2 (17,916.6 to 24,103.1)	49,262.6 (42,974.7 to 57,230.4)	25,371.9 (22,044.9 to 29,416.5)	23,890.7 (20,834.8 to 27,950.1)	58,339.0 (51,015.7 to 67,519.3)	30,219.0 (26,348.7 to 35,001.0)	28,120.0 (24,368.8 to 32,554.1)
**East Asia**									
Atrial fibrillation and flutter	2,958,525.5 (2,555,822.5 to 3,385,992.6)	1,287,908.5 (1,112,837.0 to 1,478,348.2)	1,670,617.0 (1,445,820.0 to 1,917,584.3)	3,389,296.6 (2,937,614.9 to 3,873,978.1)	1,448,362.4 (1,258,747.6 to 1,665,489.8)	1,940,934.0 (1,680,782.3 to 2,217,063.7)	3,936,287.5 (3,416,506.6 to 4,491,704.6)	1,673,914.7 (1,453,600.5 to 1,924,064.9)	2,262,372.8 (1,961,460.7 to 2,590,530.2)
Cardiomyopathy and myocarditis	137,315.5 (131,694.8 to 142,964.9)	69,992.8 (67,216.2 to 72,947.8)	67,322.7 (64,093.9 to 70,475.8)	152,342.8 (145,987.6 to 158,847.8)	77,930.6 (74,581.4 to 81,283.0)	74,412.2 (70,817.1 to 77,982.9)	168,821.9 (161,507.7 to 175,988.6)	88,425.8 (84,419.4 to 92,258.3)	80,396.1 (76,479.6 to 84,176.3)
Endocarditis	3,311.6 (2,989.3 to 3,705.2)	1,267.6 (1,106.5 to 1,457.7)	2,043.9 (1,779.5 to 2,320.8)	3,767.9 (3,380.2 to 4,218.2)	1,461.2 (1,268.8 to 1,684.0)	2,306.7 (2,029.7 to 2,626.5)	4,171.6 (3,750.5 to 4,684.6)	1,649.1 (1,415.5 to 1,910.2)	2,522.5 (2,238.1 to 2,859.0)
Hemorrhagic and other stroke	3,330,860.0 (3,244,025.0 to 3,432,217.2)	1,554,149.1 (1,511,249.4 to 1,602,990.1)	1,776,711.0 (1,732,464.3 to 1,828,537.0)	3,815,115.3 (3,724,888.5 to 3,909,097.9)	1,754,082.4 (1,710,833.1 to 1,797,742.9)	2,061,032.7 (2,014,811.2 to 2,111,540.3)	4,194,745.2 (4,098,203.7 to 4,298,756.7)	1,888,653.9 (1,841,025.4 to 1,941,714.4)	2,306,091.3 (2,253,411.5 to 2,361,149.6)
Hypertensive heart disease	684,942.6 (652,045.3 to 720,173.9)	359,411.6 (342,058.2 to 377,287.4)	325,531.0 (310,252.6 to 342,473.6)	800,855.5 (762,189.9 to 840,808.2)	416,790.0 (396,862.8 to 437,004.4)	384,065.4 (365,853.3 to 403,640.0)	978,563.1 (929,543.2 to 1,028,101.6)	509,256.0 (483,804.8 to 534,913.3)	469,307.1 (445,892.2 to 493,889.5)
Ischemic heart disease	7,931,525.4 (7,175,279.9 to 8,831,787.8)	3,304,896.8 (2,990,229.6 to 3,674,647.6)	4,626,628.6 (4,169,660.3 to 5,160,275.7)	9,325,527.6 (8,426,496.4 to 10,353,565.1)	3,851,965.2 (3,488,515.3 to 4,271,261.5)	5,473,562.0 (4,930,847.7 to 6,073,950.8)	11,121,618.8 (10,039,906.1 to 12,324,366.7)	4,575,161.9 (4,146,449.8 to 5,071,679.8)	6,546,456.7 (5,905,080.2 to 7,271,066.5)
Ischemic stroke	4,208,116.3 (4,096,390.0 to 4,333,109.9)	1,824,920.7 (1,774,999.8 to 1,881,790.8)	2,383,195.5 (2,316,654.1 to 2,453,125.1)	4,791,104.3 (4,674,600.3 to 4,915,354.5)	2,050,144.4 (1,999,789.4 to 2,104,009.7)	2,740,959.6 (2,674,860.8 to 2,814,219.7)	5,304,985.4 (5,171,227.4 to 5,446,824.5)	2,245,437.4 (2,185,853.6 to 2,307,562.5)	3,059,547.9 (2,981,571.9 to 3,142,715.4)
Other cardiovascular and circulatory diseases	13,891,170.4 (12,664,004.9 to 15,555,583.3)	5,213,212.8 (4,656,545.0 to 6,184,918.7)	8,677,957.6 (7,653,864.9 to 9,882,194.6)	15,824,633.1 (14,381,535.9 to 17,718,848.9)	5,824,439.6 (5,226,556.3 to 6,760,931.1)	10,000,192.9 (8,746,403.7 to 11,533,359.5)	16,707,052.9 (15,430,210.1 to 18,400,081.0)	6,530,848.1 (5,988,758.5 to 7,340,228.8)	10,176,204.5 (9,066,057.5 to 11,511,757.2)
Peripheral artery disease	12,825,457.6 (11,176,407.1 to 14,857,131.2)	6,588,138.8 (5,744,166.3 to 7,629,305.6)	6,237,318.7 (5,437,284.7 to 7,225,418.8)	14,749,066.8 (12,896,653.1 to 17,038,071.6)	7,561,900.1 (6,612,402.9 to 8,716,603.7)	7,187,166.0 (6,272,805.1 to 8,329,787.5)	16,872,831.7 (14,750,931.5 to 19,433,421.7)	8,618,817.4 (7,539,204.8 to 9,936,759.1)	8,254,014.2 (7,202,076.2 to 9,552,114.5)
**Southeast Asia**									
Atrial fibrillation and flutter	947,314.3 (822,806.8 to 1,090,899.7)	405,761.5 (349,342.6 to 470,480.3)	541,552.8 (469,755.8 to 621,434.4)	1,104,225.6 (959,996.4 to 1,268,395.8)	474,891.4 (409,856.1 to 548,197.5)	629,334.2 (548,177.7 to 723,646.5)	1,291,821.4 (1,123,538.5 to 1,490,653.5)	559,145.4 (482,469.2 to 649,783.0)	732,676.1 (636,647.3 to 842,347.9)
Cardiomyopathy and myocarditis	85,838.4 (81,050.2 to 90,511.2)	42,971.9 (40,451.6 to 45,375.0)	42,866.6 (40,548.9 to 45,147.3)	98,366.9 (93,069.7 to 103,696.3)	49,572.8 (46,769.0 to 52,336.1)	48,794.1 (46,033.8 to 51,481.6)	111,492.3 (105,321.4 to 117,530.0)	56,724.9 (53,434.8 to 59,861.3)	54,767.4 (51,647.0 to 57,865.1)
Endocarditis	2,848.5 (2,693.2 to 3,006.4)	1,609.4 (1,508.6 to 1,716.7)	1,239.1 (1,176.0 to 1,304.7)	3,405.6 (3,236.7 to 3,573.2)	1,942.9 (1,836.8 to 2,049.6)	1,462.7 (1,392.3 to 1,530.5)	3,950.4 (3,775.6 to 4,139.7)	2,264.8 (2,150.1 to 2,384.8)	1,685.6 (1,605.9 to 1,764.0)
Hemorrhagic and other stroke	1,166,066.0 (1,137,486.3 to 1,195,427.9)	545,210.4 (531,072.1 to 560,067.9)	620,855.6 (605,140.0 to 636,758.6)	1,302,540.3 (1,274,109.6 to 1,331,456.7)	611,925.2 (597,180.6 to 627,268.4)	690,615.1 (673,569.5 to 707,467.9)	1,463,508.7 (1,431,504.6 to 1,498,072.5)	694,475.7 (678,257.9 to 711,844.9)	769,033.1 (751,462.5 to 787,498.7)
Hypertensive heart disease	169,661.3 (156,688.6 to 182,799.6)	102,302.7 (94,424.3 to 110,539.2)	67,358.6 (61,806.7 to 72,437.7)	198,485.8 (183,156.0 to 213,286.0)	120,356.2 (110,523.0 to 129,715.2)	78,129.5 (71,963.0 to 84,014.4)	234,330.9 (216,450.8 to 252,186.4)	143,694.7 (132,395.8 to 155,120.4)	90,636.2 (83,447.9 to 97,979.0)
Ischemic heart disease	3,273,168.9 (2,868,087.1 to 3,798,150.3)	1,442,477.0 (1,273,374.7 to 1,641,935.2)	1,830,692.1 (1,582,049.0 to 2,164,916.9)	3,879,469.9 (3,406,363.2 to 4,471,084.4)	1,717,353.6 (1,530,650.9 to 1,965,652.1)	2,162,116.2 (1,872,507.1 to 2,523,743.1)	4,576,773.9 (4,047,444.1 to 5,230,906.9)	2,021,216.7 (1,805,300.1 to 2,280,033.2)	2,555,557.5 (2,229,094.3 to 2,954,560.5)
Ischemic stroke	1,161,988.3 (1,133,545.7 to 1,190,554.7)	547,416.8 (532,643.0 to 562,936.0)	614,571.6 (598,900.9 to 630,428.4)	1,324,827.5 (1,294,216.9 to 1,355,583.4)	632,117.7 (616,004.5 to 648,482.6)	692,709.8 (676,818.5 to 709,031.6)	1,487,796.2 (1,453,374.9 to 1,525,190.7)	716,905.9 (697,901.9 to 736,543.2)	770,890.4 (752,661.7 to 791,143.0)
Other cardiovascular and circulatory diseases	4,361,844.1 (3,986,677.6 to 4,765,601.1)	1,852,273.6 (1,693,628.4 to 2,054,054.9)	2,509,570.7 (2,229,971.9 to 2,801,380.9)	4,918,694.7 (4,510,182.6 to 5,348,768.8)	2,151,162.3 (1,974,488.7 to 2,360,993.2)	2,767,532.4 (2,476,600.8 to 3,097,581.6)	5,481,431.4 (5,068,409.3 to 5,981,830.0)	2,475,601.6 (2,284,326.1 to 2,697,905.4)	3,005,830.1 (2,702,845.3 to 3,340,898.8)
Peripheral artery disease	5,175,514.1 (4,526,198.0 to 5,992,958.3)	2,722,490.7 (2,375,442.1 to 3,145,781.3)	2,453,023.6 (2,139,938.7 to 2,850,546.8)	6,046,339.5 (5,278,285.7 to 7,019,836.9)	3,208,391.3 (2,813,447.5 to 3,708,150.5)	2,837,948.2 (2,479,693.2 to 3,303,505.8)	7,049,349.3 (6,179,372.8 to 8,117,091.4)	3,742,632.5 (3,276,809.2 to 4,303,017.6)	3,306,717.4 (2,882,213.4 to 3,834,207.7)
**South Asia**									
Atrial fibrillation and flutter	1,814,586.7 (1,570,123.2 to 2,097,487.7)	644,264.2 (555,087.0 to 749,160.7)	1,170,322.6 (1,011,766.4 to 1,350,317.9)	2,094,728.0 (1,814,305.9 to 2,416,198.1)	752,466.2 (648,608.1 to 875,092.4)	1,342,261.9 (1,159,718.2 to 1,544,765.1)	2,453,665.9 (2,122,832.5 to 2,837,696.4)	892,418.4 (769,701.6 to 1,042,238.2)	1,561,247.5 (1,344,508.2 to 1,802,622.0)
Cardiomyopathy and myocarditis	81,119.0 (76,694.7 to 85,781.2)	43,266.2 (40,933.4 to 45,758.2)	37,852.7 (35,618.6 to 40,344.2)	92,942.9 (87,951.5 to 97,992.4)	49,412.6 (46,765.2 to 52,229.1)	43,530.3 (40,948.2 to 46,130.8)	106,151.2 (100,494.2 to 111,879.1)	56,326.9 (53,413.5 to 59,290.7)	49,824.3 (46,919.9 to 52,821.3)
Endocarditis	4,918.7 (4,333.6 to 5,612.7)	2,655.4 (2,307.4 to 3,076.6)	2,263.4 (1,993.6 to 2,564.3)	5,973.6 (5,257.4 to 6,827.0)	3,240.4 (2,825.7 to 3,740.8)	2,733.1 (2,415.3 to 3,096.2)	7,159.5 (6,376.3 to 8,143.6)	3,897.6 (3,407.1 to 4,483.8)	3,261.9 (2,912.3 to 3,672.5)
Hemorrhagic and other stroke	2,311,165.5 (2,246,928.6 to 2,381,422.1)	1,059,344.0 (1,028,430.0 to 1,091,922.2)	1,251,821.5 (1,217,092.0 to 1,290,322.0)	2,587,939.3 (2,527,866.5 to 2,654,530.9)	1,184,090.8 (1,154,755.9 to 1,216,020.2)	1,403,848.6 (1,372,053.8 to 1,439,751.8)	2,852,771.6 (2,782,518.5 to 2,928,161.2)	1,289,037.0 (1,255,550.5 to 1,325,165.2)	1,563,734.5 (1,526,439.9 to 1,604,741.6)
Hypertensive heart disease	459,681.9 (435,313.2 to 484,451.9)	230,034.8 (218,181.8 to 242,901.3)	229,647.1 (217,095.6 to 242,604.1)	553,473.8 (524,042.2 to 583,939.3)	278,811.7 (264,134.5 to 294,542.5)	274,662.1 (259,287.1 to 289,544.0)	669,755.9 (634,759.8 to 704,889.2)	340,419.8 (321,994.0 to 358,707.4)	329,336.1 (311,325.5 to 347,176.7)
Ischemic heart disease	10,233,045.7 (9,237,815.4 to 11,399,196.3)	3,782,731.5 (3,409,642.9 to 4,214,592.4)	6,450,314.7 (5,813,545.9 to 7,201,003.3)	11,859,249.1 (10,681,611.0 to 13,190,944.7)	4,452,812.3 (4,008,702.5 to 4,955,654.4)	7,406,437.3 (6,669,861.3 to 8,245,652.6)	14,402,629.3 (13,043,776.2 to 15,993,864.6)	5,411,864.3 (4,888,344.8 to 6,016,319.6)	8,990,764.5 (8,122,927.5 to 10,026,794.0)
Ischemic stroke	2,259,158.9 (2,199,406.1 to 2,323,842.3)	994,819.3 (965,352.7 to 1,023,559.0)	1,264,339.7 (1,229,807.8 to 1,301,099.1)	2,568,236.1 (2,505,541.4 to 2,633,633.7)	1,142,652.2 (1,114,406.6 to 1,172,317.1)	1,425,584.0 (1,391,790.2 to 1,462,446.2)	2,874,071.1 (2,802,647.6 to 2,949,197.9)	1,280,773.6 (1,244,610.8 to 1,316,952.3)	1,593,297.4 (1,555,117.9 to 1,631,434.1)
Other cardiovascular and circulatory diseases	307,528.2 (286,876.5 to 328,074.7)	121,362.1 (113,054.5 to 129,392.8)	186,166.1 (173,091.4 to 200,052.5)	368,749.0 (343,919.3 to 393,744.4)	147,167.7 (137,189.7 to 157,318.5)	221,581.3 (206,315.1 to 237,305.7)	446,032.5 (416,534.9 to 475,978.7)	180,567.1 (168,696.6 to 192,221.2)	265,465.4 (247,135.3 to 283,912.5)
Peripheral artery disease	11,397,835.5 (9,954,722.2 to 13,226,939.4)	5,455,369.4 (4,765,227.2 to 6,329,337.7)	5,942,466.5 (5,195,220.0 to 6,897,937.4)	13,127,259.4 (11,496,169.6 to 15,176,286.7)	6,347,845.4 (5,553,364.6 to 7,338,171.7)	6,779,414.5 (5,919,768.3 to 7,853,189.5)	15,355,613.8 (13,412,459.5 to 17,765,011.7)	7,521,303.9 (6,579,051.8 to 8,708,560.3)	7,834,309.3 (6,831,876.9 to 9,086,615.7)
**North Africa and Middle East**									
Atrial fibrillation and flutter	425,325.0 (365,445.1 to 493,759.4)	171,313.7 (147,236.5 to 200,264.1)	254,011.3 (218,304.6 to 295,358.9)	505,860.6 (435,466.8 to 586,429.7)	202,473.4 (174,196.2 to 236,569.4)	303,387.2 (260,877.8 to 350,798.8)	592,818.1 (510,486.9 to 684,716.3)	237,293.4 (203,318.4 to 276,573.7)	355,524.7 (306,386.2 to 410,141.1)
Cardiomyopathy and myocarditis	78,766.7 (74,647.7 to 82,763.5)	38,754.5 (36,676.5 to 40,910.6)	40,012.3 (37,861.9 to 42,188.0)	92,038.2 (87,196.0 to 96,924.1)	45,120.0 (42,731.7 to 47,576.6)	46,918.1 (44,443.3 to 49,446.8)	105,930.7 (100,281.0 to 111,655.4)	52,086.4 (49,260.9 to 54,869.3)	53,844.3 (50,977.0 to 56,862.8)
Endocarditis	7,638.2 (7,314.9 to 7,974.3)	6,892.2 (6,591.9 to 7,214.0)	746.0 (697.4 to 796.8)	9,061.8 (8,736.8 to 9,375.1)	8,209.6 (7,914.7 to 8,505.6)	852.1 (802.2 to 906.6)	10,826.4 (10,451.7 to 11,211.0)	9,873.0 (9,511.3 to 10,233.6)	953.4 (900.1 to 1,010.4)
Hemorrhagic and other stroke	542,801.1 (529,075.7 to 557,228.4)	295,137.5 (287,536.8 to 303,086.9)	247,663.6 (241,322.4 to 254,418.8)	643,213.8 (628,037.3 to 659,561.2)	347,974.5 (339,573.4 to 356,568.7)	295,239.3 (288,053.2 to 302,473.9)	736,250.2 (719,995.5 to 753,118.8)	400,317.9 (391,230.7 to 409,522.5)	335,932.3 (328,054.1 to 344,149.2)
Hypertensive heart disease	94,996.3 (87,505.6 to 102,620.3)	63,469.7 (58,279.1 to 68,572.2)	31,526.6 (28,994.5 to 34,096.8)	111,452.3 (102,771.6 to 120,490.5)	74,294.6 (68,528.1 to 80,288.2)	37,157.7 (34,080.1 to 40,312.3)	128,660.2 (118,778.5 to 139,142.8)	86,041.5 (79,368.8 to 92,913.0)	42,618.7 (39,061.9 to 46,205.4)
Ischemic heart disease	2,799,720.4 (2,502,027.6 to 3,164,738.2)	1,175,033.6 (1,045,764.2 to 1,333,567.1)	1,624,687.0 (1,450,086.2 to 1,836,403.6)	3,435,710.7 (3,065,195.4 to 3,869,702.5)	1,435,920.6 (1,272,206.2 to 1,629,054.3)	1,999,789.9 (1,788,796.3 to 2,254,693.3)	4,199,813.4 (3,741,354.7 to 4,725,343.9)	1,763,932.1 (1,565,984.4 to 1,993,771.1)	2,435,881.2 (2,171,907.9 to 2,734,871.2)
Ischemic stroke	610,807.1 (595,567.2 to 626,057.4)	311,441.6 (303,180.8 to 319,550.6)	299,365.5 (292,069.6 to 307,152.5)	731,788.6 (715,200.2 to 748,474.4)	372,062.6 (363,464.0 to 381,244.0)	359,726.0 (351,122.9 to 367,949.0)	850,826.3 (832,020.8 to 868,990.0)	434,795.4 (424,878.7 to 444,043.1)	416,030.8 (406,480.0 to 425,570.6)
Other cardiovascular and circulatory diseases	8,702,502.1 (7,782,695.9 to 9,772,279.8)	3,247,102.5 (2,884,316.9 to 3,812,200.0)	5,455,400.1 (4,741,106.8 to 6,274,070.1)	9,767,526.3 (8,759,561.6 to 10,936,247.9)	3,698,238.9 (3,312,893.5 to 4,262,881.3)	6,069,287.1 (5,294,947.8 to 6,924,299.0)	10,384,230.1 (9,421,580.5 to 11,513,549.8)	4,093,721.1 (3,701,782.5 to 4,625,205.2)	6,290,508.9 (5,535,006.8 to 7,138,517.7)
Peripheral artery disease	3,868,005.6 (3,380,736.3 to 4,483,610.1)	1,926,405.6 (1,685,814.9 to 2,236,493.2)	1,941,600.3 (1,691,206.7 to 2,247,337.7)	4,547,888.8 (3,976,482.2 to 5,223,882.4)	2,251,995.1 (1,974,694.6 to 2,587,815.0)	2,295,893.5 (2,005,874.7 to 2,648,139.5)	5,313,915.1 (4,631,009.8 to 6,127,156.6)	2,639,239.7 (2,309,714.7 to 3,032,411.8)	2,674,675.3 (2,333,100.2 to 3,094,138.5)
**Central Sub-Saharan Africa**									
Atrial fibrillation and flutter	47,578.1 (40,805.8 to 55,675.5)	19,471.8 (16,454.4 to 23,012.0)	28,106.3 (24,080.3 to 32,666.4)	56,152.3 (48,140.6 to 65,601.3)	22,779.8 (19,265.6 to 26,701.3)	33,372.5 (28,645.1 to 39,118.4)	63,287.3 (54,535.1 to 73,766.9)	25,503.1 (21,766.0 to 29,821.4)	37,784.3 (32,336.7 to 44,055.5)
Cardiomyopathy and myocarditis	18,645.8 (17,405.4 to 19,984.3)	9,206.7 (8,555.8 to 9,895.5)	9,439.1 (8,802.2 to 10,151.8)	21,757.5 (20,264.0 to 23,365.2)	10,635.8 (9,840.8 to 11,441.8)	11,121.8 (10,338.7 to 11,992.4)	24,555.4 (22,882.1 to 26,258.1)	11,944.8 (11,077.9 to 12,815.1)	12,610.6 (11,723.9 to 13,534.6)
Endocarditis	295.9 (279.9 to 311.3)	173.8 (164.2 to 183.6)	122.1 (115.4 to 128.9)	344.1 (326.5 to 362.1)	201.5 (190.6 to 212.8)	142.6 (134.6 to 150.4)	385.5 (366.1 to 404.5)	225.5 (213.7 to 237.1)	160.0 (151.5 to 168.3)
Hemorrhagic and other stroke	89,115.2 (87,063.9 to 91,278.8)	52,733.1 (51,438.6 to 54,072.3)	36,382.1 (35,480.9 to 37,311.4)	102,790.2 (100,663.2 to 105,058.7)	60,447.3 (59,093.1 to 61,846.9)	42,342.9 (41,394.1 to 43,371.3)	114,436.9 (111,957.1 to 117,015.8)	66,901.7 (65,406.6 to 68,420.0)	47,535.2 (46,400.1 to 48,744.6)
Hypertensive heart disease	32,243.4 (29,619.4 to 35,022.5)	15,410.0 (14,072.0 to 16,845.4)	16,833.4 (15,435.9 to 18,204.8)	38,010.9 (34,943.9 to 41,204.9)	18,056.5 (16,329.3 to 19,822.0)	19,954.4 (18,334.9 to 21,637.0)	42,682.8 (39,267.8 to 46,347.0)	20,174.0 (18,367.8 to 22,187.1)	22,508.8 (20,740.9 to 24,356.4)
Ischemic heart disease	131,649.1 (120,657.3 to 144,397.5)	60,633.1 (55,445.4 to 66,360.2)	71,015.9 (64,857.7 to 78,054.9)	149,385.5 (137,324.1 to 163,389.1)	68,516.8 (62,938.0 to 75,024.8)	80,868.8 (74,208.1 to 88,924.4)	167,531.1 (154,665.5 to 182,287.4)	75,916.0 (70,114.3 to 82,682.0)	91,615.1 (84,115.1 to 99,970.6)
Ischemic stroke	95,512.1 (92,951.1 to 98,289.5)	55,886.0 (54,279.4 to 57,560.9)	39,626.1 (38,470.3 to 40,742.1)	110,405.6 (107,646.3 to 113,235.5)	64,181.4 (62,420.9 to 65,921.5)	46,224.2 (45,000.2 to 47,446.9)	121,037.8 (117,883.3 to 124,308.1)	69,967.0 (68,016.9 to 71,968.8)	51,070.8 (49,699.7 to 52,457.8)
Other cardiovascular and circulatory diseases	461,653.2 (369,681.2 to 573,673.9)	281,132.7 (216,334.5 to 359,861.0)	180,520.5 (148,828.6 to 218,636.8)	540,985.5 (429,541.7 to 674,876.8)	327,395.7 (250,220.1 to 425,062.6)	213,589.8 (176,128.7 to 257,214.4)	588,263.8 (466,778.4 to 734,104.9)	345,258.9 (263,921.5 to 451,375.1)	243,004.9 (199,287.6 to 294,529.8)
Peripheral artery disease	478,403.4 (417,820.2 to 555,342.7)	260,575.6 (227,745.6 to 302,685.5)	217,827.8 (189,225.8 to 252,307.5)	552,671.0 (481,370.3 to 641,248.6)	298,905.6 (260,375.2 to 347,554.5)	253,765.4 (221,535.6 to 295,927.5)	614,848.7 (534,733.7 to 717,560.6)	328,709.6 (286,488.5 to 382,624.6)	286,139.2 (248,149.3 to 332,558.2)
**Eastern Sub-Saharan Africa**									
Atrial fibrillation and flutter	157,635.6 (135,671.0 to 183,962.2)	59,688.5 (50,718.5 to 70,203.7)	97,947.0 (84,351.1 to 114,463.8)	180,274.5 (155,445.4 to 210,209.3)	67,884.9 (57,743.7 to 79,218.6)	112,389.6 (96,544.1 to 131,110.4)	207,460.9 (178,788.5 to 241,102.7)	79,286.0 (67,474.6 to 92,605.8)	128,174.9 (111,099.8 to 148,729.7)
Cardiomyopathy and myocarditis	46,256.2 (41,063.4 to 52,315.3)	21,941.0 (19,423.9 to 24,894.8)	24,315.1 (21,587.6 to 27,440.1)	52,928.6 (47,224.9 to 59,443.9)	25,020.8 (22,237.2 to 28,269.9)	27,907.8 (24,918.5 to 31,401.8)	60,926.6 (54,408.2 to 68,374.5)	28,774.5 (25,468.0 to 32,443.0)	32,152.1 (28,762.1 to 36,124.5)
Endocarditis	1,074.4 (1,011.0 to 1,134.8)	598.6 (563.3 to 634.2)	475.8 (447.1 to 504.2)	1,214.7 (1,146.3 to 1,280.5)	680.0 (642.0 to 717.6)	534.8 (504.7 to 565.4)	1,380.8 (1,307.3 to 1,456.4)	775.3 (733.2 to 817.3)	605.5 (571.3 to 639.6)
Hemorrhagic and other stroke	355,197.9 (345,954.7 to 364,807.9)	194,888.7 (190,023.6 to 200,076.3)	160,309.2 (155,863.0 to 165,157.0)	410,593.9 (400,496.1 to 421,740.1)	225,914.6 (220,458.8 to 231,916.8)	184,679.3 (179,783.7 to 190,167.6)	488,414.1 (475,381.6 to 501,932.2)	269,513.2 (262,531.5 to 276,648.4)	218,900.9 (212,477.9 to 225,589.7)
Hypertensive heart disease	88,907.1 (80,833.1 to 97,311.9)	39,382.0 (35,275.8 to 43,555.1)	49,525.1 (45,287.8 to 54,090.7)	99,157.2 (90,008.9 to 108,411.4)	43,581.3 (39,117.2 to 48,155.9)	55,576.0 (50,635.4 to 60,455.7)	112,760.5 (102,704.3 to 123,233.0)	50,027.7 (45,041.7 to 55,227.8)	62,732.8 (57,284.7 to 68,267.1)
Ischemic heart disease	1,479,461.7 (1,292,881.4 to 1,705,979.4)	592,069.6 (526,960.3 to 671,681.7)	887,392.1 (763,504.3 to 1,038,186.0)	1,711,542.9 (1,500,414.1 to 1,958,629.6)	686,008.7 (613,063.1 to 776,776.1)	1,025,534.1 (884,303.7 to 1,191,033.3)	1,997,111.3 (1,763,905.4 to 2,276,084.4)	807,686.0 (725,392.6 to 905,092.8)	1,189,425.2 (1,025,388.0 to 1,384,039.8)
Ischemic stroke	341,375.5 (332,640.8 to 351,092.6)	180,305.4 (175,655.4 to 185,399.1)	161,070.0 (156,768.6 to 165,990.9)	398,012.0 (388,757.7 to 407,962.6)	211,435.1 (206,511.5 to 216,956.2)	186,576.9 (182,023.9 to 191,402.4)	466,121.5 (454,947.8 to 478,299.7)	251,248.0 (245,071.7 to 257,756.0)	214,873.5 (209,058.0 to 220,681.4)
Other cardiovascular and circulatory diseases	1,846,509.5 (1,433,762.2 to 2,485,287.0)	1,111,684.2 (848,781.5 to 1,449,242.0)	734,825.2 (535,989.6 to 1,061,495.1)	2,070,688.7 (1,587,940.3 to 2,810,810.1)	1,234,913.8 (934,182.3 to 1,637,441.7)	835,774.9 (611,174.9 to 1,207,187.1)	2,383,065.6 (1,860,753.4 to 3,177,277.4)	1,423,875.7 (1,103,975.2 to 1,846,646.8)	959,189.9 (706,029.2 to 1,381,358.0)
Peripheral artery disease	1,848,043.2 (1,612,321.0 to 2,146,105.3)	925,032.4 (808,028.5 to 1,068,994.6)	923,010.9 (803,320.9 to 1,076,058.2)	2,135,989.6 (1,863,769.3 to 2,475,549.6)	1,061,823.9 (924,373.8 to 1,234,140.8)	1,074,165.5 (938,682.5 to 1,244,614.3)	2,489,892.2 (2,169,091.8 to 2,895,099.1)	1,244,242.8 (1,085,520.9 to 1,445,568.2)	1,245,649.4 (1,085,146.3 to 1,449,493.5)
**Southern Sub-Saharan Africa**									
Atrial fibrillation and flutter	63,316.0 (54,766.7 to 73,355.8)	26,907.1 (22,892.3 to 31,350.2)	36,408.9 (31,468.2 to 42,192.9)	73,953.3 (64,153.7 to 85,799.5)	31,843.2 (27,325.6 to 37,087.1)	42,110.1 (36,476.4 to 48,842.7)	81,395.2 (70,368.5 to 94,275.1)	35,758.1 (30,765.5 to 41,891.1)	45,637.1 (39,415.4 to 52,916.9)
Cardiomyopathy and myocarditis	36,294.8 (34,760.1 to 37,934.5)	18,110.9 (17,320.2 to 18,927.5)	18,183.9 (17,391.4 to 18,990.6)	42,856.9 (40,998.3 to 44,731.9)	21,599.2 (20,674.7 to 22,608.5)	21,257.7 (20,292.8 to 22,196.6)	47,132.3 (45,155.8 to 49,205.0)	24,002.0 (22,945.2 to 25,144.5)	23,130.3 (22,158.7 to 24,227.4)
Endocarditis	263.8 (238.0 to 294.1)	141.1 (126.9 to 157.3)	122.7 (107.5 to 141.7)	304.2 (274.3 to 337.8)	163.1 (146.6 to 181.3)	141.1 (124.5 to 162.0)	332.2 (301.4 to 369.5)	179.3 (161.4 to 199.7)	152.9 (134.7 to 175.7)
Hemorrhagic and other stroke	118,833.5 (115,742.9 to 122,230.1)	59,456.9 (57,803.5 to 61,242.9)	59,376.6 (57,900.1 to 61,002.8)	136,828.0 (133,590.7 to 140,375.4)	69,151.5 (67,363.4 to 71,136.0)	67,676.6 (66,145.5 to 69,365.9)	151,766.0 (148,276.2 to 155,711.6)	78,056.8 (76,162.6 to 80,218.0)	73,709.3 (71,959.1 to 75,628.6)
Hypertensive heart disease	30,308.5 (28,410.4 to 32,292.6)	14,568.2 (13,589.9 to 15,571.6)	15,740.3 (14,679.5 to 16,875.3)	35,529.9 (33,253.2 to 37,887.2)	17,581.5 (16,434.8 to 18,773.9)	17,948.4 (16,731.7 to 19,196.7)	38,638.6 (36,064.9 to 41,097.0)	19,392.1 (18,126.1 to 20,668.2)	19,246.6 (18,003.3 to 20,540.0)
Ischemic heart disease	401,983.1 (359,549.7 to 452,194.8)	168,004.3 (151,418.0 to 188,665.4)	233,978.8 (208,384.5 to 266,322.5)	469,148.4 (420,701.9 to 527,660.0)	200,404.2 (180,314.4 to 224,515.8)	268,744.2 (239,468.1 to 304,080.1)	517,401.8 (462,592.6 to 578,635.9)	223,116.5 (201,734.4 to 248,930.4)	294,285.3 (262,208.5 to 333,279.7)
Ischemic stroke	125,405.8 (122,492.8 to 128,801.6)	65,839.4 (64,026.4 to 67,979.7)	59,566.4 (58,220.2 to 61,122.2)	142,785.2 (139,663.0 to 146,435.0)	75,629.4 (73,735.3 to 77,901.8)	67,155.8 (65,819.3 to 68,709.6)	155,924.8 (152,532.3 to 159,716.9)	84,445.2 (82,355.8 to 86,826.8)	71,479.6 (69,894.7 to 73,167.7)
Other cardiovascular and circulatory diseases	482,664.4 (459,409.9 to 506,225.4)	320,285.2 (303,011.6 to 337,161.0)	162,379.2 (151,540.7 to 174,912.3)	573,570.9 (544,480.1 to 601,068.0)	374,656.5 (354,039.1 to 393,469.1)	198,914.4 (185,502.4 to 213,005.7)	643,030.9 (612,223.9 to 672,856.5)	426,225.5 (404,805.8 to 447,892.2)	216,805.4 (201,991.6 to 231,715.2)
Peripheral artery disease	974,635.8 (855,902.6 to 1,119,190.1)	532,673.3 (466,246.8 to 609,498.9)	441,962.5 (386,917.2 to 511,727.8)	1,130,885.8 (990,740.8 to 1,297,751.5)	624,263.2 (548,163.4 to 716,502.7)	506,622.6 (443,164.6 to 583,625.6)	1,276,830.9 (1,121,136.3 to 1,465,011.8)	713,669.5 (627,953.4 to 817,843.0)	563,161.3 (493,348.8 to 648,027.4)

**Western Sub-Saharan Africa**									
Atrial fibrillation and flutter	181,137.1<br>(155,906.4 to 211,923.6)	69,312.2<br>(58,827.4 to 81,211.1)	111,824.9<br>(96,176.4 to 130,872.4)	207,191.0<br>(178,557.4 to 241,723.0)	78,970.1<br>(67,493.9 to 92,853.3)	128,220.9<br>(110,252.6 to 148,866.5)	233,960.5<br>(200,623.0 to 272,475.4)	88,310.6<br>(75,086.9 to 103,556.1)	145,650.0<br>(124,781.3 to 170,140.2)
Cardiomyopathy and myocarditis	70,239.7<br>(65,789.2 to 74,808.5)	30,690.7<br>(28,696.9 to 32,630.5)	39,549.0<br>(36,931.4 to 42,349.5)	81,882.2<br>(76,677.6 to 87,108.0)	35,902.0<br>(33,660.5 to 38,373.9)	45,980.2<br>(42,883.5 to 49,049.6)	97,384.9<br>(91,353.3 to 103,733.7)	42,425.4<br>(39,638.5 to 45,190.6)	54,959.5<br>(51,490.0 to 58,699.4)
Endocarditis	1,341.2<br>(1,266.4 to 1,420.9)	746.4<br>(701.3 to 791.2)	594.8<br>(561.8 to 631.1)	1,537.9<br>(1,459.2 to 1,623.0)	859.8<br>(812.3 to 908.6)	678.1<br>(642.8 to 715.8)	1,744.5<br>(1,653.3 to 1,840.8)	971.4<br>(920.9 to 1,027.2)	773.1<br>(733.9 to 813.8)
Hemorrhagic and other stroke	384,381.9<br>(374,890.0 to 394,286.8)	194,301.9<br>(189,414.9 to 199,257.9)	190,079.9<br>(185,135.0 to 195,570.3)	434,174.3<br>(424,461.2 to 444,505.6)	218,454.6<br>(213,458.4 to 223,345.0)	215,719.7<br>(210,557.5 to 221,275.3)	491,265.3<br>(480,603.2 to 502,660.7)	245,730.4<br>(240,360.2 to 251,346.5)	245,534.9<br>(239,997.6 to 251,973.4)
Hypertensive heart disease	112,203.6<br>(104,257.5 to 120,140.4)	47,977.3<br>(44,230.5 to 51,865.1)	64,226.3<br>(59,906.3 to 68,741.2)	132,016.7<br>(122,949.8 to 141,174.4)	56,434.2<br>(52,058.5 to 60,916.0)	75,582.5<br>(70,502.4 to 80,717.2)	151,124.2<br>(140,971.7 to 161,632.9)	64,296.6<br>(59,433.7 to 69,604.7)	86,827.6<br>(80,976.2 to 92,826.5)
Ischemic heart disease	1,132,415.6<br>(997,863.6 to 1,294,837.7)	382,991.5<br>(341,501.2 to 432,351.8)	749,424.1<br>(654,541.3 to 865,310.1)	1,334,317.5<br>(1,175,462.6 to 1,522,805.1)	451,087.4<br>(402,746.8 to 506,387.0)	883,230.1<br>(768,847.9 to 1,023,268.5)	1,562,357.2<br>(1,379,377.8 to 1,776,072.4)	526,389.9<br>(471,555.6 to 589,362.2)	1,035,967.3<br>(904,131.7 to 1,197,871.2)
Ischemic stroke	337,661.8<br>(329,781.7 to 345,734.2)	175,536.1<br>(171,006.3 to 180,020.7)	162,125.7<br>(158,299.5 to 166,192.3)	383,009.7<br>(374,778.4 to 391,985.6)	199,505.7<br>(194,721.3 to 204,495.6)	183,504.0<br>(179,708.4 to 187,802.5)	431,002.8<br>(421,600.6 to 440,544.9)	223,373.5<br>(218,319.3 to 228,581.1)	207,629.3<br>(203,174.0 to 212,462.3)
Other cardiovascular and circulatory diseases	4,100,823.3<br>(3,795,922.3 to 4,423,619.3)	2,474,755.7<br>(2,276,581.3 to 2,670,016.2)	1,626,067.5<br>(1,457,025.9 to 1,815,835.2)	4,754,600.9<br>(4,386,348.5 to 5,125,276.0)	2,811,108.0<br>(2,580,880.0 to 3,056,229.9)	1,943,492.9<br>(1,750,614.5 to 2,172,754.3)	5,456,970.6<br>(5,055,575.6 to 5,876,229.3)	3,228,957.5<br>(2,981,195.2 to 3,492,347.1)	2,228,012.8<br>(2,007,258.3 to 2,478,956.8)
Peripheral artery disease	2,116,526.4<br>(1,844,458.3 to 2,455,103.5)	1,047,813.3<br>(910,136.1 to 1,221,079.9)	1,068,713.1<br>(928,914.2 to 1,238,985.9)	2,439,655.6<br>(2,123,499.7 to 2,841,513.8)	1,196,307.2<br>(1,039,911.3 to 1,392,195.9)	1,243,348.5<br>(1,083,357.7 to 1,452,081.7)	2,772,399.4<br>(2,409,908.6 to 3,229,364.6)	1,354,409.3<br>(1,179,493.6 to 1,584,384.1)	1,417,990.0<br>(1,233,362.2 to 1,658,447.5)

eTable 3b. Prevalent Cases of Cardiovascular Causes for Years 2005-2015, with Upper and Lower Uncertainty Bounds

	2005			2010			2015		
	Both	Female	Male	Both	Female	Male	Both	Female	Male
**Global**									
Atrial fibrillation and flutter	25,972,916.8 (23,415,025.0 to 28,914,427.2)	11,322,923.2 (10,181,595.2 to 12,680,487.6)	14,649,993.2 (13,169,850.3 to 16,360,136.2)	29,291,292.6 (26,408,048.2 to 32,683,726.0)	12,614,970.9 (11,321,421.3 to 14,132,562.5)	16,676,322.6 (14,986,719.6 to 18,633,227.4)	33,294,320.5 (29,959,778.3 to 37,202,042.0)	14,233,577.3 (12,755,732.8 to 15,923,714.5)	19,060,742.6 (17,125,194.8 to 21,369,172.2)
Cardiomyopathy and myocarditis	2,002,549.9 (1,905,403.9 to 2,093,217.5)	1,082,389.8 (1,028,336.6 to 1,135,770.1)	920,160.1 (876,577.4 to 964,133.9)	2,251,299.0 (2,136,573.8 to 2,359,228.3)	1,221,382.3 (1,155,272.0 to 1,283,672.8)	1,029,916.8 (978,668.4 to 1,081,379.1)	2,536,768.0 (2,404,778.9 to 2,661,364.5)	1,382,806.8 (1,305,845.3 to 1,458,330.1)	1,153,961.2 (1,097,698.6 to 1,212,526.0)
Endocarditis	94,244.9 (88,560.9 to 100,846.1)	68,894.0 (64,813.6 to 73,467.5)	25,350.9 (23,589.0 to 27,337.8)	103,673.0 (97,570.9 to 111,081.7)	75,419.5 (70,963.0 to 80,665.4)	28,253.5 (26,312.2 to 30,466.4)	115,670.7 (108,053.1 to 124,766.3)	84,078.3 (78,594.0 to 90,548.5)	31,592.4 (29,311.0 to 34,202.4)
Hemorrhagic and other stroke	15,661,003.2 (15,338,627.1 to 16,020,094.4)	7,604,839.6 (7,443,002.9 to 7,780,719.7)	8,056,163.5 (7,890,820.9 to 8,234,745.2)	16,896,596.9 (16,555,823.8 to 17,281,109.4)	8,084,965.0 (7,911,636.5 to 8,271,052.6)	8,811,632.6 (8,636,766.8 to 9,007,357.3)	18,669,621.6 (18,258,729.0 to 19,124,482.3)	8,900,796.9 (8,700,080.7 to 9,121,276.5)	9,768,824.3 (9,543,516.6 to 10,012,684.7)
Hypertensive heart disease	4,440,375.5 (4,181,207.6 to 4,691,139.3)	2,491,837.0 (2,341,202.2 to 2,635,812.7)	1,948,538.5 (1,837,805.8 to 2,063,169.8)	5,200,139.2 (4,894,429.5 to 5,504,762.6)	2,893,920.6 (2,720,720.5 to 3,063,857.2)	2,306,218.8 (2,173,811.5 to 2,444,169.5)	6,086,243.2 (5,732,653.4 to 6,434,074.2)	3,366,519.6 (3,164,973.1 to 3,557,979.4)	2,719,723.4 (2,562,521.1 to 2,882,677.8)
Ischemic heart disease	87,821,757.1 (80,432,245.8 to 96,508,868.2)	36,684,739.0 (33,618,292.1 to 40,325,787.9)	51,137,016.6 (46,762,084.5 to 56,155,356.1)	96,966,837.7 (88,592,301.0 to 106,752,837.8)	40,561,662.5 (37,125,583.6 to 44,636,977.1)	56,405,178.2 (51,486,272.4 to 62,234,568.8)	110,550,304.8 (100,675,889.7 to 121,798,659.7)	46,102,254.7 (42,134,035.4 to 50,800,684.3)	64,448,047.5 (58,685,079.0 to 71,017,092.4)
Ischemic stroke	20,467,281.6 (20,008,422.5 to 20,991,381.9)	9,831,782.7 (9,612,767.4 to 10,085,233.4)	10,635,498.6 (10,393,380.2 to 10,906,146.9)	22,207,180.5 (21,719,278.9 to 22,749,138.7)	10,604,734.9 (10,373,427.7 to 10,862,220.5)	11,602,446.3 (11,346,554.1 to 11,894,370.5)	24,929,025.8 (24,362,226.3 to 25,609,959.5)	11,902,196.4 (11,625,158.6 to 12,233,046.1)	13,026,828.7 (12,718,239.5 to 13,382,922.3)
Other cardiovascular and circulatory diseases	72,771,723.0 (68,089,703.7 to 77,762,925.0)	34,469,238.8 (32,306,286.1 to 36,948,606.0)	38,302,483.8 (35,273,562.6 to 41,688,821.4)	80,425,512.4 (75,346,083.4 to 86,040,625.9)	38,436,718.2 (35,963,514.2 to 41,160,746.5)	41,988,796.9 (38,722,808.2 to 45,480,569.4)	90,347,588.7 (84,710,656.4 to 96,658,717.5)	43,536,603.7 (40,727,282.2 to 46,518,087.9)	46,810,982.7 (43,331,373.8 to 50,439,841.3)
Peripheral artery disease	115,109,151.0 (101,439,286.1 to 131,404,852.3)	61,146,628.0 (53,832,225.9 to 69,563,592.2)	53,962,521.3 (47,448,694.7 to 61,885,680.8)	133,345,710.7 (117,528,239.3 to 151,949,856.5)	70,493,109.4 (62,394,969.0 to 80,219,817.4)	62,852,606.2 (55,267,003.1 to 71,862,332.5)	154,650,579.8 (136,318,041.8 to 176,210,888.8)	81,755,712.3 (72,344,609.4 to 92,826,953.7)	72,894,864.0 (64,169,063.8 to 83,410,627.0)
**Central Asia**									
Atrial fibrillation and flutter	305,544.0 (266,378.3 to 350,070.7)	142,195.8 (122,815.0 to 165,685.8)	163,348.3 (141,208.2 to 186,669.0)	329,407.2 (287,282.6 to 375,627.4)	150,171.9 (129,666.0 to 174,185.8)	179,235.3 (154,662.0 to 205,860.0)	376,888.4 (328,238.3 to 429,352.0)	171,245.1 (147,586.0 to 198,567.8)	205,643.3 (176,989.3 to 236,115.4)
Cardiomyopathy and myocarditis	8,818.6 (8,273.6 to 9,353.7)	4,083.0 (3,833.2 to 4,331.2)	4,735.6 (4,421.2 to 5,066.9)	9,979.0 (9,404.4 to 10,584.2)	4,645.8 (4,365.8 to 4,921.6)	5,333.2 (4,994.8 to 5,690.3)	11,136.1 (10,471.1 to 11,811.9)	5,183.1 (4,880.4 to 5,489.1)	5,953.0 (5,564.1 to 6,350.9)
Endocarditis	504.4 (485.0 to 522.4)	265.1 (254.1 to 276.0)	239.3 (230.4 to 247.6)	550.6 (528.8 to 571.2)	283.9 (272.5 to 295.8)	266.8 (256.3 to 276.2)	616.6 (589.0 to 643.3)	315.7 (300.7 to 331.3)	300.9 (288.2 to 313.8)
Hemorrhagic and other stroke	244,101.2 (239,466.5 to 248,758.0)	133,162.1 (130,629.6 to 135,892.2)	110,939.1 (108,728.1 to 113,180.1)	257,507.8 (252,762.6 to 262,572.3)	139,799.9 (137,163.3 to 142,708.8)	117,707.8 (115,512.5 to 120,032.8)	285,030.7 (279,091.1 to 291,260.1)	155,972.9 (152,515.4 to 159,671.6)	129,057.8 (126,281.0 to 131,955.4)
Hypertensive heart disease	28,066.4 (25,645.7 to 30,628.1)	17,338.0 (15,850.6 to 18,947.5)	10,728.4 (9,706.3 to 11,924.1)	31,122.4 (28,414.0 to 33,865.3)	19,044.0 (17,384.4 to 20,791.2)	12,078.4 (10,992.7 to 13,237.8)	35,367.7 (32,494.6 to 38,286.5)	21,365.8 (19,512.9 to 23,226.3)	14,001.9 (12,767.5 to 15,371.4)
Ischemic heart disease	1,658,058.3 (1,523,024.4 to 1,806,351.5)	803,107.7 (732,144.9 to 887,384.3)	854,950.6 (787,452.3 to 927,429.4)	1,775,799.0 (1,630,658.1 to 1,940,581.0)	852,525.9 (774,362.1 to 940,442.9)	923,273.1 (845,834.5 to 1,008,821.8)	1,959,856.4 (1,792,534.2 to 2,148,359.8)	938,381.2 (850,809.1 to 1,037,029.3)	1,021,475.3 (934,886.1 to 1,115,020.9)
Ischemic stroke	294,329.9 (288,015.3 to 301,099.4)	159,797.3 (156,299.8 to 163,611.7)	134,532.6 (131,374.2 to 137,803.7)	304,947.4 (298,598.1 to 311,348.8)	166,013.6 (162,472.8 to 169,666.0)	138,933.8 (136,168.3 to 142,073.6)	344,977.1 (337,341.1 to 353,208.7)	189,936.7 (185,552.8 to 194,748.6)	155,040.5 (151,403.7 to 159,069.8)
Other cardiovascular and circulatory diseases	176,108.1 (162,364.3 to 190,965.5)	68,283.3 (63,057.8 to 74,075.3)	107,824.8 (98,848.4 to 117,408.2)	193,614.4 (178,832.1 to 210,475.9)	74,540.4 (68,816.1 to 81,058.7)	119,074.0 (108,851.7 to 130,095.8)	221,637.7 (203,309.4 to 240,228.9)	85,358.2 (78,339.5 to 92,507.4)	136,279.6 (123,820.7 to 149,069.7)
Peripheral artery disease	1,323,910.8 (1,156,238.4 to 1,527,332.3)	748,607.1 (654,687.1 to 862,372.9)	575,303.7 (500,728.7 to 664,628.6)	1,469,633.9 (1,284,834.6 to 1,688,273.5)	829,329.7 (727,655.3 to 952,274.5)	640,304.2 (559,783.3 to 738,420.4)	1,691,612.6 (1,488,889.6 to 1,932,672.0)	953,780.4 (838,410.1 to 1,091,304.5)	737,832.4 (644,900.0 to 848,175.2)
**Eastern Europe**									
Atrial fibrillation and flutter	1,624,497.1 (1,414,970.8 to 1,854,535.8)	877,933.1 (751,495.9 to 1,022,555.0)	746,564.0 (638,308.8 to 858,200.7)	1,669,700.7 (1,458,659.6 to 1,916,302.2)	898,699.4 (771,684.4 to 1,047,731.3)	771,001.3 (666,804.8 to 886,483.4)	1,892,536.4 (1,652,447.8 to 2,163,648.9)	1,020,943.2 (879,316.5 to 1,186,633.5)	871,593.1 (753,993.3 to 1,000,673.0)
Cardiomyopathy and myocarditis	85,984.5 (79,607.9 to 92,714.5)	44,484.1 (40,892.0 to 48,422.5)	41,500.4 (37,754.2 to 45,403.6)	96,679.0 (88,690.3 to 104,892.0)	49,220.0 (44,577.5 to 53,908.1)	47,459.0 (43,026.0 to 52,488.5)	111,937.9 (102,650.8 to 121,770.3)	58,516.7 (53,290.3 to 64,068.6)	53,421.2 (47,964.8 to 59,075.0)
Endocarditis	1,963.8 (1,880.4 to 2,047.2)	1,141.1 (1,083.2 to 1,199.9)	822.6 (785.0 to 858.3)	2,117.1 (2,019.5 to 2,216.6)	1,214.7 (1,150.3 to 1,282.5)	902.4 (860.5 to 943.8)	2,326.6 (2,205.9 to 2,467.5)	1,347.5 (1,263.6 to 1,446.5)	979.1 (931.4 to 1,028.7)
Hemorrhagic and other stroke	1,073,232.3 (1,046,977.4 to 1,099,432.8)	648,250.3 (632,183.9 to 664,838.7)	424,982.0 (411,800.5 to 437,705.2)	1,036,523.5 (1,012,708.9 to 1,061,811.7)	623,524.6 (609,392.1 to 639,638.5)	412,998.9 (401,270.1 to 425,525.6)	1,129,261.4 (1,103,083.5 to 1,156,455.2)	683,093.6 (666,554.2 to 701,155.0)	446,167.8 (433,515.1 to 459,717.3)
Hypertensive heart disease	76,445.7 (69,047.2 to 84,897.1)	58,808.4 (52,819.1 to 65,852.1)	17,637.3 (15,835.9 to 19,685.1)	82,824.4 (74,503.5 to 92,460.5)	61,766.6 (55,134.1 to 69,692.7)	21,057.8 (18,804.2 to 23,558.5)	95,731.7 (86,353.7 to 107,102.1)	70,907.0 (63,619.2 to 79,932.9)	24,824.7 (21,972.0 to 27,732.8)
Ischemic heart disease	12,490,256.5 (11,522,480.2 to 13,460,872.5)	5,695,991.5 (5,212,995.1 to 6,176,309.5)	6,794,265.1 (6,231,520.3 to 7,396,455.3)	12,278,456.9 (11,282,354.9 to 13,361,015.4)	5,609,971.6 (5,115,427.1 to 6,103,199.1)	6,668,485.4 (6,091,928.4 to 7,305,242.8)	13,228,694.5 (12,169,855.1 to 14,388,370.6)	6,092,839.4 (5,547,898.3 to 6,669,666.9)	7,135,854.8 (6,530,196.0 to 7,789,227.5)
Ischemic stroke	1,847,535.5 (1,797,199.4 to 1,902,240.1)	1,145,224.7 (1,112,908.3 to 1,179,563.3)	702,310.8 (677,630.7 to 727,238.5)	1,766,923.9 (1,721,823.6 to 1,819,199.5)	1,093,689.1 (1,064,600.0 to 1,125,702.9)	673,234.8 (651,022.1 to 696,285.1)	1,939,625.7 (1,886,897.2 to 1,993,437.3)	1,201,816.7 (1,169,099.8 to 1,236,376.0)	737,808.9 (713,335.6 to 763,065.6)
Other cardiovascular and circulatory diseases	668,652.7 (602,279.6 to 736,610.2)	421,647.3 (377,161.7 to 469,598.0)	247,005.4 (220,407.7 to 278,931.7)	722,216.1 (645,728.6 to 802,733.0)	430,304.1 (383,172.7 to 477,330.6)	291,912.0 (257,672.2 to 329,508.4)	828,365.0 (741,867.6 to 923,565.9)	490,646.6 (435,557.3 to 549,629.2)	337,718.4 (294,862.5 to 382,464.4)
Peripheral artery disease	7,682,655.8 (6,674,276.6 to 8,854,735.1)	4,926,694.8 (4,300,106.6 to 5,664,065.0)	2,755,960.9 (2,394,298.4 to 3,192,506.7)	8,084,882.2 (7,064,025.5 to 9,335,234.5)	5,223,229.3 (4,562,694.8 to 6,003,015.6)	2,861,653.1 (2,500,599.3 to 3,301,146.0)	9,324,997.5 (8,157,323.3 to 10,652,514.7)	5,987,518.7 (5,232,082.0 to 6,839,527.4)	3,337,478.7 (2,915,576.0 to 3,841,688.3)
**Central Europe**									
Atrial fibrillation and flutter	1,163,921.5 (1,090,765.0 to 1,245,953.6)	586,076.8 (539,624.1 to 634,993.1)	577,844.7 (545,539.3 to 614,287.4)	1,255,874.9 (1,178,319.9 to 1,343,985.4)	630,479.5 (582,077.7 to 684,202.8)	625,395.4 (592,354.7 to 663,129.0)	1,362,096.7 (1,275,488.2 to 1,461,917.3)	681,321.2 (628,068.0 to 742,150.7)	680,775.5 (642,637.8 to 721,842.4)
Cardiomyopathy and myocarditis	68,895.0 (64,321.7 to 73,284.2)	38,658.0 (35,851.4 to 41,309.3)	30,237.0 (28,140.9 to 32,309.6)	76,807.5 (71,561.7 to 82,112.6)	43,607.0 (40,217.0 to 46,993.5)	33,200.5 (30,873.5 to 35,511.8)	85,196.7 (79,157.0 to 91,509.7)	48,791.1 (44,723.8 to 52,853.4)	36,405.6 (33,816.4 to 39,082.0)
Endocarditis	1,065.7 (1,021.6 to 1,107.0)	308.9 (291.8 to 326.3)	756.8 (724.6 to 788.7)	1,141.9 (1,095.2 to 1,187.0)	313.7 (295.5 to 332.5)	828.2 (792.2 to 865.3)	1,181.8 (1,127.7 to 1,236.7)	314.5 (294.4 to 335.7)	867.3 (824.6 to 914.3)
Hemorrhagic and other stroke	473,886.3 (464,241.3 to 484,805.3)	272,605.3 (266,386.5 to 279,421.7)	201,281.1 (196,675.2 to 206,157.6)	460,247.2 (450,447.5 to 471,111.7)	264,113.0 (257,901.3 to 271,031.6)	196,134.2 (191,773.1 to 200,605.0)	482,419.9 (470,995.2 to 495,973.3)	278,485.6 (271,056.8 to 287,367.1)	203,934.3 (199,058.3 to 209,297.1)
Hypertensive heart disease	111,697.7 (101,869.0 to 121,762.9)	75,862.1 (69,000.7 to 82,777.3)	35,835.7 (32,830.0 to 39,181.3)	123,057.6 (112,003.5 to 134,433.7)	82,814.1 (75,086.8 to 91,207.9)	40,243.6 (36,757.3 to 44,170.3)	133,931.2 (121,672.8 to 146,238.7)	89,178.1 (80,956.4 to 97,913.3)	44,753.1 (40,609.5 to 48,989.0)
Ischemic heart disease	4,569,442.1 (4,140,773.3 to 5,043,559.7)	1,919,932.4 (1,736,238.6 to 2,133,768.5)	2,649,509.8 (2,397,415.3 to 2,913,237.6)	4,710,734.2 (4,263,515.4 to 5,228,523.9)	1,988,372.1 (1,797,166.1 to 2,201,174.3)	2,722,362.2 (2,456,788.8 to 3,011,336.1)	4,887,249.7 (4,421,083.6 to 5,396,339.1)	2,054,736.6 (1,855,428.2 to 2,273,925.3)	2,832,513.1 (2,556,599.4 to 3,130,359.8)
Ischemic stroke	745,526.9 (726,633.4 to 766,336.7)	394,679.2 (384,646.3 to 406,863.7)	350,847.7 (341,063.3 to 360,456.4)	763,113.2 (744,189.7 to 783,496.5)	403,377.3 (392,299.2 to 414,648.3)	359,735.9 (350,665.9 to 369,072.5)	796,433.7 (775,591.1 to 820,957.2)	424,906.3 (412,678.5 to 439,291.1)	371,527.4 (361,686.2 to 382,321.3)
Other cardiovascular and circulatory diseases	2,202,674.4 (2,040,324.3 to 2,371,646.7)	1,055,291.0 (979,085.6 to 1,138,863.4)	1,147,383.4 (1,053,213.2 to 1,241,550.5)	2,375,892.8 (2,194,457.4 to 2,567,989.4)	1,122,557.2 (1,033,692.5 to 1,211,454.9)	1,253,335.6 (1,155,673.7 to 1,361,517.8)	2,533,555.0 (2,326,798.3 to 2,748,081.5)	1,179,003.9 (1,085,216.7 to 1,281,067.6)	1,354,551.1 (1,236,152.1 to 1,474,067.2)
Peripheral artery disease	5,002,840.6 (4,391,157.7 to 5,748,067.5)	2,872,207.1 (2,515,830.3 to 3,292,676.7)	2,130,633.6 (1,864,848.1 to 2,470,050.1)	5,466,483.9 (4,792,580.9 to 6,243,408.5)	3,137,407.6 (2,752,675.6 to 3,576,907.8)	2,329,076.4 (2,037,548.8 to 2,682,084.6)	5,907,813.7 (5,172,148.4 to 6,723,959.9)	3,400,550.7 (2,974,199.6 to 3,864,348.0)	2,507,263.0 (2,191,802.7 to 2,872,678.4)
**Western Europe**									
Atrial fibrillation and flutter	4,954,836.9 (4,471,842.9 to 5,560,761.0)	2,254,013.6 (2,006,985.7 to 2,556,049.4)	2,700,823.4 (2,429,938.1 to 3,006,049.1)	5,418,130.3 (4,877,762.0 to 6,093,610.0)	2,422,115.0 (2,159,158.0 to 2,751,255.3)	2,996,015.3 (2,690,364.3 to 3,346,308.5)	5,958,990.3 (5,366,828.5 to 6,717,292.9)	2,646,729.4 (2,350,831.3 to 3,002,320.1)	3,312,260.9 (2,982,571.3 to 3,709,443.7)
Cardiomyopathy and myocarditis	226,642.8 (212,848.8 to 239,845.6)	142,873.8 (132,691.4 to 152,519.2)	83,769.0 (78,719.9 to 89,001.5)	245,213.5 (228,724.1 to 262,104.7)	153,731.7 (141,560.0 to 166,057.5)	91,481.9 (85,335.6 to 97,997.6)	263,620.2 (244,618.5 to 282,494.3)	165,969.0 (152,310.3 to 179,523.4)	97,651.2 (90,832.7 to 104,810.6)
Endocarditis	32,111.5 (29,975.2 to 34,580.2)	28,757.5 (26,747.7 to 31,119.6)	3,354.0 (3,215.5 to 3,499.6)	33,635.9 (31,379.5 to 36,220.3)	30,025.7 (27,932.1 to 32,431.0)	3,610.2 (3,459.7 to 3,776.4)	35,838.9 (32,840.6 to 39,325.4)	31,954.1 (29,120.6 to 35,218.8)	3,884.7 (3,680.8 to 4,099.4)
Hemorrhagic and other stroke	825,463.1 (807,202.5 to 846,197.1)	436,592.0 (426,665.9 to 447,560.1)	388,871.0 (379,930.9 to 399,060.0)	865,749.9 (845,852.5 to 886,921.1)	454,216.7 (443,866.6 to 465,203.7)	411,533.2 (401,901.6 to 422,034.0)	926,435.3 (903,479.7 to 950,682.8)	482,431.8 (470,375.0 to 495,455.1)	444,003.5 (432,459.9 to 456,480.7)
Hypertensive heart disease	246,469.9 (227,660.8 to 265,277.0)	160,419.4 (148,056.1 to 173,067.4)	86,050.5 (79,301.6 to 92,521.2)	264,331.9 (241,791.2 to 287,766.0)	167,082.5 (152,220.2 to 182,605.5)	97,249.4 (88,702.3 to 106,266.9)	266,257.4 (241,981.1 to 291,055.1)	167,087.9 (151,523.9 to 183,319.9)	99,169.5 (89,961.2 to 108,744.5)
Ischemic heart disease	8,613,131.8 (7,823,669.5 to 9,589,925.0)	3,531,653.2 (3,153,829.8 to 3,996,703.6)	5,081,478.7 (4,645,001.8 to 5,609,803.1)	8,818,312.1 (8,004,317.0 to 9,830,283.7)	3,597,631.5 (3,225,060.7 to 4,072,316.6)	5,220,680.6 (4,777,339.1 to 5,762,077.2)	9,054,333.5 (8,208,626.1 to 10,087,602.1)	3,670,540.1 (3,286,094.0 to 4,154,807.5)	5,383,793.3 (4,897,589.2 to 5,957,279.8)
Ischemic stroke	1,640,601.9 (1,599,802.1 to 1,685,688.7)	825,153.9 (804,316.3 to 846,742.2)	815,448.1 (794,471.6 to 838,219.9)	1,723,760.9 (1,679,602.1 to 1,768,636.2)	857,789.1 (836,345.4 to 879,907.3)	865,971.8 (843,677.6 to 889,244.2)	1,865,822.2 (1,817,011.5 to 1,917,657.7)	924,110.6 (899,639.6 to 951,652.1)	941,711.6 (915,999.0 to 969,970.1)
Other cardiovascular and circulatory diseases	6,810,101.0 (6,367,719.8 to 7,286,596.1)	3,559,854.1 (3,327,333.5 to 3,815,942.7)	3,250,247.0 (3,018,885.0 to 3,497,949.4)	7,168,217.3 (6,626,154.8 to 7,734,055.2)	3,594,521.6 (3,331,178.8 to 3,868,737.3)	3,573,695.7 (3,280,105.7 to 3,872,946.3)	7,527,021.5 (6,960,964.1 to 8,160,391.6)	3,726,535.6 (3,449,729.3 to 4,024,071.1)	3,800,485.9 (3,486,261.1 to 4,145,171.9)
Peripheral artery disease	15,208,378.4 (13,491,847.7 to 17,220,039.5)	8,463,794.8 (7,494,080.3 to 9,574,704.2)	6,744,583.9 (5,967,024.6 to 7,638,132.5)	16,324,763.5 (14,439,572.7 to 18,588,111.1)	9,023,465.9 (7,946,093.1 to 10,231,926.0)	7,301,297.7 (6,419,508.4 to 8,378,654.0)	17,636,671.2 (15,552,320.6 to 20,051,490.6)	9,723,721.6 (8,561,244.3 to 11,068,696.6)	7,912,949.5 (6,933,054.0 to 9,067,674.5)
**High-income North America**									
Atrial fibrillation and flutter	5,129,573.6 (4,878,489.0 to 5,405,520.2)	2,348,302.8 (2,236,687.0 to 2,478,079.9)	2,781,270.8 (2,637,672.3 to 2,938,353.2)	5,699,479.9 (5,424,101.5 to 6,018,355.1)	2,547,060.6 (2,424,118.1 to 2,689,459.6)	3,152,419.3 (2,987,329.5 to 3,338,818.1)	6,309,968.2 (5,962,257.9 to 6,691,315.0)	2,780,915.9 (2,627,555.5 to 2,957,851.1)	3,529,052.2 (3,323,532.4 to 3,756,655.6)
Cardiomyopathy and myocarditis	371,678.3 (351,704.7 to 392,448.0)	210,177.4 (197,611.8 to 223,501.1)	161,501.0 (153,124.6 to 170,727.2)	415,996.9 (393,467.9 to 439,862.0)	234,659.0 (220,882.1 to 249,933.7)	181,337.9 (171,859.4 to 191,792.0)	455,212.2 (430,195.0 to 481,216.2)	256,640.6 (241,397.1 to 273,062.7)	198,571.6 (188,000.9 to 210,108.9)
Endocarditis	10,286.7 (8,953.2 to 11,760.3)	5,315.4 (4,611.7 to 6,188.7)	4,971.3 (4,271.3 to 5,716.3)	11,000.9 (9,611.6 to 12,566.6)	5,503.0 (4,788.0 to 6,359.9)	5,497.9 (4,727.8 to 6,341.4)	11,753.9 (10,198.5 to 13,544.2)	5,844.1 (5,082.7 to 6,841.2)	5,909.8 (5,073.5 to 6,865.4)
Hemorrhagic and other stroke	325,531.1 (315,755.0 to 336,393.9)	172,318.0 (167,182.3 to 178,149.7)	153,213.1 (148,462.7 to 158,178.4)	347,886.8 (338,886.5 to 358,344.9)	181,191.2 (176,407.6 to 186,662.1)	166,695.5 (162,248.7 to 171,719.6)	403,244.6 (391,490.3 to 416,046.6)	208,998.6 (202,896.5 to 215,686.1)	194,246.0 (188,360.7 to 200,618.7)
Hypertensive heart disease	394,081.1 (369,676.1 to 421,194.4)	239,815.0 (224,231.3 to 256,958.8)	154,266.1 (144,641.6 to 164,892.3)	444,438.4 (417,322.7 to 475,196.3)	266,606.1 (250,012.4 to 285,300.4)	177,832.3 (166,859.7 to 190,125.9)	489,833.6 (460,403.5 to 523,381.5)	290,359.9 (272,555.0 to 310,891.2)	199,473.7 (187,086.3 to 213,284.0)
Ischemic heart disease	5,991,471.0 (5,784,481.0 to 6,209,239.7)	2,640,315.7 (2,555,037.2 to 2,729,224.7)	3,351,155.3 (3,220,429.1 to 3,489,244.6)	5,992,600.8 (5,818,410.2 to 6,170,931.6)	2,663,998.6 (2,585,332.4 to 2,740,758.5)	3,328,602.2 (3,227,082.6 to 3,436,029.8)	6,278,341.5 (6,098,359.1 to 6,483,077.7)	2,789,029.4 (2,708,288.3 to 2,870,652.6)	3,489,312.1 (3,371,951.2 to 3,618,276.8)
Ischemic stroke	1,026,743.3 (996,764.7 to 1,059,574.1)	527,211.9 (511,288.0 to 544,649.2)	499,531.4 (484,774.6 to 515,246.0)	1,103,849.5 (1,074,855.0 to 1,135,957.8)	559,561.3 (544,485.8 to 576,896.2)	544,288.2 (530,056.9 to 560,536.1)	1,167,328.3 (1,133,547.9 to 1,205,267.4)	581,089.3 (563,547.6 to 599,833.4)	586,239.0 (569,087.9 to 605,366.9)
Other cardiovascular and circulatory diseases	5,673,188.2 (5,338,637.4 to 6,035,578.4)	2,986,195.1 (2,812,822.8 to 3,176,927.0)	2,686,993.1 (2,514,580.7 to 2,877,831.7)	6,297,376.9 (5,915,946.0 to 6,701,888.9)	3,256,856.9 (3,067,102.3 to 3,462,104.8)	3,040,519.9 (2,845,528.6 to 3,267,134.5)	6,918,564.6 (6,493,959.3 to 7,371,561.7)	3,538,666.7 (3,336,735.7 to 3,762,095.3)	3,379,897.9 (3,158,946.8 to 3,617,125.3)
Peripheral artery disease	6,823,990.5 (6,403,506.1 to 7,245,762.1)	3,557,878.6 (3,316,292.8 to 3,819,907.8)	3,266,111.8 (3,090,072.1 to 3,443,512.9)	7,682,098.8 (7,313,665.7 to 8,081,003.2)	3,959,034.9 (3,756,830.8 to 4,181,202.2)	3,723,063.9 (3,543,697.5 to 3,918,516.4)	8,697,872.3 (8,360,630.3 to 9,039,886.9)	4,529,008.4 (4,354,785.3 to 4,705,599.1)	4,168,863.9 (3,998,069.0 to 4,343,753.1)
**High-income Asia Pacific**									
Atrial fibrillation and flutter	856,439.6 (747,502.1 to 985,043.4)	255,736.5 (221,190.4 to 300,658.9)	600,703.1 (522,899.0 to 688,074.1)	989,077.6 (864,163.1 to 1,140,361.5)	294,798.5 (253,352.8 to 345,629.3)	694,279.1 (601,538.4 to 796,023.2)	1,127,073.5 (980,138.2 to 1,302,313.4)	336,984.5 (289,901.1 to 393,095.9)	790,089.1 (686,268.3 to 911,048.6)
Cardiomyopathy and myocarditis	124,210.0 (117,237.9 to 131,727.7)	76,864.2 (71,959.0 to 82,010.4)	47,345.8 (44,588.8 to 50,257.0)	137,544.4 (129,146.3 to 146,180.2)	87,057.4 (81,353.9 to 93,065.7)	50,487.1 (47,517.3 to 53,601.9)	149,682.3 (140,605.2 to 159,446.8)	96,342.4 (89,803.8 to 103,296.7)	53,339.8 (50,182.7 to 56,631.1)
Endocarditis	3,482.5 (3,097.9 to 3,903.2)	2,110.3 (1,864.5 to 2,388.6)	1,372.2 (1,225.9 to 1,546.6)	3,729.2 (3,292.0 to 4,235.7)	2,253.4 (1,972.4 to 2,574.7)	1,475.8 (1,310.3 to 1,664.4)	4,013.1 (3,539.6 to 4,532.8)	2,417.3 (2,124.8 to 2,750.1)	1,595.8 (1,417.4 to 1,805.1)
Hemorrhagic and other stroke	529,566.7 (516,575.3 to 543,948.8)	267,262.8 (260,580.7 to 274,684.5)	262,303.8 (255,854.1 to 269,738.5)	546,965.7 (534,490.1 to 560,473.9)	273,636.6 (267,327.2 to 280,775.6)	273,329.0 (267,169.9 to 280,202.0)	587,292.0 (572,980.3 to 603,859.2)	294,104.4 (286,904.4 to 302,836.1)	293,187.7 (285,827.8 to 301,760.8)
Hypertensive heart disease	256,283.8 (237,397.7 to 276,583.7)	172,017.5 (159,302.4 to 185,723.8)	84,266.3 (78,148.7 to 90,785.0)	298,380.0 (276,997.8 to 322,308.3)	198,834.0 (184,133.7 to 215,052.4)	99,546.0 (92,343.4 to 107,633.2)	332,646.3 (308,031.4 to 359,908.2)	221,056.0 (204,358.1 to 240,112.8)	111,590.3 (103,182.9 to 120,509.3)
Ischemic heart disease	2,104,517.3 (1,990,543.0 to 2,237,102.0)	991,450.3 (938,148.7 to 1,052,636.0)	1,113,067.0 (1,047,369.6 to 1,186,436.0)	2,336,624.5 (2,208,452.6 to 2,482,013.2)	1,100,916.0 (1,042,396.6 to 1,165,436.5)	1,235,708.5 (1,167,682.2 to 1,315,988.3)	2,596,645.2 (2,457,585.0 to 2,752,644.5)	1,216,369.5 (1,151,727.2 to 1,286,526.4)	1,380,275.8 (1,300,678.0 to 1,469,956.3)
Ischemic stroke	1,066,032.5 (1,039,042.2 to 1,095,921.4)	473,519.4 (461,096.0 to 487,003.9)	592,513.1 (576,724.3 to 610,737.7)	1,101,967.8 (1,076,237.2 to 1,130,114.9)	490,412.1 (478,655.9 to 503,284.8)	611,555.7 (597,120.1 to 628,184.7)	1,193,488.1 (1,162,143.7 to 1,229,285.9)	530,971.0 (516,892.6 to 546,687.4)	662,517.1 (645,073.8 to 683,050.5)
Other cardiovascular and circulatory diseases	2,431,327.8 (2,276,696.9 to 2,614,219.7)	1,174,652.3 (1,099,335.1 to 1,263,076.9)	1,256,675.5 (1,170,540.6 to 1,358,052.8)	2,624,357.9 (2,454,060.2 to 2,822,496.1)	1,272,822.3 (1,190,460.2 to 1,365,349.2)	1,351,535.7 (1,259,074.9 to 1,458,491.3)	2,792,587.3 (2,613,965.8 to 3,000,556.5)	1,361,179.7 (1,269,857.4 to 1,464,555.2)	1,431,407.7 (1,332,123.3 to 1,543,309.5)
Peripheral artery disease	5,016,608.3 (4,408,057.4 to 5,715,768.0)	2,847,871.2 (2,508,868.0 to 3,233,046.8)	2,168,737.1 (1,895,148.6 to 2,497,452.4)	5,748,586.1 (5,043,425.3 to 6,539,154.0)	3,246,323.2 (2,852,395.9 to 3,694,175.3)	2,502,263.0 (2,190,503.7 to 2,871,359.3)	6,337,310.0 (5,560,904.6 to 7,195,855.1)	3,669,524.6 (3,222,823.7 to 4,170,906.4)	2,667,785.6 (2,332,788.1 to 3,054,075.8)
**Australasia**									
Atrial fibrillation and flutter	188,412.5 (170,355.0 to 208,349.7)	73,402.8 (66,414.3 to 81,591.6)	115,009.7 (103,582.4 to 128,208.2)	214,872.1 (194,753.3 to 237,576.7)	80,530.2 (73,125.7 to 89,656.4)	134,341.9 (120,853.2 to 148,836.4)	239,289.4 (215,934.4 to 265,414.1)	89,838.3 (81,174.2 to 100,275.0)	149,451.1 (134,075.5 to 166,192.1)
Cardiomyopathy and myocarditis	18,579.8 (17,265.4 to 19,925.0)	10,245.9 (9,352.7 to 11,162.2)	8,333.9 (7,759.2 to 8,965.9)	21,138.2 (19,635.1 to 22,663.7)	11,573.1 (10,544.3 to 12,633.6)	9,565.1 (8,851.6 to 10,283.9)	23,438.9 (21,791.8 to 25,204.9)	12,863.9 (11,797.2 to 14,076.2)	10,575.0 (9,840.3 to 11,368.5)
Endocarditis	524.1 (503.7 to 542.4)	257.3 (246.4 to 268.8)	266.7 (257.1 to 276.1)	549.7 (529.0 to 571.4)	263.9 (252.7 to 276.3)	285.8 (274.9 to 296.8)	576.4 (552.2 to 602.4)	273.8 (259.9 to 288.8)	302.5 (290.6 to 315.6)
Hemorrhagic and other stroke	38,964.5 (38,067.2 to 39,915.4)	21,222.7 (20,707.7 to 21,788.5)	17,741.7 (17,268.5 to 18,234.2)	41,537.3 (40,625.5 to 42,613.2)	22,374.8 (21,835.4 to 23,067.8)	19,162.5 (18,700.4 to 19,702.6)	46,455.1 (45,252.5 to 47,851.2)	25,096.8 (24,270.2 to 25,922.9)	21,358.3 (20,809.6 to 22,010.5)
Hypertensive heart disease	12,477.2 (11,330.0 to 13,621.2)	6,458.2 (5,825.7 to 7,091.5)	6,019.0 (5,462.2 to 6,637.5)	14,973.2 (13,560.8 to 16,422.7)	7,937.2 (7,111.2 to 8,802.5)	7,036.0 (6,361.3 to 7,811.5)	16,675.5 (15,152.2 to 18,275.3)	8,728.8 (7,865.3 to 9,679.6)	7,946.6 (7,168.3 to 8,770.3)
Ischemic heart disease	336,329.6 (320,474.3 to 353,820.4)	131,146.8 (123,621.9 to 139,496.8)	205,182.8 (194,993.1 to 216,065.6)	355,917.1 (338,264.2 to 374,261.3)	136,266.4 (128,267.3 to 144,809.0)	219,650.7 (208,116.9 to 231,064.8)	374,391.3 (356,154.4 to 394,637.4)	141,494.3 (133,557.4 to 150,317.5)	232,897.0 (220,557.6 to 245,472.6)
Ischemic stroke	80,755.5 (78,765.9 to 82,916.5)	40,115.9 (39,000.2 to 41,302.5)	40,639.6 (39,471.8 to 41,841.3)	86,709.9 (84,697.5 to 89,098.4)	42,706.1 (41,600.8 to 44,181.6)	44,003.8 (42,880.1 to 45,281.9)	97,731.7 (95,064.1 to 100,797.5)	48,400.7 (46,706.1 to 50,164.6)	49,331.0 (47,985.5 to 50,972.2)
Other cardiovascular and circulatory diseases	270,156.0 (248,466.9 to 293,508.8)	141,930.9 (130,437.5 to 154,603.3)	128,225.1 (116,942.8 to 141,068.0)	299,324.2 (275,948.5 to 324,756.0)	155,276.4 (141,996.5 to 169,382.6)	144,047.8 (130,991.7 to 158,555.6)	329,843.6 (303,909.5 to 357,277.9)	169,893.7 (155,554.3 to 185,000.0)	159,949.8 (145,622.1 to 175,354.2)

Peripheral artery disease	689,896.0 (606,946.7 to 790,159.4)	329,959.1 (290,157.6 to 379,094.4)	359,937.0 (314,716.4 to 418,138.4)	788,728.4 (692,688.0 to 898,903.7)	366,216.2 (323,949.0 to 419,123.0)	422,512.2 (369,104.2 to 483,244.3)	851,902.6 (746,754.2 to 970,531.0)	402,377.1 (352,607.9 to 459,923.5)	449,525.5 (391,594.0 to 515,915.2)
**Southern Latin America**									
Atrial fibrillation and flutter	293,368.9 (255,452.5 to 336,990.4)	107,408.5 (92,577.1 to 125,095.3)	185,960.4 (162,082.5 to 214,053.3)	325,440.6 (283,947.7 to 373,547.8)	118,467.0 (102,169.1 to 137,876.2)	206,973.6 (179,840.4 to 236,804.1)	364,105.3 (319,444.0 to 421,316.7)	134,773.5 (116,818.2 to 157,649.5)	229,331.8 (199,034.0 to 264,200.2)
Cardiomyopathy and myocarditis	24,571.2 (20,815.4 to 28,645.4)	16,585.8 (14,216.6 to 18,904.4)	7,985.3 (6,612.7 to 9,891.8)	27,407.6 (23,627.4 to 31,744.0)	18,380.0 (15,961.0 to 20,917.7)	9,027.7 (7,459.5 to 11,102.2)	30,880.5 (26,712.4 to 35,276.5)	20,610.1 (18,026.7 to 23,228.4)	10,270.4 (8,498.3 to 12,250.8)
Endocarditis	4,306.8 (4,069.2 to 4,571.3)	4,249.4 (4,012.5 to 4,510.9)	57.4 (44.4 to 68.8)	4,880.6 (4,595.1 to 5,192.9)	4,800.8 (4,519.5 to 5,116.1)	79.8 (66.5 to 91.8)	5,530.0 (5,125.8 to 5,962.1)	5,434.8 (5,043.7 to 5,856.3)	95.3 (77.3 to 111.7)
Hemorrhagic and other stroke	108,465.6 (106,019.2 to 111,332.9)	58,699.7 (57,283.5 to 60,265.4)	49,765.9 (48,431.4 to 51,191.0)	114,352.7 (111,581.7 to 117,393.3)	61,849.5 (60,210.8 to 63,583.4)	52,503.2 (51,230.8 to 54,014.8)	126,534.3 (122,934.0 to 130,606.6)	68,748.6 (66,762.9 to 71,057.6)	57,785.7 (56,064.3 to 59,706.0)
Hypertensive heart disease	49,859.5 (42,858.7 to 56,824.4)	32,150.1 (27,827.8 to 36,140.3)	17,709.3 (14,811.2 to 20,857.5)	51,271.2 (44,352.1 to 58,220.4)	33,282.7 (28,995.0 to 37,577.5)	17,988.4 (14,960.1 to 21,198.2)	57,426.4 (49,902.2 to 64,975.4)	37,137.4 (32,432.2 to 41,655.5)	20,289.1 (17,022.0 to 23,583.4)
Ischemic heart disease	411,086.5 (389,921.7 to 432,795.1)	148,344.4 (139,806.5 to 157,054.4)	262,742.1 (248,326.8 to 277,317.9)	440,593.2 (417,147.9 to 465,749.0)	163,574.4 (154,109.8 to 173,332.1)	277,018.7 (262,214.1 to 292,879.6)	488,328.7 (458,416.4 to 516,911.9)	178,842.6 (167,464.2 to 189,853.9)	309,486.1 (289,061.1 to 330,028.1)
Ischemic stroke	175,530.4 (171,167.8 to 180,586.1)	90,153.1 (87,908.3 to 92,736.1)	85,377.3 (82,684.6 to 88,118.9)	185,801.6 (181,106.0 to 190,859.4)	96,303.2 (93,595.5 to 99,315.3)	89,498.4 (87,054.8 to 92,131.5)	206,766.9 (200,751.0 to 213,651.0)	107,942.4 (104,637.9 to 111,837.5)	98,824.5 (95,588.6 to 102,483.1)
Other cardiovascular and circulatory diseases	534,058.7 (418,004.1 to 661,690.6)	327,386.1 (253,687.0 to 400,567.1)	206,672.6 (161,344.4 to 265,509.2)	565,257.9 (446,754.2 to 691,888.5)	339,144.1 (265,989.2 to 408,833.1)	226,113.8 (176,661.4 to 287,037.7)	626,856.1 (507,532.4 to 754,024.9)	373,423.8 (301,074.9 to 444,747.1)	253,432.3 (201,178.8 to 312,104.5)
Peripheral artery disease	1,089,264.0 (954,469.5 to 1,238,883.9)	599,620.1 (525,393.4 to 684,601.4)	489,643.9 (428,537.4 to 562,625.8)	1,222,263.0 (1,076,503.3 to 1,394,184.3)	671,789.8 (590,558.2 to 768,288.9)	550,473.2 (480,055.8 to 633,926.9)	1,336,794.1 (1,181,289.8 to 1,530,375.8)	749,046.6 (659,895.3 to 857,966.5)	587,747.5 (513,484.1 to 681,090.2)
**Andean Latin America**									
Atrial fibrillation and flutter	87,877.0 (76,242.2 to 101,453.2)	34,324.9 (29,380.0 to 40,002.1)	53,552.0 (46,127.3 to 61,818.3)	104,475.5 (90,200.9 to 121,302.2)	40,930.3 (35,309.1 to 47,712.3)	63,545.2 (54,654.0 to 73,505.6)	123,851.3 (107,182.4 to 144,577.3)	48,470.7 (41,666.7 to 56,879.6)	75,380.6 (65,230.2 to 87,391.2)
Cardiomyopathy and myocarditis	7,959.7 (7,433.1 to 8,468.4)	4,293.4 (4,009.7 to 4,577.2)	3,666.3 (3,405.6 to 3,919.1)	9,582.3 (8,959.8 to 10,193.4)	5,268.5 (4,914.6 to 5,610.0)	4,313.8 (4,006.4 to 4,623.8)	11,449.9 (10,704.5 to 12,174.0)	6,286.9 (5,847.8 to 6,708.2)	5,163.0 (4,805.4 to 5,508.1)
Endocarditis	115.8 (105.6 to 126.4)	96.2 (90.8 to 101.6)	19.6 (12.4 to 26.9)	131.5 (120.2 to 143.1)	108.2 (102.4 to 114.1)	23.3 (15.2 to 30.8)	205.8 (192.8 to 218.6)	132.9 (125.6 to 140.4)	72.9 (65.5 to 80.4)
Hemorrhagic and other stroke	65,025.5 (63,283.4 to 66,946.6)	35,364.1 (34,363.9 to 36,491.5)	29,661.4 (28,757.1 to 30,557.4)	72,339.0 (70,354.3 to 74,354.0)	39,338.3 (38,118.9 to 40,503.1)	33,000.7 (32,082.5 to 33,946.5)	82,247.7 (79,877.5 to 84,630.8)	44,824.1 (43,467.5 to 46,162.4)	37,423.6 (36,298.2 to 38,587.9)
Hypertensive heart disease	19,417.5 (17,735.6 to 21,022.4)	11,424.8 (10,379.5 to 12,378.6)	7,992.7 (7,244.9 to 8,689.4)	23,624.0 (21,603.0 to 25,608.7)	13,971.4 (12,743.1 to 15,204.0)	9,652.6 (8,775.3 to 10,524.9)	29,363.2 (26,900.7 to 31,740.3)	17,250.1 (15,758.6 to 18,665.5)	12,113.1 (11,037.2 to 13,187.6)
Ischemic heart disease	305,840.0 (276,692.6 to 340,165.6)	133,851.6 (121,355.9 to 149,399.8)	171,988.4 (154,994.5 to 192,020.9)	373,480.0 (337,340.8 to 415,897.1)	165,022.8 (149,084.9 to 183,823.1)	208,457.1 (186,686.4 to 232,571.4)	433,378.2 (392,678.6 to 480,934.9)	191,058.4 (172,479.1 to 211,736.5)	242,319.8 (218,848.4 to 270,674.4)
Ischemic stroke	55,052.1 (53,569.6 to 56,587.7)	28,488.1 (27,660.7 to 29,347.4)	26,564.0 (25,837.5 to 27,399.8)	61,344.0 (59,735.2 to 62,997.5)	31,880.7 (30,962.5 to 32,771.4)	29,463.2 (28,652.9 to 30,367.8)	72,044.9 (69,967.0 to 74,245.3)	37,542.3 (36,413.2 to 38,743.7)	34,502.5 (33,492.1 to 35,598.8)
Other cardiovascular and circulatory diseases	430,283.4 (375,111.0 to 489,694.8)	142,638.0 (123,464.5 to 171,115.7)	287,645.4 (244,817.1 to 335,005.7)	474,753.0 (417,033.1 to 541,034.0)	161,744.9 (141,071.4 to 191,578.2)	313,008.1 (270,402.6 to 363,363.1)	563,322.7 (499,909.3 to 635,994.6)	191,559.9 (167,739.8 to 222,981.6)	371,762.8 (321,324.3 to 427,089.7)
Peripheral artery disease	970,851.7 (852,664.0 to 1,111,136.1)	493,212.7 (434,458.7 to 564,970.2)	477,639.0 (417,857.2 to 548,184.6)	1,155,884.9 (1,014,634.0 to 1,321,243.1)	588,235.2 (513,243.7 to 673,929.3)	567,649.6 (496,129.5 to 649,242.3)	1,377,704.3 (1,207,690.9 to 1,583,156.1)	700,187.1 (618,149.0 to 801,656.7)	677,517.2 (591,455.6 to 779,740.3)
**Central Latin America**									
Atrial fibrillation and flutter	366,968.4 (316,660.5 to 424,441.9)	146,638.3 (126,793.3 to 169,872.2)	220,330.1 (189,869.7 to 254,425.1)	441,321.2 (382,121.9 to 512,031.2)	176,453.8 (152,402.3 to 205,662.7)	264,867.4 (228,856.6 to 307,690.7)	530,195.9 (458,596.0 to 613,108.9)	210,074.1 (181,252.0 to 243,719.7)	320,121.9 (276,550.2 to 369,689.0)
Cardiomyopathy and myocarditis	59,854.2 (56,465.7 to 63,124.1)	31,658.5 (29,844.1 to 33,328.7)	28,195.7 (26,453.9 to 30,006.7)	69,675.6 (65,736.4 to 73,533.5)	37,272.4 (35,164.0 to 39,201.6)	32,403.3 (30,445.4 to 34,496.5)	81,739.6 (76,977.5 to 86,246.1)	43,646.6 (41,214.3 to 45,880.8)	38,093.0 (35,582.6 to 40,678.4)
Endocarditis	1,419.9 (1,295.4 to 1,565.9)	955.5 (869.7 to 1,063.6)	464.3 (421.1 to 511.7)	1,642.6 (1,494.6 to 1,813.4)	1,098.0 (999.4 to 1,215.6)	544.6 (493.8 to 599.2)	2,051.7 (1,888.9 to 2,237.6)	1,298.7 (1,185.1 to 1,434.7)	753.0 (697.6 to 815.8)
Hemorrhagic and other stroke	138,887.4 (134,099.3 to 144,290.0)	72,942.8 (70,438.2 to 75,718.5)	65,944.6 (63,661.6 to 68,523.3)	156,146.4 (150,682.6 to 162,167.7)	81,377.1 (78,500.7 to 84,540.7)	74,769.3 (72,205.0 to 77,613.4)	180,268.1 (173,791.6 to 187,228.9)	93,592.2 (90,154.9 to 97,386.7)	86,675.9 (83,669.7 to 90,019.4)
Hypertensive heart disease	135,431.0 (124,769.6 to 145,852.2)	83,228.2 (76,860.5 to 89,394.2)	52,202.8 (47,946.3 to 56,730.8)	167,167.0 (154,256.6 to 179,966.4)	102,559.6 (94,862.0 to 110,098.5)	64,607.4 (59,392.0 to 70,145.9)	209,498.6 (193,853.2 to 224,617.1)	127,051.2 (117,899.1 to 136,115.7)	82,447.5 (75,737.3 to 88,923.2)
Ischemic heart disease	1,886,833.5 (1,719,481.2 to 2,084,833.4)	903,604.5 (823,631.7 to 998,635.0)	983,229.0 (892,994.5 to 1,084,119.7)	2,289,099.4 (2,086,252.9 to 2,520,381.8)	1,105,220.7 (1,009,147.9 to 1,217,790.7)	1,183,878.7 (1,077,578.4 to 1,303,931.0)	2,743,457.6 (2,506,477.3 to 3,022,603.8)	1,314,991.4 (1,201,624.5 to 1,444,890.3)	1,428,466.4 (1,302,989.5 to 1,579,647.7)
Ischemic stroke	147,177.9 (142,580.8 to 152,261.2)	75,019.7 (72,666.9 to 77,636.3)	72,158.2 (69,850.3 to 74,722.7)	164,735.4 (159,219.2 to 170,542.5)	83,901.6 (81,049.0 to 86,906.8)	80,833.8 (78,208.4 to 83,724.6)	194,156.6 (187,314.1 to 201,434.8)	99,011.7 (95,566.4 to 102,898.4)	95,144.9 (91,834.7 to 98,817.1)
Other cardiovascular and circulatory diseases	2,285,575.6 (2,097,811.5 to 2,486,456.8)	1,057,447.2 (967,544.3 to 1,157,648.0)	1,228,128.2 (1,100,380.4 to 1,360,036.7)	2,682,471.5 (2,462,632.1 to 2,907,877.2)	1,266,860.6 (1,158,978.9 to 1,381,090.3)	1,415,611.0 (1,277,136.7 to 1,560,582.2)	3,182,497.3 (2,931,300.6 to 3,437,585.3)	1,522,022.0 (1,404,341.6 to 1,650,234.7)	1,660,475.4 (1,497,897.9 to 1,815,318.7)
Peripheral artery disease	4,031,001.4 (3,549,438.4 to 4,626,165.4)	2,107,155.8 (1,855,744.4 to 2,411,801.7)	1,923,845.5 (1,688,994.1 to 2,214,130.4)	4,893,194.4 (4,311,765.0 to 5,599,466.6)	2,550,857.9 (2,248,874.7 to 2,923,270.4)	2,342,336.5 (2,060,733.4 to 2,693,791.9)	5,908,612.7 (5,203,027.2 to 6,768,663.1)	3,064,958.8 (2,703,342.9 to 3,505,126.5)	2,843,654.1 (2,502,606.9 to 3,275,487.5)
**Tropical Latin America**									
Atrial fibrillation and flutter	372,071.7 (321,556.7 to 432,315.8)	155,883.8 (135,094.1 to 180,662.2)	216,187.8 (185,880.3 to 251,031.8)	453,302.3 (390,567.4 to 524,287.1)	190,147.3 (164,790.8 to 220,176.6)	263,155.0 (226,020.3 to 305,198.2)	548,366.4 (474,093.3 to 635,161.4)	229,666.1 (198,627.2 to 266,418.3)	318,700.2 (275,359.8 to 369,906.2)
Cardiomyopathy and myocarditis	158,988.9 (148,468.3 to 168,807.2)	78,520.3 (74,307.9 to 82,688.6)	80,468.7 (73,877.6 to 86,615.1)	162,979.3 (152,762.0 to 172,383.1)	80,902.6 (76,731.3 to 85,105.5)	82,076.7 (75,908.3 to 87,869.4)	183,249.4 (173,249.7 to 193,698.3)	91,516.6 (86,775.5 to 96,475.7)	91,732.8 (85,785.3 to 97,599.5)
Endocarditis	2,022.3 (1,724.4 to 2,370.8)	933.3 (777.9 to 1,121.6)	1,089.0 (940.2 to 1,264.5)	1,982.0 (1,712.1 to 2,299.6)	926.7 (780.7 to 1,106.4)	1,055.3 (917.1 to 1,218.9)	2,194.5 (1,894.4 to 2,540.8)	1,034.4 (869.5 to 1,223.5)	1,160.1 (1,009.4 to 1,341.3)
Hemorrhagic and other stroke	236,944.2 (227,157.0 to 247,787.0)	122,368.4 (117,437.0 to 128,185.4)	114,575.8 (109,671.0 to 119,873.6)	253,789.3 (242,984.2 to 265,471.5)	130,157.5 (124,549.8 to 136,143.9)	123,631.8 (118,281.6 to 129,508.5)	292,725.0 (279,182.0 to 307,749.3)	149,530.8 (142,630.8 to 157,232.2)	143,194.2 (136,362.3 to 150,569.8)
Hypertensive heart disease	226,919.0 (211,292.0 to 242,290.0)	130,114.9 (121,856.0 to 138,936.0)	96,804.0 (89,777.1 to 103,881.5)	230,400.3 (214,828.2 to 246,079.7)	132,259.2 (123,584.0 to 141,115.9)	98,141.1 (90,640.1 to 105,390.4)	266,990.0 (249,602.3 to 284,581.8)	152,489.7 (142,795.5 to 162,576.4)	114,500.3 (106,381.8 to 122,544.8)
Ischemic heart disease	2,569,163.3 (2,345,124.7 to 2,823,549.0)	1,042,071.3 (959,530.1 to 1,137,623.7)	1,527,091.9 (1,383,728.1 to 1,693,474.8)	2,977,680.0 (2,712,929.2 to 3,279,964.4)	1,212,094.7 (1,115,002.1 to 1,327,640.6)	1,765,585.2 (1,598,476.0 to 1,963,113.9)	3,480,147.6 (3,159,911.8 to 3,841,271.2)	1,414,322.5 (1,296,128.5 to 1,549,720.9)	2,065,824.9 (1,858,217.9 to 2,300,500.2)
Ischemic stroke	281,214.1 (269,469.2 to 295,330.8)	136,505.9 (131,059.1 to 143,172.0)	144,708.1 (138,262.6 to 152,031.6)	299,939.1 (286,747.0 to 314,563.6)	145,570.8 (139,249.1 to 152,802.2)	154,368.2 (147,507.5 to 161,917.7)	356,275.1 (338,561.1 to 375,657.6)	172,946.2 (164,865.8 to 182,222.4)	183,328.9 (173,927.0 to 194,059.0)
Other cardiovascular and circulatory diseases	4,369,284.3 (4,040,177.3 to 4,734,851.9)	1,921,454.5 (1,765,566.8 to 2,101,200.5)	2,447,829.5 (2,212,239.7 to 2,695,528.8)	4,250,655.9 (3,940,270.6 to 4,590,585.6)	1,842,655.6 (1,708,719.1 to 2,014,860.5)	2,408,000.3 (2,184,565.4 to 2,643,663.3)	4,532,284.5 (4,229,040.3 to 4,861,648.0)	1,973,642.8 (1,831,495.3 to 2,137,748.1)	2,558,641.6 (2,344,129.4 to 2,778,704.5)
Peripheral artery disease	5,297,196.3 (4,639,030.2 to 6,095,571.7)	2,854,353.8 (2,507,446.7 to 3,280,069.3)	2,442,842.3 (2,136,763.8 to 2,821,072.1)	6,415,737.7 (5,631,864.9 to 7,361,449.4)	3,469,085.7 (3,050,417.2 to 3,980,787.0)	2,946,651.9 (2,586,923.2 to 3,397,075.1)	7,757,372.9 (6,823,945.1 to 8,885,410.2)	4,193,817.0 (3,695,854.1 to 4,786,453.3)	3,563,555.5 (3,125,839.1 to 4,083,556.7)
**Caribbean**									
Atrial fibrillation and flutter	98,918.7 (85,583.6 to 115,495.1)	38,866.4 (33,494.4 to 45,292.8)	60,052.3 (51,912.3 to 69,737.5)	112,282.6 (97,054.0 to 130,149.3)	44,202.6 (37,880.1 to 51,339.4)	68,080.0 (58,797.9 to 78,933.8)	127,774.8 (110,951.5 to 148,516.8)	50,347.0 (43,078.8 to 58,714.8)	77,427.8 (67,456.6 to 89,634.1)
Cardiomyopathy and myocarditis	18,776.9 (17,743.4 to 19,878.1)	9,632.4 (9,045.4 to 10,190.4)	9,144.5 (8,638.2 to 9,719.5)	20,787.8 (19,632.9 to 22,014.2)	10,722.4 (10,118.2 to 11,381.6)	10,065.4 (9,486.2 to 10,703.0)	23,045.8 (21,676.5 to 24,441.7)	12,027.4 (11,291.8 to 12,807.1)	11,018.4 (10,350.2 to 11,659.0)
Endocarditis	958.0 (908.0 to 1,012.4)	888.3 (840.8 to 942.2)	69.8 (65.7 to 73.9)	1,085.3 (1,028.1 to 1,146.0)	1,011.0 (954.3 to 1,070.1)	74.3 (69.8 to 78.5)	1,230.3 (1,153.5 to 1,312.8)	1,150.7 (1,074.4 to 1,232.1)	79.5 (74.3 to 85.1)
Hemorrhagic and other stroke	96,714.7 (94,458.8 to 99,210.4)	51,501.8 (50,201.7 to 52,801.0)	45,212.9 (44,135.5 to 46,426.4)	104,106.2 (101,608.1 to 106,844.6)	55,241.6 (53,831.4 to 56,738.0)	48,864.6 (47,671.7 to 50,196.0)	114,404.2 (111,412.1 to 117,287.8)	60,893.6 (59,270.9 to 62,555.5)	53,510.6 (52,084.1 to 54,944.5)
Hypertensive heart disease	49,646.1 (45,934.4 to 53,655.6)	25,667.7 (23,713.8 to 27,799.3)	23,978.4 (22,056.0 to 26,007.2)	56,580.3 (52,169.9 to 61,207.6)	29,382.5 (27,116.0 to 31,702.4)	27,197.8 (25,007.2 to 29,488.6)	64,484.5 (59,417.0 to 69,616.9)	33,601.1 (30,906.6 to 36,252.0)	30,883.5 (28,345.5 to 33,345.3)
Ischemic heart disease	499,221.1 (458,865.2 to 546,013.5)	223,257.0 (205,366.8 to 244,538.6)	275,964.1 (252,888.4 to 302,365.1)	571,279.9 (523,755.4 to 625,531.5)	255,462.1 (234,779.3 to 279,983.2)	315,817.8 (288,908.9 to 346,862.4)	649,912.6 (594,603.4 to 712,186.4)	291,675.9 (266,641.2 to 320,462.8)	358,236.7 (326,370.3 to 393,695.2)
Ischemic stroke	95,634.3 (93,310.7 to 97,994.9)	47,840.9 (46,665.2 to 49,046.1)	47,793.4 (46,572.9 to 49,111.0)	104,323.0 (101,853.9 to 107,092.5)	52,176.4 (50,865.4 to 53,545.8)	52,146.6 (50,734.1 to 53,636.2)	117,887.6 (114,874.7 to 121,193.3)	59,348.4 (57,745.9 to 61,088.1)	58,539.2 (56,879.1 to 60,294.1)
Other cardiovascular and circulatory diseases	455,997.9 (421,341.3 to 493,560.1)	176,622.0 (162,105.8 to 193,400.1)	279,375.9 (254,150.5 to 305,989.0)	497,245.2 (460,189.0 to 536,267.0)	194,739.4 (179,710.4 to 212,043.6)	302,505.8 (276,041.4 to 330,773.8)	539,442.9 (501,860.5 to 578,009.9)	214,724.3 (197,842.8 to 232,893.2)	324,718.6 (298,383.0 to 353,387.3)
Peripheral artery disease	1,031,199.9 (905,843.5 to 1,177,977.0)	525,447.5 (460,604.2 to 601,511.9)	505,752.5 (444,032.5 to 581,400.2)	1,172,540.5 (1,032,311.0 to 1,346,864.8)	598,428.4 (527,778.7 to 684,241.9)	574,112.2 (503,472.9 to 662,361.4)	1,347,303.4 (1,185,121.6 to 1,544,357.5)	688,312.5 (605,835.4 to 786,688.2)	658,991.0 (575,949.7 to 759,068.2)
**Oceania**									
Atrial fibrillation and flutter	15,965.5 (13,749.2 to 18,399.5)	6,589.9 (5,659.8 to 7,657.6)	9,375.7 (8,062.6 to 10,789.2)	19,000.1 (16,382.5 to 21,859.8)	7,885.1 (6,735.8 to 9,252.7)	11,115.0 (9,603.1 to 12,775.6)	22,517.0 (19,502.6 to 25,835.0)	9,400.6 (8,070.1 to 10,921.8)	13,116.4 (11,340.9 to 15,029.1)
Cardiomyopathy and myocarditis	1,431.7 (1,352.4 to 1,509.7)	788.4 (742.7 to 835.2)	643.4 (605.2 to 682.3)	1,624.5 (1,533.1 to 1,709.3)	900.2 (849.4 to 951.3)	724.3 (680.3 to 764.9)	1,871.0 (1,762.5 to 1,971.0)	1,047.2 (983.4 to 1,109.2)	823.8 (773.6 to 871.4)
Endocarditis	423.7 (406.0 to 441.8)	312.7 (298.8 to 326.8)	111.0 (106.4 to 115.3)	506.6 (486.4 to 529.2)	374.4 (358.3 to 393.0)	132.2 (126.8 to 137.7)	613.7 (583.7 to 645.2)	455.3 (432.9 to 480.8)	158.3 (150.8 to 166.2)
Hemorrhagic and other stroke	29,494.7 (28,747.6 to 30,200.2)	15,289.5 (14,782.5 to 15,788.5)	14,205.2 (13,876.3 to 14,532.8)	34,143.9 (33,382.8 to 34,973.8)	17,864.6 (17,391.2 to 18,355.3)	16,279.3 (15,895.7 to 16,692.9)	38,961.3 (38,069.9 to 39,939.5)	20,323.9 (19,763.7 to 20,923.0)	18,637.5 (18,175.5 to 19,115.9)
Hypertensive heart disease	1,775.4 (1,636.9 to 1,911.2)	1,027.6 (942.5 to 1,115.5)	747.8 (687.2 to 807.2)	2,115.5 (1,952.1 to 2,281.1)	1,222.9 (1,121.2 to 1,320.6)	892.7 (820.1 to 970.0)	2,508.4 (2,311.7 to 2,708.3)	1,456.3 (1,339.8 to 1,577.6)	1,052.1 (965.5 to 1,141.5)
Ischemic heart disease	43,064.5 (38,927.4 to 47,780.0)	18,686.6 (16,985.3 to 20,748.9)	24,377.9 (21,801.7 to 27,270.7)	51,518.9 (46,415.3 to 57,496.7)	22,445.0 (20,373.3 to 24,912.9)	29,073.9 (26,006.6 to 32,640.2)	62,813.1 (56,704.0 to 69,835.1)	27,472.0 (24,801.8 to 30,652.8)	35,341.1 (31,670.1 to 39,554.0)
Ischemic stroke	24,362.3 (23,754.3 to 24,995.9)	11,920.9 (11,535.9 to 12,338.9)	12,441.4 (12,150.7 to 12,719.9)	28,221.3 (27,556.0 to 28,922.9)	13,961.3 (13,564.7 to 14,371.8)	14,259.9 (13,924.1 to 14,617.6)	32,854.2 (31,946.9 to 33,758.7)	16,172.8 (15,655.7 to 16,721.7)	16,681.4 (16,256.1 to 17,125.4)
Other cardiovascular and circulatory diseases	91,687.6 (80,572.9 to 104,152.8)	30,137.0 (26,783.4 to 35,211.7)	61,550.6 (52,913.8 to 71,310.5)	103,205.6 (91,030.2 to 116,241.1)	34,638.9 (30,845.6 to 40,376.7)	68,566.7 (58,297.5 to 79,378.7)	116,794.9 (103,347.2 to 131,914.4)	40,074.9 (35,677.2 to 45,753.3)	76,720.0 (66,477.8 to 89,295.9)
Peripheral artery disease	70,281.7 (61,545.9 to 81,218.4)	36,275.8 (31,652.6 to 41,936.6)	34,005.9 (29,755.4 to 39,191.5)	84,500.8 (73,755.7 to 97,964.4)	43,593.9 (37,968.9 to 50,397.5)	40,906.9 (35,729.0 to 47,578.3)	102,294.6 (89,375.5 to 119,435.6)	53,205.0 (46,207.4 to 61,536.4)	49,089.6 (42,644.4 to 57,452.7)
**East Asia**									
Atrial fibrillation and flutter	4,805,936.6 (4,170,735.2 to 5,485,250.3)	2,071,077.9 (1,794,756.8 to 2,370,720.9)	2,734,858.7 (2,374,245.5 to 3,112,883.3)	5,624,637.5 (4,893,025.1 to 6,418,811.7)	2,422,748.7 (2,102,869.2 to 2,777,120.5)	3,201,888.5 (2,782,834.8 to 3,655,046.1)	6,558,659.4 (5,710,074.2 to 7,479,918.7)	2,797,110.1 (2,427,089.0 to 3,207,014.7)	3,761,549.3 (3,277,454.0 to 4,292,604.3)
Cardiomyopathy and myocarditis	194,831.6 (186,276.1 to 203,342.5)	102,956.4 (98,150.0 to 107,774.4)	91,875.2 (87,315.5 to 96,388.8)	229,254.2 (219,133.0 to 238,894.2)	125,119.0 (119,107.1 to 131,186.9)	104,135.2 (99,223.1 to 109,128.7)	266,212.1 (254,713.8 to 277,967.7)	149,069.0 (141,761.7 to 156,429.5)	117,143.1 (111,841.9 to 122,329.3)
Endocarditis	4,876.3 (4,345.0 to 5,509.7)	1,966.8 (1,670.2 to 2,301.1)	2,909.5 (2,600.0 to 3,290.8)	5,659.2 (5,030.8 to 6,387.8)	2,378.7 (2,010.8 to 2,799.8)	3,280.5 (2,924.4 to 3,692.3)	6,660.5 (5,867.4 to 7,550.7)	2,934.8 (2,471.9 to 3,458.3)	3,725.7 (3,326.1 to 4,200.9)
Hemorrhagic and other stroke	4,452,415.6 (4,342,195.5 to 4,572,571.6)	1,927,168.4 (1,874,689.9 to 1,984,084.0)	2,525,247.3 (2,466,100.0 to 2,591,208.7)	4,678,369.0 (4,567,474.1 to 4,796,234.2)	1,963,079.0 (1,913,071.8 to 2,018,446.8)	2,715,289.7 (2,651,170.5 to 2,778,272.0)	4,933,113.7 (4,809,786.5 to 5,071,809.4)	2,009,704.7 (1,958,387.4 to 2,067,783.7)	2,923,409.0 (2,850,908.6 to 3,004,010.5)
Hypertensive heart disease	1,214,294.3 (1,153,713.2 to 1,274,661.1)	626,313.0 (595,041.4 to 657,177.4)	587,981.4 (559,146.1 to 619,149.9)	1,497,946.7 (1,425,690.8 to 1,572,357.1)	771,647.1 (733,181.6 to 810,670.9)	726,299.5 (689,997.0 to 761,671.4)	1,818,528.1 (1,729,027.3 to 1,910,199.7)	933,749.7 (885,565.9 to 978,929.1)	884,778.4 (841,961.0 to 930,326.3)
Ischemic heart disease	13,816,610.2 (12,484,326.4 to 15,308,004.1)	5,685,515.4 (5,148,861.9 to 6,288,827.8)	8,131,094.9 (7,332,955.4 to 9,030,417.1)	16,785,482.4 (15,206,674.4 to 18,657,208.7)	6,914,927.4 (6,240,209.5 to 7,672,645.7)	9,870,553.9 (8,912,485.8 to 10,965,990.0)	20,261,325.6 (18,299,731.2 to 22,520,859.3)	8,319,850.4 (7,520,799.6 to 9,229,589.6)	11,941,475.1 (10,714,136.7 to 13,265,552.7)
Ischemic stroke	5,841,031.4 (5,681,730.9 to 6,016,688.0)	2,454,426.6 (2,386,133.4 to 2,529,519.2)	3,386,604.8 (3,296,287.8 to 3,488,476.2)	6,485,359.3 (6,320,699.4 to 6,665,114.7)	2,737,094.7 (2,664,982.6 to 2,815,077.3)	3,748,264.2 (3,653,718.5 to 3,850,736.3)	7,423,861.5 (7,228,512.9 to 7,652,138.9)	3,161,923.9 (3,077,657.2 to 3,259,358.2)	4,261,937.6 (4,146,738.2 to 4,395,441.8)
Other cardiovascular and circulatory diseases	18,326,395.6 (17,106,880.0 to 19,836,795.6)	7,605,213.1 (7,084,527.8 to 8,351,081.9)	10,721,182.6 (9,748,691.4 to 11,837,585.2)	20,755,086.7 (19,508,280.8 to 22,297,946.3)	9,001,225.7 (8,408,912.2 to 9,748,090.3)	11,753,860.0 (10,782,782.0 to 12,867,471.9)	23,784,750.8 (22,398,123.8 to 25,492,655.1)	10,556,750.7 (9,846,590.3 to 11,393,692.7)	13,228,000.1 (12,220,371.1 to 14,389,492.9)
Peripheral artery disease	20,183,125.8 (17,660,454.5 to 23,243,239.3)	10,248,431.4 (8,965,354.2 to 11,796,078.4)	9,934,694.6 (8,636,943.5 to 11,521,934.4)	25,294,489.5 (22,121,028.8 to 29,155,290.0)	12,785,175.8 (11,169,758.6 to 14,684,004.2)	12,509,312.2 (10,893,294.0 to 14,461,465.8)	30,392,782.9 (26,546,376.8 to 34,990,538.8)	15,341,446.3 (13,467,528.6 to 17,627,913.1)	15,051,336.6 (13,113,490.6 to 17,370,492.5)
**Southeast Asia**									
Atrial fibrillation and flutter	1,478,672.7 (1,284,283.2 to 1,701,426.5)	638,840.3 (550,733.6 to 738,828.9)	839,832.5 (731,438.1 to 966,951.8)	1,719,434.8 (1,495,612.5 to 1,981,932.9)	742,305.9 (641,082.0 to 858,773.6)	977,129.0 (849,968.7 to 1,123,578.5)	2,006,789.8 (1,742,901.9 to 2,304,666.5)	861,294.2 (743,889.0 to 992,926.9)	1,145,495.5 (998,502.6 to 1,315,604.4)
Cardiomyopathy and myocarditis	125,056.1 (118,221.9 to 131,643.9)	64,270.4 (60,410.0 to 67,751.7)	60,785.7 (57,394.2 to 63,970.6)	142,956.7 (135,046.3 to 150,766.6)	74,086.7 (69,632.9 to 78,332.6)	68,870.0 (65,006.8 to 72,669.0)	161,979.1 (153,040.5 to 170,746.1)	84,906.1 (79,907.7 to 89,929.9)	77,072.9 (72,744.2 to 81,295.9)
Endocarditis	4,408.2 (4,198.7 to 4,616.5)	2,506.9 (2,370.8 to 2,639.7)	1,901.3 (1,813.0 to 1,991.8)	4,880.0 (4,645.7 to 5,111.3)	2,734.6 (2,586.1 to 2,881.1)	2,145.4 (2,040.4 to 2,254.4)	5,320.8 (5,011.7 to 5,628.0)	2,942.4 (2,743.8 to 3,148.0)	2,378.4 (2,250.6 to 2,502.2)
Hemorrhagic and other stroke	1,624,578.2 (1,589,075.7 to 1,659,310.2)	770,125.7 (751,414.4 to 788,999.9)	854,452.6 (835,902.9 to 873,030.5)	1,818,477.7 (1,779,631.2 to 1,857,095.4)	859,835.4 (840,162.8 to 880,254.3)	958,642.4 (939,137.5 to 978,203.5)	2,040,032.6 (1,995,559.8 to 2,086,665.5)	973,641.7 (950,221.1 to 998,349.7)	1,066,390.8 (1,041,873.1 to 1,090,835.6)
Hypertensive heart disease	270,562.4 (249,214.5 to 290,629.8)	166,272.1 (152,927.2 to 178,961.3)	104,290.3 (95,874.8 to 112,412.7)	318,736.3 (294,224.7 to 342,593.7)	195,464.3 (180,479.7 to 210,623.0)	123,272.0 (113,302.4 to 132,549.0)	372,100.3 (343,679.4 to 400,352.8)	227,936.5 (209,891.0 to 245,294.7)	144,163.7 (133,053.3 to 155,430.5)
Ischemic heart disease	5,368,478.7 (4,747,806.3 to 6,186,346.1)	2,352,196.0 (2,089,493.1 to 2,671,467.3)	3,016,283.0 (2,623,299.0 to 3,517,579.9)	6,306,305.5 (5,506,442.9 to 7,325,382.6)	2,764,047.0 (2,446,630.6 to 3,153,622.8)	3,542,258.9 (3,057,938.2 to 4,195,319.2)	7,471,104.0 (6,551,421.4 to 8,655,835.8)	3,246,054.8 (2,871,795.1 to 3,703,755.3)	4,225,048.9 (3,637,737.3 to 4,990,540.0)
Ischemic stroke	1,654,035.9 (1,617,400.9 to 1,692,091.0)	797,936.7 (777,752.7 to 818,641.2)	856,099.3 (836,729.5 to 875,843.6)	1,846,470.5 (1,805,233.8 to 1,885,997.3)	884,755.1 (863,236.6 to 906,090.0)	961,715.6 (939,662.6 to 982,452.1)	2,106,975.2 (2,059,929.3 to 2,157,906.3)	1,011,731.2 (986,322.8 to 1,038,278.9)	1,095,244.0 (1,068,059.6 to 1,121,514.8)
Other cardiovascular and circulatory diseases	5,971,021.9 (5,518,290.0 to 6,441,357.2)	2,761,066.2 (2,535,543.4 to 2,990,409.9)	3,209,956.0 (2,915,064.4 to 3,526,302.6)	6,565,347.3 (6,101,103.5 to 7,087,324.4)	3,100,264.5 (2,855,351.4 to 3,353,767.9)	3,465,083.1 (3,149,252.8 to 3,800,769.2)	7,388,688.4 (6,853,246.8 to 7,965,349.7)	3,571,993.2 (3,295,706.9 to 3,858,695.0)	3,816,695.0 (3,489,781.0 to 4,165,355.9)
Peripheral artery disease	8,203,530.7 (7,176,491.9 to 9,470,617.7)	4,390,671.5 (3,856,491.9 to 5,059,610.5)	3,812,859.6 (3,319,320.2 to 4,419,563.3)	9,619,800.2 (8,426,012.6 to 11,046,909.2)	5,127,764.0 (4,494,011.4 to 5,895,358.4)	4,492,036.8 (3,925,765.6 to 5,167,536.5)	11,392,507.8 (9,992,404.5 to 13,126,754.2)	6,061,780.0 (5,325,804.0 to 6,955,335.8)	5,330,727.3 (4,654,702.0 to 6,200,469.7)
**South Asia**									

Atrial fibrillation and flutter	2,867,895.7<br>(2,485,293.9 to 3,307,081.6)	1,049,519.9<br>(907,249.5 to 1,220,337.4)	1,818,375.7<br>(1,574,181.7 to 2,094,766.2)	3,334,644.4<br>(2,891,178.1 to 3,848,292.2)	1,224,910.7<br>(1,058,642.3 to 1,428,025.7)	2,109,733.6<br>(1,824,688.3 to 2,434,480.2)	3,899,286.2<br>(3,383,780.9 to 4,502,320.2)	1,437,237.4<br>(1,241,312.7 to 1,676,858.9)	2,462,048.9<br>(2,130,450.0 to 2,842,157.2)
Cardiomyopathy and myocarditis	119,301.2<br>(113,079.3 to 125,761.0)	63,537.2<br>(60,314.8 to 66,848.8)	55,764.0<br>(52,596.1 to 59,038.8)	132,957.5<br>(126,356.0 to 139,758.3)	71,005.2<br>(67,453.1 to 74,632.3)	61,952.3<br>(58,509.3 to 65,515.5)	149,407.8<br>(141,717.6 to 156,673.9)	80,272.2<br>(76,385.6 to 84,302.8)	69,135.6<br>(65,033.4 to 73,054.6)
Endocarditis	8,568.6<br>(7,626.0 to 9,681.0)	4,649.2<br>(4,105.7 to 5,320.8)	3,919.4<br>(3,506.0 to 4,388.0)	9,893.5<br>(8,851.9 to 11,124.5)	5,342.7<br>(4,727.5 to 6,097.1)	4,550.7<br>(4,094.8 to 5,090.5)	11,446.2<br>(10,212.5 to 12,929.9)	6,189.3<br>(5,459.8 to 7,084.7)	5,256.9<br>(4,708.3 to 5,854.6)
Hemorrhagic and other stroke	3,144,590.0<br>(3,070,813.8 to 3,225,303.4)	1,398,839.4<br>(1,363,327.5 to 1,438,080.3)	1,745,750.5<br>(1,707,367.8 to 1,789,906.2)	3,514,672.5<br>(3,432,909.7 to 3,601,953.2)	1,536,360.0<br>(1,497,713.7 to 1,578,291.7)	1,978,312.5<br>(1,934,132.2 to 2,026,071.8)	3,970,982.9<br>(3,865,438.7 to 4,086,074.8)	1,734,618.5<br>(1,687,573.3 to 1,786,687.6)	2,236,364.4<br>(2,178,258.4 to 2,300,243.9)
Hypertensive heart disease	796,210.6<br>(753,980.9 to 840,721.7)	406,825.5<br>(384,751.4 to 428,967.8)	389,385.1<br>(368,856.2 to 411,794.2)	948,262.3<br>(899,322.7 to 997,582.7)	484,930.0<br>(460,372.4 to 511,423.9)	463,332.3<br>(438,091.0 to 488,316.9)	1,140,677.8<br>(1,080,621.1 to 1,201,527.5)	585,749.0<br>(554,736.0 to 616,794.7)	554,928.8<br>(524,813.0 to 585,661.6)
Ischemic heart disease	17,353,210.8<br>(15,704,431.8 to 19,265,629.0)	6,517,477.2<br>(5,883,010.3 to 7,217,242.8)	10,835,732.8<br>(9,790,261.7 to 12,102,663.1)	19,721,230.6<br>(17,776,177.5 to 21,899,698.1)	7,473,657.5<br>(6,745,155.2 to 8,292,088.8)	12,247,572.7<br>(11,007,781.3 to 13,606,617.6)	23,148,017.9<br>(20,930,974.9 to 25,698,774.3)	8,781,643.9<br>(7,924,912.9 to 9,746,862.8)	14,366,374.0<br>(12,946,828.9 to 16,030,805.5)
Ischemic stroke	3,177,846.5<br>(3,100,065.3 to 3,256,822.9)	1,409,337.4<br>(1,372,606.0 to 1,447,231.6)	1,768,508.9<br>(1,724,654.8 to 1,810,810.3)	3,544,016.2<br>(3,462,387.1 to 3,627,913.7)	1,561,069.4<br>(1,521,842.2 to 1,602,157.7)	1,982,946.7<br>(1,938,652.0 to 2,029,096.6)	3,932,150.6<br>(3,831,984.1 to 4,047,703.7)	1,714,307.5<br>(1,667,742.0 to 1,765,752.9)	2,217,843.1<br>(2,160,750.3 to 2,281,985.0)
Other cardiovascular and circulatory diseases	529,083.0<br>(495,202.0 to 563,776.1)	215,092.8<br>(200,929.8 to 229,985.1)	313,990.2<br>(292,848.5 to 335,476.0)	623,819.3<br>(581,782.6 to 666,613.1)	253,337.1<br>(236,825.2 to 270,121.3)	370,482.2<br>(344,927.5 to 397,773.8)	748,799.9<br>(702,412.5 to 795,361.1)	306,804.7<br>(287,469.9 to 326,980.2)	441,995.2<br>(413,903.4 to 471,719.7)
Peripheral artery disease	18,082,303.0<br>(15,782,947.0 to 20,873,335.7)	8,912,317.3<br>(7,786,443.6 to 10,272,816.6)	9,169,984.8<br>(8,009,813.0 to 10,603,451.7)	21,228,968.2<br>(18,551,947.1 to 24,513,155.0)	10,469,307.8<br>(9,148,362.9 to 12,098,060.4)	10,759,660.0<br>(9,403,324.3 to 12,439,110.3)	25,029,463.1<br>(21,873,760.4 to 28,854,783.4)	12,381,089.2<br>(10,814,329.9 to 14,239,217.5)	12,648,374.1<br>(11,042,894.3 to 14,563,975.0)
**North Africa and Middle East**									
Atrial fibrillation and flutter	694,514.1<br>(598,682.7 to 804,930.9)	275,739.3<br>(237,858.0 to 321,938.7)	418,774.8<br>(360,454.7 to 484,319.5)	807,801.1<br>(697,004.2 to 933,620.3)	320,270.6<br>(276,208.3 to 372,742.8)	487,530.5<br>(420,122.9 to 564,818.2)	951,377.2<br>(823,275.9 to 1,100,396.5)	376,268.2<br>(324,735.9 to 440,395.3)	575,109.0<br>(497,317.1 to 667,921.6)
Cardiomyopathy and myocarditis	121,879.5<br>(115,613.3 to 128,344.1)	59,647.6<br>(56,373.6 to 62,906.2)	62,231.9<br>(58,784.7 to 65,691.3)	141,591.0<br>(134,377.3 to 149,033.5)	69,272.6<br>(65,596.5 to 72,920.1)	72,318.4<br>(68,463.3 to 76,399.7)	165,560.6<br>(156,966.1 to 174,151.4)	81,592.8<br>(77,146.8 to 86,021.3)	83,967.8<br>(79,455.7 to 88,527.9)
Endocarditis	12,836.2<br>(12,375.4 to 13,296.9)	11,747.0<br>(11,310.1 to 12,198.2)	1,089.2<br>(1,027.5 to 1,154.4)	15,262.7<br>(14,697.2 to 15,813.6)	14,008.3<br>(13,470.9 to 14,571.7)	1,254.4<br>(1,181.5 to 1,328.6)	18,210.0<br>(17,438.9 to 19,015.2)	16,785.3<br>(16,057.5 to 17,570.6)	1,424.7<br>(1,331.5 to 1,517.9)
Hemorrhagic and other stroke	834,200.9<br>(816,978.4 to 852,721.3)	451,860.3<br>(441,983.8 to 461,893.6)	382,340.7<br>(374,189.3 to 390,953.4)	955,532.5<br>(936,000.6 to 976,284.2)	516,608.0<br>(506,129.1 to 527,968.0)	438,924.4<br>(429,687.7 to 448,149.9)	1,093,535.3<br>(1,069,924.4 to 1,119,497.0)	594,476.8<br>(581,670.6 to 608,840.4)	499,058.6<br>(487,622.8 to 511,343.4)
Hypertensive heart disease	149,029.0<br>(137,760.0 to 160,657.0)	99,358.7<br>(91,787.5 to 107,290.3)	49,670.3<br>(45,660.0 to 53,795.8)	174,175.1<br>(161,168.4 to 187,459.5)	115,951.1<br>(107,503.4 to 124,649.7)	58,224.0<br>(53,568.0 to 62,752.9)	204,350.3<br>(189,297.3 to 219,313.0)	136,376.1<br>(126,365.4 to 146,161.7)	67,974.2<br>(62,733.1 to 73,135.4)
Ischemic heart disease	5,001,171.7<br>(4,444,833.4 to 5,641,558.5)	2,093,691.2<br>(1,861,579.5 to 2,369,545.4)	2,907,480.7<br>(2,587,341.6 to 3,289,801.9)	5,743,751.3<br>(5,109,714.8 to 6,499,957.5)	2,425,416.1<br>(2,151,550.7 to 2,757,770.0)	3,318,335.0<br>(2,954,401.5 to 3,747,380.9)	7,072,468.1<br>(6,265,905.0 to 8,007,139.8)	2,975,936.1<br>(2,637,853.3 to 3,378,580.4)	4,096,532.5<br>(3,629,716.2 to 4,639,946.7)
Ischemic stroke	986,764.0<br>(967,166.3 to 1,007,789.0)	503,699.7<br>(493,325.7 to 514,068.1)	483,064.3<br>(473,054.1 to 494,331.0)	1,126,727.8<br>(1,104,176.5 to 1,149,690.5)	576,911.0<br>(565,543.3 to 588,413.6)	549,816.8<br>(538,238.2 to 561,722.3)	1,312,092.9<br>(1,285,006.8 to 1,341,996.8)	672,113.1<br>(658,117.3 to 688,071.7)	639,979.9<br>(626,007.8 to 654,639.1)
Other cardiovascular and circulatory diseases	10,968,834.1<br>(10,020,898.4 to 12,024,776.4)	4,485,425.1<br>(4,101,011.2 to 5,017,792.3)	6,483,409.4<br>(5,773,359.8 to 7,281,157.3)	11,986,363.4<br>(11,004,588.5 to 13,072,688.0)	5,027,839.8<br>(4,623,467.5 to 5,590,714.3)	6,958,523.2<br>(6,249,661.3 to 7,699,793.3)	13,485,443.3<br>(12,438,127.7 to 14,680,520.7)	5,743,608.7<br>(5,280,613.6 to 6,379,047.7)	7,741,835.4<br>(6,997,326.2 to 8,607,512.8)
Peripheral artery disease	6,237,256.1<br>(5,447,108.0 to 7,179,457.9)	3,090,545.0<br>(2,704,354.6 to 3,555,336.1)	3,146,711.4<br>(2,739,925.8 to 3,635,330.1)	7,301,560.4<br>(6,390,624.9 to 8,381,294.4)	3,632,239.8<br>(3,184,742.4 to 4,184,351.5)	3,669,320.3<br>(3,210,432.9 to 4,233,406.1)	8,643,399.2<br>(7,580,151.2 to 9,950,144.2)	4,315,431.0<br>(3,793,446.1 to 4,951,589.5)	4,327,968.6<br>(3,799,423.9 to 5,002,717.3)
**Central Sub-Saharan Africa**									
Atrial fibrillation and flutter	73,169.3<br>(62,696.9 to 85,449.3)	29,270.7<br>(24,763.6 to 34,379.7)	43,898.6<br>(37,537.3 to 51,292.7)	85,738.5<br>(73,564.2 to 99,812.5)	34,004.4<br>(29,103.9 to 39,642.0)	51,734.2<br>(44,222.0 to 60,350.3)	101,272.7<br>(86,917.9 to 118,263.8)	39,788.8<br>(33,966.1 to 46,910.5)	61,483.9<br>(52,626.4 to 71,773.0)
Cardiomyopathy and myocarditis	28,585.3<br>(26,719.2 to 30,706.2)	13,849.3<br>(12,862.9 to 14,876.6)	14,736.1<br>(13,684.5 to 15,839.6)	33,814.4<br>(31,544.5 to 36,239.5)	16,295.5<br>(15,172.9 to 17,560.4)	17,518.9<br>(16,219.0 to 18,758.8)	39,659.9<br>(37,102.0 to 42,470.2)	19,041.3<br>(17,700.9 to 20,436.5)	20,618.5<br>(19,213.2 to 22,157.3)
Endocarditis	448.1<br>(424.4 to 471.0)	261.3<br>(247.6 to 274.8)	186.9<br>(176.6 to 196.9)	529.4<br>(502.0 to 556.7)	307.4<br>(290.8 to 323.5)	222.0<br>(209.2 to 234.0)	626.8<br>(589.9 to 661.5)	362.7<br>(340.8 to 384.1)	264.1<br>(248.4 to 279.8)
Hemorrhagic and other stroke	130,345.9<br>(127,435.7 to 133,398.1)	75,796.5<br>(74,052.0 to 77,656.7)	54,549.3<br>(53,234.2 to 55,955.3)	153,009.8<br>(149,498.9 to 156,759.0)	88,662.3<br>(86,445.7 to 90,944.2)	64,347.6<br>(62,802.5 to 66,018.4)	181,821.4<br>(177,248.6 to 186,526.4)	105,225.7<br>(102,577.8 to 108,170.6)	76,595.7<br>(74,630.4 to 78,729.5)
Hypertensive heart disease	49,152.0<br>(45,167.3 to 53,286.2)	23,132.0<br>(20,952.7 to 25,332.0)	26,020.0<br>(23,914.7 to 28,143.4)	58,410.1<br>(53,550.4 to 63,051.5)	27,317.8<br>(24,924.8 to 29,846.4)	31,092.4<br>(28,582.3 to 33,541.1)	69,328.1<br>(63,888.5 to 74,939.3)	32,156.2<br>(29,414.9 to 35,035.4)	37,171.9<br>(34,239.9 to 39,962.0)
Ischemic heart disease	190,675.8<br>(175,771.2 to 206,643.2)	85,911.1<br>(79,223.1 to 93,209.9)	104,764.8<br>(96,076.7 to 114,051.4)	225,942.8<br>(208,325.8 to 245,988.7)	101,592.4<br>(93,912.5 to 110,636.6)	124,350.4<br>(113,801.5 to 136,081.9)	262,008.2<br>(242,178.7 to 285,445.7)	116,599.9<br>(107,837.7 to 126,833.3)	145,408.3<br>(133,789.2 to 159,226.5)
Ischemic stroke	136,597.4<br>(133,219.7 to 140,242.1)	78,827.5<br>(76,735.2 to 80,952.8)	57,769.9<br>(56,149.4 to 59,426.1)	157,928.3<br>(153,913.0 to 162,340.4)	90,834.7<br>(88,124.9 to 93,594.7)	67,093.6<br>(65,348.8 to 68,980.3)	187,634.7<br>(182,345.7 to 192,989.3)	107,722.3<br>(104,531.6 to 111,025.3)	79,912.4<br>(77,565.8 to 82,173.6)
Other cardiovascular and circulatory diseases	691,305.4<br>(552,481.6 to 857,552.6)	407,834.5<br>(312,674.9 to 526,898.5)	283,470.9<br>(233,302.4 to 343,052.4)	779,168.9<br>(619,654.5 to 966,777.7)	447,528.0<br>(343,682.3 to 578,246.9)	331,640.9<br>(271,061.0 to 402,741.2)	918,896.7<br>(733,393.8 to 1,129,209.5)	529,925.2<br>(405,586.8 to 683,643.9)	388,971.6<br>(319,793.5 to 466,473.5)
Peripheral artery disease	709,005.9<br>(615,736.5 to 821,559.3)	378,769.9<br>(328,555.2 to 438,274.2)	330,236.0<br>(285,775.8 to 383,632.0)	828,703.7<br>(721,821.7 to 962,762.5)	438,438.7<br>(379,218.1 to 509,156.0)	390,264.9<br>(338,930.2 to 453,550.2)	975,814.1<br>(851,296.4 to 1,130,037.6)	511,584.6<br>(444,969.4 to 588,885.0)	464,229.5<br>(403,966.2 to 540,024.4)
**Eastern Sub-Saharan Africa**									
Atrial fibrillation and flutter	240,802.3<br>(207,967.7 to 280,518.3)	91,997.4<br>(78,574.3 to 108,060.1)	148,805.0<br>(128,441.1 to 173,550.1)	285,241.7<br>(246,883.5 to 331,480.0)	109,998.5<br>(94,023.6 to 128,746.6)	175,243.2<br>(151,971.1 to 203,125.5)	336,685.2<br>(291,703.7 to 391,099.3)	130,899.5<br>(111,828.4 to 152,983.0)	205,785.7<br>(178,727.4 to 239,228.0)
Cardiomyopathy and myocarditis	72,400.8<br>(64,628.0 to 81,321.5)	34,059.4<br>(30,190.7 to 38,493.9)	38,341.4<br>(34,149.7 to 42,971.4)	86,047.3<br>(76,565.7 to 96,793.3)	40,674.9<br>(36,075.1 to 45,722.3)	45,372.3<br>(40,349.2 to 51,139.0)	101,720.5<br>(90,625.4 to 114,363.4)	48,183.8<br>(42,928.6 to 54,261.3)	53,536.7<br>(47,735.0 to 60,512.8)
Endocarditis	1,593.4<br>(1,506.2 to 1,674.5)	886.3<br>(838.2 to 933.4)	707.0<br>(667.8 to 744.0)	1,866.5<br>(1,765.1 to 1,961.2)	1,028.7<br>(973.0 to 1,081.5)	837.7<br>(788.4 to 881.8)	2,244.8<br>(2,111.4 to 2,376.6)	1,231.8<br>(1,157.0 to 1,302.8)	1,013.0<br>(947.8 to 1,077.2)
Hemorrhagic and other stroke	570,891.3<br>(555,321.9 to 587,566.6)	313,407.7<br>(304,929.0 to 322,148.2)	257,483.6<br>(249,915.8 to 265,664.0)	677,133.0<br>(658,529.2 to 696,273.2)	370,544.3<br>(360,452.0 to 380,975.4)	306,588.8<br>(297,547.6 to 315,636.0)	840,949.9<br>(816,965.6 to 866,460.8)	461,124.5<br>(448,039.0 to 475,939.2)	379,825.4<br>(368,337.1 to 392,136.2)
Hypertensive heart disease	132,238.0<br>(120,368.8 to 144,611.0)	58,995.5<br>(52,946.5 to 65,269.8)	73,242.5<br>(67,116.6 to 80,004.6)	159,911.4<br>(144,778.2 to 175,045.5)	71,783.1<br>(63,900.8 to 79,479.0)	88,128.3<br>(80,293.0 to 95,851.4)	188,661.9<br>(171,507.4 to 206,126.0)	85,528.6<br>(76,755.2 to 94,213.2)	103,133.3<br>(94,271.3 to 112,339.0)
Ischemic heart disease	2,309,724.6<br>(2,041,180.2 to 2,624,430.6)	936,495.9<br>(837,206.3 to 1,050,524.0)	1,373,228.5<br>(1,194,363.0 to 1,592,372.8)	2,624,592.6<br>(2,310,036.8 to 2,988,940.9)	1,070,096.4<br>(956,757.1 to 1,202,042.0)	1,554,496.1<br>(1,346,695.7 to 1,801,574.7)	3,098,871.9<br>(2,735,641.9 to 3,532,901.4)	1,264,988.5<br>(1,131,992.1 to 1,434,791.8)	1,833,883.3<br>(1,598,280.7 to 2,126,671.9)
Ischemic stroke	540,659.3<br>(526,220.4 to 554,353.0)	291,211.3<br>(283,482.4 to 298,744.5)	249,447.9<br>(242,487.0 to 256,518.1)	631,435.6<br>(615,471.2 to 648,420.5)	339,716.3<br>(330,855.1 to 349,252.3)	291,719.3<br>(284,093.2 to 299,668.6)	772,966.0<br>(750,966.5 to 795,839.0)	418,378.7<br>(406,070.4 to 431,313.4)	354,587.2<br>(344,927.9 to 364,964.1)
Other cardiovascular and circulatory diseases	2,856,244.2<br>(2,240,415.4 to 3,784,207.2)	1,714,139.0<br>(1,317,502.1 to 2,226,043.6)	1,142,105.1<br>(840,093.9 to 1,625,798.8)	3,409,235.8<br>(2,663,947.9 to 4,551,259.8)	2,065,696.5<br>(1,595,147.1 to 2,676,608.7)	1,343,539.3<br>(985,939.2 to 1,924,695.2)	4,004,455.6<br>(3,153,525.7 to 5,297,800.8)	2,443,583.7<br>(1,904,957.9 to 3,125,429.5)	1,560,871.9<br>(1,145,297.7 to 2,241,668.0)
Peripheral artery disease	2,900,557.1<br>(2,542,261.9 to 3,360,508.8)	1,448,587.0<br>(1,266,140.9 to 1,683,152.1)	1,451,969.9<br>(1,268,727.0 to 1,681,537.9)	3,421,858.2<br>(2,980,085.4 to 3,972,459.9)	1,714,025.9<br>(1,496,183.6 to 1,978,990.9)	1,707,832.2<br>(1,489,402.5 to 1,978,106.7)	4,066,862.3<br>(3,566,058.3 to 4,722,590.6)	2,048,646.3<br>(1,789,802.7 to 2,373,070.7)	2,018,215.9<br>(1,766,118.1 to 2,348,415.8)
**Southern Sub-Saharan Africa**									
Atrial fibrillation and flutter	90,902.5<br>(78,555.7 to 105,114.0)	39,982.6<br>(34,323.6 to 46,586.1)	50,920.0<br>(44,099.0 to 58,940.3)	101,098.8<br>(87,783.9 to 117,263.0)	44,941.8<br>(38,797.7 to 52,466.2)	56,157.0<br>(48,628.8 to 64,896.2)	112,278.0<br>(97,295.9 to 130,165.5)	50,088.0<br>(43,320.8 to 58,126.1)	62,189.9<br>(53,741.7 to 72,206.6)
Cardiomyopathy and myocarditis	52,213.5<br>(50,119.2 to 54,421.7)	26,485.2<br>(25,352.9 to 27,674.0)	25,728.2<br>(24,625.2 to 26,848.0)	58,189.9<br>(55,777.3 to 60,635.5)	29,762.7<br>(28,438.4 to 31,075.0)	28,427.1<br>(27,207.0 to 29,740.0)	64,336.9<br>(61,556.4 to 67,046.3)	32,959.7<br>(31,472.7 to 34,462.9)	31,377.2<br>(29,995.2 to 32,716.6)
Endocarditis	357.1<br>(322.5 to 399.1)	192.0<br>(172.2 to 214.1)	165.1<br>(145.9 to 191.3)	379.5<br>(342.7 to 425.3)	203.3<br>(182.5 to 228.7)	176.1<br>(154.9 to 203.6)	410.6<br>(370.4 to 456.9)	219.7<br>(196.2 to 248.1)	191.0<br>(167.6 to 219.1)
Hemorrhagic and other stroke	164,557.4<br>(160,617.2 to 168,887.1)	84,266.1<br>(82,204.3 to 86,647.1)	80,291.3<br>(78,319.5 to 82,460.0)	175,718.6<br>(171,376.5 to 180,109.4)	89,954.0<br>(87,628.6 to 92,614.0)	85,764.6<br>(83,718.4 to 87,981.0)	186,862.8<br>(182,131.5 to 192,225.2)	95,225.6<br>(92,740.9 to 98,041.2)	91,637.3<br>(89,261.8 to 94,206.0)
Hypertensive heart disease	43,277.0<br>(40,474.9 to 46,035.6)	21,541.5<br>(20,143.3 to 22,988.7)	21,735.5<br>(20,400.7 to 23,215.0)	47,874.4<br>(44,897.7 to 50,927.2)	24,052.9<br>(22,525.8 to 25,602.7)	23,821.4<br>(22,315.2 to 25,418.9)	52,813.0<br>(49,594.8 to 56,090.7)	26,629.6<br>(24,988.4 to 28,348.1)	26,183.5<br>(24,494.4 to 27,895.5)
Ischemic heart disease	544,309.5<br>(486,877.4 to 608,979.7)	236,927.3<br>(213,472.3 to 264,572.7)	307,382.2<br>(272,912.5 to 346,897.5)	595,641.3<br>(532,594.8 to 669,043.8)	259,691.2<br>(233,362.8 to 290,636.3)	335,950.1<br>(297,919.3 to 380,965.9)	653,588.8<br>(584,539.0 to 736,283.5)	283,991.9<br>(255,828.1 to 318,332.4)	369,596.9<br>(328,378.4 to 419,248.5)
Ischemic stroke	167,599.5<br>(163,492.7 to 172,073.9)	90,413.2<br>(88,038.2 to 93,062.8)	77,186.3<br>(75,383.7 to 79,215.2)	178,011.2<br>(173,592.9 to 182,530.8)	96,539.7<br>(93,884.3 to 99,580.9)	81,471.5<br>(79,509.1 to 83,444.0)	190,017.6<br>(185,419.5 to 195,149.6)	103,089.9<br>(100,210.9 to 106,286.6)	86,927.7<br>(84,802.9 to 89,121.8)
Other cardiovascular and circulatory diseases	743,625.1<br>(710,751.0 to 776,914.3)	504,584.9<br>(481,554.6 to 529,235.3)	239,040.2<br>(223,971.8 to 254,207.6)	846,070.2<br>(807,617.6 to 885,247.1)	583,191.4<br>(554,295.8 to 612,054.2)	262,878.8<br>(246,637.7 to 278,714.7)	950,396.8<br>(911,338.1 to 990,804.2)	659,335.9<br>(631,186.6 to 688,882.3)	291,060.9<br>(274,617.3 to 308,267.3)
Peripheral artery disease	1,434,678.4<br>(1,261,514.6 to 1,649,825.4)	802,579.7<br>(705,286.5 to 921,552.1)	632,098.8<br>(554,977.1 to 728,347.4)	1,556,363.3<br>(1,370,061.6 to 1,775,239.8)	874,154.3<br>(770,555.1 to 996,441.6)	682,208.9<br>(596,663.5 to 783,047.9)	1,741,552.2<br>(1,530,173.8 to 1,991,968.6)	979,344.0<br>(859,597.6 to 1,119,069.1)	762,208.2<br>(667,487.9 to 881,147.7)
**Western Sub-Saharan Africa**									
Atrial fibrillation and flutter	262,627.6<br>(225,757.8 to 306,254.0)	99,121.6<br>(83,971.4 to 116,301.6)	163,506.0<br>(140,475.0 to 189,605.5)	300,331.8<br>(258,129.7 to 350,429.3)	113,848.9<br>(97,259.7 to 133,299.4)	186,482.9<br>(159,800.2 to 217,007.0)	344,318.5<br>(294,606.3 to 400,860.4)	130,181.7<br>(110,429.6 to 152,220.9)	214,136.7<br>(183,338.6 to 248,581.6)
Cardiomyopathy and myocarditis	111,890.2<br>(104,875.5 to 119,003.4)	48,719.1<br>(45,639.5 to 51,735.4)	63,171.1<br>(59,068.5 to 67,487.1)	131,072.4<br>(123,104.7 to 139,016.7)	57,225.8<br>(53,513.3 to 60,849.5)	73,846.6<br>(69,137.5 to 78,477.2)	155,430.8<br>(145,996.9 to 165,219.6)	67,340.2<br>(63,214.3 to 71,677.1)	88,090.6<br>(82,488.8 to 94,200.2)
Endocarditis	1,971.7<br>(1,871.5 to 2,073.9)	1,093.4<br>(1,034.9 to 1,151.2)	878.3<br>(832.9 to 924.7)	2,248.4<br>(2,132.5 to 2,359.6)	1,238.2<br>(1,169.1 to 1,302.8)	1,010.2<br>(959.9 to 1,060.5)	2,617.8<br>(2,467.1 to 2,774.7)	1,438.4<br>(1,349.3 to 1,528.4)	1,179.4<br>(1,112.7 to 1,247.7)
Hemorrhagic and other stroke	553,146.6<br>(541,102.6 to 566,351.2)	275,795.7<br>(269,742.1 to 282,433.6)	277,350.8<br>(270,832.6 to 284,659.0)	632,388.6<br>(618,396.3 to 647,630.1)	315,236.2<br>(308,191.9 to 322,926.0)	317,152.4<br>(309,493.0 to 324,827.4)	727,043.2<br>(709,295.5 to 746,946.6)	360,682.9<br>(352,132.6 to 370,683.4)	366,360.3<br>(356,968.7 to 377,288.4)
Hypertensive heart disease	177,041.9<br>(165,077.1 to 188,576.9)	75,066.7<br>(69,473.9 to 80,826.9)	101,975.2<br>(95,156.5 to 108,637.1)	204,536.9<br>(190,238.9 to 218,385.4)	86,011.4<br>(79,316.5 to 92,699.0)	118,525.5<br>(110,280.9 to 126,170.9)	239,069.1<br>(223,086.2 to 256,080.1)	100,724.9<br>(92,968.7 to 108,471.6)	138,344.1<br>(129,158.8 to 147,690.4)
Ischemic heart disease	1,759,160.2<br>(1,557,462.5 to 2,009,086.0)	593,110.9<br>(530,775.2 to 666,953.0)	1,166,049.2<br>(1,017,799.2 to 1,345,015.8)	1,991,798.2<br>(1,751,919.8 to 2,269,345.1)	678,731.3<br>(607,845.4 to 761,154.8)	1,313,066.9<br>(1,139,879.8 to 1,522,300.6)	2,345,370.4<br>(2,057,647.1 to 2,677,442.3)	791,437.3<br>(707,199.5 to 894,196.7)	1,553,933.2<br>(1,341,625.5 to 1,795,108.4)
Ischemic stroke	482,250.8<br>(472,033.9 to 493,624.3)	250,298.9<br>(244,729.1 to 256,366.6)	231,951.9<br>(227,173.1 to 237,279.7)	541,595.5<br>(530,030.3 to 553,963.5)	280,471.0<br>(274,111.8 to 287,197.0)	261,124.4<br>(255,797.7 to 266,997.0)	617,935.1<br>(603,711.5 to 633,754.9)	318,735.2<br>(310,971.2 to 327,447.7)	299,199.9<br>(292,051.3 to 306,699.6)
Other cardiovascular and circulatory diseases	6,286,117.6<br>(5,818,187.1 to 6,759,908.9)	3,712,343.5<br>(3,419,140.3 to 3,993,879.9)	2,573,773.7<br>(2,308,473.4 to 2,869,973.7)	7,205,835.2<br>(6,664,334.6 to 7,753,217.7)	4,210,972.1<br>(3,882,933.7 to 4,533,349.1)	2,994,863.0<br>(2,695,807.6 to 3,333,016.5)	8,353,382.8<br>(7,740,986.6 to 9,002,357.2)	4,857,870.5<br>(4,465,095.7 to 5,239,879.8)	3,495,512.4<br>(3,145,095.2 to 3,902,412.8)
Peripheral artery disease	3,120,617.7<br>(2,714,951.7 to 3,635,917.1)	1,511,646.2<br>(1,312,914.1 to 1,758,370.0)	1,608,971.3<br>(1,397,465.7 to 1,876,792.3)	3,584,673.8<br>(3,113,912.5 to 4,150,614.7)	1,745,003.6<br>(1,522,745.9 to 2,021,947.6)	1,839,670.2<br>(1,599,954.7 to 2,143,217.9)	4,129,936.2<br>(3,595,007.5 to 4,805,465.9)	2,000,384.7<br>(1,742,584.9 to 2,329,973.2)	2,129,551.5<br>(1,850,451.9 to 2,487,118.3)

eTable 4a. All-ages prevalence per 100,000 persons for men and women separately and combined, for all CVD level 3 and 4 subcauses, by country in 1990 and 2015									
	1990			2015			Median percent change between 1990-2015		
	Both	Female	Male	Both	Female	Male	Both	Female	Male
**Atrial fibrillation and flutter**									
Afghanistan	76.58	60.87	91.53	87.13	69.81	103.4	0.14	0.15	0.14
Albania	404.16	344.46	461.01	815.85	706.67	926.78	1.00	1.03	1.00
Algeria	110.6	91.17	129.56	202.32	157.47	246.55	0.80	0.70	0.87
American Samoa	178.91	139.72	216	185.03	163.16	206.79	0.03	0.16	-0.05
Andorra	1006.21	925.25	1091.58	1356.83	1196.42	1523.97	0.34	0.29	0.38
Angola	77.25	60.9	94.01	74.54	58.38	90.92	-0.03	-0.03	-0.02
Antigua and Barbuda	228.3	192.44	266.65	240.38	193.09	291.95	0.05	0.00	0.09
Argentina	449.06	304.51	599.31	544.24	399.68	695.08	0.21	0.31	0.16
Armenia	437.15	396.86	479.85	738.39	676.01	810.25	0.66	0.68	0.66
Australia	545.43	400.25	691.4	693.23	479.38	907.63	0.26	0.19	0.30
Austria	986.11	979.87	992.91	1889.25	1679.78	2109.12	0.90	0.71	1.09
Azerbaijan	336.5	312.18	361.88	495.08	424.14	566.85	0.46	0.35	0.55
Bahrain	93.47	67.67	112.45	123.21	97.39	139.02	0.31	0.43	0.23
Bangladesh	135.74	94.41	175.24	210.31	149.49	269.95	0.56	0.59	0.56
Barbados	278.27	246.07	312.96	411.91	336.3	494.09	0.47	0.36	0.56
Belarus	670.1	677.81	661.36	876.06	880.6	870.8	0.30	0.29	0.30
Belgium	692.38	650.2	736.51	914.71	828.68	1004.14	0.31	0.27	0.34
Belize	128.59	96.81	159.84	135.91	96.83	175.22	0.06	0.00	0.11
Benin	93.99	79.23	109.69	89.35	72.43	106.33	-0.04	-0.08	-0.02
Bermuda	206.88	157.54	256.85	287.69	224.9	351.69	0.38	0.42	0.36
Bhutan	138.24	95.42	178.03	220.07	144.51	285.12	0.57	0.50	0.58
Bolivia	133.73	103.59	163.66	200.13	153.95	246.14	0.47	0.46	0.48
Bosnia and Herzegovina	510.88	478.38	543.42	1004.38	929.64	1079.82	0.94	0.92	0.96
Botswana	78.18	67.09	89.53	115	93.04	136.98	0.46	0.38	0.52
Brazil	134.86	111.89	158.2	257.63	212.89	303.86	0.89	0.89	0.90
Brunei	107.7	35.9	171.97	187.54	67.34	300.64	0.69	0.81	0.70
Bulgaria	843.54	762.35	927.01	1181.99	1153.29	1212.11	0.39	0.50	0.30
Burkina Faso	90.57	74.88	106.99	74.02	64.19	83.97	-0.18	-0.14	-0.21
Burundi	84.18	67.51	101.44	80.41	59.89	101.37	-0.01	-0.09	0.04
Cambodia	150.64	131.41	171.25	213.5	193.34	234.66	0.45	0.49	0.41
Cameroon	102.61	79.83	125.68	95.37	73.72	117.01	-0.05	-0.07	-0.05
Canada	757.15	457.86	1061.58	1046.97	592.48	1507.84	0.37	0.29	0.40
Cape Verde	122.34	99.03	148.12	148.08	125.92	170.83	0.20	0.26	0.15
Central African Republic	115.56	90.06	141.93	111.17	88.41	134.54	-0.04	-0.02	-0.05
Chad	94.41	73.95	115.16	76.89	59.57	94.17	-0.18	-0.19	-0.18
Chile	344.81	227.65	465.14	578.19	402.35	758.73	0.65	0.74	0.60
China	246.31	219.92	271.34	455.43	399.1	508.49	0.89	0.84	0.92
Colombia	129.53	104.27	155.11	225.91	179.99	273.25	0.73	0.71	0.74
Comoros	86.89	69.2	104.46	89.85	69.61	109.73	0.04	0.01	0.05
Congo	107.3	82.45	132.28	108.46	83.86	133.02	0.01	0.02	0.01
Costa Rica	152.22	117.35	186.53	275.46	211.37	339.47	0.79	0.78	0.80
Cote d'Ivoire	92.93	62.08	121.34	96.66	62.55	129.6	0.05	0.01	0.08
Croatia	599.99	566.02	636.09	741.13	668.43	819.17	0.23	0.17	0.28
Cuba	266.05	202.05	329.09	425.19	329.79	519.69	0.58	0.62	0.56
Cyprus	548.7	464.76	634.78	700.5	616.71	790.06	0.25	0.30	0.21
Czech Republic	1074.39	1053.16	1096.9	1690.68	1527.54	1859.06	0.56	0.44	0.68
Democratic Republic of the Congo	87.97	72.3	104.04	88.51	69.41	107.71	0.01	-0.04	0.04
Denmark	717.64	632.41	805.35	1016.29	850.15	1185.08	0.41	0.34	0.46
Djibouti	81.55	63.41	99.47	121.59	91.85	151.07	0.48	0.44	0.51
Dominica	196.46	149.41	243.86	279.93	218.56	342.28	0.43	0.47	0.42
Dominican Republic	129.39	90.68	167.59	215.82	161.75	270.25	0.66	0.77	0.61
Ecuador	135.76	104.03	167.13	214.06	165.56	262.58	0.58	0.59	0.58
Egypt	147.12	128.1	166.01	168.43	142.44	193.89	0.14	0.11	0.17
El Salvador	135.18	105.37	166.4	242.79	192.11	300.08	0.80	0.82	0.82
Equatorial Guinea	122.37	96.08	147.53	111.74	76.75	145.02	-0.09	-0.20	-0.02
Eritrea	57.43	48.62	66.35	76.2	64.33	88.02	0.33	0.33	0.34
Estonia	703.63	719.39	685.7	982.29	1004.1	956.68	0.38	0.38	0.38

Ethiopia	93.32	66.66	120.14	98.52	74.85	122.28	0.06	0.13	0.03
Federated States of Micronesia	168.63	137.71	198.23	220.82	187.9	252.17	0.30	0.36	0.26
Fiji	164.62	129.7	198.46	293.56	245.91	339.62	0.77	0.88	0.70
Finland	1227.86	1187.79	1270.38	1873.95	1732.58	2019.66	0.51	0.45	0.56
France	971.4	893.79	1053.23	1275.44	1140.66	1418.93	0.31	0.27	0.34
Gabon	160.93	129.42	193.76	145.59	115.09	175.4	-0.10	-0.11	-0.10
Georgia	624.23	606.34	643.96	852.02	820.34	886.67	0.36	0.35	0.37
Germany	1042.33	1010.63	1076.39	1481.67	1297.52	1672.06	0.41	0.28	0.53
Ghana	86.64	65.76	106.85	104.58	83.02	126.35	0.22	0.27	0.20
Greece	863.52	714.55	1016.3	1295.17	1144.78	1453	0.49	0.59	0.41
Greenland	297.86	173.24	405.71	569.18	279.4	820.42	0.88	0.60	0.97
Grenada	208.96	182.02	236.61	223.59	191.63	255.34	0.09	0.06	0.10
Guam	212.77	175.47	245.34	408.11	343.98	470.45	0.94	0.97	0.95
Guatemala	109.16	80.74	137.99	149.99	117.91	183.47	0.40	0.48	0.36
Guinea	101.77	78.73	125.32	96.62	73.51	119.62	-0.05	-0.07	-0.04
Guinea-Bissau	107.01	84	130.57	98.39	75.3	121.77	-0.07	-0.10	-0.05
Guyana	125	93.91	157.09	175.03	134.63	215.04	0.41	0.43	0.38
Haiti	125.85	96.4	156.18	148.44	116.97	180.59	0.21	0.23	0.20
Honduras	107.97	82.45	133.3	159.91	121.38	198.44	0.49	0.47	0.50
Hungary	833.14	816.34	851.31	1055.94	1062.3	1048.93	0.26	0.29	0.23
Iceland	1198.23	894.12	1499.67	1476.96	1082.11	1880.26	0.22	0.20	0.23
India	168.38	125.58	208.24	239.84	185.5	290.34	0.43	0.48	0.40
Indonesia	196.44	163.62	228.97	276.66	229.49	323.15	0.43	0.42	0.44
Iran	111.32	81.75	139.81	187.92	135.92	239.15	0.66	0.63	0.69
Iraq	119.74	96.55	142.51	108.75	91.48	125.6	-0.09	-0.05	-0.12
Ireland	729.83	576.66	884.35	836.4	622.66	1056.48	0.13	0.07	0.18
Israel	633.97	521.61	746.75	808.13	688.34	929.95	0.27	0.31	0.24
Italy	933.16	826.52	1046.31	1293.18	1126.49	1470.41	0.37	0.36	0.39
Jamaica	213.8	168.87	259.9	280.78	213.98	348.11	0.30	0.25	0.32
Japan	356.24	195.25	523.02	703.03	423.53	997.38	0.93	1.14	0.85
Jordan	111.37	85.76	134.54	131.37	102.83	158.53	0.16	0.19	0.16
Kazakhstan	414.3	419.53	408.74	499.34	473.32	527.22	0.21	0.13	0.29
Kenya	80.97	59.15	102.94	88.5	68.38	108.62	0.10	0.16	0.06
Kiribati	174.84	153.36	196.77	200	180.35	220.16	0.15	0.18	0.12
Kuwait	82.09	48.72	111.27	108.11	73.43	135.18	0.30	0.49	0.20
Kyrgyzstan	354.46	347.84	361.36	357.21	314.43	400.91	0.01	-0.10	0.11
Laos	172.02	148.64	195.43	194.68	165.87	223.77	0.15	0.13	0.17
Latvia	721.96	736.73	704.94	964.66	989.98	935.17	0.32	0.33	0.31
Lebanon	186.28	143.28	230.47	261.16	197.25	324.41	0.39	0.36	0.39
Lesotho	114.35	95.15	134.7	112.91	102.35	123.68	-0.02	0.07	-0.09
Liberia	89.18	66.31	112.18	94.26	72.97	115.2	0.06	0.10	0.03
Libya	107.31	82.6	129.44	162.23	130.15	193.92	0.50	0.56	0.49
Lithuania	664.49	652.24	678.13	959.31	969.13	947.89	0.43	0.47	0.38
Luxembourg	982.59	953.46	1012.91	1089.81	1021.11	1158.79	0.11	0.07	0.14
Macedonia	530.43	451.14	608.79	833.74	756.19	911.98	0.55	0.66	0.48
Madagascar	91.78	62.42	121.22	90.77	68.91	112.76	0.00	0.12	-0.06
Malawi	78.13	61.52	95.32	88.55	71.46	105.71	0.15	0.18	0.13
Malaysia	189.98	151.03	227.78	299.27	230.11	369.71	0.57	0.52	0.62
Maldives	158.77	98.52	216	251.94	188.1	315.42	0.58	0.90	0.46
Mali	103.78	79.66	128.43	77.85	62.13	93.25	-0.24	-0.21	-0.26
Malta	614.27	551.39	679.39	990.94	944	1038.26	0.58	0.69	0.49
Marshall Islands	144.49	119.72	168.18	201.72	171.89	230.53	0.39	0.43	0.37
Mauritania	91.34	72.87	109.93	100.31	80.64	119.71	0.11	0.11	0.10
Mauritius	226.8	206.98	246.71	447.49	389.52	506.9	0.95	0.87	1.02
Mexico	136.2	109.21	163.48	214.8	167.42	262.66	0.57	0.52	0.59
Moldova	531.53	497.83	568.5	697.73	647.71	751.81	0.29	0.28	0.30
Mongolia	285.61	238.42	333.92	340.9	284.35	398.6	0.18	0.18	0.18
Montenegro	588.7	559.4	618.27	896.85	829.71	965.6	0.51	0.47	0.54
Morocco	125.59	104.18	147.09	212.15	169.39	255.9	0.67	0.61	0.72
Mozambique	88.54	71.8	106.8	91.96	75.73	108.95	0.05	0.06	0.03
Myanmar	202.62	172.67	233.79	281.84	240.44	325.14	0.42	0.42	0.43

Namibia	96.28	78.41	114.93	106.53	89.94	124	0.11	0.15	0.08
Nepal	143.42	119.79	167.4	214.67	159.96	272.85	0.51	0.34	0.65
Netherlands	1282.12	1167.06	1399.8	1730.14	1435.51	2029.69	0.34	0.23	0.43
New Zealand	1186.76	1104.46	1271.58	1548.99	1353.73	1751.72	0.29	0.21	0.35
Nicaragua	100.09	81.49	118.98	171.33	137.84	205.78	0.73	0.70	0.75
Niger	75.33	57.92	93.32	82.13	59.49	104.4	0.10	0.04	0.14
Nigeria	89.24	68.87	109.21	86.34	65.11	106.77	-0.03	-0.05	-0.02
North Korea	235.21	248.05	221.61	420.28	424.52	415.85	0.79	0.72	0.89
Northern Mariana Islands	135.26	94.6	171.81	135.05	88.53	181.9	0.01	-0.06	0.08
Norway	1533.67	1297.47	1775.21	2011.62	1521.28	2496.27	0.30	0.17	0.39
Oman	86.34	75.28	95.16	117.38	92.36	130.07	0.35	0.22	0.36
Pakistan	164.06	108.83	215.32	186.17	132.82	236.67	0.14	0.22	0.10
Palestine	76.42	62.69	89.81	101.66	81.61	121.15	0.32	0.29	0.33
Panama	151.32	113.08	188.72	242.93	184.93	300.6	0.63	0.66	0.63
Papua New Guinea	134.55	115.38	152.85	170.46	148.48	191.58	0.27	0.29	0.26
Paraguay	129.5	101.49	156.88	193.82	146.07	240.14	0.48	0.43	0.51
Peru	128.62	99.91	157.34	215.73	170.77	260.75	0.68	0.71	0.66
Philippines	160.14	139.08	180.75	236.83	206.1	267	0.49	0.49	0.49
Poland	862.11	851.62	873.14	1471.87	1450.92	1494.42	0.69	0.69	0.70
Portugal	937.33	820.05	1063.46	1304.09	1163.9	1453.58	0.37	0.40	0.34
Puerto Rico	283.19	216.37	354.23	409.93	339.87	485.62	0.44	0.56	0.36
Qatar	85.98	51.92	102.79	92.87	61.62	104.56	0.07	0.17	0.01
Romania	520.83	504.38	537.75	688.73	703.38	673.13	0.31	0.38	0.24
Russia	642.25	671.88	608.61	860.25	860.57	859.87	0.33	0.27	0.40
Rwanda	69.05	54.45	84.15	84.65	71.75	98.68	0.23	0.32	0.18
Saint Lucia	203.62	159.14	249.43	281.27	220.65	344.21	0.40	0.40	0.41
Saint Vincent and the Grenadines	175.32	145.6	205.34	242.33	187.95	295.71	0.39	0.29	0.45
Samoa	178.94	153.06	202.78	259.15	229.34	287.14	0.44	0.49	0.41
Sao Tome and Principe	126.61	97.86	155.9	99.68	80.27	119.24	-0.21	-0.18	-0.23
Saudi Arabia	97.2	78.9	111.6	136.37	100.6	163.85	0.38	0.25	0.44
Senegal	89.32	68.92	109.92	88.24	72.36	104.7	-0.01	0.05	-0.04
Serbia	450.82	412.92	489.74	598.01	576.84	620.21	0.31	0.38	0.25
Seychelles	287.42	265.74	309.19	374.25	334.48	412.97	0.30	0.25	0.33
Sierra Leone	96.61	67.94	125.87	84.24	61.59	107.35	-0.11	-0.08	-0.12
Singapore	200.38	64.39	332.82	383.62	137.97	638.03	0.87	1.11	0.87
Slovakia	896.77	878.27	916.13	1542.77	1533.52	1552.53	0.70	0.73	0.67
Slovenia	759.7	750.85	769.09	1250.58	1162.73	1340.01	0.62	0.53	0.71
Solomon Islands	148.18	106.9	187.26	178.69	138.76	217.39	0.21	0.30	0.16
Somalia	91.88	70.31	113.83	82.84	63.84	102.02	-0.10	-0.09	-0.10
South Africa	130.6	111.74	149.91	166.51	149.09	184.54	0.27	0.33	0.23
South Korea	167.99	89.84	245.79	416.03	218.83	615.63	1.40	1.37	1.42
South Sudan	84.63	63.81	105.64	98.16	75.02	121.22	0.16	0.18	0.15
Spain	1096.67	1020.51	1176	1474.24	1342.46	1609.43	0.33	0.31	0.35
Sri Lanka	263.88	202.9	323.68	416.07	350.2	486.88	0.60	0.74	0.53
Sudan	99.37	79.45	119.13	113.78	90.82	136.56	0.15	0.15	0.15
Suriname	152.49	120.49	183.47	221.16	182.43	259.74	0.45	0.51	0.41
Swaziland	80.6	64.51	98.59	101.01	84.75	117.62	0.25	0.31	0.19
Sweden	1370.22	1073.75	1673.89	1941.62	1564.05	2319.94	0.40	0.45	0.36
Switzerland	645.03	588.18	704.57	686.33	598.71	775.93	0.06	0.01	0.09
Syria	98.94	76.42	120.75	140.75	115.4	165.48	0.41	0.50	0.36
Taiwan	332.96	268.11	393.16	627.9	530.1	726.03	0.93	1.00	0.91
Tajikistan	280.88	252.08	310.02	274.08	208.67	337.97	-0.03	-0.17	0.09
Tanzania	81.13	63.36	99.22	89.51	66.44	112.87	0.12	0.06	0.16
Thailand	233.13	195.84	271.1	503.27	419.88	589.06	1.16	1.14	1.17
The Bahamas	138.71	116.7	161.03	259.72	215.39	305.96	0.85	0.82	0.88
The Gambia	81.37	53.85	108.51	76.87	53.99	100.2	-0.05	0.00	-0.07
Timor-Leste	115.73	94.86	136.08	216.95	177.2	255.48	0.89	0.89	0.89
Togo	88.48	69.72	107.72	86.27	67.61	105.38	-0.02	-0.02	-0.01
Tonga	208.12	176.55	238.91	272.91	248.4	297.29	0.31	0.40	0.24
Trinidad and Tobago	173.74	140.84	206.88	287.35	233.31	342.83	0.64	0.64	0.64
Tunisia	155.21	120.04	189.95	255.66	208.04	304.39	0.62	0.71	0.58

Turkey	179.81	149.12	211.26	248.36	214.53	283.39	0.37	0.43	0.33
Turkmenistan	278.83	261.72	296.41	350.19	300.98	401.08	0.25	0.14	0.34
Uganda	78.85	60.19	97.75	71.56	56.64	86.5	-0.08	-0.05	-0.10
Ukraine	737.47	753.23	719.13	948.01	944.29	952.41	0.28	0.25	0.32
United Arab Emirates	74.04	48.3	87.41	105.6	53.36	124.64	0.41	0.09	0.41
United Kingdom	1105.86	983.88	1234.88	1344.46	1138.67	1555.39	0.21	0.15	0.25
United States	1430.44	1338.87	1526.75	1832.69	1624.88	2047.53	0.27	0.21	0.32
Uruguay	565.6	391.37	750.57	699.85	539.07	872.08	0.23	0.37	0.15
Uzbekistan	289.59	271.08	308.5	371.71	320.76	424.34	0.27	0.17	0.37
Vanuatu	181.98	125.29	235.58	216.63	171.81	260.34	0.15	0.34	0.06
Venezuela	121.53	94.5	148.14	201.49	160.8	242.52	0.64	0.69	0.61
Vietnam	246.8	230.81	263.28	346.83	326.44	367.66	0.44	0.44	0.44
Virgin Islands, U.S.	187.35	146.45	230.67	469.8	365.39	584.56	1.48	1.47	1.50
Yemen	81.93	72.46	91.57	96.68	77.22	115.75	0.18	0.06	0.26
Zambia	79.15	61.25	97.36	80.66	64.5	96.88	0.03	0.06	0.01
Zimbabwe	87.97	67.24	108.88	89.29	69.73	109.43	0.02	0.04	0.01
**Cardiomyopathy and myocarditis**									
Afghanistan	11.57	10.88	12.23	11.85	11.06	12.59	0.03	0.02	0.04
Albania	30.09	29.98	30.19	56.77	60.43	53.05	0.87	1.00	0.75
Algeria	32.5	31.33	33.64	50.95	50.23	51.67	0.54	0.58	0.51
American Samoa	32.55	37.83	27.54	33.51	39.28	27.76	0.03	0.04	0.00
Andorra	48.28	55.2	40.98	56.14	67.14	44.68	0.15	0.21	0.08
Angola	31.49	30.27	32.73	32.42	30.92	33.94	0.04	0.03	0.05
Antigua and Barbuda	51.69	53.68	49.57	58.96	59.02	58.89	0.14	0.09	0.18
Argentina	36.7	47.8	25.16	47.51	64.66	29.62	0.29	0.35	0.18
Armenia	9.12	7.76	10.55	12.15	10.86	13.65	0.31	0.38	0.27
Australia	68.01	71.74	64.26	86.51	95.05	77.95	0.26	0.32	0.20
Austria	62.67	74.53	49.74	71.53	83.38	59.09	0.13	0.11	0.17
Azerbaijan	10.52	9.12	11.99	14.1	12.13	16.11	0.33	0.32	0.33
Bahrain	27.74	27.38	28	29.94	29.48	30.21	0.07	0.07	0.07
Bangladesh	10.67	10.43	10.91	14.01	14.64	13.39	0.32	0.41	0.24
Barbados	62.2	62.73	61.63	89.05	87.8	90.4	0.42	0.39	0.45
Belarus	28.1	24.68	31.97	36.39	33.38	39.88	0.28	0.34	0.23
Belgium	33.6	39.5	27.44	37.78	46.02	29.21	0.11	0.16	0.05
Belize	34.35	35.26	33.44	39.83	38.88	40.78	0.16	0.10	0.23
Benin	31.76	30.08	33.55	38.47	34.93	42.02	0.22	0.17	0.27
Bermuda	51.54	54.22	48.84	67.03	73.26	60.68	0.29	0.34	0.24
Bhutan	12.4	12.28	12.52	16.73	17.63	15.96	0.33	0.42	0.26
Bolivia	7.69	8.82	6.57	12.8	15.07	10.54	0.64	0.68	0.58
Bosnia and Herzegovina	57.15	55.02	59.28	107.86	110.63	105.06	0.86	0.99	0.75
Botswana	48.61	46.26	51.02	63.1	57.94	68.27	0.29	0.25	0.33
Brazil	62.22	61.14	63.32	86.97	85.49	88.49	0.39	0.39	0.39
Brunei	20.14	16.58	23.33	21.79	18.67	24.72	0.05	0.09	0.03
Bulgaria	34.77	36.22	33.29	51.44	60.77	41.64	0.47	0.67	0.24
Burkina Faso	29.54	27.41	31.78	32.53	30.17	34.91	0.11	0.10	0.11
Burundi	20.78	19.95	21.63	21.18	19.44	22.96	0.05	0.00	0.11
Cambodia	11.99	12.04	11.94	13.72	14.16	13.25	0.17	0.19	0.14
Cameroon	37.26	32.91	41.67	39.03	34.42	43.65	0.07	0.06	0.07
Canada	73.55	86.62	60.26	95.65	114.75	76.28	0.29	0.32	0.24
Cape Verde	49.87	49.75	50	67.91	71.39	64.34	0.35	0.42	0.28
Central African Republic	36.77	35.95	37.63	35.54	34.96	36.13	-0.03	-0.03	-0.04
Chad	30.57	27.38	33.81	31.74	28.17	35.31	0.04	0.03	0.05
Chile	23.59	29.27	17.76	41.77	50.93	32.37	0.74	0.71	0.79
China	11.06	11.47	10.68	17.55	19.84	15.38	0.62	0.75	0.48
Colombia	27.44	29.55	25.32	41.29	44.81	37.67	0.49	0.51	0.47
Comoros	26.14	25.28	27	29.48	27.8	31.13	0.13	0.10	0.16
Congo	40.87	38.34	43.41	41.36	38.79	43.92	0.02	0.02	0.02
Costa Rica	50.38	47.17	53.53	73.82	71	76.65	0.45	0.49	0.41
Cote d'Ivoire	31.87	26.2	37.1	36.98	29.75	43.97	0.17	0.14	0.20
Croatia	40.17	42.71	37.47	62.61	71.84	52.71	0.55	0.67	0.39
Cuba	46.25	42.88	49.56	72.88	70.51	75.22	0.56	0.63	0.50

Cyprus	25.09	26.94	23.19	26.23	29.6	22.64	0.02	0.08	-0.05
Czech Republic	35.48	39.83	30.87	59.64	70.39	48.54	0.67	0.75	0.56
Democratic Republic of the Congo	35.07	34.42	35.74	34.16	32.7	35.62	-0.02	-0.05	0.00
Denmark	31.15	35.66	26.5	32.94	37.28	28.54	0.05	0.04	0.07
Djibouti	24.43	24.54	24.31	32.61	31.86	33.35	0.33	0.29	0.36
Dominica	60.48	53.58	67.42	79.97	73.07	86.98	0.33	0.37	0.30
Dominican Republic	23.01	23.54	22.48	29.68	32.2	27.15	0.28	0.36	0.21
Ecuador	15.15	16.63	13.69	20.04	22.08	18	0.33	0.33	0.32
Egypt	35.28	35.96	34.6	44.81	45.61	44.02	0.27	0.27	0.27
El Salvador	19.99	19.47	20.52	28.67	29.01	28.29	0.44	0.49	0.39
Equatorial Guinea	40.11	38.75	41.41	45	40.39	49.39	0.12	0.04	0.19
Eritrea	20.21	19.51	20.92	22.08	21.14	23.02	0.10	0.09	0.11
Estonia	43.02	45.33	40.38	58.47	65.67	50.01	0.35	0.44	0.23
Ethiopia	39.42	36.9	41.95	43.69	41.65	45.74	0.12	0.14	0.10
Federated States of Micronesia	18.65	20.87	16.52	23.23	27.9	18.77	0.24	0.33	0.13
Fiji	14.35	15.96	12.8	18.6	21.94	15.37	0.29	0.37	0.19
Finland	45.8	55.85	35.15	64.37	82.6	45.57	0.39	0.47	0.27
France	65.4	79.18	50.87	75.93	93.26	57.47	0.15	0.17	0.12
Gabon	59.06	59.69	58.41	57.6	57.54	57.65	-0.03	-0.04	-0.01
Georgia	14.08	14.74	13.35	19.58	21.87	17.07	0.38	0.48	0.27
Germany	49.34	60.03	37.85	57.29	69.32	44.86	0.15	0.15	0.17
Ghana	29.63	25.45	33.68	39.23	34.8	43.69	0.34	0.38	0.32
Greece	36.11	41.23	30.86	43.89	54.52	32.73	0.21	0.32	0.05
Greenland	45.99	32.71	57.47	62.39	41.73	80.3	0.33	0.26	0.36
Grenada	50.13	52.61	47.6	58.98	63.52	54.47	0.19	0.22	0.17
Guam	25.11	30.04	20.81	38.66	49.45	28.16	0.55	0.65	0.37
Guatemala	23.28	22.88	23.68	28.93	30.05	27.77	0.27	0.34	0.20
Guinea	21.17	18.34	24.06	24.79	21.09	28.47	0.17	0.15	0.19
Guinea-Bissau	31.96	27.92	36.1	36.83	31.33	42.4	0.16	0.13	0.19
Guyana	31.09	30.56	31.63	34.46	33	35.91	0.11	0.08	0.14
Haiti	22.83	22.08	23.6	23.77	23.73	23.82	0.07	0.09	0.05
Honduras	21.26	19.92	22.59	24.36	23.29	25.42	0.15	0.17	0.13
Hungary	55.25	58.74	51.48	90.92	105.35	75.02	0.64	0.78	0.45
Iceland	22.5	19.74	25.24	24.97	22.78	27.21	0.10	0.14	0.06
India	6.44	7.51	5.45	7.65	8.89	6.49	0.19	0.18	0.19
Indonesia	14.84	13.49	16.19	19.91	18.67	21.13	0.36	0.40	0.33
Iran	15.73	15.76	15.69	18.04	18.21	17.87	0.13	0.13	0.12
Iraq	20.65	18.67	22.61	22.76	20.68	24.78	0.10	0.11	0.09
Ireland	34.04	35.93	32.13	37.19	39.72	34.59	0.08	0.10	0.06
Israel	24.19	24.98	23.4	30.33	35.43	25.14	0.25	0.41	0.07
Italy	81.11	100.61	60.43	106.52	137.62	73.44	0.30	0.36	0.20
Jamaica	54.26	52.9	55.67	70.42	67.65	73.2	0.28	0.27	0.30
Japan	67.93	75.5	60.09	101.18	127.72	73.23	0.46	0.67	0.19
Jordan	13.55	13.06	14	15.4	15.04	15.74	0.12	0.14	0.11
Kazakhstan	15.97	15.98	15.96	19.33	18.8	19.89	0.21	0.18	0.25
Kenya	17.34	16.57	18.11	17.59	17.04	18.14	0.02	0.03	0.01
Kiribati	20.35	22.97	17.68	23.17	26.98	19.25	0.14	0.18	0.09
Kuwait	20.53	20.52	20.54	21.71	22.5	21.09	0.04	0.08	0.01
Kyrgyzstan	15.77	14.04	17.57	17.45	14.7	20.26	0.11	0.05	0.15
Laos	12.86	12.75	12.97	13.93	14.05	13.8	0.10	0.12	0.08
Latvia	49.87	49.56	50.23	66.76	70.31	62.63	0.32	0.40	0.23
Lebanon	25.81	26.65	24.95	33.2	35.01	31.41	0.27	0.30	0.24
Lesotho	56.18	53.23	59.32	55.4	56.32	54.46	-0.02	0.06	-0.09
Liberia	31.32	26.08	36.59	39.09	33.15	44.94	0.25	0.27	0.24
Libya	25.16	23.66	26.5	29.6	29.17	30.03	0.17	0.22	0.13
Lithuania	31.36	30.51	32.3	47.66	48.98	46.12	0.50	0.59	0.41
Luxembourg	37.17	43.25	30.85	36.15	43.41	28.85	-0.03	0.00	-0.07
Macedonia	36.16	34.38	37.92	60.85	63.6	58.08	0.66	0.83	0.51
Madagascar	23.46	21.7	25.23	25.21	23.51	26.93	0.09	0.09	0.08
Malawi	19.64	18.72	20.6	21.99	20.77	23.21	0.14	0.12	0.15
Malaysia	24.33	24.9	23.78	28.97	29.33	28.6	0.19	0.18	0.20

Maldives	11.1	10.19	11.97	14.72	13.69	15.74	0.32	0.34	0.32
Mali	32.58	28.01	37.25	33.15	28.61	37.6	0.03	0.03	0.03
Malta	27.97	30.02	25.85	35.71	41.48	29.9	0.25	0.36	0.13
Marshall Islands	18.22	20.52	16.02	21.98	25.93	18.17	0.20	0.26	0.13
Mauritania	37.45	32.73	42.19	45.66	39.12	52.12	0.23	0.20	0.25
Mauritius	19.47	20.48	18.46	27.45	31.24	23.56	0.39	0.51	0.26
Mexico	22.38	23.03	21.72	28.3	29.6	27	0.26	0.28	0.23
Moldova	16.39	14.05	18.95	21.54	18.68	24.62	0.30	0.31	0.28
Mongolia	13	9.64	16.44	15.44	11.04	19.93	0.18	0.14	0.20
Montenegro	69.85	77.97	61.65	92.95	99.47	86.27	0.32	0.26	0.38
Morocco	27.44	28.5	26.37	40.92	43.85	37.93	0.48	0.52	0.42
Mozambique	8.9	8.52	9.32	10.41	9.81	11.04	0.18	0.16	0.20
Myanmar	15.65	14.34	17.01	21.24	20	22.53	0.39	0.42	0.37
Namibia	55.2	51.38	59.18	61.04	58.47	63.75	0.11	0.14	0.08
Nepal	10.71	10.95	10.47	12.84	13.05	12.62	0.21	0.20	0.22
Netherlands	40.61	48.44	32.6	51.05	59.87	42.08	0.25	0.23	0.27
New Zealand	41.71	44.11	39.25	52.54	55.51	49.46	0.24	0.24	0.24
Nicaragua	24.56	23.93	25.21	35.9	35.85	35.96	0.47	0.50	0.44
Niger	28.67	24.76	32.72	36.64	29.67	43.5	0.29	0.21	0.35
Nigeria	39.49	34.89	44.01	43.78	38.56	48.81	0.11	0.11	0.11
North Korea	12.61	14.44	10.66	18.89	24.85	12.66	0.50	0.72	0.19
Northern Mariana Islands	29.62	26.59	32.33	30.25	26.22	34.31	0.04	0.00	0.09
Norway	35.89	41.97	29.68	38.47	45.87	31.16	0.06	0.09	0.04
Oman	10.32	10.96	9.81	12.01	12.76	11.63	0.16	0.16	0.18
Pakistan	10.78	10.17	11.34	12.05	11.34	12.72	0.12	0.12	0.13
Palestine	17.25	16.59	17.9	18.46	17.83	19.07	0.06	0.07	0.05
Panama	34.09	30.08	38.01	51.37	46.22	56.49	0.53	0.56	0.52
Papua New Guinea	12.45	13.44	11.51	14.39	15.95	12.89	0.16	0.19	0.12
Paraguay	34.09	32.97	35.18	37.41	36.9	37.9	0.09	0.11	0.07
Peru	13.81	14.36	13.26	21.77	23.52	20.02	0.58	0.64	0.51
Philippines	14.26	14.7	13.82	15.95	17.17	14.75	0.13	0.18	0.08
Poland	38.04	40.77	35.17	63.25	72.81	52.97	0.65	0.77	0.49
Portugal	27.54	31.09	23.72	35.56	43.51	27.09	0.27	0.38	0.12
Puerto Rico	70.37	79.05	61.14	101.2	122.48	78.21	0.43	0.54	0.27
Qatar	23.97	21.26	25.3	26.44	22.42	27.94	0.09	0.04	0.09
Romania	44.93	42.14	47.8	79.72	83.04	76.19	0.76	0.96	0.58
Russia	39.69	37.05	42.70	58.82	56.99	60.96	0.47	0.53	0.41
Rwanda	19.71	18.94	20.5	22.38	22.2	22.58	0.14	0.18	0.11
Saint Lucia	52.88	50.43	55.4	74.25	72.74	75.82	0.42	0.45	0.40
Saint Vincent and the Grenadines	29.13	29.05	29.21	39.06	36.77	41.3	0.35	0.27	0.43
Samoa	24.87	28.86	21.19	33.33	41.9	25.27	0.33	0.44	0.19
Sao Tome and Principe	44.97	39.78	50.27	47.82	43.73	51.94	0.06	0.10	0.04
Saudi Arabia	29.01	28.87	29.12	35.89	35.16	36.46	0.22	0.20	0.23
Senegal	35.69	30.74	40.68	41.19	36.87	45.68	0.16	0.20	0.13
Serbia	74.46	73.5	75.44	111.51	117.59	105.13	0.48	0.58	0.38
Seychelles	23.11	26.63	19.57	26.58	31.66	21.64	0.14	0.18	0.10
Sierra Leone	32.27	26.78	37.86	34.79	29.7	39.98	0.10	0.13	0.08
Singapore	32.23	33.35	31.14	53.99	62.83	44.83	0.64	0.85	0.40
Slovakia	27.34	28.53	26.08	39.93	44.81	34.77	0.44	0.55	0.32
Slovenia	87.29	94.65	79.50	160.09	180.25	139.56	0.81	0.88	0.73
Solomon Islands	14.32	14.95	13.73	15.91	17.27	14.6	0.11	0.15	0.07
Somalia	22.34	20.91	23.8	22.56	20.84	24.31	0.01	0.00	0.02
South Africa	77.25	78.15	76.32	99.15	102	96.2	0.28	0.30	0.25
South Korea	24.08	26.18	22	35.11	43.47	26.64	0.41	0.62	0.17
South Sudan	23.16	21.63	24.7	25.45	23.45	27.45	0.10	0.09	0.11
Spain	47.92	54.37	41.19	59.41	71.83	46.67	0.23	0.31	0.12
Sri Lanka	24.83	25.31	24.35	33.37	36.46	30.05	0.36	0.45	0.26
Sudan	15.46	15.25	15.67	17.5	17.32	17.68	0.14	0.14	0.13
Suriname	35.79	36.38	35.22	45.53	47.24	43.83	0.27	0.29	0.24
Swaziland	47.29	42.63	52.5	51.69	48.98	54.45	0.09	0.15	0.03
Sweden	56.23	56.58	55.87	56.07	58.85	53.29	-0.01	0.03	-0.06

Switzerland	41.64	49.1	33.83	44.31	52.84	35.58	0.06	0.07	0.04
Syria	24.69	23.62	25.72	29.29	29.16	29.42	0.18	0.23	0.14
Taiwan	34.72	41.12	28.78	77.2	104.86	49.45	1.27	1.58	0.77
Tajikistan	10.98	9.52	12.45	12.58	9.92	15.17	0.14	0.04	0.22
Tanzania	23.09	22.02	24.19	24.63	22.58	26.71	0.08	0.04	0.13
Thailand	29.56	33.41	25.64	45.43	54.62	35.99	0.53	0.63	0.40
The Bahamas	41.35	39.68	43.05	59.84	58.53	61.2	0.43	0.46	0.41
The Gambia	32.17	25.55	38.7	37.91	31.37	44.59	0.18	0.23	0.16
Timor-Leste	12.24	11.07	13.37	15.28	14.71	15.83	0.26	0.34	0.19
Togo	30.03	27.07	33.07	34.29	30.84	37.82	0.15	0.15	0.16
Tonga	16.74	19.61	13.94	21.09	27.17	15.04	0.26	0.38	0.08
Trinidad and Tobago	35.55	34.97	36.14	47.21	47.4	47	0.31	0.34	0.29
Tunisia	26.46	27.96	24.98	39.8	45.3	34.17	0.48	0.60	0.35
Turkey	20.07	20.47	19.66	26.39	27.62	25.11	0.31	0.34	0.27
Turkmenistan	9.04	8.46	9.64	10.15	9.08	11.25	0.11	0.06	0.16
Uganda	21.73	20.04	23.43	21.3	19.66	22.95	0.00	-0.01	0.00
Ukraine	28.39	25.94	31.23	35.85	34.38	37.59	0.25	0.32	0.20
United Arab Emirates	26.27	26.05	26.38	29.3	26.67	30.25	0.10	0.01	0.13
United Kingdom	29.89	38.15	21.16	32.16	40.63	23.48	0.07	0.06	0.10
United States	118.14	130.13	105.54	129.96	143.31	116.17	0.09	0.10	0.09
Uruguay	61.39	65.78	56.74	80.22	92.55	67.01	0.30	0.40	0.17
Uzbekistan	6.87	6.52	7.22	7.07	6.45	7.72	0.02	-0.02	0.06
Vanuatu	17.72	19.61	15.94	18.32	20.71	15.99	0.00	0.03	-0.04
Venezuela	19.17	22.93	15.47	30.97	36.24	25.67	0.60	0.57	0.63
Vietnam	23.4	23.67	23.11	34.88	37.32	32.39	0.53	0.60	0.45
Virgin Islands, U.S.	62.15	54.72	70.03	111.87	104.09	120.43	0.78	0.88	0.70
Yemen	14.32	13.85	14.79	15.07	14.13	15.99	0.05	0.02	0.08
Zambia	19.79	18.9	20.69	21.85	20.73	22.97	0.12	0.11	0.13
Zimbabwe	42.74	36.45	49.09	40.45	35.06	46	-0.05	-0.04	-0.06
**Cerebrovascular disease**									
Afghanistan	379.00	416.87	342.95	392.23	435.55	351.55	0.04	0.05	0.03
Albania	476.97	494.59	460.20	856.76	853.92	859.65	0.78	0.71	0.86
Algeria	340.60	372.83	309.16	474.98	522.22	428.40	0.37	0.38	0.36
American Samoa	547.56	503.52	589.25	554.43	561.31	547.59	0.01	0.11	-0.07
Andorra	423.48	425.21	421.66	517.63	506	529.75	0.21	0.18	0.24
Angola	316.94	364.87	267.83	304.59	354.67	253.85	-0.03	-0.02	-0.04
Antigua and Barbuda	498.48	516.45	479.25	460.40	476.79	442.53	-0.08	-0.08	-0.08
Argentina	467.13	479.83	453.93	466.49	483.01	449.26	0.00	0.00	-0.01
Armenia	657.57	732.03	578.66	945.29	1,021.36	857.67	0.42	0.38	0.46
Australia	445.65	461.64	429.57	478.6	490.67	466.5	0.07	0.06	0.08
Austria	536.61	561.83	509.13	637.41	618.73	657.03	0.18	0.10	0.27
Azerbaijan	602.37	683.78	517.42	724.69	794.59	653.98	0.19	0.15	0.25
Bahrain	360.11	377.8	347.1	441.56	490.67	411.5	0.22	0.29	0.18
Bangladesh	492.87	473.13	511.74	550.93	523.26	578.07	0.13	0.11	0.14
Barbados	513.34	540.97	483.58	637.13	659.42	612.91	0.23	0.21	0.25
Belarus	1,155.79	1,369.17	914.11	1,426.88	1,643.82	1,175.23	0.22	0.19	0.27
Belgium	488.02	505.35	469.88	560.64	566.02	555.05	0.14	0.11	0.16
Belize	270.02	274.08	266.03	290.79	297.51	284.04	0.08	0.09	0.08
Benin	381.76	395.13	367.53	413.23	418.54	407.89	0.09	0.06	0.12
Bermuda	406.14	415.12	397.05	480.18	497.12	462.92	0.18	0.19	0.16
Bhutan	345.04	324.81	363.83	398.87	362.31	430.36	0.14	0.10	0.17
Bolivia	217.82	235.69	200.08	268.23	292.33	244.22	0.21	0.22	0.20
Bosnia and Herzegovina	652.08	740.11	563.95	1,057.09	1,132.03	981.45	0.60	0.51	0.72
Botswana	290.36	316.79	263.31	366.42	407.94	324.85	0.25	0.28	0.23
Brazil	275.15	271.03	279.35	285.34	279.44	291.43	0.03	0.02	0.03
Brunei	291.76	294.82	289.02	441.76	460.73	423.92	0.47	0.51	0.43
Bulgaria	1,167.22	1,177.02	1,157.15	1,390.32	1,458.16	1,319.11	0.18	0.23	0.13
Burkina Faso	366.65	381.51	351.11	306.15	325.89	286.15	-0.16	-0.14	-0.18
Burundi	456.59	501.29	410.32	416.23	441.68	390.23	-0.06	-0.10	-0.01
Cambodia	370.26	361.81	379.32	474.12	490.34	457.09	0.31	0.37	0.24
Cameroon	371.59	376.66	366.46	341.23	345.46	337.01	-0.07	-0.07	-0.06

Canada	375.24	392.86	357.33	484.51	486.09	482.91	0.28	0.23	0.33
Cape Verde	521.07	534.47	506.25	559.66	595.01	523.36	0.07	0.11	0.03
Central African Republic	478.20	527.92	426.79	426.58	481.61	370.09	-0.11	-0.09	-0.13
Chad	327.66	334.12	321.1	301.42	303.72	299.13	-0.08	-0.09	-0.07
Chile	420.05	444.57	394.86	554.67	585.36	523.17	0.30	0.30	0.30
China	615.00	564.91	662.52	845.81	725.57	959.07	0.40	0.30	0.49
Colombia	133.46	142.45	124.35	177.44	187.13	167.46	0.32	0.30	0.33
Comoros	405.16	439.74	370.80	402.57	434.68	371.05	0.00	-0.01	0.00
Congo	502.01	580.80	422.80	437.76	526.74	348.91	-0.12	-0.09	-0.17
Costa Rica	107.3	111.4	103.26	162.08	167.09	157.07	0.49	0.48	0.50
Cote d'Ivoire	439.59	396.80	479.00	437.75	406.89	467.56	0.01	0.03	-0.01
Croatia	1,041.12	1,156.02	919.02	1,284.62	1,359.74	1,204.00	0.22	0.17	0.30
Cuba	544.90	560.26	529.78	690.69	706.37	675.16	0.26	0.25	0.26
Cyprus	292.27	275.14	309.83	415.91	388.19	445.54	0.39	0.39	0.40
Czech Republic	894.46	977.42	806.51	905.28	968.55	839.96	0.00	-0.02	0.03
Democratic Republic of the Congo	301.40	357.54	243.87	283.31	325.22	241.19	-0.06	-0.09	-0.01
Denmark	564.30	560.37	568.35	576.42	555.33	597.84	0.02	-0.01	0.04
Djibouti	354.45	377.14	332.01	514.81	551.55	478.39	0.45	0.46	0.43
Dominica	436.17	452.91	419.31	517.22	546.04	487.95	0.19	0.21	0.18
Dominican Republic	308.44	308.13	308.75	404.62	407.19	402.03	0.31	0.31	0.30
Ecuador	216.11	225.92	206.41	244.58	257.78	231.37	0.13	0.14	0.12
Egypt	347.49	367.62	327.49	441.26	461.24	421.69	0.27	0.25	0.29
El Salvador	100.41	104.16	96.48	131.6	139.7	122.45	0.32	0.34	0.28
Equatorial Guinea	442.07	512.93	374.27	428.40	461.67	396.76	-0.03	-0.10	0.06
Eritrea	302.02	336.77	266.84	359.79	408.48	311.27	0.20	0.22	0.18
Estonia	1,098.42	1,273.82	898.83	1,154.32	1,353.84	920.04	0.04	0.05	0.01
Ethiopia	382.26	402.78	361.62	488.57	535.77	441.2	0.29	0.34	0.23
Federated States of Micronesia	536.12	519.83	551.71	604.13	597.94	610.04	0.12	0.14	0.10
Fiji	565.30	557.60	572.76	699.90	713.13	687.12	0.23	0.27	0.19
Finland	553.72	589.96	515.27	657.60	649.16	666.31	0.17	0.09	0.27
France	451.26	458.31	443.82	561.27	563.17	559.26	0.24	0.22	0.25
Gabon	596.69	680.91	508.95	472.69	558.57	388.73	-0.21	-0.18	-0.24
Georgia	1,138.82	1,235.41	1,032.29	1,336.73	1,396.30	1,271.57	0.17	0.12	0.22
Germany	512.51	547.39	475.03	629.90	607.09	653.49	0.22	0.10	0.36
Ghana	373.85	415.96	333.08	403.54	434.63	372.14	0.09	0.05	0.14
Greece	515.03	504.42	525.91	632.03	619.75	644.92	0.22	0.22	0.21
Greenland	293.14	292.88	293.36	447.27	399.37	488.80	0.50	0.35	0.62
Grenada	513.20	557.73	467.51	497.07	531.59	462.76	-0.02	-0.04	0.01
Guam	604.55	602.31	606.51	932.20	926.36	937.88	0.56	0.54	0.57
Guatemala	89.35	91.63	87.04	100.75	107.46	93.75	0.15	0.19	0.11
Guinea	377.45	396.21	358.27	387.24	393.21	381.30	0.03	-0.01	0.07
Guinea-Bissau	416.71	431.96	401.09	407.49	420.00	394.83	-0.01	-0.02	0.00
Guyana	389.13	393.32	384.81	542.93	558.63	527.38	0.40	0.42	0.38
Haiti	307.25	318.32	295.86	356.46	379.08	333.35	0.19	0.21	0.17
Honduras	118.59	126.25	111.00	141.31	140.11	142.52	0.19	0.11	0.29
Hungary	1,143.98	1,206.65	1,076.19	1,156.22	1,269.61	1,031.23	0.00	0.05	-0.05
Iceland	316.32	309.44	323.13	383.41	366.39	400.8	0.20	0.17	0.22
India	385.35	358.76	410.12	439.91	391.37	485.01	0.14	0.09	0.19
Indonesia	543.59	510.87	576.04	664.09	631.04	696.66	0.24	0.25	0.23
Iran	313.07	317.42	308.88	447.73	464.98	430.74	0.41	0.44	0.37
Iraq	387.02	413.14	361.37	386.99	422.92	351.95	0.00	0.02	-0.03
Ireland	389.52	394.19	384.81	407.09	389.57	425.13	0.03	-0.02	0.09
Israel	304.39	291.27	317.56	328.6	316.66	340.73	0.07	0.08	0.06
Italy	491.22	488.10	494.53	560.25	548.86	572.36	0.13	0.12	0.14
Jamaica	438.67	458.55	418.28	467.83	478.67	456.89	0.05	0.03	0.08
Japan	796.70	720.47	875.67	960.10	826.37	1,100.94	0.18	0.13	0.22
Jordan	318.02	346.57	292.19	314.68	346.43	284.47	-0.02	-0.01	-0.04
Kazakhstan	784.24	905.27	655.62	913.37	1,003.19	817.13	0.16	0.11	0.25
Kenya	246.68	256.52	236.77	248.04	251.3	244.79	0.01	-0.02	0.04
Kiribati	600.83	577.77	624.38	668.28	628.87	708.71	0.12	0.09	0.14
Kuwait	291.29	272.46	307.76	351.77	363.2	342.85	0.19	0.31	0.10

Kyrgyzstan	714.59	801.27	624.18	694.75	738.76	649.79	-0.03	-0.08	0.04
Laos	412.82	402.09	423.57	466.81	463.15	470.51	0.15	0.17	0.13
Latvia	1,166.89	1,375.27	926.88	1,324.28	1,533.88	1,080.18	0.12	0.10	0.15
Lebanon	423.31	486.69	358.19	478.97	533.89	424.62	0.12	0.09	0.17
Lesotho	350.93	371.54	329.10	397.07	457.73	335.19	0.13	0.23	0.01
Liberia	330.62	330.17	331.07	381.94	393.30	370.77	0.16	0.19	0.13
Libya	270.89	288.10	255.48	431.57	473.54	390.10	0.58	0.63	0.52
Lithuania	1,028.86	1,226.65	808.73	1,407.93	1,659.68	1,115.14	0.35	0.34	0.36
Luxembourg	496.09	517.24	474.08	516.04	515.41	516.68	0.04	0.00	0.09
Macedonia	746.71	785.17	708.70	1,035.77	1,091.42	979.62	0.37	0.38	0.37
Madagascar	465.47	469.22	461.70	512.09	544.22	479.76	0.11	0.17	0.05
Malawi	323.99	361.23	285.43	398.4	444.76	351.87	0.25	0.25	0.26
Malaysia	384.15	366.23	401.53	498.02	473.68	522.81	0.29	0.29	0.30
Maldives	282.61	221.47	340.66	318.72	297.29	340.04	0.13	0.34	0.00
Mali	397.38	435.66	358.26	312.48	336.97	288.48	-0.20	-0.22	-0.18
Malta	355.83	348.59	363.33	554.46	528.23	580.89	0.53	0.49	0.56
Marshall Islands	453.25	420.55	484.52	553.30	527.97	577.76	0.22	0.25	0.19
Mauritania	415.28	429.84	400.63	396.33	419.12	373.86	-0.04	-0.02	-0.06
Mauritius	582.83	559.08	606.68	773.37	743.07	804.42	0.31	0.32	0.30
Mexico	97.61	104.72	90.43	101.83	99.3	104.39	0.04	-0.06	0.15
Moldova	892.77	1,003.04	771.79	967.22	1,072.21	853.72	0.07	0.05	0.09
Mongolia	578.75	610.71	546.04	897.73	943.39	851.14	0.54	0.53	0.55
Montenegro	661.23	741.99	579.77	1,065.31	1,162.38	965.93	0.59	0.55	0.65
Morocco	312.21	347.93	276.36	433.01	481.93	382.94	0.37	0.37	0.37
Mozambique	427.42	450.30	402.48	551.37	600.18	500.33	0.30	0.34	0.25
Myanmar	559.23	520.95	599.06	694.66	660.24	730.66	0.27	0.29	0.26
Namibia	372.67	398.06	346.18	424.49	477.84	368.28	0.14	0.20	0.07
Nepal	366.85	360.66	373.13	453.47	426.59	482.05	0.25	0.19	0.31
Netherlands	434.74	452.47	416.59	520.41	507.01	534.04	0.19	0.12	0.26
New Zealand	462.47	477.43	447.05	505.01	496.99	513.34	0.08	0.03	0.13
Nicaragua	101.78	108.37	95.1	135.55	146.18	124.62	0.34	0.35	0.33
Niger	293.38	300.33	286.19	320.5	313.83	327.07	0.11	0.05	0.16
Nigeria	323.55	334.45	312.86	295.16	298.26	292.16	-0.09	-0.11	-0.06
North Korea	559.22	621.59	493.16	806.12	850.22	760.02	0.45	0.37	0.55
Northern Mariana Islands	495.81	444.24	542.15	487.88	426.27	549.92	0.00	-0.03	0.04
Norway	527.61	528.29	526.90	484.95	482.2	487.66	-0.09	-0.09	-0.09
Oman	237.13	265.91	214.17	311.34	342.61	295.48	0.31	0.28	0.37
Pakistan	441.78	391.36	488.57	477.63	437.56	515.56	0.08	0.12	0.06
Palestine	262.02	289.59	235.14	311.84	343.23	281.33	0.18	0.17	0.18
Panama	146.49	143.72	149.2	196.26	196.87	195.66	0.36	0.39	0.34
Papua New Guinea	461.67	464.62	458.86	589.32	624.15	555.86	0.28	0.34	0.21
Paraguay	254.98	253.51	256.43	373.80	357.45	389.64	0.45	0.40	0.50
Peru	197.93	211.82	184.05	247.07	266.21	227.91	0.25	0.26	0.24
Philippines	341.83	322.49	360.77	480.64	468.73	492.32	0.42	0.46	0.38
Poland	747.06	819.89	670.45	918.90	985.20	847.56	0.22	0.19	0.25
Portugal	606.14	598.03	614.86	706.58	693.15	720.90	0.15	0.15	0.15
Puerto Rico	422.61	425.74	419.28	547.04	571.26	520.87	0.28	0.33	0.23
Qatar	259.76	250.67	264.25	278.14	301.53	269.4	0.06	0.18	0.01
Romania	1,035.89	1,111.38	958.24	1,294.51	1,384.72	1,198.48	0.24	0.24	0.24
Russia	1,135.43	1,361.92	878.22	1,395.94	1,585.03	1,173.90	0.22	0.16	0.32
Rwanda	370.20	398.31	341.09	394.46	448.44	335.74	0.07	0.13	-0.01
Saint Lucia	481.42	493.14	469.34	511.66	532.95	489.54	0.08	0.09	0.06
Saint Vincent and the Grenadines	423.84	464.51	382.77	483.92	504.99	463.23	0.15	0.09	0.22
Samoa	614.96	608.18	621.21	671.69	680.31	663.61	0.09	0.11	0.06
Sao Tome and Principe	387.19	396.85	377.34	367.59	388.51	346.52	-0.05	-0.02	-0.08
Saudi Arabia	250.41	261.2	241.93	311.17	297.37	321.76	0.22	0.12	0.31
Senegal	358.09	360.92	355.23	340.11	358.61	320.92	-0.04	0.00	-0.09
Serbia	907.91	981.51	832.35	1,196.91	1,287.98	1,101.39	0.30	0.30	0.31
Seychelles	528.80	505.57	552.12	557.09	542.88	570.92	0.05	0.07	0.03
Sierra Leone	401.75	382.66	421.24	425.88	432.23	419.40	0.08	0.15	0.02
Singapore	494.95	515.53	474.91	699.08	718.13	679.35	0.38	0.37	0.40

Slovakia	759.24	818.19	697.51	894.17	956.83	828.05	0.16	0.16	0.17
Slovenia	750.18	833.74	661.58	922.26	981.87	861.57	0.21	0.17	0.28
Solomon Islands	550.52	529.94	570.00	588.36	582.44	594.10	0.07	0.10	0.04
Somalia	391.06	425.37	356.13	395.42	431.55	358.96	0.01	0.02	0.01
South Africa	500.54	504.39	496.6	552.69	562.70	542.33	0.10	0.11	0.09
South Korea	609.10	664.41	554.04	962.45	991.56	933.00	0.53	0.45	0.63
South Sudan	399.37	431.19	367.25	464.05	507.30	420.94	0.16	0.18	0.15
Spain	461.12	473.48	448.25	561.98	577.81	545.74	0.21	0.21	0.20
Sri Lanka	372.12	344.88	398.83	647.82	637.77	658.62	0.76	0.87	0.68
Sudan	323.92	349.84	298.21	376.73	416.71	337.04	0.17	0.19	0.14
Suriname	406.49	417.45	395.89	519.49	536.42	502.62	0.27	0.28	0.27
Swaziland	279.96	291.60	266.95	364.37	412.67	315.05	0.30	0.41	0.17
Sweden	645.76	575.83	717.38	591.35	496.89	686	-0.09	-0.14	-0.06
Switzerland	443.70	449.38	437.76	471.33	469.22	473.48	0.06	0.04	0.07
Syria	303.30	308.48	298.29	417.22	435.93	398.95	0.37	0.40	0.33
Taiwan	589.39	539.14	636.04	768.49	766.2	770.79	0.33	0.44	0.25
Tajikistan	499.94	565.74	433.35	421.13	453.17	389.85	-0.16	-0.20	-0.10
Tanzania	333.92	362.88	304.45	373.18	401.55	344.46	0.14	0.12	0.15
Thailand	424.42	390.40	459.06	703.41	655.08	753.12	0.66	0.67	0.64
The Bahamas	379.52	396.10	362.71	506.81	536.00	476.36	0.32	0.34	0.30
The Gambia	338.66	322.25	354.84	334.23	319.91	348.82	-0.01	-0.01	-0.01
Timor-Leste	286.91	280.83	292.83	419.66	420.95	418.42	0.48	0.51	0.44
Togo	345.44	351.07	339.66	334.87	333.31	336.47	-0.02	-0.05	0.00
Tonga	530.08	563.23	497.73	572.43	603.31	541.71	0.08	0.07	0.08
Trinidad and Tobago	419.18	426.08	412.22	539.83	543.27	536.30	0.27	0.26	0.29
Tunisia	392.52	396.20	388.89	555.55	580.29	530.23	0.40	0.45	0.34
Turkey	348.2	383.18	312.36	414.07	465.14	361.2	0.18	0.20	0.15
Turkmenistan	545.50	608.74	480.49	677.55	717.96	635.75	0.23	0.17	0.31
Uganda	336.50	368.14	304.44	310.09	336.69	283.47	-0.06	-0.08	-0.05
Ukraine	1,239.98	1,448.54	997.27	1,398.53	1,588.21	1,174.48	0.12	0.09	0.17
United Arab Emirates	324.05	319.86	326.22	361.58	353.61	364.49	0.10	0.09	0.10
United Kingdom	857.09	924.07	786.25	937.29	950	924.26	0.09	0.03	0.17
United States	410.38	426.97	392.94	414.12	410.45	417.9	0.00	-0.04	0.05
Uruguay	618.19	637.72	597.47	626.67	672.53	577.53	0.01	0.05	-0.04
Uzbekistan	466.13	518.18	412.94	533.39	567.39	498.28	0.14	0.09	0.20
Vanuatu	680.15	631.28	726.35	747.17	738.48	755.65	0.06	0.14	0.00
Venezuela	146.09	151.41	140.84	199.67	205.78	193.5	0.35	0.35	0.35
Vietnam	573.47	537.18	610.88	630.61	604.20	657.59	0.13	0.14	0.11
Virgin Islands, U.S.	382.44	389.32	375.16	676.25	678.18	674.12	0.75	0.72	0.77
Yemen	289.07	326.36	251.09	356.50	402.27	311.67	0.23	0.23	0.24
Zambia	342.39	379.75	304.4	422.48	457.41	387.47	0.25	0.22	0.29
Zimbabwe	289.71	301.57	277.76	232.68	258.8	205.79	-0.20	-0.14	-0.26
**Endocarditis**									
Afghanistan	1.42	2.57	0.33	1.67	3.04	0.37	0.18	0.19	0.14
Albania	0.46	0.4	0.52	0.65	0.44	0.86	0.39	0.09	0.64
Algeria	2.15	3.83	0.51	3.78	6.98	0.62	0.73	0.79	0.21
American Samoa	5.16	7.71	2.76	5.66	8.79	2.54	0.09	0.14	-0.08
Andorra	8.09	14.34	1.51	9.3	16.47	1.83	0.14	0.14	0.19
Angola	0.59	0.67	0.5	0.59	0.68	0.5	0.01	0.01	0.01
Antigua and Barbuda	2.49	4.34	0.51	2.49	4.34	0.48	0.00	-0.01	-0.06
Argentina	5.47	10.98	-0.25	7.74	15.14	0.01	0.41	0.37	-1.02
Armenia	0.61	0.6	0.62	1.06	0.99	1.14	0.72	0.63	0.82
Australia	2.67	2.67	2.68	2.16	2.05	2.27	-0.20	-0.24	-0.16
Austria	11.65	20.41	2.11	11.12	19.75	2.07	-0.05	-0.04	-0.03
Azerbaijan	0.63	0.68	0.58	0.86	0.84	0.87	0.35	0.22	0.49
Bahrain	2.3	4.54	0.65	2.51	5.66	0.58	0.08	0.24	-0.12
Bangladesh	0.39	0.46	0.33	0.66	0.77	0.55	0.68	0.68	0.66
Barbados	3.19	5.62	0.57	4.41	7.84	0.68	0.37	0.39	0.18
Belarus	0.83	0.93	0.71	1.19	1.34	1.02	0.42	0.42	0.42
Belgium	9.36	16.48	1.9	9.62	16.73	2.22	0.02	0.01	0.15
Belize	1.12	2.02	0.24	1.22	2.17	0.26	0.09	0.07	0.08

Benin	0.71	0.8	0.61	0.7	0.82	0.59	0.01	0.02	-0.01
Bermuda	1.95	3.41	0.47	2.77	5.08	0.41	0.41	0.48	-0.12
Bhutan	0.45	0.51	0.4	0.75	0.85	0.66	0.65	0.65	0.66
Bolivia	-1.06	-0.9	-1.22	-0.74	-0.75	-0.73	-0.32	-0.18	-0.41
Bosnia and Herzegovina	0.54	0.41	0.68	0.75	0.39	1.12	0.37	-0.06	0.64
Botswana	0.4	0.38	0.41	0.47	0.46	0.48	0.18	0.20	0.16
Brazil	0.69	0.63	0.75	1.02	0.95	1.1	0.47	0.50	0.45
Brunei	1.24	1.43	1.07	1.34	1.52	1.18	0.06	0.03	0.08
Bulgaria	0.93	0.57	1.3	1.05	0.5	1.62	0.12	-0.12	0.24
Burkina Faso	0.64	0.71	0.57	0.66	0.75	0.58	0.04	0.05	0.02
Burundi	0.62	0.69	0.55	0.53	0.59	0.48	-0.12	-0.13	-0.10
Cambodia	0.55	0.64	0.45	0.61	0.69	0.52	0.13	0.10	0.18
Cameroon	0.72	0.8	0.64	0.7	0.77	0.62	-0.02	-0.02	0.00
Canada	2.88	3.11	2.65	2.78	2.66	2.9	-0.05	-0.15	0.07
Cape Verde	0.86	1.03	0.67	0.99	1.19	0.8	0.15	0.14	0.19
Central African Republic	0.6	0.68	0.51	0.62	0.7	0.54	0.04	0.04	0.04
Chad	0.66	0.75	0.58	0.68	0.77	0.6	0.03	0.03	0.04
Chile	5.01	9.86	0.02	9.26	17.5	0.79	0.82	0.75	37.99
China	0.26	0.21	0.32	0.44	0.4	0.48	0.72	0.95	0.57
Colombia	0.81	1.09	0.53	0.97	1.24	0.68	0.18	0.13	0.27
Comoros	0.71	0.78	0.64	0.67	0.75	0.6	-0.05	-0.03	-0.07
Congo	0.71	0.79	0.62	0.69	0.8	0.58	-0.02	0.01	-0.06
Costa Rica	0.92	1.09	0.74	1.17	1.37	0.98	0.27	0.24	0.30
Cote d'Ivoire	0.68	0.74	0.63	0.7	0.76	0.63	0.03	0.03	0.02
Croatia	0.5	0.36	0.66	0.48	0.27	0.7	-0.06	-0.25	0.05
Cuba	2.53	4.79	0.31	4.01	7.71	0.34	0.57	0.60	0.09
Cyprus	3.57	6.07	1.02	4.33	7.32	1.14	0.19	0.18	0.09
Czech Republic	0.82	0.56	1.11	0.96	0.55	1.38	0.15	-0.01	0.23
Democratic Republic of the Congo	0.52	0.62	0.43	0.51	0.59	0.43	-0.02	-0.04	0.00
Denmark	8.21	14.42	1.82	6.82	11.72	1.83	-0.17	-0.19	0.00
Djibouti	0.71	0.78	0.63	0.84	0.91	0.76	0.18	0.17	0.20
Dominica	1.92	3.47	0.36	2.77	5.04	0.46	0.45	0.45	0.27
Dominican Republic	1.19	2.1	0.29	2.24	4.15	0.32	0.88	0.96	0.11
Ecuador	0.39	0.54	0.24	0.53	0.66	0.41	0.37	0.22	0.68
Egypt	2.58	4.62	0.55	3.26	6.02	0.56	0.27	0.30	0.02
El Salvador	0.61	0.76	0.46	0.78	0.96	0.57	0.28	0.27	0.27
Equatorial Guinea	0.73	0.83	0.63	0.7	0.76	0.64	-0.04	-0.08	0.02
Eritrea	0.55	0.61	0.5	0.59	0.67	0.52	0.07	0.09	0.05
Estonia	1.01	1.08	0.94	1.37	1.53	1.18	0.34	0.41	0.24
Ethiopia	0.63	0.68	0.57	0.64	0.7	0.58	0.03	0.02	0.03
Federated States of Micronesia	5.08	7.55	2.72	6.95	10.63	3.45	0.36	0.40	0.26
Fiji	5.41	7.83	3.07	9.35	14.01	4.85	0.72	0.78	0.57
Finland	9.5	17.06	1.48	9.8	17.77	1.59	0.02	0.04	0.06
France	6.73	11.48	1.72	6.97	11.71	1.93	0.03	0.02	0.12
Gabon	0.88	1	0.76	0.86	1.02	0.71	-0.02	0.02	-0.07
Georgia	0.96	0.95	0.97	1.28	1.21	1.37	0.33	0.26	0.41
Germany	10.09	17.9	1.7	10.26	18.19	2.07	0.01	0.01	0.20
Ghana	0.71	0.84	0.57	0.78	0.87	0.68	0.11	0.03	0.21
Greece	6.34	11.15	1.42	9.63	16.8	2.11	0.51	0.50	0.47
Greenland	2.67	4.37	1.2	2.92	4.9	1.2	0.07	0.11	-0.03
Grenada	2.2	3.87	0.49	2.54	4.56	0.53	0.17	0.19	0.12
Guam	5.99	9.5	2.92	13.99	21.46	6.73	1.36	1.27	1.34
Guatemala	0.56	0.67	0.44	0.62	0.75	0.49	0.15	0.14	0.15
Guinea	0.71	0.81	0.62	0.68	0.77	0.6	-0.04	-0.05	-0.02
Guinea-Bissau	0.72	0.81	0.63	0.75	0.83	0.66	0.06	0.04	0.08
Guyana	1.3	2.24	0.34	1.76	3.11	0.42	0.35	0.39	0.24
Haiti	0.98	1.64	0.3	1.22	2.12	0.29	0.28	0.32	0.00
Honduras	0.56	0.67	0.45	0.75	0.87	0.62	0.33	0.29	0.38
Hungary	0.82	0.62	1.04	0.97	0.66	1.32	0.18	0.06	0.26
Iceland	5.23	9.11	1.39	5.33	9.03	1.56	0.01	-0.02	0.10
India	0.45	0.5	0.4	0.68	0.76	0.61	0.52	0.51	0.54

Indonesia	0.58	0.65	0.52	0.78	0.87	0.69	0.35	0.35	0.35
Iran	1.61	2.99	0.28	3.46	6.53	0.43	1.11	1.14	0.51
Iraq	2.3	4.24	0.39	2.4	4.44	0.42	0.05	0.05	0.08
Ireland	5.64	9.94	1.31	5.14	8.86	1.3	-0.10	-0.12	-0.02
Israel	4.17	7.21	1.12	4.45	7.71	1.14	0.06	0.07	0.01
Italy	5.54	9.7	1.11	7.47	12.95	1.63	0.34	0.33	0.45
Jamaica	1.96	3.51	0.37	2.53	4.6	0.44	0.28	0.30	0.18
Japan	1.92	2.27	1.57	2.45	2.86	2.03	0.25	0.24	0.26
Jordan	2.28	4.34	0.41	2.8	5.3	0.42	0.21	0.21	0.02
Kazakhstan	0.7	0.8	0.58	0.86	0.91	0.8	0.23	0.13	0.38
Kenya	0.47	0.52	0.42	0.51	0.55	0.47	0.08	0.06	0.11
Kiribati	4.98	7.45	2.46	5.7	8.63	2.69	0.15	0.16	0.10
Kuwait	1.78	3.35	0.4	2.34	4.7	0.5	0.30	0.39	0.22
Kyrgyzstan	0.54	0.59	0.48	0.57	0.58	0.56	0.07	-0.01	0.17
Laos	0.65	0.71	0.6	0.7	0.76	0.63	0.09	0.09	0.08
Latvia	0.57	0.57	0.56	0.64	0.7	0.58	0.12	0.20	0.01
Lebanon	3.37	6.16	0.5	4.61	8.77	0.49	0.35	0.41	-0.03
Lesotho	0.4	0.43	0.36	0.51	0.54	0.48	0.28	0.24	0.32
Liberia	0.68	0.75	0.61	0.68	0.77	0.59	0.00	0.03	-0.03
Libya	1.83	3.34	0.48	3.26	6.01	0.54	0.77	0.78	0.12
Lithuania	1.62	1.73	1.5	2.54	2.84	2.2	0.55	0.63	0.45
Luxembourg	9.88	17.54	1.91	9.97	17.92	1.98	0.01	0.02	0.04
Macedonia	0.58	0.43	0.73	0.71	0.44	0.99	0.21	0.01	0.34
Madagascar	0.79	0.84	0.74	0.77	0.82	0.72	-0.01	-0.01	-0.01
Malawi	0.51	0.58	0.44	0.54	0.6	0.47	0.07	0.04	0.11
Malaysia	0.85	0.88	0.81	1.11	1.08	1.14	0.31	0.23	0.40
Maldives	0.53	0.59	0.47	0.66	0.75	0.57	0.25	0.26	0.22
Mali	0.71	0.83	0.58	0.65	0.76	0.55	-0.06	-0.07	-0.05
Malta	5.76	10.02	1.34	9.45	16.76	2.08	0.61	0.65	0.51
Marshall Islands	3.96	5.95	2.06	5.8	8.78	2.93	0.46	0.47	0.41
Mauritania	0.78	0.87	0.68	0.78	0.89	0.67	0.01	0.02	-0.01
Mauritius	0.81	1	0.62	0.94	0.97	0.91	0.15	-0.03	0.44
Mexico	0.66	0.82	0.5	0.85	1.01	0.69	0.28	0.22	0.37
Moldova	0.56	0.58	0.54	0.76	0.77	0.75	0.33	0.30	0.37
Mongolia	0.37	0.34	0.39	0.57	0.54	0.61	0.55	0.56	0.55
Montenegro	0.53	0.37	0.69	0.72	0.4	1.05	0.34	0.08	0.49
Morocco	2.11	3.62	0.59	3.51	6.27	0.68	0.64	0.71	0.14
Mozambique	0.53	0.61	0.44	0.61	0.68	0.53	0.16	0.13	0.22
Myanmar	0.59	0.65	0.53	0.76	0.84	0.68	0.32	0.31	0.33
Namibia	0.44	0.45	0.44	0.49	0.48	0.5	0.11	0.07	0.15
Nepal	0.43	0.51	0.35	0.64	0.75	0.53	0.50	0.48	0.52
Netherlands	8.11	14.57	1.51	8.41	14.7	2.01	0.03	0.01	0.31
New Zealand	1.03	1.08	0.99	1.11	1.04	1.17	0.05	-0.05	0.17
Nicaragua	0.59	0.73	0.45	0.8	0.99	0.61	0.37	0.37	0.37
Niger	0.65	0.73	0.57	0.58	0.66	0.51	-0.08	-0.08	-0.08
Nigeria	0.66	0.73	0.6	0.65	0.7	0.59	-0.02	-0.03	0.00
North Korea	0.39	0.32	0.45	0.58	0.59	0.57	0.50	0.82	0.26
Northern Mariana Islands	3.64	5.48	1.97	3.59	5	2.17	0.00	-0.08	0.12
Norway	9.95	17.7	2.03	7.33	12.97	1.75	-0.27	-0.27	-0.15
Oman	1.76	3.3	0.53	2.02	4.69	0.66	0.14	0.42	0.24
Pakistan	0.48	0.53	0.43	0.66	0.74	0.58	0.39	0.42	0.35
Palestine	1.72	3.09	0.38	2.21	4.07	0.4	0.27	0.31	0.03
Panama	0.77	0.89	0.65	1.04	1.18	0.9	0.37	0.34	0.42
Papua New Guinea	3.04	4.63	1.53	4.28	6.53	2.11	0.41	0.41	0.39
Paraguay	0.75	0.67	0.83	1.03	0.9	1.16	0.36	0.33	0.39
Peru	0.53	0.65	0.41	0.63	0.76	0.5	0.20	0.18	0.22
Philippines	0.51	0.63	0.39	0.68	0.82	0.54	0.35	0.32	0.41
Poland	0.82	0.56	1.1	1.02	0.45	1.62	0.22	-0.19	0.46
Portugal	6.85	11.88	1.45	8.85	15.44	1.83	0.27	0.28	0.24
Puerto Rico	2.56	4.58	0.41	4.3	7.83	0.48	0.67	0.70	0.15
Qatar	1.27	2.87	0.48	1.3	3.52	0.47	0.01	0.21	-0.04

Romania	1.15	0.73	1.58	1.61	0.83	2.44	0.39	0.13	0.53
Russia	0.7	0.82	0.55	1.03	1.09	0.95	0.46	0.32	0.71
Rwanda	0.59	0.66	0.51	0.56	0.63	0.49	-0.04	-0.05	-0.04
Saint Lucia	2.09	3.59	0.53	2.83	4.99	0.59	0.38	0.40	0.13
Saint Vincent and the Grenadines	1.83	3.15	0.5	2.17	4.01	0.37	0.19	0.27	-0.26
Samoa	6.36	9.85	3.13	9.81	15.47	4.51	0.53	0.56	0.43
Sao Tome and Principe	0.88	0.97	0.78	0.83	0.93	0.73	-0.05	-0.04	-0.06
Saudi Arabia	1.82	3.64	0.4	2.49	5.07	0.5	0.34	0.37	0.24
Senegal	0.64	0.73	0.56	0.69	0.8	0.58	0.08	0.10	0.05
Serbia	0.47	0.3	0.65	0.59	0.33	0.87	0.24	0.08	0.33
Seychelles	0.88	0.95	0.8	0.99	1.09	0.9	0.13	0.14	0.12
Sierra Leone	0.68	0.75	0.61	0.7	0.79	0.6	0.04	0.07	0.00
Singapore	1.69	2.11	1.29	1.94	2.28	1.59	0.12	0.06	0.20
Slovakia	0.63	0.45	0.81	0.69	0.41	0.99	0.09	-0.10	0.20
Slovenia	0.69	0.44	0.97	0.81	0.33	1.29	0.15	-0.26	0.32
Solomon Islands	3.59	4.97	2.28	4.62	6.61	2.69	0.29	0.33	0.18
Somalia	0.7	0.76	0.64	0.64	0.7	0.59	-0.08	-0.09	-0.08
South Africa	0.56	0.61	0.51	0.6	0.65	0.54	0.06	0.06	0.06
South Korea	1.42	1.79	1.06	1.56	1.93	1.18	0.06	0.05	0.07
South Sudan	0.65	0.72	0.58	0.67	0.73	0.6	0.03	0.02	0.04
Spain	8.48	15.06	1.63	11.48	20.69	2.04	0.34	0.36	0.24
Sri Lanka	0.73	0.78	0.68	0.96	0.96	0.97	0.33	0.23	0.45
Sudan	1.66	2.95	0.38	2.06	3.73	0.4	0.24	0.26	0.04
Suriname	1.43	2.63	0.28	2.12	3.91	0.33	0.47	0.48	0.20
Swaziland	0.37	0.38	0.35	0.48	0.49	0.47	0.30	0.28	0.32
Sweden	6.55	11.14	1.84	6.75	11.47	2.02	0.02	0.02	0.08
Switzerland	7.06	12.52	1.35	7.26	12.59	1.82	0.02	0.00	0.33
Syria	1.62	2.98	0.31	2.88	5.47	0.35	0.76	0.82	0.13
Taiwan	0.89	0.58	1.17	1.5	1.28	1.72	0.72	1.21	0.51
Tajikistan	0.47	0.48	0.46	0.47	0.44	0.5	-0.01	-0.10	0.08
Tanzania	0.56	0.62	0.5	0.59	0.64	0.53	0.06	0.05	0.07
Thailand	0.77	0.87	0.68	1.15	1.18	1.13	0.49	0.35	0.68
The Bahamas	1.68	2.93	0.42	2.92	5.27	0.48	0.72	0.78	0.13
The Gambia	0.68	0.76	0.59	0.65	0.73	0.57	-0.04	-0.05	-0.04
Timor-Leste	0.56	0.65	0.47	0.58	0.63	0.53	0.04	-0.03	0.14
Togo	0.64	0.73	0.56	0.63	0.71	0.55	-0.01	-0.02	0.00
Tonga	6.92	10.46	3.46	10.38	16.17	4.63	0.50	0.54	0.33
Trinidad and Tobago	1.79	3.16	0.41	2.93	5.31	0.47	0.62	0.66	0.14
Tunisia	2.85	5.15	0.57	5.45	10.08	0.71	0.89	0.93	0.22
Turkey	3.59	6.64	0.46	5.04	9.48	0.44	0.39	0.42	-0.05
Turkmenistan	0.47	0.5	0.44	0.59	0.59	0.6	0.27	0.18	0.36
Uganda	0.48	0.54	0.42	0.49	0.54	0.45	0.04	0.00	0.08
Ukraine	0.88	0.98	0.76	1.18	1.28	1.06	0.33	0.30	0.38
United Arab Emirates	1.38	2.88	0.6	1.37	3.23	0.7	-0.02	0.11	0.15
United Kingdom	4.52	7.8	1.05	5.52	9.57	1.37	0.22	0.22	0.30
United States	3.16	3.27	3.05	3.32	3.26	3.39	0.04	0.00	0.10
Uruguay	11.63	21.53	1.13	14.84	27.34	1.45	0.27	0.26	0.27
Uzbekistan	0.45	0.49	0.41	0.59	0.6	0.58	0.30	0.21	0.40
Vanuatu	5.16	7.01	3.41	6.2	8.95	3.52	0.16	0.24	-0.01
Venezuela	-0.29	0.15	-0.74	0.48	0.87	0.09	-2.62	4.58	-1.12
Vietnam	0.61	0.7	0.52	0.78	0.89	0.67	0.31	0.29	0.33
Virgin Islands, U.S.	1.86	3.18	0.46	4.45	7.9	0.65	1.36	1.45	0.42
Yemen	1.63	2.87	0.38	1.84	3.32	0.4	0.13	0.16	0.05
Zambia	0.5	0.57	0.43	0.6	0.66	0.55	0.22	0.17	0.30
Zimbabwe	0.3	0.26	0.34	0.32	0.27	0.36	0.07	0.07	0.07
**Hemorrhagic and other stroke**									
Afghanistan	197.61	228.18	168.5	208.76	242.28	177.29	0.06	0.06	0.06
Albania	214.25	234.30	195.16	348.01	368.74	326.95	0.61	0.56	0.67
Algeria	163.67	185.28	142.58	213.88	244.56	183.62	0.29	0.30	0.27
American Samoa	295.63	282.93	307.66	314.44	325.96	302.99	0.06	0.15	-0.02
Andorra	148.34	151.54	144.96	182.5	182.46	182.54	0.22	0.20	0.24

Angola	162.62	187.89	136.72	162.75	189.51	135.64	0.01	0.02	0.00
Antigua and Barbuda	248.81	263.55	233.04	241.72	256.76	225.32	-0.03	-0.03	-0.04
Argentina	189.59	202.01	176.69	185.11	194.79	175.02	-0.03	-0.04	-0.01
Armenia	307.97	342.11	271.8	416.01	448.98	378.05	0.33	0.29	0.37
Australia	152.97	167.14	138.72	155.17	167.02	143.3	0.01	-0.01	0.03
Austria	229.35	243.77	213.63	245.58	246.36	244.76	0.06	0.01	0.13
Azerbaijan	280.38	316.58	242.60	329.54	362.35	296.34	0.17	0.14	0.21
Bahrain	171.16	188.69	158.26	197.56	232.8	175.97	0.15	0.23	0.10
Bangladesh	261.64	256.11	266.91	294.71	284.41	304.82	0.14	0.12	0.16
Barbados	249.04	265.96	230.81	299.01	314.88	281.75	0.19	0.18	0.21
Belarus	425.95	494.37	348.46	521.96	589.63	443.47	0.21	0.18	0.26
Belgium	175.24	188.66	161.2	199.23	208.47	189.64	0.13	0.10	0.16
Belize	155.72	164.12	147.46	171.05	182.25	159.78	0.10	0.11	0.09
Benin	207.41	209.11	205.6	225.75	223.17	228.34	0.10	0.07	0.12
Bermuda	212.01	223.41	200.47	240.14	254.56	225.45	0.13	0.13	0.12
Bhutan	190.66	182.46	198.28	206.88	190.8	220.73	0.07	0.03	0.10
Bolivia	124.41	137.01	111.89	149.42	164.58	134.32	0.18	0.18	0.18
Bosnia and Herzegovina	294.87	346.64	243.04	418.55	469.34	367.29	0.40	0.34	0.49
Botswana	165.19	174.66	155.51	206.04	221.69	190.37	0.24	0.26	0.22
Brazil	134.25	137.38	131.06	135.11	136.03	134.15	0.00	-0.02	0.01
Brunei	121.96	133.94	111.23	172.65	195.63	151.03	0.37	0.41	0.32
Bulgaria	458.74	488.32	428.32	538.22	585.57	488.52	0.17	0.19	0.13
Burkina Faso	196.52	201.23	191.58	175.76	180.89	170.56	-0.10	-0.10	-0.10
Burundi	238.35	264.53	211.25	226.69	243.69	209.33	-0.02	-0.06	0.03
Cambodia	203.95	200.32	207.84	253.30	258.82	247.49	0.27	0.31	0.23
Cameroon	199.43	200.12	198.73	193.17	191.44	194.9	-0.01	-0.03	0.00
Canada	102.86	113.36	92.17	126.59	133.53	119.55	0.22	0.17	0.27
Cape Verde	269.94	275.81	263.45	303.01	313.02	292.72	0.12	0.13	0.11
Central African Republic	226.77	253.34	199.30	214.02	242.00	185.28	-0.05	-0.04	-0.07
Chad	179.2	179.74	178.66	171.49	169.05	173.92	-0.04	-0.06	-0.02
Chile	169.81	187.24	151.91	212.82	231.95	193.18	0.23	0.22	0.25
China	279.18	266.70	291.02	344.59	286.88	398.95	0.26	0.09	0.41
Colombia	72.11	77.87	66.27	91.44	97.13	85.58	0.26	0.24	0.28
Comoros	211.02	230.95	191.21	221.37	240.72	202.37	0.05	0.04	0.06
Congo	236.14	276.04	196.04	219.05	263.83	174.33	-0.07	-0.04	-0.11
Costa Rica	59.68	62.98	56.44	81.49	84.89	78.1	0.35	0.33	0.37
Cote d'Ivoire	238.19	216.53	258.15	239.27	223.75	254.25	0.01	0.04	0.00
Croatia	419.57	483.40	351.75	506.11	560.09	448.18	0.20	0.15	0.26
Cuba	273.29	291.91	254.95	330.89	347.21	314.73	0.20	0.18	0.22
Cyprus	115.34	114.56	116.14	169.36	160.26	179.08	0.44	0.37	0.51
Czech Republic	349.23	396.43	299.20	322.79	363.82	280.45	-0.08	-0.09	-0.07
Democratic Republic of the Congo	157.06	185.60	127.82	147.76	169.55	125.87	-0.06	-0.08	-0.01
Denmark	200.2	206.89	193.31	208.97	209.1	208.84	0.04	0.01	0.07
Djibouti	188.81	203.84	173.95	265.53	287.87	243.39	0.40	0.41	0.39
Dominica	237.4	255.27	219.4	269.27	290.3	247.91	0.14	0.14	0.14
Dominican Republic	175.07	183.2	167.05	210.38	218.73	201.96	0.20	0.19	0.21
Ecuador	123.39	132.82	114.08	140.6	150.92	130.28	0.14	0.14	0.15
Egypt	173.44	185.90	161.06	206.47	218.79	194.40	0.19	0.17	0.21
El Salvador	57.39	60.65	53.98	67.91	72.48	62.73	0.19	0.19	0.17
Equatorial Guinea	207.41	240.13	176.11	216.56	239.08	195.15	0.05	0.00	0.11
Eritrea	172.81	192.03	153.35	197.84	223.38	172.39	0.15	0.17	0.13
Estonia	399.44	460.41	330.05	420.76	488.01	341.79	0.04	0.05	0.02
Ethiopia	202.17	218.91	185.32	264.02	292.64	235.3	0.32	0.35	0.28
Federated States of Micronesia	285.27	287.33	283.29	309.52	315.54	303.79	0.08	0.09	0.07
Fiji	315.57	321.90	309.44	367.09	383.87	350.87	0.15	0.19	0.12
Finland	193.9	208.75	178.15	233.64	237.53	229.62	0.19	0.13	0.27
France	151.28	157.18	145.05	184.7	187.79	181.41	0.21	0.19	0.24
Gabon	262.80	302.99	220.94	224.24	263.82	185.55	-0.15	-0.13	-0.16
Georgia	490.07	528.53	447.64	565.82	583.08	546.95	0.15	0.10	0.21
Germany	176.5	190.73	161.21	215.02	210.59	219.6	0.21	0.10	0.34
Ghana	207.81	225.49	190.7	225.6	237.85	213.23	0.10	0.06	0.14

Greece	180.33	182.11	178.51	225.05	223.65	226.53	0.24	0.22	0.26
Greenland	114.18	122.70	106.81	157.97	155.73	159.9	0.36	0.26	0.46
Grenada	252.11	277.23	226.34	261.17	281.56	240.90	0.05	0.03	0.09
Guam	316.91	325.11	309.74	448.75	457.99	439.77	0.43	0.41	0.44
Guatemala	53.9	56.53	51.23	58.87	62.99	54.57	0.12	0.13	0.09
Guinea	205.56	212.58	198.38	213.44	213.03	213.86	0.04	0.00	0.08
Guinea-Bissau	223.83	228.78	218.76	223.23	226.92	219.50	0.01	0.00	0.02
Guyana	217.67	229.80	205.15	288.02	306.84	269.38	0.33	0.34	0.32
Haiti	171.02	183.45	158.22	202.19	219.80	184.18	0.21	0.22	0.21
Honduras	72.35	77.38	67.35	78.74	78.36	79.12	0.09	0.01	0.18
Hungary	443.50	486.20	397.32	439.04	497.43	374.68	-0.02	0.02	-0.06
Iceland	110.73	111.82	109.66	129.8	126.65	133.03	0.16	0.12	0.19
India	200.71	190.00	210.68	225.6	199.86	249.51	0.12	0.05	0.19
Indonesia	283.02	266.12	299.77	339.54	324.21	354.65	0.22	0.23	0.20
Iran	146.81	154.41	139.49	188.88	204.93	173.07	0.27	0.30	0.22
Iraq	182.73	200.5	165.28	190.09	213.1	167.65	0.04	0.06	0.01
Ireland	143.44	149.63	137.2	153.42	151.87	155.02	0.06	0.01	0.12
Israel	107.37	107.35	107.39	115.39	113.37	117.45	0.07	0.05	0.08
Italy	173.72	182.85	164.03	192.15	192.81	191.44	0.10	0.05	0.15
Jamaica	225.06	240.60	209.12	236.9	248.9	224.81	0.04	0.02	0.06
Japan	275.79	278.61	272.87	314.12	291.51	337.92	0.12	0.03	0.20
Jordan	145.92	165.66	128.06	147.82	168.68	127.96	0.00	0.01	-0.01
Kazakhstan	357.77	405.13	307.44	416.95	451.69	379.72	0.17	0.11	0.24
Kenya	136.28	142.71	129.81	142.96	145.16	140.75	0.05	0.02	0.09
Kiribati	324.67	320.00	329.44	365.70	349.92	381.89	0.13	0.10	0.17
Kuwait	148.47	152.7	144.77	175.59	190.35	164.07	0.17	0.23	0.12
Kyrgyzstan	329.57	366.67	290.87	329.95	347.28	312.25	0.00	-0.05	0.07
Laos	221.84	217.25	226.44	252.08	249.39	254.81	0.15	0.16	0.15
Latvia	414.83	488.11	330.42	442.11	522.07	348.98	0.05	0.06	0.04
Lebanon	194.27	233.05	154.43	216.22	254.95	177.89	0.10	0.08	0.14
Lesotho	175.55	183.46	167.17	197.95	216.32	179.21	0.12	0.18	0.07
Liberia	188.44	187.37	189.52	213.47	215.44	211.53	0.14	0.15	0.12
Libya	139.51	153.68	126.82	207.06	234.5	179.95	0.47	0.51	0.41
Lithuania	364.29	439.02	281.13	494.44	590.44	382.8	0.34	0.33	0.34
Luxembourg	182.76	196.04	168.95	194.9	201.85	187.93	0.07	0.03	0.11
Macedonia	325.34	366.14	285.01	430.29	476.61	383.56	0.31	0.29	0.33
Madagascar	242.73	254.22	231.20	275.25	295.05	255.33	0.15	0.17	0.12
Malawi	174.87	196.3	152.69	209.96	233.8	186.02	0.22	0.21	0.24
Malaysia	197.35	189.9	204.57	242.18	232.21	252.34	0.22	0.22	0.23
Maldives	150.85	126.73	173.75	164.92	154.78	174.99	0.09	0.22	0.01
Mali	211.72	230.00	193.04	180.13	189.1	171.33	-0.14	-0.17	-0.10
Malta	134.56	136.07	133	215.05	205.56	224.61	0.57	0.49	0.65
Marshall Islands	252.86	243.69	261.63	304.15	296.55	311.49	0.20	0.21	0.19
Mauritania	222.24	224.84	219.63	216.66	223.32	210.09	-0.02	0.00	-0.04
Mauritius	295.48	283.74	307.26	371.72	356.16	387.66	0.24	0.25	0.24
Mexico	55.77	60.67	50.81	55.95	55.66	56.24	0.00	-0.09	0.10
Moldova	352.75	399.76	301.17	391.70	435.78	344.04	0.09	0.07	0.13
Mongolia	285.00	305.91	263.59	445.79	471.45	419.61	0.55	0.53	0.58
Montenegro	293.07	342.67	243.05	434.82	496.57	371.60	0.47	0.44	0.51
Morocco	153.16	175.37	130.88	196.42	227.23	164.9	0.27	0.28	0.25
Mozambique	213.43	228.98	196.47	272.09	296.82	246.22	0.28	0.30	0.26
Myanmar	280.59	258.50	303.58	348.15	328.34	368.86	0.27	0.29	0.25
Namibia	185.7	194.87	176.14	216.86	234.3	198.48	0.17	0.20	0.13
Nepal	197.81	192.84	202.85	235.04	222.01	248.89	0.20	0.16	0.24
Netherlands	151.2	162.09	140.07	181.97	183.93	179.98	0.19	0.13	0.27
New Zealand	162.01	177.98	145.56	190.98	204.79	176.64	0.16	0.13	0.19
Nicaragua	58.96	63.04	54.81	75.3	80.99	69.44	0.29	0.29	0.28
Niger	172.59	174.68	170.42	178.88	173.64	184.05	0.05	0.00	0.10
Nigeria	182.02	185.57	178.54	167.11	166.58	167.62	-0.08	-0.10	-0.06
North Korea	271.54	296.39	245.23	364.60	375.37	353.34	0.35	0.27	0.45
Northern Mariana Islands	293.68	271.22	313.86	294.22	264.86	323.78	0.02	-0.01	0.06

Norway	170.22	175.21	165.12	164.8	166.68	162.94	-0.04	-0.05	-0.03
Oman	120.18	136.75	106.96	147.54	172.4	134.93	0.22	0.25	0.25
Pakistan	230.04	212.33	246.48	248.04	230.5	264.64	0.08	0.09	0.08
Palestine	134.84	152.48	117.64	158.42	178.89	138.51	0.16	0.16	0.16
Panama	76.68	77.41	75.96	99.08	100.14	98.04	0.32	0.31	0.32
Papua New Guinea	269.01	278.97	259.49	339.97	366.74	314.24	0.27	0.32	0.21
Paraguay	122.07	125.61	118.61	179.07	178.46	179.66	0.45	0.41	0.50
Peru	117.95	128.5	107.40	138.4	151.45	125.33	0.17	0.18	0.17
Philippines	185.79	173.92	197.41	252.22	243.55	260.73	0.37	0.41	0.33
Poland	310.01	351.79	266.07	361.92	412.41	307.59	0.16	0.16	0.14
Portugal	215.65	217.34	213.84	249.68	246.23	253.35	0.14	0.12	0.16
Puerto Rico	209.38	220.08	198.01	256.45	274.11	237.36	0.22	0.23	0.19
Qatar	140.99	147.81	137.62	141.93	165.15	133.24	-0.01	0.10	-0.04
Romania	412.59	462.47	361.27	495.22	550.59	436.28	0.19	0.18	0.20
Russia	422.94	495.96	340.01	531.85	593.53	459.40	0.25	0.19	0.34
Rwanda	201.71	220.08	182.68	219.71	249.17	187.67	0.09	0.14	0.03
Saint Lucia	242.95	258.43	227.00	263.39	280.61	245.51	0.10	0.09	0.10
Saint Vincent and the Grenadines	228.32	252.83	203.57	253.61	271.15	236.40	0.12	0.07	0.17
Samoa	328.01	337.41	319.36	353.89	365.55	342.94	0.07	0.08	0.07
Sao Tome and Principe	210.73	215.43	205.94	216.98	223.24	210.67	0.03	0.04	0.02
Saudi Arabia	117.84	125.2	112.06	143.1	140.25	145.28	0.19	0.10	0.27
Senegal	192.48	191.9	193.07	187.52	193.37	181.46	-0.02	0.01	-0.05
Serbia	371.04	422.49	318.21	460.18	516.35	401.26	0.23	0.21	0.25
Seychelles	254.31	237.45	271.25	274.56	260.85	287.9	0.07	0.09	0.05
Sierra Leone	220.8	211.57	230.21	236.3	238.15	234.42	0.09	0.14	0.04
Singapore	173.93	196.43	152.02	231.67	259.45	202.91	0.30	0.30	0.30
Slovakia	307.31	345.42	267.41	349.09	387.69	308.35	0.12	0.11	0.14
Slovenia	305.56	350.2	258.23	353.5	394.88	311.38	0.14	0.12	0.19
Solomon Islands	300.87	308.02	294.09	330.55	341.30	320.13	0.10	0.11	0.09
Somalia	202.12	223.19	180.66	212.18	233.90	190.25	0.05	0.05	0.05
South Africa	248.33	242.51	254.29	277.64	272.99	282.47	0.11	0.12	0.11
South Korea	241.89	272.76	211.15	346.92	382.21	311.2	0.39	0.36	0.42
South Sudan	208.91	230.91	186.71	240.46	266.26	214.73	0.15	0.15	0.15
Spain	166.76	175.5	157.64	198.61	208.64	188.31	0.18	0.18	0.18
Sri Lanka	192.08	181.72	202.24	308.76	304.29	313.56	0.63	0.69	0.58
Sudan	172.75	193.49	152.18	194.62	223.21	166.25	0.13	0.16	0.10
Suriname	220.91	233.97	208.26	268.76	281.94	255.64	0.21	0.20	0.23
Swaziland	146.61	151.95	140.65	180.48	196.33	164.3	0.23	0.29	0.16
Sweden	205.3	195.34	215.49	179.13	164.56	193.73	-0.14	-0.16	-0.12
Switzerland	155.79	161.86	149.44	168.47	173.4	163.42	0.07	0.07	0.08
Syria	160.12	169.26	151.26	202.3	215.42	189.5	0.26	0.27	0.24
Taiwan	261.38	250.48	271.49	305.72	299.37	312.09	0.19	0.21	0.19
Tajikistan	237.58	269.03	205.76	214.92	235.18	195.13	-0.10	-0.13	-0.05
Tanzania	179.75	197.84	161.33	202.88	220.93	184.6	0.15	0.13	0.17
Thailand	228.00	208.98	247.37	331.70	307.75	356.33	0.45	0.47	0.44
The Bahamas	216.23	230.73	201.53	261.65	279.9	242.62	0.20	0.20	0.19
The Gambia	189.51	180.89	198.02	193.94	185.3	202.75	0.03	0.03	0.03
Timor-Leste	174.71	171.15	178.18	215.7	216.7	214.73	0.25	0.28	0.22
Togo	190.5	190.9	190.1	193	189.35	196.72	0.02	0.00	0.05
Tonga	279.09	305.84	252.98	302.29	323	281.7	0.08	0.05	0.11
Trinidad and Tobago	218.8	228.54	208.99	271.34	279.27	263.20	0.23	0.21	0.25
Tunisia	184.15	193.95	174.46	242.3	260.14	224.05	0.30	0.32	0.27
Turkey	162.64	184.94	139.79	186.7	213.13	159.34	0.14	0.14	0.13
Turkmenistan	262.76	292.29	232.40	331.90	350.43	312.73	0.25	0.19	0.34
Uganda	182.44	201.62	163.01	177.65	192.55	162.73	-0.01	-0.03	0.02
Ukraine	463.30	534.69	380.22	525.38	591.03	447.84	0.13	0.10	0.17
United Arab Emirates	170.41	178.64	166.13	185.76	203.7	179.22	0.08	0.13	0.06
United Kingdom	301.77	334.19	267.47	304.22	330.92	276.84	0.00	-0.01	0.03
United States	118.64	125.91	110.99	110.43	112.24	108.55	-0.08	-0.11	-0.04
Uruguay	237.15	253.29	220.03	231.94	251.8	210.66	-0.03	-0.01	-0.05
Uzbekistan	220.46	244.16	196.23	253.03	268.92	236.63	0.14	0.09	0.20

Vanuatu	364.83	361.90	367.59	412.76	423.97	401.83	0.09	0.14	0.05
Venezuela	79.74	84.24	75.31	104.46	108.64	100.25	0.29	0.28	0.31
Vietnam	274.66	251.02	299.02	312.58	291.74	333.87	0.17	0.18	0.15
Virgin Islands, U.S.	203.31	214.38	191.58	304.07	313.37	293.84	0.48	0.45	0.51
Yemen	157.75	182.06	133.00	185.93	217.25	155.25	0.18	0.19	0.17
Zambia	184.04	205.57	162.15	227.56	245.66	209.41	0.25	0.21	0.31
Zimbabwe	156.85	161.32	152.34	136.04	146.4	125.38	-0.13	-0.09	-0.17
**Hypertensive heart disease**									
Afghanistan	9.48	12.69	6.42	11.61	15.7	7.78	0.23	0.24	0.22
Albania	27.64	32.34	23.17	66.86	76.83	56.73	1.40	1.36	1.44
Algeria	30.45	41.63	19.55	54.21	71.73	36.93	0.75	0.70	0.86
American Samoa	30.57	32.31	28.93	28.43	33.02	23.86	-0.07	0.02	-0.18
Andorra	55.63	67.86	42.74	68.14	80.55	55.21	0.21	0.18	0.27
Angola	49.49	45.81	53.26	48.24	44.41	52.11	-0.02	-0.02	-0.01
Antigua and Barbuda	128.61	143.1	113.11	144.36	156.58	131.03	0.12	0.09	0.16
Argentina	82.48	102.24	61.94	89.61	115.14	62.98	0.08	0.12	0.02
Armenia	27.69	33.3	21.74	58.17	69.22	45.44	1.07	1.05	1.06
Australia	45.24	49.28	41.17	60.78	62.2	59.35	0.33	0.25	0.43
Austria	62.28	81.39	41.46	69.58	85.22	53.16	0.11	0.04	0.26
Azerbaijan	18.38	23.26	13.29	32.29	37.86	26.66	0.74	0.61	0.99
Bahrain	13.78	19.14	9.84	17.15	26.92	11.17	0.24	0.40	0.13
Bangladesh	35.72	33.41	37.93	66.85	62.59	71.04	0.89	0.88	0.90
Barbados	142.31	165.94	116.84	214.78	236.02	191.7	0.50	0.41	0.62
Belarus	32.49	42.72	20.91	46.53	62.29	28.26	0.42	0.45	0.34
Belgium	37.07	48.61	25	41.73	53.16	29.84	0.12	0.09	0.18
Belize	61.18	60.96	61.39	65.22	63.48	66.98	0.07	0.04	0.10
Benin	55.85	52.96	58.92	53.51	49.29	57.75	-0.03	-0.06	-0.01
Bermuda	82.29	87.83	76.67	118.91	130.37	107.23	0.44	0.47	0.39
Bhutan	37.62	35.55	39.53	64.62	60.1	68.51	0.70	0.67	0.71
Bolivia	9.94	12.1	7.8	20.33	24.01	16.66	1.01	0.95	1.10
Bosnia and Herzegovina	34.85	43.39	26.3	80.1	100.25	59.77	1.27	1.29	1.24
Botswana	32.69	30.86	34.56	51.9	45.91	57.89	0.58	0.48	0.66
Brazil	75.98	87.54	64.25	124.09	140.09	107.54	0.62	0.59	0.66
Brunei	22.92	23.14	22.73	31.82	33.84	29.91	0.35	0.41	0.28
Bulgaria	97.08	111.78	81.96	151.25	189.44	111.18	0.55	0.68	0.35
Burkina Faso	51.68	46.98	56.6	45.02	43	47.07	-0.12	-0.08	-0.16
Burundi	44.14	40.86	47.53	42.71	37.2	48.34	0.00	-0.07	0.06
Cambodia	20.3	24.03	16.29	30.35	36.57	23.83	0.53	0.54	0.51
Cameroon	45.86	41.52	50.25	49.47	44.26	54.68	0.10	0.08	0.11
Canada	56.77	67.18	46.19	83.68	96.45	70.74	0.46	0.43	0.51
Cape Verde	83.14	80.28	86.3	113.45	115.35	111.5	0.36	0.43	0.29
Central African Republic	72.11	67.55	76.83	70.27	67.66	72.94	-0.02	0.00	-0.05
Chad	57.67	50.79	64.66	51.05	44.34	57.75	-0.11	-0.13	-0.10
Chile	47.21	58.02	36.12	83.6	104.25	62.39	0.74	0.77	0.70
China	56.66	60.93	52.61	125.47	132.2	119.13	1.26	1.20	1.33
Colombia	50.55	61.39	39.57	104.17	125.06	82.64	1.04	1.02	1.07
Comoros	53.47	49.34	57.57	54.56	49.31	59.72	0.02	0.00	0.04
Congo	66.06	58.21	73.94	69.73	62.53	76.92	0.06	0.08	0.04
Costa Rica	65.01	72.82	57.33	125.48	139.4	111.58	0.91	0.89	0.92
Cote d'Ivoire	50.36	38.26	61.51	55.05	39.46	70.1	0.10	0.04	0.15
Croatia	78.28	96.33	59.11	136.88	168.67	102.76	0.74	0.74	0.72
Cuba	116.93	116.98	116.88	202.19	207.7	196.73	0.71	0.76	0.67
Cyprus	40.63	43.52	37.67	42.46	47.03	37.57	0.02	0.06	-0.03
Czech Republic	66.84	88.92	43.44	116.84	151.79	80.76	0.73	0.70	0.84
Democratic Republic of the Congo	61.25	58.51	64.06	61.9	57.26	66.56	0.01	-0.02	0.04
Denmark	41.55	50.78	32.05	41.55	48.3	34.68	-0.01	-0.05	0.07
Djibouti	51.2	48.51	53.87	76.04	69.45	82.57	0.48	0.43	0.53
Dominica	119.81	122.95	116.64	179.42	190.4	168.27	0.51	0.55	0.46
Dominican Republic	63.7	59.79	67.56	101.42	101.48	101.36	0.59	0.69	0.50
Ecuador	38.39	43.59	33.25	68.47	77.51	59.43	0.79	0.78	0.79
Egypt	34.99	47.71	22.35	38.28	51.32	25.52	0.09	0.07	0.14

El Salvador	42.8	52.37	32.77	81.88	97.58	64.13	0.92	0.86	0.98
Equatorial Guinea	78.49	72.53	84.18	74.18	61.21	86.51	-0.05	-0.15	0.03
Eritrea	32.55	32.15	32.96	44.19	43.35	45.02	0.37	0.35	0.38
Estonia	48.58	66.01	28.76	81.18	109.63	47.77	0.65	0.65	0.64
Ethiopia	48.89	40.69	57.15	48.49	42.93	54.07	0.00	0.06	-0.04
Federated States of Micronesia	21.68	24.71	18.77	29.02	34.61	23.7	0.33	0.39	0.25
Fiji	20.83	26.65	15.19	41.98	55.53	28.88	1.00	1.07	0.89
Finland	41.91	55.41	27.6	65.83	82.76	48.38	0.55	0.48	0.72
France	56.61	72.72	39.62	67.79	83.4	51.17	0.19	0.14	0.28
Gabon	120.34	113.28	127.68	111.67	106.98	116.25	-0.07	-0.06	-0.09
Georgia	50.18	65.35	33.45	83.32	105.91	58.6	0.65	0.61	0.74
Germany	56.71	75.57	36.44	73.95	90.72	56.62	0.29	0.19	0.53
Ghana	39.3	33.39	45.01	56.33	50.98	61.73	0.45	0.54	0.40
Greece	51.13	58.8	43.27	69.75	82.86	55.98	0.35	0.40	0.28
Greenland	35.4	38.67	32.58	57.83	60.1	55.86	0.60	0.54	0.67
Grenada	100.22	113.8	86.28	109.16	125.25	93.17	0.11	0.11	0.10
Guam	43.12	52.06	35.31	82.95	96.41	69.87	0.94	0.86	1.01
Guatemala	29.62	35.71	23.43	45.36	56.2	34.04	0.56	0.60	0.49
Guinea	61.08	52.49	69.87	62.14	52.67	71.58	0.02	0.00	0.03
Guinea-Bissau	59.86	51.73	68.19	56.56	46.91	66.33	-0.04	-0.09	-0.01
Guyana	67.7	68.25	67.14	85.55	90.7	80.46	0.27	0.33	0.21
Haiti	65.91	62.7	69.22	73.17	73.42	72.92	0.14	0.19	0.09
Honduras	44.08	46.97	41.21	58	62.06	53.95	0.32	0.32	0.32
Hungary	80.97	102.5	57.68	133.43	174.17	88.51	0.64	0.69	0.53
Iceland	31.67	34.67	28.69	37.74	40.78	34.64	0.18	0.17	0.19
India	42.15	44.59	39.89	69.88	75.74	64.43	0.66	0.70	0.62
Indonesia	31.14	35.88	26.43	46.79	54.77	38.93	0.52	0.54	0.50
Iran	31.54	41.07	22.36	41.47	53.6	29.52	0.29	0.28	0.30
Iraq	25.45	33.31	17.74	23.3	31.41	15.39	-0.09	-0.06	-0.13
Ireland	27.1	32.58	21.58	29.2	34.25	24.01	0.07	0.04	0.10
Israel	25.1	27.6	22.59	34.52	41.15	27.77	0.37	0.48	0.22
Italy	78.84	94.23	62.5	93.2	113.76	71.34	0.17	0.20	0.13
Jamaica	149.24	150.78	147.66	193.99	191.66	196.34	0.29	0.26	0.31
Japan	108.33	140.8	74.7	209.58	275.04	140.64	0.89	0.93	0.83
Jordan	33.1	42.5	24.6	40.14	53.8	27.14	0.20	0.25	0.09
Kazakhstan	37.63	50.66	23.78	47.1	60.15	33.11	0.25	0.19	0.39
Kenya	59.9	54.87	64.97	65.6	62.62	68.59	0.10	0.15	0.06
Kiribati	18.49	22.18	14.73	20.42	25.25	15.47	0.11	0.14	0.06
Kuwait	25.08	30.35	20.47	32.76	43.79	24.15	0.29	0.42	0.16
Kyrgyzstan	26.92	33.34	20.22	26.62	30.64	22.51	-0.01	-0.08	0.11
Laos	23.91	27.84	19.98	26.17	30.42	21.89	0.11	0.11	0.12
Latvia	31.16	42.37	18.25	49	67.07	27.96	0.56	0.57	0.51
Lebanon	33.04	45.84	19.89	48.83	67.49	30.36	0.46	0.46	0.51
Lesotho	47.31	42.79	52.09	48.78	47.83	49.75	0.03	0.12	-0.05
Liberia	50.82	39.89	61.82	53.88	44.39	63.22	0.06	0.12	0.03
Libya	24.69	33.23	17.04	30.41	41.67	19.27	0.22	0.24	0.13
Lithuania	35.47	46.5	23.2	62.71	84.4	37.48	0.75	0.80	0.59
Luxembourg	44.06	58.78	28.75	39.77	52.59	26.91	-0.10	-0.10	-0.07
Macedonia	50.9	56.63	45.23	91.56	109.27	73.69	0.78	0.91	0.61
Madagascar	54.59	43.77	65.44	52.91	46.51	59.34	-0.02	0.07	-0.08
Malawi	42.01	37.85	46.32	48.85	45.08	52.64	0.18	0.21	0.16
Malaysia	24.38	26.87	21.96	39.17	41.57	36.73	0.60	0.54	0.67
Maldives	17.18	15.37	18.9	35.71	36.23	35.2	1.08	1.35	0.87
Mali	62.33	50.95	73.95	50.4	42.67	57.99	-0.18	-0.15	-0.20
Malta	28.43	33.03	23.67	42.65	51.49	33.74	0.47	0.53	0.39
Marshall Islands	17.43	20.75	14.25	24.25	29.33	19.35	0.39	0.41	0.35
Mauritania	55.35	47.36	63.39	66.52	57.6	75.33	0.21	0.22	0.20
Mauritius	39.33	49.4	29.22	89.6	106.53	72.25	1.25	1.14	1.43
Mexico	45.1	55.5	34.6	77.49	93.19	61.64	0.71	0.67	0.77
Moldova	26.35	32.44	19.67	38.99	48.54	28.66	0.46	0.48	0.44
Mongolia	18.27	16.69	19.88	20.45	20.16	20.74	0.11	0.20	0.04

Montenegro	48.46	63.09	33.7	62.23	75.59	48.55	0.27	0.19	0.42
Morocco	33.54	44.85	22.18	55.16	72.25	37.67	0.63	0.60	0.68
Mozambique	38.59	35.63	41.82	38.52	36.05	41.1	0.00	0.02	-0.01
Myanmar	34.84	44.81	24.46	52.68	68.43	36.21	0.55	0.55	0.53
Namibia	42.94	38.35	47.73	45.05	41.02	49.3	0.05	0.07	0.04
Nepal	38.01	41.45	34.52	57.48	57.46	57.49	0.52	0.39	0.69
Netherlands	29.4	39.47	19.09	39.42	48.69	29.99	0.33	0.23	0.55
New Zealand	32.87	42.51	22.93	41.49	49.7	32.96	0.24	0.15	0.41
Nicaragua	42.65	52.12	33.04	82.11	96.88	66.92	0.94	0.87	1.05
Niger	42.55	35.17	50.18	48.53	38.78	58.12	0.15	0.11	0.18
Nigeria	62.27	54.07	70.3	70.05	59.45	80.26	0.13	0.10	0.14
North Korea	61.62	81.11	40.98	113.96	149.51	76.8	0.86	0.85	0.89
Northern Mariana Islands	18.46	21.38	15.84	16.88	18.18	15.58	-0.07	-0.14	0.01
Norway	40.91	49.8	31.83	39.26	47.28	31.33	-0.05	-0.05	-0.03
Oman	27.77	39.29	18.57	22.72	37	15.48	-0.18	-0.06	-0.17
Pakistan	43.99	41.43	46.36	52.7	52.97	52.44	0.20	0.28	0.14
Palestine	13.42	17.46	9.49	16.97	22.17	11.91	0.25	0.26	0.24
Panama	53.55	60.51	46.74	91.74	104.45	79.1	0.74	0.75	0.73
Papua New Guinea	13.81	16.22	11.51	16.05	18.77	13.43	0.16	0.16	0.17
Paraguay	98.62	98.86	98.38	136.49	137.6	135.41	0.37	0.38	0.36
Peru	24.41	29.25	19.56	51.33	61.75	40.9	1.11	1.11	1.10
Philippines	36.19	42.52	29.99	49.67	59.63	39.89	0.38	0.41	0.34
Poland	59.45	78.46	39.46	109.76	146.46	70.27	0.83	0.85	0.76
Portugal	45.41	55.44	34.62	55.12	68.79	40.55	0.20	0.23	0.15
Puerto Rico	147.02	153.42	140.22	223.32	248.88	195.71	0.51	0.61	0.39
Qatar	13.42	18.82	10.76	14.63	24.05	11.11	0.08	0.26	0.02
Romania	70.64	87.66	53.13	131.87	169.96	91.33	0.85	0.93	0.71
Russia	30.3	44.19	14.53	43.27	60.28	23.29	0.42	0.36	0.59
Rwanda	39.44	36.78	42.18	52.43	51.28	53.68	0.34	0.40	0.28
Saint Lucia	111.78	115.61	107.83	158.45	165.49	151.15	0.44	0.44	0.43
Saint Vincent and the Grenadines	93.7	101.22	86.11	136.89	140.82	133.03	0.47	0.39	0.56
Samoa	26.23	30.62	22.18	41.35	49.31	33.87	0.57	0.60	0.52
Sao Tome and Principe	82.16	71.1	93.43	70.94	63.65	78.28	-0.14	-0.10	-0.16
Saudi Arabia	11.51	19.14	5.5	16.52	27.52	8.07	0.41	0.41	0.44
Senegal	58.19	47.31	69.17	62.45	52.98	72.27	0.08	0.12	0.06
Serbia	49.05	57.55	40.33	83.13	99.95	65.49	0.68	0.72	0.61
Seychelles	85.16	110.95	59.25	114.74	148.86	81.53	0.34	0.34	0.37
Sierra Leone	50.88	38.57	63.45	46.89	37.21	56.77	-0.06	-0.02	-0.08
Singapore	54.38	63.09	45.89	114.36	131.71	96.4	1.06	1.05	1.05
Slovakia	53.16	67.78	37.86	85.34	112.87	56.3	0.59	0.65	0.47
Slovenia	78.66	102.5	53.38	156.14	198.62	112.89	0.96	0.92	1.08
Solomon Islands	16.3	16.18	16.42	20.25	21.69	18.86	0.24	0.34	0.15
Somalia	49.97	43.59	56.45	44.5	39.11	49.93	-0.11	-0.10	-0.11
South Africa	65.74	64.3	67.22	79.44	81.63	77.17	0.20	0.26	0.14
South Korea	60.94	77.63	44.33	117.57	147.64	87.14	0.87	0.85	0.90
South Sudan	47.79	41.23	54.42	56.2	48.89	63.49	0.18	0.19	0.17
Spain	49.15	63.03	34.68	59.46	76.25	42.23	0.20	0.20	0.20
Sri Lanka	46.77	52.51	41.14	68.78	81.99	54.58	0.49	0.58	0.35
Sudan	18.27	25.04	11.57	20.29	27.71	12.93	0.11	0.11	0.12
Suriname	76.17	82.78	69.78	112.58	126.91	98.31	0.47	0.53	0.41
Swaziland	33.31	28.96	38.16	43.61	39.24	48.06	0.31	0.35	0.25
Sweden	35.21	43.54	26.69	35.76	44.46	27.05	0.00	0.02	0.00
Switzerland	51.38	62.83	39.39	56.14	66.54	45.5	0.09	0.06	0.14
Syria	16.21	21.54	11.05	20.08	27.72	12.63	0.23	0.28	0.13
Taiwan	90.72	87.76	93.47	223.03	225.82	220.24	1.51	1.60	1.43
Tajikistan	22.57	25.66	19.45	23.33	22.92	23.73	0.03	-0.11	0.22
Tanzania	44.5	39.92	49.15	51.99	44.38	59.68	0.19	0.13	0.24
Thailand	33.21	39.17	27.14	63.31	74.87	51.42	0.90	0.90	0.90
The Bahamas	91.26	101.13	81.25	173	190.79	154.44	0.87	0.86	0.88
The Gambia	48.74	35.2	62.08	48.66	36.74	60.81	0.00	0.04	-0.02
Timor-Leste	17.21	18.65	15.8	31.71	35.48	28.06	0.86	0.92	0.79

Togo	52.28	46.63	58.07	53.2	46.61	59.93	0.03	0.01	0.05
Tonga	25.67	31.12	20.35	37.82	48.54	27.16	0.47	0.56	0.33
Trinidad and Tobago	84.66	88.68	80.6	146.16	156.39	135.66	0.71	0.74	0.67
Tunisia	34.47	46.53	22.56	54.63	75.37	33.39	0.56	0.60	0.46
Turkey	34.6	46.89	22.02	55.92	77.12	33.98	0.61	0.63	0.53
Turkmenistan	20.55	24.07	16.92	25.6	28.36	22.74	0.24	0.17	0.33
Uganda	46.95	41.58	52.38	44.21	39.99	48.43	-0.04	-0.03	-0.06
Ukraine	33.28	45.02	19.62	46.08	61.21	28.2	0.38	0.35	0.43
United Arab Emirates	11.83	17.32	8.97	15.28	21.76	12.91	0.28	0.24	0.42
United Kingdom	27.8	35.67	19.47	31.14	37.45	24.67	0.12	0.05	0.26
United States	127.07	154.73	97.97	142	165.83	117.37	0.11	0.07	0.18
Uruguay	85.36	93.31	76.92	102.33	120.01	83.39	0.19	0.28	0.08
Uzbekistan	33.47	40.55	26.23	44.52	51.05	37.78	0.32	0.25	0.43
Vanuatu	21.42	21.74	21.12	26.86	30.64	23.17	0.21	0.37	0.05
Venezuela	37.03	49.63	24.63	93.21	116.19	70.02	1.49	1.32	1.80
Vietnam	60.16	77.66	42.11	101.45	132.93	69.29	0.73	0.74	0.70
Virgin Islands, U.S.	96.62	97.59	95.6	251.98	247.95	256.4	1.58	1.51	1.65
Yemen	13.8	19.08	8.43	14.37	18.93	9.91	0.04	-0.01	0.17
Zambia	44	39.71	48.36	42.35	39.25	45.45	-0.03	0.00	-0.05
Zimbabwe	33.18	25.87	40.55	40.18	31.85	48.75	0.21	0.23	0.21
**Ischemic heart disease**									
Afghanistan	766.34	682.08	846.56	936.34	844.57	1,022.50	0.23	0.24	0.21
Albania	1,479.22	1,022.39	1,914.29	2,647.10	1,847.77	3,459.31	0.78	0.79	0.80
Algeria	608.14	536.18	678.33	1,214.44	1,040.21	1,386.27	0.97	0.91	1.01
American Samoa	441.24	395.52	484.50	564.38	533.64	594.96	0.27	0.35	0.22
Andorra	2,055.77	1,804.56	2,320.64	2,282.69	1830.88	2,753.47	0.10	0.01	0.17
Angola	226.16	200.60	252.35	210.95	189.88	232.30	-0.06	-0.05	-0.07
Antigua and Barbuda	1,146.08	1,070.28	1,227.16	1,261.34	1,137.25	1,396.67	0.10	0.06	0.14
Argentina	713.52	468.30	968.40	733.67	519.20	957.48	0.03	0.11	-0.01
Armenia	2,301.45	2,263.47	2,341.71	3,641.92	3,525.29	3,776.25	0.56	0.54	0.59
Australia	1,438.97	1,123.35	1,756.30	1,207.58	940.17	1,475.68	-0.17	-0.17	-0.17
Austria	2,257.72	2,041.83	2,492.95	2,264.05	1,778.41	2,773.81	-0.01	-0.13	0.10
Azerbaijan	1,696.51	1,713.02	1,679.28	2,258.05	2,130.11	2,387.50	0.32	0.23	0.41
Bahrain	844.64	684.27	962.59	1,396.54	1164.52	1,538.62	0.64	0.69	0.59
Bangladesh	727.92	603.99	846.34	1,152.40	948.37	1,352.50	0.60	0.58	0.62
Barbados	1,382.91	1,362.06	1,405.39	2,053.91	1,892.04	2,229.84	0.47	0.38	0.57
Belarus	4,111.24	3,667.80	4,613.49	5,629.90	4,783.68	6,611.53	0.36	0.29	0.42
Belgium	2,376.29	2,024.01	2,744.90	2,366.18	1,875.27	2,876.45	-0.01	-0.08	0.03
Belize	586.84	501.50	670.75	655.95	547.27	765.28	0.12	0.09	0.15
Benin	549.03	415.98	690.59	683.37	485.72	881.79	0.25	0.17	0.29
Bermuda	1,023.60	905.58	1,143.11	1,390.71	1,266.34	1,517.47	0.35	0.39	0.32
Bhutan	630.02	505.23	745.97	1,038.01	826.73	1,219.91	0.63	0.62	0.62
Bolivia	402.59	341.11	463.65	635.47	540.34	730.25	0.55	0.56	0.55
Bosnia and Herzegovina	2,022.16	1,620.91	2,423.85	3,750.04	2,876.96	4,631.21	0.83	0.76	0.88
Botswana	568.26	465.25	673.73	649.91	498.09	801.87	0.14	0.07	0.18
Brazil	973.28	782.38	1,167.20	1,627.34	1,308.27	1,957.08	0.66	0.66	0.66
Brunei	289.96	226.67	346.61	446.79	356.28	531.97	0.49	0.52	0.49
Bulgaria	3,808.60	2,888.47	4,754.63	4,585.86	3,760.82	5,451.73	0.20	0.29	0.14
Burkina Faso	706.36	489.12	933.71	667.91	471.57	866.81	-0.05	-0.03	-0.06
Burundi	755.45	635.39	879.72	669.23	518.94	822.79	-0.08	-0.16	-0.03
Cambodia	442.80	387.53	502.01	641.32	596.20	688.71	0.48	0.56	0.41
Cameroon	549.89	382.2	719.69	564.25	393.42	734.98	0.04	0.04	0.04
Canada	1,667.19	1,326.07	2,014.17	1,509.83	1,289.50	1,733.25	-0.10	-0.03	-0.15
Cape Verde	583.51	451.5	729.49	827.54	684.21	974.72	0.41	0.50	0.33
Central African Republic	276.89	243.72	311.18	251.14	221.94	281.12	-0.09	-0.09	-0.09
Chad	590.86	435.78	748.2	541.03	402.42	679.32	-0.08	-0.07	-0.09
Chile	511.26	360.18	666.43	768.78	548.28	995.18	0.48	0.50	0.47
China	663.10	565.65	755.54	1,416.36	1,191.44	1,628.22	1.18	1.13	1.21
Colombia	805.77	816.90	794.49	1,244.51	1,222.07	1,267.63	0.53	0.49	0.58
Comoros	633.61	486.49	779.79	606.74	447.95	762.66	-0.04	-0.08	-0.02
Congo	319.40	282.23	356.76	285.19	252.65	317.69	-0.10	-0.10	-0.11

Costa Rica	846.03	811.78	879.74	1,512.68	1424.06	1,601.18	0.77	0.73	0.80
Cote d'Ivoire	511.09	320.1	687.07	540.07	349.8	723.85	0.07	0.10	0.07
Croatia	3,518.14	2,904.57	4,170.14	4,402.83	3,633.00	5,229.02	0.24	0.24	0.24
Cuba	1,525.01	1,324.99	1,722.03	2,264.46	2,016.13	2,510.45	0.47	0.51	0.44
Cyprus	1,168.07	939.64	1,402.33	1,362.87	1064.1	1,682.25	0.14	0.11	0.17
Czech Republic	3,946.07	3,629.59	4,281.55	4,115.80	3,605.69	4,642.31	0.03	-0.01	0.07
Democratic Republic of the Congo	242.9	224.06	262.2	225.95	199.98	252.05	-0.07	-0.11	-0.04
Denmark	2,617.60	2,143.27	3,105.78	2,340.11	1,700.20	2,990.24	-0.11	-0.21	-0.05
Djibouti	799.68	664.41	933.36	1,036.41	841.64	1,229.44	0.29	0.26	0.31
Dominica	1,078.80	937.80	1,220.85	1,483.48	1,324.79	1,644.68	0.38	0.41	0.36
Dominican Republic	718.72	581.05	854.56	1,239.47	1,081.07	1,398.91	0.72	0.85	0.64
Ecuador	572.91	511.2	633.94	827.88	733.92	921.86	0.45	0.44	0.46
Egypt	1,009.24	886.61	1,131.02	1,311.23	1,108.70	1,509.57	0.30	0.25	0.34
El Salvador	596.18	584.22	608.71	1,053.51	1,047.78	1,059.99	0.77	0.79	0.76
Equatorial Guinea	301.65	267.04	334.76	299.70	230.96	365.08	0.00	-0.13	0.09
Eritrea	551.27	460.59	643.09	606.7	512.74	700.32	0.11	0.12	0.10
Estonia	3,861.36	3,529.26	4,239.28	4,113.60	3,803.27	4,477.98	0.06	0.07	0.05
Ethiopia	928.88	690.04	1,169.24	1,059.65	834.64	1,285.41	0.15	0.22	0.11
Federated States of Micronesia	458.13	429.76	485.28	661.28	617.97	702.53	0.44	0.43	0.44
Fiji	483.18	422.37	542.12	826.63	744.71	905.83	0.70	0.75	0.66
Finland	2,684.26	2,326.78	3,063.54	2,384.36	1,906.67	2,876.72	-0.12	-0.19	-0.08
France	1,566.33	1,291.50	1,856.13	1,497.04	1185.14	1,829.12	-0.05	-0.09	-0.02
Gabon	408.31	375.73	442.26	334.50	299.87	368.36	-0.18	-0.20	-0.17
Georgia	3,541.77	3,577.30	3,502.58	3,910.52	3,890.42	3,932.51	0.10	0.08	0.11
Germany	2,681.89	2,423.26	2,959.76	2,489.69	2,013.11	2,982.45	-0.08	-0.17	-0.01
Ghana	614.43	408.95	813.42	772.50	536.09	1,011.31	0.27	0.32	0.27
Greece	1,930.97	1,478.36	2,395.21	2,221.53	1,714.51	2,753.66	0.14	0.15	0.14
Greenland	682.54	502.98	837.94	842.23	552.68	1,093.28	0.21	0.09	0.27
Grenada	1,025.33	988.51	1,063.11	1,130.31	1,071.05	1,189.20	0.12	0.09	0.14
Guam	573.35	541.29	601.36	1,190.70	1,136.45	1,243.43	1.10	1.11	1.10
Guatemala	466.92	436.92	497.36	598.64	587.86	609.90	0.31	0.37	0.26
Guinea	594.42	403.47	789.64	687.94	460.53	914.28	0.16	0.14	0.16
Guinea-Bissau	599.75	409.62	794.47	665.22	447.05	886.11	0.12	0.10	0.13
Guyana	605.48	514.98	698.92	1,041.84	911.28	1,171.14	0.73	0.77	0.69
Haiti	468.54	391.89	547.46	598.11	518.98	678.97	0.31	0.35	0.29
Honduras	423.81	394.16	453.24	765.66	708.13	823.19	0.81	0.80	0.83
Hungary	4,159.25	3,437.13	4,940.40	4,407.39	3,657.85	5,233.66	0.05	0.06	0.05
Iceland	1,628.75	1,305.95	1,948.71	1,691.70	1263.25	2,129.33	0.03	-0.04	0.08
India	967.92	734.80	1,185.05	1,438.00	1,121.30	1,732.31	0.49	0.53	0.47
Indonesia	780.05	660.83	898.27	1,135.16	919.57	1,347.65	0.48	0.41	0.53
Iran	633.00	484.61	776.00	1,583.23	1,263.48	1,898.19	1.46	1.56	1.41
Iraq	865.61	743.21	985.78	899.38	792.04	1,004.05	0.04	0.06	0.02
Ireland	1,636.41	1,286.81	1,989.10	1,403.17	1012.17	1,805.75	-0.15	-0.22	-0.10
Israel	1,601.26	998.64	2,206.08	1,375.71	854.26	1,905.99	-0.15	-0.15	-0.14
Italy	2,172.62	1,798.35	2,569.73	2,091.55	1,686.30	2,522.40	-0.05	-0.07	-0.03
Jamaica	935.85	820.55	1,054.10	1,280.59	1,107.45	1,455.10	0.35	0.34	0.36
Japan	1,034.13	955.93	1,115.14	1,632.30	1,485.03	1,787.40	0.55	0.53	0.56
Jordan	745.95	636.98	844.56	991.35	848.17	1,127.60	0.31	0.32	0.32
Kazakhstan	2,411.58	2,497.44	2,320.33	2,865.64	2,785.95	2,951.03	0.19	0.12	0.27
Kenya	632.06	504.72	760.29	668.56	549.14	787.96	0.06	0.09	0.04
Kiribati	452.69	447.15	458.35	582.45	572.68	592.48	0.29	0.28	0.30
Kuwait	662.67	433.64	862.99	970.27	766.52	1,129.30	0.44	0.74	0.29
Kyrgyzstan	1,712.65	1,775.06	1,647.56	1,919.85	1,820.85	2,020.96	0.12	0.02	0.23
Laos	447.51	445.24	449.79	586.75	578.24	595.35	0.33	0.31	0.35
Latvia	4,055.08	3,471.13	4,727.69	4,333.30	3,834.83	4,913.83	0.06	0.09	0.03
Lebanon	1,134.14	957.45	1,315.68	1,571.87	1,311.13	1,829.98	0.37	0.35	0.37
Lesotho	766.37	629.41	911.39	653.36	590.44	717.55	-0.15	-0.06	-0.22
Liberia	531.78	340.14	724.5	728.68	492.32	961.22	0.38	0.45	0.33
Libya	544.98	462.10	619.21	1,149.85	1,018.63	1,279.51	1.09	1.18	1.06
Lithuania	4,310.51	3,697.75	4,992.46	4,949.82	4,335.35	5,664.43	0.13	0.16	0.12
Luxembourg	1,465.23	1,380.96	1,552.95	1,393.16	1,211.18	1,575.87	-0.05	-0.12	0.01

Macedonia	2,057.48	1,432.50	2,675.13	2,948.16	2,151.13	3,752.21	0.42	0.49	0.39
Madagascar	896.48	661.43	1,132.29	892.37	708.88	1,077.00	0.01	0.08	-0.03
Malawi	680.9	619.63	744.33	649.6	593.61	705.8	-0.03	-0.03	-0.03
Malaysia	718.56	629.52	804.97	1,179.61	1,017.15	1,345.08	0.64	0.61	0.67
Maldives	553.63	348.93	748.02	742.81	586.63	898.12	0.34	0.67	0.20
Mali	455.23	301.17	612.64	380.88	257.39	501.93	-0.15	-0.14	-0.17
Malta	1,501.58	1,273.52	1,737.76	2,138.73	1,646.21	2,635.23	0.40	0.27	0.48
Marshall Islands	381.20	349.70	411.32	569.63	528.88	608.99	0.49	0.51	0.48
Mauritania	550.61	376.06	726.20	636.08	431.67	837.66	0.16	0.15	0.16
Mauritius	900.78	794.49	1,007.51	1,604.53	1,393.82	1,820.44	0.76	0.74	0.78
Mexico	672.75	630.58	715.35	1,083.74	996.41	1,171.96	0.60	0.57	0.63
Moldova	2,808.24	2,413.29	3,241.54	3,058.60	2,497.96	3,664.73	0.07	0.02	0.11
Mongolia	1,308.53	1,234.62	1,384.18	1,761.22	1,682.40	1,841.64	0.34	0.35	0.32
Montenegro	1,737.84	1,352.61	2,126.44	2,992.90	2,256.30	3,747.03	0.70	0.65	0.74
Morocco	789.71	675.41	904.47	1,491.54	1,241.51	1,747.40	0.87	0.82	0.91
Mozambique	879.74	753.4	1017.48	897.23	779.39	1,020.50	0.03	0.04	0.01
Myanmar	416.35	345.14	490.47	582.52	486.82	682.60	0.43	0.44	0.43
Namibia	575.68	436.91	720.48	605.34	475.3	742.33	0.05	0.09	0.03
Nepal	577.12	559.35	595.15	958.62	848.67	1,075.55	0.67	0.52	0.83
Netherlands	2,187.88	1,808.90	2,575.46	2,000.25	1,507.52	2,501.23	-0.09	-0.17	-0.04
New Zealand	1,664.01	1,179.64	2,163.23	1,768.21	1,162.22	2,397.38	0.05	-0.03	0.09
Nicaragua	559.79	559.67	559.9	873.70	866.66	880.94	0.57	0.55	0.59
Niger	451.89	302.53	606.29	606.22	375.51	833.19	0.36	0.25	0.40
Nigeria	581.06	394.83	763.57	589.69	395.04	777.1	0.02	0.00	0.02
North Korea	541.25	554.83	526.86	1,105.96	1,110.82	1,100.88	1.05	1.01	1.10
Northern Mariana Islands	382.6	291.68	464.32	470.55	329.34	612.76	0.25	0.14	0.35
Norway	2,218.69	1,758.25	2,689.53	1,742.88	1,353.18	2,128.04	-0.22	-0.23	-0.22
Oman	490.32	456.27	517.49	849.38	708.63	920.76	0.73	0.55	0.77
Pakistan	854.78	636.53	1,057.32	1,137.29	885.22	1,375.92	0.34	0.39	0.31
Palestine	545.60	498.03	592.00	806.59	728.80	882.21	0.46	0.45	0.47
Panama	792.91	750.1	834.78	1,285.17	1219.47	1,350.50	0.65	0.65	0.66
Papua New Guinea	305.63	273.73	336.07	474.52	421.12	525.83	0.55	0.54	0.57
Paraguay	1,102.87	770.04	1,428.13	1,469.47	988.65	1,935.81	0.32	0.27	0.34
Peru	557.60	487.35	627.88	736.54	654.05	819.15	0.32	0.34	0.31
Philippines	718.70	622.16	813.24	1,260.29	1,080.37	1,436.90	0.77	0.75	0.79
Poland	3,631.21	3,050.15	4,242.32	4,262.24	3,534.65	5,045.28	0.16	0.15	0.18
Portugal	2,177.38	2,106.38	2,253.73	2,485.48	2,358.04	2,621.37	0.12	0.11	0.14
Puerto Rico	1,335.48	1,179.58	1,501.24	1,862.06	1,763.20	1,968.86	0.38	0.48	0.30
Qatar	688.77	443.66	809.78	825.88	603.83	908.91	0.18	0.34	0.11
Romania	3,618.12	2,887.37	4,369.89	4,512.36	3,682.15	5,396.02	0.24	0.27	0.22
Russia	5,207.71	4,553.26	5,950.90	6,148.52	5,165.41	7,303.05	0.17	0.13	0.22
Rwanda	650.52	526.46	778.96	677.07	602.7	757.97	0.05	0.15	-0.02
Saint Lucia	946.77	837.97	1,058.83	1,319.01	1,175.86	1,467.63	0.41	0.41	0.41
Saint Vincent and the Grenadines	856.16	788.16	924.84	1,179.20	1,027.83	1,327.79	0.39	0.31	0.45
Samoa	570.08	552.67	586.13	837.25	811.70	861.25	0.46	0.46	0.46
Sao Tome and Principe	552.37	376.62	731.37	521.64	385.27	659.05	-0.05	0.02	-0.10
Saudi Arabia	672.30	601.55	727.94	1,168.57	900.84	1,374.26	0.71	0.47	0.86
Senegal	550.85	386.7	716.55	569.43	446.92	696.42	0.04	0.16	-0.02
Serbia	2,957.87	2,221.97	3,713.55	4,127.21	3,176.62	5,124.21	0.38	0.42	0.37
Seychelles	971.42	984.91	957.86	1,346.38	1,283.19	1,407.89	0.38	0.30	0.46
Sierra Leone	638.63	392.1	890.27	652.61	430.66	879.12	0.04	0.12	0.01
Singapore	704.34	615.25	791.12	979.95	865.19	1,098.80	0.36	0.38	0.36
Slovakia	3,111.26	2,272.46	3,989.68	3,496.82	2,566.15	4,478.82	0.11	0.12	0.11
Slovenia	2,989.08	2,522.86	3,483.44	3,868.63	3,033.99	4,718.26	0.28	0.19	0.33
Solomon Islands	388.85	319.38	454.62	488.83	416.92	558.52	0.26	0.31	0.23
Somalia	789.93	627.09	955.67	662.4	530.74	795.29	-0.16	-0.15	-0.17
South Africa	776.80	672.96	883.06	974.06	854.40	1,097.95	0.25	0.26	0.24
South Korea	396.46	384.73	408.13	918.71	873.83	964.14	1.25	1.21	1.28
South Sudan	821.17	642.63	1,001.39	816.02	654.85	976.73	0.00	0.02	-0.02
Spain	1,742.94	1,483.10	2,013.65	1,689.40	1,421.64	1,964.08	-0.04	-0.05	-0.04
Sri Lanka	769.03	658.74	877.20	1,584.94	1,433.74	1,747.47	1.09	1.20	1.03

Sudan	626.13	516.62	734.78	850.71	720.38	980.08	0.36	0.40	0.34
Suriname	799.56	703.47	892.59	1,152.62	1,056.37	1,248.52	0.44	0.50	0.40
Swaziland	594.8	484.59	718.06	638.04	557.70	720.07	0.07	0.15	0.00
Sweden	2,629.01	1,977.28	3,296.54	2,610.63	1,804.13	3,418.73	-0.02	-0.09	0.02
Switzerland	2,017.49	1,680.45	2,370.46	1,915.78	1,417.81	2,424.97	-0.06	-0.16	0.01
Syria	497.47	417.38	574.99	970.94	853.95	1,085.07	0.94	1.03	0.87
Taiwan	823.76	698.79	939.76	1,601.40	1497.31	1,705.83	0.99	1.17	0.87
Tajikistan	1,427.30	1,384.19	1,470.92	1,317.38	1,133.33	1,497.14	-0.08	-0.18	0.02
Tanzania	879.77	716.64	1,045.80	890.93	708.22	1075.88	0.03	0.00	0.05
Thailand	652.90	548.57	759.14	1,674.79	1,478.30	1,876.94	1.56	1.69	1.47
The Bahamas	806.71	747.77	866.47	1,462.85	1,350.57	1,579.95	0.79	0.78	0.80
The Gambia	531.31	305.68	753.73	581.81	367.61	800.23	0.10	0.20	0.07
Timor-Leste	324.65	267.92	379.95	652.34	565.19	736.80	1.03	1.13	0.96
Togo	510.3	353.23	671.44	564.34	397.2	735.44	0.12	0.13	0.11
Tonga	634.50	605.52	662.77	825.63	818.21	833.00	0.30	0.35	0.25
Trinidad and Tobago	977.50	889.66	1,066.00	1,603.38	1,464.38	1,746.07	0.62	0.63	0.62
Tunisia	1,061.84	888.64	1,232.93	2,131.60	1852.44	2,417.31	0.98	1.06	0.93
Turkey	1,278.63	1,075.50	1,486.75	1,552.34	1450.56	1,657.73	0.21	0.34	0.11
Turkmenistan	1,259.65	1,229.81	1,290.32	1,612.81	1,461.16	1,769.64	0.27	0.18	0.36
Uganda	602.78	489.59	717.45	474.14	395.26	553.1	-0.20	-0.18	-0.21
Ukraine	5,126.24	4,744.17	5,570.86	6,769.93	6,011.52	7,665.72	0.31	0.26	0.37
United Arab Emirates	303.18	234.09	339.06	462.65	292.34	524.73	0.51	0.23	0.53
United Kingdom	2,951.95	2,216.22	3,730.18	2,406.33	1,881.44	2,944.35	-0.19	-0.15	-0.22
United States	2,068.35	1,775.03	2,376.82	1,771.15	1,552.63	1,997.05	-0.15	-0.13	-0.17
Uruguay	867.85	665.76	1,082.39	925.99	781.23	1,081.06	0.06	0.17	-0.01
Uzbekistan	1,551.91	1,529.99	1,574.32	2,025.09	1,813.22	2,243.93	0.30	0.18	0.42
Vanuatu	504.30	404.90	598.30	617.56	541.30	691.93	0.18	0.30	0.11
Venezuela	671.96	681.54	662.52	1,173.45	1,169.84	1,177.08	0.73	0.70	0.75
Vietnam	773.31	796.94	748.95	1,018.05	1,036.97	998.73	0.35	0.32	0.38
Virgin Islands, U.S.	993.93	890.56	1,103.42	2,153.62	1,969.47	2,356.03	1.14	1.19	1.11
Yemen	542.68	504.36	581.69	788.38	698.33	876.59	0.45	0.38	0.50
Zambia	714.43	587.69	843.32	751.27	630.39	872.50	0.06	0.08	0.05
Zimbabwe	728.25	497.36	961.07	504.83	365.99	647.74	-0.31	-0.26	-0.32
**Ischemic stroke**									
Afghanistan	193.87	201.20	186.9	195.79	206.09	186.11	0.01	0.03	0.00
Albania	279.57	275.35	283.58	531.6	505.19	558.43	0.89	0.82	0.96
Algeria	188.62	199.06	178.43	272.27	288.83	255.93	0.42	0.43	0.41
American Samoa	268.99	234.73	301.43	254.54	248.94	260.11	-0.06	0.06	-0.14
Andorra	290.5	289.31	291.76	351.27	339.71	363.31	0.20	0.17	0.23
Angola	171.13	195.04	146.63	159.16	183.65	134.36	-0.06	-0.05	-0.08
Antigua and Barbuda	264.63	267.01	262.07	231.64	232.67	230.51	-0.13	-0.13	-0.12
Argentina	291.03	290.34	291.74	295.61	300.91	290.08	0.01	0.03	-0.01
Armenia	367.23	406.96	325.12	553.75	597.95	502.84	0.48	0.45	0.52
Australia	310.66	311.81	309.51	339.86	338.69	341.02	0.09	0.08	0.09
Austria	324.31	336.14	311.41	406.6	385.3	428.97	0.24	0.14	0.36
Azerbaijan	339.2	383.78	292.68	414.14	451.33	376.51	0.21	0.17	0.27
Bahrain	198.56	199.66	197.74	251.7	267.64	241.93	0.26	0.33	0.21
Bangladesh	243.91	227.44	259.64	271.28	251.87	290.31	0.12	0.11	0.13
Barbados	277.39	286.86	267.18	353.22	359.09	346.85	0.26	0.24	0.29
Belarus	767.37	919.15	595.45	942.23	1,098.17	761.34	0.22	0.19	0.26
Belgium	328.49	331.07	325.79	377.62	372.79	382.64	0.14	0.12	0.16
Belize	127.41	122.94	131.81	130.66	125.51	135.84	0.03	0.02	0.04
Benin	187.99	199.67	175.56	201.9	210.65	193.12	0.08	0.06	0.11
Bermuda	207.33	202.98	211.74	251.77	253.67	249.84	0.21	0.24	0.17
Bhutan	165.78	152.96	177.7	201.63	181.28	219.16	0.20	0.17	0.22
Bolivia	109.03	113.76	104.33	133.35	140.56	126.16	0.20	0.21	0.19
Bosnia and Herzegovina	373.98	410.15	337.78	663.57	687.46	639.47	0.75	0.66	0.87
Botswana	138.16	155.07	120.84	173.24	199.46	146.99	0.25	0.28	0.21
Brazil	159.97	152.25	167.8	164.63	157.61	171.87	0.02	0.03	0.02
Brunei	182.92	172.08	192.62	281.28	275.96	286.28	0.49	0.55	0.45
Bulgaria	735.40	713.14	758.30	883.71	901.29	865.26	0.19	0.26	0.13

Burkina Faso	183.62	195.12	171.59	143.07	158.87	127.07	-0.22	-0.18	-0.25
Burundi	235.13	255.7	213.84	203.03	212.75	193.09	-0.11	-0.15	-0.06
Cambodia	180.22	174.76	186.07	232.76	244.13	220.81	0.32	0.41	0.22
Cameroon	187.05	192.45	181.57	161.88	167.53	156.23	-0.12	-0.12	-0.12
Canada	289.68	295.6	283.66	373	366.86	379.22	0.27	0.23	0.31
Cape Verde	271.67	279.62	262.89	272.99	299.85	245.41	0.00	0.06	-0.07
Central African Republic	272.85	297.34	247.52	232.59	260.84	203.59	-0.15	-0.12	-0.18
Chad	161.94	168.77	155.02	141.37	147.31	135.44	-0.12	-0.13	-0.12
Chile	263.18	268.51	257.71	358.46	368.42	348.23	0.34	0.35	0.33
China	353.18	313.19	391.12	519.88	453.07	582.81	0.50	0.46	0.53
Colombia	76.67	80.97	72.31	101.34	106.23	96.3	0.31	0.30	0.32
Comoros	210.33	226	194.76	195.75	209.09	182.66	-0.07	-0.07	-0.06
Congo	286.88	327.92	245.63	237.59	283.68	191.57	-0.17	-0.13	-0.22
Costa Rica	63.12	64.78	61.49	95.24	98.27	92.22	0.49	0.50	0.48
Cote d'Ivoire	217.31	196.24	236.72	213.98	198.11	229.31	-0.01	0.02	-0.02
Croatia	648.56	699.62	594.30	810.64	830.37	789.47	0.24	0.18	0.32
Cuba	285.53	282.08	288.93	373.84	371.46	376.2	0.30	0.31	0.29
Cyprus	193.54	177.69	209.79	260.49	240.7	281.64	0.32	0.33	0.31
Czech Republic	568.76	603.60	531.84	601.26	622.96	578.86	0.05	0.02	0.08
Democratic Republic of the Congo	162.12	190.48	133.07	152.97	174.26	131.58	-0.05	-0.08	-0.01
Denmark	381.34	368.49	394.56	383.33	360.95	406.07	0.00	-0.02	0.02
Djibouti	180.58	189.32	171.94	266.36	281.64	251.22	0.47	0.48	0.45
Dominica	212.79	212.09	213.49	260.38	266.3	254.37	0.23	0.26	0.20
Dominican Republic	144.53	134.65	154.27	205.56	199.92	211.23	0.42	0.47	0.37
Ecuador	107.1	107.37	106.82	119.1	120.72	117.47	0.11	0.13	0.10
Egypt	187.64	195.67	179.65	245.96	254.47	237.62	0.31	0.30	0.33
El Salvador	56.83	57.14	56.51	79.06	82.38	75.32	0.40	0.44	0.35
Equatorial Guinea	255.29	294.94	217.35	231.1	244.2	218.63	-0.09	-0.17	0.01
Eritrea	141.79	158.41	124.95	176.5	200.54	152.56	0.25	0.27	0.23
Estonia	732.19	849.51	598.69	763.33	896.69	606.74	0.03	0.05	0.00
Ethiopia	196.31	200.91	191.68	239.39	258.97	219.73	0.23	0.30	0.16
Federated States of Micronesia	267.72	248.47	286.14	310.45	296.72	323.52	0.15	0.19	0.12
Fiji	265.4	250.83	279.51	350.1	346.01	354.06	0.31	0.37	0.26
Finland	376.13	397.78	353.16	441.55	428.39	455.11	0.16	0.07	0.27
France	316.83	318.78	314.77	392.71	391.6	393.89	0.23	0.22	0.24
Gabon	358.26	405	309.56	270.19	318.49	222.98	-0.25	-0.21	-0.28
Georgia	679.29	738.69	613.78	806.11	848.22	760.05	0.18	0.14	0.23
Germany	351.58	372.31	329.3	429.86	411	449.36	0.21	0.10	0.34
Ghana	179.6	203.94	156.04	192.07	212.06	171.88	0.08	0.05	0.12
Greece	351.71	340.37	363.34	424.14	411.51	437.4	0.20	0.20	0.19
Greenland	194.09	185.55	201.48	306.47	259.6	347.11	0.55	0.39	0.68
Grenada	277.96	297.15	258.27	249.96	263.46	236.54	-0.09	-0.10	-0.06
Guam	302.55	291.68	312.04	503.39	487.03	519.29	0.68	0.68	0.69
Guatemala	50.22	50.15	50.3	56.68	59.84	53.38	0.15	0.21	0.09
Guinea	186.48	199.19	173.49	187.84	195.39	180.32	0.01	-0.02	0.04
Guinea-Bissau	208.78	219.26	198.04	198.4	207.45	189.24	-0.04	-0.05	-0.03
Guyana	187.00	178.8	195.46	269.88	266.14	273.57	0.45	0.49	0.41
Haiti	151.07	150.21	151.96	167.66	172.95	162.25	0.14	0.17	0.11
Honduras	61.31	62.47	60.16	78.58	79.88	77.28	0.29	0.28	0.29
Hungary	731.03	749.77	710.77	742.77	797.62	682.31	0.01	0.06	-0.05
Iceland	222.64	213.94	231.27	268.43	253.16	284.03	0.19	0.17	0.21
India	197.6	180.87	213.19	226.33	202.46	248.5	0.15	0.12	0.17
Indonesia	275.6	258.22	292.84	339.36	320.48	357.97	0.25	0.25	0.24
Iran	178.26	173.88	182.49	269.83	270.74	268.93	0.49	0.53	0.45
Iraq	215.95	224.24	207.81	207.99	222.01	194.33	-0.04	-0.01	-0.07
Ireland	262.66	259.46	265.89	267.59	250.02	285.69	0.01	-0.05	0.06
Israel	214.23	201.62	226.89	228.57	218.1	239.21	0.06	0.08	0.05
Italy	336.23	323.3	349.94	382.97	370.47	396.27	0.13	0.14	0.12
Jamaica	227.4	231.18	223.52	243.3	240.89	245.73	0.06	0.03	0.09
Japan	539.58	457.89	624.21	664.92	549.39	786.59	0.21	0.18	0.23
Jordan	183.15	192.75	174.47	177.79	187.87	168.2	-0.04	-0.04	-0.05

Kazakhstan	448.70	523.91	368.78	522.23	579.32	461.06	0.16	0.11	0.25
Kenya	125.1	129.44	120.74	118.79	120.57	117.01	-0.05	-0.06	-0.03
Kiribati	296.51	273.89	319.59	322.15	294.65	350.36	0.09	0.08	0.10
Kuwait	152.01	128.77	172.34	184.78	180.68	187.99	0.20	0.38	0.08
Kyrgyzstan	407.46	458.32	354.42	384.11	410.34	357.31	-0.06	-0.11	0.01
Laos	205.86	200.05	211.67	228.07	226.16	229.99	0.12	0.14	0.11
Latvia	788.69	930.39	625.47	918.10	1,051.42	762.84	0.15	0.12	0.21
Lebanon	241.52	265.86	216.52	275.37	291.32	259.58	0.13	0.08	0.18
Lesotho	188.93	201.75	175.37	210.83	252.79	168.03	0.11	0.25	-0.05
Liberia	155.31	156.9	153.71	181.76	191.3	172.37	0.18	0.22	0.13
Libya	144.39	147.93	141.22	235.42	251.28	219.75	0.62	0.68	0.55
Lithuania	696.50	821.46	557.43	952.28	1,111.20	767.46	0.35	0.34	0.36
Luxembourg	331.01	338.92	322.77	336.41	328.1	344.77	0.02	-0.03	0.06
Macedonia	443.04	439.15	446.89	632.29	642.09	622.41	0.41	0.45	0.38
Madagascar	238.88	231.76	246.02	253.15	266.5	239.73	0.07	0.16	-0.01
Malawi	162.76	179.83	145.09	202.44	226.93	177.85	0.26	0.28	0.25
Malaysia	200.87	191.27	210.19	267.88	252.62	283.43	0.33	0.32	0.34
Maldives	143.89	106.65	179.27	164.67	152.73	176.54	0.14	0.42	-0.01
Mali	201.21	222.31	179.64	145.64	161.63	129.97	-0.27	-0.27	-0.26
Malta	237.17	227.51	247.17	355.4	336.31	374.65	0.47	0.46	0.48
Marshall Islands	215.83	191.01	239.58	264.24	244.43	283.37	0.22	0.28	0.18
Mauritania	208.19	220.74	195.56	193.46	210.35	176.81	-0.06	-0.04	-0.09
Mauritius	303.83	290.62	317.11	419.72	405.08	434.71	0.36	0.38	0.35
Mexico	58.19	60.22	56.13	60.83	58.91	62.76	0.04	-0.03	0.11
Moldova	569.41	637.57	494.63	604.57	667.78	536.24	0.05	0.03	0.07
Mongolia	314.18	324.64	303.47	477.35	496.74	457.57	0.51	0.52	0.50
Montenegro	389.14	421.72	356.27	656	691.82	619.33	0.67	0.63	0.72
Morocco	171.35	183.93	158.71	249.07	266.05	231.7	0.44	0.43	0.44
Mozambique	227.54	235.12	219.27	296.01	321.57	269.28	0.31	0.37	0.24
Myanmar	293.35	274.19	313.3	363.33	348.73	378.6	0.27	0.29	0.25
Namibia	200.33	216.62	183.33	221.89	258.65	183.17	0.11	0.19	0.00
Nepal	181.07	179.23	182.94	230.96	215.79	247.09	0.29	0.21	0.37
Netherlands	299.22	304.55	293.76	352.78	335.6	370.25	0.17	0.10	0.24
New Zealand	318.38	317.39	319.39	330.3	308.02	353.44	0.02	-0.04	0.09
Nicaragua	57.08	60.37	53.75	75.4	80.83	69.81	0.33	0.34	0.31
Niger	133.56	138.85	128.1	155.39	154.87	155.9	0.18	0.13	0.24
Nigeria	154.77	162.72	146.99	140.76	145.75	135.96	-0.09	-0.10	-0.07
North Korea	302.58	339.47	263.51	458.77	489.96	426.18	0.52	0.45	0.63
Northern Mariana Islands	212.82	183.52	239.15	202.32	168.59	236.29	-0.03	-0.07	0.01
Norway	376.16	370.81	381.63	336.68	331.46	341.83	-0.11	-0.11	-0.12
Oman	127.85	140.35	117.88	170.64	181.05	165.37	0.33	0.28	0.39
Pakistan	227.32	193.11	259.07	243.01	219.77	265.01	0.07	0.14	0.03
Palestine	137.66	147.95	127.61	163.46	174.28	152.94	0.17	0.17	0.18
Panama	83.59	80.8	86.32	111.47	110.8	112.14	0.36	0.39	0.33
Papua New Guinea	207.86	201.1	214.32	266.82	274.54	259.41	0.29	0.37	0.21
Paraguay	149.6	144.28	154.81	211.96	197.09	226.38	0.40	0.35	0.45
Peru	95.47	98.02	92.91	122.47	128.84	116.1	0.28	0.31	0.25
Philippines	168.44	160.48	176.23	241.72	237.66	245.7	0.45	0.49	0.41
Poland	457.74	488.46	425.43	579.82	595.83	562.59	0.25	0.21	0.31
Portugal	409.46	398.61	421.13	475.78	465.97	486.24	0.14	0.15	0.13
Puerto Rico	225.54	216.43	235.21	304.77	313.35	295.5	0.34	0.43	0.25
Qatar	127.88	112.98	135.24	144.01	144.7	143.75	0.11	0.26	0.05
Romania	650.10	674.15	625.37	834.69	871.95	795.04	0.27	0.28	0.26
Russia	748.74	908.81	566.96	905.27	1,039.88	747.18	0.20	0.14	0.31
Rwanda	183.17	194.46	171.48	190.24	216.75	161.41	0.04	0.12	-0.05
Saint Lucia	254.9	250.64	259.28	261.65	264.62	258.57	0.04	0.06	0.02
Saint Vincent and the Grenadines	208.44	223.37	193.37	243.01	246.33	239.75	0.17	0.11	0.25
Samoa	305.73	288.39	321.72	334.95	331.19	338.48	0.09	0.14	0.05
Sao Tome and Principe	193.06	199.55	186.45	165.13	180.33	149.82	-0.14	-0.10	-0.19
Saudi Arabia	144.53	148.16	141.67	176.86	166.19	185.06	0.20	0.10	0.28
Senegal	179.9	183.69	176.08	165.65	180.29	150.48	-0.07	-0.02	-0.14

Serbia	562.42	583.98	540.27	766.72	800.30	731.51	0.35	0.36	0.34
Seychelles	290.33	282.43	298.26	294.58	294.12	295.02	0.01	0.04	-0.02
Sierra Leone	194.98	183.88	206.31	203.85	209.56	198.03	0.07	0.16	-0.02
Singapore	334.16	332.44	335.84	482.54	474.23	491.14	0.41	0.40	0.43
Slovakia	472.35	492.54	451.20	566.25	589.68	541.52	0.18	0.18	0.19
Slovenia	464.91	503.19	424.33	585.83	602.23	569.13	0.24	0.18	0.32
Solomon Islands	265.91	237.28	293.01	276.62	258.23	294.44	0.04	0.09	0.01
Somalia	204.66	217.04	192.06	198.02	214.28	181.6	-0.03	-0.01	-0.05
South Africa	268.77	277.31	260.04	291.4	305.56	276.74	0.08	0.10	0.06
South Korea	381.36	403.54	359.28	636.86	630.08	643.73	0.62	0.52	0.73
South Sudan	206.35	217.37	195.23	240.8	259.98	221.68	0.17	0.20	0.14
Spain	311.95	315.71	308.04	377.09	382.68	371.35	0.20	0.20	0.19
Sri Lanka	191.25	174.22	207.94	354.84	348.1	362.08	0.88	1.02	0.77
Sudan	164.63	170.49	158.82	195.06	207.51	182.7	0.19	0.22	0.16
Suriname	200.82	198.64	202.93	263.33	265.39	261.28	0.31	0.33	0.29
Swaziland	145.46	152.32	137.8	196.66	229.43	163.2	0.35	0.50	0.18
Sweden	457.74	395.93	521.06	427.8	346.39	509.37	-0.08	-0.13	-0.04
Switzerland	303.44	301.99	304.95	317.1	309.97	324.39	0.04	0.02	0.05
Syria	154.48	151.19	157.66	226.16	231.21	221.24	0.45	0.52	0.39
Taiwan	343.43	301.7	382.16	478.85	482.52	475.17	0.42	0.62	0.28
Tajikistan	280.19	314.28	245.69	220.18	231.59	209.03	-0.22	-0.26	-0.15
Tanzania	168.33	180.13	156.31	185.74	197.56	173.76	0.12	0.11	0.13
Thailand	208.74	191.15	226.65	386.97	360.11	414.6	0.85	0.88	0.83
The Bahamas	176.33	179.11	173.52	259.9	269.99	249.36	0.46	0.49	0.42
The Gambia	163.37	155.57	171.06	153.67	148.58	158.85	-0.06	-0.04	-0.07
Timor-Leste	124.83	121.67	127.91	217.62	218.27	216.99	0.76	0.81	0.71
Togo	168.49	174.6	162.21	154.81	157.65	151.91	-0.07	-0.09	-0.05
Tonga	267.37	273.58	261.3	286.6	296.26	277	0.07	0.08	0.06
Trinidad and Tobago	213.76	210.7	216.83	282.79	278.04	287.67	0.31	0.31	0.31
Tunisia	219.98	213.83	226.05	325.09	331.5	318.54	0.46	0.53	0.39
Turkey	198.35	209.99	186.42	239.6	263.49	214.87	0.20	0.25	0.15
Turkmenistan	302.22	337.17	266.29	365.1	386.68	342.79	0.20	0.14	0.28
Uganda	168.63	181.57	155.53	147.76	160.38	135.13	-0.11	-0.11	-0.11
Ukraine	814.45	958.73	646.56	909.43	1,037.28	758.42	0.11	0.08	0.17
United Arab Emirates	163.52	153.01	168.98	183.81	158.17	193.15	0.11	0.02	0.13
United Kingdom	573.47	607.74	537.23	650.1	635.41	665.17	0.13	0.04	0.23
United States	309.02	318.25	299.32	318.97	312.6	325.56	0.02	-0.02	0.07
Uruguay	398.26	401.56	394.76	410.05	435.06	383.25	0.02	0.08	-0.04
Uzbekistan	262.09	291.22	232.32	296.73	314.72	278.16	0.12	0.07	0.19
Vanuatu	333.9	286.26	378.94	354.32	334.01	374.12	0.03	0.14	-0.05
Venezuela	80.46	81.79	79.15	109.09	111.62	106.55	0.34	0.35	0.33
Vietnam	315.87	301.07	331.12	332.32	324.86	339.95	0.08	0.10	0.06
Virgin Islands, U.S.	192.23	186.41	198.39	387	377.89	397.02	0.99	1.00	0.98
Yemen	145.88	159.45	132.06	182.95	197.14	169.06	0.25	0.23	0.28
Zambia	173.56	190.3	156.55	211.5	230.2	192.75	0.23	0.22	0.25
Zimbabwe	145.91	153.62	138.13	109.71	124.9	94.08	-0.25	-0.19	-0.32
**Other cardiovascular and circulatory diseases**									
Afghanistan	731.42	553.09	901.19	823.67	640.16	995.98	0.13	0.16	0.11
Albania	1580.12	1384.1	1766.81	3077.36	2794.24	3365.05	0.93	1.00	0.90
Algeria	1269.44	1352.33	1188.57	1619.45	1811.96	1429.59	0.26	0.32	0.18
American Samoa	677.9	354.93	983.62	711.51	369.41	1051.84	0.05	0.04	0.07
Andorra	1624.82	1714.77	1529.98	1767.58	1814.26	1718.95	0.08	0.05	0.11
Angola	705.97	728.96	682.42	691.39	703.46	679.15	-0.01	-0.03	0.00
Antigua and Barbuda	1435.5	1082.97	1812.56	1586.52	1242.26	1961.94	0.10	0.14	0.08
Argentina	969.59	1173.79	757.34	1072.29	1288.61	846.55	0.10	0.09	0.12
Armenia	113.57	134.73	91.14	202.44	234.07	166.01	0.76	0.71	0.79
Australia	1018.81	1091.47	945.75	1209.8	1251.79	1167.7	0.18	0.14	0.23
Austria	1377.44	1613.49	1120.23	1575.95	1710.64	1434.58	0.13	0.06	0.26
Azerbaijan	137.68	134.85	140.63	222.58	205.73	239.63	0.60	0.51	0.69
Bahrain	687.51	543.15	793.67	647.86	634.29	656.17	-0.06	0.16	-0.18
Bangladesh	60.87	45.5	75.56	103.58	77.23	129.43	0.72	0.71	0.74

Barbados	1327.27	1267.32	1391.87	1847.5	1745.89	1957.94	0.38	0.37	0.39
Belarus	111.87	144.11	75.36	141.38	181.61	94.73	0.25	0.25	0.24
Belgium	1852.2	1901.66	1800.44	1816.31	1829.58	1802.53	-0.03	-0.04	-0.01
Belize	1127.83	769.12	1480.52	1161.66	851.93	1473.24	0.03	0.11	0.00
Benin	2308.46	2833.62	1749.71	2231.24	2703.04	1757.6	-0.03	-0.04	0.02
Bermuda	1546.13	1299.16	1796.23	1808.44	1668.45	1951.1	0.16	0.28	0.08
Bhutan	75.93	56.12	94.34	122.18	89.23	150.54	0.59	0.57	0.58
Bolivia	726.29	486.11	964.82	940.49	601.03	1278.7	0.27	0.22	0.30
Bosnia and Herzegovina	1240.88	1012.66	1469.35	2234.73	1780.14	2693.53	0.78	0.74	0.81
Botswana	412.98	658.07	162.07	638.94	1020.57	256.95	0.54	0.54	0.58
Brazil	1953.66	1643.92	2268.29	2131.36	1831.64	2441.09	0.08	0.10	0.07
Brunei	275.3	223.68	321.51	371.98	324.85	416.33	0.31	0.40	0.26
Bulgaria	2568.08	2086.17	3063.56	3230.23	2875.04	3,602.99	0.25	0.37	0.17
Burkina Faso	2229.36	2770.73	1662.77	2033.41	2507.34	1553.32	-0.08	-0.09	-0.06
Burundi	993.91	1,233.82	745.59	909.36	1060.75	754.67	-0.05	-0.12	0.05
Cambodia	890.14	610.01	1190.27	973.77	795.02	1161.48	0.12	0.32	0.01
Cameroon	2583.48	2820.7	2343.27	2538.91	2659.15	2418.73	0.00	-0.05	0.05
Canada	1165.08	1386.15	940.21	1371.45	1522.17	1218.6	0.16	0.09	0.27
Cape Verde	4250.1	4624.99	3835.53	4775.14	5616.57	3911.17	0.12	0.21	0.02
Central African Republic	1012.68	1226.89	791.21	971.59	1158.84	779.34	-0.04	-0.05	-0.01
Chad	1932.02	2383.39	1474.08	1807.01	2123.04	1491.73	-0.06	-0.11	0.02
Chile	338.36	382.36	293.18	610.06	666.69	551.91	0.77	0.72	0.85
China	1161.5	881.94	1426.7	1655.84	1492.03	1810.14	0.45	0.71	0.30
Colombia	1286.24	1317.4	1254.69	2221.15	2383.97	2053.3	0.71	0.80	0.62
Comoros	1173.11	1492.65	855.61	1154.5	1444.53	869.7	-0.01	-0.03	0.02
Congo	1018.74	1243.37	792.94	1018.77	1223.29	814.56	0.00	-0.01	0.03
Costa Rica	1060.91	1035.34	1086.07	1651.72	1637.84	1665.59	0.54	0.56	0.51
Cote d'Ivoire	1959.56	2276.11	1667.89	2258.99	2487.21	2038.54	0.16	0.10	0.24
Croatia	2701.25	2529.22	2884.05	3782.54	3511.69	4073.23	0.39	0.38	0.40
Cuba	668.08	521.06	812.88	1034.32	844.52	1222.32	0.53	0.61	0.49
Cyprus	1693.67	1801.06	1583.55	1672.23	1778.91	1558.19	-0.03	-0.03	-0.04
Czech Republic	2093.13	2218.66	1960.07	3116.75	3222.62	3007.48	0.48	0.44	0.52
Democratic Republic of the Congo	875.57	1085.5	660.45	794.35	934.34	653.64	-0.09	-0.14	-0.01
Denmark	1580.88	1524.31	1639.1	1615.31	1475.6	1757.25	0.02	-0.03	0.06
Djibouti	1157.92	1427.66	891.35	1589.95	2029.57	1154.23	0.37	0.42	0.29
Dominica	884.41	745.81	1024.04	1119.79	988.82	1252.84	0.27	0.33	0.24
Dominican Republic	1351.81	871.3	1825.93	1430.91	1039.19	1825.22	0.05	0.18	0.00
Ecuador	606.29	403.56	806.78	756.23	540.61	971.9	0.25	0.34	0.21
Egypt	1747.55	1568.59	1925.28	1827.74	1656.93	1995.03	0.05	0.05	0.04
El Salvador	1215.46	1146.52	1287.66	1550.1	1569.53	1528.14	0.28	0.37	0.20
Equatorial Guinea	1175.57	1471.47	892.46	1138.48	1311.22	974.19	-0.03	-0.11	0.09
Eritrea	692.15	837	545.48	879.66	1107.96	652.19	0.28	0.33	0.20
Estonia	397.78	401.78	393.22	549.46	553.54	544.67	0.37	0.37	0.37
Ethiopia	728.98	961.82	494.67	832.69	1124.59	539.81	0.15	0.18	0.10
Federated States of Micronesia	943.89	561.25	1310.17	950.92	671.26	1217.28	0.00	0.19	-0.08
Fiji	768.26	615.58	916.21	990.82	942.26	1037.76	0.28	0.52	0.12
Finland	1078.02	1213.66	934.13	1431.9	1518.66	1342.47	0.31	0.24	0.41
France	2632.56	2501.44	2770.82	2805.71	2559.69	3067.66	0.06	0.02	0.10
Gabon	1662.89	2168.95	1135.72	1445.86	1814.01	1086	-0.13	-0.16	-0.04
Georgia	501	419.81	590.54	678.06	564.03	802.8	0.34	0.34	0.35
Germany	1666.06	1864.88	1452.46	1909.1	1961.91	1854.49	0.13	0.05	0.26
Ghana	2046.77	2048.54	2045.05	2466.69	2541.1	2391.52	0.22	0.25	0.19
Greece	918.96	874.15	964.91	1020.44	1022.77	1018	0.10	0.16	0.04
Greenland	1540.61	1265.63	1778.59	2258.4	1676.51	2762.92	0.44	0.31	0.51
Grenada	978.81	706.58	1258.18	1005.09	750.44	1258.16	0.04	0.07	0.02
Guam	610.81	578.33	639.18	788.8	794.95	782.82	0.30	0.38	0.24
Guatemala	652.91	502.46	805.52	767.39	623.29	917.79	0.20	0.26	0.17
Guinea	3021.01	3059.12	2982.04	3038.65	2981.51	3095.53	0.01	-0.02	0.04
Guinea-Bissau	2382.07	2813.12	1940.61	2405.7	2788.63	2017.98	0.02	0.00	0.05
Guyana	561.04	388.98	738.68	705.3	538.14	870.86	0.26	0.38	0.19
Haiti	928.82	583.29	1284.52	920.42	623.47	1223.86	0.02	0.09	-0.01

Honduras	725.22	601.09	848.48	750.88	639.64	862.13	0.04	0.06	0.02
Hungary	1117.62	1110.33	1125.51	1620.03	1644.16	1593.42	0.44	0.47	0.41
Iceland	1194.97	1160.27	1229.37	1243.3	1190.48	1297.26	0.03	0.02	0.04
India	19.67	17.94	21.29	32.2	30.19	34.08	0.64	0.68	0.60
Indonesia	671.24	558.76	782.76	862.45	764.65	958.84	0.30	0.38	0.25
Iran	5,985.76	3,723.63	8,165.73	4837.16	3584.34	6071.26	-0.21	-0.06	-0.27
Iraq	1400.41	1185.26	1611.65	1328	1183.71	1468.72	-0.05	0.00	-0.09
Ireland	762.26	777.5	746.88	793.33	787.2	799.64	0.03	0.00	0.06
Israel	563.43	512.1	614.95	617.17	591.65	643.12	0.09	0.15	0.04
Italy	995.57	1031.75	957.18	1109.9	1096.1	1124.58	0.10	0.06	0.16
Jamaica	1350.61	1028.31	1681.15	1591.07	1253.25	1931.57	0.17	0.21	0.14
Japan	1268.16	1146.83	1393.86	1754.01	1616.06	1899.3	0.35	0.39	0.33
Jordan	1246.53	1311.24	1187.97	1250.02	1435.22	1073.76	-0.01	0.08	-0.11
Kazakhstan	503.13	343.87	672.37	646.27	429.33	878.73	0.28	0.25	0.31
Kenya	408.68	565.16	251.1	507.55	750.77	264.38	0.25	0.33	0.06
Kiribati	869.01	549.33	1195.37	953.41	654.53	1259.97	0.10	0.19	0.06
Kuwait	733.05	751.78	716.66	760.49	895.64	655.01	0.02	0.17	-0.10
Kyrgyzstan	222.99	220.66	225.42	240.59	229.48	251.95	0.08	0.04	0.12
Laos	861.65	600.48	1123.18	846.14	628.09	1066.37	0.00	0.06	-0.03
Latvia	248.87	294.62	196.17	340.71	398.93	272.91	0.35	0.34	0.37
Lebanon	1423.97	1454.85	1392.24	1567.15	1718.89	1416.95	0.09	0.17	0.01
Lesotho	667.89	1036.48	277.57	710.94	1147.76	265.26	0.06	0.11	-0.05
Liberia	2089.1	2495.28	1680.64	2212.74	2652.66	1779.93	0.06	0.07	0.07
Libya	1776.08	1452.46	2065.9	1688.63	1558.5	1817.23	-0.06	0.06	-0.13
Lithuania	498.85	537.75	455.56	780.04	842.66	707.21	0.54	0.55	0.53
Luxembourg	3169.19	3037.71	3306.04	2664.7	2497.81	2832.25	-0.16	-0.18	-0.15
Macedonia	1935.54	1482.35	2383.42	2759.45	2253.49	3269.86	0.41	0.50	0.36
Madagascar	1215.06	1335.47	1,094.26	1195.06	1391.4	997.5	0.00	0.05	-0.07
Malawi	1037.53	1269.12	797.79	1155.83	1450.96	859.62	0.13	0.16	0.10
Malaysia	2204.43	1991.36	2411.21	2520.03	2621.96	2416.2	0.14	0.31	0.00
Maldives	2569.85	1530.47	3556.91	2758.37	1989.16	3523.33	0.07	0.29	-0.01
Mali	2179.77	2651.47	1697.82	1885.19	2235.87	1541.46	-0.12	-0.15	-0.07
Malta	909.18	808.92	1013.02	1209.97	1090.64	1330.26	0.30	0.33	0.28
Marshall Islands	801.49	602.58	991.71	870.02	719.59	1015.28	0.08	0.19	0.02
Mauritania	2064.19	2488.34	1637.49	2387.74	2875.54	1906.68	0.16	0.16	0.17
Mauritius	485.14	475.27	495.06	741.59	795.57	686.29	0.51	0.66	0.36
Mexico	836.23	647.66	1026.77	1073.77	883.92	1265.55	0.28	0.36	0.22
Moldova	223.95	179.07	273.19	300.64	237.91	368.45	0.32	0.31	0.33
Mongolia	349.15	196.25	505.68	400.1	236.64	566.87	0.14	0.20	0.11
Montenegro	2066.92	1786.48	2349.81	2484.38	2184.83	2791.05	0.19	0.21	0.17
Morocco	3222.66	2243.9	4,205.23	3641.54	2890.02	4410.61	0.12	0.28	0.04
Mozambique	2940.69	2813.52	3079.33	2953.75	2903.07	3006.77	0.01	0.04	-0.02
Myanmar	938.82	700.4	1186.94	1153.42	1034.07	1278.24	0.26	0.50	0.11
Namibia	584.96	915.86	239.67	638.53	1005.65	251.79	0.09	0.10	0.05
Nepal	46.85	35.97	57.9	71.83	50.22	94.8	0.55	0.40	0.66
Netherlands	1519.4	1772.85	1260.2	1915.43	2005.31	1824.04	0.25	0.13	0.43
New Zealand	647.44	668.96	625.26	780.15	751.57	809.81	0.19	0.11	0.27
Nicaragua	777.5	762.78	792.44	1104.33	1121.72	1086.45	0.43	0.48	0.39
Niger	1750.61	2109.55	1379.57	1822.38	2113.17	1536.31	0.05	0.01	0.13
Nigeria	1999.22	2536.02	1473.17	2094.28	2553.6	1652.03	0.05	0.01	0.12
North Korea	1126.58	1186.44	1063.2	1400.55	1639.77	1150.56	0.25	0.39	0.09
Northern Mariana Islands	876.82	636.15	1093.13	885.89	632.21	1141.38	0.03	0.00	0.07
Norway	1578.68	1529.99	1628.46	1366.54	1276.19	1455.85	-0.14	-0.17	-0.12
Oman	14,355.49	9,232.59	18,442.27	11,031.67	8,207.19	12,463.87	-0.23	-0.12	-0.33
Pakistan	57.95	36.46	77.89	73.11	50.93	94.11	0.27	0.40	0.21
Palestine	726.34	743.75	709.35	736.29	800.47	673.9	0.00	0.07	-0.06
Panama	774.8	710.2	837.99	1257.47	1197.88	1316.73	0.65	0.71	0.61
Papua New Guinea	1050.62	650.16	1432.85	1095.17	729.57	1446.44	0.04	0.12	0.01
Paraguay	1473.39	1054.68	1882.58	1537.94	1186.31	1878.99	0.03	0.12	-0.01
Peru	841.91	512.41	1171.5	1082.73	735.89	1430.06	0.29	0.44	0.22
Philippines	129.83	117.86	141.56	151.75	150.18	153.28	0.18	0.28	0.09

Poland	1177.99	1033.3	1330.16	1860.37	1658.98	2077.11	0.56	0.59	0.55
Portugal	1374.49	1288.28	1467.21	1441.17	1415.87	1468.15	0.03	0.09	-0.02
Puerto Rico	1315.11	1211.15	1425.64	1598.75	1599.22	1598.24	0.21	0.31	0.11
Qatar	1224.2	956.6	1356.31	1151.5	987.96	1212.65	-0.07	0.01	-0.12
Romania	863.49	597.96	1136.65	1380.22	1001.01	1783.83	0.59	0.66	0.56
Russia	368.13	416.75	312.92	474.77	518.75	423.13	0.28	0.24	0.34
Rwanda	921.73	1101.6	735.5	1097.94	1378.18	793.11	0.20	0.26	0.09
Saint Lucia	1057.63	820.76	1301.58	1350.51	1113.1	1597.02	0.29	0.37	0.25
Saint Vincent and the Grenadines	1206.14	941.62	1473.3	1456.48	1167.93	1739.73	0.21	0.24	0.19
Samoa	943.26	657.17	1206.97	1019.72	763.4	1260.41	0.07	0.16	0.04
Sao Tome and Principe	3156.34	3,904.67	2394.17	2683.14	3244.65	2117.37	-0.15	-0.17	-0.11
Saudi Arabia	1329.47	1117.36	1496.31	1553.69	1451.21	1632.43	0.15	0.28	0.07
Senegal	1116.38	1854.39	371.4	1250.57	2025.04	447.73	0.13	0.10	0.22
Serbia	1522.1	1350.21	1698.61	2157.78	1944.35	2381.63	0.40	0.43	0.39
Seychelles	1500.44	1144.26	1858.15	1577.17	1294.27	1852.57	0.05	0.13	-0.01
Sierra Leone	2353.46	2740.07	1958.84	2234.85	2525.58	1938.16	-0.03	-0.06	0.02
Singapore	275.15	349.44	202.79	470.26	598.19	337.76	0.67	0.68	0.63
Slovakia	1978.2	1858.95	2103.09	2791.61	2701.06	2887.17	0.39	0.44	0.36
Slovenia	1560.81	1618.22	1499.95	2590.8	2523.85	2658.96	0.64	0.54	0.74
Solomon Islands	853.32	441.48	1243.28	872.77	487.33	1246.3	0.02	0.10	0.00
Somalia	1068.78	1334.43	798.38	973.99	1179.98	766.1	-0.09	-0.11	-0.04
South Africa	1066.26	1362.5	763.12	1496.81	2009.69	965.81	0.40	0.47	0.26
South Korea	801.48	837.69	765.43	1038.22	1126.74	948.64	0.26	0.31	0.20
South Sudan	1130.23	1343.03	915.43	1235.89	1495.54	977.01	0.10	0.12	0.07
Spain	1997.91	1816.29	2187.14	1969.08	1818.11	2123.95	-0.02	-0.01	-0.04
Sri Lanka	2183.35	1521.45	2832.5	2392.06	1926.69	2892.31	0.11	0.28	0.04
Sudan	2124.57	1385.36	2857.99	2101.45	1460.66	2737.54	-0.01	0.06	-0.04
Suriname	874	649.25	1091.57	1049.88	842.87	1256.12	0.20	0.29	0.15
Swaziland	437.64	656.98	192.34	589.04	926.68	244.28	0.34	0.41	0.26
Sweden	1602.25	1462.32	1745.57	1509.3	1395.67	1623.15	-0.07	-0.05	-0.09
Switzerland	2770.92	2712.43	2832.17	2792.73	2623.48	2965.78	0.00	-0.04	0.04
Syria	886.21	635.1	1129.27	766.05	622.23	906.37	-0.14	-0.03	-0.20
Taiwan	1255.63	1366.19	1152.99	2162.91	2747.35	1576.51	0.76	1.03	0.41
Tajikistan	24.31	18.88	29.81	29.28	20.92	37.45	0.20	0.11	0.25
Tanzania	1101.84	1334.19	865.35	1188.53	1402.16	972.28	0.10	0.07	0.15
Thailand	2054.65	1742.01	2373.04	2518.8	2523.57	2513.9	0.22	0.44	0.06
The Bahamas	780.08	698.27	863.02	1038.97	991.88	1088.08	0.32	0.40	0.25
The Gambia	2065.83	2106.39	2025.84	2160.42	2219.84	2099.83	0.05	0.05	0.04
Timor-Leste	738.8	517.72	954.31	841	650.71	1025.4	0.15	0.27	0.08
Togo	2052.79	2490.64	1603.59	2181.72	2615.23	1737.97	0.07	0.06	0.10
Tonga	353.71	201.96	501.77	392.4	248.93	535.1	0.11	0.23	0.06
Trinidad and Tobago	488.57	448.13	529.31	690.03	677.09	703.31	0.40	0.49	0.32
Tunisia	2096.49	2016	2176	2433.65	2630.63	2232.04	0.15	0.29	0.01
Turkey	2438.61	2264.52	2616.98	2967.89	3159.72	2769.27	0.21	0.38	0.05
Turkmenistan	70.84	71.24	70.42	86.7	85.43	88.02	0.21	0.19	0.24
Uganda	662.21	896.15	425.21	671.17	889.09	453.04	0.03	0.00	0.09
Ukraine	103.67	123.11	81.06	128.5	151.61	101.22	0.23	0.22	0.24
United Arab Emirates	309.27	288.97	319.81	367.36	362.68	369.06	0.17	0.24	0.14
United Kingdom	1109.18	1149.58	1066.45	1164.34	1148.6	1180.48	0.05	0.00	0.10
United States	1849.28	1928.89	1765.56	1984.18	1982.38	1986.04	0.07	0.03	0.11
Uruguay	1386.54	1363.11	1411.41	1508.36	1526.13	1489.33	0.08	0.11	0.05
Uzbekistan	48.96	35.13	63.1	67.25	46.98	88.19	0.36	0.33	0.39
Vanuatu	817.65	377.48	1233.87	872.36	473.29	1261.54	0.03	0.22	-0.02
Venezuela	400.52	459.25	342.7	864.26	942.13	785.72	1.13	1.03	1.26
Vietnam	860.22	879.48	840.36	1249.47	1381.01	1115.1	0.49	0.59	0.37
Virgin Islands, U.S.	1226.54	1038.55	1425.67	2105.72	1778.91	2464.93	0.70	0.69	0.71
Yemen	1422.45	1013.28	1839.13	1259.57	948.33	1564.41	-0.12	-0.07	-0.15
Zambia	993.68	1208.54	775.19	1047.57	1289.43	805.04	0.07	0.08	0.05
Zimbabwe	507.61	729.02	284.34	599.9	855.27	337.04	0.18	0.17	0.19
**Peripheral artery disease**									
Afghanistan	653.96	645.14	662.36	756.16	761.2	751.42	0.16	0.18	0.14

Albania	1591.44	1653.79	1532.06	3412.93	3484.08	3340.63	1.13	1.09	1.17
Algeria	1011.8	1028.41	995.6	1897.16	1869.53	1924.41	0.85	0.79	0.90
American Samoa	787.15	787.74	786.59	861	934.88	787.49	0.09	0.18	0.00
Andorra	3256.32	3704.63	2783.63	4190.56	4603.82	3759.97	0.28	0.24	0.33
Angola	775.66	818.05	732.21	765.7	801.21	729.73	0.00	-0.01	0.01
Antigua and Barbuda	2211.16	2360.63	2051.28	2389.93	2445.49	2329.34	0.08	0.03	0.13
Argentina	1667.25	1757.06	1573.89	1944.88	2152.12	1728.62	0.16	0.22	0.10
Armenia	1863.27	2060.54	1654.19	3601.6	3914.45	3241.26	0.90	0.87	0.93
Australia	2414.7	2346.29	2483.48	2957.49	2790.19	3125.22	0.22	0.18	0.25
Austria	3436.01	4034.54	2783.87	4226.71	4622.53	3811.25	0.22	0.14	0.35
Azerbaijan	1409.23	1612.68	1196.94	2189.67	2346.78	2030.71	0.54	0.44	0.68
Bahrain	934.06	900.66	958.62	1299.65	1356.46	1264.86	0.38	0.50	0.31
Bangladesh	846.91	814.35	878.02	1272.73	1257.57	1287.6	0.52	0.55	0.49
Barbados	2801.73	3118.93	2459.92	4221.58	4392.2	4036.15	0.49	0.40	0.62
Belarus	3076.33	3711.6	2356.8	4431.54	5336.07	3382.27	0.43	0.43	0.42
Belgium	3347.64	3771.9	2903.71	4023.36	4406.49	3625.13	0.19	0.16	0.23
Belize	1177.56	1159.56	1195.25	1280.94	1205.16	1357.18	0.09	0.04	0.15
Benin	1129.77	1207.41	1047.17	1100.91	1129.25	1072.45	-0.02	-0.06	0.04
Bermuda	2129.07	2111.03	2147.33	3068.38	3105.75	3030.3	0.43	0.46	0.40
Bhutan	930.29	884.88	972.49	1455.03	1374.02	1524.76	0.55	0.53	0.55
Bolivia	1655.28	1673.97	1636.72	2418.91	2423.88	2413.96	0.44	0.42	0.45
Bosnia and Herzegovina	2256.86	2551.76	1961.64	4962.2	5382.14	4538.38	1.17	1.09	1.28
Botswana	1211.7	1345.91	1074.31	1700.78	1753.57	1647.95	0.40	0.30	0.52
Brazil	1942.58	2068.57	1814.6	3644.06	3885.22	3394.84	0.86	0.86	0.85
Brunei	551.54	566.05	538.56	1024.74	1135.88	920.16	0.80	0.94	0.66
Bulgaria	3683.06	3861.77	3499.33	5749.08	6401	5064.89	0.55	0.65	0.44
Burkina Faso	1077.14	1138.86	1012.54	909.97	989.09	829.83	-0.15	-0.13	-0.17
Burundi	1008.59	1052.98	962.63	941.52	903.98	979.87	-0.04	-0.12	0.06
Cambodia	770.67	809.22	729.37	1149.32	1276.82	1015.42	0.52	0.60	0.43
Cameroon	1149.55	1151.96	1147.11	1087.89	1076.39	1099.38	-0.04	-0.05	-0.02
Canada	1783.83	1792.19	1775.33	2238.26	2170.27	2307.2	0.24	0.20	0.28
Cape Verde	1526.19	1579.47	1467.26	1861.36	2018.51	1700	0.21	0.27	0.15
Central African Republic	1031.76	1073.03	989.09	1000	1055.75	942.76	-0.03	-0.01	-0.04
Chad	1045.96	1051.34	1040.5	875.4	870.35	880.44	-0.16	-0.17	-0.15
Chile	1366.27	1435.03	1295.65	2223.44	2391.86	2050.52	0.60	0.64	0.55
China	1074.55	1129.88	1022.05	2125.8	2202.08	2053.95	1.02	0.97	1.06
Colombia	1430.41	1523.37	1336.24	2620.91	2714.72	2524.2	0.82	0.77	0.87
Comoros	1090.92	1134.08	1048.05	1103.93	1098.58	1109.17	0.01	-0.03	0.06
Congo	1219.11	1278.17	1159.75	1206.07	1254.2	1158.01	-0.01	-0.02	0.00
Costa Rica	1618.64	1670.52	1567.59	2973.27	3010.25	2936.33	0.81	0.78	0.85
Cote d'Ivoire	1098.53	975.57	1211.83	1177.75	1006.53	1343.13	0.08	0.04	0.12
Croatia	3411.36	3917.58	2873.41	5492.44	6151.8	4784.79	0.60	0.56	0.65
Cuba	2761.88	2753.07	2770.55	4582.37	4623.2	4541.93	0.64	0.67	0.62
Cyprus	2410.21	2479.84	2338.8	3170.53	3283.72	3049.52	0.29	0.30	0.27
Czech Republic	3564.91	4078.04	3020.98	5020.39	5562.86	4460.46	0.40	0.35	0.46
Democratic Republic of the Congo	883.88	962.82	803	828	866	789.8	-0.06	-0.10	-0.01
Denmark	3731.94	4155.55	3295.97	4035.8	4284.93	3782.69	0.08	0.03	0.14
Djibouti	1043.97	1066.28	1021.92	1613.94	1581.82	1645.78	0.54	0.48	0.60
Dominica	2001.22	1987.57	2014.97	2896.17	2931.9	2859.87	0.46	0.48	0.43
Dominican Republic	1320.28	1222.64	1416.63	2291.88	2259.16	2324.82	0.73	0.84	0.64
Ecuador	1466.33	1480.45	1452.38	2365.01	2383.19	2346.83	0.62	0.61	0.62
Egypt	1335.04	1412.47	1258.14	1572.05	1653.42	1492.38	0.18	0.17	0.19
El Salvador	1382.32	1422.76	1339.98	2514.8	2585.87	2434.46	0.83	0.82	0.84
Equatorial Guinea	1246.32	1326.92	1169.2	1242.25	1173.49	1307.65	0.00	-0.11	0.12
Eritrea	660.94	717.74	603.42	873.13	929.64	816.82	0.33	0.30	0.36
Estonia	3319.3	4028.47	2512.29	5159.5	6193.64	3945.26	0.54	0.52	0.55
Ethiopia	970.95	922.17	1020.04	1144.1	1129.09	1159.16	0.19	0.23	0.15
Federated States of Micronesia	812.98	838.56	788.49	1107.98	1176.88	1042.36	0.36	0.40	0.31
Fiji	745.04	745.2	744.9	1419.96	1480.97	1360.97	0.89	0.98	0.81
Finland	3105.13	3634.61	2543.38	4125.42	4523.08	3715.54	0.31	0.24	0.44
France	3235.52	3663.36	2784.39	4042.19	4479.28	3576.8	0.24	0.22	0.28

Gabon	1699.98	1826.34	1568.35	1513.13	1599.75	1428.45	-0.11	-0.12	-0.09
Georgia	2794.48	3219.57	2325.64	4209.51	4757.89	3609.69	0.50	0.47	0.54
Germany	3609.09	4120.98	3059.14	4654.45	4844.65	4457.79	0.28	0.17	0.44
Ghana	954.78	941.95	967.21	1167.86	1174.9	1160.74	0.24	0.26	0.22
Greece	3191.16	3351.9	3026.3	4424.6	4743.11	4090.31	0.37	0.41	0.34
Greenland	702.08	659.88	738.6	1297.45	1043.08	1517.99	0.81	0.57	1.00
Grenada	2074.77	2283.23	1860.84	2233.37	2429.29	2038.67	0.09	0.07	0.12
Guam	940.17	991.34	895.48	2014.31	2117.05	1914.46	1.16	1.15	1.17
Guatemala	1107.4	1097.25	1117.71	1513.16	1555.33	1469.15	0.40	0.44	0.35
Guinea	1194.94	1190.98	1198.97	1134.96	1106.75	1163.04	-0.05	-0.07	-0.03
Guinea-Bissau	1254.59	1263.19	1245.78	1194.98	1171.65	1218.61	-0.04	-0.07	-0.01
Guyana	1219.1	1192.72	1246.32	1933.89	1936.14	1931.67	0.59	0.62	0.56
Haiti	1272.73	1261.49	1284.3	1494.05	1518.72	1468.85	0.21	0.22	0.19
Honduras	1174.62	1194.33	1155.04	1774.25	1770.84	1777.66	0.51	0.48	0.55
Hungary	4150.95	4669.03	3590.52	5764.46	6626.17	4814.54	0.38	0.41	0.33
Iceland	2322.39	2474.46	2171.66	2945.28	3060.92	2827.17	0.25	0.23	0.28
India	1045.31	1045.28	1045.33	1520.99	1565.65	1479.49	0.46	0.50	0.42
Indonesia	1061.33	1088.69	1034.19	1554.8	1605.87	1504.48	0.49	0.49	0.48
Iran	965.35	891.9	1036.13	1590.52	1479.92	1699.46	0.62	0.63	0.62
Iraq	1074.65	1069.92	1079.31	1014.97	1065.66	965.54	-0.06	-0.01	-0.11
Ireland	2637.89	2835.76	2438.27	2756.13	2865.3	2643.72	0.03	0.00	0.07
Israel	2090.15	2179.61	2000.36	2458.88	2661.47	2252.85	0.17	0.22	0.12
Italy	3527.26	3886.53	3146.06	4482.48	4924.28	4012.76	0.26	0.26	0.26
Jamaica	2031.67	2069.85	1992.52	2690.14	2666.41	2714.06	0.31	0.28	0.35
Japan	2296.3	2538.37	2045.51	4037.62	4549.11	3498.94	0.72	0.77	0.66
Jordan	956.89	928.56	982.53	1097.07	1086.79	1106.86	0.13	0.16	0.11
Kazakhstan	1877.85	2287.89	1442.1	2444.05	2813.69	2047.99	0.30	0.23	0.42
Kenya	897.41	877.66	917.3	963.51	987.1	939.93	0.08	0.13	0.03
Kiribati	851.79	928.61	773.38	1030.38	1139.53	918.43	0.21	0.23	0.19
Kuwait	677.04	509.82	823.3	890.81	804.45	958.21	0.30	0.56	0.15
Kyrgyzstan	1636.6	1933.71	1326.72	1676.59	1856.39	1492.95	0.02	-0.04	0.12
Laos	973.5	1027.31	919.61	1149.89	1215.14	1083.99	0.20	0.20	0.20
Latvia	3357.89	4051.42	2559.05	4993.74	6015.09	3804.25	0.47	0.47	0.47
Lebanon	1746.98	1662.23	1834.07	2400.22	2280.08	2519.14	0.36	0.36	0.36
Lesotho	1812.86	1954.26	1663.11	1771.38	2030.46	1507.06	-0.03	0.04	-0.10
Liberia	1075.45	1037.79	1113.33	1152.84	1140.41	1165.07	0.08	0.10	0.05
Libya	996.79	963.14	1026.93	1577.3	1587.64	1567.08	0.57	0.63	0.52
Lithuania	3208.54	3737.14	2620.24	5379.82	6416.53	4174.14	0.66	0.70	0.57
Luxembourg	3152.5	3588.94	2698.24	3300.15	3611.14	2987.91	0.05	0.01	0.10
Macedonia	2161.63	2204.59	2119.18	3724.69	3976.7	3470.47	0.70	0.78	0.62
Madagascar	1109.32	1014.91	1204.04	1108.19	1087.19	1129.32	0.01	0.08	-0.05
Malawi	1009.57	1042.29	975.69	1131.46	1168.44	1094.34	0.14	0.14	0.15
Malaysia	1001.02	989.36	1012.34	1648.99	1599.14	1699.78	0.64	0.61	0.67
Maldives	924.09	722.73	1115.31	1447.44	1363.47	1530.95	0.56	0.88	0.38
Mali	1209.17	1200.1	1218.43	933.7	955.37	912.47	-0.22	-0.20	-0.24
Malta	2403.46	2551.32	2250.34	3913.66	4148.27	3677.15	0.60	0.60	0.59
Marshall Islands	630.58	658.62	603.76	914.87	971.93	859.76	0.45	0.47	0.42
Mauritania	1095.87	1126.19	1065.38	1235.33	1259.73	1211.26	0.13	0.12	0.15
Mauritius	1323.92	1467.35	1179.91	2759.45	2956.25	2557.78	1.06	1.00	1.13
Mexico	1453.98	1527.36	1379.83	2342.22	2398.8	2285.05	0.60	0.56	0.64
Moldova	2283.26	2578.42	1959.45	3264.1	3726.58	2764.09	0.41	0.43	0.39
Mongolia	1241.36	1269.73	1212.33	1473.53	1578.11	1366.83	0.18	0.23	0.12
Montenegro	2767.37	3133.01	2398.54	4446.02	4776.18	4108	0.59	0.51	0.69
Morocco	1127.93	1147.49	1108.3	1961.55	1955.21	1968.04	0.72	0.69	0.76
Mozambique	1208.99	1262.17	1151.01	1273.63	1334.06	1210.41	0.06	0.06	0.06
Myanmar	1174.74	1220.67	1126.94	1727.95	1818.1	1633.66	0.51	0.52	0.49
Namibia	1527.96	1615.99	1436.11	1752.95	1870.29	1629.34	0.15	0.16	0.14
Nepal	1017.54	1122.65	910.9	1536.24	1560.29	1510.67	0.52	0.40	0.68
Netherlands	2861.38	3224.09	2490.44	3563.1	3794.25	3328.08	0.23	0.17	0.32
New Zealand	2334.06	2267.65	2402.51	2905.6	2694.4	3124.87	0.23	0.17	0.28
Nicaragua	1133.05	1215.88	1048.96	1878.82	1978.48	1776.32	0.67	0.63	0.71

Niger	920.69	916.3	925.23	1001.67	929.14	1073.02	0.10	0.02	0.18
Nigeria	1049.31	1048.39	1050.2	1050.81	1018.67	1081.75	0.00	-0.03	0.03
North Korea	944.79	1211.46	662.36	1656.43	2036.1	1259.65	0.76	0.69	0.91
Northern Mariana Islands	521.05	484.89	553.55	556.12	465.28	647.61	0.08	-0.03	0.20
Norway	3534.45	3918.36	3141.88	3339.11	3621.52	3059.98	-0.06	-0.08	-0.04
Oman	692.62	775.4	626.58	927.08	995.85	892.21	0.33	0.28	0.42
Pakistan	1115	1032.97	1191.14	1365.3	1362.73	1367.73	0.23	0.32	0.15
Palestine	720.54	730.46	710.85	962.48	973.74	951.54	0.32	0.32	0.32
Panama	1618.25	1631.84	1604.97	2758.78	2781.19	2736.5	0.73	0.73	0.74
Papua New Guinea	535.02	575.26	496.61	745.85	808.33	685.82	0.40	0.41	0.38
Paraguay	1826.31	1881.81	1772.07	2755.34	2741.87	2768.41	0.49	0.44	0.55
Peru	1374.51	1391.73	1357.29	2341.98	2402.79	2281.09	0.71	0.73	0.68
Philippines	844.85	903.86	787.07	1333.92	1437.93	1231.83	0.59	0.60	0.58
Poland	3309.85	3782.62	2812.62	4927.57	5581.34	4223.97	0.47	0.46	0.49
Portugal	3158.33	3428.56	2867.69	4204.05	4624.75	3755.43	0.31	0.33	0.28
Puerto Rico	2908.24	2889.54	2928.13	4391.75	4660.6	4101.32	0.50	0.60	0.39
Qatar	666.48	557.16	720.45	727.57	673.31	747.87	0.08	0.19	0.03
Romania	3330.32	3613.59	3038.91	5377.08	5938.67	4779.33	0.60	0.63	0.56
Russia	2918.43	3657.95	2078.64	4140.65	4943.21	3198.16	0.41	0.34	0.52
Rwanda	796.41	814.4	777.77	990.41	1060.83	913.81	0.25	0.31	0.18
Saint Lucia	1968.32	1987.72	1948.36	2801.32	2834.37	2767	0.44	0.44	0.45
Saint Vincent and the Grenadines	1707.02	1813.41	1599.56	2420.73	2433.4	2408.3	0.43	0.34	0.52
Samoa	942.59	1016.44	874.52	1358.35	1494.17	1230.81	0.43	0.46	0.40
Sao Tome and Principe	1575.88	1562.81	1589.2	1241.91	1280.37	1203.17	-0.21	-0.18	-0.24
Saudi Arabia	836.62	882.68	800.39	1195.32	1138.14	1239.25	0.41	0.27	0.52
Senegal	1046.87	1038.63	1055.19	1064.67	1106.57	1021.23	0.02	0.07	-0.02
Serbia	3041.48	3290.16	2786.11	4997.18	5416.27	4557.62	0.63	0.63	0.62
Seychelles	1809.61	2031.62	1586.65	2410.92	2663.64	2164.9	0.33	0.31	0.36
Sierra Leone	1209.4	1114.16	1306.62	1057.8	1002.92	1113.81	-0.11	-0.09	-0.13
Singapore	1257.8	1454.74	1065.99	2410.33	2829.11	1976.62	0.87	0.91	0.81
Slovakia	2824.71	3155.75	2478.02	4028.18	4614.69	3409.31	0.41	0.45	0.36
Slovenia	3270.8	3827.51	2680.49	5184.2	5802.76	4554.53	0.56	0.50	0.67
Solomon Islands	646.7	598.25	692.57	789.77	769.68	809.24	0.22	0.29	0.17
Somalia	1129.89	1130.68	1129.08	1010.61	1003.73	1017.57	-0.10	-0.11	-0.10
South Africa	2032.84	2221.59	1839.7	2624.16	2946.71	2290.22	0.29	0.32	0.24
South Korea	896.83	1039.76	754.53	2103.85	2438.56	1765.08	1.28	1.28	1.26
South Sudan	1111.32	1101.2	1121.53	1252.02	1235.41	1268.59	0.13	0.12	0.13
Spain	3084.81	3508.59	2643.3	3690.96	4212.75	3155.7	0.19	0.19	0.18
Sri Lanka	1412.11	1362.06	1461.19	2396.45	2506.03	2278.65	0.72	0.86	0.59
Sudan	832.9	820.08	845.62	991.79	993.63	989.98	0.20	0.21	0.18
Suriname	1495.94	1529.7	1463.25	2232.21	2351.7	2113.17	0.49	0.53	0.44
Swaziland	1266.67	1304.25	1224.63	1570.67	1677.84	1461.24	0.24	0.28	0.19
Sweden	3980.33	4307.61	3645.12	3946.38	4163.01	3729.31	-0.02	-0.04	0.01
Switzerland	3276.34	3678.81	2854.85	3654.46	3980.85	3320.71	0.11	0.08	0.15
Syria	912.44	877.74	946.04	1331.3	1364.61	1298.81	0.45	0.55	0.36
Taiwan	1131.49	1148.79	1115.42	2335.74	2491.2	2179.77	1.11	1.19	1.02
Tajikistan	1160.66	1272.42	1047.56	1100.86	1090.11	1111.35	-0.05	-0.15	0.06
Tanzania	1063.34	1084.13	1042.18	1169.81	1131.89	1208.2	0.12	0.06	0.18
Thailand	1290.34	1331.23	1248.69	2875.24	2984.18	2763.18	1.23	1.23	1.21
The Bahamas	1481.12	1582.98	1377.84	2813.65	2967.85	2652.85	0.88	0.85	0.91
The Gambia	929.27	808.1	1048.71	920.63	836.31	1006.61	-0.01	0.04	-0.04
Timor-Leste	595.41	604.1	586.93	1184.55	1204.26	1165.45	1.01	1.01	1.00
Togo	994.28	1005.84	982.42	991.53	994.77	988.21	0.01	-0.01	0.02
Tonga	1084.88	1155.84	1015.64	1471.79	1649.12	1295.42	0.35	0.42	0.27
Trinidad and Tobago	1738.38	1805.54	1670.72	3015.62	3129.9	2898.31	0.72	0.71	0.72
Tunisia	1400.18	1350.87	1448.9	2431.95	2481.26	2381.48	0.71	0.81	0.62
Turkey	1755.5	1785.16	1725.11	2339.36	2509.14	2163.57	0.32	0.40	0.25
Turkmenistan	1207.86	1386.78	1023.93	1553.45	1695.82	1406.22	0.28	0.21	0.36
Uganda	878.01	880.93	875.05	798.82	814.51	783.12	-0.08	-0.07	-0.09
Ukraine	3526.71	4248.6	2686.65	4909.64	5770.37	3893	0.38	0.35	0.44
United Arab Emirates	529.03	483.97	552.43	745.64	528.24	824.88	0.39	0.08	0.47

United Kingdom	3565.3	3937.99	3171.08	3700.62	3907.67	3488.4	0.03	-0.01	0.09
United States	1850.29	1942.03	1753.81	2437.32	2512.79	2359.29	0.31	0.29	0.33
Uruguay	2351.52	2484.69	2210.15	2716.66	3063.44	2345.18	0.15	0.22	0.05
Uzbekistan	1166.08	1329.11	999.43	1510.93	1640.35	1377.26	0.29	0.22	0.37
Vanuatu	856.07	760.26	946.66	1033.52	1029.01	1037.92	0.17	0.32	0.05
Venezuela	1412.82	1466.87	1359.6	2433.06	2527.32	2338	0.70	0.71	0.69
Vietnam	1372.14	1558.32	1180.22	1949.13	2245.68	1646.19	0.45	0.46	0.44
Virgin Islands, U.S.	2006.67	2004.24	2009.25	5070.66	5079.72	5060.7	1.50	1.51	1.49
Yemen	729.57	784.53	673.61	845.63	851.53	839.86	0.16	0.08	0.24
Zambia	976.37	989.68	962.83	1052.86	1082.57	1023.07	0.09	0.11	0.08
Zimbabwe	1256.38	1272.13	1240.49	1235.23	1240	1230.32	-0.01	-0.02	0.00

eTable 4b. All-ages mortality rate for men and women separately and combined, for all CVD level 3 and 4 subcauses, by country in 1990 and 2015									
	1990			2015			Median percent change between 1990-2015		
	Both	Female	Male	Both	Female	Male	Both	Female	Male
**Aortic aneurysm**									
Afghanistan	0.56	0.3	0.8	0.78	0.45	1.09	1.10	1.17	1.05
Albania	0.68	0.44	0.91	1.58	1.09	2.08	0.69	0.82	0.64
Algeria	0.46	0.33	0.59	0.77	0.64	0.9	0.87	1.16	0.73
American Samoa	1.73	1.68	1.77	1.32	1.39	1.25	-0.04	-0.17	0.05
Andorra	6.56	4.41	8.84	8.32	5.4	11.37	0.05	0.11	0.02
Angola	0.9	0.54	1.27	0.92	0.68	1.15	1.22	1.54	1.16
Antigua and Barbuda	2.05	1.75	2.36	2.29	2.01	2.61	0.25	0.33	0.18
Argentina	6.26	3.99	8.62	6.78	5.21	8.42	0.06	0.16	0.06
Armenia	4.62	3.42	5.9	9.44	6.97	12.28	0.57	0.61	0.59
Australia	8.48	5.63	11.35	6.73	5.45	8.01	-0.17	-0.02	-0.23
Austria	5.62	4.37	6.98	5.28	4.46	6.14	0.06	0.16	-0.01
Azerbaijan	0.86	0.37	1.38	1.28	0.53	2.03	0.83	0.91	0.75
Bahrain	0.36	0.18	0.49	0.32	0.18	0.4	0.55	0.72	0.48
Bangladesh	0.29	0.19	0.39	0.58	0.38	0.77	2.39	2.81	2.12
Barbados	3.52	2.37	4.76	4.57	3.4	5.84	0.07	0.17	0.05
Belarus	1.84	1.26	2.49	4.49	2.85	6.38	0.79	0.80	0.74
Belgium	7.65	4.62	10.81	7.57	4.76	10.48	0.04	0.07	0.03
Belize	0.96	0.89	1.02	1.28	1.05	1.51	0.51	0.55	0.49
Benin	0.61	0.57	0.64	0.41	0.25	0.58	0.18	-0.23	0.51
Bermuda	8.33	4.15	12.57	8.56	5.06	12.13	0.21	0.37	0.18
Bhutan	0.32	0.21	0.42	0.77	0.43	1.06	3.43	3.36	3.14
Bolivia	0.61	0.44	0.78	1.12	0.88	1.36	1.81	1.93	1.82
Bosnia and Herzegovina	1.91	1.13	2.7	3.79	2.4	5.19	0.58	0.64	0.60
Botswana	1.11	0.79	1.44	1.74	0.89	2.59	0.07	-0.17	0.17
Brazil	2.27	1.21	3.34	4.79	3.6	6.02	0.96	1.67	0.70
Brunei	1.62	1.53	1.69	2.66	2.75	2.58	0.51	0.65	0.40
Bulgaria	2.17	1.3	3.07	3.89	2.61	5.23	0.46	0.56	0.45
Burkina Faso	0.77	0.82	0.73	0.45	0.38	0.53	0.11	-0.13	0.38
Burundi	1.51	0.88	2.15	0.67	0.45	0.91	0.09	0.21	0.04
Cambodia	0.59	0.43	0.76	0.98	0.75	1.22	2.27	2.56	2.16
Cameroon	1.05	1.02	1.09	0.57	0.36	0.78	-0.34	-0.56	-0.15
Canada	7.15	4.64	9.7	5.95	4.46	7.46	-0.24	-0.18	-0.25
Cape Verde	0.38	0.18	0.61	0.61	0.29	0.94	1.07	1.09	1.05
Central African Republic	1.45	0.76	2.16	1.6	0.83	2.39	0.29	0.20	0.30
Chad	0.59	0.6	0.59	0.36	0.24	0.48	-0.10	-0.40	0.16
Chile	2.42	1.93	2.92	4.34	4.11	4.59	0.65	0.76	0.58
China	0.64	0.37	0.89	0.97	0.59	1.33	0.71	1.09	0.51
Colombia	1.92	0.97	2.89	2.69	1.63	3.77	0.52	0.69	0.51
Comoros	1.15	0.58	1.71	0.74	0.44	1.04	0.43	0.61	0.40
Congo	1.61	1	2.22	1.37	1.2	1.54	0.27	0.49	0.25
Costa Rica	1.34	0.56	2.11	2.64	1.34	3.94	0.58	0.87	0.53
Cote d'Ivoire	0.82	0.6	1.02	0.51	0.24	0.77	-0.17	-0.45	-0.03
Croatia	2.52	1.62	3.48	7.07	4.54	9.79	1.46	1.30	1.64
Cuba	5.52	2.93	8.06	7.82	4.86	10.74	0.05	0.18	0.02
Cyprus	7.11	3.37	10.95	5.04	2.52	7.73	-0.05	0.05	-0.10
Czech Republic	2.83	1.62	4.11	5.13	3.79	6.51	1.17	1.72	0.96
Democratic Republic of the Congo	0.58	0.47	0.69	0.7	0.49	0.91	0.89	0.65	1.07
Denmark	11.17	6.63	15.84	11.24	7.61	14.92	0.26	0.43	0.19
Djibouti	1.03	0.5	1.55	1.54	0.73	2.34	0.73	0.75	0.71
Dominica	2.73	2.64	2.82	5.33	4.68	5.98	0.48	0.52	0.45
Dominican Republic	0.75	0.4	1.1	1.55	1.03	2.08	0.79	1.19	0.65
Ecuador	1.03	0.69	1.36	1.94	1.36	2.52	0.89	0.99	0.84
Egypt	1.66	1.5	1.82	2.1	1.86	2.33	0.81	0.92	0.74
El Salvador	0.63	0.25	1.04	1.18	0.46	2	0.99	0.87	1.10
Equatorial Guinea	1.56	0.93	2.16	1.45	0.95	1.92	1.05	1.20	1.00
Eritrea	0.76	0.43	1.1	0.86	0.59	1.13	0.84	0.99	0.80
Estonia	3.54	2.49	4.74	5.42	4.04	7.03	0.64	0.65	0.70
Ethiopia	1.12	0.51	1.73	0.74	0.45	1.04	1.04	1.50	0.92
Federated States of Micronesia	1.94	1.35	2.49	2.51	2.04	2.96	0.49	0.66	0.40
Fiji	2.26	1.62	2.87	4.49	3.86	5.1	0.40	0.56	0.32
Finland	10.33	6.14	14.78	11.17	7.76	14.69	0.12	0.28	0.05
France	5.75	3.9	7.7	6.49	4.4	8.71	0.09	0.07	0.11
Gabon	2.83	1.66	4.04	2.29	1.71	2.85	0.31	0.59	0.20
Georgia	1.09	0.65	1.57	4.14	1.68	6.82	1.95	1.18	2.15
Germany	6.16	4.65	7.78	6.64	5.39	7.93	0.11	0.23	0.01
Ghana	1.03	1.36	0.7	0.75	0.77	0.72	0.08	-0.10	0.43

Greece	6.38	4.12	8.7	11.11	7	15.42	0.43	0.36	0.50
Greenland	1.52	1.24	1.75	1.72	1.35	2.05	0.36	0.43	0.32
Grenada	3.28	1.9	4.69	4.46	2.73	6.18	0.38	0.45	0.34
Guam	3.52	1.15	5.59	6.37	3.52	9.13	-0.05	0.52	-0.12
Guatemala	0.55	0.23	0.87	0.63	0.27	1	0.70	0.72	0.72
Guinea	0.57	0.6	0.53	0.38	0.22	0.53	0.12	-0.35	0.60
Guinea-Bissau	0.88	0.81	0.95	0.49	0.29	0.7	-0.17	-0.46	0.08
Guyana	1.19	0.87	1.52	2.25	1.55	2.95	0.86	0.83	0.87
Haiti	1.22	0.97	1.48	1.37	1.1	1.64	0.83	0.81	0.88
Honduras	0.33	0.23	0.43	0.77	0.33	1.22	1.54	0.50	2.26
Hungary	3.65	2.86	4.5	5.12	4.37	5.96	0.63	0.62	0.67
Iceland	4.67	3.49	5.85	4.8	3.29	6.34	0.19	0.19	0.17
India	0.47	0.27	0.65	1.28	0.7	1.82	2.81	2.92	2.60
Indonesia	0.63	0.44	0.81	1.21	0.88	1.54	1.38	1.56	1.27
Iran	0.27	0.14	0.39	0.6	0.36	0.85	2.28	3.22	1.93
Iraq	1.72	1.73	1.72	1.9	1.81	1.99	0.37	0.46	0.33
Ireland	5.55	4.1	7.02	6.01	4.48	7.58	0.37	0.46	0.31
Israel	2.22	1.54	2.91	2.38	1.73	3.03	0.15	0.16	0.18
Italy	4.7	2.41	7.13	7.59	4.63	10.74	0.42	0.58	0.43
Jamaica	1.55	1.49	1.61	2.93	2.53	3.34	0.48	0.41	0.54
Japan	4.89	4.18	5.62	14.82	14.75	14.88	0.86	1.08	0.68
Jordan	0.83	0.48	1.14	0.75	0.47	1.02	0.57	0.89	0.41
Kazakhstan	2.63	1.93	3.37	2.81	1.71	3.99	0.06	-0.02	0.10
Kenya	0.5	0.33	0.68	0.7	0.41	0.98	0.83	0.75	0.83
Kiribati	0.75	0.58	0.93	0.98	0.88	1.08	0.64	1.12	0.36
Kuwait	0.35	0.21	0.47	0.53	0.29	0.72	1.06	0.85	1.12
Kyrgyzstan	0.57	0.43	0.71	0.52	0.36	0.68	0.20	0.24	0.15
Laos	0.8	0.56	1.04	0.81	0.61	1.01	1.58	1.94	1.41
Latvia	3.2	1.94	4.65	4.26	2.76	6.02	0.35	0.40	0.36
Lebanon	0.95	0.58	1.33	0.99	0.76	1.22	0.55	0.68	0.55
Lesotho	1.26	0.83	1.72	1.86	1.07	2.67	-0.16	-0.27	-0.07
Liberia	0.6	0.56	0.64	0.29	0.19	0.39	0.14	-0.27	0.59
Libya	0.34	0.24	0.43	0.69	0.46	0.91	0.69	0.81	0.62
Lithuania	2.44	1.58	3.39	4.4	2.82	6.25	0.57	0.53	0.61
Luxembourg	6.54	4.26	8.91	4.67	3.53	5.81	0.08	0.19	0.04
Macedonia	1.04	0.54	1.54	2.08	1.14	3.02	0.71	0.75	0.74
Madagascar	1.43	0.74	2.12	1.49	0.83	2.14	0.78	0.90	0.71
Malawi	0.48	0.34	0.63	0.57	0.35	0.79	1.33	1.03	1.45
Malaysia	1.78	0.91	2.63	2.52	1.16	3.9	0.32	0.41	0.24
Maldives	0.9	0.57	1.22	0.92	0.55	1.28	1.42	1.87	1.18
Mali	0.69	0.72	0.67	0.24	0.2	0.28	-0.36	-0.49	-0.22
Malta	2.85	1.35	4.41	3.88	2.07	5.72	0.18	0.27	0.18
Marshall Islands	1.48	0.92	2.01	2.25	1.55	2.92	0.51	0.57	0.50
Mauritania	0.77	0.68	0.85	0.32	0.25	0.38	-0.20	-0.33	-0.08
Mauritius	0.95	0.61	1.3	1.29	0.76	1.83	0.08	-0.07	0.18
Mexico	0.63	0.27	0.98	0.8	0.38	1.22	0.23	0.25	0.26
Moldova	0.92	0.61	1.26	1.49	1.04	1.98	0.71	0.86	0.62
Mongolia	0.43	0.36	0.49	0.62	0.56	0.67	0.78	1.24	0.53
Montenegro	5.57	3.74	7.4	10.42	6.32	14.62	0.53	0.55	0.45
Morocco	0.61	0.51	0.72	1.09	0.92	1.25	1.04	1.08	1.04
Mozambique	0.54	0.28	0.83	0.87	0.42	1.33	1.77	1.65	1.76
Myanmar	0.81	0.75	0.88	1.47	1.35	1.6	1.73	1.93	1.66
Namibia	1.58	0.97	2.21	1.26	0.57	1.99	-0.08	-0.29	-0.01
Nepal	0.27	0.18	0.37	0.57	0.31	0.85	2.68	2.45	2.66
Netherlands	9.63	5.39	13.96	10.21	7.16	13.31	0.08	0.30	0.01
New Zealand	11.24	8.23	14.35	9.24	7.9	10.64	-0.08	0.02	-0.14
Nicaragua	0.31	0.15	0.47	0.6	0.3	0.91	1.27	1.37	1.25
Niger	0.38	0.36	0.4	0.2	0.12	0.28	0.19	-0.20	0.51
Nigeria	0.7	0.71	0.69	0.32	0.2	0.44	-0.13	-0.43	0.17
North Korea	0.64	0.53	0.75	1.15	0.92	1.38	0.34	0.34	0.34
Northern Mariana Islands	1.57	2.05	1.15	0.93	0.91	0.96	-0.17	-0.22	0.00
Norway	13.14	8.44	17.95	10.43	8.33	12.5	0.06	0.20	0.03
Oman	0.44	0.16	0.67	0.6	0.2	0.81	1.37	1.33	1.07
Pakistan	0.53	0.21	0.83	1.04	0.43	1.62	1.53	1.94	1.38
Palestine	0.48	0.42	0.54	0.74	0.56	0.92	1.05	1.03	1.07
Panama	1.6	0.77	2.4	2.13	1.12	3.12	0.13	0.23	0.11
Papua New Guinea	1.22	0.93	1.49	1.5	1.2	1.78	0.49	0.50	0.49
Paraguay	1.21	0.69	1.73	2.72	1.64	3.77	0.75	1.02	0.61
Peru	0.67	0.46	0.88	0.93	0.74	1.13	1.15	1.36	1.09
Philippines	0.82	0.55	1.09	1.06	0.77	1.34	0.37	0.48	0.31

Poland	4.24	2	6.6	5.31	2.97	7.82	0.29	0.51	0.24
Portugal	2.86	1.92	3.87	3.93	2.47	5.49	0.47	0.37	0.52
Puerto Rico	2.37	1.27	3.53	2.51	1.9	3.18	-0.09	0.08	-0.11
Qatar	0.38	0.16	0.49	0.29	0.14	0.34	0.35	0.68	0.23
Romania	2.06	1.69	2.45	3.76	3.32	4.22	0.48	0.59	0.40
Russia	0.86	0.65	1.11	5.05	3.38	7.02	3.82	3.59	3.87
Rwanda	0.9	0.64	1.16	0.71	0.54	0.9	0.85	0.93	0.81
Saint Lucia	4.45	4.13	4.78	6.72	6.91	6.53	0.42	0.53	0.33
Saint Vincent and the Grenadines	3.59	1.9	5.29	3.21	2.4	4	-0.25	0.17	-0.43
Samoa	1.71	1.25	2.13	2.32	2.39	2.25	0.30	0.46	0.25
Sao Tome and Principe	0.98	0.83	1.14	0.61	0.4	0.81	0.07	-0.17	0.26
Saudi Arabia	0.2	0.11	0.28	0.31	0.14	0.45	1.60	1.68	1.42
Senegal	0.63	0.55	0.7	0.38	0.24	0.52	0.06	-0.24	0.30
Serbia	2.81	1.52	4.14	5.87	3.87	7.97	0.61	0.91	0.52
Seychelles	2.01	1.09	2.94	2.02	1.39	2.63	0.11	0.37	0.02
Sierra Leone	0.55	0.39	0.72	0.35	0.22	0.48	0.10	-0.04	0.24
Singapore	2.34	1.42	3.23	3.11	2.5	3.74	0.16	0.40	0.09
Slovakia	1.92	1.43	2.43	2.91	2.03	3.84	0.77	0.51	1.00
Slovenia	2.65	1.81	3.54	4.63	3.2	6.08	0.75	0.66	0.83
Solomon Islands	1.43	0.88	1.95	1.81	1.3	2.3	0.51	0.66	0.46
Somalia	1.15	0.56	1.75	0.83	0.41	1.26	0.14	0.13	0.16
South Africa	2.35	1.67	3.06	2.39	1.4	3.41	-0.13	-0.25	-0.08
South Korea	1.37	1.38	1.35	2.81	2.93	2.68	0.79	0.82	0.76
South Sudan	0.81	0.51	1.12	0.85	0.52	1.19	0.68	0.62	0.73
Spain	3.84	2.11	5.64	5.36	2.89	7.89	0.42	0.32	0.51
Sri Lanka	1.2	0.76	1.63	1.89	1.4	2.42	0.69	0.78	0.68
Sudan	0.52	0.31	0.72	0.65	0.4	0.89	1.23	1.36	1.17
Suriname	1.36	0.68	2.02	2.14	1.08	3.19	0.45	0.54	0.41
Swaziland	1.28	0.84	1.77	1.89	0.97	2.84	-0.05	-0.27	0.05
Sweden	13.39	8.88	18.01	12.49	9.82	15.16	0.10	0.22	0.06
Switzerland	7.15	4.43	10	6.67	4.8	8.58	0.10	0.21	0.06
Syria	0.7	0.49	0.9	0.89	0.72	1.06	-0.01	0.50	-0.23
Taiwan	2.22	1.02	3.33	3.61	2.39	4.83	0.25	0.77	0.13
Tajikistan	0.36	0.23	0.5	0.39	0.23	0.55	1.01	1.04	0.93
Tanzania	0.63	0.41	0.86	0.78	0.44	1.12	1.11	0.83	1.19
Thailand	0.88	0.47	1.31	3.07	2	4.16	1.21	1.90	0.95
The Bahamas	2.36	2.02	2.71	4.16	4.1	4.23	0.54	0.66	0.45
The Gambia	0.5	0.49	0.52	0.26	0.15	0.38	-0.04	-0.36	0.26
Timor-Leste	0.36	0.28	0.44	0.64	0.56	0.71	2.75	3.15	2.59
Togo	0.69	0.66	0.73	0.43	0.21	0.65	-0.14	-0.49	0.12
Tonga	0.94	0.26	1.61	1.52	0.46	2.57	0.46	0.61	0.43
Trinidad and Tobago	3.54	2.37	4.72	6.27	4.62	7.98	0.46	0.69	0.34
Tunisia	0.65	0.36	0.95	1.06	0.66	1.47	0.63	0.97	0.51
Turkey	2.15	1.17	3.17	2.03	1.3	2.77	0.66	0.98	0.55
Turkmenistan	0.47	0.29	0.65	0.97	0.55	1.4	1.91	1.92	1.83
Uganda	0.75	0.54	0.97	0.9	0.52	1.28	1.38	0.99	1.50
Ukraine	1.94	1.34	2.64	3.58	2.41	4.96	0.49	0.52	0.45
United Arab Emirates	0.7	0.28	0.92	1.22	0.41	1.52	1.40	1.61	1.16
United Kingdom	15.81	11.32	20.56	13.36	10.74	16.04	0.02	0.13	-0.04
United States	6.35	4.19	8.61	4.11	3.28	4.98	-0.33	-0.22	-0.38
Uruguay	8.04	5.61	10.61	9.6	7.91	11.4	0.08	0.12	0.10
Uzbekistan	0.28	0.19	0.36	0.4	0.26	0.55	0.70	0.73	0.66
Vanuatu	1.89	1.06	2.67	2.19	1.56	2.8	0.30	0.51	0.24
Venezuela	1.29	0.67	1.91	1.86	1.14	2.58	0.17	0.47	0.05
Vietnam	0.79	0.6	0.99	1.8	1.45	2.15	1.41	1.63	1.27
Virgin Islands, U.S.	2.41	1.5	3.38	5.12	3.55	6.84	-0.01	0.11	-0.06
Yemen	0.39	0.24	0.54	0.59	0.35	0.82	2.17	2.13	2.17
Zambia	0.61	0.51	0.72	1.15	0.59	1.72	1.72	0.81	2.21
Zimbabwe	0.4	0.24	0.56	0.71	0.29	1.14	0.47	0.03	0.63
**Atrial fibrillation and flutter**									
Afghanistan	0.56	0.63	0.5	0.81	0.93	0.69	1.01	1.16	0.87
Albania	2.24	2.49	1.99	4.79	5.18	4.4	0.56	0.52	0.58
Algeria	0.82	0.92	0.73	1.86	1.91	1.82	1.45	1.24	1.72
American Samoa	0.86	0.94	0.78	0.62	0.79	0.44	-0.10	-0.17	-0.15
Andorra	5.95	7.26	4.57	9.53	11.24	7.75	0.31	0.33	0.34
Angola	0.25	0.25	0.25	0.27	0.25	0.29	1.15	0.92	1.42
Antigua and Barbuda	1.92	2.03	1.8	1.98	1.99	1.96	0.14	0.13	0.16
Argentina	3.14	3.25	3.02	4.15	4.7	3.58	0.30	0.27	0.28
Armenia	2.1	2.51	1.66	4.79	5.61	3.84	0.73	0.70	0.73
Australia	3.27	3.69	2.86	4.91	5.17	4.65	0.57	0.41	0.77

Austria	7.87	10.52	4.99	13.73	17.35	9.92	0.97	0.87	1.23
Azerbaijan	1.13	1.44	0.8	2.03	2.29	1.77	1.21	1.07	1.59
Bahrain	0.55	0.55	0.55	0.51	0.65	0.43	0.61	0.97	0.38
Bangladesh	0.44	0.36	0.51	0.91	0.65	1.16	2.53	2.47	2.50
Barbados	2.66	2.74	2.58	4.09	4.13	4.04	0.26	0.20	0.33
Belarus	3.61	4.83	2.23	5.39	7.13	3.37	0.08	0.15	0.02
Belgium	5.75	7.57	3.86	8.43	10.64	6.14	0.53	0.46	0.68
Belize	1.41	1.22	1.59	1.05	0.84	1.26	-0.18	-0.13	-0.21
Benin	0.33	0.33	0.32	0.24	0.24	0.24	0.25	0.27	0.23
Bermuda	1.43	1.26	1.61	2.19	1.98	2.41	0.80	0.76	0.82
Bhutan	0.57	0.46	0.67	1.09	0.79	1.35	2.40	2.60	2.16
Bolivia	0.91	0.83	1	2.07	1.87	2.28	2.44	2.30	2.61
Bosnia and Herzegovina	3.38	3.88	2.89	6.38	7.29	5.45	0.44	0.37	0.51
Botswana	0.21	0.21	0.2	0.29	0.28	0.29	-0.07	-0.08	-0.05
Brazil	0.72	0.72	0.72	1.95	1.96	1.94	1.51	1.46	1.54
Brunei	0.46	0.31	0.59	0.69	0.5	0.87	0.36	0.44	0.33
Bulgaria	4.79	5.07	4.5	7.95	9.16	6.68	0.35	0.40	0.26
Burkina Faso	0.37	0.35	0.39	0.34	0.36	0.31	0.68	0.85	0.52
Burundi	0.41	0.37	0.44	0.36	0.3	0.41	1.01	0.93	1.08
Cambodia	0.73	0.88	0.57	1.14	1.23	1.05	2.00	1.83	2.40
Cameroon	0.42	0.38	0.46	0.35	0.31	0.39	0.04	0.04	0.03
Canada	3.11	3.44	2.78	4.92	5.25	4.59	0.44	0.29	0.60
Cape Verde	0.58	0.54	0.62	0.66	0.6	0.71	0.45	0.43	0.49
Central African Republic	0.57	0.56	0.58	0.64	0.62	0.66	0.29	0.25	0.34
Chad	0.32	0.33	0.32	0.23	0.23	0.23	0.00	-0.01	0.00
Chile	2.78	2.75	2.81	4.84	5.2	4.47	0.59	0.55	0.60
China	0.98	1.12	0.86	2.19	2.23	2.15	1.52	1.64	1.54
Colombia	0.94	0.88	1	1.68	1.59	1.77	0.94	0.81	1.04
Comoros	0.39	0.35	0.42	0.37	0.31	0.42	0.98	0.87	1.11
Congo	0.47	0.43	0.5	0.52	0.46	0.58	0.64	0.34	1.03
Costa Rica	1.2	1.08	1.31	2.35	2.15	2.55	0.57	0.56	0.59
Cote d'Ivoire	0.25	0.21	0.29	0.23	0.17	0.29	0.23	0.14	0.30
Croatia	3.5	4.2	2.75	5.29	6.29	4.2	0.33	0.23	0.43
Cuba	2.49	2.11	2.87	4.31	4.01	4.6	0.27	0.33	0.22
Cyprus	5.99	6.59	5.38	6.21	7.12	5.23	0.38	0.51	0.24
Czech Republic	5.54	6.91	4.09	9	10.78	7.16	0.95	0.81	1.17
Democratic Republic of the Congo	0.34	0.36	0.32	0.49	0.47	0.51	1.23	1.09	1.42
Denmark	4.56	5.86	3.23	6.44	7.84	5.02	0.77	0.66	0.97
Djibouti	0.31	0.28	0.34	0.51	0.41	0.6	0.83	0.74	0.94
Dominica	1.95	1.69	2.22	2.9	2.69	3.11	0.12	0.37	-0.05
Dominican Republic	0.98	0.77	1.18	1.97	1.66	2.28	0.74	0.84	0.68
Ecuador	1.02	0.89	1.15	1.82	1.61	2.03	0.78	0.82	0.75
Egypt	1.21	1.48	0.94	1.35	1.61	1.09	0.56	0.65	0.52
El Salvador	1.08	0.97	1.2	2.45	2.09	2.86	1.41	1.19	1.59
Equatorial Guinea	0.72	0.69	0.75	0.31	0.26	0.37	-0.09	-0.20	0.00
Eritrea	0.19	0.21	0.16	0.29	0.32	0.27	1.48	1.31	1.72
Estonia	3.94	5.33	2.35	7.08	9.37	4.38	0.92	0.77	1.12
Ethiopia	0.39	0.3	0.48	0.45	0.34	0.55	2.37	2.29	2.45
Federated States of Micronesia	1.1	1.06	1.14	1.46	1.51	1.42	0.51	0.57	0.45
Fiji	0.62	0.59	0.64	1.13	1.17	1.08	0.27	0.28	0.25
Finland	7.97	10.83	4.93	15.39	20.05	10.58	0.99	0.87	1.25
France	7.88	10.29	5.35	12.1	15.33	8.67	0.48	0.41	0.59
Gabon	0.67	0.61	0.74	0.71	0.64	0.77	0.67	0.58	0.76
Georgia	2.81	3.57	1.97	6.15	7.58	4.59	0.69	0.78	0.68
Germany	8.2	11.03	5.15	12.02	14.86	9.08	0.50	0.42	0.75
Ghana	0.21	0.23	0.2	0.34	0.39	0.28	1.38	1.79	1.02
Greece	5.66	6.34	4.96	11.07	12.89	9.17	0.61	0.62	0.56
Greenland	0.63	0.66	0.6	1	0.84	1.14	0.89	0.67	1.12
Grenada	1.92	1.93	1.9	2.28	2.45	2.12	0.18	0.25	0.11
Guam	0.81	0.91	0.72	1.84	1.93	1.75	0.19	0.05	0.30
Guatemala	0.73	0.61	0.86	1.39	1.23	1.55	1.80	1.95	1.69
Guinea	0.27	0.26	0.29	0.27	0.25	0.3	0.67	0.66	0.67
Guinea-Bissau	0.33	0.31	0.34	0.27	0.24	0.3	0.18	0.10	0.26
Guyana	1.36	1.16	1.57	1.03	0.89	1.18	-0.26	-0.22	-0.28
Haiti	1.02	0.94	1.11	1.4	1.33	1.47	1.23	1.27	1.21
Honduras	0.89	0.8	0.99	1.72	1.5	1.93	1.00	0.81	1.20
Hungary	4.36	5.31	3.32	6.33	8.12	4.36	0.68	0.62	0.66
Iceland	7.87	8.51	7.22	11.14	11.99	10.27	0.64	0.78	0.53
India	0.69	0.61	0.77	1.28	1.08	1.47	1.55	1.70	1.42
Indonesia	0.7	0.7	0.7	1.05	1.1	1	0.84	1.02	0.69

Iran	0.66	0.61	0.7	1.4	1.25	1.55	1.92	2.19	1.80
Iraq	1	1.12	0.88	0.78	0.91	0.66	-0.04	0.10	-0.16
Ireland	4.9	5.68	4.1	5.08	5.78	4.36	0.32	0.36	0.29
Israel	4.15	4.57	3.73	6.22	7.53	4.9	0.62	0.69	0.48
Italy	6.34	7.9	4.68	11.93	14.55	9.15	0.66	0.51	0.84
Jamaica	2.49	2.33	2.65	3.53	3.25	3.81	0.10	0.15	0.07
Japan	2.23	1.91	2.56	6.35	5.71	7.03	0.75	0.76	0.74
Jordan	1.07	1.02	1.1	0.94	0.98	0.89	0.50	0.87	0.25
Kazakhstan	1.84	2.54	1.1	1.97	2.54	1.37	0.07	0.09	0.14
Kenya	0.28	0.24	0.32	0.34	0.28	0.4	0.57	0.60	0.53
Kiribati	1.11	1.32	0.89	1.31	1.61	1	0.49	0.70	0.31
Kuwait	0.29	0.3	0.29	0.3	0.28	0.33	0.40	0.25	0.57
Kyrgyzstan	1.79	2.35	1.2	1.98	2.36	1.6	0.44	0.47	0.57
Laos	0.88	0.95	0.81	0.94	0.97	0.92	1.62	1.66	1.63
Latvia	4.19	5.53	2.65	6.49	8.67	3.95	0.57	0.55	0.56
Lebanon	1.61	1.76	1.46	2.12	2.4	1.84	0.94	0.76	1.09
Lesotho	0.42	0.41	0.44	0.52	0.56	0.48	-0.31	-0.27	-0.34
Liberia	0.23	0.19	0.27	0.26	0.22	0.3	1.65	1.48	1.84
Libya	0.56	0.62	0.51	1.16	1.29	1.03	0.72	0.92	0.55
Lithuania	3.77	4.65	2.78	6.3	8.19	4.1	0.45	0.51	0.29
Luxembourg	6.47	8.64	4.21	8.16	10.69	5.63	0.91	0.77	1.14
Macedonia	3.36	3.46	3.27	5.53	6.22	4.84	0.40	0.48	0.30
Madagascar	0.53	0.43	0.64	0.57	0.46	0.68	0.80	0.86	0.76
Malawi	0.25	0.21	0.29	0.36	0.32	0.41	1.84	2.04	1.67
Malaysia	0.8	0.73	0.87	0.99	0.75	1.24	0.15	0.13	0.17
Maldives	0.64	0.44	0.83	1.28	0.95	1.62	3.65	5.21	2.85
Mali	0.34	0.31	0.37	0.24	0.22	0.26	0.30	0.30	0.29
Malta	4.39	5.17	3.58	7.15	9.05	5.23	0.41	0.45	0.33
Marshall Islands	0.71	0.73	0.7	0.96	0.96	0.96	0.33	0.23	0.43
Mauritania	0.25	0.25	0.26	0.25	0.25	0.25	0.85	0.82	0.89
Mauritius	0.9	1.1	0.71	2.08	2.3	1.85	0.83	0.56	1.19
Mexico	1.07	0.97	1.17	1.9	1.79	2.02	0.71	0.67	0.74
Moldova	2.52	3.08	1.9	4.29	5.27	3.23	0.80	0.86	0.75
Mongolia	1.38	1.51	1.26	1.35	1.57	1.13	0.15	0.44	-0.05
Montenegro	7.09	8.59	5.58	11.49	12.56	10.41	0.32	0.33	0.37
Morocco	0.98	1.11	0.85	1.92	2.09	1.75	1.22	1.10	1.39
Mozambique	0.34	0.31	0.38	0.41	0.36	0.47	0.86	0.81	0.91
Myanmar	1.57	2.16	0.95	1.95	2.54	1.34	0.80	0.86	0.91
Namibia	0.29	0.29	0.29	0.33	0.31	0.36	0.32	0.28	0.38
Nepal	0.55	0.51	0.59	0.91	0.68	1.16	1.91	1.61	2.11
Netherlands	8.96	11.59	6.26	12.65	15.64	9.61	0.43	0.32	0.62
New Zealand	5.92	7.42	4.37	8.65	10.21	7.04	0.63	0.46	0.87
Nicaragua	0.72	0.67	0.76	1.75	1.6	1.9	1.86	1.79	1.92
Niger	0.15	0.14	0.15	0.18	0.17	0.19	1.63	1.62	1.64
Nigeria	0.22	0.19	0.24	0.16	0.13	0.19	0.33	0.27	0.39
North Korea	0.99	1.42	0.54	2.65	3.72	1.53	1.00	1.02	1.05
Northern Mariana Islands	0.39	0.5	0.3	0.34	0.35	0.32	0.21	0.24	0.29
Norway	10.19	12.25	8.08	12.15	14.69	9.64	0.59	0.45	0.75
Oman	0.53	0.68	0.4	0.57	0.85	0.42	0.72	1.09	0.65
Pakistan	1.04	0.55	1.5	1.08	0.61	1.52	0.32	0.52	0.23
Palestine	0.57	0.63	0.52	0.79	0.88	0.7	0.81	1.08	0.63
Panama	1.25	1.08	1.41	2.07	1.81	2.34	0.41	0.41	0.41
Papua New Guinea	0.47	0.57	0.37	0.56	0.65	0.48	0.43	0.30	0.62
Paraguay	0.89	0.84	0.94	1.61	1.46	1.76	0.40	0.45	0.37
Peru	0.84	0.76	0.92	1.74	1.66	1.81	2.14	2.15	2.15
Philippines	0.7	0.71	0.69	0.99	1.07	0.9	0.50	0.60	0.40
Poland	4.88	6.12	3.57	8.71	11.01	6.22	0.84	0.83	0.82
Portugal	6.58	8.04	5.02	10.3	12.48	7.96	0.67	0.65	0.70
Puerto Rico	2.45	2.1	2.82	4.04	4.1	3.96	0.40	0.40	0.38
Qatar	0.35	0.44	0.31	0.19	0.27	0.16	-0.03	0.20	-0.08
Romania	3	3.45	2.53	5.01	6.01	3.94	0.35	0.40	0.26
Russia	3.08	4.39	1.58	4.87	6.37	3.11	0.30	0.27	0.51
Rwanda	0.31	0.3	0.32	0.41	0.36	0.46	2.01	1.81	2.21
Saint Lucia	1.82	1.69	1.95	3.16	3.05	3.26	0.62	0.64	0.62
Saint Vincent and the Grenadines	1.83	1.83	1.82	2.19	2.09	2.29	0.00	0.05	-0.05
Samoa	0.98	1.06	0.89	1.65	1.89	1.44	0.61	0.33	0.88
Sao Tome and Principe	0.53	0.5	0.57	0.51	0.42	0.6	0.54	0.31	0.78
Saudi Arabia	0.58	0.68	0.49	0.75	0.85	0.68	1.16	1.44	1.06
Senegal	0.24	0.23	0.24	0.28	0.27	0.29	1.06	1.01	1.13
Serbia	3.65	4.23	3.07	5.99	7.07	4.85	0.26	0.25	0.25

Seychelles	2.05	2.27	1.83	1.83	2.14	1.52	-0.02	0.01	-0.06
Sierra Leone	0.2	0.15	0.25	0.17	0.14	0.2	0.35	0.38	0.38
Singapore	1.14	0.84	1.42	2.46	1.85	3.09	0.88	0.73	1.05
Slovakia	4.74	5.71	3.74	7.93	10.3	5.43	0.94	0.91	0.82
Slovenia	3.9	4.91	2.83	7.66	9.55	5.73	0.96	0.82	1.16
Solomon Islands	0.85	0.66	1.04	1.01	0.86	1.16	0.38	0.47	0.35
Somalia	0.4	0.35	0.44	0.34	0.29	0.4	0.26	0.20	0.32
South Africa	0.52	0.53	0.52	0.75	0.82	0.67	0.22	0.37	0.07
South Korea	0.88	0.9	0.86	2.78	2.69	2.88	1.76	1.55	1.97
South Sudan	0.25	0.22	0.28	0.34	0.28	0.4	1.03	0.92	1.13
Spain	6.89	8.76	4.95	11.48	14.47	8.41	0.69	0.59	0.82
Sri Lanka	1.7	1.47	1.91	2.22	2.25	2.2	0.40	0.46	0.29
Sudan	0.99	1.07	0.92	1.06	1.14	0.98	0.87	0.93	0.84
Suriname	1.24	1.17	1.31	1.8	1.8	1.81	0.33	0.47	0.22
Swaziland	0.23	0.21	0.24	0.31	0.3	0.33	-0.11	-0.13	-0.08
Sweden	12.49	13.07	11.9	19.74	21.25	18.23	0.86	0.79	0.92
Switzerland	4.82	6.09	3.5	6.37	7.87	4.83	0.55	0.44	0.71
Syria	1.52	1.59	1.46	1.97	2.17	1.77	-0.02	0.38	-0.23
Taiwan	1.4	1.42	1.38	3.31	2.98	3.65	0.78	0.57	0.97
Tajikistan	1.4	1.6	1.18	1.31	1.29	1.32	0.74	0.61	0.96
Tanzania	0.28	0.24	0.32	0.38	0.26	0.5	1.29	0.89	1.56
Thailand	1.45	1.52	1.37	2.82	2.78	2.87	0.24	0.24	0.27
The Bahamas	0.86	0.87	0.84	1.87	1.98	1.76	0.89	0.83	0.92
The Gambia	0.22	0.19	0.26	0.22	0.17	0.26	0.75	0.89	0.67
Timor-Leste	0.4	0.44	0.36	0.88	0.94	0.81	3.55	3.39	3.75
Togo	0.29	0.28	0.3	0.24	0.21	0.26	0.15	0.18	0.12
Tonga	1.35	1.42	1.29	2.3	2.69	1.91	0.55	0.76	0.33
Trinidad and Tobago	1.25	1.23	1.28	1.84	1.93	1.74	0.21	0.37	0.08
Tunisia	1.31	1.37	1.26	2.44	2.64	2.24	0.85	1.01	0.71
Turkey	1.93	2.24	1.6	2.17	2.55	1.78	0.96	1.01	0.91
Turkmenistan	1.19	1.5	0.88	1.37	1.66	1.08	0.62	0.68	0.62
Uganda	0.35	0.3	0.4	0.38	0.31	0.45	1.10	1.10	1.08
Ukraine	3.82	5.11	2.32	6.53	8.36	4.37	0.38	0.38	0.46
United Arab Emirates	0.21	0.26	0.18	0.23	0.27	0.22	0.43	0.84	0.45
United Kingdom	5.44	7.34	3.43	10.08	13.15	6.94	1.25	1.15	1.49
United States	4.57	5.79	3.3	7.14	8.9	5.32	0.61	0.53	0.72
Uruguay	4.32	4.58	4.04	6.3	7.55	4.97	0.32	0.31	0.26
Uzbekistan	1.52	1.85	1.18	1.74	2.06	1.42	0.34	0.39	0.33
Vanuatu	1.21	0.92	1.48	1.05	0.93	1.16	-0.06	0.03	-0.10
Venezuela	0.74	0.69	0.78	1.4	1.4	1.4	0.55	0.76	0.39
Vietnam	1.93	2.32	1.52	2.96	3.57	2.34	0.61	0.64	0.60
Virgin Islands, U.S.	1.02	0.99	1.05	3.71	3.54	3.89	0.70	0.67	0.72
Yemen	0.78	0.92	0.63	0.84	0.9	0.79	1.07	0.93	1.32
Zambia	0.26	0.24	0.28	0.32	0.29	0.36	0.75	0.84	0.69
Zimbabwe	0.4	0.38	0.42	0.57	0.51	0.63	0.19	0.14	0.24
**Cardiomyopathy and myocarditis**									
Afghanistan	4.07	4.98	3.2	4.35	5.18	3.57	0.52	0.53	0.56
Albania	11.2	12.08	10.36	20.71	20.06	21.36	0.34	0.22	0.48
Algeria	8.52	8.2	8.83	7.55	6.91	8.19	-0.02	-0.07	0.04
American Samoa	5.36	5	5.69	5.92	5.3	6.53	0.39	0.06	0.72
Andorra	6.46	6.63	6.28	6.4	5.9	6.93	-0.17	-0.20	-0.10
Angola	4.23	4.24	4.22	3.39	3.35	3.44	0.61	0.50	0.75
Antigua and Barbuda	6.59	7.92	5.17	6.83	6.65	7.03	0.15	-0.03	0.46
Argentina	23.82	19.75	28.05	9.28	8.18	10.44	-0.62	-0.64	-0.60
Armenia	1.48	1.23	1.75	2.47	1.98	3.04	0.27	0.24	0.31
Australia	8.22	6.8	9.64	5.06	3.79	6.34	-0.35	-0.44	-0.28
Austria	53.89	69.1	37.31	19.96	22.78	17	-0.58	-0.62	-0.49
Azerbaijan	2.62	1.73	3.55	3.08	1.6	4.58	0.43	0.21	0.51
Bahrain	2.86	2.37	3.22	1.97	1.59	2.2	0.19	0.11	0.22
Bangladesh	0.46	0.43	0.49	0.62	0.62	0.62	1.32	1.80	1.02
Barbados	9.07	9.63	8.45	9.97	8.32	11.76	-0.10	-0.31	0.18
Belarus	9.4	5.41	13.92	19.44	8.8	31.79	0.51	0.29	0.56
Belgium	14.64	17.11	12.06	11.14	12.6	9.62	-0.20	-0.23	-0.15
Belize	5.2	6.69	3.73	5.19	5.05	5.33	0.10	-0.05	0.42
Benin	1.58	1.38	1.8	1.33	0.9	1.75	0.41	0.13	0.60
Bermuda	7.16	6.46	7.87	6.66	5.15	8.21	0.10	-0.11	0.27
Bhutan	0.59	0.57	0.61	0.85	0.79	0.91	1.61	1.97	1.40
Bolivia	4.48	4.55	4.4	3.47	3.68	3.26	0.19	0.21	0.20
Bosnia and Herzegovina	25.59	29.65	21.53	82.62	95.27	69.86	1.47	1.35	1.52
Botswana	5.51	5.97	5.05	8.33	7.9	8.76	-0.01	-0.09	0.10

Brazil	6.87	5.98	7.77	9.31	7.68	11	0.26	0.16	0.34
Brunei	2.99	2.2	3.7	3.24	2.2	4.23	-0.02	-0.09	0.03
Bulgaria	11.16	11.34	10.98	33.99	37.23	30.58	1.48	1.54	1.37
Burkina Faso	1.9	1.7	2.1	1.53	1.24	1.82	0.49	0.30	0.63
Burundi	3.87	3.43	4.32	1.69	1.45	1.94	0.02	0.02	0.04
Cambodia	3.78	3.78	3.77	2.48	2.23	2.74	0.30	0.25	0.38
Cameroon	2.12	1.8	2.45	1.74	1.19	2.29	0.00	-0.18	0.11
Canada	3.88	2.73	5.05	3.31	2.35	4.29	-0.22	-0.27	-0.17
Cape Verde	0.93	0.42	1.5	0.99	0.53	1.46	0.38	0.63	0.29
Central African Republic	4.43	3.96	4.91	4.55	3.73	5.4	0.16	0.04	0.24
Chad	1.45	1.37	1.53	1.26	0.97	1.56	0.25	0.03	0.44
Chile	4.02	3.49	4.56	5.05	3.76	6.38	0.15	-0.11	0.41
China	1.35	1.13	1.56	2.06	1.77	2.33	0.73	1.06	0.54
Colombia	2.47	2.12	2.82	2.11	1.84	2.4	-0.07	-0.13	-0.01
Comoros	2.79	2.36	3.21	1.53	1.28	1.78	0.14	0.12	0.17
Congo	4.48	4.21	4.76	3.64	4.05	3.22	0.19	0.19	0.18
Costa Rica	1.67	1.45	1.88	4.06	3.35	4.76	0.96	0.82	1.07
Cote d'Ivoire	2.07	1.5	2.59	1.63	0.98	2.25	0.03	-0.12	0.10
Croatia	9.68	10.64	8.66	20.04	23.56	16.27	0.82	0.82	0.76
Cuba	2.54	2.97	2.13	7.38	6.75	8	1.14	0.60	1.88
Cyprus	7.07	6.98	7.16	4.17	3.66	4.73	-0.20	-0.24	-0.13
Czech Republic	6.32	5.87	6.79	6.71	6.12	7.33	0.27	0.21	0.34
Democratic Republic of the Congo	2.53	2.67	2.38	2.34	2.18	2.51	0.43	0.29	0.61
Denmark	5.55	5.55	5.55	5.05	4.4	5.71	0.14	-0.01	0.30
Djibouti	2.46	1.83	3.07	2.84	1.9	3.77	0.28	0.19	0.33
Dominica	11.24	11.57	10.91	18.77	13.27	24.35	0.26	-0.02	0.52
Dominican Republic	4.31	4.99	3.63	2.87	3.73	2	-0.42	-0.36	-0.49
Ecuador	2.63	2.46	2.8	1.72	1.41	2.03	-0.34	-0.42	-0.26
Egypt	17.18	15.66	18.69	14.21	12.83	15.57	0.16	0.24	0.10
El Salvador	3.71	3.14	4.3	1.21	0.76	1.72	-0.64	-0.75	-0.52
Equatorial Guinea	5.53	5.36	5.68	3.71	3.22	4.18	0.38	0.26	0.50
Eritrea	2.14	1.9	2.38	1.94	1.86	2.02	0.41	0.45	0.38
Estonia	15.53	8.66	23.34	14.85	7.4	23.61	0.02	-0.14	0.16
Ethiopia	2.77	2.28	3.25	1.55	1.23	1.87	0.66	0.57	0.73
Federated States of Micronesia	6.13	5.99	6.26	7.11	6.43	7.77	0.30	0.17	0.43
Fiji	3.84	3.6	4.06	8.97	8.25	9.66	0.65	0.49	0.81
Finland	6.86	7.03	6.68	10.93	6.45	15.56	0.64	-0.07	1.45
France	11.53	12.04	10.98	11.44	11.78	11.08	-0.04	-0.07	-0.01
Gabon	5.28	4.3	6.3	4.04	3.7	4.38	0.20	0.28	0.13
Georgia	4.19	3.6	4.84	7	4.37	9.87	0.30	0.03	0.50
Germany	19.56	23.09	15.77	14.85	14.42	15.3	-0.22	-0.34	-0.04
Ghana	2.63	3.3	1.99	3.68	3.89	3.46	1.05	0.85	1.40
Greece	17.87	21.41	14.23	14.58	17.85	11.15	-0.33	-0.34	-0.34
Greenland	7.75	3.89	11.08	8.66	3.03	13.53	0.33	0.03	0.37
Grenada	11.15	14.08	8.14	11.99	12.23	11.75	0.07	-0.14	0.44
Guam	4.3	4.68	3.96	8.19	6.82	9.52	0.00	-0.28	0.30
Guatemala	3.25	2.8	3.69	0.95	0.8	1.11	-0.57	-0.59	-0.55
Guinea	1.7	1.79	1.61	1.31	0.97	1.64	0.28	-0.05	0.62
Guinea-Bissau	2.21	2.02	2.4	1.82	1.45	2.2	0.18	0.01	0.32
Guyana	7.45	8.09	6.8	8.29	7.32	9.25	0.09	-0.08	0.30
Haiti	8.76	11.11	6.34	6.2	7.18	5.2	0.16	0.04	0.38
Honduras	3.29	2.97	3.62	3.07	2.55	3.59	-0.01	-0.17	0.18
Hungary	28.47	27.13	29.92	20.75	17.23	24.63	-0.16	-0.33	0.04
Iceland	3.66	3.38	3.93	2.61	2.07	3.17	-0.17	-0.22	-0.13
India	0.67	0.57	0.76	1.14	1.03	1.24	1.35	1.78	1.07
Indonesia	2.11	1.78	2.43	2.46	2.17	2.75	0.44	0.58	0.35
Iran	2.22	1.83	2.6	3.12	2.18	4.04	0.95	0.90	1.00
Iraq	5.21	4.24	6.16	4.22	3.13	5.29	0.00	0.02	-0.03
Ireland	12.1	12.91	11.28	5.28	4.44	6.13	-0.45	-0.54	-0.34
Israel	5.64	5.94	5.34	2.63	2.65	2.61	-0.50	-0.54	-0.45
Italy	37.33	42.9	31.43	16.93	18.42	15.35	-0.60	-0.65	-0.54
Jamaica	6.84	8.19	5.46	5.69	5.53	5.86	-0.35	-0.44	-0.20
Japan	5.36	6.31	4.39	6.36	7.61	5.05	-0.27	-0.29	-0.27
Jordan	0.92	0.85	0.99	0.46	0.3	0.61	-0.14	-0.30	-0.03
Kazakhstan	5.41	5.36	5.45	9.45	6.96	12.12	0.74	0.41	1.04
Kenya	1.14	1.05	1.23	1.2	0.94	1.46	0.34	0.21	0.44
Kiribati	10.26	8.94	11.59	7.29	5.34	9.28	-0.10	-0.17	-0.03
Kuwait	1.21	0.98	1.4	0.62	0.43	0.77	-0.30	-0.41	-0.24
Kyrgyzstan	3.62	2.2	5.11	5.92	2.66	9.24	1.12	0.79	1.12
Laos	5.56	5.64	5.49	3.16	2.91	3.4	0.44	0.42	0.51

Latvia	19.94	12.3	28.73	31.37	17.6	47.41	0.60	0.41	0.73
Lebanon	4.51	3.72	5.32	2.74	2.4	3.08	-0.10	-0.16	-0.03
Lesotho	6.17	6.2	6.13	10.66	10.93	10.39	-0.02	-0.07	0.02
Liberia	1.75	1.65	1.85	1.02	0.79	1.26	0.36	0.03	0.73
Libya	5.85	5.72	5.97	3.64	3	4.28	-0.47	-0.49	-0.44
Lithuania	6.77	4.3	9.52	15.11	6.99	24.55	0.94	0.40	1.26
Luxembourg	11.72	12.64	10.76	6.5	7.35	5.66	-0.16	-0.16	-0.16
Macedonia	33.84	38.49	29.25	49	57.72	40.2	0.25	0.25	0.23
Madagascar	3.46	2.73	4.18	3.09	2.28	3.89	0.48	0.41	0.51
Malawi	1.75	1.74	1.77	1.38	1.18	1.59	0.53	0.38	0.67
Malaysia	2.7	2.27	3.1	2.62	1.57	3.69	-0.10	-0.24	-0.02
Maldives	1.96	2.28	1.66	1.27	1.19	1.34	0.57	0.62	0.67
Mali	1.95	2.16	1.73	1.05	1.08	1.02	-0.01	-0.08	0.08
Malta	12.69	14.9	10.4	6.07	6.45	5.68	-0.58	-0.64	-0.50
Marshall Islands	5.43	5.15	5.69	7.91	6.87	8.91	0.43	0.25	0.61
Mauritania	1.6	1.46	1.74	0.9	0.78	1.01	0.06	-0.03	0.13
Mauritius	2.22	1.69	2.75	3.91	2.58	5.27	0.40	0.14	0.60
Mexico	1.09	0.95	1.23	1.28	1.06	1.49	0.13	0.01	0.22
Moldova	2.22	1.73	2.76	4.67	2.56	6.94	1.22	0.61	1.59
Mongolia	6.23	6.63	5.82	5.65	3.76	7.57	0.08	-0.21	0.40
Montenegro	21.71	24.47	18.93	31.21	30.08	32.36	0.18	0.15	0.27
Morocco	3.94	4.22	3.65	2.76	2.28	3.25	-0.19	-0.37	0.06
Mozambique	1.77	1.59	1.97	2.14	1.74	2.56	0.86	0.69	0.99
Myanmar	3.78	3.84	3.72	3.96	4.09	3.83	0.54	0.74	0.40
Namibia	6.44	6.38	6.51	5.39	4.48	6.35	-0.05	-0.16	0.06
Nepal	0.49	0.39	0.6	0.64	0.48	0.81	1.31	1.45	1.20
Netherlands	13.98	15.12	12.83	7.52	8.13	6.89	-0.45	-0.47	-0.43
New Zealand	7.37	4.92	9.88	4.62	2.64	6.69	-0.30	-0.43	-0.22
Nicaragua	2.11	1.96	2.27	1.28	1.05	1.51	-0.29	-0.37	-0.20
Niger	1.36	1.27	1.45	0.85	0.62	1.08	0.36	0.09	0.59
Nigeria	1.6	1.38	1.81	0.95	0.55	1.33	0.10	-0.22	0.33
North Korea	1.94	1.72	2.17	2.12	2	2.24	-0.17	-0.09	-0.24
Northern Mariana Islands	2.99	3.12	2.87	3.04	2.02	4.07	0.43	0.14	0.70
Norway	4.33	4.58	4.07	4.83	5.56	4.1	0.49	0.48	0.48
Oman	0.59	0.63	0.56	0.69	0.52	0.77	0.93	0.48	1.29
Pakistan	0.65	0.4	0.88	1.11	0.91	1.31	1.22	2.16	0.84
Palestine	1.71	1.27	2.13	1.2	0.61	1.79	-0.06	-0.26	0.02
Panama	1.72	1.31	2.11	3.39	2.6	4.17	0.68	0.69	0.69
Papua New Guinea	5.44	6.48	4.45	6.37	7.17	5.6	0.39	0.28	0.56
Paraguay	1.79	1.7	1.87	1.95	1.55	2.33	-0.15	-0.24	-0.07
Peru	2.16	2.08	2.25	0.97	0.86	1.09	-0.31	-0.40	-0.21
Philippines	1.49	1.17	1.8	3.1	2.97	3.22	1.21	1.68	0.91
Poland	23.33	23.04	23.64	31.96	33.17	30.67	0.41	0.47	0.36
Portugal	7.2	8.44	5.88	6.57	6.93	6.19	-0.03	-0.12	0.13
Puerto Rico	7.46	8.32	6.54	5.98	6.7	5.21	-0.31	-0.42	-0.22
Qatar	1.35	1.18	1.43	0.57	0.45	0.61	-0.24	-0.23	-0.24
Romania	22.68	22.16	23.23	39.3	35.03	43.84	0.40	0.28	0.53
Russia	20.88	13.72	29.01	42.87	29.12	59.02	0.69	0.87	0.56
Rwanda	2.67	2.56	2.78	1.6	1.38	1.83	0.35	0.24	0.45
Saint Lucia	11.11	12.36	9.81	11.81	10.85	12.8	0.00	-0.20	0.26
Saint Vincent and the Grenadines	3.98	5.06	2.89	5.72	5.17	6.25	0.19	-0.06	0.63
Samoa	4.49	4	4.95	6.4	6.32	6.48	0.36	0.20	0.54
Sao Tome and Principe	2.03	1.65	2.42	1.97	1.55	2.39	0.55	0.45	0.63
Saudi Arabia	2.46	2.69	2.28	2.73	1.94	3.34	0.85	0.45	1.19
Senegal	1.44	1.2	1.69	1.19	0.9	1.49	0.42	0.27	0.54
Serbia	18.58	19.85	17.27	27.34	28.38	26.25	0.14	0.09	0.22
Seychelles	2.07	1.19	2.96	1.91	1.02	2.77	0.02	-0.07	0.07
Sierra Leone	1.72	1.32	2.13	1.38	1.08	1.69	0.30	0.23	0.39
Singapore	3.87	3.22	4.5	2.38	2.12	2.66	-0.46	-0.48	-0.44
Slovakia	5.89	4.35	7.5	7.68	5.25	10.23	0.56	0.29	0.84
Slovenia	78.71	92.31	64.30	44.34	54.01	34.49	-0.44	-0.45	-0.43
Solomon Islands	5.48	5.77	5.21	6.99	6.95	7.03	0.48	0.35	0.63
Somalia	2.95	2.79	3.12	2.04	1.89	2.2	0.01	-0.01	0.05
South Africa	9.38	10.38	8.36	10.61	10.7	10.52	-0.03	-0.09	0.04
South Korea	2.88	3.13	2.63	2.32	2.64	1.99	-0.30	-0.28	-0.33
South Sudan	2.55	2.41	2.69	2.17	1.97	2.36	0.22	0.18	0.29
Spain	13.21	15.86	10.45	11.88	12.31	11.44	-0.08	-0.25	0.18
Sri Lanka	6.58	3.85	9.25	2.21	1.6	2.86	-0.64	-0.60	-0.65
Sudan	5.69	5.81	5.57	3.79	3.51	4.06	0.19	0.12	0.28
Suriname	5.56	6.97	4.19	6.03	6.35	5.72	0.00	-0.13	0.22

Swaziland	6.43	6.57	6.29	9.69	9.45	9.94	-0.05	-0.13	0.04
Sweden	9.23	8.53	9.94	7.52	7.13	7.91	-0.04	-0.08	0.00
Switzerland	8.38	8.06	8.73	6.1	5.79	6.42	-0.15	-0.20	-0.09
Syria	5.91	5.09	6.71	5.07	4.16	5.96	-0.34	-0.17	-0.43
Taiwan	2.47	1.91	3	2.28	1.68	2.89	-0.30	-0.34	-0.27
Tajikistan	2.51	2.16	2.86	2.2	1.44	2.95	0.64	0.35	0.82
Tanzania	1.91	1.72	2.11	1.75	1.43	2.08	0.54	0.45	0.60
Thailand	8.15	6.72	9.6	1.98	1.35	2.63	-0.82	-0.86	-0.76
The Bahamas	7.34	6.57	8.12	9.53	7.37	11.77	0.13	-0.09	0.33
The Gambia	1.28	1.18	1.38	0.86	0.54	1.18	0.21	-0.06	0.43
Timor-Leste	3.31	3.47	3.14	2.02	2.09	1.95	0.36	0.35	0.42
Togo	1.65	1.47	1.83	1.28	0.79	1.78	0.07	-0.17	0.22
Tonga	1.24	0.53	1.94	1.84	0.71	2.96	0.35	0.26	0.38
Trinidad and Tobago	3.59	4.2	2.97	6.48	5.07	7.93	0.49	0.05	1.10
Tunisia	3.42	3.7	3.13	2.79	2.21	3.38	-0.17	-0.36	0.05
Turkey	2.82	2.01	3.64	1.4	0.89	1.93	-0.12	-0.19	-0.07
Turkmenistan	3.14	2.96	3.33	2.59	1.87	3.33	0.16	-0.04	0.32
Uganda	2.02	1.89	2.15	1.88	1.4	2.36	0.79	0.51	1.01
Ukraine	9.82	6.19	14.05	17.15	9.21	26.54	0.42	0.27	0.46
United Arab Emirates	3.65	2.76	4.11	3.44	1.8	4.04	0.25	0.24	0.21
United Kingdom	10.65	12.61	8.58	5.89	6.31	5.46	-0.33	-0.40	-0.22
United States	10.92	8.51	13.45	8.86	6.97	10.83	-0.16	-0.18	-0.14
Uruguay	14.05	11.64	16.62	10.55	8.12	13.16	-0.32	-0.44	-0.19
Uzbekistan	0.72	0.68	0.77	0.94	0.76	1.12	0.52	0.41	0.61
Vanuatu	6.02	5.87	6.16	7.51	7.28	7.73	0.36	0.27	0.44
Venezuela	2.34	1.98	2.7	2.37	1.95	2.79	-0.18	-0.15	-0.19
Vietnam	2.13	1.76	2.52	2.91	2.62	3.2	0.44	0.62	0.33
Virgin Islands, U.S.	7.97	6.34	9.69	19.44	13.65	25.79	0.14	0.01	0.24
Yemen	5.65	6.28	5.01	3.4	3.41	3.39	0.20	0.12	0.34
Zambia	1.79	1.9	1.68	2.25	1.69	2.81	0.78	0.38	1.19
Zimbabwe	3.34	3.38	3.29	4.98	4.03	5.95	0.23	0.00	0.47
**Cerebrovascular disease**									
Afghanistan	81.24	112.60	51.38	102.23	138.75	67.93	0.77	0.80	0.81
Albania	83.30	82.86	83.73	161.76	159.30	164.27	0.41	0.41	0.41
Algeria	43.90	47.98	39.93	60.25	65.40	55.17	0.49	0.47	0.51
American Samoa	52.63	41.65	63.02	34.28	36.33	32.24	-0.18	-0.13	-0.24
Andorra	69.19	73.99	64.13	68.95	69.19	68.7	-0.18	-0.19	-0.15
Angola	65.63	75.72	55.29	49.92	61.23	38.47	0.57	0.58	0.52
Antigua and Barbuda	105.07	117.01	92.30	67.01	69.49	64.31	-0.29	-0.31	-0.25
Argentina	85.96	83.68	88.34	58.78	62.17	55.24	-0.33	-0.35	-0.32
Armenia	85.93	98.15	72.99	123.95	130.21	116.74	0.09	0.01	0.20
Australia	74.46	87.59	61.27	61.3	71.75	50.83	-0.14	-0.17	-0.10
Austria	146.50	177.48	112.73	70.28	84.38	55.49	-0.46	-0.46	-0.45
Azerbaijan	81.74	93.60	69.36	82.53	83.57	81.48	0.24	0.16	0.38
Bahrain	18.82	21.76	16.65	10.07	12.41	8.64	-0.08	-0.06	-0.08
Bangladesh	57.59	66.23	49.34	97.83	98.19	97.48	1.89	1.84	2.04
Barbados	115.48	128.25	101.72	108.67	121.09	95.17	-0.23	-0.25	-0.21
Belarus	174.00	209.47	133.83	217.72	242.08	189.47	-0.09	-0.10	-0.05
Belgium	119.78	142.78	95.71	83.83	96.33	70.85	-0.27	-0.30	-0.22
Belize	43.99	45.9	42.11	35.06	31.46	38.67	-0.12	-0.14	-0.09
Benin	41.54	46.61	36.15	47.46	51.44	43.47	0.91	0.86	0.95
Bermuda	63.38	63.07	63.69	49.82	49	50.67	-0.07	-0.13	-0.03
Bhutan	34.07	36.55	31.77	37.98	34.48	40.99	0.99	0.97	1.04
Bolivia	48.89	57.00	40.83	51.57	59.61	43.57	0.60	0.53	0.68
Bosnia and Herzegovina	98.66	111.44	85.87	146.41	157.43	135.28	0.14	0.03	0.26
Botswana	39.36	49.27	29.22	54.92	63.03	46.81	-0.06	-0.09	0.03
Brazil	67.35	64.30	70.45	69.32	66.83	71.89	-0.05	-0.07	-0.03
Brunei	31.46	32.64	30.41	31.24	31.52	30.97	-0.11	-0.13	-0.09
Bulgaria	278.39	286.23	270.34	303.52	325.70	280.23	-0.11	-0.12	-0.12
Burkina Faso	26.61	31.52	21.47	23.95	29.81	18.03	0.64	0.66	0.57
Burundi	105.64	124.16	86.48	41.63	47.34	35.8	-0.09	-0.09	-0.05
Cambodia	70.34	78.78	61.30	70.01	72.70	67.18	0.90	0.87	1.01
Cameroon	49.30	55.58	42.94	51.44	56.49	46.39	0.27	0.25	0.27
Canada	53.6	60.21	46.88	49.57	56.32	42.72	-0.16	-0.21	-0.11
Cape Verde	75.67	73.49	78.07	72.39	73.45	71.31	0.22	0.28	0.17
Central African Republic	111.35	123.79	98.48	109.29	120.08	98.21	0.10	0.05	0.13
Chad	38.93	46.52	31.23	41.00	46.76	35.25	0.48	0.40	0.56
Chile	70.30	70.80	69.80	70.66	75.82	65.36	-0.08	-0.12	-0.06
China	115.11	111.46	118.58	136.98	112.89	159.68	0.34	0.34	0.36
Colombia	35.41	37.79	33.01	33.67	36.05	31.21	0.04	-0.04	0.10

Comoros	65.93	73.48	58.42	37.31	42.7	32.02	0.19	0.24	0.18
Congo	94.91	111.97	77.76	70.10	97.30	42.94	0.08	0.07	-0.04
Costa Rica	26.86	28.17	25.58	30.27	32.03	28.51	-0.09	-0.11	-0.09
Cote d'Ivoire	47.48	46.39	48.47	51.40	49.90	52.85	0.41	0.43	0.37
Croatia	192.30	213.19	170.09	189.40	218.82	157.83	-0.14	-0.16	-0.13
Cuba	71.58	73.90	69.30	100.52	105.23	95.87	0.03	0.00	0.06
Cyprus	102.38	97.53	107.35	48.73	44.27	53.5	-0.37	-0.36	-0.36
Czech Republic	225.49	260.49	188.39	122.92	146.35	98.73	-0.35	-0.35	-0.35
Democratic Republic of the Congo	50.31	65.49	34.75	50.17	59.21	41.09	0.54	0.43	0.82
Denmark	124.66	140.74	108.11	79.79	87.85	71.60	-0.20	-0.23	-0.16
Djibouti	44.28	42.31	46.22	57.18	54.57	59.76	0.44	0.51	0.42
Dominica	57.11	65.12	49.03	75.91	78.44	73.33	0.00	0.03	0.02
Dominican Republic	36.5	35.21	37.78	67.26	64.87	69.67	0.59	0.58	0.60
Ecuador	33.42	32.56	34.26	37.11	36.58	37.64	0.11	0.13	0.09
Egypt	77.02	77.31	76.74	61.25	59.56	62.91	0.11	0.17	0.07
El Salvador	38.75	38.28	39.24	29.42	29.97	28.79	-0.19	-0.20	-0.20
Equatorial Guinea	118.43	140.02	97.78	44.30	50.35	38.54	-0.22	-0.24	-0.19
Eritrea	53.52	64.44	42.47	50.57	65.33	35.86	0.46	0.47	0.37
Estonia	230.08	279.30	174.08	126.09	152.50	95.08	-0.41	-0.45	-0.38
Ethiopia	70.84	75.38	66.28	41.07	45.18	36.94	0.69	0.70	0.65
Federated States of Micronesia	76.16	69.17	82.85	70.41	66.63	74.01	0.04	0.05	0.03
Fiji	68.42	70.94	65.97	73.16	81.61	64.98	-0.25	-0.26	-0.27
Finland	122.32	147.36	95.75	87.66	103.31	71.53	-0.26	-0.29	-0.22
France	95.61	108.87	81.64	73.08	83.12	62.39	-0.26	-0.28	-0.25
Gabon	103.88	107.42	100.19	70.59	84.02	57.47	0.07	0.16	-0.06
Georgia	204.50	223.96	183.05	257.71	252.06	263.88	-0.02	-0.06	0.04
Germany	142.47	173.96	108.63	90.71	105.57	75.35	-0.35	-0.36	-0.31
Ghana	51.67	79.96	24.26	57.55	78.46	36.44	0.65	0.55	1.06
Greece	189.94	220.85	158.24	177.17	206.39	146.50	-0.23	-0.26	-0.22
Greenland	53.24	57.88	49.22	46.26	38.72	52.79	0.04	-0.12	0.21
Grenada	118.88	137.52	99.75	95.05	110.98	79.21	-0.21	-0.21	-0.21
Guam	41.59	40.27	42.75	75.60	76.85	74.40	-0.05	-0.06	-0.06
Guatemala	19.43	20.29	18.55	24.82	27.24	22.3	0.89	0.95	0.80
Guinea	46.39	59.86	32.62	52.42	60.96	43.92	0.87	0.76	1.12
Guinea-Bissau	60.02	70.79	48.99	63.34	73.82	52.74	0.51	0.49	0.55
Guyana	109.92	103.55	116.50	94.92	89.21	100.59	-0.16	-0.12	-0.18
Haiti	107.47	143.45	70.43	91.07	118.23	63.32	0.37	0.31	0.49
Honduras	58.31	58.92	57.70	56.14	58.62	53.66	0.00	-0.04	0.04
Hungary	224.08	244.23	202.29	138.72	157.83	117.66	-0.28	-0.32	-0.27
Iceland	67.45	75.04	59.93	46.67	46.65	46.68	-0.20	-0.22	-0.16
India	57.00	59.28	54.88	61.15	57.62	64.42	0.47	0.48	0.48
Indonesia	75.38	83.08	67.74	110.02	115.49	104.62	0.79	0.79	0.82
Iran	44.43	38.63	50.02	54.64	45.94	63.21	0.69	0.87	0.60
Iraq	66.89	73.71	60.19	52.67	55.54	49.86	-0.04	0.02	-0.07
Ireland	74.11	87.34	60.77	44.33	49.14	39.37	-0.24	-0.25	-0.22
Israel	59.86	63.91	55.81	41.85	45.47	38.16	-0.25	-0.27	-0.23
Italy	131.71	145.60	116.98	119.83	138.84	99.63	-0.20	-0.22	-0.19
Jamaica	103.77	121.98	85.11	117.19	131.05	103.23	-0.12	-0.12	-0.10
Japan	107.82	112.27	103.21	133.14	139.25	126.70	-0.24	-0.27	-0.22
Jordan	44.2	49.1	39.76	24.91	24.31	25.49	-0.04	-0.04	-0.02
Kazakhstan	126.73	154.25	97.49	121.62	127.86	114.93	-0.05	-0.10	0.08
Kenya	28.22	32.92	23.49	27.2	28.87	25.52	0.24	0.19	0.33
Kiribati	111.20	85.55	137.39	119.46	81.63	158.26	0.35	0.32	0.34
Kuwait	8.7	8.93	8.49	11.68	9.46	13.41	0.82	0.44	1.18
Kyrgyzstan	129.98	152.77	106.21	98.00	93.03	103.08	-0.02	-0.11	0.15
Laos	91.82	106.16	77.46	62.41	65.30	59.48	0.66	0.61	0.77
Latvia	281.11	342.20	210.74	235.28	287.16	174.86	-0.15	-0.17	-0.13
Lebanon	53.16	74.26	31.48	39.84	58.18	21.69	0.11	0.01	0.14
Lesotho	55.02	60.13	49.60	86.61	102.17	70.73	-0.10	-0.10	-0.14
Liberia	41.70	48.47	34.89	39.09	47.22	31.09	1.17	1.07	1.24
Libya	30.16	33.13	27.49	41.07	43.43	38.74	0.13	0.21	0.07
Lithuania	128.87	151.66	103.51	159.74	187.54	127.41	0.08	0.06	0.08
Luxembourg	155.90	182.71	127.98	58.58	69.27	47.84	-0.43	-0.46	-0.40
Macedonia	169.39	171.75	167.06	211.71	222.19	201.14	0.07	0.06	0.06
Madagascar	77.00	81.84	72.14	69.32	74.07	64.55	0.49	0.51	0.45
Malawi	32.77	41.93	23.3	27.42	30.83	24	0.61	0.46	0.92
Malaysia	51.26	51.97	50.57	51.25	42.69	59.98	-0.08	-0.10	-0.03
Maldives	30.42	24.87	35.70	25.23	16.72	33.69	0.94	0.95	0.88
Mali	56.20	73.44	38.59	36.46	48.56	24.6	0.18	0.19	0.16
Malta	72.72	74.42	70.96	64.99	70.82	59.12	-0.22	-0.21	-0.24

Marshall Islands	61.70	52.65	70.36	64.29	58.43	69.95	0.03	0.03	0.02
Mauritania	43.63	54.22	32.98	33.2	43.58	22.97	0.42	0.44	0.34
Mauritius	89.88	81.61	98.19	80.29	76.40	84.27	-0.29	-0.30	-0.28
Mexico	25.37	26.78	23.95	31.52	33.84	29.18	0.19	0.14	0.23
Moldova	151.01	168.59	131.72	137.05	141.71	132.02	-0.04	-0.09	0.03
Mongolia	66.63	68.82	64.39	124.11	114.37	134.04	1.21	1.29	1.23
Montenegro	195.18	240.12	149.85	235.06	259.01	210.54	-0.02	-0.02	0.03
Morocco	44.20	49.86	38.52	55.21	63.03	47.21	0.42	0.41	0.41
Mozambique	49.57	49.64	49.49	56.42	55.79	57.09	0.74	0.68	0.76
Myanmar	94.45	95.69	93.15	100.27	89.14	111.91	0.53	0.47	0.61
Namibia	51.00	59.08	42.57	38.41	39.52	37.23	-0.14	-0.19	-0.05
Nepal	38.10	40.19	35.98	45.98	41.63	50.60	1.12	1.03	1.23
Netherlands	85.77	99.29	71.95	64.68	74.26	54.95	-0.23	-0.27	-0.19
New Zealand	76.89	89.99	63.38	61.86	73.94	49.32	-0.10	-0.13	-0.10
Nicaragua	21.73	22.56	20.89	30.11	31.51	28.66	0.62	0.64	0.61
Niger	32.63	36.31	28.83	34.16	37.71	30.66	1.24	1.24	1.25
Nigeria	37.04	43.40	30.81	22.52	22.84	22.21	0.13	0.03	0.30
North Korea	91.65	109.05	73.22	169.74	184.95	153.84	0.38	0.30	0.52
Northern Mariana Islands	36.86	37.73	36.08	19.73	14.31	25.18	-0.25	-0.33	-0.17
Norway	131.56	148.69	114.05	67.97	81.11	54.98	-0.31	-0.34	-0.29
Oman	16.57	20.88	13.13	16.95	20.63	15.08	0.65	0.68	0.83
Pakistan	56.42	51.10	61.35	64.53	55.73	72.85	0.47	0.50	0.44
Palestine	30.48	33.77	27.27	32.27	30.84	33.66	0.39	0.37	0.48
Panama	45.19	43.91	46.44	49.84	46.91	52.76	-0.06	-0.09	-0.03
Papua New Guinea	86.48	99.02	74.51	84.21	102.01	67.10	0.16	0.19	0.11
Paraguay	48.17	52.42	44.02	61.66	62.37	60.97	-0.01	-0.01	0.01
Peru	37.52	37.39	37.65	23.07	24.28	21.86	-0.06	-0.06	-0.07
Philippines	38.02	32.27	43.65	64.89	57.56	72.08	0.81	0.87	0.76
Poland	120.25	144.96	94.27	113.78	129.54	96.82	-0.02	-0.09	0.07
Portugal	257.93	276.97	237.45	144.24	155.24	132.51	-0.40	-0.40	-0.40
Puerto Rico	48.4	47.2	49.67	60.24	67.23	52.69	0.06	0.03	0.04
Qatar	15.88	20.34	13.67	7.53	8.87	7.03	-0.15	-0.14	-0.10
Romania	198.76	216.67	180.35	277.94	297.36	257.27	0.13	0.11	0.16
Russia	240.16	301.25	170.79	257.22	293.78	214.28	-0.12	-0.14	-0.04
Rwanda	62.58	79.37	45.19	34.03	39.4	28.19	0.22	0.13	0.37
Saint Lucia	104.56	117.92	90.80	106.31	124.48	87.45	-0.05	-0.04	-0.07
Saint Vincent and the Grenadines	77.20	95.31	58.91	88.78	97.44	80.27	-0.04	-0.06	0.03
Samoa	59.06	50.77	66.70	57.58	62.50	52.96	-0.08	-0.08	-0.07
Sao Tome and Principe	63.06	73.50	52.42	60.25	73.11	47.30	0.52	0.53	0.48
Saudi Arabia	23.22	25.09	21.76	22.79	21.86	23.5	0.63	0.72	0.61
Senegal	39.65	44.13	35.11	47.12	55.95	37.97	1.03	1.10	0.87
Serbia	202.91	228.50	176.65	246.85	277.78	214.41	-0.06	-0.09	-0.04
Seychelles	78.56	79.89	77.21	51.36	50.53	52.17	-0.28	-0.32	-0.23
Sierra Leone	37.60	33.20	42.10	45.50	52.87	37.98	0.94	1.38	0.57
Singapore	59.93	66.02	54.00	51.9	60.17	43.33	-0.25	-0.28	-0.24
Slovakia	131.06	143.19	118.35	97.22	107.70	86.17	-0.14	-0.20	-0.09
Slovenia	136.89	152.90	119.91	103.92	120.48	87.06	-0.24	-0.26	-0.23
Solomon Islands	92.54	90.18	94.78	87.76	90.95	84.67	0.10	0.12	0.06
Somalia	77.36	93.05	61.38	56.79	68.47	44.99	0.12	0.12	0.12
South Africa	66.55	88.07	44.53	68.84	84.32	52.81	-0.12	-0.15	-0.02
South Korea	113.90	126.39	101.46	87.72	95.93	79.42	-0.33	-0.35	-0.31
South Sudan	52.94	62.61	43.17	50.24	59.46	41.05	0.44	0.44	0.46
Spain	118.52	134.39	101.98	74.46	86.66	61.96	-0.36	-0.38	-0.35
Sri Lanka	28.97	23.96	33.89	65.89	60.94	71.21	1.43	1.43	1.36
Sudan	66.89	77.08	56.77	60.13	66.92	53.40	0.58	0.57	0.61
Suriname	68.93	75.21	62.86	96.18	101.67	90.70	0.28	0.29	0.28
Swaziland	44.63	51.26	37.23	65.25	75.55	54.74	-0.07	-0.10	-0.04
Sweden	123.09	140.11	105.66	89	104.59	73.39	-0.15	-0.18	-0.13
Switzerland	101.96	115.85	87.41	55.37	64.58	45.97	-0.36	-0.38	-0.35
Syria	64.39	59.87	68.77	47.96	44.99	50.85	-0.43	-0.24	-0.53
Taiwan	74.61	68.48	80.29	65.59	57.78	73.42	-0.34	-0.37	-0.32
Tajikistan	77.25	85.24	69.17	56.14	56.45	55.83	0.35	0.32	0.41
Tanzania	37.87	44.03	31.6	34.14	36.61	31.65	0.50	0.40	0.63
Thailand	60.50	61.64	59.34	78.83	67.39	90.59	-0.17	-0.26	-0.07
The Bahamas	55.94	60.67	51.15	67.57	74.26	60.58	0.05	-0.01	0.09
The Gambia	33.1	39.36	26.93	29.57	30.58	28.54	0.63	0.61	0.79
Timor-Leste	42.77	50.13	35.60	49.34	59.28	39.72	1.42	1.45	1.39
Togo	44.27	52.35	35.98	43.45	43.48	43.41	0.35	0.27	0.50
Tonga	51.83	62.95	40.97	59.93	70.91	49.02	0.05	0.04	0.07
Trinidad and Tobago	82.81	85.56	80.05	85.10	85.50	84.69	-0.15	-0.13	-0.17

Tunisia	55.03	48.57	61.40	66.12	60.75	71.63	0.20	0.31	0.12
Turkey	43.74	51.13	36.17	41.69	43.66	39.64	0.66	0.51	0.90
Turkmenistan	75.13	81.73	68.34	84.01	79.76	88.41	0.57	0.48	0.70
Uganda	50.96	63.26	38.51	36.7	38.04	35.36	0.39	0.21	0.70
Ukraine	237.75	288.65	178.51	216.94	244.64	184.22	-0.26	-0.28	-0.20
United Arab Emirates	28.55	32.58	26.46	26.16	24.84	26.64	0.18	0.36	0.22
United Kingdom	130.61	156.79	102.92	82.51	95.99	68.68	-0.23	-0.27	-0.18
United States	60.76	70.44	50.58	50.84	57.75	43.69	-0.14	-0.18	-0.08
Uruguay	128.30	139.34	116.58	122.05	149.39	92.76	-0.14	-0.15	-0.19
Uzbekistan	79.10	88.34	69.66	78.17	78.44	77.89	0.15	0.11	0.23
Vanuatu	102.25	94.04	110.00	96.49	98.99	94.05	0.02	0.07	-0.03
Venezuela	35.61	37.53	33.72	43.08	44.37	41.78	-0.02	0.02	-0.04
Vietnam	96.47	99.76	93.09	130.02	131.09	128.92	0.41	0.40	0.43
Virgin Islands, U.S.	44.12	43.12	45.17	93.83	88.76	99.40	-0.01	-0.03	0.02
Yemen	60.31	73.96	46.41	58.33	69.25	47.64	0.85	0.85	0.91
Zambia	35.45	47.82	22.86	40.69	42.21	39.17	0.64	0.37	1.30
Zimbabwe	27.54	34.15	20.87	33.00	34.32	31.65	-0.02	-0.16	0.22
**Endocarditis**									
Afghanistan	1.01	1.51	0.53	1.34	1.96	0.75	0.87	0.88	1.03
Albania	0.56	0.52	0.6	0.82	0.67	0.97	0.06	-0.06	0.16
Algeria	1.82	2.18	1.48	2.31	2.82	1.8	0.39	0.42	0.38
American Samoa	1.74	1.61	1.86	2.12	2.52	1.72	0.57	0.65	0.38
Andorra	1.21	1.45	0.95	1.74	2	1.47	0.21	0.23	0.25
Angola	1.61	1.83	1.38	1.44	1.63	1.25	0.83	0.76	1.04
Antigua and Barbuda	2.04	2.17	1.91	1.6	1.45	1.77	-0.12	-0.22	0.00
Argentina	3.12	4.55	1.64	3.7	5.46	1.86	0.16	0.06	0.23
Armenia	0.22	0.21	0.22	0.33	0.3	0.37	0.17	0.09	0.25
Australia	1	1.05	0.95	1.6	1.66	1.54	0.68	0.60	0.76
Austria	2.79	3.73	1.76	1.62	2.17	1.04	-0.34	-0.34	-0.34
Azerbaijan	0.58	0.52	0.65	0.7	0.52	0.88	0.47	0.31	0.59
Bahrain	0.94	1.1	0.82	0.62	0.76	0.53	0.13	0.16	0.17
Bangladesh	0.13	0.15	0.12	0.24	0.26	0.22	2.08	2.43	1.92
Barbados	2.96	3.4	2.49	4.01	4.43	3.55	0.12	0.05	0.21
Belarus	0.61	0.52	0.71	0.99	0.7	1.32	0.19	0.07	0.27
Belgium	2.78	3.87	1.63	5.04	6.63	3.38	0.91	0.79	1.19
Belize	0.98	1.35	0.61	0.78	0.96	0.61	-0.09	-0.08	0.01
Benin	1.54	1.69	1.38	1.17	1.22	1.11	0.31	0.28	0.39
Bermuda	1.6	1.34	1.86	1.35	0.99	1.71	0.00	-0.17	0.15
Bhutan	0.17	0.19	0.16	0.34	0.35	0.34	2.57	2.87	2.54
Bolivia	2.21	2.05	2.37	2.29	2.24	2.35	0.60	0.64	0.63
Bosnia and Herzegovina	0.33	0.27	0.38	0.5	0.38	0.62	0.19	0.05	0.33
Botswana	0.78	0.91	0.65	0.91	0.9	0.93	-0.20	-0.27	-0.08
Brazil	0.74	0.67	0.81	1.23	1.12	1.35	0.56	0.52	0.59
Brunei	0.99	0.71	1.24	1.27	0.87	1.66	0.17	0.11	0.22
Bulgaria	1.52	1.24	1.81	1.42	1.13	1.73	-0.24	-0.30	-0.19
Burkina Faso	1.61	1.84	1.38	1.4	1.56	1.23	0.62	0.55	0.74
Burundi	2.16	2.31	2	1.16	1.25	1.07	0.26	0.36	0.30
Cambodia	1.59	1.77	1.4	1.34	1.41	1.27	0.69	0.75	0.90
Cameroon	1.81	1.98	1.63	1.37	1.35	1.38	-0.07	-0.15	0.03
Canada	1.07	1.19	0.96	1.73	1.83	1.63	0.47	0.31	0.65
Cape Verde	1.33	1.45	1.2	1.27	1.39	1.15	0.24	0.26	0.28
Central African Republic	1.82	1.99	1.64	1.93	1.95	1.91	0.20	0.09	0.33
Chad	1.37	1.6	1.13	1.19	1.27	1.11	0.27	0.18	0.42
Chile	0.92	1.16	0.67	1.4	1.7	1.08	0.39	0.21	0.64
China	0.36	0.32	0.39	0.46	0.39	0.52	0.46	0.62	0.37
Colombia	0.67	0.63	0.71	0.94	0.81	1.08	0.54	0.30	0.77
Comoros	1.91	1.99	1.84	1.25	1.35	1.15	0.36	0.43	0.35
Congo	1.85	2.07	1.63	1.69	2.09	1.28	0.35	0.27	0.41
Costa Rica	0.69	0.57	0.8	1.16	1.07	1.24	0.36	0.49	0.27
Cote d'Ivoire	1.53	1.51	1.55	1.25	1.14	1.35	0.08	0.03	0.14
Croatia	0.74	0.71	0.78	0.78	0.73	0.83	-0.08	-0.15	0.00
Cuba	0.81	1.04	0.59	1.23	1.56	0.9	0.11	0.05	0.17
Cyprus	2.68	3.16	2.18	1.99	2.18	1.78	0.00	-0.01	0.06
Czech Republic	0.86	0.79	0.93	1.41	1.3	1.52	0.97	0.90	1.04
Democratic Republic of the Congo	1.11	1.38	0.84	1.12	1.32	0.92	0.56	0.51	0.71
Denmark	1.34	1.71	0.95	1.87	2.02	1.71	0.75	0.47	1.28
Djibouti	1.78	1.73	1.83	1.95	1.73	2.17	0.22	0.16	0.29
Dominica	1.64	2.18	1.11	2.34	2.85	1.82	0.08	0.13	0.13
Dominican Republic	1.05	1.36	0.75	1.07	1.33	0.82	-0.11	-0.14	-0.05
Ecuador	0.87	0.8	0.94	0.9	0.77	1.03	0.04	-0.03	0.11

Egypt	3.83	5.83	1.84	3.41	5.16	1.7	0.25	0.35	0.24
El Salvador	1.09	1.1	1.07	0.98	0.9	1.08	-0.03	-0.15	0.12
Equatorial Guinea	2.35	2.75	1.98	1.56	1.56	1.56	0.38	0.21	0.69
Eritrea	1.5	1.63	1.36	1.43	1.63	1.23	0.51	0.50	0.51
Estonia	1.04	0.8	1.32	1.34	1.23	1.47	0.38	0.56	0.27
Ethiopia	1.64	1.74	1.55	1.14	1.17	1.11	1.03	1.02	1.18
Federated States of Micronesia	2.63	3.28	2.01	3.22	4.04	2.44	0.40	0.38	0.42
Fiji	4.79	5.84	3.78	7.3	8.58	6.06	0.07	-0.04	0.20
Finland	0.85	0.93	0.76	0.84	0.85	0.83	0.02	-0.07	0.15
France	2.43	3.16	1.66	4.35	5.01	3.63	0.73	0.51	1.16
Gabon	2.24	2.37	2.1	1.93	2.17	1.69	0.36	0.38	0.34
Georgia	0.58	0.47	0.7	1.02	0.78	1.29	0.39	0.44	0.34
Germany	3.1	4.42	1.68	3.55	4.53	2.54	0.18	0.09	0.51
Ghana	1.55	2.07	1.05	1.56	1.68	1.45	0.49	0.30	0.91
Greece	1.87	3.04	0.66	2.75	4.2	1.23	0.21	0.10	0.58
Greenland	0.97	0.69	1.21	0.97	0.64	1.25	0.22	0.25	0.21
Grenada	3.41	4.49	2.3	3.7	4.82	2.58	0.09	0.08	0.14
Guam	1.02	1.06	0.99	3.34	3.51	3.19	0.88	0.85	0.90
Guatemala	0.91	1.02	0.79	0.69	0.65	0.73	0.14	-0.03	0.43
Guinea	1.62	1.94	1.3	1.11	1.21	1.01	0.16	0.11	0.28
Guinea-Bissau	1.85	2.12	1.57	1.57	1.71	1.43	0.22	0.16	0.32
Guyana	2.13	2.92	1.31	2.28	2.67	1.88	0.05	-0.06	0.41
Haiti	1.86	2.38	1.34	1.57	2.06	1.07	0.40	0.42	0.44
Honduras	0.85	0.93	0.76	0.9	0.87	0.93	0.12	-0.09	0.43
Hungary	1.44	1.44	1.43	1.85	1.9	1.79	0.49	0.40	0.58
Iceland	0.71	0.84	0.59	0.95	0.93	0.98	0.55	0.41	0.79
India	0.18	0.19	0.18	0.39	0.36	0.41	1.93	2.05	1.91
Indonesia	0.87	0.84	0.9	1.22	1.19	1.25	0.75	0.84	0.71
Iran	0.4	0.34	0.46	0.58	0.43	0.72	1.01	1.02	1.08
Iraq	2.91	4.77	1.08	2.45	3.92	1.01	0.04	0.13	0.08
Ireland	1.32	1.73	0.91	1.16	1.17	1.16	0.12	-0.09	0.54
Israel	0.88	1.02	0.73	1.38	1.41	1.35	0.70	0.42	1.09
Italy	0.97	1.16	0.77	2.99	3.73	2.21	1.73	1.65	1.74
Jamaica	1.49	1.9	1.07	2.06	2.47	1.65	0.08	0.08	0.14
Japan	1.66	2.03	1.28	2.67	3.34	1.97	-0.01	-0.03	-0.02
Jordan	1.18	1.72	0.69	0.78	0.97	0.59	0.14	0.13	0.36
Kazakhstan	0.67	0.74	0.59	0.76	0.69	0.84	0.14	0.04	0.32
Kenya	0.81	0.89	0.72	0.87	0.89	0.85	0.40	0.37	0.47
Kiribati	3.9	4.39	3.4	4.45	4.86	4.03	0.44	0.55	0.38
Kuwait	0.42	0.66	0.22	0.41	0.55	0.3	0.36	0.19	0.97
Kyrgyzstan	0.29	0.25	0.33	0.24	0.18	0.31	0.11	0.08	0.12
Laos	2.08	2.23	1.94	1.6	1.6	1.6	1.00	1.14	1.21
Latvia	0.82	0.61	1.05	0.86	0.74	1.01	0.08	0.20	0.01
Lebanon	1.45	1.76	1.13	1.3	1.68	0.92	0.34	0.24	0.39
Lesotho	0.8	0.94	0.65	1.14	1.2	1.09	-0.15	-0.27	0.01
Liberia	1.6	1.72	1.47	0.95	1.05	0.86	0.41	0.35	0.54
Libya	1.58	1.87	1.33	1.47	1.78	1.16	-0.21	-0.10	-0.28
Lithuania	0.77	0.61	0.96	1.16	0.97	1.39	0.31	0.38	0.27
Luxembourg	2.53	3.43	1.59	3.08	4.12	2.05	0.85	0.73	1.06
Macedonia	0.4	0.41	0.4	0.46	0.42	0.49	-0.04	-0.16	0.09
Madagascar	2.37	2.35	2.4	2.21	2.08	2.35	0.56	0.50	0.60
Malawi	1.17	1.35	0.99	1.01	1.07	0.95	0.67	0.63	0.83
Malaysia	1.59	1.17	1.99	2.61	1.7	3.53	0.52	0.60	0.46
Maldives	1.12	1.26	0.99	0.63	0.62	0.65	0.41	0.64	0.50
Mali	1.81	2.31	1.3	1.15	1.46	0.86	0.18	0.17	0.25
Malta	1.44	1.84	1.03	2.43	2.81	2.05	0.47	0.27	0.81
Marshall Islands	2.2	2.74	1.68	2.93	3.59	2.28	0.35	0.27	0.44
Mauritania	1.39	1.63	1.15	0.99	1.16	0.81	0.36	0.33	0.41
Mauritius	0.71	0.82	0.61	1.23	1.08	1.39	0.38	-0.01	0.92
Mexico	0.46	0.48	0.43	0.58	0.55	0.61	0.22	0.04	0.41
Moldova	0.33	0.27	0.39	0.56	0.39	0.74	0.79	0.55	0.96
Mongolia	0.24	0.25	0.23	0.27	0.23	0.31	0.36	0.32	0.45
Montenegro	0.48	0.49	0.47	0.65	0.59	0.71	0.12	0.13	0.13
Morocco	2.86	3.6	2.12	3.66	4.74	2.55	0.45	0.48	0.41
Mozambique	1.17	1.33	0.99	1.27	1.26	1.28	0.72	0.49	1.10
Myanmar	1.55	1.72	1.37	2	2.24	1.76	0.91	1.11	0.87
Namibia	0.82	0.9	0.73	0.62	0.57	0.67	-0.11	-0.20	0.00
Nepal	0.14	0.13	0.14	0.23	0.2	0.27	2.01	1.93	2.16
Netherlands	2.03	2.67	1.38	5.21	6.25	4.15	1.61	1.30	2.17
New Zealand	0.91	0.91	0.9	0.69	0.64	0.73	-0.16	-0.25	-0.06

Nicaragua	0.51	0.53	0.49	0.54	0.52	0.55	0.24	0.17	0.35
Niger	1.27	1.46	1.07	0.81	0.92	0.7	0.42	0.45	0.48
Nigeria	1.27	1.35	1.19	0.74	0.67	0.82	0.09	-0.02	0.26
North Korea	0.45	0.43	0.48	0.59	0.63	0.55	-0.01	0.16	-0.15
Northern Mariana Islands	0.92	1.54	0.36	0.8	1.13	0.46	0.23	0.30	0.54
Norway	1.72	2.24	1.19	2.12	2.63	1.61	0.64	0.43	0.98
Oman	1.49	1.59	1.42	1.3	1.38	1.26	0.47	0.53	0.57
Pakistan	0.2	0.15	0.25	0.38	0.31	0.45	1.44	1.88	1.23
Palestine	1.03	1.36	0.7	0.94	1.22	0.67	0.21	0.35	0.18
Panama	0.63	0.54	0.72	1.06	0.95	1.17	0.43	0.49	0.39
Papua New Guinea	1.93	2.7	1.19	2.43	3.41	1.48	0.52	0.48	0.57
Paraguay	0.75	0.79	0.72	1.12	1.06	1.19	0.17	0.12	0.23
Peru	1.15	1.19	1.12	1.04	1.11	0.97	0.45	0.45	0.54
Philippines	0.5	0.55	0.45	0.94	0.96	0.91	0.99	0.84	1.19
Poland	1.1	1	1.22	0.95	0.71	1.21	-0.11	-0.27	0.04
Portugal	1.62	2.06	1.15	2.88	3.5	2.21	0.89	0.81	1.06
Puerto Rico	1.54	1.65	1.42	2.26	2.6	1.89	0.26	0.14	0.31
Qatar	0.27	0.24	0.29	0.16	0.16	0.16	0.08	0.39	0.00
Romania	0.5	0.31	0.69	0.66	0.47	0.87	0.08	0.21	0.02
Russia	0.33	0.37	0.29	1.21	0.85	1.62	2.00	1.03	3.39
Rwanda	1.71	1.94	1.48	1.17	1.26	1.07	0.55	0.56	0.64
Saint Lucia	3.33	4.16	2.48	3.7	4.29	3.09	0.04	-0.06	0.21
Saint Vincent and the Grenadines	3.36	3.38	3.33	1.66	2.1	1.23	-0.59	-0.43	-0.71
Samoa	2.3	2.94	1.71	3.09	4.22	2.03	0.32	0.13	0.41
Sao Tome and Principe	2.3	2.52	2.07	1.71	1.82	1.6	0.22	0.15	0.32
Saudi Arabia	0.63	0.82	0.49	0.64	0.7	0.59	0.68	0.71	0.87
Senegal	1.21	1.38	1.05	1.05	1.18	0.91	0.51	0.46	0.57
Serbia	0.61	0.52	0.7	0.87	0.79	0.96	0.11	0.14	0.09
Seychelles	1.65	1.56	1.75	1.68	1.53	1.83	0.13	0.05	0.21
Sierra Leone	1.53	1.53	1.54	1.14	1.19	1.09	0.21	0.17	0.28
Singapore	0.64	0.54	0.74	1.37	1.39	1.35	0.86	1.04	0.72
Slovakia	0.93	0.81	1.05	0.85	0.7	1.02	0.07	-0.08	0.24
Slovenia	0.95	0.85	1.06	0.74	0.62	0.86	-0.22	-0.31	-0.12
Solomon Islands	2.35	3.13	1.62	3	3.98	2.05	0.50	0.46	0.56
Somalia	2.01	2.32	1.69	1.39	1.61	1.17	0.02	0.04	0.03
South Africa	1.12	1.33	0.9	1.07	1.14	1	-0.18	-0.24	-0.09
South Korea	1.78	2.34	1.23	1.1	1.3	0.89	-0.46	-0.52	-0.35
South Sudan	1.82	2.12	1.51	1.48	1.69	1.27	0.21	0.20	0.29
Spain	2.48	3.33	1.6	3.56	4.61	2.48	0.46	0.34	0.66
Sri Lanka	1.5	1.26	1.74	1.81	1.33	2.32	0.29	0.01	0.51
Sudan	1.4	1.77	1.03	1.33	1.66	0.99	0.72	0.79	0.79
Suriname	1.14	1.59	0.7	1.27	1.56	0.98	0.03	-0.06	0.26
Swaziland	0.83	0.96	0.68	1.06	1.05	1.07	-0.15	-0.29	0.05
Sweden	1.76	2.24	1.26	2.37	2.98	1.76	0.59	0.47	0.75
Switzerland	1.73	2.15	1.29	6.47	7.66	5.25	3.41	3.01	4.08
Syria	1.07	1.58	0.59	0.96	1.48	0.46	-0.31	-0.04	-0.48
Taiwan	1.32	1.15	1.48	3.11	3.03	3.19	0.75	0.98	0.59
Tajikistan	0.25	0.23	0.28	0.22	0.18	0.26	0.63	0.60	0.66
Tanzania	1.24	1.3	1.17	1.15	1.16	1.14	0.57	0.55	0.61
Thailand	1.58	1.63	1.54	4.08	3.88	4.29	0.88	0.82	1.06
The Bahamas	1.71	2.04	1.36	2.34	2.78	1.89	0.20	0.11	0.30
The Gambia	1.21	1.39	1.04	0.78	0.8	0.76	0.17	0.18	0.22
Timor-Leste	1.45	1.59	1.31	1.16	1.28	1.04	0.85	0.94	1.05
Togo	1.43	1.65	1.21	1.04	1.03	1.05	0.02	-0.02	0.11
Tonga	2.65	3.58	1.74	3.94	5.2	2.69	0.37	0.37	0.42
Trinidad and Tobago	1.4	1.51	1.29	1.88	1.98	1.78	0.11	0.15	0.09
Tunisia	2.14	2.49	1.79	2.55	2.83	2.27	0.20	0.20	0.25
Turkey	1.58	2.07	1.07	1.33	1.74	0.9	0.49	0.54	0.52
Turkmenistan	0.38	0.32	0.44	0.44	0.33	0.55	0.62	0.58	0.67
Uganda	1.23	1.4	1.06	1.19	1.16	1.23	0.89	0.72	1.20
Ukraine	0.73	0.65	0.83	1.05	0.85	1.29	0.19	0.14	0.24
United Arab Emirates	0.8	0.86	0.77	1.04	0.88	1.1	0.66	0.82	0.76
United Kingdom	1.39	1.73	1.02	2.36	2.72	1.99	1.06	0.88	1.40
United States	1.78	1.89	1.67	2.55	2.7	2.4	0.48	0.43	0.54
Uruguay	3.24	4.29	2.12	5.1	6.53	3.56	0.43	0.22	0.73
Uzbekistan	0.19	0.18	0.19	0.21	0.17	0.25	0.32	0.19	0.44
Vanuatu	2.57	3.27	1.91	3.25	4.11	2.4	0.39	0.31	0.47
Venezuela	0.41	0.4	0.42	0.38	0.35	0.41	-0.25	-0.25	-0.23
Vietnam	0.92	0.92	0.93	1.4	1.44	1.36	0.62	0.72	0.56
Virgin Islands, U.S.	2.09	2.46	1.7	4.76	5.22	4.25	0.06	0.00	0.16

Yemen	1.4	1.88	0.91	1.21	1.6	0.82	0.73	0.77	1.04
Zambia	1.19	1.44	0.92	1.36	1.29	1.42	0.64	0.41	1.06
Zimbabwe	0.25	0.15	0.36	0.36	0.16	0.56	0.17	-0.14	0.30
**Hemorrhagic and other stroke**									
Afghanistan	45.39	63.69	27.98	57.62	79.88	36.73	0.79	0.83	0.81
Albania	68.27	66.60	69.86	134.18	131.25	137.15	0.43	0.44	0.41
Algeria	24.46	26.26	22.7	28.22	30.21	26.25	0.25	0.25	0.27
American Samoa	35.30	27.08	43.07	23.2	24.21	22.19	-0.18	-0.11	-0.24
Andorra	29.19	30.54	27.76	29.71	28.63	30.84	-0.16	-0.18	-0.11
Angola	44.63	49.47	39.67	33.72	40.01	27.34	0.56	0.58	0.51
Antigua and Barbuda	46.83	48.42	45.13	37.45	36.12	38.9	-0.11	-0.14	-0.08
Argentina	30.99	28.89	33.17	27.28	26.41	28.18	-0.14	-0.20	-0.08
Armenia	36.8	40.33	33.07	48.72	50.15	47.07	0.01	-0.04	0.07
Australia	35.73	42.5	28.93	31.12	36.58	25.65	-0.09	-0.13	-0.04
Austria	51.82	60.04	42.86	30	34.55	25.22	-0.35	-0.35	-0.34
Azerbaijan	58.51	67.05	49.60	58.19	59.79	56.57	0.22	0.16	0.34
Bahrain	10.64	11.53	9.98	4.63	5.15	4.31	-0.25	-0.26	-0.23
Bangladesh	33.17	38.02	28.54	53.41	51.83	54.97	1.74	1.63	1.97
Barbados	43.43	45.25	41.46	54.67	57.75	51.32	0.03	0.02	0.05
Belarus	52.97	59.99	45.02	56.10	56.53	55.60	-0.23	-0.25	-0.16
Belgium	49.11	56.4	41.48	41.32	46.06	36.4	-0.12	-0.15	-0.07
Belize	20.7	20.62	20.77	19.72	17.12	22.34	0.05	0.05	0.07
Benin	24.47	25.32	23.58	29.68	29.71	29.66	1.02	0.98	1.04
Bermuda	27.43	25.84	29.04	24.04	21.84	26.28	0.03	-0.06	0.11
Bhutan	21.09	22.62	19.66	20.47	18.04	22.56	0.73	0.67	0.82
Bolivia	26.89	31.44	22.37	24.46	27.97	20.97	0.38	0.30	0.48
Bosnia and Herzegovina	47.45	51.39	43.50	30.09	31.22	28.95	-0.52	-0.56	-0.47
Botswana	22.96	28.42	17.36	31.56	34.85	28.26	-0.07	-0.12	0.05
Brazil	46.57	44.60	48.57	46.53	45.11	47.99	-0.07	-0.09	-0.06
Brunei	17.77	18.51	17.1	17.03	17.03	17.03	-0.14	-0.17	-0.11
Bulgaria	145.60	146.25	144.93	123.15	127.08	119.04	-0.31	-0.33	-0.30
Burkina Faso	15.6	18.11	12.96	14.45	17.21	11.65	0.69	0.67	0.68
Burundi	69.79	79.37	59.87	25.59	27.87	23.26	-0.14	-0.15	-0.11
Cambodia	51.94	56.08	47.51	47.59	48.89	46.22	0.75	0.77	0.78
Cameroon	29.98	31.76	28.17	29.9	30.79	29.01	0.21	0.19	0.21
Canada	16.09	17.6	14.56	14.77	16.2	13.32	-0.16	-0.22	-0.11
Cape Verde	41.77	36.71	47.36	39.49	36.4	42.65	0.21	0.27	0.16
Central African Republic	73.08	77.74	68.27	71.39	74.52	68.17	0.10	0.03	0.13
Chad	22.36	25.36	19.32	24.41	26.18	22.63	0.54	0.45	0.63
Chile	24.44	23.99	24.9	29.6	29.87	29.32	0.11	0.02	0.18
China	80.79	77.06	84.33	81.90	65.50	97.36	0.14	0.12	0.16
Colombia	20.56	21.98	19.12	20.59	21.95	19.2	0.09	0.00	0.16
Comoros	44.27	45.81	42.73	24.37	26.14	22.64	0.16	0.22	0.14
Congo	59.57	66.84	52.25	43.01	57.45	28.6	0.05	0.06	-0.05
Costa Rica	11.1	11.63	10.57	15.76	16.36	15.16	0.14	0.11	0.17
Cote d'Ivoire	30.51	26.85	33.88	32.84	29.23	36.33	0.40	0.44	0.35
Croatia	90.44	98.02	82.39	77.05	85.04	68.47	-0.25	-0.29	-0.22
Cuba	29.28	29.74	28.84	38.26	37.34	39.16	-0.04	-0.12	0.04
Cyprus	50.21	47.32	53.17	23.88	20.55	27.44	-0.37	-0.39	-0.34
Czech Republic	75.76	84.42	66.58	36.5	40.48	32.38	-0.42	-0.44	-0.40
Democratic Republic of the Congo	31.66	39.27	23.86	31.02	34.99	27.03	0.52	0.41	0.74
Denmark	59.68	66.55	52.62	38.87	41.86	35.84	-0.19	-0.22	-0.14
Djibouti	29.98	26.5	33.41	36.75	32.14	41.31	0.37	0.42	0.36
Dominica	24.58	26.18	22.97	37.74	35.62	39.9	0.16	0.16	0.19
Dominican Republic	17.61	17.08	18.14	35.38	32.76	38	0.73	0.65	0.81
Ecuador	15.64	15.37	15.91	17.55	17.36	17.74	0.12	0.13	0.11
Egypt	48.94	45.03	52.81	37.75	33.55	41.87	0.08	0.13	0.04
El Salvador	20.17	19.98	20.37	15.53	16.01	14.99	-0.18	-0.18	-0.20
Equatorial Guinea	74.21	83.17	65.65	28.99	31.44	26.65	-0.19	-0.20	-0.17
Eritrea	37.67	43.06	32.21	33.21	41.12	25.33	0.36	0.38	0.27
Estonia	45.01	50.82	38.39	24.32	26.68	21.54	-0.42	-0.47	-0.36
Ethiopia	45.87	47.42	44.32	23.61	25.1	22.11	0.50	0.50	0.47
Federated States of Micronesia	46.70	43.65	49.61	41.84	40.64	42.98	0.01	0.02	0.00
Fiji	44.20	48.21	40.31	45.89	54.10	37.94	-0.28	-0.27	-0.30
Finland	37.32	41.38	33.02	27.01	29.3	24.65	-0.26	-0.28	-0.22
France	44.09	48.07	39.89	34.99	38.47	31.29	-0.23	-0.24	-0.23
Gabon	64.47	61.84	67.21	42.06	46.07	38.14	0.02	0.11	-0.07
Georgia	126.31	141.11	109.99	148.87	147.95	149.88	-0.09	-0.12	-0.02
Germany	52.37	61.16	42.94	35.86	40.27	31.3	-0.30	-0.30	-0.28
Ghana	30.27	44.75	16.24	30.69	38.75	22.55	0.50	0.36	0.90

Greece	87.85	99.41	75.99	81.62	90.12	72.69	-0.24	-0.28	-0.19
Greenland	36.01	39.79	32.75	30.32	26.1	33.99	0.00	-0.14	0.17
Grenada	51.71	58.78	44.46	48.29	53.35	43.25	-0.08	-0.11	-0.03
Guam	23.68	22.3	24.89	38.64	38.47	38.82	-0.15	-0.15	-0.16
Guatemala	10.61	11.24	9.98	13.27	14.38	12.12	0.85	0.85	0.82
Guinea	27.7	33.24	22.04	31.52	33.71	29.35	0.89	0.75	1.10
Guinea-Bissau	36.90	40.53	33.19	40.50	44.62	36.32	0.56	0.56	0.58
Guyana	57.79	53.27	62.46	56.25	53.06	59.41	-0.05	0.02	-0.09
Haiti	54.77	74.28	34.69	48.61	63.36	33.52	0.44	0.36	0.61
Honduras	40.83	40.64	41.02	25.98	25.88	26.07	-0.34	-0.39	-0.28
Hungary	74.99	77.73	72.02	35.55	37.11	33.83	-0.45	-0.49	-0.41
Iceland	29.76	33.14	26.42	21.14	20.44	21.85	-0.18	-0.22	-0.11
India	32.83	34.47	31.29	33.04	31.1	34.84	0.39	0.38	0.40
Indonesia	52.56	56.32	48.84	73.87	74.26	73.49	0.73	0.70	0.78
Iran	23.22	19.79	26.53	25.86	21.69	29.97	0.53	0.71	0.43
Iraq	31.41	32.99	29.85	24.49	24.63	24.35	-0.05	0.02	-0.08
Ireland	32.84	38.1	27.53	21.39	23	19.74	-0.17	-0.19	-0.13
Israel	27.09	28.88	25.3	20.35	21.03	19.66	-0.19	-0.25	-0.12
Italy	52.13	55.75	48.29	57.48	63.8	50.77	-0.03	-0.06	-0.01
Jamaica	43.21	49.75	36.5	58.67	63.86	53.44	0.06	0.06	0.08
Japan	40.17	39.88	40.46	52.41	51.61	53.26	-0.20	-0.24	-0.16
Jordan	13.29	14.52	12.18	6.98	6.37	7.55	-0.10	-0.15	-0.04
Kazakhstan	49.63	58.93	39.74	47.21	50.06	44.16	-0.06	-0.08	0.02
Kenya	17.88	20.62	15.12	17.34	17.97	16.72	0.25	0.19	0.36
Kiribati	68.60	52.45	85.10	73.80	49.43	98.80	0.35	0.31	0.36
Kuwait	3.87	3.73	3.99	4.13	2.76	5.2	0.44	0.01	0.80
Kyrgyzstan	61.52	71.93	50.66	49.61	48.14	51.11	0.05	-0.01	0.20
Laos	66.12	74.14	58.09	42.29	43.04	41.54	0.56	0.52	0.65
Latvia	82.49	98.65	63.88	47.9	52.75	42.24	-0.41	-0.47	-0.31
Lebanon	25.71	36.66	14.45	16.95	24.93	9.05	-0.02	-0.12	0.04
Lesotho	29.78	31.91	27.52	46.66	52.37	40.83	-0.10	-0.12	-0.10
Liberia	26.34	28.68	23.99	23.22	26.17	20.33	1.04	0.94	1.13
Libya	18.7	19.66	17.84	19.86	20.09	19.63	-0.12	-0.05	-0.16
Lithuania	31.43	35.2	27.24	31.83	34.16	29.11	-0.12	-0.16	-0.06
Luxembourg	69.17	79.26	58.67	29.82	33.94	25.68	-0.35	-0.39	-0.30
Macedonia	78.30	80.71	75.91	89.66	93.60	85.68	-0.02	-0.05	-0.01
Madagascar	52.11	52.56	51.66	45.32	45.02	45.64	0.44	0.43	0.43
Malawi	20.45	25.76	14.96	15.51	16.64	14.38	0.46	0.28	0.79
Malaysia	34.98	34.98	34.97	33.26	26.85	39.78	-0.12	-0.16	-0.07
Maldives	20.76	17.06	24.28	15.64	10.18	21.08	0.76	0.75	0.73
Mali	33.74	42.43	24.85	22.13	28.2	16.19	0.19	0.20	0.19
Malta	34.41	34.88	33.92	31.48	32.89	30.06	-0.21	-0.22	-0.20
Marshall Islands	40.44	34.72	45.91	40.51	36.95	43.95	-0.01	-0.01	-0.01
Mauritania	24.97	29.05	20.88	18.76	23.03	14.54	0.40	0.42	0.34
Mauritius	59.80	51.26	68.38	52.03	46.88	57.30	-0.31	-0.32	-0.30
Mexico	13.03	13.75	12.29	16.84	17.7	15.97	0.24	0.16	0.31
Moldova	65.44	71.65	58.62	55.12	53.33	57.06	-0.11	-0.19	0.00
Mongolia	56.84	56.90	56.78	111.07	101.94	120.39	1.32	1.48	1.27
Montenegro	167.91	206.02	129.48	192.18	213.84	170.00	-0.07	-0.05	-0.04
Morocco	20.77	22.61	18.92	22.45	24.53	20.32	0.23	0.22	0.24
Mozambique	30.39	28.61	32.34	33.89	31.07	36.84	0.71	0.63	0.73
Myanmar	62.46	62.06	62.88	64.85	56.51	73.57	0.50	0.45	0.57
Namibia	28.86	32.6	24.95	21.3	21.36	21.24	-0.16	-0.21	-0.08
Nepal	22.47	23.5	21.44	25.08	22.94	27.36	0.97	0.93	1.02
Netherlands	39.45	44.32	34.47	31.22	34.86	27.5	-0.20	-0.23	-0.16
New Zealand	40.54	48.74	32.1	31.5	38.34	24.4	-0.14	-0.16	-0.12
Nicaragua	11.02	11.72	10.31	16.87	17.63	16.08	0.79	0.76	0.83
Niger	20.74	21.43	20.02	20.55	21.77	19.35	1.13	1.21	1.04
Nigeria	22.19	24.08	20.34	13.45	12.41	14.46	0.13	0.02	0.29
North Korea	64.57	76.17	52.28	108.20	116.19	99.86	0.25	0.17	0.38
Northern Mariana Islands	26.74	27.38	26.17	14.36	10.43	18.31	-0.25	-0.33	-0.17
Norway	59.12	65.73	52.36	32.09	37.06	27.18	-0.28	-0.32	-0.24
Oman	14.15	17.86	11.2	11.64	13.23	10.83	0.33	0.25	0.54
Pakistan	31.66	28.71	34.4	35.97	31.13	40.55	0.46	0.50	0.44
Palestine	23.8	26.08	21.58	19.74	18.28	21.16	0.09	0.05	0.18
Panama	19.49	19.05	19.92	25.46	23.43	27.48	0.11	0.05	0.18
Papua New Guinea	60.21	70.08	50.79	56.33	69.23	43.93	0.11	0.14	0.07
Paraguay	31.61	34.73	28.55	43.88	44.35	43.42	0.08	0.07	0.11
Peru	19.64	18.57	20.72	11.54	11.79	11.29	-0.10	-0.08	-0.13
Philippines	27.21	21.6	32.71	47.55	40.29	54.68	0.85	0.96	0.78

Poland	40.99	43.45	38.40	36.9	37.04	36.75	-0.07	-0.13	0.00
Portugal	109.07	112.57	105.31	67.46	68.29	66.57	-0.34	-0.36	-0.32
Puerto Rico	18.91	17.23	20.69	31.23	33.4	28.89	0.41	0.40	0.38
Qatar	9.73	11.32	8.94	4.62	4.61	4.62	-0.15	-0.20	-0.09
Romania	87.59	93.38	81.63	124.09	129.75	118.06	0.15	0.12	0.17
Russia	59.92	71.52	46.75	65.31	67.07	63.26	-0.10	-0.17	0.04
Rwanda	39.22	49.86	28.21	19.63	22.75	16.23	0.13	0.04	0.26
Saint Lucia	46.28	50.60	41.84	54.38	61.8	46.68	0.10	0.11	0.08
Saint Vincent and the Grenadines	34.06	40.22	27.83	47.56	49.06	46.09	0.16	0.13	0.25
Samoa	36.37	31.41	40.94	33.52	36.69	30.54	-0.13	-0.12	-0.13
Sao Tome and Principe	34.20	36.34	32.02	33.44	37.78	29.07	0.56	0.59	0.49
Saudi Arabia	11.13	11.63	10.74	10.1	8.76	11.13	0.50	0.49	0.55
Senegal	23.86	24.71	22.99	27.74	30.7	24.67	0.99	1.05	0.85
Serbia	85.24	96.25	73.93	74.52	81.85	66.84	-0.33	-0.36	-0.29
Seychelles	44.89	43.27	46.52	29.14	26.55	31.66	-0.29	-0.34	-0.23
Sierra Leone	23.6	19.43	27.85	28.09	31.31	24.81	0.91	1.41	0.55
Singapore	27.4	29.01	25.83	19.31	20.41	18.17	-0.39	-0.44	-0.34
Slovakia	55.02	57.31	52.62	30.46	30.89	30	-0.36	-0.43	-0.28
Slovenia	41.39	43.29	39.38	41.04	44.9	37.11	-0.01	-0.03	0.01
Solomon Islands	60.51	61.15	59.91	56.53	60.53	52.65	0.08	0.10	0.05
Somalia	49.74	59.99	39.30	37.14	45.31	28.89	0.15	0.17	0.14
South Africa	35.73	45.87	25.36	35.77	41.48	29.85	-0.14	-0.20	-0.03
South Korea	52.88	57.16	48.62	32.96	33.93	31.98	-0.46	-0.49	-0.42
South Sudan	36.53	41.68	31.34	32.4	36.70	28.11	0.36	0.34	0.39
Spain	44.82	47.69	41.83	35.92	39.77	31.98	-0.18	-0.20	-0.18
Sri Lanka	16.04	12.61	19.4	26.79	22.78	31.11	0.78	0.72	0.80
Sudan	36.50	42.04	31.01	29.87	33.91	25.85	0.43	0.46	0.42
Suriname	34.12	36.15	32.16	54.90	55.77	54.03	0.48	0.47	0.50
Swaziland	26.7	29.94	23.08	37.89	42.35	33.34	-0.10	-0.13	-0.06
Sweden	49.63	55.44	43.69	36.31	41	31.61	-0.14	-0.18	-0.09
Switzerland	44.95	50.14	39.52	24.64	28.24	20.95	-0.36	-0.37	-0.34
Syria	38.11	32.96	43.09	24.88	21.35	28.31	-0.50	-0.35	-0.58
Taiwan	41.97	38.63	45.07	33.53	28.43	38.64	-0.40	-0.45	-0.36
Tajikistan	42.12	47.54	36.64	30.59	31.85	29.37	0.35	0.35	0.41
Tanzania	23.55	26.78	20.26	19.53	20.69	18.36	0.38	0.30	0.46
Thailand	48.27	47.71	48.84	53.49	42.53	64.76	-0.29	-0.39	-0.19
The Bahamas	26.77	27.47	26.07	36.16	37.26	35.01	0.17	0.10	0.23
The Gambia	19.32	21.55	17.12	17.4	16.6	18.22	0.65	0.60	0.80
Timor-Leste	32.89	37.91	28.00	33.19	39.25	27.32	1.12	1.15	1.09
Togo	26.16	28.88	23.38	26.57	24.31	28.87	0.40	0.29	0.54
Tonga	30.32	37.74	23.09	33.11	39.51	26.75	-0.01	-0.03	0.04
Trinidad and Tobago	36.31	36.59	36.02	45.5	44.47	46.56	0.03	0.06	0.02
Tunisia	26.62	22.57	30.61	28.04	23.77	32.41	0.05	0.10	0.02
Turkey	20.87	23.38	18.31	18.71	18.38	19.05	0.56	0.40	0.80
Turkmenistan	67.64	72.46	62.69	76.16	70.70	81.81	0.58	0.48	0.72
Uganda	33.13	41.26	24.9	23.19	23.6	22.77	0.35	0.14	0.70
Ukraine	64.85	73.80	54.43	61.10	60.11	62.26	-0.23	-0.31	-0.11
United Arab Emirates	17.7	19.9	16.56	16.8	16.53	16.89	0.23	0.50	0.24
United Kingdom	54.95	64.55	44.79	38.04	43.52	32.43	-0.16	-0.19	-0.11
United States	19.09	21.29	16.76	16.44	17.64	15.21	-0.11	-0.18	-0.03
Uruguay	40.03	41.59	38.37	42.94	48.68	36.79	-0.03	-0.07	-0.02
Uzbekistan	41.99	48.45	35.39	40.46	42.08	38.78	0.13	0.09	0.21
Vanuatu	63.55	61.46	65.52	60.77	64.61	57.02	0.04	0.07	0.00
Venezuela	16.81	17.66	15.97	26.16	25.9	26.42	0.26	0.26	0.29
Vietnam	61.16	58.87	63.52	77.31	71.95	82.79	0.32	0.31	0.34
Virgin Islands, U.S.	23.82	22.49	25.23	50.59	45.13	56.59	-0.01	-0.06	0.04
Yemen	35.11	41.97	28.12	31.20	36.62	25.9	0.69	0.71	0.71
Zambia	22.84	29.92	15.64	25.07	24.18	25.96	0.57	0.25	1.24
Zimbabwe	18.15	21.39	14.87	21.67	20.74	22.64	-0.02	-0.19	0.23
**Hypertensive heart disease**									
Afghanistan	12.34	18.67	6.3	10.89	16.67	5.46	0.25	0.32	0.20
Albania	4.05	4.31	3.8	4.13	4.6	3.65	-0.26	-0.21	-0.31
Algeria	7.7	10.25	5.21	11.27	14.46	8.11	0.60	0.54	0.73
American Samoa	10.32	7.92	12.59	5.1	4.73	5.46	-0.38	-0.40	-0.35
Andorra	4.12	4.83	3.38	4.78	5.38	4.14	-0.02	0.01	0.01
Angola	15.3	21.54	8.91	12.01	16.96	7.01	0.65	0.56	0.77
Antigua and Barbuda	26.33	32.93	19.26	18.46	21.86	14.75	-0.22	-0.23	-0.18
Argentina	11.24	11.16	11.32	14.28	15.87	12.62	0.25	0.25	0.21
Armenia	11.75	14.58	8.75	22.02	26.1	17.32	0.47	0.43	0.54
Australia	3.89	5.21	2.56	4.07	5.49	2.64	0.10	0.07	0.13

Austria	15.24	20.24	9.8	26.72	36.57	16.38	0.98	1.06	0.87
Azerbaijan	5.03	6.24	3.77	5.66	6.46	4.85	0.39	0.37	0.53
Bahrain	2.05	3.14	1.25	1.36	2.35	0.76	0.14	0.24	0.08
Bangladesh	7.94	7.93	7.95	7.63	7.26	7.99	0.63	0.75	0.55
Barbados	17.28	21.37	12.86	17.54	20.97	13.81	-0.16	-0.22	-0.09
Belarus	6.54	7.82	5.09	15.9	17.55	14	0.79	0.82	0.88
Belgium	4.7	6.26	3.06	5.85	8.41	3.18	0.31	0.40	0.11
Belize	11	12.14	9.88	8.34	8.19	8.49	-0.16	-0.15	-0.14
Benin	5.12	7.25	2.85	4.86	6.68	3.04	0.59	0.56	0.76
Bermuda	10.06	9.3	10.82	7.29	6.72	7.88	-0.14	-0.19	-0.11
Bhutan	8.28	8.63	7.95	7.45	6.97	7.86	0.61	0.71	0.58
Bolivia	2.68	3.54	1.83	3.4	4.67	2.13	0.94	0.96	0.85
Bosnia and Herzegovina	3.74	4.64	2.83	6.39	8.11	4.66	0.33	0.31	0.32
Botswana	12.88	17.43	8.21	17.05	22.29	11.81	-0.12	-0.10	-0.07
Brazil	7.4	7.87	6.92	11.56	12.51	10.59	0.45	0.43	0.45
Brunei	2.94	2.36	3.46	3.58	2.95	4.18	0.11	0.17	0.10
Bulgaria	27.94	29.07	26.77	77.39	83.76	70.70	1.26	1.23	1.27
Burkina Faso	7.5	10.81	4.03	7.21	10.71	3.66	0.76	0.76	0.73
Burundi	31.63	41.92	20.98	14.15	19.64	8.55	0.01	0.08	-0.08
Cambodia	10.08	11.14	8.95	9.25	9.81	8.66	0.76	0.79	0.79
Cameroon	6.17	8.51	3.79	5.65	8.18	3.13	0.13	0.22	-0.01
Canada	2.22	2.88	1.54	2.76	3.5	2.01	0.14	0.03	0.27
Cape Verde	4.6	6.21	2.81	3.94	5.63	2.2	0.14	0.24	0.04
Central African Republic	25.44	34.06	16.52	24.75	31.59	17.73	0.10	0.01	0.23
Chad	4.88	7.36	2.37	4.32	6.35	2.3	0.25	0.22	0.38
Chile	9.23	10.05	8.38	16.66	20.15	13.08	0.65	0.65	0.57
China	20.04	20.73	19.39	18.13	16.71	19.47	0.02	0.07	0.02
Colombia	11.41	12.85	9.95	9.57	10.45	8.65	-0.08	-0.18	0.02
Comoros	19.47	24.54	14.43	10.9	14.32	7.55	0.18	0.24	0.13
Congo	22.36	30.9	13.77	16.83	25.85	7.82	0.11	0.05	0.01
Costa Rica	5.98	6.88	5.1	13.07	15.07	11.06	0.76	0.73	0.78
Cote d'Ivoire	5.66	7.43	4.03	4.94	6.64	3.3	0.14	0.20	0.04
Croatia	40.16	55.16	24.22	30.44	39.55	20.66	-0.33	-0.41	-0.18
Cuba	5.11	5.69	4.54	17.91	19.02	16.8	1.58	1.35	1.84
Cyprus	12.07	13.74	10.36	7.55	8.48	6.55	-0.15	-0.10	-0.16
Czech Republic	3.65	3.92	3.37	8.45	9.69	7.18	1.81	1.88	1.73
Democratic Republic of the Congo	14.3	21.18	7.24	14.09	19.23	8.93	0.55	0.44	0.92
Denmark	4.95	5.97	3.91	4.77	5.66	3.87	0.21	0.18	0.26
Djibouti	10.79	13.36	8.26	14.05	16.44	11.67	0.48	0.45	0.63
Dominica	33.78	40.76	26.74	30.54	32.8	28.24	-0.31	-0.31	-0.28
Dominican Republic	8.05	8.5	7.61	14.78	15.85	13.71	0.59	0.61	0.56
Ecuador	7.71	9.01	6.42	14.69	15.39	13.98	0.90	0.72	1.17
Egypt	9.46	12.74	6.21	9.47	12.1	6.9	0.41	0.45	0.47
El Salvador	5.54	6.48	4.56	8.5	10.36	6.4	0.63	0.63	0.52
Equatorial Guinea	29.15	40.95	17.85	11.94	15.5	8.55	-0.13	-0.16	0.03
Eritrea	14.23	19.04	9.36	13.26	19.28	7.27	0.45	0.48	0.25
Estonia	8.66	10.19	6.92	83.92	105.78	58.25	9.40	9.52	8.77
Ethiopia	18.24	19.96	16.52	11.49	14.11	8.86	0.82	1.01	0.59
Federated States of Micronesia	14.34	13.34	15.3	12.62	12.28	12.94	0.00	0.02	-0.01
Fiji	12.4	13.3	11.52	24.31	26.6	22.09	0.37	0.30	0.43
Finland	8.55	11.43	5.49	13.52	17.64	9.27	0.63	0.56	0.78
France	7.62	10.3	4.79	8.71	11.71	5.51	0.11	0.08	0.14
Gabon	27.5	36.18	18.46	17.24	24.93	9.71	0.00	0.05	-0.12
Georgia	10.29	12.77	7.55	59.66	61.74	57.38	3.60	3.28	4.53
Germany	19.47	26.14	12.31	28.01	39.13	16.51	0.48	0.59	0.33
Ghana	15.8	24.63	7.25	7.43	12.83	1.96	-0.29	-0.15	-0.60
Greece	8.86	11.81	5.84	18.81	23.16	14.25	0.75	0.56	1.06
Greenland	3.02	3.81	2.34	3.98	4.06	3.91	0.58	0.40	0.89
Grenada	19.25	23.46	14.93	18.19	23.33	13.08	-0.06	-0.02	-0.12
Guam	33.15	31.43	34.65	22.4	20.58	24.17	-0.64	-0.66	-0.62
Guatemala	4.28	5.42	3.13	4.4	5.74	3	0.52	0.54	0.44
Guinea	6.63	10.47	2.69	6	8.93	3.08	0.51	0.48	0.82
Guinea-Bissau	7.55	10.99	4.03	6.78	9.95	3.58	0.32	0.33	0.32
Guyana	31.22	33.56	28.81	23.09	24.31	21.89	-0.27	-0.25	-0.27
Haiti	24.03	31.65	16.2	20.88	26.11	15.53	0.41	0.31	0.61
Honduras	8.24	8.99	7.5	13.31	15.16	11.46	0.69	0.64	0.72
Hungary	33.48	41.59	24.7	38.35	47.47	28.29	0.33	0.21	0.45
Iceland	3.56	3.98	3.15	4.05	4.38	3.7	0.32	0.40	0.27
India	12.54	12.32	12.74	13.44	13.32	13.55	0.48	0.66	0.34
Indonesia	7.32	8.69	5.96	8.61	10.29	6.94	0.46	0.56	0.39

Iran	12.6	12.67	12.54	20.92	19.74	22.09	1.30	1.49	1.25
Iraq	10.03	13.15	6.97	7.42	9.55	5.35	-0.09	-0.01	-0.12
Ireland	3.3	4.12	2.47	3	3.56	2.43	0.16	0.16	0.19
Israel	6.34	7.54	5.14	2.3	3.07	1.52	-0.61	-0.58	-0.65
Italy	21.73	28.64	14.39	36.39	47.78	24.28	0.48	0.37	0.60
Jamaica	30.51	35.81	25.07	21.62	24.94	18.28	-0.44	-0.39	-0.46
Japan	10.3	12.99	7.5	8.2	11.21	5.04	-0.51	-0.49	-0.57
Jordan	13.03	16.17	10.19	11.41	11.98	10.88	0.50	0.45	0.66
Kazakhstan	8.15	9.5	6.72	13.96	14.61	13.26	0.70	0.67	0.82
Kenya	8.32	11.69	4.92	8.42	11.12	5.71	0.31	0.30	0.46
Kiribati	12.82	11.86	13.79	6.36	5.6	7.13	-0.37	-0.33	-0.39
Kuwait	5.2	5.7	4.76	3.93	4.03	3.85	0.02	-0.04	0.12
Kyrgyzstan	7.37	8.27	6.44	6.83	6.57	7.09	0.21	0.18	0.31
Laos	12.97	15.18	10.76	8.62	9.43	7.79	0.63	0.64	0.68
Latvia	2.92	3.52	2.22	21.88	26.66	16.32	6.73	6.54	7.06
Lebanon	8.56	11.05	6	5.3	7.72	2.9	-0.08	-0.08	-0.19
Lesotho	19.24	23.25	14.99	28.66	37.7	19.44	-0.16	-0.14	-0.20
Liberia	5.46	7.91	2.99	4.47	6.71	2.27	0.91	0.82	0.94
Libya	3.66	5.24	2.24	4.48	6.15	2.83	0.03	0.10	-0.02
Lithuania	3.87	4.28	3.41	11.47	12.47	10.31	1.60	1.51	1.70
Luxembourg	7.37	10.69	3.91	5.35	7.29	3.4	0.10	-0.02	0.39
Macedonia	19.47	22.3	16.67	22.22	25.97	18.44	-0.02	-0.04	-0.02
Madagascar	22.72	27.55	17.87	21.14	26.66	15.58	0.55	0.62	0.43
Malawi	12.13	17.42	6.66	11.1	14.79	7.39	0.75	0.66	1.11
Malaysia	2.17	2.33	2.01	1.97	1.67	2.27	-0.16	-0.20	-0.07
Maldives	3.86	5.07	2.71	2.16	2.59	1.73	0.31	0.51	0.28
Mali	7.26	11.11	3.33	4.12	6.46	1.82	0.05	0.08	0.01
Malta	4.71	5.25	4.14	6.4	7.32	5.48	0.18	0.15	0.21
Marshall Islands	11.1	9.23	12.89	10.76	9.36	12.11	-0.04	-0.05	-0.02
Mauritania	5.08	7.7	2.44	3.39	5.39	1.41	0.27	0.30	0.13
Mauritius	15.03	15.9	14.16	22.4	24.74	20.01	0.19	0.17	0.18
Mexico	5.68	7.22	4.12	7.45	9.51	5.37	0.26	0.19	0.32
Moldova	3.9	4.52	3.22	16.66	19.43	13.67	3.52	3.70	3.39
Mongolia	8.56	9.05	8.06	5.51	5.28	5.74	-0.23	-0.16	-0.24
Montenegro	2.55	3.49	1.6	3.46	4.37	2.53	0.14	0.21	0.20
Morocco	7.64	10.15	5.12	8.3	11.25	5.29	0.24	0.26	0.20
Mozambique	14.29	18.04	10.21	15.31	19.14	11.3	0.67	0.63	0.78
Myanmar	15.92	22.36	9.22	15.8	20.85	10.52	0.46	0.52	0.57
Namibia	16.06	20.18	11.75	11.45	13.57	9.21	-0.19	-0.19	-0.15
Nepal	8.83	9.02	8.63	8.93	8.17	9.74	0.78	0.79	0.80
Netherlands	3.35	4.47	2.19	4.3	5.79	2.78	0.31	0.27	0.34
New Zealand	4.59	5.7	3.46	4	5.32	2.63	-0.03	0.00	-0.11
Nicaragua	3.66	4.24	3.07	8.55	9.56	7.5	1.73	1.64	1.87
Niger	4.06	6.06	1.99	3.77	5.62	1.96	1.01	1.03	1.11
Nigeria	5.23	7.85	2.67	2.79	4.01	1.61	0.00	0.01	0.12
North Korea	12	16.19	7.57	19.33	24.59	13.83	0.21	0.19	0.33
Northern Mariana Islands	3.07	4.15	2.09	1.7	1.77	1.63	-0.22	-0.23	-0.07
Norway	4.7	5.83	3.54	3.53	4.92	2.16	0.01	0.03	-0.10
Oman	1.25	2.14	0.55	1.57	2.47	1.12	1.04	0.97	2.31
Pakistan	11.37	8.41	14.13	9.95	7.4	12.37	0.13	0.21	0.07
Palestine	5.09	5.74	4.45	6.9	6.91	6.9	0.80	0.82	0.90
Panama	3.94	4.29	3.61	5.77	6.2	5.35	0.25	0.23	0.27
Papua New Guinea	14.4	15.76	13.11	11.81	13.31	10.36	-0.02	-0.02	-0.01
Paraguay	4.09	4.53	3.65	11	11.55	10.47	1.10	1.14	1.12
Peru	2.41	3.16	1.66	2.14	2.85	1.43	0.36	0.32	0.37
Philippines	7.75	6.75	8.72	15.98	14.87	17.07	1.19	1.31	1.09
Poland	9.54	11.53	7.45	10.11	11.51	8.61	0.09	0.02	0.21
Portugal	7.75	10.14	5.17	10.44	13.39	7.3	0.44	0.41	0.51
Puerto Rico	10.45	10.62	10.27	21.09	22.77	19.27	0.73	0.55	0.85
Qatar	1.16	1.94	0.78	0.4	0.72	0.28	-0.38	-0.26	-0.36
Romania	29.1	34.65	23.4	58.42	65.95	50.41	0.63	0.54	0.74
Russia	4.26	5.18	3.2	13.98	15.62	12.05	1.73	1.66	2.01
Rwanda	20.93	29.87	11.67	13.03	17.67	7.98	0.39	0.34	0.52
Saint Lucia	23.41	28.82	17.83	21.88	26.86	16.72	-0.12	-0.15	-0.09
Saint Vincent and the Grenadines	23.34	28.8	17.82	28.18	31.12	25.29	0.00	0.00	0.08
Samoa	12	11.19	12.75	11.7	13.14	10.35	-0.07	-0.11	-0.04
Sao Tome and Principe	2.98	4.52	1.41	2.59	3.97	1.21	0.40	0.36	0.42
Saudi Arabia	0.92	1.52	0.46	1.03	1.5	0.66	0.84	0.96	1.18
Senegal	5.02	7.42	2.6	5.11	7.84	2.29	0.75	0.76	0.54
Serbia	11.87	14.28	9.39	20.37	25.23	15.27	0.33	0.33	0.30

Seychelles	77.24	97.74	56.65	62.68	83.63	42.28	-0.10	-0.08	-0.15
Sierra Leone	4.46	5.71	3.19	5.26	7.78	2.69	0.91	1.07	0.51
Singapore	8.2	9.14	7.29	14.12	14.08	14.17	0.50	0.22	0.84
Slovakia	6.48	7.55	5.36	14.97	17.52	12.28	1.83	1.74	1.92
Slovenia	18.28	24.08	12.12	25.73	35.06	16.24	0.41	0.37	0.43
Solomon Islands	16.23	14.64	17.75	13.98	12.82	15.11	0.00	-0.02	0.04
Somalia	19.34	26.17	12.39	14.39	19.83	8.9	0.14	0.15	0.13
South Africa	21.04	29.56	12.32	22.84	30.15	15.27	-0.07	-0.09	0.02
South Korea	7.6	9.8	5.41	8.44	12.57	4.26	-0.03	0.10	-0.29
South Sudan	16.46	21.63	11.25	15.22	20.33	10.12	0.42	0.43	0.42
Spain	4.85	6.9	2.72	10.01	14.18	5.72	1.11	0.99	1.28
Sri Lanka	4.16	3.18	5.12	8.96	8.76	9.18	1.30	1.63	1.02
Sudan	9.88	14.33	5.48	6.87	10.02	3.74	0.27	0.36	0.18
Suriname	15.33	19.1	11.69	14.23	17.12	11.34	-0.14	-0.14	-0.13
Swaziland	15.51	19.22	11.36	19.69	25.26	14	-0.20	-0.20	-0.19
Sweden	3.89	4.95	2.8	8.29	11.07	5.51	1.51	1.47	1.48
Switzerland	10.15	13.73	6.4	17.93	24.59	11.11	1.08	1.01	1.16
Syria	2.48	2.59	2.36	2.22	2.52	1.92	-0.31	0.00	-0.48
Taiwan	8.04	7.23	8.79	12.37	11.7	13.04	0.17	0.24	0.12
Tajikistan	7.49	9.23	5.74	6.36	7.05	5.69	0.60	0.57	0.77
Tanzania	12.1	15.92	8.21	11.1	14.08	8.08	0.53	0.49	0.62
Thailand	3.66	3.52	3.82	4.03	4.4	3.65	-0.30	-0.15	-0.41
The Bahamas	28.4	31.69	25.07	46.95	54.89	38.66	0.43	0.41	0.42
The Gambia	4.24	6.3	2.22	3.22	4.52	1.9	0.43	0.51	0.47
Timor-Leste	5.85	7.26	4.47	6.43	8.51	4.42	1.32	1.47	1.14
Togo	5.82	8.64	2.93	4.65	6.13	3.13	0.11	0.09	0.36
Tonga	5.36	5.52	5.2	6.27	6.45	6.09	0.07	0.09	0.07
Trinidad and Tobago	23.06	25.75	20.35	20.56	21.39	19.71	-0.26	-0.28	-0.23
Tunisia	5.8	7.01	4.6	4.96	6.08	3.81	-0.13	-0.07	-0.19
Turkey	12.7	14.83	10.52	10.75	12.86	8.57	0.49	0.58	0.43
Turkmenistan	7.59	8.94	6.2	8.84	9.51	8.15	0.64	0.62	0.74
Uganda	17.47	24.14	10.71	12.14	15.15	9.13	0.35	0.28	0.62
Ukraine	7.52	8.92	5.88	15.67	17.95	12.97	0.71	0.73	0.75
United Arab Emirates	3.19	4.65	2.44	2.23	2.84	2	-0.09	0.14	0.00
United Kingdom	5.31	6.04	4.53	5.61	6.19	5.01	0.28	0.23	0.36
United States	10.03	11.48	8.51	11.69	12.27	11.09	0.20	0.06	0.39
Uruguay	9.69	10.5	8.83	16.47	20.63	12.01	0.54	0.57	0.40
Uzbekistan	34.47	34.61	34.33	36.98	35.52	38.48	0.26	0.29	0.25
Vanuatu	18.57	17.23	19.83	15.38	15.62	15.15	-0.10	-0.07	-0.12
Venezuela	12.79	13.99	11.6	14.98	16.38	13.58	-0.05	0.01	-0.09
Vietnam	9.78	11.85	7.66	11.69	14.16	9.16	0.29	0.35	0.26
Virgin Islands, U.S.	16.12	15.65	16.62	40.23	39.86	40.64	0.17	0.20	0.14
Yemen	8.28	11.78	4.71	5.9	8.44	3.41	0.40	0.47	0.37
Zambia	11.4	16.93	5.77	12.67	15.66	9.66	0.60	0.45	1.28
Zimbabwe	4.23	5.47	2.97	4.31	4.81	3.79	-0.16	-0.27	0.04
**Ischemic heart disease**									
Afghanistan	167.76	181.49	154.68	172.94	184.35	162.22	0.45	0.48	0.44
Albania	94.73	75.10	113.42	197.67	164.39	231.48	0.52	0.60	0.46
Algeria	64.78	63.24	66.27	88.02	85.53	90.48	0.48	0.46	0.49
American Samoa	63.36	38.76	86.64	59.96	53.10	66.78	0.19	0.36	0.14
Andorra	126.08	103.23	150.17	137.73	106.94	169.82	-0.10	-0.11	-0.11
Angola	55.12	54.21	56.05	43.37	48.70	37.97	0.64	0.76	0.51
Antigua and Barbuda	111.24	114.01	108.27	93.75	94.16	93.30	-0.06	-0.05	-0.08
Argentina	162.67	141.13	185.05	144.78	144.35	145.24	-0.13	-0.10	-0.15
Armenia	216.61	210.12	223.49	328.31	303.37	357.04	0.15	0.10	0.20
Australia	201.71	184.92	218.58	131.22	125.78	136.68	-0.32	-0.31	-0.32
Austria	271.52	271.81	271.20	220.71	230.09	210.87	-0.08	-0.04	-0.13
Azerbaijan	220.85	218.49	223.31	236.44	207.89	265.32	0.31	0.24	0.39
Bahrain	92.05	80.41	100.62	40.38	35.32	43.48	-0.25	-0.28	-0.23
Bangladesh	27.44	27.46	27.42	87.47	74.64	100.05	4.41	4.22	4.62
Barbados	147.11	143.22	151.30	125.62	127.05	124.06	-0.30	-0.29	-0.30
Belarus	418.18	454.15	377.45	664.22	673.00	654.04	0.15	0.16	0.17
Belgium	238.24	229.29	247.60	161.09	152.41	170.12	-0.29	-0.31	-0.27
Belize	78.70	74.33	83.00	58.51	46.74	70.35	-0.18	-0.21	-0.16
Benin	42.7	43.48	41.86	49.11	46.19	52.04	0.92	0.79	1.02
Bermuda	197.82	144.70	251.61	119.22	94.99	143.91	-0.29	-0.27	-0.30
Bhutan	65.50	67.32	63.81	87.86	76.67	97.49	1.39	1.38	1.42
Bolivia	63.03	66.05	60.03	78.89	87.55	70.25	0.90	0.94	0.84
Bosnia and Herzegovina	167.99	152.85	183.14	175.76	164.94	186.69	-0.20	-0.21	-0.19
Botswana	43.14	44.54	41.71	65.54	60.81	70.27	0.01	-0.03	0.07

Brazil	84.44	73.49	95.57	94.33	83.75	105.27	0.04	0.02	0.04
Brunei	45.48	34.69	55.13	56.00	41.94	69.22	0.11	0.09	0.12
Bulgaria	403.17	371.62	435.61	474.96	458.83	491.89	-0.04	-0.05	-0.04
Burkina Faso	36.9	36.46	37.36	39.76	43.33	36.14	0.96	1.09	0.81
Burundi	59.36	57.08	61.73	30.62	29.48	31.78	0.19	0.23	0.17
Cambodia	85.72	85.03	86.45	87.05	77.12	97.48	0.94	0.83	1.06
Cameroon	42.85	46.59	39.07	46.01	43.24	48.77	0.30	0.14	0.46
Canada	184.15	159.74	208.99	142.47	131.94	153.14	-0.30	-0.30	-0.29
Cape Verde	44.26	37.51	51.73	61.85	62.56	61.13	0.78	1.14	0.52
Central African Republic	75.71	70.93	80.66	76.36	67.70	85.26	0.14	0.04	0.19
Chad	32.47	33.8	31.11	35.56	33.41	37.7	0.54	0.38	0.66
Chile	104.18	96.44	112.13	85.09	79.64	90.69	-0.25	-0.32	-0.19
China	52.24	49.67	54.68	105.62	91.49	118.93	1.28	1.43	1.20
Colombia	68.97	62.69	75.34	78.71	71.69	85.96	0.24	0.15	0.32
Comoros	45.22	38.12	52.27	28.65	27.16	30.11	0.33	0.50	0.24
Congo	92.83	97.11	88.53	67.61	86.09	49.15	0.06	0.09	-0.03
Costa Rica	71.19	63.38	78.87	76.76	67.41	86.10	-0.13	-0.16	-0.11
Cote d'Ivoire	50.48	43.64	56.78	51.48	42.21	60.44	0.33	0.29	0.33
Croatia	275.61	262.48	289.56	333.85	363.14	302.42	0.06	0.14	-0.02
Cuba	197.05	173.92	219.83	212.15	201.46	222.75	-0.21	-0.19	-0.23
Cyprus	221.17	161.38	282.48	120.46	76.82	167.12	-0.28	-0.33	-0.25
Czech Republic	406.28	385.56	428.24	317.87	329.30	306.07	-0.06	-0.01	-0.12
Democratic Republic of the Congo	35.79	39.19	32.31	36.42	36.03	36.81	0.57	0.46	0.76
Denmark	339.50	312.56	367.22	146.94	135.71	158.36	-0.46	-0.46	-0.46
Djibouti	36.75	24.77	48.59	54.69	40.22	69.04	0.67	0.89	0.56
Dominica	98.00	104.22	91.74	105.29	102.32	108.31	-0.19	-0.16	-0.20
Dominican Republic	60.87	52.22	69.42	124.10	115.80	132.46	0.76	0.90	0.65
Ecuador	53.69	48.29	59.02	66.93	58.74	75.12	0.24	0.22	0.26
Egypt	137.48	133.77	141.17	138.06	121.14	154.63	0.41	0.37	0.44
El Salvador	71.46	68.11	74.97	100.02	97.35	103.04	0.49	0.45	0.49
Equatorial Guinea	94.90	97.38	92.53	49.33	51.10	47.65	0.08	0.11	0.06
Eritrea	28.25	24.75	31.79	35.84	37.54	34.14	0.97	1.22	0.75
Estonia	455.30	479.74	427.50	390.09	433.22	339.44	-0.09	-0.09	-0.09
Ethiopia	69.26	63.31	75.25	48.30	44.72	51.90	1.03	0.99	1.04
Federated States of Micronesia	96.10	75.00	116.29	120.43	96.68	143.06	0.41	0.41	0.42
Fiji	109.18	55.40	161.29	167.83	109.61	224.12	0.07	0.28	0.03
Finland	315.70	292.73	340.07	236.13	225.44	247.15	-0.23	-0.22	-0.24
France	143.75	136.02	151.90	129.15	122	136.77	-0.13	-0.15	-0.12
Gabon	103.59	98.49	108.90	66.85	74.07	59.79	0.01	0.12	-0.10
Georgia	373.05	373.28	372.80	410.02	367.79	456.20	-0.15	-0.17	-0.12
Germany	342.59	350.35	334.25	244.45	243.62	245.31	-0.27	-0.26	-0.27
Ghana	45.05	58.68	31.86	55.71	62.36	49.00	0.82	0.68	1.12
Greece	220.67	191.60	250.49	277.86	271.12	284.93	0.03	0.13	-0.04
Greenland	77.96	55.90	97.05	71.34	40.82	97.80	0.09	-0.04	0.13
Grenada	141.60	140.26	142.97	111.84	114.09	109.61	-0.22	-0.20	-0.24
Guam	80.74	59.06	99.67	232.73	199.12	265.39	0.51	0.67	0.43
Guatemala	45.56	43.77	47.37	49.39	51.41	47.29	0.60	0.70	0.49
Guinea	44.04	51.37	36.55	50.50	49.63	51.37	0.90	0.67	1.21
Guinea-Bissau	57.26	62.20	52.19	60.88	63.27	58.46	0.53	0.46	0.63
Guyana	109.23	90.24	128.83	132.79	108.81	156.54	0.19	0.23	0.16
Haiti	129.66	140.11	118.89	102.20	109.45	94.80	0.28	0.24	0.32
Honduras	71.89	72.16	71.63	115.05	122.85	107.25	0.66	0.64	0.68
Hungary	379.03	349.03	411.48	350.62	377.03	321.50	0.07	0.14	-0.01
Iceland	194.97	165.43	224.25	131.03	96.18	166.62	-0.22	-0.27	-0.20
India	91.56	76.44	105.63	122.76	96.29	147.36	0.84	0.92	0.76
Indonesia	65.51	62.28	68.71	87.50	72.67	102.12	0.64	0.50	0.75
Iran	90.10	69.50	109.95	125.80	90.82	160.27	0.91	1.04	0.84
Iraq	147.26	145.54	148.96	122.76	111.66	133.57	0.02	0.04	0.00
Ireland	210.81	182.88	238.99	120.76	99.57	142.58	-0.27	-0.27	-0.28
Israel	167.75	150.75	184.81	83.05	76.71	89.49	-0.47	-0.48	-0.45
Italy	185.05	168.02	203.12	188.07	186.46	189.78	-0.11	-0.09	-0.12
Jamaica	78.60	79.54	77.63	86.07	87.76	84.36	-0.15	-0.09	-0.20
Japan	95.13	90.96	99.46	127.26	129.80	124.58	-0.18	-0.16	-0.20
Jordan	79.58	71.72	86.69	47.18	32.39	61.24	0.01	-0.12	0.08
Kazakhstan	211.46	224.41	197.71	239.71	226.76	253.59	0.13	0.10	0.18
Kenya	15.64	13.35	17.94	17.74	14.32	21.15	0.46	0.46	0.44
Kiribati	85.34	70.41	100.59	93.58	66.60	121.26	0.38	0.31	0.41
Kuwait	43.44	26.75	58.03	40.99	18.9	58.23	0.27	-0.04	0.38
Kyrgyzstan	171.83	182.86	160.33	188.30	177.19	199.64	0.42	0.42	0.47
Laos	116.52	124.65	108.38	85.25	78.23	92.35	0.79	0.64	0.97

Latvia	463.26	477.36	447.02	444.60	471.04	413.82	-0.03	-0.03	-0.03
Lebanon	190.98	140.80	242.55	141.74	119.89	163.37	0.09	0.10	0.11
Lesotho	53.05	46.34	60.15	96.29	96.54	96.05	0.03	0.10	-0.04
Liberia	35.8	39.96	31.61	38.27	43.79	32.83	1.48	1.34	1.64
Libya	59.69	56.74	62.34	87.22	79.81	94.54	0.21	0.30	0.15
Lithuania	427.29	438.80	414.49	449.09	467.24	427.97	-0.09	-0.09	-0.10
Luxembourg	223.27	206.50	240.72	110.66	108.24	113.08	-0.25	-0.25	-0.25
Macedonia	145.68	113.27	177.71	184.30	157.48	211.35	0.08	0.14	0.04
Madagascar	51.11	37.68	64.58	48.06	36.37	59.83	0.56	0.62	0.50
Malawi	34.25	39.25	29.07	32.12	30.99	33.26	0.80	0.57	1.13
Malaysia	85.82	71.08	100.13	103.22	67.16	139.94	0.11	0.03	0.14
Maldives	65.01	51.15	78.17	59.75	37.24	82.13	1.14	1.11	1.09
Mali	49.87	62.07	37.41	33.54	41.83	25.41	0.22	0.22	0.24
Malta	191.64	157.43	227.07	181.41	163.17	199.80	-0.18	-0.14	-0.20
Marshall Islands	73.25	50.86	94.66	108.77	82.46	134.17	0.46	0.51	0.46
Mauritania	49.01	52.13	45.87	34.79	39.32	30.32	0.33	0.35	0.27
Mauritius	165.00	136.00	194.11	148.11	129.10	167.60	-0.29	-0.29	-0.28
Mexico	47.68	43.79	51.61	77.98	74.29	81.72	0.57	0.53	0.60
Moldova	322.09	344.25	297.78	352.86	364.69	340.07	0.16	0.15	0.17
Mongolia	133.22	130.03	136.49	150.23	125.46	175.50	0.33	0.33	0.36
Montenegro	159.55	145.91	173.31	221.11	174.18	269.15	0.12	0.09	0.14
Morocco	66.36	66.34	66.38	87.73	88.11	87.35	0.50	0.48	0.51
Mozambique	29.5	27.89	31.25	40.35	36.81	44.06	1.09	0.98	1.14
Myanmar	48.26	48.28	48.25	51.01	44.4	57.93	0.53	0.46	0.62
Namibia	64.79	60.35	69.42	51.43	47.13	55.96	-0.10	-0.06	-0.13
Nepal	71.80	77.44	66.07	90.68	82.52	99.37	1.21	1.08	1.38
Netherlands	199.98	168.00	232.67	118.45	102.98	134.18	-0.40	-0.40	-0.39
New Zealand	212.24	181.08	244.35	144.47	131.94	157.46	-0.24	-0.22	-0.25
Nicaragua	30.49	28.26	32.75	61.59	61.58	61.60	1.37	1.55	1.20
Niger	28.21	27.46	28.99	31.6	28.89	34.26	1.40	1.28	1.51
Nigeria	30.61	34.26	27.02	21	18.9	23.01	0.27	0.08	0.55
North Korea	50.70	55.68	45.43	105.47	112.86	97.75	0.55	0.56	0.56
Northern Mariana Islands	32.77	28.2	36.88	27.68	17.37	38.07	0.18	0.08	0.23
Norway	304.53	251.13	359.14	141.56	137.80	145.27	-0.38	-0.34	-0.41
Oman	52.48	55.36	50.18	48.79	49.06	48.66	0.49	0.49	0.54
Pakistan	121.53	109.36	132.83	156.57	136.24	175.82	0.64	0.70	0.60
Palestine	65.18	53.57	76.51	75.44	50.83	99.36	0.52	0.42	0.57
Panama	70.62	62.72	78.34	75.24	66.99	83.44	-0.09	-0.09	-0.09
Papua New Guinea	71.10	57.73	83.87	98.75	88.38	108.71	0.66	0.77	0.61
Paraguay	59.38	58.41	60.34	81.86	72.53	90.92	0.07	0.04	0.10
Peru	55.79	53.81	57.77	46.94	46.7	47.18	0.28	0.26	0.30
Philippines	61.20	48.85	73.30	91.47	73.95	108.67	0.58	0.59	0.58
Poland	360.08	333.56	387.98	270.56	265.72	275.77	-0.23	-0.19	-0.26
Portugal	170.49	159.22	182.62	122.55	118.12	127.27	-0.23	-0.21	-0.25
Puerto Rico	172.34	148.51	197.66	152.80	153.19	152.39	-0.24	-0.26	-0.24
Qatar	47.95	47.37	48.23	21.81	15.79	24.06	-0.19	-0.35	-0.13
Romania	298.32	280.68	316.46	363.89	347.88	380.92	-0.01	0.00	-0.02
Russia	340.45	371.24	305.49	439.32	436.68	442.42	0.06	0.03	0.11
Rwanda	40.05	37.8	42.38	29.94	27.39	32.71	0.68	0.64	0.69
Saint Lucia	104.93	96.53	113.58	97.27	99.32	95.14	-0.13	-0.07	-0.19
Saint Vincent and the Grenadines	117.12	130.48	103.63	142.56	147.13	138.07	0.01	0.04	0.01
Samoa	76.56	58.69	93.03	101.21	108.61	94.26	0.25	0.39	0.18
Sao Tome and Principe	53.41	61.36	45.31	48.2	60.16	36.14	0.44	0.51	0.31
Saudi Arabia	48.92	47.55	50.00	49.79	36.62	59.90	0.68	0.52	0.79
Senegal	34.6	32.1	37.12	41.07	40.91	41.23	1.03	1.13	0.92
Serbia	221.53	209.00	234.41	281.67	278.67	284.80	-0.02	0.00	-0.04
Seychelles	130.70	112.01	149.47	105.85	98.65	112.86	-0.11	-0.06	-0.15
Sierra Leone	38.72	28.92	48.73	43.38	44.55	42.19	0.80	1.32	0.52
Singapore	105.88	90.98	120.38	100.27	90.59	110.30	-0.18	-0.21	-0.14
Slovakia	377.18	351.84	403.73	292.75	312.92	271.46	-0.10	-0.06	-0.16
Slovenia	176.76	172.14	181.66	172.48	186.46	158.25	-0.02	0.01	-0.07
Solomon Islands	95.89	72.84	117.71	116.39	93.04	139.01	0.40	0.42	0.41
Somalia	46.73	41.52	52.04	40.13	37.6	42.68	0.29	0.36	0.25
South Africa	77.15	95.58	58.30	83.98	92.60	75.06	-0.07	-0.14	0.06
South Korea	81.20	83.25	79.15	59.74	65.08	54.34	-0.36	-0.33	-0.39
South Sudan	35.55	33.24	37.87	41.91	43.09	40.73	0.79	0.95	0.66
Spain	164.65	153.47	176.29	124.67	122.61	126.78	-0.23	-0.23	-0.23
Sri Lanka	112.62	79.51	145.09	114.88	89.80	141.84	0.09	0.08	0.10
Sudan	115.64	105.96	125.25	100.51	89.26	111.67	0.52	0.52	0.52
Suriname	102.80	93.47	111.84	95.32	82.93	107.67	-0.15	-0.15	-0.14

Swaziland	43.27	41.16	45.63	75.96	73.69	78.28	0.11	0.09	0.11
Sweden	357.09	314.21	401.01	220.49	214.28	226.71	-0.27	-0.25	-0.29
Switzerland	213.24	197.91	229.30	145.59	140.27	151.04	-0.20	-0.21	-0.19
Syria	104.82	93.34	115.93	114.80	98.98	130.23	-0.17	0.07	-0.29
Taiwan	65.94	48.64	82.01	81.83	67.84	95.87	-0.07	0.04	-0.13
Tajikistan	143.89	146.25	141.49	109.13	97.22	120.76	0.41	0.33	0.49
Tanzania	36.54	34.21	38.91	37.74	34.57	40.95	0.73	0.71	0.71
Thailand	68.39	56.61	80.40	90.43	72.68	108.70	-0.16	-0.13	-0.18
The Bahamas	87.78	79.71	95.96	101.92	96.57	107.50	0.01	-0.02	0.03
The Gambia	34.31	35.69	32.94	29.25	26.47	32.09	0.56	0.54	0.66
Timor-Leste	43.64	42.74	44.51	58.31	60.50	56.18	1.81	1.94	1.70
Togo	41.03	42.01	40.03	43.85	35.39	52.50	0.47	0.29	0.63
Tonga	74.78	57.70	91.44	100.35	79.13	121.45	0.22	0.26	0.19
Trinidad and Tobago	147.52	133.09	162.05	168.36	146.66	190.63	-0.06	-0.04	-0.07
Tunisia	51.39	41.29	61.37	72.68	57.59	88.12	0.41	0.46	0.38
Turkey	151.03	119.77	183.06	80.04	65.92	94.66	-0.08	-0.03	-0.11
Turkmenistan	186.04	184.75	187.37	197.72	178.48	217.61	0.49	0.46	0.52
Uganda	24.29	23.82	24.77	25.98	22.43	29.53	1.07	0.90	1.20
Ukraine	448.49	498.85	389.89	721.22	757.88	677.91	0.30	0.29	0.35
United Arab Emirates	50.64	38.73	56.82	56.84	23.74	68.90	0.45	0.10	0.47
United Kingdom	308.79	274.36	345.20	153.15	132.52	174.29	-0.40	-0.42	-0.38
United States	236.27	221.24	252.07	164.46	153.49	175.80	-0.28	-0.31	-0.25
Uruguay	209.46	186.11	234.26	149.08	150.66	147.38	-0.36	-0.36	-0.36
Uzbekistan	174.28	183.24	165.12	195.74	187.87	203.88	0.31	0.28	0.36
Vanuatu	116.04	79.46	150.62	137.94	105.41	169.67	0.29	0.34	0.28
Venezuela	79.46	70.09	88.69	100.75	85.85	115.77	0.03	0.05	0.01
Vietnam	63.40	69.17	57.45	78.66	81.27	76.00	0.30	0.25	0.37
Virgin Islands, U.S.	132.40	117.04	148.66	285.85	247.42	328.10	0.01	-0.01	0.02
Yemen	121.20	131.80	110.41	104.53	105.23	103.85	0.65	0.58	0.75
Zambia	34.89	42.15	27.52	54.74	48.03	61.48	1.24	0.76	1.99
Zimbabwe	29.05	37.88	20.14	36.15	40.14	32.05	0.02	-0.12	0.28
**Ischemic stroke**									
Afghanistan	35.84	48.91	23.4	44.60	58.87	31.2	0.77	0.79	0.83
Albania	15.04	16.26	13.87	27.59	28.05	27.12	0.34	0.28	0.40
Algeria	19.45	21.72	17.23	32.03	35.19	28.91	0.79	0.76	0.84
American Samoa	17.34	14.57	19.95	11.08	12.12	10.05	-0.20	-0.17	-0.25
Andorra	40	43.45	36.37	39.24	40.57	37.85	-0.19	-0.19	-0.17
Angola	21	26.25	15.62	16.21	21.22	11.13	0.61	0.62	0.57
Antigua and Barbuda	58.24	68.59	47.17	29.56	33.37	25.4	-0.44	-0.44	-0.42
Argentina	54.97	54.79	55.16	31.5	35.76	27.06	-0.44	-0.43	-0.47
Armenia	49.13	57.82	39.91	75.23	80.06	69.67	0.16	0.06	0.31
Australia	38.73	45.09	32.34	30.18	35.17	25.18	-0.18	-0.21	-0.15
Austria	94.68	117.45	69.87	40.29	49.83	30.27	-0.52	-0.52	-0.51
Azerbaijan	23.23	26.54	19.76	24.34	23.78	24.91	0.29	0.18	0.49
Bahrain	8.18	10.23	6.67	5.45	7.26	4.34	0.15	0.18	0.16
Bangladesh	24.42	28.21	20.8	44.42	46.36	42.51	2.11	2.20	2.16
Barbados	72.05	83	60.26	54	63.34	43.84	-0.38	-0.39	-0.38
Belarus	121.03	149.48	88.81	161.62	185.55	133.87	-0.03	-0.03	0.02
Belgium	70.67	86.38	54.22	42.51	50.27	34.45	-0.37	-0.40	-0.33
Belize	23.29	25.28	21.34	15.34	14.34	16.34	-0.28	-0.28	-0.24
Benin	17.07	21.3	12.57	17.78	21.73	13.81	0.75	0.74	0.81
Bermuda	35.95	37.23	34.65	25.78	27.15	24.39	-0.15	-0.19	-0.14
Bhutan	12.98	13.93	12.11	17.51	16.44	18.42	1.42	1.51	1.42
Bolivia	22	25.56	18.46	27.11	31.64	22.6	0.87	0.82	0.93
Bosnia and Herzegovina	51.21	60.05	42.37	116.31	126.21	106.33	0.74	0.54	1.01
Botswana	16.4	20.85	11.85	23.37	28.17	18.55	-0.04	-0.02	0.02
Brazil	20.78	19.7	21.88	22.79	21.71	23.9	0.02	-0.01	0.03
Brunei	13.7	14.13	13.31	14.21	14.49	13.94	-0.06	-0.07	-0.06
Bulgaria	132.79	139.97	125.41	180.36	198.62	161.20	0.11	0.10	0.09
Burkina Faso	11.01	13.41	8.51	9.51	12.6	6.38	0.59	0.68	0.42
Burundi	35.86	44.79	26.61	16.04	19.47	12.55	0.03	0.03	0.06
Cambodia	18.4	22.7	13.79	22.42	23.8	20.96	1.37	1.20	1.82
Cameroon	19.32	23.83	14.76	21.54	25.7	17.38	0.37	0.35	0.41
Canada	37.51	42.61	32.32	34.8	40.12	29.4	-0.16	-0.20	-0.12
Cape Verde	33.9	36.77	30.72	32.91	37.04	28.66	0.24	0.30	0.21
Central African Republic	38.27	46.06	30.22	37.9	45.55	30.04	0.12	0.10	0.14
Chad	16.57	21.16	11.91	16.59	20.57	12.62	0.41	0.38	0.47
Chile	45.87	46.81	44.9	41.06	45.96	36.03	-0.18	-0.19	-0.19
China	34.32	34.4	34.24	55.08	47.39	62.32	0.81	0.84	0.84
Colombia	14.85	15.81	13.88	13.08	14.1	12.02	-0.04	-0.11	0.00

Comoros	21.66	27.67	15.68	12.94	16.57	9.38	0.26	0.27	0.29
Congo	35.34	45.12	25.5	27.09	39.85	14.35	0.13	0.10	-0.01
Costa Rica	15.77	16.54	15	14.5	15.66	13.34	-0.26	-0.25	-0.27
Cote d'Ivoire	16.97	19.54	14.59	18.56	20.67	16.52	0.43	0.42	0.43
Croatia	101.85	115.17	87.70	112.36	133.79	89.36	-0.03	-0.05	-0.04
Cuba	42.3	44.16	40.47	62.27	67.88	56.71	0.08	0.08	0.07
Cyprus	52.17	50.2	54.18	24.85	23.72	26.06	-0.36	-0.33	-0.38
Czech Republic	149.73	176.07	121.80	86.42	105.87	66.35	-0.31	-0.30	-0.33
Democratic Republic of the Congo	18.65	26.22	10.89	19.15	24.22	14.06	0.60	0.48	0.98
Denmark	64.98	74.19	55.5	40.91	45.99	35.76	-0.21	-0.23	-0.19
Djibouti	14.3	15.8	12.81	20.43	22.43	18.45	0.61	0.68	0.59
Dominica	32.53	38.94	26.07	38.16	42.82	33.43	-0.11	-0.06	-0.13
Dominican Republic	18.89	18.13	19.64	31.88	32.11	31.66	0.46	0.52	0.40
Ecuador	17.78	17.19	18.36	19.57	19.23	19.91	0.10	0.12	0.08
Egypt	28.09	32.28	23.92	23.5	26.01	21.04	0.17	0.23	0.16
El Salvador	18.58	18.29	18.87	13.88	13.96	13.8	-0.20	-0.22	-0.21
Equatorial Guinea	44.22	56.85	32.13	15.31	18.91	11.89	-0.27	-0.27	-0.23
Eritrea	15.85	21.38	10.26	17.36	24.21	10.54	0.71	0.68	0.69
Estonia	185.08	228.48	135.69	101.77	125.82	73.54	-0.41	-0.45	-0.38
Ethiopia	24.97	27.96	21.96	17.46	20.08	14.83	1.05	1.07	1.02
Federated States of Micronesia	29.46	25.52	33.24	28.57	25.99	31.03	0.09	0.12	0.08
Fiji	24.22	22.73	25.66	27.27	27.51	27.04	-0.21	-0.22	-0.22
Finland	84.99	105.98	62.72	60.65	74.01	46.88	-0.27	-0.30	-0.22
France	51.52	60.79	41.75	38.09	44.65	31.1	-0.29	-0.30	-0.27
Gabon	39.41	45.58	32.98	28.53	37.94	19.33	0.14	0.25	-0.02
Georgia	78.19	82.85	73.06	108.83	104.11	114.00	0.08	0.06	0.13
Germany	90.09	112.80	65.7	54.86	65.31	44.05	-0.38	-0.39	-0.33
Ghana	21.4	35.22	8.02	26.86	39.71	13.88	0.87	0.79	1.41
Greece	102.09	121.44	82.25	95.55	116.27	73.81	-0.23	-0.24	-0.24
Greenland	17.23	18.09	16.48	15.93	12.62	18.8	0.10	-0.08	0.29
Grenada	67.17	78.74	55.29	46.76	57.63	35.95	-0.31	-0.28	-0.35
Guam	17.91	17.97	17.86	36.96	38.38	35.58	0.08	0.06	0.07
Guatemala	8.81	9.05	8.57	11.55	12.86	10.17	0.94	1.06	0.78
Guinea	18.69	26.62	10.58	20.9	27.25	14.57	0.87	0.78	1.18
Guinea-Bissau	23.11	30.26	15.8	22.85	29.2	16.42	0.44	0.40	0.51
Guyana	52.13	50.28	54.04	38.67	36.14	41.18	-0.27	-0.27	-0.27
Haiti	52.70	69.17	35.74	42.47	54.87	29.8	0.31	0.26	0.39
Honduras	17.48	18.28	16.68	30.17	32.75	27.59	0.80	0.73	0.86
Hungary	149.10	166.50	130.27	103.17	120.72	83.82	-0.20	-0.23	-0.19
Iceland	37.69	41.9	33.51	25.53	26.21	24.83	-0.22	-0.21	-0.20
India	24.17	24.8	23.58	28.1	26.52	29.58	0.60	0.65	0.58
Indonesia	22.82	26.76	18.9	36.15	41.23	31.13	0.95	1.01	0.95
Iran	21.21	18.83	23.49	28.78	24.25	33.24	0.86	1.04	0.79
Iraq	35.48	40.72	30.35	28.18	30.91	25.51	-0.03	0.04	-0.06
Ireland	41.27	49.24	33.23	22.94	26.14	19.64	-0.29	-0.29	-0.28
Israel	32.77	35.02	30.51	21.5	24.45	18.5	-0.29	-0.28	-0.31
Italy	79.58	89.85	68.69	62.35	75.04	48.86	-0.31	-0.31	-0.33
Jamaica	60.57	72.23	48.61	58.53	67.19	49.79	-0.25	-0.24	-0.24
Japan	67.65	72.39	62.75	80.73	87.64	73.45	-0.27	-0.29	-0.26
Jordan	30.9	34.58	27.58	17.94	17.94	17.94	-0.01	0.01	0.00
Kazakhstan	77.11	95.32	57.76	74.41	77.80	70.77	-0.04	-0.11	0.13
Kenya	10.34	12.3	8.37	9.85	10.9	8.8	0.23	0.21	0.29
Kiribati	42.60	33.11	52.29	45.66	32.2	59.46	0.35	0.35	0.33
Kuwait	4.83	5.2	4.5	7.54	6.69	8.21	1.11	0.75	1.52
Kyrgyzstan	68.46	80.84	55.55	48.4	44.89	51.97	-0.08	-0.18	0.11
Laos	25.7	32.02	19.37	20.11	22.26	17.94	0.94	0.88	1.15
Latvia	198.62	243.55	146.86	187.39	234.41	132.62	-0.04	-0.05	-0.05
Lebanon	27.46	37.6	17.03	22.9	33.25	12.65	0.24	0.15	0.24
Lesotho	25.24	28.21	22.08	39.95	49.81	29.9	-0.10	-0.05	-0.18
Liberia	15.35	19.79	10.9	15.87	21.06	10.76	1.40	1.28	1.51
Libya	11.46	13.47	9.65	21.21	23.33	19.11	0.54	0.61	0.51
Lithuania	97.44	116.46	76.27	127.92	153.38	98.30	0.14	0.13	0.13
Luxembourg	86.73	103.45	69.32	28.76	35.33	22.15	-0.50	-0.51	-0.49
Macedonia	91.10	91.04	91.15	122.05	128.59	115.46	0.14	0.16	0.11
Madagascar	24.88	29.27	20.48	24	29.06	18.91	0.61	0.68	0.52
Malawi	12.32	16.17	8.34	11.91	14.19	9.63	0.87	0.77	1.16
Malaysia	16.28	16.99	15.6	18	15.83	20.2	0.02	0.03	0.06
Maldives	9.66	7.81	11.42	9.59	6.54	12.62	1.32	1.46	1.21
Mali	22.46	31	13.74	14.33	20.36	8.41	0.17	0.20	0.12
Malta	38.31	39.55	37.04	33.51	37.93	29.06	-0.24	-0.21	-0.29

Marshall Islands	21.27	17.94	24.45	23.78	21.47	26	0.10	0.13	0.10
Mauritania	18.66	25.17	12.1	14.45	20.55	8.43	0.46	0.47	0.36
Mauritius	30.08	30.35	29.81	28.26	29.53	26.97	-0.25	-0.27	-0.24
Mexico	12.34	13.02	11.65	14.68	16.14	13.21	0.14	0.12	0.15
Moldova	85.57	96.93	73.10	81.93	88.38	74.96	0.01	-0.01	0.05
Mongolia	9.79	11.91	7.61	13.03	12.43	13.65	0.59	0.46	0.93
Montenegro	27.27	34.11	20.37	42.88	45.17	40.54	0.30	0.25	0.48
Morocco	23.43	27.25	19.6	32.76	38.5	26.89	0.59	0.59	0.59
Mozambique	19.17	21.03	17.15	22.53	24.71	20.25	0.81	0.83	0.81
Myanmar	31.98	33.63	30.27	35.42	32.63	38.34	0.61	0.56	0.72
Namibia	22.14	26.48	17.62	17.11	18.16	15.99	-0.11	-0.17	-0.01
Nepal	15.62	16.69	14.54	20.89	18.69	23.23	1.37	1.24	1.55
Netherlands	46.32	54.96	37.48	33.47	39.4	27.44	-0.27	-0.30	-0.23
New Zealand	36.34	41.25	31.29	30.36	35.61	24.92	-0.07	-0.08	-0.08
Nicaragua	10.71	10.84	10.57	13.24	13.88	12.58	0.45	0.51	0.40
Niger	11.89	14.88	8.8	13.61	15.94	11.31	1.47	1.36	1.73
Nigeria	14.85	19.32	10.47	9.07	10.43	7.76	0.13	0.06	0.34
North Korea	27.08	32.88	20.94	61.53	68.76	53.98	0.69	0.62	0.87
Northern Mariana Islands	10.12	10.34	9.91	5.37	3.88	6.87	-0.25	-0.34	-0.17
Norway	72.44	82.96	61.69	35.88	44.05	27.8	-0.34	-0.36	-0.34
Oman	2.41	3.01	1.93	5.31	7.4	4.25	2.66	3.48	2.56
Pakistan	24.76	22.39	26.96	28.56	24.61	32.3	0.48	0.53	0.46
Palestine	6.68	7.69	5.69	12.53	12.57	12.5	1.49	1.49	1.67
Panama	25.7	24.86	26.52	24.38	23.48	25.28	-0.19	-0.20	-0.19
Papua New Guinea	26.27	28.94	23.73	27.88	32.79	23.17	0.27	0.32	0.21
Paraguay	16.57	17.69	15.48	17.79	18.02	17.56	-0.16	-0.14	-0.17
Peru	17.88	18.83	16.93	11.53	12.49	10.58	-0.01	-0.03	0.00
Philippines	10.8	10.67	10.94	17.34	17.27	17.4	0.70	0.70	0.70
Poland	79.26	101.50	55.87	76.88	92.5	60.07	0.00	-0.07	0.12
Portugal	148.85	164.40	132.13	76.78	86.95	65.94	-0.45	-0.44	-0.47
Puerto Rico	29.49	29.97	28.98	29	33.83	23.8	-0.16	-0.19	-0.19
Qatar	6.15	9.02	4.73	2.91	4.26	2.4	-0.15	-0.07	-0.11
Romania	111.18	123.29	98.72	153.85	167.61	139.21	0.12	0.10	0.14
Russia	180.24	229.73	124.05	191.91	226.72	151.03	-0.12	-0.13	-0.07
Rwanda	23.36	29.51	16.98	14.4	16.65	11.96	0.39	0.29	0.57
Saint Lucia	58.28	67.32	48.97	51.93	62.68	40.77	-0.16	-0.16	-0.19
Saint Vincent and the Grenadines	43.14	55.09	31.08	41.22	48.39	34.18	-0.21	-0.19	-0.17
Samoa	22.69	19.36	25.76	24.06	25.8	22.42	0.00	0.01	0.02
Sao Tome and Principe	28.86	37.16	20.41	26.81	35.33	18.23	0.49	0.47	0.48
Saudi Arabia	12.09	13.46	11.02	12.69	13.1	12.38	0.74	0.94	0.68
Senegal	15.79	19.42	12.12	19.38	25.25	13.3	1.12	1.19	0.93
Serbia	117.68	132.24	102.72	172.33	195.93	147.57	0.13	0.11	0.14
Seychelles	33.66	36.63	30.69	22.23	23.99	20.51	-0.27	-0.30	-0.24
Sierra Leone	14.01	13.77	14.25	17.41	21.57	13.18	1.00	1.38	0.62
Singapore	32.54	37.02	28.17	32.59	39.76	25.16	-0.13	-0.15	-0.16
Slovakia	76.03	85.88	65.73	66.77	76.81	56.17	0.02	-0.05	0.07
Slovenia	95.50	109.61	80.53	62.88	75.58	49.95	-0.34	-0.35	-0.34
Solomon Islands	32.03	29.02	34.87	31.23	30.42	32.02	0.13	0.18	0.10
Somalia	27.62	33.06	22.08	19.65	23.16	16.1	0.08	0.06	0.11
South Africa	30.82	42.2	19.17	33.07	42.85	22.96	-0.08	-0.10	-0.01
South Korea	61.02	69.23	52.84	54.76	62	47.44	-0.22	-0.24	-0.21
South Sudan	16.4	20.93	11.83	17.85	22.76	12.94	0.65	0.65	0.67
Spain	73.7	86.71	60.15	38.54	46.89	29.98	-0.47	-0.48	-0.46
Sri Lanka	12.93	11.34	14.48	39.1	38.16	40.1	2.23	2.22	2.12
Sudan	30.38	35.04	25.76	30.26	33	27.55	0.76	0.73	0.84
Suriname	34.81	39.06	30.71	41.28	45.9	36.67	0.09	0.12	0.06
Swaziland	17.93	21.31	14.14	27.36	33.21	21.39	-0.03	-0.04	-0.01
Sweden	73.46	84.68	61.97	52.69	63.59	41.77	-0.16	-0.17	-0.15
Switzerland	57.01	65.72	47.89	30.74	36.34	25.01	-0.37	-0.38	-0.35
Syria	26.28	26.9	25.68	23.08	23.64	22.54	-0.33	-0.11	-0.44
Taiwan	32.64	29.85	35.23	32.06	29.34	34.78	-0.26	-0.26	-0.26
Tajikistan	35.13	37.69	32.53	25.54	24.6	26.46	0.35	0.32	0.43
Tanzania	14.32	17.25	11.34	14.62	15.92	13.29	0.72	0.59	0.92
Thailand	12.23	13.93	10.5	25.34	24.86	25.83	0.33	0.22	0.51
The Bahamas	29.17	33.2	25.08	31.41	37.01	25.57	-0.07	-0.10	-0.06
The Gambia	13.78	17.81	9.82	12.16	13.98	10.31	0.62	0.63	0.78
Timor-Leste	9.88	12.22	7.6	16.15	20.03	12.4	2.50	2.53	2.56
Togo	18.1	23.47	12.6	16.88	19.17	14.54	0.29	0.26	0.45
Tonga	21.5	25.21	17.88	26.82	31.4	22.27	0.13	0.15	0.12
Trinidad and Tobago	46.51	48.97	44.02	39.6	41.04	38.13	-0.30	-0.27	-0.32

Tunisia	28.41	26	30.79	38.08	36.98	39.21	0.34	0.50	0.23
Turkey	22.87	27.75	17.86	22.98	25.28	20.6	0.75	0.61	1.00
Turkmenistan	7.49	9.27	5.65	7.85	9.06	6.6	0.48	0.50	0.55
Uganda	17.83	22	13.61	13.52	14.45	12.59	0.48	0.34	0.73
Ukraine	172.90	214.85	124.08	155.84	184.53	121.96	-0.27	-0.27	-0.24
United Arab Emirates	10.85	12.68	9.9	9.36	8.31	9.75	0.12	0.16	0.20
United Kingdom	75.67	92.25	58.13	44.46	52.47	36.25	-0.29	-0.32	-0.23
United States	41.67	49.14	33.81	34.39	40.11	28.48	-0.15	-0.19	-0.10
Uruguay	88.28	97.75	78.21	79.11	100.71	55.97	-0.19	-0.18	-0.27
Uzbekistan	37.1	39.88	34.26	37.71	36.36	39.1	0.19	0.15	0.26
Vanuatu	38.7	32.58	44.48	35.72	34.38	37.03	0.00	0.08	-0.05
Venezuela	18.8	19.87	17.75	16.92	18.47	15.36	-0.27	-0.20	-0.33
Vietnam	35.31	40.89	29.57	52.7	59.14	46.13	0.57	0.57	0.62
Virgin Islands, U.S.	20.29	20.63	19.94	43.24	43.63	42.82	0.00	-0.01	0.00
Yemen	25.2	31.99	18.29	27.13	32.64	21.74	1.09	1.09	1.22
Zambia	12.61	17.9	7.22	15.62	18.04	13.2	0.78	0.58	1.46
Zimbabwe	9.39	12.75	6	11.33	13.58	9.01	0.00	-0.10	0.22
**Other cardiovascular and circulatory diseases**									
Afghanistan	9.49	13.77	5.41	14.03	20.35	8.09	1.09	1.17	1.06
Albania	10.89	9.27	12.44	19.68	16.48	22.93	0.31	0.31	0.32
Algeria	7.33	8.56	6.13	7.69	9.37	6.04	0.15	0.20	0.09
American Samoa	5.07	3.18	6.85	5.74	5.91	5.58	0.42	0.85	0.21
Andorra	9.64	10.03	9.22	14.31	15.13	13.46	0.24	0.36	0.18
Angola	13.7	14.95	12.42	10.92	12.86	8.96	0.59	0.63	0.54
Antigua and Barbuda	7.01	7.6	6.38	14.68	14.43	14.95	1.33	1.20	1.51
Argentina	9.82	9.68	9.96	18.74	21.98	15.35	0.87	0.99	0.67
Armenia	1.09	1.16	1.02	3.34	3.62	3.01	1.31	1.37	1.21
Australia	6.98	7.28	6.68	9.51	10.24	8.76	0.43	0.42	0.43
Austria	22.32	28.33	15.78	20.4	26.64	13.85	0.03	0.07	-0.01
Azerbaijan	1.77	1.74	1.81	3.26	2.68	3.85	1.26	1.01	1.49
Bahrain	3.3	3.72	2.98	1.9	2.5	1.53	-0.01	0.11	-0.09
Bangladesh	0.42	0.48	0.37	0.72	0.69	0.74	1.89	1.80	2.11
Barbados	7.17	8.18	6.08	23.22	27.32	18.76	1.66	1.66	1.62
Belarus	2.17	2.57	1.71	2.89	3.17	2.55	-0.03	-0.03	0.00
Belgium	16.12	19.74	12.34	27.76	33.58	21.71	0.81	0.77	0.86
Belize	4.3	4.64	3.97	8.96	8.49	9.43	1.29	1.31	1.36
Benin	11.36	12.88	9.74	7.87	7.52	8.21	0.16	0.00	0.37
Bermuda	7.17	7.22	7.12	13.55	12.5	14.63	1.23	0.93	1.51
Bhutan	0.47	0.53	0.41	0.91	0.85	0.95	2.48	2.40	2.72
Bolivia	12.68	13.36	12.01	11.01	11.81	10.22	0.33	0.31	0.36
Bosnia and Herzegovina	19.14	19.09	19.19	14.1	11.67	16.55	-0.43	-0.55	-0.31
Botswana	3.26	3.93	2.58	4.29	4.71	3.87	-0.13	-0.15	-0.05
Brazil	5.96	5.73	6.19	8.96	9.46	8.45	0.39	0.48	0.29
Brunei	2.85	3	2.71	3.56	3.55	3.56	0.13	0.08	0.19
Bulgaria	26.2	21.91	30.6	39.13	34.24	44.26	0.22	0.21	0.23
Burkina Faso	14.56	17.7	11.27	10.24	12.1	8.36	0.29	0.21	0.39
Burundi	35.63	38.12	33.05	13.99	14.36	13.63	-0.09	-0.09	-0.05
Cambodia	6.9	7.28	6.5	5.1	4.95	5.27	0.43	0.40	0.51
Cameroon	14.54	17.14	11.91	9.47	9.95	8.99	-0.20	-0.27	-0.11
Canada	6.97	7.05	6.9	9.79	10.55	9.02	0.28	0.27	0.27
Cape Verde	18.78	16.73	21.04	10.95	12.42	9.43	-0.25	-0.04	-0.42
Central African Republic	18.01	19.73	16.23	18.96	20.4	17.48	0.19	0.13	0.22
Chad	10.72	13.72	7.68	8.11	9.25	6.98	0.07	-0.05	0.26
Chile	2.42	2.42	2.42	6.6	7.77	5.39	1.50	1.65	1.25
China	12.48	13.32	11.68	5.02	4.35	5.65	-0.55	-0.57	-0.51
Colombia	5.26	5.19	5.32	6.07	6.56	5.57	0.26	0.27	0.21
Comoros	24.53	25	24.06	12.99	13.27	12.71	0.11	0.13	0.13
Congo	16.3	18.24	14.34	13.24	17.64	8.85	0.19	0.20	0.09
Costa Rica	1.75	1.88	1.63	5.79	6.06	5.53	1.66	1.54	1.77
Cote d'Ivoire	12.19	12.92	11.52	8.57	8.36	8.77	-0.08	-0.13	-0.04
Croatia	34.48	36.46	32.37	14.27	15.65	12.8	-0.64	-0.65	-0.63
Cuba	2.06	2.42	1.7	11.08	10.69	11.45	2.97	2.12	4.15
Cyprus	17.34	17.41	17.27	11.31	11.37	11.24	-0.12	-0.04	-0.14
Czech Republic	21.65	23.47	19.72	24.76	27.61	21.82	0.37	0.37	0.37
Democratic Republic of the Congo	8.7	10.71	6.65	9.39	10.74	8.02	0.66	0.59	0.85
Denmark	27.1	29.1	25.05	18.18	20.34	15.99	-0.16	-0.13	-0.19
Djibouti	18.68	16.29	21.04	22.45	18.68	26.19	0.34	0.34	0.36
Dominica	5.48	6.79	4.15	18.32	20.4	16.21	1.53	1.57	1.66
Dominican Republic	3.47	3.54	3.4	6.87	7.25	6.48	0.71	0.76	0.65
Ecuador	2.9	2.87	2.93	4.89	4.81	4.97	0.68	0.69	0.68

Egypt	18.75	18.9	18.6	16.19	15.16	17.18	0.21	0.22	0.22
El Salvador	3.59	3.54	3.64	5.27	5.97	4.48	0.56	0.72	0.33
Equatorial Guinea	20.12	21.9	18.42	11.54	12.31	10.8	0.18	0.17	0.20
Eritrea	19.25	21.67	16.8	17.64	21.36	13.94	0.43	0.44	0.36
Estonia	2.87	3.23	2.46	12.8	14.86	10.37	3.77	3.64	3.82
Ethiopia	27.69	28.33	27.05	13.82	14.04	13.61	0.46	0.42	0.50
Federated States of Micronesia	8.5	7.81	9.17	10.35	11.1	9.63	0.37	0.57	0.22
Fiji	9.58	8.78	10.35	21.87	22.39	21.36	0.59	0.65	0.53
Finland	7.58	9.38	5.67	18.98	22.9	14.94	1.58	1.47	1.77
France	32.15	35	29.14	32.97	36.21	29.52	-0.01	-0.02	0.00
Gabon	19.95	19.88	20.01	14.6	16.67	12.58	0.15	0.26	0.04
Georgia	1.47	1.73	1.19	12.78	9.55	16.31	5.72	3.65	8.86
Germany	11.27	13.74	8.61	31.84	38.2	25.27	1.90	1.95	1.91
Ghana	4.16	2.56	5.71	3.77	2.65	4.91	0.34	0.65	0.18
Greece	10.46	12.39	8.49	9.21	10.67	7.67	-0.28	-0.31	-0.23
Greenland	9.29	7.6	10.76	11.42	8.88	13.61	0.47	0.54	0.43
Grenada	6.95	7.43	6.45	15.7	16.51	14.89	1.24	1.19	1.31
Guam	3.74	3.73	3.76	10.99	11.86	10.15	0.54	0.58	0.45
Guatemala	2.3	2.26	2.34	2.87	3.05	2.69	0.85	0.96	0.72
Guinea	11.85	15.85	7.75	7.9	8.89	6.91	0.11	-0.02	0.42
Guinea-Bissau	16.4	20.21	12.5	11.17	12.4	9.93	-0.03	-0.12	0.14
Guyana	4.7	4.63	4.77	10.39	9.92	10.86	1.16	1.19	1.17
Haiti	8.62	10.88	6.3	9.43	11.47	7.34	0.79	0.69	0.97
Honduras	1.76	1.91	1.6	2.52	2.79	2.25	0.50	0.42	0.58
Hungary	28.87	30.56	27.05	20.94	22.91	18.76	-0.16	-0.20	-0.12
Iceland	8.57	9.93	7.22	9.91	10.33	9.48	0.34	0.32	0.41
India	0.64	0.71	0.58	1.38	1.31	1.46	1.96	1.81	2.16
Indonesia	4.11	4.06	4.15	4.66	4.6	4.73	0.40	0.47	0.36
Iran	20.00	16.53	23.35	15.46	12.02	18.85	0.06	0.15	0.02
Iraq	15.99	18.1	13.92	12.89	14.02	11.79	-0.01	0.06	-0.04
Ireland	9.24	10.28	8.19	8.91	9.5	8.3	0.22	0.24	0.23
Israel	3.09	3.73	2.44	11.42	14.29	8.5	2.99	2.96	2.93
Italy	9.37	10.43	8.23	16.94	20.14	13.54	0.59	0.59	0.56
Jamaica	4.29	4.88	3.68	10.79	12.54	9.02	0.96	1.12	0.81
Japan	4.81	5.02	4.58	12.5	15.33	9.52	0.60	0.80	0.32
Jordan	4.42	5.49	3.45	2.83	2.98	2.7	0.10	0.07	0.21
Kazakhstan	2.46	2.49	2.43	9.3	6.03	12.8	2.75	1.64	3.84
Kenya	10.21	11.03	9.38	10.21	9.94	10.48	0.29	0.23	0.37
Kiribati	7.9	6.71	9.12	13.52	14.58	12.44	1.16	2.03	0.60
Kuwait	1.1	1.28	0.96	1.97	2.05	1.9	1.41	1.17	1.75
Kyrgyzstan	1.73	2.1	1.35	2.91	2.54	3.29	1.19	0.78	1.89
Laos	9.19	9.86	8.51	4.93	4.67	5.2	0.32	0.26	0.43
Latvia	2.74	3.39	1.99	8.17	7.98	8.4	2.03	1.33	3.43
Lebanon	7.51	8.2	6.8	7.13	8.95	5.32	0.41	0.43	0.31
Lesotho	4.22	4.75	3.66	6.85	7.99	5.69	-0.08	-0.10	-0.06
Liberia	11.82	14.16	9.47	6.26	7.02	5.52	0.23	0.06	0.46
Libya	6.97	7.59	6.42	7.09	7.86	6.32	-0.14	-0.02	-0.23
Lithuania	2.9	3.27	2.5	12.6	11.52	13.85	2.77	2.04	3.85
Luxembourg	32.58	37.67	27.28	24.49	29.69	19.28	0.14	0.13	0.13
Macedonia	14.51	12.74	16.26	9.15	7.65	10.66	-0.46	-0.51	-0.42
Madagascar	29.55	29.5	29.60	25.39	25.04	25.75	0.43	0.43	0.42
Malawi	13.89	16.2	11.5	11.04	10.99	11.09	0.53	0.36	0.80
Malaysia	8.33	8.21	8.44	7.31	6.56	8.07	-0.19	-0.11	-0.21
Maldives	8.31	8.11	8.5	3.71	2.74	4.67	0.05	0.00	0.10
Mali	14.96	20.27	9.52	6.77	8.71	4.86	-0.17	-0.22	-0.06
Malta	9.93	10.22	9.63	11.78	13	10.55	0.03	0.05	0.00
Marshall Islands	7.76	6.79	8.68	13.24	15.43	11.12	0.69	1.14	0.34
Mauritania	11.61	14.83	8.38	5.37	6.62	4.13	-0.13	-0.19	-0.04
Mauritius	3.8	2.88	4.73	5.22	4.86	5.6	0.09	0.26	-0.01
Mexico	3.36	3.32	3.41	4.12	4.25	3.99	0.18	0.16	0.18
Moldova	1.91	1.91	1.91	3.55	3.13	4	0.96	0.78	1.15
Mongolia	8.02	5.99	10.09	3.59	2.84	4.35	-0.47	-0.34	-0.54
Montenegro	7.66	8.38	6.92	9.17	9.38	8.95	-0.01	0.06	-0.03
Morocco	14.75	15.28	14.23	10.7	11.33	10.06	-0.17	-0.16	-0.18
Mozambique	15.7	15.8	15.6	18.5	18.01	19.02	0.80	0.73	0.87
Myanmar	8.73	10.79	6.59	8.04	9.47	6.53	0.34	0.41	0.34
Namibia	4.16	4.68	3.62	2.96	2.9	3.02	-0.18	-0.24	-0.09
Nepal	0.44	0.47	0.4	0.71	0.65	0.77	1.88	1.76	2.09
Netherlands	16.51	18.84	14.14	21.34	23.72	18.92	0.31	0.23	0.41
New Zealand	9.22	9.42	9.03	12.19	12.8	11.55	0.47	0.45	0.49

Nicaragua	1.62	1.71	1.53	2.74	3.08	2.38	0.98	1.11	0.82
Niger	9.05	10.61	7.45	5.05	5.54	4.56	0.20	0.14	0.30
Nigeria	10.17	12.71	7.68	4.59	4.7	4.47	-0.16	-0.27	0.05
North Korea	7.81	9.74	5.77	8.84	10.84	6.75	-0.15	-0.13	-0.15
Northern Mariana Islands	3.38	3.75	3.04	3.28	3.05	3.52	0.37	0.44	0.38
Norway	19.86	22.7	16.96	17.74	22.53	13	0.19	0.21	0.13
Oman	41.70	45.07	39.01	18.21	18.05	18.29	-0.30	-0.33	-0.26
Pakistan	0.67	0.53	0.81	1.27	1.06	1.47	1.43	1.80	1.23
Palestine	4.6	4.93	4.28	5.2	5.25	5.15	0.49	0.61	0.46
Panama	2.01	2	2.02	5.26	5.16	5.35	1.22	1.19	1.26
Papua New Guinea	7.81	8.66	6.99	8.75	10.3	7.25	0.34	0.38	0.29
Paraguay	2.35	2.51	2.2	5.51	5.75	5.28	0.82	0.93	0.76
Peru	1.99	1.85	2.12	4.03	4.25	3.81	2.10	2.33	1.88
Philippines	0.9	0.89	0.91	2.39	2.23	2.54	1.81	1.64	1.98
Poland	18.41	15.41	21.56	12.65	11.97	13.39	-0.29	-0.21	-0.35
Portugal	11.64	12.27	10.97	16.84	19.34	14.17	0.54	0.68	0.38
Puerto Rico	6.07	6.7	5.41	11.89	12.13	11.63	0.67	0.31	1.12
Qatar	2.64	3.46	2.24	1.37	1.8	1.2	-0.07	0.03	-0.06
Romania	18.34	13.81	22.99	15.57	9.74	21.79	-0.31	-0.43	-0.23
Russia	2.74	2.89	2.56	9.29	8.91	9.73	1.79	1.71	1.92
Rwanda	21.88	25.78	17.85	12.24	13	11.43	0.27	0.16	0.42
Saint Lucia	7.17	8.05	6.27	18.93	21.05	16.73	1.47	1.38	1.58
Saint Vincent and the Grenadines	7.28	9.04	5.51	19.22	19.2	19.24	1.19	0.96	1.64
Samoa	6.28	5.23	7.25	10.51	13.28	7.9	0.60	0.94	0.29
Sao Tome and Principe	15.47	21.78	9.05	13.29	19.06	7.47	0.38	0.36	0.36
Saudi Arabia	3.01	3.54	2.6	3.7	4.2	3.32	1.04	1.37	0.90
Senegal	10.09	12.21	7.95	7.16	8.39	5.89	0.22	0.15	0.29
Serbia	11.09	10.58	11.61	19.91	18.84	21.03	0.38	0.33	0.44
Seychelles	14.26	12.64	15.89	11.37	10.81	11.92	-0.12	-0.07	-0.14
Sierra Leone	10.01	9.84	10.2	7.46	8.31	6.59	0.19	0.25	0.12
Singapore	1.78	2	1.57	3.14	3.5	2.76	0.53	0.39	0.66
Slovakia	26.15	24.69	27.68	16.27	17.68	14.78	-0.28	-0.24	-0.33
Slovenia	13.95	15.24	12.58	19.49	24.05	14.84	0.40	0.48	0.26
Solomon Islands	8.48	7.84	9.08	10.93	11.41	10.45	0.49	0.63	0.38
Somalia	28.09	31.87	24.23	19.72	22.31	17.1	0.04	0.04	0.06
South Africa	5.13	6.4	3.83	6.55	7.95	5.11	0.09	0.10	0.10
South Korea	1.84	2.04	1.64	3.63	4.31	2.95	0.72	0.80	0.59
South Sudan	22.26	25.21	19.29	19.38	21.61	17.15	0.30	0.28	0.33
Spain	14.9	16.33	13.4	18.9	22.74	14.96	0.29	0.34	0.20
Sri Lanka	18.19	12.68	23.59	4.97	3.72	6.32	-0.71	-0.72	-0.70
Sudan	9.18	10.69	7.68	8.41	9.55	7.28	0.62	0.63	0.64
Suriname	3.92	4.3	3.55	9.27	9.5	9.04	1.18	1.11	1.27
Swaziland	4.12	4.68	3.49	5.99	6.76	5.21	-0.08	-0.12	-0.03
Sweden	22.93	25.77	20.02	20.3	23.39	17.21	0.04	0.00	0.08
Switzerland	33.97	37.4	30.37	17	20.2	13.73	-0.41	-0.40	-0.44
Syria	2.94	2.92	2.97	2.96	3	2.92	-0.23	0.05	-0.37
Taiwan	6.39	6.27	6.5	6.04	6.04	6.03	-0.28	-0.26	-0.30
Tajikistan	0.92	1.04	0.81	0.94	0.86	1.02	0.88	0.65	1.20
Tanzania	14.61	15.8	13.39	13.07	12.68	13.47	0.51	0.38	0.64
Thailand	6.8	5.34	8.28	12.98	12.1	13.89	0.22	0.54	0.02
The Bahamas	5.46	5.91	4.99	14.85	17.01	12.61	1.36	1.34	1.32
The Gambia	8.28	10.34	6.26	4.78	4.9	4.65	0.05	-0.02	0.25
Timor-Leste	4.53	5.06	4.02	3.56	4.23	2.92	0.69	0.77	0.60
Togo	11.09	13.5	8.61	7.17	6.82	7.52	-0.10	-0.22	0.09
Tonga	1.06	1.15	0.99	1.91	2.32	1.51	0.65	0.92	0.40
Trinidad and Tobago	2.2	2.61	1.79	10.06	10.89	9.2	2.77	2.64	3.05
Tunisia	8.75	8.98	8.53	10.05	9.77	10.33	0.15	0.15	0.17
Turkey	9.64	10.4	8.86	6.34	6.88	5.79	0.15	0.19	0.13
Turkmenistan	1.28	1.37	1.18	1.4	1.36	1.45	0.54	0.51	0.61
Uganda	18.26	20.84	15.66	15.16	14.61	15.71	0.61	0.42	0.86
Ukraine	2.14	2.39	1.84	3.15	3.28	3	0.20	0.16	0.26
United Arab Emirates	0.84	0.81	0.85	1	0.69	1.11	0.60	0.61	0.66
United Kingdom	16.09	18.63	13.41	20.02	22.79	17.18	0.51	0.46	0.57
United States	11.07	11.63	10.5	11.96	13.09	10.79	0.11	0.12	0.10
Uruguay	7.8	7.86	7.73	24.83	30.74	18.51	1.89	2.12	1.46
Uzbekistan	0.67	0.77	0.57	1.22	1.17	1.26	1.11	0.89	1.43
Vanuatu	7.85	6.26	9.34	9.05	8.35	9.74	0.26	0.37	0.20
Venezuela	1.79	1.85	1.73	3.92	4.21	3.62	0.78	0.95	0.63
Vietnam	4.96	5.26	4.65	6.03	6.33	5.72	0.28	0.33	0.28
Virgin Islands, U.S.	6.15	5.97	6.34	25.77	25.13	26.47	0.96	0.98	0.94

Yemen	10.13	12.64	7.58	8.82	10.96	6.73	0.67	0.72	0.68
Zambia	15.58	19.47	11.61	17.99	16.43	19.56	0.65	0.31	1.24
Zimbabwe	4.47	5.32	3.62	5.58	5.51	5.66	0.03	-0.12	0.26
**Peripheral artery disease**									
Afghanistan	0.01	0.01	0.01	0.02	0.01	0.02	1.59	1.94	1.48
Albania	0.02	0.02	0.03	0.1	0.08	0.12	2.33	2.55	2.24
Algeria	0.02	0.01	0.02	0.05	0.04	0.06	2.38	2.50	2.40
American Samoa	0.06	0.02	0.1	0.36	0.05	0.67	6.67	2.23	9.03
Andorra	2.68	2.13	3.25	5.08	4.52	5.66	0.64	1.01	0.54
Angola	0.05	0.06	0.04	0.09	0.11	0.08	2.98	2.91	3.46
Antigua and Barbuda	0.83	0.83	0.83	1.47	1.41	1.53	0.99	1.03	0.99
Argentina	0.27	0.18	0.36	0.43	0.44	0.43	0.60	1.18	0.31
Armenia	0.03	0.03	0.02	0.14	0.16	0.12	2.77	2.67	2.95
Australia	2.41	2.47	2.35	3.95	4.12	3.78	0.73	0.70	0.76
Austria	2.15	2.04	2.28	6.68	7.24	6.09	2.53	3.10	2.02
Azerbaijan	0.02	0.02	0.02	0.06	0.06	0.06	2.95	2.63	3.76
Bahrain	0.17	0.06	0.25	0.44	0.35	0.5	3.69	9.49	2.73
Bangladesh	0.02	0.01	0.02	0.05	0.05	0.06	3.98	5.26	3.33
Barbados	4.04	5.63	2.34	7.71	10.84	4.32	0.62	0.64	0.60
Belarus	0.14	0.14	0.13	1.34	1.11	1.61	6.43	5.71	7.56
Belgium	0.38	0.38	0.39	2.1	2.08	2.13	4.85	5.05	4.79
Belize	0.17	0.12	0.22	0.22	0.19	0.26	0.50	1.12	0.22
Benin	0.01	0.02	0.01	0.02	0.03	0.01	1.45	2.14	0.52
Bermuda	1.51	1.47	1.56	3.07	3.72	2.41	1.42	1.93	0.92
Bhutan	0.03	0.03	0.03	0.13	0.12	0.13	7.14	9.48	5.86
Bolivia	0.02	0.02	0.02	0.07	0.08	0.07	4.01	4.97	3.38
Bosnia and Herzegovina	0.07	0.05	0.1	0.24	0.19	0.29	1.85	2.55	1.77
Botswana	0.17	0.14	0.21	0.55	0.45	0.66	1.16	1.38	1.01
Brazil	0.32	0.28	0.37	1.25	1.2	1.3	2.56	2.86	2.32
Brunei	0.07	0.02	0.11	0.14	0.04	0.23	0.94	0.95	1.00
Bulgaria	0.06	0.04	0.07	0.41	0.34	0.48	4.95	5.21	4.95
Burkina Faso	0.02	0.02	0.01	0.03	0.05	0.02	2.80	4.18	1.02
Burundi	0.09	0.08	0.1	0.04	0.05	0.04	0.14	0.44	-0.02
Cambodia	0.01	0.01	0.01	0.03	0.03	0.04	5.03	5.47	5.09
Cameroon	0.03	0.03	0.03	0.04	0.06	0.03	0.96	1.69	0.26
Canada	1.8	1.61	2	2.87	3.12	2.62	0.45	0.67	0.28
Cape Verde	0.07	0.11	0.02	0.2	0.34	0.05	3.32	3.49	3.49
Central African Republic	0.1	0.12	0.08	0.11	0.14	0.09	0.36	0.38	0.34
Chad	0.01	0.02	0.01	0.02	0.03	0.01	1.34	1.93	0.43
Chile	0.37	0.3	0.43	2.21	2.16	2.25	4.53	5.02	4.28
China	0.03	0.03	0.04	0.13	0.11	0.15	3.55	4.12	3.32
Colombia	0.11	0.1	0.11	0.24	0.26	0.21	1.49	1.59	1.32
Comoros	0.09	0.11	0.07	0.07	0.09	0.05	0.68	0.86	0.52
Congo	0.13	0.15	0.11	0.16	0.22	0.11	0.82	0.86	0.61
Costa Rica	0.14	0.12	0.17	0.45	0.41	0.48	1.53	1.86	1.36
Cote d'Ivoire	0.02	0.02	0.02	0.03	0.04	0.02	0.81	1.50	0.17
Croatia	0.09	0.08	0.1	5.49	5.74	5.22	56.06	65.44	49.32
Cuba	1.16	1.18	1.14	5	4.96	5.03	2.31	2.29	2.41
Cyprus	1.05	1.08	1.02	0.94	0.96	0.91	0.23	0.37	0.20
Czech Republic	0.22	0.18	0.26	2.33	2.29	2.37	12.04	14.40	10.55
Democratic Republic of the Congo	0.06	0.07	0.04	0.05	0.07	0.04	0.56	0.64	0.58
Denmark	4.31	4.74	3.87	3.66	4.38	2.92	0.07	0.17	-0.03
Djibouti	0.06	0.08	0.05	0.15	0.16	0.14	1.79	1.76	2.20
Dominica	0.5	0.36	0.65	1.75	1.53	1.96	1.69	2.92	1.14
Dominican Republic	0.06	0.07	0.06	0.2	0.21	0.19	1.77	1.65	1.95
Ecuador	0.02	0.02	0.03	0.09	0.09	0.1	2.95	3.88	2.40
Egypt	0.03	0.02	0.03	0.06	0.06	0.06	2.33	3.18	1.87
El Salvador	0.02	0.02	0.02	0.07	0.06	0.08	2.80	2.93	2.76
Equatorial Guinea	0.1	0.12	0.09	0.28	0.27	0.28	5.03	4.69	6.09
Eritrea	0.03	0.04	0.02	0.05	0.07	0.03	1.49	1.56	1.04
Estonia	0.05	0.06	0.04	1.69	1.79	1.57	35.85	32.71	41.24
Ethiopia	0.05	0.05	0.05	0.06	0.07	0.05	2.76	3.36	2.50
Federated States of Micronesia	0.05	0.02	0.07	0.17	0.13	0.21	3.10	5.05	2.55
Fiji	0.02	0.01	0.02	0.11	0.15	0.07	3.78	7.27	1.61
Finland	2.51	2.84	2.17	6.78	8.17	5.34	1.81	2.00	1.63
France	0.37	0.44	0.29	1.56	1.65	1.47	3.14	2.66	3.94
Gabon	0.37	0.34	0.4	0.45	0.54	0.37	1.00	1.46	0.62
Georgia	0.06	0.06	0.06	0.61	0.5	0.73	6.77	6.19	7.53
Germany	0.99	1.08	0.88	8.17	8.25	8.08	7.77	7.66	8.22
Ghana	0.02	0.03	0.01	0.04	0.04	0.03	1.68	1.55	2.31

Greece	0.45	0.48	0.43	0.4	0.42	0.38	-0.27	-0.28	-0.24
Greenland	0.78	0.7	0.85	1.72	1.5	1.91	1.65	1.90	1.57
Grenada	0.95	0.81	1.09	1.86	2.02	1.71	1.07	1.71	0.71
Guam	0.04	0.02	0.05	1.45	0.74	2.15	20.36	15.34	23.84
Guatemala	0.02	0.01	0.03	0.05	0.05	0.05	2.61	4.71	1.74
Guinea	0.01	0.02	0.01	0.02	0.03	0.01	1.39	1.99	0.52
Guinea-Bissau	0.02	0.02	0.02	0.03	0.04	0.01	0.85	1.46	0.07
Guyana	0.18	0.03	0.32	0.36	0.32	0.39	1.00	8.97	0.16
Haiti	0.12	0.1	0.13	0.19	0.2	0.18	1.73	2.19	1.39
Honduras	0.02	0.02	0.03	0.09	0.09	0.1	3.78	4.65	3.46
Hungary	0.52	0.27	0.79	3.47	3.08	3.9	6.93	12.46	5.30
Iceland	0.52	0.74	0.31	0.94	1.22	0.65	1.12	1.16	1.28
India	0.04	0.03	0.05	0.13	0.1	0.16	3.45	3.88	3.23
Indonesia	0.02	0.01	0.02	0.05	0.05	0.06	3.08	3.74	2.64
Iran	0.02	0.01	0.02	0.06	0.04	0.08	4.06	5.73	3.62
Iraq	0.04	0.03	0.05	0.04	0.03	0.05	0.30	0.64	0.24
Ireland	3.27	2.85	3.69	2.47	2.5	2.44	-0.04	0.18	-0.19
Israel	1.05	0.83	1.28	3.13	3.15	3.11	2.24	3.01	1.79
Italy	1.41	1	1.85	3.37	3.57	3.16	1.11	1.95	0.62
Jamaica	1.5	1.37	1.64	3.48	3.72	3.25	0.86	1.36	0.53
Japan	0.06	0.05	0.08	0.9	0.99	0.81	7.97	11.33	5.75
Jordan	0.02	0.02	0.02	0.03	0.03	0.04	2.13	2.44	2.00
Kazakhstan	0.26	0.25	0.28	0.36	0.28	0.46	0.41	0.33	0.53
Kenya	0.05	0.06	0.03	0.07	0.08	0.06	1.13	1.16	1.13
Kiribati	0.03	0.03	0.03	0.12	0.09	0.15	4.59	4.14	5.23
Kuwait	0.01	0	0.01	0.02	0.01	0.02	1.94	2.57	1.71
Kyrgyzstan	0.26	0.25	0.28	0.25	0.16	0.34	0.23	-0.04	0.44
Laos	0.02	0.02	0.02	0.04	0.03	0.05	4.63	5.34	4.67
Latvia	0.09	0.09	0.09	2.34	2.41	2.25	25.80	27.42	25.07
Lebanon	0.05	0.03	0.06	0.1	0.09	0.11	2.03	2.44	1.96
Lesotho	0.25	0.18	0.32	0.62	0.58	0.65	0.48	0.90	0.27
Liberia	0.02	0.02	0.02	0.02	0.03	0.01	1.34	2.07	0.41
Libya	0.01	0.01	0.02	0.05	0.04	0.07	2.34	2.70	2.21
Lithuania	0.14	0.16	0.13	3.25	2.35	4.28	19.61	13.16	28.75
Luxembourg	0.84	0.75	0.93	1.36	1.45	1.27	1.47	1.83	1.21
Macedonia	0.04	0.03	0.05	0.15	0.12	0.18	2.47	2.71	2.39
Madagascar	0.09	0.1	0.09	0.1	0.11	0.09	0.85	1.00	0.69
Malawi	0.03	0.04	0.02	0.05	0.06	0.04	2.29	2.40	2.30
Malaysia	0.02	0.02	0.03	0.07	0.04	0.1	1.97	2.00	1.94
Maldives	0.05	0.08	0.02	0.08	0.1	0.06	2.88	3.10	4.33
Mali	0.02	0.02	0.01	0.02	0.04	0.01	1.31	2.01	0.15
Malta	0.96	0.81	1.12	2.91	3.14	2.68	1.65	2.28	1.19
Marshall Islands	0.04	0.02	0.07	0.35	0.27	0.42	6.65	10.34	5.42
Mauritania	0.02	0.03	0.01	0.03	0.05	0.01	1.33	1.80	0.32
Mauritius	0.05	0.03	0.07	0.26	0.23	0.3	3.26	5.04	2.60
Mexico	0.27	0.29	0.25	0.39	0.44	0.33	0.37	0.38	0.32
Moldova	0.05	0.04	0.05	1.05	0.76	1.36	23.76	20.49	26.67
Mongolia	0.06	0.03	0.09	0.11	0.05	0.17	1.40	1.65	1.26
Montenegro	0.15	0.16	0.14	0.29	0.27	0.31	0.64	0.66	0.73
Morocco	0.04	0.03	0.06	0.09	0.06	0.11	1.36	1.51	1.35
Mozambique	0.03	0.04	0.03	0.07	0.08	0.07	2.91	2.85	3.36
Myanmar	0.02	0.02	0.01	0.06	0.07	0.05	4.88	5.32	5.02
Namibia	0.23	0.17	0.29	0.36	0.29	0.43	0.80	1.05	0.62
Nepal	0.02	0.02	0.03	0.07	0.05	0.09	4.82	4.72	4.85
Netherlands	0.65	0.6	0.7	4.38	4.5	4.26	5.95	6.58	5.50
New Zealand	2.07	2	2.15	2.42	2.88	1.94	0.31	0.56	0.05
Nicaragua	0.02	0.01	0.02	0.07	0.06	0.07	3.48	5.49	2.47
Niger	0.01	0.01	0.01	0.01	0.02	0.01	2.22	3.02	1.06
Nigeria	0.02	0.02	0.02	0.03	0.04	0.02	1.57	2.42	0.79
North Korea	0.03	0.03	0.02	0.06	0.08	0.04	0.70	0.75	0.66
Northern Mariana Islands	0.07	0.07	0.07	0.47	0.55	0.39	8.52	12.82	5.93
Norway	0.23	0.13	0.33	2.28	2.42	2.14	12.55	23.29	8.63
Oman	0.02	0.01	0.03	0.06	0.03	0.08	3.99	4.32	3.59
Pakistan	0.05	0.03	0.06	0.1	0.08	0.13	2.05	3.13	1.66
Palestine	0.01	0.01	0.01	0.02	0.02	0.02	2.02	2.16	2.01
Panama	0.61	0.5	0.72	0.94	0.91	0.97	0.32	0.59	0.17
Papua New Guinea	0.02	0.01	0.03	0.06	0.04	0.09	2.60	2.91	2.61
Paraguay	0.03	0.03	0.03	0.25	0.23	0.26	5.22	4.88	5.80
Peru	0.04	0.04	0.04	0.09	0.1	0.09	2.83	2.95	2.90
Philippines	0.03	0.02	0.04	0.08	0.05	0.11	2.29	2.22	2.35

Poland	2.94	2.04	3.88	4.2	3.74	4.69	0.49	0.97	0.29
Portugal	1.54	1.39	1.7	4.9	4.81	5.01	2.41	2.73	2.16
Puerto Rico	0.97	1.05	0.89	2.96	3.43	2.46	1.63	1.41	1.76
Qatar	0.07	0.05	0.09	0.05	0.02	0.06	0.17	-0.15	0.20
Romania	1.11	0.57	1.67	2.1	1.22	3.04	0.54	0.74	0.49
Russia	0.05	0.06	0.04	4.51	4.21	4.86	89.37	82.21	102.44
Rwanda	0.06	0.06	0.05	0.08	0.08	0.07	2.20	2.37	2.17
Saint Lucia	0.98	1.12	0.84	2.71	3.58	1.8	1.63	2.01	1.10
Saint Vincent and the Grenadines	0.57	1.1	0.04	0.91	0.91	0.91	0.36	-0.21	15.17
Samoa	0.05	0.02	0.07	0.35	0.3	0.4	5.89	9.22	5.48
Sao Tome and Principe	0.04	0.06	0.03	0.07	0.1	0.04	1.63	2.18	0.91
Saudi Arabia	0.01	0.01	0.01	0.03	0.02	0.03	5.47	6.77	4.92
Senegal	0.01	0.02	0.01	0.02	0.04	0.01	1.94	2.72	0.61
Serbia	0.16	0.1	0.21	0.5	0.47	0.54	1.52	2.65	1.05
Seychelles	0.08	0.07	0.08	0.18	0.19	0.16	1.54	1.77	1.33
Sierra Leone	0.01	0.01	0.01	0.02	0.03	0.01	1.87	3.00	0.73
Singapore	0.09	0.12	0.07	1.43	1.69	1.15	12.87	10.97	15.46
Slovakia	0.13	0.09	0.18	1.05	1.04	1.06	8.25	11.89	6.58
Slovenia	0.09	0.05	0.13	0.37	0.39	0.35	3.23	6.83	1.89
Solomon Islands	0.02	0.01	0.04	0.1	0.05	0.14	3.80	4.39	3.81
Somalia	0.08	0.1	0.05	0.06	0.08	0.04	0.18	0.20	0.20
South Africa	0.86	0.75	0.98	1.78	1.5	2.07	0.77	0.79	0.76
South Korea	0.17	0.06	0.27	0.32	0.25	0.39	0.68	2.42	0.29
South Sudan	0.06	0.07	0.05	0.08	0.09	0.07	1.03	1.06	1.12
Spain	0.94	0.81	1.07	3.29	3.32	3.26	2.59	3.01	2.29
Sri Lanka	0.05	0.03	0.07	0.13	0.09	0.17	1.58	1.64	1.64
Sudan	0.01	0.01	0.01	0.02	0.01	0.02	2.54	2.68	2.53
Suriname	0.08	0.03	0.13	0.15	0.1	0.21	0.71	1.98	0.42
Swaziland	0.24	0.14	0.35	0.57	0.46	0.67	0.53	0.97	0.27
Sweden	1.83	2.11	1.54	3	3.53	2.46	0.94	0.87	1.02
Switzerland	2.6	2.45	2.75	2.42	2.31	2.53	0.11	0.09	0.15
Syria	0.03	0.02	0.04	0.06	0.04	0.07	0.65	1.30	0.39
Taiwan	0.06	0.05	0.07	0.15	0.13	0.18	1.12	1.47	1.11
Tajikistan	0.03	0.03	0.03	0.04	0.04	0.05	1.80	1.65	1.89
Tanzania	0.07	0.07	0.07	0.1	0.1	0.1	1.62	1.58	1.70
Thailand	0.02	0.02	0.03	0.11	0.09	0.13	2.00	2.15	1.95
The Bahamas	0.75	0.66	0.83	1.98	2.35	1.59	1.36	2.03	0.84
The Gambia	0.01	0.02	0.01	0.02	0.03	0.01	1.66	2.80	0.52
Timor-Leste	0.01	0.01	0.01	0.02	0.02	0.02	6.39	7.43	5.71
Togo	0.01	0.02	0.01	0.02	0.03	0.01	0.97	1.65	0.23
Tonga	0.05	0.04	0.06	0.29	0.25	0.32	4.38	6.74	3.51
Trinidad and Tobago	0.7	0.65	0.75	1.53	1.86	1.2	0.83	1.60	0.28
Tunisia	0.04	0.02	0.07	0.11	0.07	0.15	1.51	3.00	1.17
Turkey	0.52	0.35	0.69	0.64	0.48	0.8	1.20	1.68	1.10
Turkmenistan	0.04	0.04	0.05	0.18	0.13	0.22	4.80	4.27	5.16
Uganda	0.06	0.06	0.06	0.08	0.07	0.09	1.79	1.87	1.87
Ukraine	0.15	0.16	0.14	0.97	0.84	1.14	4.45	3.86	5.37
United Arab Emirates	0.02	0.01	0.02	0.03	0.02	0.04	1.32	1.84	1.24
United Kingdom	4.58	4.86	4.28	4.56	5.54	3.55	0.21	0.36	0.02
United States	1.52	1.58	1.46	2.64	2.84	2.43	0.79	0.79	0.79
Uruguay	0.16	0.08	0.25	0.22	0.26	0.18	0.25	1.71	-0.26
Uzbekistan	0.05	0.05	0.04	0.08	0.08	0.08	0.98	1.02	1.09
Vanuatu	0.03	0.01	0.05	0.09	0.04	0.13	1.77	2.07	1.87
Venezuela	0.16	0.17	0.15	0.26	0.32	0.2	0.33	0.63	0.07
Vietnam	0.02	0.03	0.02	0.13	0.14	0.12	4.83	4.83	5.24
Virgin Islands, U.S.	0.47	0.24	0.71	4.2	6.21	2	3.27	11.74	0.33
Yemen	0.01	0.01	0.01	0.01	0.01	0.02	3.06	3.11	3.37
Zambia	0.06	0.07	0.04	0.09	0.1	0.09	1.54	1.26	2.09
Zimbabwe	0.02	0.02	0.02	0.03	0.02	0.03	0.57	0.36	0.77

Niger	920.69	916.3	925.23	1001.67	929.14	1073.02	0.10	0.02	0.18
Nigeria	1049.31	1048.39	1050.2	1050.81	1018.67	1081.75	0.00	-0.03	0.03
North Korea	944.79	1211.46	662.36	1656.43	2036.1	1259.65	0.76	0.69	0.91
Northern Mariana Islands	521.05	484.89	553.55	556.12	465.28	647.61	0.08	-0.03	0.20
Norway	3534.45	3918.36	3141.88	3339.11	3621.52	3059.98	-0.06	-0.08	-0.04
Oman	692.62	775.4	626.58	927.08	995.85	892.21	0.33	0.28	0.42
Pakistan	1115	1032.97	1191.14	1365.3	1362.73	1367.73	0.23	0.32	0.15
Palestine	720.54	730.46	710.85	962.48	973.74	951.54	0.32	0.32	0.32
Panama	1618.25	1631.84	1604.97	2758.78	2781.19	2736.5	0.73	0.73	0.74
Papua New Guinea	535.02	575.26	496.61	745.85	808.33	685.82	0.40	0.41	0.38
Paraguay	1826.31	1881.81	1772.07	2755.34	2741.87	2768.41	0.49	0.44	0.55
Peru	1374.51	1391.73	1357.29	2341.98	2402.79	2281.09	0.71	0.73	0.68
Philippines	844.85	903.86	787.07	1333.92	1437.93	1231.83	0.59	0.60	0.58
Poland	3309.85	3782.62	2812.62	4927.57	5581.34	4223.97	0.47	0.46	0.49
Portugal	3158.33	3428.56	2867.69	4204.05	4624.75	3755.43	0.31	0.33	0.28
Puerto Rico	2908.24	2889.54	2928.13	4391.75	4660.6	4101.32	0.50	0.60	0.39
Qatar	666.48	557.16	720.45	727.57	673.31	747.87	0.08	0.19	0.03
Romania	3330.32	3613.59	3038.91	5377.08	5938.67	4779.33	0.60	0.63	0.56
Russia	2918.43	3657.95	2078.64	4140.65	4943.21	3198.16	0.41	0.34	0.52
Rwanda	796.41	814.4	777.77	990.41	1060.83	913.81	0.25	0.31	0.18
Saint Lucia	1968.32	1987.72	1948.36	2801.32	2834.37	2767	0.44	0.44	0.45
Saint Vincent and the Grenadines	1707.02	1813.41	1599.56	2420.73	2433.4	2408.3	0.43	0.34	0.52
Samoa	942.59	1016.44	874.52	1358.35	1494.17	1230.81	0.43	0.46	0.40
Sao Tome and Principe	1575.88	1562.81	1589.2	1241.91	1280.37	1203.17	-0.21	-0.18	-0.24
Saudi Arabia	836.62	882.68	800.39	1195.32	1138.14	1239.25	0.41	0.27	0.52
Senegal	1046.87	1038.63	1055.19	1064.67	1106.57	1021.23	0.02	0.07	-0.02
Serbia	3041.48	3290.16	2786.11	4997.18	5416.27	4557.62	0.63	0.63	0.62
Seychelles	1809.61	2031.62	1586.65	2410.92	2663.64	2164.9	0.33	0.31	0.36
Sierra Leone	1209.4	1114.16	1306.62	1057.8	1002.92	1113.81	-0.11	-0.09	-0.13
Singapore	1257.8	1454.74	1065.99	2410.33	2829.11	1976.62	0.87	0.91	0.81
Slovakia	2824.71	3155.75	2478.02	4028.18	4614.69	3409.31	0.41	0.45	0.36
Slovenia	3270.8	3827.51	2680.49	5184.2	5802.76	4554.53	0.56	0.50	0.67
Solomon Islands	646.7	598.25	692.57	789.77	769.68	809.24	0.22	0.29	0.17
Somalia	1129.89	1130.68	1129.08	1010.61	1003.73	1017.57	-0.10	-0.11	-0.10
South Africa	2032.84	2221.59	1839.7	2624.16	2946.71	2290.22	0.29	0.32	0.24
South Korea	896.83	1039.76	754.53	2103.85	2438.56	1765.08	1.28	1.28	1.26
South Sudan	1111.32	1101.2	1121.53	1252.02	1235.41	1268.59	0.13	0.12	0.13
Spain	3084.81	3508.59	2643.3	3690.96	4212.75	3155.7	0.19	0.19	0.18
Sri Lanka	1412.11	1362.06	1461.19	2396.45	2506.03	2278.65	0.72	0.86	0.59
Sudan	832.9	820.08	845.62	991.79	993.63	989.98	0.20	0.21	0.18
Suriname	1495.94	1529.7	1463.25	2232.21	2351.7	2113.17	0.49	0.53	0.44
Swaziland	1266.67	1304.25	1224.63	1570.67	1677.84	1461.24	0.24	0.28	0.19
Sweden	3980.33	4307.61	3645.12	3946.38	4163.01	3729.31	-0.02	-0.04	0.01
Switzerland	3276.34	3678.81	2854.85	3654.46	3980.85	3320.71	0.11	0.08	0.15
Syria	912.44	877.74	946.04	1331.3	1364.61	1298.81	0.45	0.55	0.36
Taiwan	1131.49	1148.79	1115.42	2335.74	2491.2	2179.77	1.11	1.19	1.02
Tajikistan	1160.66	1272.42	1047.56	1100.86	1090.11	1111.35	-0.05	-0.15	0.06
Tanzania	1063.34	1084.13	1042.18	1169.81	1131.89	1208.2	0.12	0.06	0.18
Thailand	1290.34	1331.23	1248.69	2875.24	2984.18	2763.18	1.23	1.23	1.21
The Bahamas	1481.12	1582.98	1377.84	2813.65	2967.85	2652.85	0.88	0.85	0.91
The Gambia	929.27	808.1	1048.71	920.63	836.31	1006.61	-0.01	0.04	-0.04
Timor-Leste	595.41	604.1	586.93	1184.55	1204.26	1165.45	1.01	1.01	1.00
Togo	994.28	1005.84	982.42	991.53	994.77	988.21	0.01	-0.01	0.02
Tonga	1084.88	1155.84	1015.64	1471.79	1649.12	1295.42	0.35	0.42	0.27
Trinidad and Tobago	1738.38	1805.54	1670.72	3015.62	3129.9	2898.31	0.72	0.71	0.72
Tunisia	1400.18	1350.87	1448.9	2431.95	2481.26	2381.48	0.71	0.81	0.62
Turkey	1755.5	1785.16	1725.11	2339.36	2509.14	2163.57	0.32	0.40	0.25
Turkmenistan	1207.86	1386.78	1023.93	1553.45	1695.82	1406.22	0.28	0.21	0.36
Uganda	878.01	880.93	875.05	798.82	814.51	783.12	-0.08	-0.07	-0.09
Ukraine	3526.71	4248.6	2686.65	4909.64	5770.37	3893	0.38	0.35	0.44
United Arab Emirates	529.03	483.97	552.43	745.64	528.24	824.88	0.39	0.08	0.47

United Kingdom	3565.3	3937.99	3171.08	3700.62	3907.67	3488.4	0.03	-0.01	0.09
United States	1850.29	1942.03	1753.81	2437.32	2512.79	2359.29	0.31	0.29	0.33
Uruguay	2351.52	2484.69	2210.15	2716.66	3063.44	2345.18	0.15	0.22	0.05
Uzbekistan	1166.08	1329.11	999.43	1510.93	1640.35	1377.26	0.29	0.22	0.37
Vanuatu	856.07	760.26	946.66	1033.52	1029.01	1037.92	0.17	0.32	0.05
Venezuela	1412.82	1466.87	1359.6	2433.06	2527.32	2338	0.70	0.71	0.69
Vietnam	1372.14	1558.32	1180.22	1949.13	2245.68	1646.19	0.45	0.46	0.44
Virgin Islands, U.S.	2006.67	2004.24	2009.25	5070.66	5079.72	5060.7	1.50	1.51	1.49
Yemen	729.57	784.53	673.61	845.63	851.53	839.86	0.16	0.08	0.24
Zambia	976.37	989.68	962.83	1052.86	1082.57	1023.07	0.09	0.11	0.08
Zimbabwe	1256.38	1272.13	1240.49	1235.23	1240	1230.32	-0.01	-0.02	0.00

eTable 4c. All-ages DALY rate for men and women separately and combined, for all CVD level 3 and 4 subcauses, by country in 1990 and 2015									
	1990			2015			Median percent change between 1990-2015		
	Both	Female	Male	Both	Female	Male	Both	Female	Male
**Aortic aneurysm**									
Afghanistan	15.76	8.49	22.67	21.96	12.07	31.25	1.29	1.44	1.18
Albania	16	9.91	21.8	31.42	20.04	42.99	1.21	1.34	1.15
Algeria	11.41	7.07	15.64	15.75	11.54	19.9	0.98	1.31	0.84
American Samoa	38.18	30.67	45.3	32.48	29.68	35.27	0.03	0.02	0.05
Andorra	91.17	48.84	135.81	92.29	44.31	142.29	0.02	-0.07	0.04
Angola	24.34	13.07	35.89	23.92	16.67	31.27	0.97	1.44	0.82
Antigua and Barbuda	37.22	27.2	47.94	39.44	29.63	50.14	0.17	0.19	0.17
Argentina	112.32	59.61	167.1	102.36	60.7	145.82	0.08	0.16	0.07
Armenia	91.84	58.85	126.81	156.51	97.4	224.59	0.90	0.86	0.94
Australia	131.11	74.48	188.04	78.12	53.83	102.47	-0.31	-0.20	-0.34
Austria	95.25	62.89	130.51	73.53	52.01	96.12	-0.08	-0.03	-0.11
Azerbaijan	21.43	9.41	33.96	28.58	13.36	43.98	1.01	1.17	0.94
Bahrain	10.73	4.72	15.15	8.8	4.21	11.61	0.19	0.25	0.14
Bangladesh	7.79	5.54	9.94	13.55	10.39	16.64	3.04	3.71	2.63
Barbados	60.66	34.29	89.07	76.71	47.88	108.05	0.20	0.29	0.18
Belarus	39.08	22.69	57.64	91.04	47.86	141.13	1.01	0.98	0.97
Belgium	116.01	55.76	179.05	92.67	46.6	140.56	-0.09	-0.06	-0.09
Belize	18.37	15.57	21.13	28.06	21.99	34.16	0.82	0.78	0.84
Benin	14.18	12.56	15.9	10.85	5.73	15.99	0.45	-0.14	0.88
Bermuda	165.75	69.61	263.1	134.37	63.32	206.78	0.03	0.09	0.04
Bhutan	8.12	5.69	10.38	17.05	10.57	22.63	3.71	3.53	3.54
Bolivia	13.66	9.55	17.75	18.99	14.5	23.46	2.00	2.29	1.84
Bosnia and Herzegovina	47.02	24.84	69.23	72.59	40.53	104.94	0.57	0.60	0.59
Botswana	27.45	19.59	35.51	42.27	21.48	63.08	0.19	-0.15	0.35
Brazil	56.9	30.59	83.62	95.73	66.3	126.14	1.12	1.72	0.90
Brunei	36.61	29.76	42.74	55.49	50.98	59.73	0.55	0.74	0.43
Bulgaria	54.65	30.52	79.46	85.84	52.53	120.8	0.53	0.64	0.51
Burkina Faso	18.74	19.4	18.04	11.14	8.44	13.87	0.10	-0.21	0.44
Burundi	42.87	21.48	65.01	17.37	10.28	24.63	-0.09	0.08	-0.17
Cambodia	16.03	11.2	21.19	22.48	16.94	28.29	2.42	2.79	2.18
Cameroon	24.02	22.67	25.37	13.77	7.86	19.68	-0.24	-0.54	0.01
Canada	111.99	61.77	163.08	75.41	46.53	104.7	-0.30	-0.25	-0.30
Cape Verde	8.08	3.32	13.35	11.78	4.88	18.86	1.25	1.31	1.17
Central African Republic	35.83	16.37	55.95	41.52	18.34	65.31	0.41	0.30	0.40
Chad	13.7	13.42	13.98	9.08	5.37	12.78	-0.04	-0.43	0.31
Chile	46.47	30.65	62.72	67.7	50.56	85.29	0.67	0.78	0.65
China	15.06	8.33	21.44	20.3	10.71	29.35	1.00	1.09	0.90
Colombia	42.58	22.3	63.12	48.75	28.47	69.66	0.53	0.58	0.56
Comoros	31.4	13.53	49.15	18.96	10.02	27.75	0.31	0.60	0.22
Congo	38.71	21.83	55.67	31.55	26.96	36.13	0.16	0.54	0.03
Costa Rica	27.99	11.75	43.98	46.62	21.63	71.57	0.75	0.91	0.72
Cote d'Ivoire	21.4	14.62	27.65	13.27	5.94	20.35	-0.13	-0.42	0.01
Croatia	56.34	32.24	81.95	122.76	65.88	183.8	1.35	1.11	1.51
Cuba	87.37	44.47	129.63	109.68	61.46	157.45	0.20	0.32	0.17
Cyprus	108.3	45.85	172.33	78.17	32.24	127.28	-0.16	-0.17	-0.15
Czech Republic	58.62	29.33	89.67	86.69	54.64	119.77	0.84	1.24	0.71
Democratic Republic of the Congo	14.18	10.67	17.78	16.92	10.75	23.13	0.94	0.63	1.12
Denmark	178.72	90.3	269.72	152.42	90.36	215.46	0.05	0.21	0.01
Djibouti	29.12	11.88	46.15	39.63	15.91	63.15	0.85	0.83	0.81
Dominica	49.81	44.32	55.33	90.13	67.49	113.13	0.64	0.51	0.71
Dominican Republic	16.34	8.68	23.9	25.95	15.38	36.59	1.14	1.44	1.03
Ecuador	21.96	15.39	28.46	34.23	23.86	44.6	1.18	1.21	1.16
Egypt	37.92	29.4	46.39	46.07	34.69	57.21	1.10	1.13	1.06
El Salvador	14.12	6.6	22	21.15	9.96	33.79	1.23	1.23	1.26
Equatorial Guinea	39.14	20.35	57.12	35.32	21.85	48.12	0.72	1.01	0.61
Eritrea	24.59	11.81	37.53	24.16	14.36	33.92	0.72	1.03	0.59
Estonia	73.68	46.18	104.97	88.02	56.69	124.8	0.52	0.46	0.60

Ethiopia	29.83	12.2	47.56	17.18	9.29	25.1	0.79	1.31	0.64
Federated States of Micronesia	46.85	28.69	64.23	58.76	40.67	76	0.69	0.90	0.60
Fiji	63.43	40.05	86.09	109.49	85.48	132.71	0.50	0.79	0.39
Finland	180.02	84.74	281.1	157.14	83.73	232.8	0.00	0.10	-0.02
France	84.26	41.64	129.2	78.92	39.27	121.12	0.04	0.02	0.07
Gabon	60.35	31.72	90.18	46.3	31.55	60.72	0.15	0.44	0.02
Georgia	23.47	12.26	35.84	78.66	27.09	135.06	2.38	1.38	2.62
Germany	93.96	58.42	132.14	88.11	58.58	118.65	0.05	0.13	0.01
Ghana	26.83	35.73	18.22	18.88	19.2	18.55	0.18	-0.07	0.62
Greece	109.91	61.36	159.7	157.1	81.71	236.23	0.45	0.34	0.51
Greenland	36.29	25.08	45.99	37.67	27.33	46.63	0.45	0.56	0.39
Grenada	64.41	33.42	96.22	85.46	42.25	128.39	0.45	0.42	0.44
Guam	87.37	23.94	142.78	127.14	57.68	194.65	0.08	0.77	0.00
Guatemala	12.1	6.37	17.91	11.64	5.87	17.66	0.77	0.75	0.75
Guinea	13.62	14.4	12.82	9.47	5.18	13.74	0.30	-0.32	0.95
Guinea-Bissau	21.67	19.09	24.31	13.21	7.23	19.27	-0.11	-0.45	0.17
Guyana	27.32	18.47	36.46	53.6	34.61	72.41	1.23	1.18	1.23
Haiti	28.33	21.27	35.61	30.12	22.01	38.4	1.04	0.95	1.11
Honduras	7.45	5.45	9.44	13.08	5.57	20.6	1.75	0.55	2.51
Hungary	74.34	50.88	99.71	85.17	62.23	110.45	0.47	0.46	0.51
Iceland	71.92	46.29	97.33	62.88	35.38	90.97	0.02	-0.10	0.07
India	11.7	7.16	15.93	30.97	17.47	43.52	3.55	3.58	3.34
Indonesia	14.92	10.86	18.95	27.36	19.53	35.08	1.83	1.88	1.76
Iran	6.75	3.93	9.46	13.92	8.61	19.15	2.94	3.45	2.68
Iraq	35.69	32.13	39.18	42.93	35.81	49.88	0.49	0.50	0.48
Ireland	89.77	56.37	123.46	79.04	46.21	112.85	0.08	0.01	0.12
Israel	36.27	21.98	50.61	31.28	17.9	44.88	0.04	-0.03	0.08
Italy	80.9	33.68	131	98.46	48.07	152.03	0.33	0.48	0.34
Jamaica	25.55	21.84	29.34	47.56	34.9	60.33	0.77	0.58	0.89
Japan	75.27	53.28	98.05	161.82	125.21	200.36	0.91	1.12	0.78
Jordan	20.24	10.97	28.63	17.75	9.64	25.47	0.32	0.37	0.30
Kazakhstan	56.04	32.11	81.47	62.56	30.35	97.08	0.21	0.08	0.25
Kenya	12.04	7.19	16.93	17.72	9.45	26	1.04	0.86	1.09
Kiribati	19.92	13.22	26.76	25.51	19.81	31.37	0.81	1.31	0.55
Kuwait	11.16	6.92	14.87	15.32	7.97	21.06	0.70	0.34	0.84
Kyrgyzstan	13.49	9.46	17.69	13.11	8.48	17.84	0.37	0.34	0.37
Laos	21.08	14	28.17	19.56	14.25	24.93	1.57	1.94	1.37
Latvia	63.28	34.4	96.55	74.33	39.86	114.47	0.36	0.31	0.41
Lebanon	21.68	12.29	31.34	17.71	12.73	22.65	0.42	0.60	0.39
Lesotho	26.58	17.69	35.99	42.42	22.91	62.32	0.00	-0.23	0.11
Liberia	14.9	13.8	15.99	7.02	4.32	9.69	0.22	-0.24	0.64
Libya	8.59	5.83	11.06	15.9	9.5	22.23	0.97	0.91	0.96
Lithuania	54.61	30.97	80.91	85.69	44.85	133.18	0.58	0.47	0.63
Luxembourg	106.65	56.49	158.85	61.36	35.99	86.83	-0.18	-0.14	-0.17
Macedonia	23.26	11.83	34.55	44.68	22.06	67.5	1.07	1.01	1.11
Madagascar	40.02	18.5	61.6	41.6	20.36	62.98	0.93	1.07	0.83
Malawi	12.54	8.32	16.91	13.22	6.84	19.62	1.27	0.81	1.42
Malaysia	35.26	18.15	51.87	47.05	21.7	72.88	0.41	0.36	0.40
Maldives	20.65	14.26	26.72	14.99	9.65	20.3	0.95	0.91	0.95
Mali	16.64	17.59	15.68	5.96	4.87	7.04	-0.37	-0.52	-0.20
Malta	48.56	20.64	77.46	56.13	24.24	88.28	0.16	0.18	0.15
Marshall Islands	39.33	21.3	56.58	58.71	34.06	82.52	0.79	0.89	0.77
Mauritania	18.65	15.58	21.74	7.5	5.48	9.49	-0.27	-0.38	-0.19
Mauritius	23.93	15.86	32.04	25.35	14.65	36.31	0.04	-0.11	0.13
Mexico	12.61	6.39	18.89	13.84	6.96	20.8	0.45	0.40	0.48
Moldova	23.47	13.9	33.97	36.57	22.25	52.05	0.89	0.99	0.82
Mongolia	8.94	7.28	10.65	13.89	11.67	16.15	1.27	1.74	0.98
Montenegro	126.15	73.64	179.12	203.48	116.62	292.41	0.56	0.58	0.54
Morocco	14.33	9.76	18.91	21.12	14.74	27.66	1.21	1.26	1.19
Mozambique	13.65	6.24	21.74	21.5	8.72	34.87	1.86	1.47	1.90
Myanmar	18.61	16.18	21.15	31.82	27.34	36.51	1.99	2.18	1.84

Namibia	34.95	21.64	48.83	26.87	12.07	42.46	-0.11	-0.34	-0.02
Nepal	7.03	4.49	9.62	13.41	7.54	19.65	3.36	3.12	3.35
Netherlands	147.75	65.76	231.59	131.93	77.62	187.14	-0.03	0.24	-0.09
New Zealand	174.81	114.02	237.47	114.19	88.23	141.13	-0.22	-0.12	-0.25
Nicaragua	7.02	3.87	10.22	10.67	6.03	15.44	1.71	1.82	1.66
Niger	10.01	9.13	10.93	5.11	2.83	7.36	0.19	-0.28	0.56
Nigeria	17.24	17.15	17.33	8.16	4.75	11.43	-0.14	-0.49	0.18
North Korea	17.56	12.92	22.47	27.66	18.68	37.05	0.67	0.60	0.69
Northern Mariana Islands	40.5	45.23	36.24	26.51	21.93	31.12	-0.18	-0.34	0.00
Norway	197.05	105.85	290.31	125.55	81.41	169.17	-0.17	-0.06	-0.19
Oman	15.72	5.37	23.98	17.41	4.65	23.88	0.89	0.57	0.67
Pakistan	11.97	5.11	18.35	25.47	11.53	38.66	2.20	2.58	2.03
Palestine	13.94	10.92	16.88	20.65	13.23	27.86	1.25	0.93	1.41
Panama	32.55	15.19	49.53	37.54	18.23	56.74	0.22	0.29	0.20
Papua New Guinea	35.76	23.75	47.22	43.12	29.71	56	0.65	0.68	0.65
Paraguay	26.64	15.61	37.42	55.45	32.26	77.93	1.22	1.34	1.12
Peru	14.9	9.63	20.18	15.96	11.84	20.09	1.21	1.48	1.10
Philippines	20.76	13.14	28.23	24.47	17.27	31.54	0.63	0.82	0.54
Poland	90.29	37.41	145.9	98.04	46.4	153.62	0.26	0.41	0.23
Portugal	51.69	28.22	76.92	58.64	28.1	91.21	0.37	0.16	0.47
Puerto Rico	37.51	18.02	58.22	31.7	20.36	43.95	-0.09	0.10	-0.13
Qatar	11.53	4.29	15.11	8.53	3.84	10.28	0.02	0.22	-0.05
Romania	47.54	34.26	61.19	69.33	52.71	87.02	0.53	0.64	0.46
Russia	17.29	10.44	25.08	104.91	57.9	160.11	4.64	4.38	4.69
Rwanda	25.43	16.02	35.17	17.36	11.86	23.34	0.59	0.73	0.51
Saint Lucia	82.71	70.08	95.72	107.04	95.5	119.02	0.53	0.61	0.47
Saint Vincent and the Grenadines	68.28	31.62	105.3	61.13	39.49	82.38	-0.09	0.35	-0.25
Samoa	40.47	25.35	54.4	44.82	39.85	49.49	0.29	0.61	0.18
Sao Tome and Principe	20.63	16.68	24.66	13.7	8.65	18.78	0.18	-0.10	0.34
Saudi Arabia	4.95	2.36	6.98	7.72	2.96	11.38	1.73	1.38	1.70
Senegal	15.14	12.89	17.42	9.17	5.39	13.1	0.18	-0.20	0.43
Serbia	62.79	30.43	96.02	108.74	61.98	157.78	0.69	0.96	0.62
Seychelles	39.25	18.15	60.44	36.67	20.93	52	0.04	0.23	-0.02
Sierra Leone	14.36	9.84	18.96	9.41	5.6	13.31	0.17	-0.03	0.30
Singapore	46.77	21.82	71.07	42.33	24.88	60.4	0.05	0.27	0.01
Slovakia	42.98	28.02	58.65	59.61	34.26	86.35	0.66	0.38	0.85
Slovenia	56.54	33.22	81.27	74.35	41.54	107.75	0.54	0.40	0.62
Solomon Islands	37.35	21.56	52.3	47.6	30.79	63.89	0.66	0.84	0.61
Somalia	31.54	13.36	50.05	22.22	9.58	34.98	0.04	0.06	0.04
South Africa	44.52	31.08	58.27	46.92	25.44	69.17	0.00	-0.22	0.12
South Korea	27.67	22.73	32.58	38.35	31.96	44.82	0.60	0.59	0.61
South Sudan	22.5	13	32.1	21.34	11.88	30.77	0.50	0.44	0.52
Spain	65.63	27.68	105.17	71.55	29.42	114.76	0.30	0.19	0.38
Sri Lanka	22.79	13.4	32	30.67	20.56	41.53	0.89	1.05	0.86
Sudan	13.94	7.62	20.21	16.56	9.13	23.94	1.31	1.37	1.25
Suriname	29.41	12.76	45.53	43.19	18.41	67.89	0.68	0.72	0.64
Swaziland	30.18	20.79	40.68	45.63	23.83	67.9	0.10	-0.21	0.24
Sweden	199.95	112.36	289.66	157.41	104.87	210.04	-0.05	0.07	-0.09
Switzerland	107.35	52.19	165.12	81.38	46.77	116.76	-0.06	0.05	-0.07
Syria	17.29	9.75	24.58	18.62	12.47	24.63	-0.10	0.38	-0.30
Taiwan	50.41	18.9	79.66	59.96	33.32	86.69	0.18	0.69	0.09
Tajikistan	8.31	5.03	11.62	9.68	5.73	13.55	1.28	1.29	1.24
Tanzania	16.33	9.45	23.34	17.94	9.33	26.66	1.08	0.85	1.11
Thailand	22.05	11.06	33.24	48.31	28.41	68.78	1.12	1.61	0.93
The Bahamas	53.13	40	66.44	79.66	68.34	91.47	0.65	0.81	0.56
The Gambia	12.22	11.73	12.71	6.38	3.4	9.42	-0.03	-0.43	0.31
Timor-Leste	10.96	8.56	13.3	13.6	12.01	15.14	2.24	2.71	1.93
Togo	17.02	15.5	18.57	11.3	5.11	17.63	0.00	-0.48	0.35
Tonga	21.11	5.07	36.76	31.94	6.92	56.84	0.67	0.58	0.62
Trinidad and Tobago	70.57	42.25	99.11	120.65	76.13	166.36	0.62	0.81	0.52
Tunisia	13.86	6.52	21.11	19.37	9.65	29.31	0.87	1.08	0.78

Turkey	50.07	19.3	81.59	43.15	21.19	65.88	0.64	1.07	0.55
Turkmenistan	11.7	6.76	16.77	25.85	13.74	38.37	2.70	2.61	2.65
Uganda	19.21	12.46	26.06	23.19	11.43	34.95	1.44	0.89	1.62
Ukraine	40.54	23.93	59.87	70.37	40.52	105.64	0.61	0.63	0.57
United Arab Emirates	26.65	8.85	35.9	45.64	14.1	57.13	1.15	1.28	0.94
United Kingdom	231.15	137.95	329.74	152.65	104.03	202.49	-0.18	-0.10	-0.22
United States	103.14	58.77	149.8	64.01	42.09	86.66	-0.33	-0.26	-0.35
Uruguay	147.06	89.81	207.83	146.33	96.94	199.23	0.12	0.16	0.11
Uzbekistan	5.61	3.31	7.95	8.48	4.67	12.41	1.03	0.97	1.02
Vanuatu	45.74	23.5	66.77	56.12	35.71	76.02	0.40	0.69	0.32
Venezuela	26.17	13.39	38.74	33.54	18.35	48.87	0.24	0.46	0.13
Vietnam	14.73	10.08	19.53	28.21	19.31	37.31	1.78	1.94	1.64
Virgin Islands, U.S.	51.96	28.53	76.78	85.45	49.05	125.46	0.23	0.34	0.18
Yemen	10.83	5.9	15.85	16.32	8.83	23.66	2.18	2.21	2.11
Zambia	15.24	12	18.54	30.04	12.77	47.35	2.30	0.85	3.07
Zimbabwe	9.91	6.39	13.45	17.69	7.32	28.36	0.56	-0.02	0.84
**Atrial fibrillation and flutter**									
Afghanistan	13.76	13.65	13.87	17.27	17.57	16.98	1.06	1.21	0.94
Albania	59.79	55.06	64.31	117.86	107.96	127.93	1.21	1.26	1.17
Algeria	17.76	17.03	18.47	33.48	30.23	36.69	1.69	1.51	1.86
American Samoa	23.59	20.42	26.59	22.21	21.61	22.81	0.15	0.12	0.16
Andorra	130.11	125.37	135.11	176.03	161.65	191.02	0.36	0.32	0.40
Angola	9.38	7.91	10.89	9.14	7.51	10.79	1.03	0.90	1.14
Antigua and Barbuda	36.55	33.4	39.91	37.23	32.26	42.66	0.13	0.05	0.20
Argentina	69.52	55.31	84.29	81.56	69.5	94.14	0.39	0.43	0.37
Armenia	59.45	58.9	60.02	106.02	105.08	107.11	0.99	1.01	0.96
Australia	77.25	64.31	90.25	96.31	74.96	117.72	0.45	0.29	0.58
Austria	150.33	166.63	132.57	261.05	257.9	264.36	1.06	0.81	1.41
Azerbaijan	42	42.36	41.62	62.62	57.83	67.47	1.24	1.08	1.42
Bahrain	13.36	11.34	14.85	15.62	14.52	16.3	0.69	0.79	0.63
Bangladesh	16.35	12.16	20.36	25.52	18.32	32.58	2.62	2.78	2.47
Barbados	46.14	42.83	49.71	68.22	60.44	76.68	0.40	0.30	0.50
Belarus	92.8	103.13	81.1	124.5	137.36	109.58	0.16	0.25	0.09
Belgium	106.94	112.94	100.66	137.91	139.03	136.74	0.47	0.38	0.58
Belize	23.49	18.76	28.14	21.67	15.96	27.41	0.10	0.07	0.11
Benin	11.09	9.81	12.45	10.02	8.44	11.6	0.75	0.68	0.81
Bermuda	30.15	23.99	36.38	41.29	33.05	49.69	0.74	0.65	0.80
Bhutan	17.83	12.99	22.32	27.73	18.55	35.64	2.49	2.47	2.33
Bolivia	20.84	17.38	24.28	34.48	28.52	40.42	2.57	2.55	2.58
Bosnia and Herzegovina	83.72	83.42	84.03	146.15	141.7	150.63	0.77	0.66	0.87
Botswana	8.66	7.72	9.63	12.27	10.22	14.33	0.15	0.10	0.19
Brazil	18.82	16.4	21.28	38.52	33.54	43.67	1.58	1.56	1.59
Brunei	13.57	5.77	20.56	22.96	10.08	35.08	0.72	0.77	0.74
Bulgaria	127.68	119.53	136.05	180.37	183.03	177.58	0.38	0.46	0.29
Burkina Faso	11.6	10.06	13.21	9.96	9.23	10.71	0.61	0.69	0.54
Burundi	11.85	9.92	13.85	10.66	8.22	13.16	1.02	0.89	1.11
Cambodia	21.9	21.73	22.08	30.59	29.84	31.37	2.41	2.45	2.39
Cameroon	12.63	10.26	15.03	11.34	9	13.67	0.21	0.19	0.21
Canada	90.54	65.37	116.15	123.83	84.22	164	0.43	0.28	0.54
Cape Verde	15.44	12.86	18.29	17.5	14.97	20.1	0.75	0.82	0.69
Central African Republic	15.92	13.56	18.37	16.33	14.02	18.7	0.27	0.27	0.28
Chad	11.07	9.38	12.79	8.6	7.12	10.08	0.14	0.12	0.16
Chile	58.72	45.74	72.05	91.96	74.82	109.56	0.80	0.76	0.84
China	31.75	30.42	33.01	58.6	53.07	63.82	1.74	1.83	1.68
Colombia	19.74	16.6	22.93	33.25	27.76	38.9	1.25	1.07	1.39
Comoros	11.81	9.67	13.93	11.58	9.03	14.07	1.12	1.03	1.19
Congo	13.92	11.41	16.44	14.42	11.74	17.09	0.48	0.31	0.67
Costa Rica	23.87	19.29	28.38	41.92	33.81	50.03	0.84	0.82	0.86
Cote d'Ivoire	10.26	7.18	13.1	10.53	7	13.94	0.46	0.42	0.49
Croatia	87.23	88.79	85.57	110.93	109.34	112.63	0.37	0.27	0.47
Cuba	43.29	34.01	52.44	69.82	57.24	82.28	0.55	0.61	0.51

Cyprus	98.92	92.53	105.47	106.1	99.69	112.94	0.25	0.27	0.23
Czech Republic	150.9	158.7	142.62	227.1	220.95	233.44	0.87	0.67	1.09
Democratic Republic of the Congo	10.98	9.82	12.16	12.86	10.93	14.8	0.91	0.81	0.99
Denmark	101.79	100.8	102.81	141.86	130.9	153.01	0.72	0.57	0.88
Djibouti	10.52	8.35	12.65	15.52	11.73	19.28	1.07	1.02	1.12
Dominica	33.88	27.35	40.45	47.97	39.53	56.54	0.28	0.43	0.17
Dominican Republic	20.31	14.85	25.7	34.3	26.64	42.01	1.27	1.46	1.16
Ecuador	20.69	16.53	24.81	33.05	26.6	39.5	1.23	1.29	1.19
Egypt	24.93	25.75	24.11	27.29	27.18	27.39	0.89	0.90	0.89
El Salvador	22.4	18.18	26.82	40.57	32.69	49.48	1.69	1.66	1.72
Equatorial Guinea	18.48	15.76	21.07	12.37	8.82	15.74	0.33	0.11	0.48
Eritrea	7.56	6.82	8.32	9.99	9.02	10.96	1.37	1.32	1.42
Estonia	99.62	112.22	85.29	143.86	160.75	124.03	0.84	0.71	0.96
Ethiopia	12.43	9.03	15.86	12.73	9.65	15.83	2.24	2.34	2.19
Federated States of Micronesia	26.52	23.43	29.47	33.69	31.02	36.23	0.72	0.78	0.67
Fiji	20.68	17.43	23.83	36.98	33.13	40.69	0.56	0.59	0.54
Finland	176.42	191.48	160.46	274.37	282.3	266.2	0.78	0.64	0.95
France	143.85	148.2	139.26	187.21	186.3	188.19	0.45	0.35	0.54
Gabon	20.06	16.69	23.57	18.59	15.24	21.88	0.40	0.35	0.45
Georgia	82.52	86.65	77.95	129.57	135.27	123.33	0.59	0.68	0.52
Germany	160.1	174.97	144.13	218.91	214.12	223.87	0.54	0.38	0.75
Ghana	9.45	7.98	10.88	12.08	10.71	13.47	1.18	1.40	1.03
Greece	122.48	112.47	132.75	191.11	186.32	196.14	0.58	0.66	0.50
Greenland	33.84	22.46	43.69	62.51	34.2	87.05	1.57	1.17	1.74
Grenada	36.25	33.43	39.14	38.54	36.26	40.8	0.16	0.21	0.12
Guam	26.79	24.14	29.1	51.12	45.66	56.42	0.42	0.39	0.43
Guatemala	16.68	12.8	20.62	24.55	20.31	28.97	1.70	2.01	1.50
Guinea	11.43	9.37	13.53	10.95	8.67	13.21	0.81	0.79	0.81
Guinea-Bissau	12.53	10.37	14.74	11.06	8.72	13.44	0.36	0.29	0.41
Guyana	23.12	18.61	27.78	25.93	20.68	31.14	0.27	0.29	0.26
Haiti	21.58	18.36	24.9	26.75	23.3	30.28	1.39	1.40	1.38
Honduras	17.57	14.18	20.93	28.59	22.97	34.21	1.56	1.46	1.64
Hungary	115.25	119.92	110.19	143.93	154.45	132.34	0.60	0.53	0.63
Iceland	164.32	137.43	190.98	201.11	162.85	240.19	0.42	0.40	0.44
India	21.34	16.75	25.61	32.48	25.56	38.91	1.61	1.86	1.41
Indonesia	24.05	21.24	26.83	34.08	30.24	37.86	1.19	1.29	1.12
Iran	16.46	13.61	19.2	28.84	23.11	34.49	2.34	2.45	2.27
Iraq	20.16	19.57	20.74	17.11	16.85	17.37	0.05	0.16	-0.02
Ireland	106.19	95.02	117.46	109.65	90.91	128.96	0.27	0.18	0.34
Israel	90.97	82.77	99.19	114.53	109.56	119.59	0.52	0.58	0.47
Italy	132.4	130.67	134.23	193.81	187.55	200.46	0.60	0.49	0.73
Jamaica	38.26	32.33	44.33	52.23	43.15	61.39	0.30	0.32	0.27
Japan	49.7	31.38	68.69	103.21	69.34	138.89	0.84	0.99	0.77
Jordan	19.48	17.31	21.44	19.89	17.77	21.91	0.54	0.60	0.49
Kazakhstan	54.25	60.26	47.86	63.48	65.6	61.21	0.27	0.24	0.34
Kenya	9.55	7.23	11.87	10.79	8.35	13.24	0.57	0.63	0.52
Kiribati	28.28	28.73	27.82	32.23	33.17	31.27	0.61	0.78	0.49
Kuwait	10.09	7.13	12.69	12.5	9.36	14.95	0.53	0.53	0.53
Kyrgyzstan	48.73	52.49	44.8	52.02	50.63	53.45	0.51	0.44	0.62
Laos	24.88	23.74	26.03	26.19	23.94	28.46	1.92	1.91	1.92
Latvia	102.36	114.42	88.47	139.35	156.32	119.59	0.58	0.54	0.61
Lebanon	31.32	29.32	33.38	39.53	36.84	42.2	1.20	0.95	1.44
Lesotho	13.84	11.98	15.81	14.33	13.7	14.97	-0.34	-0.30	-0.38
Liberia	9.79	7.48	12.11	10.41	8.17	12.63	1.76	1.68	1.85
Libya	14.84	13.48	16.06	24.74	23.17	26.28	0.77	1.01	0.60
Lithuania	92.42	98.26	85.92	136.04	149.42	120.47	0.48	0.54	0.39
Luxembourg	140.11	151.49	128.26	150.2	154.9	145.48	0.53	0.37	0.71
Macedonia	83.1	75.75	90.37	128.48	124.65	132.35	0.67	0.78	0.58
Madagascar	13.81	9.89	17.74	14.2	10.91	17.51	0.94	1.13	0.82
Malawi	9.4	7.54	11.33	11.07	8.99	13.16	1.56	1.66	1.47
Malaysia	24.41	20.36	28.33	34.89	26.98	42.96	0.51	0.51	0.51

Maldives	21.45	14.13	28.4	32.27	24.11	40.39	3.03	3.79	2.65
Mali	12.33	10.13	14.57	8.9	7.47	10.29	0.28	0.30	0.27
Malta	92.37	90.8	94	141.59	146.23	136.92	0.54	0.61	0.46
Marshall Islands	20.63	18.35	22.81	27.4	24.39	30.31	0.60	0.57	0.62
Mauritania	10.19	8.58	11.81	10.69	8.97	12.39	0.93	0.88	0.98
Mauritius	29.09	28.79	29.4	56.38	52.44	60.42	0.90	0.74	1.04
Mexico	20.86	17.36	24.4	33.63	27.86	39.45	1.12	1.06	1.17
Moldova	73.52	75.25	71.62	103.92	106.29	101.37	0.72	0.76	0.68
Mongolia	40.85	37.53	44.24	44.43	40.96	47.98	0.59	0.86	0.41
Montenegro	124.01	126.65	121.36	197.75	192.11	203.53	0.54	0.51	0.58
Morocco	21.23	20.84	21.61	35.62	33.51	37.78	1.53	1.41	1.64
Mozambique	11.32	9.26	13.57	11.93	9.75	14.22	0.94	0.92	0.95
Myanmar	34.93	39.26	30.42	43.96	46.38	41.43	1.22	1.23	1.26
Namibia	11.02	9.45	12.65	12.02	10.22	13.92	0.29	0.31	0.27
Nepal	18.09	15.46	20.75	26.99	19.77	34.67	2.41	2.14	2.56
Netherlands	182.33	184.55	180.05	241.43	224.34	258.8	0.44	0.28	0.61
New Zealand	156.65	156.92	156.36	199.38	186.68	212.57	0.52	0.34	0.71
Nicaragua	15.37	12.91	17.87	28.64	23.81	33.61	2.32	2.33	2.31
Niger	7.94	6.44	9.49	8.76	6.74	10.75	1.58	1.45	1.66
Nigeria	9.84	7.79	11.85	8.83	6.64	10.93	0.63	0.58	0.65
North Korea	31.1	35.83	26.08	64.66	73.61	55.3	1.20	1.26	1.17
Northern Mariana Islands	15.84	12.96	18.44	15.42	10.98	19.89	0.22	0.15	0.26
Norway	217.39	204.61	230.46	260.3	223.36	296.8	0.56	0.33	0.79
Oman	12.96	13.61	12.43	15.48	15.28	15.58	1.03	1.02	1.08
Pakistan	24.6	14.53	33.95	26.25	16.93	35.07	0.61	0.85	0.49
Palestine	12.72	12.29	13.15	17.03	16.07	17.96	1.03	1.08	1.00
Panama	23.92	18.68	29.04	36.53	28.76	44.27	0.62	0.65	0.60
Papua New Guinea	17.37	16.76	17.95	20.87	19.66	22.03	0.66	0.59	0.72
Paraguay	19.35	16.09	22.53	30.48	24.44	36.34	0.68	0.72	0.65
Peru	19.04	15.6	22.47	32.05	26.95	37.15	2.46	2.47	2.47
Philippines	20.68	18.82	22.5	30.17	27.98	32.32	1.01	1.05	0.98
Poland	125.72	133.49	117.54	202.91	213.61	191.4	0.86	0.82	0.90
Portugal	139.25	136.5	142.21	189.42	185.21	193.9	0.64	0.58	0.69
Puerto Rico	44.18	34.95	54	64.43	57.25	72.19	0.57	0.60	0.54
Qatar	11.07	8.78	12.21	10.18	8.09	10.96	0.27	0.25	0.25
Romania	76.67	78.61	74.67	104.91	112.64	96.69	0.43	0.53	0.33
Russia	85.71	99.08	70.54	118.56	129.46	105.75	0.29	0.27	0.34
Rwanda	9.51	7.99	11.09	11.15	9.5	12.93	1.77	1.84	1.71
Saint Lucia	34.48	28.95	40.18	49.59	42.52	56.94	0.70	0.73	0.68
Saint Vincent and the Grenadines	31.45	28.43	34.5	40.17	33.81	46.42	0.29	0.28	0.29
Samoa	25.66	23.87	27.3	36.97	35.73	38.13	0.68	0.53	0.81
Sao Tome and Principe	15.39	12.51	18.32	12.78	10.34	15.25	0.50	0.48	0.52
Saudi Arabia	14.18	13.47	14.73	18.82	16.17	20.85	1.32	1.27	1.34
Senegal	10.05	8.18	11.95	10.21	8.68	11.81	1.01	1.11	0.96
Serbia	76.09	76.56	75.6	105.71	110.12	101.09	0.36	0.39	0.32
Seychelles	42.81	41.96	43.66	46.68	44.66	48.65	0.21	0.14	0.27
Sierra Leone	10.27	7.3	13.3	8.91	6.65	11.22	0.56	0.57	0.57
Singapore	28.44	12.78	43.69	51.87	24.14	80.58	1.10	1.10	1.19
Slovakia	128.15	133.45	122.59	205.88	219.49	191.53	0.92	0.85	0.96
Slovenia	103.46	109.27	97.31	168.87	168.11	169.65	0.91	0.73	1.13
Solomon Islands	22.37	16.97	27.47	26.37	21.38	31.21	0.54	0.63	0.50
Somalia	12.36	9.82	14.94	10.73	8.42	13.07	0.31	0.29	0.32
South Africa	15.62	14.04	17.23	20.25	19.09	21.45	0.23	0.29	0.18
South Korea	23.87	16.14	31.56	57.74	37.15	78.58	1.79	1.59	1.91
South Sudan	9.98	7.81	12.17	11.67	9.07	14.27	0.93	0.92	0.93
Spain	151.19	154.53	147.71	203.52	204.15	202.88	0.61	0.48	0.73
Sri Lanka	38.66	31.17	46	54.67	48.79	60.99	0.99	1.10	0.91
Sudan	19.54	18.74	20.33	20.84	19.81	21.86	1.08	1.10	1.06
Suriname	24.23	20.4	27.93	34.82	30.54	39.07	0.64	0.78	0.54
Swaziland	9.17	7.62	10.89	11.51	9.93	13.12	-0.07	-0.06	-0.07
Sweden	222.32	190.45	254.96	308.36	269.36	347.45	0.66	0.62	0.71

Switzerland	92.43	93.07	91.77	102.2	98.92	105.55	0.37	0.24	0.51
Syria	24.74	23.42	26.02	30.94	30.37	31.49	0.04	0.40	-0.16
Taiwan	42.97	36.73	48.76	78.3	67.33	89.31	0.80	0.76	0.84
Tajikistan	38.2	37.09	39.33	37.75	31.13	44.21	0.93	0.68	1.16
Tanzania	9.89	7.81	12.01	11.2	8.02	14.42	1.18	1.01	1.29
Thailand	33.91	30.81	37.07	67.15	58.77	75.78	0.92	0.94	0.92
The Bahamas	20	17.9	22.12	38.73	34.79	42.84	1.13	1.06	1.20
The Gambia	9.17	6.49	11.82	8.63	6.22	11.09	0.75	0.88	0.65
Timor-Leste	15.14	13.89	16.36	27.52	25.08	29.87	3.74	3.78	3.69
Togo	10.5	8.78	12.27	9.82	7.87	11.81	0.42	0.45	0.39
Tonga	31.24	29.14	33.28	43.42	43.6	43.25	0.53	0.74	0.37
Trinidad and Tobago	26.51	22.86	30.19	40.59	35.64	45.67	0.45	0.56	0.37
Tunisia	27.41	25.08	29.71	42.79	39.9	45.75	1.10	1.23	0.99
Turkey	32.74	32.3	33.19	38.97	38.13	39.84	1.26	1.21	1.30
Turkmenistan	36.77	37.68	35.84	44.76	42.13	47.47	1.03	0.98	1.11
Uganda	10.72	8.45	13.02	9.97	7.82	12.13	0.91	0.95	0.86
Ukraine	101.67	113.75	87.61	143.06	156.55	127.14	0.30	0.33	0.30
United Arab Emirates	9.01	7.25	9.92	12.66	7.84	14.41	0.76	0.54	0.77
United Kingdom	134.88	135.46	134.26	185.09	182.38	187.86	0.69	0.61	0.78
United States	157.63	155.29	160.08	202.86	192.03	214.05	0.38	0.27	0.50
Uruguay	90.4	73.71	108.12	110.63	100.24	121.76	0.37	0.46	0.30
Uzbekistan	39.99	40.95	39	49.55	46.76	52.44	0.66	0.59	0.74
Vanuatu	28.09	20.59	35.18	30.14	25.09	35.07	0.23	0.36	0.16
Venezuela	17.33	14.26	20.35	29.06	24.69	33.46	0.63	0.85	0.48
Vietnam	38.89	40.27	37.47	53.51	54.86	52.13	1.00	1.10	0.94
Virgin Islands, U.S.	26.18	21.83	30.8	71.39	58.96	85.04	1.04	1.11	0.99
Yemen	15.87	16.7	15.02	17.84	16.98	18.68	1.39	1.19	1.60
Zambia	9.46	7.72	11.23	9.97	8.14	11.8	0.78	0.87	0.70
Zimbabwe	11.29	9.27	13.32	13.01	10.52	15.58	0.02	-0.01	0.03
**Cardiomyopathy and myocarditis**									
Afghanistan	165.3	203.56	128.88	162.05	195.83	130.33	0.61	0.65	0.60
Albania	266.93	256.43	276.93	368.03	304.22	432.86	0.55	0.37	0.71
Algeria	486.79	473.23	500.02	248.72	221.92	275.15	-0.27	-0.34	-0.21
American Samoa	187.27	175.83	198.1	197.2	168.46	225.79	0.28	0.01	0.53
Andorra	94.52	69.45	120.95	76.62	48.14	106.31	-0.19	-0.29	-0.13
Angola	230.03	238.94	220.91	164.26	163.52	165	0.45	0.33	0.58
Antigua and Barbuda	173.25	173.36	173.14	163.25	128.11	201.57	0.04	-0.19	0.30
Argentina	573.4	421.3	731.49	171.58	118.89	226.56	-0.64	-0.68	-0.62
Armenia	45.98	32.34	60.44	58.89	35.76	85.52	0.43	0.25	0.55
Australia	183.36	119.74	247.32	99.88	61.53	138.32	-0.37	-0.43	-0.32
Austria	586.67	652.35	515.11	196.01	178.75	214.14	-0.60	-0.68	-0.50
Azerbaijan	116.51	75.53	159.27	115.06	54.8	176.03	0.49	0.11	0.65
Bahrain	144.42	126.27	157.77	73.37	57.9	82.84	-0.26	-0.36	-0.22
Bangladesh	20.33	19.95	20.69	28.16	28.52	27.81	2.22	2.59	1.91
Barbados	226.14	198.28	256.16	230.5	155.88	311.58	-0.03	-0.28	0.19
Belarus	307.51	153.21	482.27	608.36	232.41	1,044.48	0.70	0.42	0.74
Belgium	199.16	189.68	209.09	121.83	109.33	134.82	-0.30	-0.36	-0.25
Belize	196.8	230.42	163.74	169.44	152.48	186.49	0.03	-0.17	0.30
Benin	72.04	65.98	78.49	57.12	40	74.3	0.51	0.16	0.78
Bermuda	213.46	163.94	263.61	139.2	76.57	203.03	-0.17	-0.44	0.02
Bhutan	28.22	28.77	27.72	39.38	38.16	40.42	2.13	2.23	2.04
Bolivia	250.1	239.62	260.52	101.77	95.08	108.44	-0.12	-0.14	-0.11
Bosnia and Herzegovina	456.1	462.35	449.84	1,012.30	996.42	1,028.32	1.26	1.11	1.39
Botswana	199.95	205.51	194.26	276.36	250.59	302.15	0.04	-0.08	0.16
Brazil	233.07	192.19	274.6	224.7	160.31	291.25	0.21	0.05	0.34
Brunei	130.19	90.4	165.81	115.69	69.46	159.2	-0.09	-0.22	-0.02
Bulgaria	223.54	196.27	251.58	516.06	468.11	566.37	1.25	1.28	1.23
Burkina Faso	79.34	74.83	84.07	63.03	51.43	74.79	0.48	0.25	0.67
Burundi	150.8	128.15	174.24	66.62	56.57	76.89	-0.01	0.00	-0.03
Cambodia	211.39	212.77	209.92	98.73	83.68	114.54	0.14	-0.01	0.30
Cameroon	85.78	77.32	94.35	71.94	51.65	92.22	0.12	-0.11	0.28

Canada	99.45	65.46	134.03	76.82	51.96	102.04	-0.19	-0.21	-0.17
Cape Verde	39.7	20.71	60.7	37.55	22.69	52.81	0.46	0.72	0.34
Central African Republic	192.22	178.29	206.62	185.59	152.54	219.52	0.18	0.02	0.29
Chad	64.07	63.93	64.22	58.7	47.66	69.71	0.34	0.09	0.57
Chile	98.26	74.1	123.07	103.92	61.16	147.83	0.22	-0.11	0.46
China	64.33	52.46	75.59	59.72	46.54	72.13	0.38	0.45	0.32
Colombia	103.62	88.67	118.77	60.67	48.98	72.73	-0.22	-0.32	-0.14
Comoros	111.45	90.17	132.59	52.74	40	65.24	0.02	-0.05	0.06
Congo	178.57	167.07	190.14	141.31	151.1	131.53	0.12	0.13	0.10
Costa Rica	68.52	57.27	79.6	107.94	77.73	138.1	0.65	0.41	0.83
Cote d'Ivoire	89.55	71.61	106.09	69.07	45.79	91.55	0.09	-0.08	0.19
Croatia	166.93	147.7	187.36	247.69	225.71	271.27	0.60	0.58	0.62
Cuba	73.46	73.86	73.06	157.73	109.61	205.4	1.06	0.42	1.70
Cyprus	112.4	92.68	132.63	63	41.7	85.77	-0.35	-0.47	-0.25
Czech Republic	161.72	124.64	201.01	132.63	94.02	172.48	0.02	-0.09	0.10
Democratic Republic of the Congo	130.17	138.8	121.33	106.33	101.79	110.9	0.33	0.19	0.49
Denmark	109.58	85.27	134.59	87.54	55.81	119.77	-0.01	-0.21	0.12
Djibouti	106.86	80.03	133.37	93.53	56.57	130.16	0.20	-0.01	0.30
Dominica	330.56	293.77	367.63	471.51	257.12	689.3	0.29	-0.13	0.57
Dominican Republic	193.8	206.6	181.16	74.21	80	68.39	-0.48	-0.47	-0.50
Ecuador	105.73	100.04	111.36	51.16	41.56	60.75	-0.32	-0.41	-0.25
Egypt	728.75	650.84	806.13	501.53	434.28	567.39	0.19	0.20	0.17
El Salvador	162.95	133.17	194.14	42.63	23.65	64.08	-0.61	-0.74	-0.51
Equatorial Guinea	247.53	247.61	247.46	157.07	141.56	171.81	0.22	0.10	0.34
Eritrea	94.42	78.29	110.75	71.54	61.07	81.96	0.34	0.33	0.32
Estonia	505.92	260.75	784.92	397.68	172.41	662.19	0.00	-0.21	0.14
Ethiopia	117.04	100.36	133.82	53.2	40.25	66.19	0.42	0.24	0.55
Federated States of Micronesia	250.39	257.8	243.29	221.79	193.53	248.71	0.19	0.00	0.38
Fiji	159.21	150.9	167.26	310.92	287.61	333.47	0.70	0.60	0.79
Finland	158.28	121.57	197.21	237.75	107.61	371.9	0.72	-0.02	1.22
France	171.6	134.15	211.1	128.33	100.79	157.64	-0.17	-0.19	-0.15
Gabon	183.44	148.96	219.35	135.62	116.17	154.63	0.11	0.14	0.07
Georgia	130.8	92.3	173.28	187.28	85.15	298.99	0.44	-0.01	0.66
Germany	285.77	267.86	305.01	205.65	147.31	265.98	-0.19	-0.38	-0.02
Ghana	120.3	156.46	85.29	138.13	147.21	128.96	0.93	0.64	1.41
Greece	253.2	264.69	241.41	158.37	161.76	154.8	-0.37	-0.39	-0.35
Greenland	274.52	128.18	401.18	244.8	84.94	383.41	0.24	-0.05	0.31
Grenada	312.07	346.16	277.1	320.47	264.27	376.32	0.13	-0.14	0.46
Guam	150.49	164.92	137.88	221.93	179.46	263.21	0.09	-0.20	0.41
Guatemala	148.39	128.32	168.75	39.45	30.86	48.41	-0.51	-0.54	-0.49
Guinea	78.95	86.82	70.91	53.66	41.25	66.02	0.28	-0.09	0.70
Guinea-Bissau	94.8	91.11	98.57	81.15	67.71	94.77	0.27	0.09	0.44
Guyana	283.69	286.09	281.22	268.77	214.85	322.18	0.08	-0.13	0.28
Haiti	417.67	501.58	331.28	230.01	243.08	216.65	0.06	-0.09	0.28
Honduras	189.01	169.5	208.39	101.96	70.48	133.44	-0.15	-0.37	0.03
Hungary	547.64	410.88	695.58	388.26	233.36	559.03	-0.09	-0.32	0.09
Iceland	61.17	45.91	76.3	33.7	20.26	47.43	-0.36	-0.48	-0.29
India	25.49	21.93	28.82	49.77	47.13	52.22	2.35	3.03	1.88
Indonesia	93.56	69.33	117.59	84.17	67.9	100.22	0.39	0.57	0.27
Iran	96.98	86.79	106.81	83.94	59.16	108.35	0.65	0.38	0.84
Iraq	280.2	220.37	338.95	197.33	137.64	255.53	-0.13	-0.16	-0.12
Ireland	188.83	158.91	219.01	81.87	46.83	117.94	-0.47	-0.64	-0.34
Israel	124.48	119.05	129.94	45.3	36.39	54.36	-0.56	-0.64	-0.49
Italy	433.83	423.62	444.67	170.22	151.53	190.09	-0.57	-0.63	-0.51
Jamaica	207.72	217.55	197.63	146.43	117.69	175.4	-0.33	-0.46	-0.18
Japan	121.12	108.83	133.84	96.02	85.22	107.38	-0.30	-0.29	-0.30
Jordan	46.42	43.93	48.68	19.54	13.02	25.75	-0.36	-0.54	-0.23
Kazakhstan	152.33	117.35	189.5	291.36	163.1	428.79	1.08	0.59	1.37
Kenya	43.63	38.93	48.36	40.76	29.38	52.13	0.29	0.07	0.47
Kiribati	464	423.56	505.29	281.21	203.16	361.28	-0.14	-0.26	-0.05
Kuwait	61	53.76	67.34	28.42	20.35	34.72	-0.42	-0.56	-0.33

Kyrgyzstan	138.1	81.27	197.37	224.66	100.44	351.51	1.30	0.84	1.42
Laos	320.08	326.98	313.17	154.7	141.52	168.01	0.34	0.25	0.43
Latvia	700.85	394.63	1,053.58	968.6	454.9	1,566.87	0.61	0.30	0.77
Lebanon	181.78	147.56	216.94	70.92	61.32	80.42	-0.32	-0.36	-0.29
Lesotho	200.05	195.78	204.57	345.67	327.66	364.05	0.08	-0.01	0.13
Liberia	84.46	86.9	82	43.08	34.48	51.54	0.32	-0.03	0.71
Libya	416.01	405.85	425.1	170.25	134.12	205.94	-0.56	-0.61	-0.53
Lithuania	225.64	128.08	334.21	461.63	185.16	783.16	1.05	0.47	1.32
Luxembourg	193.83	165.14	223.69	74.85	62.72	87.04	-0.45	-0.49	-0.41
Macedonia	495.97	532.14	460.23	611.61	649.21	573.69	0.33	0.32	0.34
Madagascar	140.88	107.35	174.52	111.23	73.43	149.26	0.47	0.30	0.54
Malawi	85.92	83.37	88.55	53.47	43.39	63.59	0.35	0.16	0.51
Malaysia	108.25	91.65	124.36	89.63	54.4	125.5	-0.12	-0.33	0.01
Maldives	99.99	118.53	82.38	35.39	31.8	38.95	-0.05	-0.24	0.21
Mali	86.26	99.84	72.39	51.22	53.92	48.57	0.05	-0.05	0.20
Malta	207.36	209.31	205.34	92.99	75.08	111.04	-0.55	-0.64	-0.46
Marshall Islands	242.61	247.79	237.65	279.16	237.65	319.24	0.39	0.14	0.63
Mauritania	66.18	58.55	73.85	39.13	33.02	45.15	0.08	0.00	0.14
Mauritius	84.64	65.05	104.31	116.43	67.82	166.24	0.35	0.00	0.58
Mexico	54.23	47.69	60.84	49.31	38.83	59.89	0.20	0.05	0.32
Moldova	82.16	58.81	107.77	148.62	71.46	232.04	1.20	0.51	1.56
Mongolia	173.94	169.6	178.39	194.25	108.08	282.17	0.63	0.09	1.06
Montenegro	392.97	356.86	429.39	511.53	412.13	613.29	0.26	0.15	0.35
Morocco	247.74	285.35	209.98	112.76	100.4	125.41	-0.32	-0.47	-0.10
Mozambique	71.76	65.68	78.4	66.68	49.09	85.08	0.70	0.34	0.98
Myanmar	150.69	112.56	190.37	127.36	105.69	150.02	0.48	0.76	0.29
Namibia	215.54	204.22	227.35	171.42	134.09	210.75	-0.08	-0.22	0.04
Nepal	21.66	16.35	27.04	28.06	20.55	36.04	1.96	2.08	1.84
Netherlands	208.78	176.16	242.14	99.29	82.96	115.89	-0.48	-0.51	-0.46
New Zealand	200.05	118.69	283.91	115.6	60.83	172.47	-0.31	-0.42	-0.23
Nicaragua	109.37	102.24	116.6	44.2	34.36	54.31	-0.28	-0.39	-0.18
Niger	71.32	71.53	71.1	39.66	31.59	47.6	0.30	0.03	0.56
Nigeria	70.86	64.66	76.94	42.97	27.66	57.71	0.10	-0.21	0.34
North Korea	108.9	89.13	129.84	75.79	62.23	89.96	-0.25	-0.21	-0.28
Northern Mariana Islands	119.89	123.76	116.41	117.17	77.91	156.71	0.23	-0.14	0.57
Norway	85.69	68.25	103.51	69.24	57.49	80.86	0.05	0.02	0.09
Oman	33.38	38.29	29.47	25.58	19.06	28.89	0.31	-0.10	0.64
Pakistan	22.82	14.48	30.56	47.86	41.71	53.68	2.16	3.56	1.52
Palestine	108.59	81.85	134.66	58.4	29.05	86.93	-0.18	-0.44	-0.06
Panama	68.71	51.68	85.36	97.81	66.64	128.8	0.51	0.38	0.58
Papua New Guinea	265.56	325.76	208.1	267.31	300.25	235.65	0.37	0.23	0.57
Paraguay	61.25	57.97	64.46	53.03	40.82	64.87	-0.07	-0.20	0.03
Peru	115.3	111.39	119.21	34.85	29.4	40.32	-0.38	-0.47	-0.29
Philippines	67.66	53.92	81.11	87.18	73.51	100.6	0.78	0.89	0.71
Poland	414.55	326.53	507.13	439.79	324.29	564.1	0.23	0.13	0.30
Portugal	120.8	114.99	127.06	89.18	69.37	110.3	-0.11	-0.30	0.08
Puerto Rico	174.92	165.26	185.19	104.35	92.43	117.23	-0.36	-0.45	-0.27
Qatar	67.18	67.06	67.23	26.19	22.05	27.74	-0.46	-0.55	-0.43
Romania	387.81	321.34	456.2	606.93	413.76	812.53	0.64	0.37	0.84
Russia	693.49	384.24	1,044.66	1,285.98	676.02	2,002.27	0.72	0.71	0.71
Rwanda	119.14	108.89	129.75	60.11	48.7	72.52	0.18	0.06	0.28
Saint Lucia	316.24	307.8	324.93	274.66	197.77	354.51	0.03	-0.24	0.29
Saint Vincent and the Grenadines	127.01	144.25	109.6	154.9	115.66	193.42	0.24	-0.13	0.69
Samoa	160.06	143.32	175.49	171.62	159.1	183.37	0.26	0.14	0.36
Sao Tome and Principe	93.05	79.6	106.75	80.88	63.39	98.5	0.55	0.40	0.65
Saudi Arabia	107.8	129.23	90.95	81.28	55.5	101.08	0.32	-0.19	0.84
Senegal	60.6	52.43	68.84	47.41	35.29	59.98	0.53	0.30	0.68
Serbia	340.97	313.32	369.37	408.21	349.83	469.43	0.17	0.08	0.25
Seychelles	64.89	31.83	98.1	54.72	23.31	85.3	-0.07	-0.22	-0.01
Sierra Leone	86.46	78.11	94.98	63.82	53.48	74.37	0.32	0.17	0.45
Singapore	147.54	110.82	183.29	64.45	48.27	81.21	-0.50	-0.51	-0.47

Slovakia	170.2	115.39	227.6	182.26	96.83	272.41	0.28	-0.05	0.50
Slovenia	1,017.07	1,002.22	1,032.82	450.6	433.39	468.12	-0.48	-0.51	-0.45
Solomon Islands	221.69	249.46	195.4	254.79	261.9	247.9	0.50	0.35	0.67
Somalia	121.32	111.17	131.66	78.17	69.21	87.22	-0.04	-0.08	-0.02
South Africa	283.73	292.9	274.34	288.18	261.38	315.93	-0.04	-0.15	0.09
South Korea	100.87	97.73	104	44.22	39.13	49.37	-0.49	-0.55	-0.44
South Sudan	111.34	104.92	117.82	76.79	67.19	86.36	0.10	0.02	0.17
Spain	191.44	182.71	200.53	144.9	110.67	180.02	-0.10	-0.32	0.13
Sri Lanka	251.85	159.19	342.71	70.3	49.71	92.44	-0.61	-0.58	-0.61
Sudan	338.65	352.65	324.76	183.17	173.14	193.13	0.05	-0.03	0.13
Suriname	208.94	238.93	179.92	171.46	150.42	192.43	-0.06	-0.25	0.18
Swaziland	233.75	237.36	229.72	335.02	319.09	351.28	0.04	-0.07	0.13
Sweden	173.78	122.77	226.03	117.48	79.52	155.52	-0.19	-0.26	-0.14
Switzerland	141.75	105.19	180.04	81.68	59.08	104.79	-0.28	-0.34	-0.24
Syria	233.77	192.78	273.45	143.13	103.95	181.35	-0.49	-0.42	-0.54
Taiwan	103.34	73.8	130.76	70.78	50.03	91.6	-0.32	-0.35	-0.30
Tajikistan	91.94	74.46	109.62	85.62	52.63	117.84	0.82	0.42	1.06
Tanzania	84.54	75.53	93.71	63.53	51.13	76.09	0.44	0.31	0.52
Thailand	328.91	254.69	404.5	55.46	35.39	76.11	-0.84	-0.86	-0.82
The Bahamas	265.48	205.57	326.22	264.28	160.52	372.5	0.10	-0.17	0.29
The Gambia	58.2	55.93	60.43	37.3	24.64	50.21	0.19	-0.13	0.47
Timor-Leste	211.96	223.6	200.62	95.36	93.39	97.26	0.16	0.10	0.23
Togo	72.07	66.72	77.55	55.16	36.21	74.55	0.16	-0.13	0.37
Tonga	44.57	19.69	68.84	55.59	19.71	91.27	0.38	0.16	0.40
Trinidad and Tobago	118.79	125.7	111.84	179.83	114.72	246.67	0.44	-0.09	1.00
Tunisia	178.02	216.22	140.28	87.71	71.04	104.78	-0.34	-0.54	-0.04
Turkey	153.37	114.16	193.54	58.45	39.1	78.5	-0.28	-0.36	-0.22
Turkmenistan	164.63	154.35	175.19	110.31	73.88	147.98	0.12	-0.15	0.35
Uganda	83.6	76.2	91.09	72.65	52.45	92.85	0.77	0.44	1.01
Ukraine	312.78	170.54	478.29	527.91	244.08	863.16	0.56	0.38	0.61
United Arab Emirates	176.81	136.59	197.71	149.97	81.72	174.84	0.06	-0.14	0.08
United Kingdom	168.6	149.2	189.12	92.94	70.92	115.5	-0.32	-0.43	-0.22
United States	262.51	182.38	346.79	189.47	134.37	246.44	-0.22	-0.24	-0.20
Uruguay	292.14	204.41	385.26	174.44	102.75	251.24	-0.33	-0.46	-0.24
Uzbekistan	30.01	26.12	33.98	36.92	26.96	47.22	0.65	0.44	0.80
Vanuatu	225.26	237.56	213.63	276.81	276.43	277.19	0.40	0.30	0.50
Venezuela	76.77	62.38	90.93	58.35	40.79	76.05	-0.26	-0.30	-0.25
Vietnam	74.69	42.7	107.66	69.62	44.19	95.6	0.35	0.59	0.22
Virgin Islands, U.S.	245.38	169.9	325.33	409.55	221.44	616.31	0.25	0.02	0.36
Yemen	352.14	390.42	313.17	156	154.11	157.85	-0.06	-0.15	0.05
Zambia	78.19	78.68	77.69	76.43	51.25	101.67	0.64	0.14	1.09
Zimbabwe	129	117.52	140.58	183.38	128.77	239.6	0.24	-0.06	0.49
**Cerebrovascular disease**									
Afghanistan	2,462.74	3,465.83	1,507.82	2,918.51	4,012.44	1,891.33	0.95	0.98	0.98
Albania	1,508.74	1,376.37	1,634.81	2,338.99	2,044.66	2,638.06	0.74	0.72	0.76
Algeria	1,200.56	1,300.37	1,103.20	1,148.91	1,232.80	1,066.17	0.37	0.34	0.39
American Samoa	1,388.31	954.51	1,798.93	941.46	885.05	997.58	-0.18	-0.02	-0.25
Andorra	816.31	727.28	910.18	661.73	528.1	800.97	-0.19	-0.25	-0.13
Angola	1,898.78	2,083.92	1,709.07	1,330.65	1,569.99	1,088.19	0.41	0.45	0.34
Antigua and Barbuda	1,890.78	1,861.03	1,922.59	1,185.79	1,085.90	1,294.72	-0.31	-0.36	-0.25
Argentina	1,732.81	1,522.08	1,951.84	967.16	866.19	1,072.52	-0.34	-0.35	-0.33
Armenia	1,583.71	1,654.27	1,508.93	1,922.23	1,771.27	2,096.10	0.35	0.21	0.52
Australia	1,017.37	1,046.05	988.52	633.2	648.28	618.08	-0.28	-0.31	-0.24
Austria	1,861.60	1,998.76	1,712.16	790.27	806.16	773.59	-0.50	-0.53	-0.45
Azerbaijan	1,712.75	1,751.08	1,672.75	1,602.92	1,488.70	1,718.49	0.41	0.30	0.54
Bahrain	597.65	643.71	563.77	305.33	331.11	289.55	-0.26	-0.28	-0.24
Bangladesh	1,473.20	1,671.45	1,283.77	2,161.09	2,011.02	2,308.26	2.41	2.02	2.89
Barbados	1,832.03	1,791.35	1,875.86	1,654.77	1,614.89	1,698.11	-0.14	-0.17	-0.12
Belarus	3,089.89	3,282.97	2,871.21	3,506.14	3,267.19	3,783.32	-0.02	-0.07	0.06
Belgium	1,577.92	1,655.71	1,496.52	933.42	926.92	940.18	-0.33	-0.37	-0.27
Belize	960.58	922.44	998.08	785.09	677.92	892.9	-0.03	-0.07	0.02

Benin	1,194.30	1,199.61	1,188.65	1,312.54	1,286.29	1,338.89	1.09	1.02	1.11
Bermuda	1,206.73	1,081.27	1,333.77	725.4	599.18	854.04	-0.24	-0.34	-0.15
Bhutan	1,018.50	1,078.92	962.36	814.87	698.01	915.48	0.80	0.58	0.98
Bolivia	1,468.41	1,642.22	1,295.80	1,007.28	1,066.36	948.43	0.48	0.41	0.57
Bosnia and Herzegovina	1,951.31	1,992.19	1,910.39	2,104.87	2,016.91	2,193.64	0.10	-0.01	0.20
Botswana	933.25	1,072.82	790.38	1,224.64	1,276.78	1,172.45	0.01	-0.07	0.13
Brazil	1,628.17	1,488.35	1,770.21	1,261.27	1,124.43	1,402.68	-0.02	-0.05	0.00
Brunei	742.95	730.49	754.11	694.94	651.01	736.28	-0.05	-0.10	0.00
Bulgaria	4,762.34	4,435.85	5,098.02	4,228.33	3,985.72	4,482.94	-0.13	-0.14	-0.13
Burkina Faso	767.3	862.83	667.33	667.02	765.8	566.96	0.61	0.61	0.59
Burundi	2,903.27	3,228.08	2,567.07	1,066.94	1,144.66	987.53	-0.18	-0.20	-0.15
Cambodia	2,040.81	2,078.25	2,000.70	1,671.26	1,596.15	1,750.14	1.00	0.93	1.08
Cameroon	1,279.82	1,338.60	1,220.30	1,279.14	1,273.15	1,285.13	0.33	0.26	0.37
Canada	770.15	767.48	772.88	579.13	571.73	586.63	-0.21	-0.26	-0.17
Cape Verde	1,648.50	1,474.95	1,840.43	1,344.74	1,280.74	1,410.45	0.26	0.36	0.18
Central African Republic	2,736.04	2,874.33	2,593.06	2,691.75	2,758.01	2,623.73	0.20	0.12	0.22
Chad	1,095.12	1,191.65	997.18	1,161.45	1,205.59	1,117.41	0.54	0.45	0.61
Chile	1,287.93	1,203.94	1,374.20	989.1	930.63	1,049.14	-0.12	-0.17	-0.07
China	2,579.52	2,334.22	2,812.22	2,515.87	1,836.80	3,155.52	0.45	0.28	0.56
Colombia	842.02	859.12	824.69	587.93	583.1	592.91	-0.07	-0.16	0.01
Comoros	1,737.27	1,796.93	1,677.99	926.43	995.76	858.36	0.15	0.20	0.11
Congo	2,241.25	2,541.46	1,939.48	1,563.71	2,122.19	1,006.06	-0.01	0.04	-0.18
Costa Rica	501.34	504.96	497.78	443.48	423.95	462.99	-0.07	-0.13	-0.02
Cote d'Ivoire	1,430.99	1,281.17	1,569.02	1,444.35	1,322.18	1,562.36	0.42	0.47	0.37
Croatia	3,239.86	3,214.16	3,267.17	2,477.27	2,482.04	2,472.16	-0.18	-0.20	-0.15
Cuba	1,313.79	1,300.83	1,326.55	1,445.12	1,339.77	1,549.48	0.05	-0.02	0.12
Cyprus	1,315.71	1,188.75	1,445.91	630.83	504.2	766.19	-0.44	-0.50	-0.39
Czech Republic	3,349.67	3,427.64	3,267.01	1,519.65	1,535.03	1,503.77	-0.44	-0.46	-0.41
Democratic Republic of the Congo	1,301.53	1,602.23	993.41	1,199.92	1,325.70	1,073.50	0.50	0.34	0.76
Denmark	1,692.45	1,695.82	1,688.98	972.53	922.91	1,022.94	-0.29	-0.34	-0.24
Djibouti	1,229.55	1,066.60	1,390.59	1,398.61	1,223.39	1,572.26	0.56	0.59	0.50
Dominica	1,106.35	1,181.31	1,030.83	1,257.67	1,130.42	1,386.93	0.03	-0.05	0.13
Dominican Republic	930.29	882.16	977.79	1,119.87	1,004.77	1,235.72	0.62	0.57	0.67
Ecuador	865.22	810.26	919.58	695.18	639.41	750.96	0.12	0.13	0.12
Egypt	2,441.07	2,159.36	2,720.84	1,485.27	1,245.66	1,719.92	0.05	0.04	0.05
El Salvador	1,101.71	1,047.17	1,158.83	532.61	518.35	548.73	-0.28	-0.27	-0.30
Equatorial Guinea	2,937.72	3,285.22	2,605.24	1,101.87	1,205.99	1,002.85	-0.28	-0.31	-0.26
Eritrea	1,634.74	1,810.14	1,457.12	1,331.11	1,603.78	1,059.43	0.42	0.48	0.28
Estonia	3,661.26	3,880.47	3,411.81	1,655.97	1,714.12	1,587.70	-0.42	-0.47	-0.37
Ethiopia	1,832.40	1,871.02	1,793.55	926.55	971.7	881.24	0.58	0.59	0.54
Federated States of Micronesia	2,114.58	1,828.63	2,388.30	1,735.61	1,489.84	1,969.69	0.10	0.09	0.11
Fiji	2,004.03	1,947.80	2,058.52	1,845.67	1,934.45	1,759.83	-0.20	-0.17	-0.23
Finland	1,835.53	1,893.61	1,773.91	1,031.70	1,004.28	1,059.97	-0.36	-0.41	-0.30
France	1,201.84	1,147.15	1,259.51	772.36	726.35	821.35	-0.29	-0.32	-0.26
Gabon	2,153.04	2,099.51	2,208.79	1,328.38	1,474.68	1,185.38	-0.08	0.02	-0.18
Georgia	3,751.89	3,639.86	3,875.45	3,863.16	3,198.79	4,589.85	0.04	-0.05	0.14
Germany	1,861.71	2,005.33	1,707.41	1,035.48	1,024.57	1,046.77	-0.37	-0.43	-0.31
Ghana	1,405.39	2,034.46	796.22	1,387.42	1,708.03	1,063.57	0.66	0.46	1.12
Greece	2,429.32	2,609.31	2,244.70	1,801.14	1,859.99	1,739.36	-0.25	-0.28	-0.21
Greenland	1,442.25	1,410.91	1,469.38	1,129.09	930	1,301.70	0.09	-0.06	0.22
Grenada	2,290.41	2,494.03	2,081.44	1,676.45	1,699.89	1,653.16	-0.20	-0.24	-0.15
Guam	1,116.81	980.2	1,236.13	1,584.16	1,416.70	1,746.92	0.05	0.06	0.04
Guatemala	624.51	623	626.04	547.91	553.24	542.35	0.61	0.69	0.54
Guinea	1,396.54	1,663.57	1,123.54	1,406.54	1,530.04	1,283.63	0.89	0.75	1.09
Guinea-Bissau	1,721.49	1,883.39	1,555.69	1,849.04	2,040.85	1,654.83	0.58	0.57	0.58
Guyana	2,643.62	2,316.06	2,981.78	2,225.54	2,011.39	2,437.62	-0.04	0.01	-0.08
Haiti	2,932.13	3,780.57	2,058.71	2,072.54	2,566.57	1,567.71	0.36	0.28	0.49
Honduras	2,997.95	2,912.57	3,082.73	1,229.44	1,098.21	1,360.67	-0.36	-0.43	-0.29
Hungary	3,792.41	3,624.54	3,974.01	1,916.47	1,850.47	1,989.23	-0.35	-0.39	-0.32
Iceland	877.19	883.83	870.62	501.81	430.06	575.1	-0.34	-0.43	-0.25
India	1,500.77	1,499.24	1,502.19	1,392.35	1,201.55	1,569.67	0.59	0.50	0.66

Indonesia	1,915.90	1,980.11	1,852.23	2,505.32	2,408.98	2,600.27	1.02	0.95	1.10
Iran	1,191.76	1,045.39	1,332.81	1,026.79	845.48	1,205.39	0.64	0.64	0.64
Iraq	1,567.40	1,598.66	1,536.71	1,251.08	1,213.50	1,287.74	-0.01	0.02	-0.03
Ireland	1,085.10	1,158.64	1,010.89	541.53	511.99	571.95	-0.39	-0.46	-0.31
Israel	946.78	954.61	938.92	499.08	462.47	536.31	-0.36	-0.42	-0.30
Italy	1,716.76	1,679.64	1,756.14	1,154.90	1,155.98	1,153.74	-0.26	-0.29	-0.24
Jamaica	1,790.05	1,929.83	1,646.69	1,690.74	1,694.51	1,686.94	-0.10	-0.13	-0.06
Japan	1,642.51	1,461.41	1,830.13	1,457.01	1,203.90	1,723.57	-0.21	-0.26	-0.18
Jordan	878.34	993.52	774.11	473.53	441.98	503.55	-0.19	-0.31	-0.05
Kazakhstan	2,366.07	2,515.01	2,207.80	2,459.73	2,245.02	2,689.79	0.13	0.02	0.27
Kenya	690.64	772.81	607.9	658.57	655.45	661.7	0.32	0.20	0.48
Kiribati	3,410.11	2,411.19	4,429.85	3,511.69	2,069.15	4,991.40	0.46	0.32	0.49
Kuwait	305.78	306.52	305.14	346.18	271.34	404.59	0.40	0.03	0.72
Kyrgyzstan	2,490.01	2,610.33	2,364.51	2,142.88	1,800.91	2,492.13	0.22	0.03	0.43
Laos	2,654.32	2,814.44	2,493.98	1,584.87	1,505.18	1,665.35	0.66	0.55	0.79
Latvia	4,200.14	4,554.32	3,792.19	3,081.11	3,213.27	2,927.18	-0.15	-0.20	-0.08
Lebanon	1,211.34	1,661.23	749.06	686.32	982.16	393.46	-0.01	-0.09	0.01
Lesotho	1,172.79	1,187.58	1,157.12	1,806.11	1,906.44	1,703.74	-0.04	-0.04	-0.06
Liberia	1,318.85	1,426.86	1,210.22	1,026.31	1,166.84	888.05	1.01	0.99	0.99
Libya	1,207.30	1,249.23	1,169.74	994.68	980	1,009.18	-0.12	-0.08	-0.16
Lithuania	2,182.68	2,297.78	2,054.58	2,248.74	2,246.16	2,251.73	0.03	-0.01	0.08
Luxembourg	2,100.58	2,191.03	2,006.44	680.84	679.57	682.11	-0.54	-0.58	-0.49
Macedonia	3,037.41	2,992.12	3,082.16	3,297.64	3,183.91	3,412.38	0.17	0.15	0.19
Madagascar	2,100.08	2,109.11	2,091.01	1,817.47	1,794.83	1,840.25	0.61	0.60	0.58
Malawi	902.99	1,105.23	693.63	651.23	670.24	632.15	0.56	0.34	0.91
Malaysia	1,210.83	1,148.17	1,271.64	1,109.89	880.57	1,343.46	-0.03	-0.13	0.05
Maldives	866.25	704.17	1,020.16	476.04	307.59	643.56	0.47	0.23	0.62
Mali	1,611.04	2,013.05	1,200.28	1,055.69	1,330.59	786.23	0.16	0.15	0.17
Malta	1,133.03	1,094.13	1,173.31	789.75	745.47	834.38	-0.30	-0.32	-0.29
Marshall Islands	1,917.70	1,533.56	2,285.06	1,781.16	1,452.31	2,098.73	0.12	0.12	0.12
Mauritania	1,183.81	1,340.45	1,026.24	841.34	1,012.99	672.06	0.30	0.34	0.22
Mauritius	2,015.28	1,593.07	2,439.22	1,501.72	1,204.83	1,805.93	-0.27	-0.28	-0.26
Mexico	546.53	554.74	538.23	526.37	510.97	541.92	0.27	0.18	0.36
Moldova	2,960.45	3,078.90	2,830.51	2,482.01	2,281.25	2,699.07	0.02	-0.08	0.14
Mongolia	1,779.76	1,805.92	1,752.99	3,193.35	2,765.05	3,630.35	1.63	1.61	1.70
Montenegro	3,068.62	3,350.70	2,784.09	3,410.65	3,499.93	3,319.25	0.07	0.04	0.12
Morocco	1,179.22	1,268.60	1,089.49	1,082.66	1,168.44	994.88	0.38	0.38	0.37
Mozambique	1,244.32	1,169.06	1,326.37	1,335.85	1,203.57	1,474.22	0.95	0.83	1.02
Myanmar	2,203.14	1,936.79	2,480.32	2,159.37	1,676.11	2,664.78	0.72	0.63	0.77
Namibia	1,113.16	1,191.29	1,031.64	797.71	748.74	849.3	-0.16	-0.25	-0.07
Nepal	1,054.38	1,027.95	1,081.20	1,035.05	880.17	1,199.75	1.24	1.10	1.36
Netherlands	1,162.99	1,187.20	1,138.24	785.11	767.83	802.68	-0.27	-0.32	-0.21
New Zealand	1,113.50	1,167.38	1,057.97	691.43	733.44	647.81	-0.26	-0.29	-0.23
Nicaragua	547.1	552.39	541.73	514.76	495.05	535.04	0.68	0.62	0.74
Niger	1,150.69	1,168.82	1,131.96	979.26	996.78	962.04	0.98	0.98	0.97
Nigeria	1,076.38	1,160.80	993.66	624.27	586.46	660.68	0.05	-0.07	0.19
North Korea	2,272.10	2,405.92	2,130.38	3,575.99	3,398.76	3,761.21	0.67	0.57	0.81
Northern Mariana Islands	1,143.45	1,030.72	1,244.78	668.6	439.44	899.39	-0.27	-0.42	-0.15
Norway	1,643.69	1,647.66	1,639.63	714.32	721.63	707.09	-0.43	-0.47	-0.40
Oman	575.1	738.66	444.62	421.14	444.03	409.53	0.25	0.09	0.54
Pakistan	1,325.17	1,168.27	1,470.79	1,491.18	1,230.20	1,738.23	0.69	0.67	0.70
Palestine	863.49	894.64	833.11	754.39	647.25	858.53	0.33	0.15	0.50
Panama	853.71	784.93	920.99	722.8	614.33	830.67	-0.10	-0.16	-0.05
Papua New Guinea	2,946.16	3,198.89	2,704.94	2,596.22	2,962.88	2,243.92	0.20	0.24	0.15
Paraguay	1,054.38	1,109.87	1,000.15	1,164.09	1,089.19	1,236.73	0.18	0.11	0.26
Peru	1,308.61	1,164.12	1,453.13	476.05	448.69	503.45	-0.25	-0.22	-0.27
Philippines	1,044.34	799.44	1,284.14	1,633.80	1,306.97	1,954.60	1.16	1.26	1.10
Poland	2,121.70	2,176.39	2,064.18	1,633.40	1,507.19	1,769.23	-0.11	-0.21	0.00
Portugal	3,567.95	3,404.14	3,744.12	1,617.73	1,488.47	1,755.58	-0.45	-0.49	-0.42
Puerto Rico	796.64	710.98	887.71	786.87	759.94	815.97	0.06	0.04	0.06
Qatar	566.69	655.08	523.05	271.05	276.03	269.18	-0.34	-0.43	-0.28

Romania	3,505.01	3,536.54	3,472.57	3,909.22	3,715.49	4,115.42	0.17	0.12	0.22
Russia	3,888.94	4,306.52	3,414.73	3,921.26	3,779.67	4,087.53	-0.06	-0.15	0.07
Rwanda	1,744.60	2,082.10	1,395.13	828.29	902.57	747.49	0.11	0.01	0.22
Saint Lucia	1,983.12	2,064.93	1,898.86	1,580.82	1,631.08	1,528.64	-0.06	-0.07	-0.05
Saint Vincent and the Grenadines	1,494.34	1,727.30	1,259.06	1,592.60	1,556.70	1,627.84	0.08	-0.03	0.24
Samoa	1,588.37	1,241.97	1,907.66	1,232.38	1,185.13	1,276.74	-0.09	-0.02	-0.13
Sao Tome and Principe	1,576.38	1,651.25	1,500.14	1,390.97	1,548.24	1,232.51	0.57	0.64	0.46
Saudi Arabia	595.17	624.31	572.24	504.62	419.92	569.7	0.48	0.27	0.65
Senegal	1,113.91	1,140.97	1,086.60	1,171.01	1,287.15	1,050.62	1.04	1.15	0.86
Serbia	3,433.03	3,615.48	3,245.69	3,392.69	3,459.74	3,322.37	-0.04	-0.08	0.01
Seychelles	1,497.01	1,227.22	1,767.96	1,013.42	771.41	1,249.01	-0.25	-0.33	-0.19
Sierra Leone	1,285.05	1,086.90	1,487.30	1,369.59	1,510.07	1,226.23	0.91	1.37	0.53
Singapore	1,201.90	1,186.64	1,216.76	731.69	706.02	758.26	-0.30	-0.34	-0.26
Slovakia	2,245.31	2,165.22	2,329.19	1,455.52	1,341.80	1,575.51	-0.22	-0.30	-0.15
Slovenia	2,166.84	2,107.85	2,229.39	1,250.00	1,199.71	1,301.20	-0.32	-0.36	-0.29
Solomon Islands	2,740.44	2,693.89	2,784.50	2,451.40	2,465.97	2,437.29	0.17	0.18	0.15
Somalia	2,109.73	2,427.91	1,785.87	1,493.83	1,728.18	1,257.30	0.05	0.05	0.05
South Africa	1,268.30	1,538.12	992.2	1,223.38	1,295.76	1,148.44	-0.09	-0.20	0.09
South Korea	2,342.49	2,291.48	2,393.28	1,261.92	1,124.73	1,400.78	-0.38	-0.45	-0.32
South Sudan	1,506.94	1,719.02	1,292.87	1,277.13	1,461.06	1,093.75	0.35	0.35	0.35
Spain	1,589.66	1,569.06	1,611.13	784.19	778.18	790.36	-0.41	-0.44	-0.38
Sri Lanka	712.93	551.35	871.41	1,097.65	895.87	1,314.55	1.17	1.17	1.17
Sudan	2,255.21	2,522.39	1,990.12	1,618.80	1,729.30	1,509.11	0.39	0.35	0.44
Suriname	1,606.23	1,603.13	1,609.22	1,808.75	1,670.74	1,946.25	0.29	0.24	0.33
Swaziland	1,029.12	1,109.24	939.51	1,439.18	1,540.71	1,335.50	0.02	-0.03	0.06
Sweden	1,547.93	1,539.99	1,556.06	916.08	915.17	916.98	-0.29	-0.32	-0.26
Switzerland	1,209.51	1,197.96	1,221.62	571.44	575.22	567.58	-0.41	-0.44	-0.39
Syria	1,984.83	1,722.03	2,239.20	1,152.91	959.73	1,341.38	-0.52	-0.40	-0.58
Taiwan	1,563.48	1,289.63	1,817.69	1,047.66	817.77	1,278.33	-0.34	-0.39	-0.30
Tajikistan	1,454.18	1,530.32	1,377.12	1,101.20	1,071.28	1,130.42	0.48	0.40	0.58
Tanzania	989.7	1,088.86	888.78	787.87	812.13	763.32	0.51	0.41	0.60
Thailand	1,349.43	1,119.42	1,583.66	1,507.75	1,080.98	1,946.78	0.08	-0.02	0.15
The Bahamas	1,281.82	1,242.80	1,321.37	1,281.40	1,235.74	1,329.02	0.10	0.05	0.14
The Gambia	962.3	1,061.42	864.6	778.22	754.88	802.01	0.50	0.40	0.64
Timor-Leste	1,467.43	1,590.75	1,347.22	1,125.90	1,254.20	1,001.56	1.00	1.09	0.91
Togo	1,250.40	1,364.29	1,133.55	1,195.89	1,104.33	1,289.61	0.44	0.29	0.62
Tonga	1,181.18	1,343.06	1,023.23	1,127.97	1,138.23	1,117.76	0.05	-0.02	0.15
Trinidad and Tobago	1,662.30	1,603.86	1,721.17	1,563.16	1,378.15	1,753.09	-0.11	-0.14	-0.08
Tunisia	1,326.70	1,126.04	1,524.92	1,153.17	933.93	1,377.57	0.17	0.16	0.16
Turkey	986.68	1,068.30	903.06	737.05	682.47	793.56	0.42	0.20	0.69
Turkmenistan	1,762.90	1,759.78	1,766.11	2,017.04	1,691.75	2,353.43	0.92	0.71	1.13
Uganda	1,304.15	1,544.60	1,060.54	898.62	850.2	947.08	0.40	0.14	0.75
Ukraine	3,864.24	4,177.88	3,499.27	3,367.61	3,283.09	3,467.43	-0.19	-0.24	-0.12
United Arab Emirates	1,013.66	1,115.96	960.53	931.52	883.03	949.19	0.15	0.13	0.20
United Kingdom	1,780.91	1,899.91	1,655.03	955.37	981.15	928.95	-0.34	-0.38	-0.29
United States	901	921.39	879.55	692.92	682.35	703.85	-0.17	-0.24	-0.10
Uruguay	2,091.97	2,018.25	2,170.24	1,514.15	1,556.36	1,468.94	-0.19	-0.17	-0.22
Uzbekistan	1,543.44	1,572.17	1,514.06	1,492.15	1,324.26	1,665.54	0.30	0.17	0.43
Vanuatu	2,769.02	2,559.73	2,966.92	2,645.73	2,624.37	2,666.55	0.09	0.14	0.04
Venezuela	789.74	792.18	787.33	775.25	691.89	859.32	-0.05	-0.07	-0.02
Vietnam	1,814.50	1,541.19	2,096.23	2,055.30	1,563.46	2,557.72	0.64	0.56	0.68
Virgin Islands, U.S.	968.75	867.57	1,075.92	1,527.43	1,243.97	1,839.00	0.18	0.12	0.23
Yemen	2,146.88	2,523.55	1,763.30	1,641.28	1,902.96	1,384.97	0.61	0.61	0.63
Zambia	934.49	1,215.79	648.43	1,013.91	958.57	1,069.40	0.82	0.37	1.63
Zimbabwe	906.74	1,045.02	767.29	1,023.28	965.47	1,082.79	-0.01	-0.20	0.23
**Endocarditis**									
Afghanistan	35.83	51.91	20.52	44.64	63.63	26.81	1.05	1.10	1.07
Albania	19.83	20.38	19.31	20.31	17.69	22.98	0.15	0.00	0.30
Algeria	81.68	90.19	73.38	72.49	82.48	62.63	0.27	0.29	0.23
American Samoa	57	47.53	65.96	69.79	77.68	61.94	0.49	0.72	0.26
Andorra	18.26	18.58	17.93	20.11	18.39	21.9	0.11	0.02	0.21

Angola	76.29	81.6	70.86	62.57	66.77	58.32	0.66	0.59	0.74
Antigua and Barbuda	56.08	48.86	63.79	37.98	27.97	48.9	-0.25	-0.38	-0.14
Argentina	63.87	79.01	48.15	57.78	68.63	46.46	0.07	-0.01	0.18
Armenia	7.8	6.98	8.68	7.88	6	10.04	0.13	-0.03	0.27
Australia	19.74	17.79	21.7	22.49	19.94	25.04	0.32	0.24	0.40
Austria	47.77	54.42	40.52	21.85	24.36	19.22	-0.46	-0.48	-0.43
Azerbaijan	25.26	22	28.66	26.37	19.33	33.48	0.57	0.34	0.75
Bahrain	43.22	48.69	39.2	21.63	23.95	20.21	-0.27	-0.31	-0.24
Bangladesh	5.11	6	4.25	9.63	10.95	8.34	3.38	3.58	3.25
Barbados	70.01	68.52	71.62	77.46	72.89	82.44	0.05	-0.02	0.12
Belarus	17.76	11.66	24.68	27.74	14.57	43.03	0.35	0.17	0.40
Belgium	41.13	50.22	31.61	55.53	62.22	48.57	0.54	0.39	0.78
Belize	35.85	44.03	27.79	25.98	30.24	21.69	-0.14	-0.13	-0.11
Benin	77.98	79.04	76.85	53.25	53.66	52.84	0.31	0.30	0.31
Bermuda	45.76	33.8	57.86	26.67	14.73	38.85	-0.26	-0.48	-0.11
Bhutan	7.07	8.1	6.11	14.1	15.08	13.27	3.48	3.54	3.52
Bolivia	110.49	93.66	127.22	64.48	55.11	73.83	0.26	0.27	0.25
Bosnia and Herzegovina	9.76	7.05	12.48	10.69	7.04	14.38	0.11	-0.02	0.21
Botswana	35.19	38.49	31.82	37.19	34.76	39.61	-0.21	-0.32	-0.07
Brazil	31.55	27.94	35.22	35.62	30.17	41.25	0.42	0.35	0.48
Brunei	34.73	23.41	44.85	36.65	22.56	49.9	0.08	-0.02	0.14
Bulgaria	38.01	29.72	46.52	29.69	21.03	38.77	-0.24	-0.32	-0.17
Burkina Faso	73.47	78.09	68.64	61.69	64.24	59.12	0.56	0.50	0.62
Burundi	91.93	93.15	90.67	49.53	52.04	46.97	0.21	0.27	0.14
Cambodia	94.55	99.48	89.27	58	56.51	59.55	0.50	0.43	0.59
Cameroon	81.57	84.88	78.23	62.21	59.22	65.21	0.02	-0.06	0.10
Canada	19.45	18.73	20.19	24.73	22.25	27.25	0.33	0.18	0.47
Cape Verde	59.9	61.99	57.59	46.34	47.69	44.96	0.20	0.21	0.20
Central African Republic	67.62	68.69	66.53	69.72	64.72	74.85	0.26	0.12	0.37
Chad	66.71	72.38	60.97	62.69	64.82	60.57	0.38	0.31	0.45
Chile	23.13	25.08	21.12	26.33	26.22	26.44	0.31	0.13	0.52
China	14.59	12.49	16.58	9.85	7.44	12.12	0.00	-0.03	0.01
Colombia	27.45	25.59	29.33	27.8	22.32	33.45	0.35	0.08	0.61
Comoros	80.72	77.43	83.98	46.85	47.58	46.14	0.25	0.32	0.18
Congo	65.49	68.48	62.49	58.93	68.24	49.64	0.28	0.25	0.26
Costa Rica	25	18.76	31.15	29.85	23.98	35.71	0.25	0.33	0.21
Cote d'Ivoire	74.85	72.55	76.98	58.21	53.56	62.69	0.10	0.06	0.13
Croatia	18.36	15.61	21.29	14.66	11.82	17.72	-0.14	-0.22	-0.07
Cuba	22.61	24.32	20.93	23.3	23.47	23.14	-0.01	-0.08	0.06
Cyprus	44.64	47.73	41.47	32.2	30.13	34.41	-0.16	-0.26	-0.04
Czech Republic	20.54	16.62	24.69	27.69	21.47	34.11	0.67	0.55	0.77
Democratic Republic of the Congo	47.32	54.02	40.45	43.49	48.24	38.7	0.49	0.44	0.56
Denmark	21.82	24.28	19.29	27.14	24.03	30.29	0.54	0.20	0.98
Djibouti	81.15	74.03	88.19	71.09	57.52	84.55	0.20	0.07	0.27
Dominica	47.45	57.02	37.8	52.77	55.77	49.73	0.01	-0.03	0.10
Dominican Republic	53.89	65.24	42.68	32.58	34.72	30.42	-0.18	-0.27	-0.06
Ecuador	41.3	36.91	45.65	27.79	22.27	33.32	-0.06	-0.14	0.00
Egypt	154.42	218.85	90.44	99.68	140.53	59.68	0.12	0.16	0.10
El Salvador	51.78	51.48	52.1	29.6	25.54	34.2	-0.15	-0.27	-0.03
Equatorial Guinea	90.29	98.43	82.51	58.28	55.49	60.94	0.24	0.07	0.42
Eritrea	69.68	70.24	69.12	58.45	61.33	55.58	0.47	0.47	0.43
Estonia	31.39	17.67	47	31.79	21.64	43.71	0.29	0.46	0.26
Ethiopia	72.08	74.89	69.25	41.47	40.35	42.59	0.80	0.66	0.92
Federated States of Micronesia	96.71	115.82	78.42	100.86	119.66	82.96	0.40	0.38	0.43
Fiji	187.98	218.8	158.12	240.28	269.46	212.06	0.11	0.03	0.20
Finland	18.79	16.77	20.94	14.97	12.47	17.54	-0.09	-0.17	-0.01
France	36.49	38.61	34.26	47.62	44.4	51.05	0.45	0.24	0.70
Gabon	66.71	64.36	69.16	57.03	58.05	56.04	0.28	0.31	0.23
Georgia	18.51	13.66	23.86	24.85	16.24	34.27	0.35	0.28	0.38
Germany	48.85	57.87	39.15	44.63	46.17	43.04	0.03	-0.10	0.24
Ghana	77.9	100.42	56.1	67.66	68.93	66.37	0.47	0.20	0.91

Greece	29.39	42.77	15.67	33.2	43.87	22	0.14	0.03	0.43
Greenland	33.24	19.32	45.3	26.51	17.09	34.67	0.11	0.26	0.05
Grenada	95.57	110.7	80.05	90.38	100.49	80.34	0.04	0.02	0.08
Guam	34.34	33.54	35.04	87.45	83.37	91.42	0.89	0.83	0.92
Guatemala	52.74	58.6	46.8	33.56	28.62	38.72	0.17	-0.07	0.47
Guinea	83.32	92.2	74.24	49.31	51.77	46.87	0.12	0.08	0.16
Guinea-Bissau	86.02	93.26	78.61	76.39	81.93	70.78	0.33	0.31	0.36
Guyana	80.54	104.2	56.12	74.86	82.7	67.09	0.05	-0.08	0.34
Haiti	86.17	97.82	74.18	55.01	64.85	44.97	0.23	0.25	0.18
Honduras	46.15	47.59	44.73	29.6	22.94	36.25	0.00	-0.27	0.30
Hungary	34.96	31.5	38.71	34.16	30.16	38.56	0.25	0.14	0.35
Iceland	12.78	12.94	12.63	13.7	10.93	16.53	0.24	0.00	0.50
India	6.49	6.85	6.15	15.28	15.12	15.44	3.05	3.14	2.98
Indonesia	42	34.84	49.09	44.76	41.39	48.08	0.64	0.90	0.46
Iran	19.02	15.7	22.22	16.58	11.86	21.24	0.66	0.53	0.73
Iraq	105.76	157.44	55.01	81.62	118.22	45.91	-0.04	0.01	-0.03
Ireland	24.08	28.7	19.42	18.45	15.77	21.21	-0.06	-0.32	0.34
Israel	18.44	19.89	16.98	20.6	17.37	23.88	0.35	0.04	0.72
Italy	18.93	19.55	18.28	35.82	37.89	33.62	1.07	1.01	1.13
Jamaica	43.8	47.89	39.6	49.31	51.38	47.22	0.07	0.06	0.10
Japan	31.51	33.74	29.19	27.23	27.55	26.9	-0.23	-0.26	-0.20
Jordan	42.22	56.88	28.96	25.56	28.96	22.33	-0.09	-0.20	0.13
Kazakhstan	18.75	17.47	20.12	22.15	16.6	28.11	0.28	0.09	0.46
Kenya	33.01	34.51	31.51	32.82	31.18	34.47	0.38	0.28	0.49
Kiribati	154.9	164.79	144.79	163.47	165.79	161.08	0.49	0.54	0.47
Kuwait	18.34	28.66	9.3	14.72	18.69	11.63	-0.01	-0.24	0.62
Kyrgyzstan	11.71	9.19	14.34	9.23	6.35	12.17	0.11	0.03	0.15
Laos	125.27	127.05	123.49	81.36	77.17	85.59	0.80	0.75	0.85
Latvia	25.34	14.82	37.46	22.87	15.24	31.75	0.05	0.16	0.01
Lebanon	49.42	54.25	44.46	29.31	35.37	23.31	0.03	0.01	0.01
Lesotho	32.54	36.03	28.85	47.26	45.15	49.42	-0.10	-0.25	0.09
Liberia	86.28	88.36	84.18	42.2	45.08	39.36	0.27	0.24	0.27
Libya	101.21	110.33	93.04	60.6	64.86	56.4	-0.36	-0.31	-0.41
Lithuania	24.13	15.23	34.02	30.44	19.77	42.84	0.27	0.32	0.24
Luxembourg	43.59	51.45	35.41	40.46	44.26	36.65	0.33	0.16	0.57
Macedonia	13.2	14.06	12.35	11.9	10.55	13.26	-0.03	-0.19	0.16
Madagascar	104.66	97.34	112	90.17	77.25	103.17	0.61	0.50	0.66
Malawi	56.1	60.68	51.36	41.05	40.21	41.9	0.58	0.47	0.71
Malaysia	66.97	46.66	86.68	93.71	55.97	132.16	0.48	0.36	0.52
Maldives	65.95	72.41	59.81	22.14	21.94	22.34	-0.10	-0.15	-0.04
Mali	85.69	101.86	69.16	59.58	71.17	48.22	0.23	0.23	0.25
Malta	28.5	33.07	23.76	38.39	37.87	38.91	0.35	0.15	0.65
Marshall Islands	86.74	103.5	70.7	101.41	117.09	86.27	0.41	0.34	0.48
Mauritania	59.71	63.4	56	43.09	47.9	38.35	0.32	0.35	0.28
Mauritius	29.01	33.03	24.97	39.1	31.47	46.91	0.32	-0.09	0.87
Mexico	21.76	22.78	20.73	19.41	16.78	22.08	0.18	-0.05	0.43
Moldova	10.51	7.57	13.73	17.34	10.05	25.23	1.00	0.65	1.19
Mongolia	7.56	6.85	8.29	8.08	5.71	10.5	0.56	0.42	0.65
Montenegro	11.95	10.35	13.57	13.6	11.04	16.22	0.10	0.06	0.13
Morocco	135.98	160.94	110.93	117.91	142.25	92.99	0.30	0.32	0.26
Mozambique	47.51	51.86	42.76	46.13	42.07	50.39	0.77	0.45	1.14
Myanmar	66.21	53.37	79.58	68.97	63.57	74.61	0.82	1.24	0.54
Namibia	34.54	35.66	33.38	25.21	21.68	28.94	-0.15	-0.28	-0.02
Nepal	5.08	4.7	5.46	9	7.56	10.53	3.05	2.93	3.10
Netherlands	31.52	35.89	27.04	60.2	60.36	60.04	1.08	0.77	1.49
New Zealand	18.22	16.27	20.23	10.89	8.73	13.13	-0.28	-0.39	-0.18
Nicaragua	29.25	30.38	28.11	20	18.72	21.3	0.22	0.11	0.33
Niger	72.42	79.93	64.66	40.44	45.82	35.14	0.30	0.34	0.27
Nigeria	60.78	60.72	60.83	33.49	29.85	36.99	0.00	-0.09	0.09
North Korea	21.43	17.04	26.09	15.81	13.75	17.96	-0.20	-0.09	-0.28
Northern Mariana Islands	34.63	57.18	14.37	30.5	43.05	17.86	0.11	0.02	0.45

Norway	25.15	27.56	22.68	24.59	24.23	24.94	0.27	0.07	0.53
Oman	69.39	68.75	69.91	39.81	35.17	42.17	-0.02	-0.07	0.01
Pakistan	6.27	4.91	7.53	14.61	13	16.12	2.51	3.20	2.08
Palestine	47.63	55.41	40.05	32.39	35.96	28.92	0.03	0.04	0.05
Panama	25.39	21.36	29.34	30.9	26.71	35.06	0.29	0.34	0.25
Papua New Guinea	79.98	106.35	54.81	91.37	121.88	62.05	0.56	0.54	0.57
Paraguay	31.66	32.96	30.39	35.83	32.74	38.83	0.21	0.12	0.30
Peru	66.3	66.81	65.78	32.01	31.82	32.21	0.00	-0.04	0.03
Philippines	26.38	29.33	23.49	43.15	43.72	42.6	1.26	1.06	1.50
Poland	31.04	23.96	38.49	22.71	13.31	32.84	-0.15	-0.37	0.00
Portugal	34.2	35.85	32.42	42.02	41.18	42.91	0.48	0.34	0.64
Puerto Rico	42.65	34.88	50.91	44.14	36.53	52.35	0.11	0.02	0.19
Qatar	11.71	10.39	12.37	6.69	6.84	6.63	-0.21	-0.10	-0.25
Romania	13.51	8.05	19.13	14.58	9.24	20.27	0.13	0.22	0.09
Russia	7.77	6.98	8.66	41.39	23.83	62.01	3.95	2.32	5.38
Rwanda	82.12	87.75	76.3	48.16	48.61	47.69	0.37	0.31	0.43
Saint Lucia	94.51	104.86	83.85	77.14	73.71	80.7	-0.03	-0.17	0.14
Saint Vincent and the Grenadines	114.79	100.14	129.59	42.03	47.52	36.64	-0.63	-0.49	-0.73
Samoa	78.71	95.25	63.47	83.64	106.17	62.48	0.24	0.14	0.28
Sao Tome and Principe	119.17	123.98	114.27	76.78	78.26	75.29	0.15	0.12	0.19
Saudi Arabia	27.87	35.79	21.64	18.88	19.92	18.08	0.19	0.05	0.38
Senegal	53.87	58.3	49.41	42.05	44.77	39.23	0.53	0.49	0.54
Serbia	17.16	13.43	20.99	19	15.13	23.06	0.08	0.09	0.08
Seychelles	61.89	55.55	68.25	54.73	44.8	64.4	-0.02	-0.14	0.08
Sierra Leone	87.04	86.02	88.09	57.84	59.65	55.99	0.19	0.19	0.18
Singapore	18.66	14.16	23.04	25.91	21.62	30.36	0.60	0.70	0.56
Slovakia	23.66	18.32	29.26	19.23	12.94	25.86	-0.03	-0.20	0.11
Slovenia	22.21	17.36	27.36	13.28	8.89	17.74	-0.30	-0.42	-0.21
Solomon Islands	87.95	116.18	61.22	106.36	139.63	74.11	0.57	0.54	0.59
Somalia	85.61	93.12	77.97	56.58	62.48	50.62	-0.02	-0.01	-0.04
South Africa	45.01	50.88	39	37.54	36.83	38.27	-0.21	-0.31	-0.07
South Korea	47.01	54.76	39.29	17.71	17.07	18.35	-0.57	-0.65	-0.45
South Sudan	80.45	89.9	70.92	57.16	62.48	51.86	0.13	0.10	0.17
Spain	46.21	50.74	41.49	42.9	46.59	39.11	0.11	0.03	0.19
Sri Lanka	68.74	60.51	76.81	57.28	40.98	74.81	0.17	-0.09	0.40
Sudan	69.89	81.59	58.28	54.54	63.08	46.06	0.52	0.53	0.51
Suriname	42.36	53.26	31.8	35.52	38.11	32.93	-0.04	-0.15	0.14
Swaziland	37.07	41.85	31.73	46.07	43.38	48.83	-0.09	-0.28	0.14
Sweden	24.58	26.6	22.5	26.11	27.35	24.87	0.28	0.18	0.39
Switzerland	23.25	23.41	23.08	61.25	58.82	63.73	2.27	1.95	2.62
Syria	43.18	56.21	30.58	28.27	39.2	17.61	-0.46	-0.25	-0.60
Taiwan	33.9	24.36	42.76	49.61	40.8	58.45	0.44	0.60	0.37
Tajikistan	10.53	8.94	12.14	8.85	6.82	10.83	0.64	0.53	0.71
Tanzania	55.27	55.07	55.47	45.41	44.16	46.68	0.56	0.53	0.57
Thailand	75.96	78.05	73.83	100.37	90.15	110.87	0.28	0.17	0.40
The Bahamas	59.44	63.01	55.83	59.12	59.82	58.4	0.10	0.00	0.19
The Gambia	60.8	67.15	54.54	35.63	36.47	34.77	0.09	0.07	0.12
Timor-Leste	94.36	98.61	90.22	57.14	57.79	56.51	0.56	0.54	0.59
Togo	67.8	73.99	61.46	47.03	46.08	48	0.05	0.00	0.12
Tonga	84.33	108.2	61.04	105.73	124.89	86.66	0.38	0.34	0.50
Trinidad and Tobago	44.74	42.31	47.19	47.42	41.86	53.12	0.01	-0.01	0.02
Tunisia	80.02	89.13	71.02	64.77	66.02	63.49	0.09	0.04	0.15
Turkey	44.94	48.63	41.16	30.29	34.06	26.39	0.28	0.32	0.23
Turkmenistan	20.48	15.87	25.22	20.37	14.42	26.52	0.66	0.61	0.68
Uganda	53.08	56.8	49.3	51.2	47.17	55.23	0.96	0.73	1.21
Ukraine	20.4	14.1	27.74	28.86	18.33	41.3	0.31	0.25	0.33
United Arab Emirates	37.24	38.22	36.73	45.01	36.72	48.03	0.51	0.38	0.59
United Kingdom	23.67	25.79	21.43	31.84	31.37	32.31	0.66	0.46	0.92
United States	34.77	31.49	38.22	42.59	38.63	46.68	0.32	0.26	0.37
Uruguay	64.85	74.36	54.76	73.73	76.77	70.48	0.27	0.11	0.49
Uzbekistan	7.93	7.01	8.86	7.88	5.63	10.2	0.33	0.12	0.49

Vanuatu	89.71	113.87	66.86	113.16	139.34	87.64	0.44	0.37	0.52
Venezuela	17.07	16.05	18.07	11.96	9.72	14.23	-0.32	-0.35	-0.29
Vietnam	34.78	24.1	45.79	36.6	28.46	44.91	0.52	0.81	0.35
Virgin Islands, U.S.	61.42	65.42	57.19	88.22	82.05	95	0.07	-0.02	0.20
Yemen	73.86	90.28	57.14	49.41	60.53	38.51	0.41	0.44	0.41
Zambia	53.32	60.46	46.05	53.08	46.34	59.84	0.67	0.34	1.08
Zimbabwe	13	6.16	19.9	17.72	5.87	29.92	0.19	-0.18	0.32
**Hemorrhagic and other stroke**									
Afghanistan	1,562.82	2,217.20	939.86	1,874.68	2,630.80	1,164.69	0.97	1.03	0.96
Albania	1,232.24	1,106.44	1,352.06	1,898.17	1,648.26	2,152.11	0.73	0.72	0.74
Algeria	836.34	899.9	774.35	663.69	705.12	622.82	0.13	0.11	0.16
American Samoa	1,021.66	687.16	1,338.28	682.51	638.05	726.74	-0.19	-0.02	-0.27
Andorra	394.87	344.09	448.41	316.2	239.07	396.58	-0.20	-0.29	-0.13
Angola	1,445.25	1,541.42	1,346.70	1,004.32	1,157.08	849.58	0.39	0.44	0.32
Antigua and Barbuda	1,007.41	933.99	1,085.93	752.58	656.95	856.86	-0.17	-0.23	-0.12
Argentina	774.83	681.62	871.7	569.61	487.72	655.06	-0.13	-0.18	-0.08
Armenia	808.51	808.74	808.27	904.55	806.9	1,017.03	0.25	0.12	0.38
Australia	551.75	580	523.35	346.95	358.42	335.45	-0.27	-0.32	-0.22
Austria	792.04	810.71	771.69	386.89	384.59	389.31	-0.42	-0.44	-0.39
Azerbaijan	1,269.19	1,300.22	1,236.81	1,164.94	1,093.96	1,236.74	0.38	0.28	0.50
Bahrain	398.78	404.76	394.39	165.9	168.02	164.59	-0.40	-0.42	-0.38
Bangladesh	956.94	1,082.42	837.04	1,405.29	1,273.18	1,534.85	2.41	1.95	2.97
Barbados	860.28	802.56	922.48	938.73	874.34	1,008.72	0.04	0.00	0.07
Belarus	1,209.57	1,215.32	1,203.05	1,202.80	1,028.67	1,404.81	-0.14	-0.21	-0.06
Belgium	764.92	779.54	749.62	506.26	491.61	521.48	-0.25	-0.29	-0.19
Belize	582.04	547.97	615.54	506.41	435.69	577.54	0.04	0.00	0.07
Benin	851.71	792.32	914.9	938.76	864.59	1,013.22	1.10	1.06	1.08
Bermuda	638.51	555.68	722.38	405.13	315.22	496.77	-0.19	-0.32	-0.09
Bhutan	737.67	785.24	693.46	534.91	454.37	604.26	0.63	0.41	0.82
Bolivia	1,007.72	1,125.22	891.03	601.81	632.5	571.23	0.29	0.22	0.38
Bosnia and Herzegovina	1,145.13	1,129.97	1,160.31	583.98	553.56	614.69	-0.48	-0.52	-0.45
Botswana	620.44	701.23	537.73	802.56	808.67	796.44	-0.01	-0.10	0.12
Brazil	1,268.43	1,175.24	1,363.09	957.47	869.4	1,048.49	-0.05	-0.07	-0.03
Brunei	517.47	516.01	518.78	455.84	430.16	479.99	-0.10	-0.15	-0.05
Bulgaria	2,753.23	2,512.94	3,000.28	1,939.36	1,747.97	2,140.22	-0.31	-0.34	-0.29
Burkina Faso	518.47	569.06	465.53	462.55	512.15	412.3	0.66	0.63	0.66
Burundi	2,125.61	2,303.94	1,941.03	740.35	770.76	709.28	-0.22	-0.24	-0.19
Cambodia	1,647.85	1,636.66	1,659.85	1,254.80	1,184.59	1,328.53	0.86	0.82	0.91
Cameroon	913.53	905.05	922.12	887.57	838.64	936.46	0.29	0.23	0.32
Canada	325.65	331.57	319.63	232.8	230.4	235.24	-0.25	-0.31	-0.20
Cape Verde	1,095.18	909.14	1,300.92	885.4	784.19	989.32	0.25	0.35	0.17
Central African Republic	2,000.66	2,035.45	1,964.68	1,970.66	1,945.42	1,996.58	0.20	0.11	0.23
Chad	766.58	788.28	744.56	831.22	819.18	843.22	0.58	0.49	0.63
Chile	554.05	523.13	585.81	528.69	483.02	575.59	0.10	-0.01	0.19
China	1,896.61	1,697.98	2,085.04	1,624.46	1,156.02	2,065.71	0.27	0.11	0.37
Colombia	596.94	615.01	578.63	418.2	417.87	418.54	-0.06	-0.16	0.02
Comoros	1,288.50	1,254.66	1,322.12	671.22	688.23	654.52	0.13	0.19	0.07
Congo	1,579.15	1,727.26	1,430.27	1,089.59	1,437.09	742.61	-0.02	0.04	-0.18
Costa Rica	274.86	281.95	267.88	278.08	263.51	292.63	0.06	-0.03	0.15
Cote d'Ivoire	1,054.68	875.08	1,220.16	1,046.15	899.84	1,187.47	0.40	0.46	0.33
Croatia	1,776.81	1,728.68	1,827.96	1,164.86	1,118.53	1,214.58	-0.29	-0.33	-0.26
Cuba	676.3	674.05	678.51	705.96	627.12	784.05	0.00	-0.11	0.11
Cyprus	749.82	679.18	822.27	352.71	273.14	437.77	-0.45	-0.53	-0.39
Czech Republic	1,347.93	1,330.62	1,366.27	555.38	525.21	586.52	-0.49	-0.53	-0.45
Democratic Republic of the Congo	938.24	1,110.90	761.32	847.52	903.38	791.37	0.47	0.32	0.69
Denmark	920.34	923.67	916.91	525.99	499.28	553.13	-0.29	-0.35	-0.24
Djibouti	921.76	752.52	1,088.99	1,000.84	817.93	1,182.13	0.48	0.50	0.44
Dominica	591.74	606.24	577.12	732.06	617.74	848.19	0.12	0.01	0.23
Dominican Republic	562.46	544.26	580.42	687.76	602.97	773.11	0.65	0.52	0.76
Ecuador	518.38	496.63	539.88	410.07	385.86	434.28	0.11	0.11	0.11
Egypt	1,831.83	1,513.81	2,147.66	1,035.03	788.57	1,276.39	-0.02	-0.06	-0.01

El Salvador	738.51	711.33	766.97	345.97	342.45	349.94	-0.30	-0.29	-0.33
Equatorial Guinea	2,088.41	2,240.42	1,942.98	808.83	863.32	757	-0.26	-0.28	-0.25
Eritrea	1,250.78	1,331.43	1,169.11	969.04	1,133.05	805.63	0.35	0.42	0.21
Estonia	1,011.97	992.66	1,033.94	455.77	427.33	489.16	-0.43	-0.49	-0.36
Ethiopia	1,318.34	1,318.69	1,317.98	607.08	619.95	594.17	0.44	0.43	0.41
Federated States of Micronesia	1,470.72	1,293.83	1,640.05	1,145.71	999.98	1,284.52	0.05	0.03	0.06
Fiji	1,434.51	1,437.47	1,431.64	1,254.07	1,375.95	1,136.23	-0.24	-0.20	-0.29
Finland	780.48	739.07	824.41	432.55	394.53	471.74	-0.37	-0.41	-0.33
France	651.82	592.45	714.43	412.39	374.2	453.04	-0.30	-0.32	-0.28
Gabon	1,482.29	1,360.82	1,608.82	900.01	934.16	866.64	-0.09	-0.01	-0.18
Georgia	2,470.84	2,432.95	2,512.63	2,371.34	1,965.88	2,814.83	-0.03	-0.13	0.08
Germany	828.2	848.64	806.23	462.75	447.99	478.01	-0.37	-0.41	-0.33
Ghana	955.2	1,311.34	610.31	892.23	1,027.14	755.94	0.57	0.36	0.96
Greece	1,276.73	1,328.51	1,223.62	938.63	907.97	970.82	-0.26	-0.31	-0.19
Greenland	1,071.42	1,102.35	1,044.64	797.65	697.62	884.37	0.04	-0.10	0.16
Grenada	1,184.01	1,271.20	1,094.52	995.09	966.22	1,023.77	-0.08	-0.15	0.01
Guam	720.84	620.78	808.24	933.4	816	1,047.51	-0.04	-0.03	-0.05
Guatemala	444.57	444.28	444.85	379.79	379.88	379.7	0.57	0.62	0.52
Guinea	997.63	1,105.53	887.32	979.66	995.3	964.1	0.84	0.72	0.98
Guinea-Bissau	1,232.35	1,267.10	1,196.76	1,353.95	1,432.02	1,274.92	0.61	0.64	0.58
Guyana	1,634.75	1,434.18	1,841.82	1,446.85	1,330.38	1,562.20	0.00	0.08	-0.05
Haiti	1,828.90	2,374.25	1,267.50	1,294.32	1,613.66	967.99	0.36	0.28	0.49
Honduras	2,612.53	2,532.52	2,691.97	793.7	674.95	912.45	-0.52	-0.60	-0.45
Hungary	1,547.86	1,425.70	1,680.00	649.5	582.58	723.28	-0.46	-0.51	-0.41
Iceland	452.52	462.73	442.4	254.69	214.48	295.77	-0.35	-0.45	-0.24
India	1,001.67	1,017.64	986.8	875.56	759.45	983.46	0.50	0.40	0.58
Indonesia	1,460.66	1,476.80	1,444.65	1,854.97	1,734.81	1,973.39	0.96	0.88	1.04
Iran	763.39	670.92	852.5	565.66	466.93	662.91	0.41	0.41	0.41
Iraq	903.17	866.16	939.51	701.62	649.51	752.43	-0.04	0.01	-0.07
Ireland	578.39	618.65	537.77	304.85	285.6	324.68	-0.35	-0.43	-0.26
Israel	491.55	498.92	484.16	268.96	237.5	300.95	-0.34	-0.43	-0.24
Italy	809.88	770.91	851.24	616.79	591.11	644.1	-0.17	-0.21	-0.13
Jamaica	960.8	1,022.43	897.58	977.04	958.98	995.25	-0.03	-0.07	0.02
Japan	830.01	712.52	951.73	727.86	574.66	889.2	-0.22	-0.27	-0.18
Jordan	353.76	402.35	309.79	180	164.85	194.42	-0.23	-0.36	-0.08
Kazakhstan	1,120.15	1,168.05	1,069.25	1,146.33	1,068.97	1,229.23	0.11	0.05	0.20
Kenya	493.59	550.69	436.1	474.83	466.67	482.99	0.33	0.20	0.51
Kiribati	2,368.73	1,665.26	3,086.87	2,387.94	1,383.45	3,418.30	0.43	0.28	0.46
Kuwait	170.47	172.58	168.62	165.49	118.69	202.02	0.20	-0.20	0.55
Kyrgyzstan	1,395.05	1,465.95	1,321.10	1,249.32	1,083.50	1,418.66	0.26	0.10	0.46
Laos	2,121.37	2,193.57	2,049.08	1,207.94	1,122.14	1,294.59	0.58	0.48	0.69
Latvia	1,498.39	1,582.92	1,401.03	847.83	799.8	903.78	-0.34	-0.43	-0.23
Lebanon	716.42	997.49	427.6	357.67	519.53	197.45	-0.13	-0.20	-0.11
Lesotho	725.36	717.28	733.92	1,130.60	1,126.17	1,135.13	-0.03	-0.07	-0.01
Liberia	981.47	998.79	964.05	705.62	755.22	656.82	0.86	0.84	0.85
Libya	955.39	964.77	946.99	611.93	581.07	642.43	-0.32	-0.30	-0.34
Lithuania	745.46	753.87	736.1	657.28	605.67	717.3	-0.11	-0.18	-0.04
Luxembourg	1,072.64	1,094.59	1,049.80	380.15	367.29	393.07	-0.49	-0.55	-0.43
Macedonia	1,640.98	1,649.14	1,632.91	1,630.57	1,575.32	1,686.31	0.07	0.03	0.11
Madagascar	1,578.32	1,525.38	1,631.44	1,329.36	1,236.96	1,422.34	0.57	0.52	0.56
Malawi	635.59	765.19	501.44	421.41	413.49	429.36	0.43	0.19	0.79
Malaysia	911.59	855.52	965.99	791.01	610.42	974.96	-0.08	-0.19	0.01
Maldives	653.48	531.42	769.39	329.96	206.39	452.84	0.36	0.09	0.51
Mali	1,141.45	1,365.97	912.05	768.94	926.69	614.3	0.19	0.18	0.20
Malta	622.56	603.68	642.12	425.67	388.46	463.18	-0.31	-0.35	-0.28
Marshall Islands	1,398.68	1,128.51	1,657.05	1,240.99	1,013.91	1,460.27	0.07	0.06	0.07
Mauritania	792.12	836.21	747.77	562.15	635.96	489.35	0.30	0.34	0.22
Mauritius	1,459.93	1,112.00	1,809.28	1,069.50	820.99	1,324.13	-0.28	-0.29	-0.27
Mexico	359.68	368.61	350.66	345.82	332.78	358.99	0.27	0.16	0.38
Moldova	1,501.15	1,539.25	1,459.36	1,186.65	1,032.99	1,352.77	-0.04	-0.16	0.10
Mongolia	1,556.12	1,539.69	1,572.94	2,862.41	2,469.67	3,263.13	1.69	1.74	1.71

Montenegro	2,692.35	2,939.84	2,442.70	2,810.90	2,913.47	2,705.88	0.01	-0.01	0.04
Morocco	715.55	747.11	683.87	555.12	579.02	530.66	0.17	0.16	0.16
Mozambique	843.98	756.44	939.42	890.64	751.4	1,036.29	0.92	0.76	1.00
Myanmar	1,598.24	1,360.70	1,845.44	1,537.33	1,161.54	1,930.33	0.68	0.61	0.72
Namibia	709.53	738.52	679.29	502.94	456.69	551.67	-0.17	-0.27	-0.09
Nepal	722.23	694.23	750.64	652.78	558.73	752.8	1.07	0.97	1.13
Netherlands	613.03	618.99	606.93	416.83	408.01	425.79	-0.26	-0.31	-0.21
New Zealand	652.82	715.5	588.21	388.98	427.43	349.07	-0.29	-0.32	-0.25
Nicaragua	357.58	372.08	342.87	347.82	337.86	358.06	0.73	0.64	0.84
Niger	872.89	831.86	915.3	689.47	679.13	699.64	0.84	0.90	0.78
Nigeria	754.6	757.18	752.08	431.12	375.58	484.6	0.04	-0.08	0.15
North Korea	1,703.06	1,792.33	1,608.51	2,444.03	2,302.54	2,591.89	0.53	0.42	0.66
Northern Mariana Islands	891.37	810.82	963.77	511.24	339.25	684.45	-0.28	-0.43	-0.17
Norway	826.15	824.69	827.65	371.19	364.7	377.61	-0.41	-0.46	-0.37
Oman	517.58	672.78	393.77	317.64	329.58	311.58	0.05	-0.11	0.32
Pakistan	864.27	766.09	955.38	968.55	799.21	1,128.87	0.69	0.65	0.70
Palestine	714.61	728.79	700.78	509.71	421.2	595.75	0.08	-0.08	0.24
Panama	488.34	460.12	515.94	454.01	382.79	524.82	-0.01	-0.11	0.07
Papua New Guinea	2,229.01	2,459.50	2,009.02	1,890.47	2,186.19	1,606.33	0.16	0.19	0.11
Paraguay	800	856.22	745.06	917.78	864.49	969.47	0.23	0.14	0.33
Peru	962.95	828.22	1,097.72	306.51	285.79	327.27	-0.34	-0.31	-0.37
Philippines	842.25	616.84	1,062.98	1,297.65	1,005.93	1,583.99	1.13	1.26	1.05
Poland	954.95	888.77	1,024.55	704.42	588.85	828.8	-0.15	-0.25	-0.06
Portugal	1,742.73	1,601.53	1,894.58	858.78	734.76	991.04	-0.41	-0.46	-0.35
Puerto Rico	397.59	342.91	455.74	464.53	432.13	499.52	0.26	0.23	0.27
Qatar	425.68	490.2	393.82	189.8	185.39	191.45	-0.38	-0.49	-0.32
Romania	1,809.62	1,802.29	1,817.16	1,987.06	1,850.50	2,132.41	0.15	0.09	0.21
Russia	1,222.99	1,277.00	1,161.66	1,325.47	1,146.96	1,535.10	0.01	-0.13	0.18
Rwanda	1,226.49	1,469.63	974.74	551.47	599.69	499.02	0.05	-0.05	0.17
Saint Lucia	1,046.19	1,073.79	1,017.77	932.76	936.72	928.65	0.05	0.03	0.08
Saint Vincent and the Grenadines	820.54	918.12	721.98	981.14	918.33	1,042.79	0.21	0.08	0.39
Samoa	1,086.48	849.44	1,304.97	801.16	773.16	827.45	-0.14	-0.07	-0.17
Sao Tome and Principe	1,085.32	1,047.27	1,124.07	950.53	985.74	915.06	0.56	0.64	0.45
Saudi Arabia	363.34	387.01	344.72	276.82	219.8	320.62	0.33	0.08	0.54
Senegal	778.71	749.58	808.11	804.6	832.68	775.5	1.01	1.12	0.84
Serbia	1,724.23	1,836.14	1,609.32	1,244.95	1,245.38	1,244.49	-0.29	-0.35	-0.24
Seychelles	982.87	757.44	1,209.28	670.09	473.77	861.2	-0.24	-0.33	-0.19
Sierra Leone	950.97	765.43	1,140.35	967.64	1,027.70	906.34	0.82	1.29	0.47
Singapore	661.79	640.34	682.69	346.77	312.13	382.64	-0.39	-0.46	-0.33
Slovakia	1,099.34	1,023.67	1,178.59	569.1	486.31	656.45	-0.38	-0.46	-0.30
Slovenia	865.22	792.94	941.86	577.85	525.32	631.31	-0.22	-0.26	-0.18
Solomon Islands	1,975.02	1,987.08	1,963.61	1,739.00	1,790.78	1,688.82	0.15	0.16	0.13
Somalia	1,507.39	1,742.87	1,267.70	1,079.04	1,262.85	893.53	0.06	0.07	0.05
South Africa	790.24	922.25	655.15	734.98	738.36	731.49	-0.12	-0.24	0.05
South Korea	1,320.36	1,277.25	1,363.27	601.24	509.72	693.87	-0.48	-0.55	-0.40
South Sudan	1,131.36	1,257.41	1,004.11	913.07	1,013.87	812.57	0.28	0.27	0.29
Spain	749.51	689.75	811.76	423.67	399.35	448.61	-0.33	-0.35	-0.30
Sri Lanka	498.2	378.98	615.13	578.34	447.95	718.51	0.63	0.58	0.68
Sudan	1,568.96	1,759.04	1,380.37	1,009.79	1,097.50	922.72	0.25	0.23	0.27
Suriname	983.22	974.17	991.99	1,170.47	1,052.66	1,287.83	0.36	0.29	0.43
Swaziland	692.2	730.38	649.5	943.84	975.5	911.52	-0.01	-0.07	0.04
Sweden	711.86	707.38	716.45	422.38	411.15	433.64	-0.29	-0.33	-0.24
Switzerland	612.58	600.32	625.41	282.06	283.68	280.41	-0.43	-0.45	-0.41
Syria	1,390.66	1,145.94	1,627.53	713.64	547.83	875.41	-0.57	-0.48	-0.62
Taiwan	982.79	820.48	1,133.46	613.64	456.02	771.79	-0.38	-0.47	-0.32
Tajikistan	896.09	965.01	826.34	679.36	685.24	673.62	0.48	0.43	0.57
Tanzania	691.04	746.01	635.09	515.92	524.55	507.18	0.41	0.32	0.48
Thailand	1,133.56	907.43	1,363.86	1,100.54	731.53	1,480.17	-0.06	-0.18	0.01
The Bahamas	753.81	710.83	797.39	786.74	726.52	849.55	0.15	0.08	0.21
The Gambia	668.08	694.71	641.83	534.27	487.21	582.26	0.49	0.38	0.60
Timor-Leste	1,229.45	1,316.97	1,144.13	831.69	911.35	754.49	0.76	0.83	0.69

Togo	874.55	896.59	851.94	842.59	726.97	960.93	0.45	0.29	0.60
Tonga	779.36	899.6	662.03	711.94	716.32	707.57	0.01	-0.08	0.12
Trinidad and Tobago	871.08	841.91	900.47	938.15	823.91	1,055.43	0.02	-0.02	0.06
Tunisia	827.51	688.62	964.71	603.75	454.49	756.51	-0.02	-0.07	0.01
Turkey	631.42	657.63	604.56	412.79	363.29	464.05	0.24	0.04	0.47
Turkmenistan	1,616.03	1,589.47	1,643.33	1,856.52	1,522.69	2,201.75	0.92	0.70	1.14
Uganda	939.13	1,116.99	758.94	642	597.47	686.57	0.39	0.10	0.77
Ukraine	1,355.43	1,366.91	1,342.07	1,243.58	1,069.93	1,448.68	-0.15	-0.25	-0.04
United Arab Emirates	696.39	769.71	658.31	636.95	637.75	636.66	0.15	0.18	0.17
United Kingdom	869.89	921.44	815.37	485.38	497.46	473	-0.31	-0.35	-0.26
United States	398.19	405.78	390.2	309.71	299.12	320.66	-0.16	-0.24	-0.08
Uruguay	819.53	786.63	854.45	680.68	660.79	701.98	-0.07	-0.10	-0.05
Uzbekistan	884.97	933.43	835.44	831.96	769.7	896.26	0.26	0.15	0.39
Vanuatu	1,930.85	1,847.27	2,009.89	1,844.12	1,874.17	1,814.81	0.09	0.13	0.05
Venezuela	474.82	487.56	462.29	554.87	488.22	622.1	0.13	0.07	0.21
Vietnam	1,286.67	1,012.87	1,568.92	1,407.32	973.84	1,850.12	0.58	0.47	0.62
Virgin Islands, U.S.	596.96	531.92	665.87	906.79	711.63	1,121.31	0.14	0.04	0.21
Yemen	1,562.62	1,803.10	1,317.73	1,049.23	1,201.64	899.94	0.41	0.42	0.42
Zambia	673.89	857.3	487.38	706.61	627.62	785.81	0.76	0.27	1.57
Zimbabwe	739.25	831.09	646.64	838.21	759.38	919.35	-0.01	-0.21	0.24
**Hypertensive heart disease**									
Afghanistan	323.81	491.91	163.78	281.72	430.56	141.96	0.43	0.50	0.37
Albania	65.7	64.78	66.57	61.28	62.81	59.73	0.05	0.12	-0.02
Algeria	158.86	209.86	109.1	190.65	248.2	133.89	0.72	0.67	0.77
American Samoa	228.31	152.79	299.8	126.09	104.76	147.31	-0.33	-0.28	-0.34
Andorra	52.83	52.25	53.43	49.32	45.27	53.55	-0.06	-0.11	-0.01
Angola	377.05	502.34	248.66	288.95	388.9	187.7	0.54	0.49	0.58
Antigua and Barbuda	433.52	490.92	372.12	288.64	305.09	270.69	-0.26	-0.32	-0.19
Argentina	193.42	173.7	213.92	184.85	171.68	198.59	0.13	0.13	0.14
Armenia	158.43	184.2	131.11	251.77	277.82	221.77	0.77	0.70	0.85
Australia	53.72	59.86	47.55	43.9	48.27	39.52	-0.05	-0.11	0.01
Austria	187.1	220.11	151.13	231.39	275.74	184.84	0.47	0.47	0.48
Azerbaijan	106.89	117.07	96.27	113.39	118.1	108.62	0.60	0.54	0.69
Bahrain	52.66	79.5	32.91	32.64	53.56	19.84	-0.10	-0.06	-0.11
Bangladesh	222.13	217.39	226.66	180.48	170.32	190.44	0.89	0.96	0.82
Barbados	263.03	296.95	226.47	265.97	288.53	241.45	-0.04	-0.11	0.04
Belarus	122.62	125.5	119.35	266.8	239.21	298.8	0.87	0.78	1.01
Belgium	61.78	72.57	50.49	55.9	71.26	39.93	0.03	0.10	-0.08
Belize	198.6	204.65	192.66	171.87	160.81	183	0.03	-0.01	0.08
Benin	127.67	168.21	84.54	131.09	164.65	97.4	0.95	0.84	1.16
Bermuda	184.83	157.37	212.63	107.32	85.62	129.42	-0.26	-0.35	-0.20
Bhutan	228.8	231.7	226.11	167.22	146.4	185.15	0.64	0.54	0.71
Bolivia	60.84	75.68	46.09	55.24	69.78	40.75	0.96	1.00	0.90
Bosnia and Herzegovina	88.68	101	76.34	103.88	117.38	90.26	0.19	0.14	0.24
Botswana	309.12	397.43	218.72	398.9	490.85	306.87	-0.01	-0.04	0.06
Brazil	171.35	173.97	168.7	204.04	204.85	203.2	0.50	0.48	0.52
Brunei	63	44.1	79.92	70.94	50.46	90.22	0.15	0.16	0.15
Bulgaria	496.09	462.91	530.22	1,088.31	1,022.54	1,157.34	1.14	1.11	1.16
Burkina Faso	193	265.85	116.75	182.42	253	110.92	0.75	0.72	0.77
Burundi	781.79	958.78	598.6	317.99	410.86	223.1	-0.09	-0.03	-0.18
Cambodia	270.53	277.06	263.53	216.8	215.45	218.21	0.95	0.95	0.97
Cameroon	150.78	197.74	103.22	140.01	188.55	91.49	0.23	0.26	0.15
Canada	36.16	39.18	33.08	40.02	40.57	39.45	0.16	0.03	0.30
Cape Verde	103.33	127.17	76.97	88.19	113.86	61.82	0.32	0.40	0.23
Central African Republic	561.04	699.32	418.07	563.27	655.82	468.26	0.22	0.09	0.35
Chad	122.25	174.35	69.4	113.21	153.9	72.62	0.34	0.26	0.50
Chile	135.64	136.73	134.51	182.26	189.37	174.97	0.54	0.49	0.58
China	391.08	373.54	407.72	285.75	236.91	331.75	0.08	0.03	0.13
Colombia	198.23	212.92	183.34	131.28	133.76	128.73	-0.12	-0.22	-0.01
Comoros	465.33	533.07	398.02	247.22	297.99	197.37	0.15	0.21	0.07
Congo	486.63	635.44	337.04	358.41	534.97	182.11	0.04	0.05	-0.14

Costa Rica	97.08	104.56	89.72	164.66	171.84	157.48	0.78	0.70	0.85
Cote d'Ivoire	156.33	188.71	126.5	135.96	171	102.11	0.23	0.29	0.10
Croatia	493.1	627.91	349.85	348.12	394.14	298.73	-0.24	-0.35	-0.05
Cuba	105.86	111.15	100.64	273.07	260.05	285.97	1.47	1.24	1.73
Cyprus	142.22	151.01	133.21	86.13	85.34	86.98	-0.29	-0.34	-0.25
Czech Republic	72.54	69.35	75.93	128.78	126.73	130.89	1.20	1.20	1.21
Democratic Republic of the Congo	320.83	450.21	188.25	310.13	393.89	225.93	0.57	0.41	0.95
Denmark	68.35	71.51	65.09	53.33	54.44	52.21	-0.04	-0.08	0.01
Djibouti	265.76	294.79	237.08	316.2	327.94	304.57	0.63	0.53	0.69
Dominica	550.31	627.79	472.27	493.32	469.88	517.14	-0.19	-0.26	-0.08
Dominican Republic	161.12	165.65	156.65	213.12	215.4	210.82	0.78	0.79	0.78
Ecuador	140.41	158.33	122.7	226.71	217.27	236.15	1.26	0.96	1.65
Egypt	217.88	272.8	163.33	211.11	249.47	173.53	0.68	0.65	0.77
El Salvador	121.03	134.86	106.54	140.74	162.08	116.62	0.73	0.78	0.61
Equatorial Guinea	646.69	845.88	456.1	273.33	333.36	216.23	-0.20	-0.26	-0.09
Eritrea	398.33	485.38	310.18	323.17	435.25	211.5	0.42	0.50	0.20
Estonia	155.37	151.95	159.26	1,019.08	1,063.88	966.49	7.37	7.35	7.19
Ethiopia	448.12	456.31	439.89	238.22	270.63	205.71	0.66	0.80	0.45
Federated States of Micronesia	328.22	294.93	360.08	279.36	251.85	305.55	0.14	0.14	0.15
Fiji	324.44	338.13	311.18	562.77	592.48	534.05	0.51	0.47	0.54
Finland	118.56	133.26	102.96	153.12	151.63	154.65	0.48	0.26	0.77
France	85.55	101.23	69.03	73.72	85.66	61.01	-0.04	-0.09	0.01
Gabon	536.51	661.19	406.64	314.23	424.05	206.88	-0.12	-0.07	-0.23
Georgia	174.37	195.84	150.69	746.67	679.05	820.63	3.32	2.73	4.23
Germany	255.42	299.98	207.53	247.03	301.89	190.3	0.09	0.13	0.03
Ghana	426.22	650.41	209.11	189.68	315.48	62.61	-0.25	-0.16	-0.52
Greece	107.07	134.2	79.26	173.98	195.87	151	0.64	0.47	0.94
Greenland	85.53	92.98	79.09	109.01	103.56	113.73	0.78	0.59	0.98
Grenada	356.22	415.31	295.57	289.68	331.02	248.6	-0.11	-0.11	-0.10
Guam	720.56	642.59	788.67	436.98	356.03	515.66	-0.55	-0.59	-0.52
Guatemala	97.18	122.14	71.87	78.17	96.37	59.19	0.48	0.50	0.46
Guinea	170.98	260.08	79.88	155.68	217.81	93.84	0.71	0.59	1.14
Guinea-Bissau	197.18	272.36	120.18	187.96	261.03	113.97	0.39	0.39	0.41
Guyana	624.48	632.34	616.36	520.94	526.02	515.9	-0.05	-0.03	-0.06
Haiti	534.41	684.7	379.7	425.25	504.38	344.39	0.53	0.39	0.77
Honduras	197.56	200.07	195.07	224.66	230.87	218.45	0.78	0.75	0.80
Hungary	517.35	569.94	460.47	475.65	501.16	447.54	0.18	0.05	0.32
Iceland	46.23	45.47	46.99	42.48	38.39	46.66	0.07	0.00	0.13
India	322.28	302.14	341.04	312.32	285.35	337.38	0.66	0.77	0.57
Indonesia	180.3	204.98	155.83	197.14	219.66	174.93	0.69	0.72	0.68
Iran	259.45	262.15	256.85	369.73	340.14	398.89	1.72	1.63	1.82
Iraq	210.7	265.75	156.65	170.31	208.24	133.32	0.00	0.05	-0.01
Ireland	47.57	51.24	43.87	36.84	34.96	38.77	-0.05	-0.16	0.08
Israel	91.85	100.76	82.91	26.36	31	21.65	-0.65	-0.63	-0.68
Italy	251.53	301.75	198.24	300.27	348.59	248.9	0.31	0.20	0.45
Jamaica	427.89	480.86	373.55	305.58	323.63	287.39	-0.32	-0.33	-0.29
Japan	135.31	151.18	118.86	89.74	105.91	72.7	-0.41	-0.37	-0.47
Jordan	237.18	288.87	190.4	202.32	198.27	206.18	0.29	0.07	0.58
Kazakhstan	183.94	192.38	174.97	318.16	297.6	340.18	0.88	0.77	1.03
Kenya	176.94	232.69	120.79	185.75	225.8	145.71	0.46	0.37	0.64
Kiribati	328.36	284.88	372.75	164.57	129.43	200.6	-0.29	-0.30	-0.29
Kuwait	137.61	140.28	135.27	95.77	90.55	99.84	-0.14	-0.25	-0.04
Kyrgyzstan	147.31	148.75	145.8	146.4	126.71	166.51	0.40	0.27	0.55
Laos	337.63	367.03	308.19	205.24	206.63	203.84	0.69	0.63	0.77
Latvia	47.93	48.68	47.06	307.03	306.78	307.32	6.44	6.11	6.77
Lebanon	170.08	212.93	126.06	88.23	126.6	50.24	-0.10	-0.08	-0.23
Lesotho	411.82	475.73	344.14	611.33	749.62	470.24	-0.07	-0.06	-0.13
Liberia	146.5	203.11	89.57	115.59	163.28	68.66	1.04	0.95	1.08
Libya	82.28	115	52.97	92.61	123.42	62.17	0.20	0.26	0.15
Lithuania	81.09	76.71	85.97	204.11	174.61	238.43	1.53	1.31	1.74
Luxembourg	93.82	122.04	64.44	57.83	69.74	45.88	-0.12	-0.23	0.08

Macedonia	283.08	315.25	251.28	302.03	328.35	275.48	0.15	0.12	0.18
Madagascar	559.61	623.89	495.12	510.74	586.58	434.43	0.70	0.77	0.57
Malawi	289.27	398.13	176.59	223.52	269.17	177.7	0.67	0.49	1.10
Malaysia	49.98	50.4	49.57	47.39	38.74	56.2	0.00	-0.13	0.13
Maldives	96.33	127.3	66.92	41.85	48.67	35.07	0.17	0.08	0.34
Mali	189.11	283.26	92.92	111.14	167.04	56.35	0.04	0.03	0.08
Malta	70.31	71.34	69.25	84.1	83.56	84.65	0.20	0.17	0.23
Marshall Islands	280.79	220.55	338.39	264.56	212.32	315.01	0.13	0.14	0.13
Mauritania	130.17	184.93	75.09	86.81	127.46	46.73	0.22	0.22	0.15
Mauritius	312.41	293.1	331.8	391.55	365.66	418.08	0.23	0.20	0.25
Mexico	92.65	113.24	71.84	102.75	121.83	83.48	0.46	0.38	0.56
Moldova	85.41	92.48	77.65	267.09	282.37	250.58	2.80	2.80	2.84
Mongolia	179.93	184.35	175.41	126.7	105.51	148.32	0.03	-0.02	0.10
Montenegro	36.75	44	29.44	47.81	55	40.45	0.26	0.25	0.29
Morocco	169.16	219.67	118.46	153.96	203	103.77	0.37	0.38	0.31
Mozambique	320.63	374.96	261.41	323.81	361.61	284.27	0.84	0.70	0.97
Myanmar	363.45	478.81	243.4	341.17	414.65	264.34	0.65	0.63	0.79
Namibia	355.77	424.96	283.56	242.57	270.35	213.32	-0.21	-0.25	-0.15
Nepal	233.64	216.66	250.88	208.32	174.61	244.17	1.04	0.98	1.07
Netherlands	45.76	52.81	38.55	45.51	52.26	38.65	0.08	0.04	0.12
New Zealand	69.55	74.42	64.53	45.27	51.26	39.06	-0.22	-0.22	-0.24
Nicaragua	72.44	81.51	63.24	126.03	132.86	119.01	2.10	1.95	2.31
Niger	114.35	161.36	65.76	98.69	136.15	61.84	1.01	0.96	1.18
Nigeria	138.9	197.03	81.94	77.99	103.19	53.72	0.02	-0.03	0.17
North Korea	248.82	304.56	189.78	354.63	401.67	305.48	0.51	0.46	0.65
Northern Mariana Islands	78.62	97.87	61.33	48.49	46.34	50.66	-0.23	-0.36	-0.03
Norway	56.16	61	51.21	34.02	41.09	27.04	-0.21	-0.18	-0.27
Oman	33.17	54.04	16.53	37.16	53.05	29.1	0.91	0.78	1.95
Pakistan	270.45	195.82	339.72	245.57	174.76	312.59	0.37	0.41	0.32
Palestine	110.08	117.6	102.74	142.41	128.97	155.48	0.96	0.75	1.21
Panama	73.18	75.38	71.02	87.71	87.49	87.93	0.27	0.24	0.30
Papua New Guinea	399.61	416.49	383.5	316.52	338.67	295.23	0.08	0.09	0.07
Paraguay	82.86	88.67	77.18	178.52	173.95	182.95	1.30	1.22	1.42
Peru	52.45	64.74	40.16	41.44	48.88	33.99	0.63	0.52	0.78
Philippines	199.26	157.02	240.63	357.98	301.9	413.02	1.48	1.66	1.37
Poland	182.86	193.96	171.19	150.47	137.3	164.66	-0.05	-0.20	0.12
Portugal	98.65	118.26	77.55	98.75	114.92	81.51	0.20	0.13	0.30
Puerto Rico	198.1	180.05	217.29	308.07	275.56	343.18	0.67	0.50	0.83
Qatar	35.2	53.26	26.29	13.53	22.81	10.06	-0.47	-0.42	-0.47
Romania	458.2	516.09	398.66	736.15	745.51	726.18	0.68	0.54	0.88
Russia	91.63	97.23	85.26	237.5	225.25	251.89	1.41	1.25	1.63
Rwanda	514.78	687.05	336.4	277.24	350.19	197.89	0.26	0.19	0.34
Saint Lucia	398.94	458.96	337.12	314.36	346	281.52	-0.07	-0.11	-0.01
Saint Vincent and the Grenadines	434.79	513.64	355.16	483.24	488.45	478.13	0.12	0.03	0.29
Samoa	270.68	233.95	304.53	219.28	222.34	216.4	-0.05	-0.03	-0.07
Sao Tome and Principe	73.12	101.49	44.22	68.49	96.11	40.65	0.67	0.65	0.64
Saudi Arabia	23.29	37.14	12.39	25.93	36.24	18.01	0.95	0.85	1.41
Senegal	130.43	181.39	78.99	129.11	185.3	70.87	0.92	0.94	0.72
Serbia	178.48	198.15	158.28	251.6	281.94	219.77	0.38	0.37	0.37
Seychelles	1,411.24	1,547.78	1,274.12	1,095.12	1,247.63	946.64	-0.14	-0.14	-0.15
Sierra Leone	124.96	149.03	100.39	147.44	206.74	86.92	1.11	1.37	0.60
Singapore	164.77	162.8	166.69	223.59	176.02	272.86	0.57	0.21	0.94
Slovakia	118.36	120.94	115.66	217.57	210.62	224.91	1.20	0.96	1.44
Slovenia	260.37	303.26	214.88	261.72	300.19	222.56	0.18	0.11	0.26
Solomon Islands	406.48	373.64	437.58	348.2	314.74	380.62	0.12	0.08	0.14
Somalia	473.7	603.22	341.86	340.84	441.98	238.77	0.07	0.09	0.03
South Africa	394.45	524.25	261.63	413.89	498.44	326.35	0.00	-0.09	0.18
South Korea	151.24	168.79	133.78	90.06	114.06	65.76	-0.31	-0.24	-0.42
South Sudan	422.1	527.25	315.96	346.03	436.17	256.15	0.30	0.31	0.29
Spain	62.6	80.17	44.28	86.11	108.2	63.44	0.64	0.51	0.81
Sri Lanka	114.18	83.6	144.17	181.11	157.7	206.29	1.23	1.52	1.05

Sudan	226.11	318.45	134.51	152.86	217.51	88.69	0.31	0.35	0.26
Suriname	290.42	335	247.27	254.15	276.85	231.54	0.00	-0.01	0.03
Swaziland	368.33	441.06	286.98	457.68	563.05	350.1	-0.10	-0.12	-0.10
Sweden	45.8	51.08	40.4	73.06	85.11	60.98	0.92	0.91	0.89
Switzerland	102.01	121.71	81.39	138.24	167.15	108.68	0.68	0.61	0.75
Syria	57.84	58.35	57.34	45.76	50.21	41.42	-0.34	-0.07	-0.50
Taiwan	150.66	112.28	186.29	179.87	146.43	213.42	0.18	0.25	0.15
Tajikistan	148.14	172.25	123.74	126.75	132.78	120.86	0.67	0.55	0.87
Tanzania	279.85	341.09	217.53	229.47	272.73	185.68	0.55	0.50	0.58
Thailand	106.81	91.81	122.1	82.51	81.13	83.94	-0.25	-0.10	-0.36
The Bahamas	598.81	608.96	588.52	800.15	822.82	776.5	0.47	0.43	0.49
The Gambia	111.53	156.65	67.06	84.12	109.95	57.79	0.41	0.39	0.53
Timor-Leste	175.19	207.3	143.89	138.69	175.36	103.16	1.06	1.24	0.84
Togo	151.01	212.37	88.05	127.34	155.08	98.94	0.27	0.16	0.59
Tonga	110.9	111.78	110.04	113.79	104.07	123.45	0.13	0.08	0.18
Trinidad and Tobago	426.72	447.84	405.44	374.12	342.25	406.83	-0.17	-0.23	-0.09
Tunisia	102.71	123.14	82.53	73.46	86.94	59.65	-0.04	-0.01	-0.07
Turkey	179.41	188.92	169.66	137.58	149.12	125.63	0.46	0.48	0.42
Turkmenistan	174.45	188.71	159.78	211.51	206.01	217.19	1.03	0.94	1.17
Uganda	402.67	524.59	279.14	269.07	299.87	238.23	0.35	0.18	0.67
Ukraine	139.68	143.47	135.26	256.98	248.6	266.87	0.70	0.67	0.76
United Arab Emirates	100.64	128.92	85.96	77.5	91.88	72.26	-0.04	0.02	0.02
United Kingdom	82.07	80.35	83.88	73.23	66.94	79.69	0.10	0.00	0.21
United States	183.45	175.08	192.26	216.2	180.49	253.12	0.27	0.06	0.48
Uruguay	154.46	148.77	160.49	184.08	192.72	174.82	0.34	0.39	0.26
Uzbekistan	677.84	626.7	730.12	748.1	658.49	840.67	0.48	0.47	0.49
Vanuatu	427.89	402.02	452.35	372.4	371.62	373.17	-0.01	0.03	-0.04
Venezuela	234.43	241.18	227.8	223.99	210.2	237.9	-0.07	-0.07	-0.06
Vietnam	186.8	200.59	172.59	190.51	194.18	186.77	0.48	0.48	0.49
Virgin Islands, U.S.	329.17	291.64	368.91	620.41	527.72	722.27	0.41	0.41	0.41
Yemen	195.1	267.69	121.18	142.57	199.72	86.6	0.54	0.60	0.49
Zambia	258.32	368.29	146.5	279.67	305.34	253.92	0.81	0.44	1.76
Zimbabwe	94.38	114.8	73.8	99.35	99.97	98.7	-0.08	-0.25	0.16
**Ischemic heart disease**									
Afghanistan	4,618.47	4,859.57	4,388.95	4,620.05	4,688.19	4,556.08	0.65	0.65	0.64
Albania	1,804.86	1,208.86	2,372.50	3,289.09	2,217.35	4,378.09	1.05	1.12	1.01
Algeria	1,439.53	1,295.79	1,579.74	1,670.22	1,490.80	1,847.17	0.66	0.63	0.69
American Samoa	1,622.88	721.4	2,476.20	1,627.90	1,177.04	2,076.43	0.22	0.72	0.13
Andorra	1,797.69	1,095.68	2,537.90	1,559.27	858.52	2,289.43	-0.13	-0.20	-0.11
Angola	1,378.51	1,192.37	1,569.24	1,026.38	1,035.44	1,017.20	0.50	0.67	0.35
Antigua and Barbuda	1,992.37	1,708.39	2,296.12	1,557.22	1,278.89	1,860.76	-0.13	-0.18	-0.09
Argentina	2,772.24	1,927.09	3,650.69	2,063.88	1,575.73	2,573.29	-0.12	-0.07	-0.13
Armenia	3,922.61	3,048.91	4,848.63	5,038.82	3,654.92	6,632.81	0.43	0.35	0.50
Australia	3,114.46	2,272.87	3,960.62	1,509.23	1,111.66	1,907.83	-0.44	-0.46	-0.42
Austria	3,944.62	3,201.41	4,754.41	2,442.58	1,971.01	2,937.55	-0.26	-0.28	-0.25
Azerbaijan	4,533.24	3,667.70	5,436.40	4,530.12	3,299.54	5,775.17	0.50	0.37	0.59
Bahrain	2,590.58	1,925.61	3,079.62	1,125.57	817.86	1,313.99	-0.37	-0.41	-0.37
Bangladesh	757.91	742.36	772.77	2,033.94	1,476.49	2,580.66	5.24	3.99	6.23
Barbados	2,353.65	1,962.59	2,775.05	2,083.20	1,787.96	2,404.08	-0.16	-0.16	-0.16
Belarus	6,761.24	5,876.41	7,763.42	10,081.15	7,935.42	12,570.26	0.28	0.27	0.30
Belgium	3,548.65	2,736.65	4,398.27	1,973.78	1,469.58	2,497.86	-0.37	-0.40	-0.34
Belize	1,467.34	1,217.97	1,712.52	1,242.68	905.74	1,581.63	0.01	-0.06	0.05
Benin	935.2	853.57	1,022.06	1,193.06	972.39	1,414.60	1.42	1.14	1.59
Bermuda	3,769.80	2,291.94	5,266.34	1,831.06	1,120.02	2,555.72	-0.38	-0.41	-0.36
Bhutan	1,663.56	1,606.65	1,716.44	1,737.13	1,322.17	2,094.38	1.34	1.01	1.54
Bolivia	1,407.74	1,391.86	1,423.51	1,278.24	1,259.11	1,297.30	0.96	0.96	0.96
Bosnia and Herzegovina	3,243.89	2,465.13	4,023.50	2,793.71	2,094.99	3,498.89	-0.12	-0.17	-0.09
Botswana	1,016.06	915.14	1,119.38	1,555.26	1,240.87	1,869.94	0.17	0.06	0.26
Brazil	1,927.27	1,471.31	2,390.42	1,871.07	1,419.13	2,338.12	0.22	0.21	0.23
Brunei	1,119.28	711.35	1,484.39	1,287.54	794.38	1,751.59	0.17	0.13	0.21
Bulgaria	6,553.84	5,062.74	8,086.92	6,819.12	5,335.33	8,376.35	0.02	0.01	0.03

Burkina Faso	892.88	821.41	967.68	946.16	910.22	982.56	0.97	1.00	0.90
Burundi	1,471.34	1,245.29	1,705.32	727.41	619.59	837.57	0.11	0.13	0.08
Cambodia	2,257.21	1,988.87	2,544.71	2,016.33	1,580.80	2,473.70	1.18	1.00	1.31
Cameroon	907.33	894.96	919.85	1,006.48	826.51	1,186.34	0.47	0.22	0.68
Canada	2,921.01	2,008.03	3,849.69	1,833.35	1,302.74	2,371.42	-0.34	-0.35	-0.33
Cape Verde	839.77	607.06	1,097.12	1,028.32	931.68	1,127.54	0.89	1.40	0.58
Central African Republic	1,686.45	1,336.91	2,047.84	1,754.00	1,268.21	2,252.73	0.26	0.11	0.32
Chad	690.94	643.97	738.59	801.92	652.34	951.14	0.69	0.45	0.84
Chile	1,611.99	1,260.15	1,973.38	1,214.61	876.02	1,562.25	-0.13	-0.25	-0.04
China	1,190.69	1,027.08	1,345.89	1,862.36	1,356.14	2,339.20	1.32	1.15	1.41
Colombia	1,390.38	1,145.61	1,638.32	1,324.63	1,049.10	1,608.67	0.27	0.13	0.39
Comoros	1,095.00	778.21	1,409.75	679.86	555.55	801.93	0.34	0.55	0.23
Congo	2,032.51	1,893.08	2,172.66	1,386.03	1,646.00	1,126.46	-0.03	0.09	-0.18
Costa Rica	1,275.11	999.12	1,546.68	1,264.10	934.39	1,593.36	0.04	-0.03	0.09
Cote d'Ivoire	1,231.84	933.13	1,507.07	1,241.58	928.24	1,544.25	0.42	0.41	0.40
Croatia	4,604.96	3,577.26	5,697.04	4,322.89	3,750.11	4,937.60	0.01	0.08	-0.03
Cuba	3,206.92	2,570.70	3,833.59	3,022.53	2,406.46	3,632.76	-0.10	-0.11	-0.09
Cyprus	3,198.35	1,964.70	4,463.45	1,838.65	873.62	2,870.27	-0.33	-0.48	-0.26
Czech Republic	6,741.05	5,193.57	8,381.40	4,124.57	3,367.29	4,906.22	-0.24	-0.22	-0.25
Democratic Republic of the Congo	797.98	768.67	828	777.6	655.38	900.45	0.59	0.38	0.77
Denmark	4,849.09	3,631.53	6,102.17	1,831.10	1,333.28	2,336.86	-0.53	-0.56	-0.52
Djibouti	939.81	521.34	1,353.34	1,323.38	814.59	1,827.64	0.92	1.16	0.79
Dominica	1,736.84	1,660.61	1,813.63	1,784.51	1,448.67	2,125.68	-0.07	-0.14	-0.02
Dominican Republic	1,356.04	1,092.74	1,615.83	2,082.06	1,741.98	2,424.38	1.07	1.19	0.99
Ecuador	1,079.91	878.21	1,279.39	1,140.13	846.86	1,433.46	0.48	0.38	0.54
Egypt	2,945.12	2,541.22	3,346.24	2,911.82	2,203.88	3,605.12	0.71	0.57	0.80
El Salvador	1,545.16	1,346.41	1,753.27	1,676.39	1,447.44	1,935.19	0.61	0.59	0.63
Equatorial Guinea	2,146.47	1,902.84	2,379.59	1,108.28	1,024.30	1,188.16	-0.02	0.01	-0.04
Eritrea	812.12	584.33	1,042.79	899.91	801.96	997.51	0.94	1.31	0.68
Estonia	7,187.94	5,977.62	8,565.24	4,651.63	4,133.50	5,260.00	-0.17	-0.18	-0.17
Ethiopia	1,801.57	1,521.96	2,082.96	1,072.59	891.38	1,254.41	0.86	0.79	0.88
Federated States of Micronesia	2,466.42	1,651.33	3,246.67	2,955.19	1,980.44	3,883.58	0.61	0.60	0.61
Fiji	3,321.30	1,391.17	5,191.57	4,476.43	2,419.23	6,465.53	0.17	0.46	0.12
Finland	5,137.81	3,666.57	6,698.69	2,841.95	1,969.71	3,740.97	-0.37	-0.40	-0.34
France	1,980.95	1,411.98	2,580.91	1,424.43	988.02	1,889.08	-0.20	-0.25	-0.17
Gabon	2,046.99	1,691.11	2,417.71	1,203.82	1,152.11	1,254.38	-0.12	-0.01	-0.21
Georgia	6,762.58	5,616.82	8,026.28	6,278.40	4,394.77	8,338.74	-0.06	-0.16	0.00
Germany	4,941.08	4,069.38	5,877.62	2,866.08	2,226.93	3,526.92	-0.35	-0.38	-0.32
Ghana	1,075.86	1,329.23	830.5	1,254.45	1,261.62	1,247.22	0.96	0.65	1.39
Greece	3,479.25	2,475.50	4,508.80	3,432.29	2,616.33	4,288.66	0.00	0.06	-0.03
Greenland	2,017.65	1,181.86	2,740.98	1,729.95	896.78	2,452.34	0.20	0.08	0.23
Grenada	2,622.80	2,338.19	2,914.87	1,992.38	1,684.71	2,298.16	-0.17	-0.19	-0.15
Guam	2,054.30	1,258.61	2,749.33	4,671.27	3,314.64	5,989.86	0.69	0.94	0.60
Guatemala	1,052.86	958.64	1,148.44	922.33	839.41	1,008.88	0.61	0.66	0.56
Guinea	997.12	1,096.51	895.51	1,171.44	1,041.02	1,301.24	1.20	0.81	1.65
Guinea-Bissau	1,331.68	1,324.46	1,339.08	1,507.69	1,446.88	1,569.26	0.66	0.58	0.74
Guyana	2,435.14	1,768.97	3,122.88	3,127.40	2,350.32	3,897.00	0.46	0.54	0.40
Haiti	2,986.78	3,052.02	2,919.63	2,193.59	2,149.48	2,238.67	0.41	0.33	0.50
Honduras	1,796.04	1,650.83	1,940.23	2,048.50	1,895.35	2,201.65	0.79	0.74	0.83
Hungary	6,655.48	4,992.89	8,453.97	4,691.24	4,022.93	5,427.96	-0.10	-0.04	-0.13
Iceland	2,839.83	1,933.13	3,738.57	1,596.59	867.8	2,341.00	-0.35	-0.47	-0.28
India	2,302.12	1,724.74	2,839.86	2,803.30	1,865.57	3,674.72	1.09	1.03	1.05
Indonesia	1,607.04	1,407.08	1,805.30	2,166.96	1,590.84	2,734.77	1.08	0.81	1.26
Iran	2,271.86	1,719.49	2,804.16	2,653.37	1,749.96	3,543.27	1.23	1.07	1.29
Iraq	3,107.46	2,819.41	3,390.26	2,804.98	2,280.92	3,316.09	0.12	0.09	0.14
Ireland	3,488.57	2,470.34	4,515.83	1,623.01	968.12	2,297.30	-0.43	-0.52	-0.38
Israel	2,740.02	2,171.44	3,310.67	1,031.98	734.68	1,334.31	-0.55	-0.60	-0.51
Italy	2,765.54	1,981.50	3,597.43	2,046.41	1,583.46	2,538.63	-0.19	-0.17	-0.18
Jamaica	1,252.84	1,133.78	1,374.96	1,312.36	1,152.39	1,473.59	0.00	0.00	-0.02
Japan	1,453.03	1,124.69	1,793.20	1,486.90	1,137.04	1,855.36	-0.09	-0.09	-0.10
Jordan	1,759.12	1,387.96	2,094.98	1,094.78	608.61	1,557.45	-0.06	-0.32	0.09

Kazakhstan	3,958.58	3,276.48	4,683.44	4,644.90	3,398.35	5,980.55	0.28	0.18	0.34
Kenya	350.33	253.43	447.9	429	288.78	569.19	0.70	0.61	0.73
Kiribati	2,438.58	1,717.62	3,174.57	2,682.89	1,544.29	3,850.83	0.56	0.38	0.60
Kuwait	1,319.01	689.92	1,869.27	1,292.32	484.33	1,922.91	0.21	-0.18	0.33
Kyrgyzstan	3,093.77	2,709.23	3,494.83	3,448.45	2,622.06	4,292.42	0.57	0.44	0.67
Laos	2,942.81	2,858.82	3,026.91	1,990.68	1,618.65	2,366.41	0.88	0.64	1.09
Latvia	7,494.18	6,132.11	9,063.06	6,107.30	5,024.88	7,367.92	-0.05	-0.08	-0.03
Lebanon	4,075.06	2,607.15	5,583.37	2,462.62	1,811.35	3,107.32	0.05	0.07	0.07
Lesotho	1,116.29	859.21	1,388.51	2,042.31	1,749.75	2,340.82	0.14	0.22	0.08
Liberia	850.34	889.11	811.35	877.62	927.32	828.73	1.67	1.53	1.77
Libya	1,491.81	1,296.56	1,666.67	1,944.56	1,579.36	2,305.43	0.38	0.42	0.35
Lithuania	6,527.25	5,339.43	7,849.23	6,050.53	4,799.22	7,505.78	-0.07	-0.09	-0.05
Luxembourg	3,493.91	2,556.05	4,470.09	1,375.63	1,018.26	1,734.44	-0.44	-0.47	-0.41
Macedonia	2,988.79	2,061.66	3,905.05	3,277.41	2,339.80	4,223.28	0.18	0.23	0.17
Madagascar	1,212.26	765.67	1,660.29	1,176.48	742.17	1,613.49	0.81	0.83	0.74
Malawi	880.86	953.46	805.72	718.59	604.97	832.63	0.76	0.40	1.16
Malaysia	1,870.57	1,363.06	2,363.08	2,266.45	1,313.72	3,236.88	0.28	0.10	0.36
Maldives	1,624.22	1,287.54	1,943.95	1,040.76	600.21	1,478.87	0.72	0.31	0.95
Mali	1,080.00	1,302.28	852.9	728.03	868.01	590.82	0.19	0.16	0.23
Malta	3,176.37	2,263.79	4,121.44	2,450.66	1,739.67	3,167.40	-0.23	-0.23	-0.23
Marshall Islands	2,099.93	1,214.19	2,946.99	2,974.61	1,842.30	4,068.07	0.70	0.79	0.68
Mauritania	1,122.15	1,082.52	1,162.02	764.38	775.03	753.88	0.24	0.26	0.20
Mauritius	3,829.20	2,618.67	5,044.68	2,879.69	1,994.23	3,787.00	-0.26	-0.27	-0.25
Mexico	922.05	738.87	1,107.14	1,292.73	1,020.30	1,567.93	0.85	0.77	0.91
Moldova	5,433.61	5,173.01	5,719.53	5,342.00	4,607.94	6,135.61	0.19	0.11	0.28
Mongolia	2,770.63	2,530.37	3,016.59	3,343.76	2,313.00	4,395.43	0.77	0.56	0.90
Montenegro	2,810.56	1,976.64	3,651.76	3,702.02	2,431.34	5,002.95	0.27	0.23	0.29
Morocco	1,475.21	1,379.56	1,571.23	1,663.65	1,518.03	1,812.66	0.69	0.64	0.73
Mozambique	732.33	622.93	851.6	930.72	734.06	1,136.43	1.31	1.09	1.42
Myanmar	1,103.84	973.19	1,239.81	1,132.06	827.68	1,450.38	0.80	0.60	0.93
Namibia	1,390.06	1,132.86	1,658.44	1,077.26	845.76	1,321.13	-0.10	-0.12	-0.10
Nepal	1,747.51	1,714.30	1,781.22	1,918.55	1,571.21	2,287.91	1.51	1.25	1.73
Netherlands	3,207.52	2,127.82	4,311.72	1,597.83	1,075.75	2,128.63	-0.46	-0.47	-0.45
New Zealand	3,479.05	2,392.63	4,598.79	1,757.74	1,240.43	2,294.84	-0.39	-0.41	-0.37
Nicaragua	672.96	579.7	767.65	1,008.82	870.92	1,150.65	1.67	1.72	1.64
Niger	707.12	631.13	785.67	750.15	601.71	896.17	1.47	1.21	1.64
Nigeria	733.92	750.2	717.96	502.41	400.62	600.42	0.24	-0.01	0.50
North Korea	1,246.69	1,119.30	1,381.60	2,195.29	1,849.79	2,556.36	0.87	0.83	0.90
Northern Mariana Islands	975.41	660.58	1,258.37	888.16	450.36	1,329.07	0.14	-0.08	0.24
Norway	4,513.24	2,958.65	6,102.93	1,618.16	1,180.99	2,050.25	-0.53	-0.51	-0.53
Oman	1,295.03	1,190.80	1,378.17	1,165.74	866.88	1,317.27	0.53	0.32	0.60
Pakistan	2,652.53	2,259.89	3,016.91	3,377.49	2,713.36	4,006.20	0.92	0.90	0.91
Palestine	1,733.69	1,249.91	2,205.53	1,914.94	1,083.59	2,723.07	0.68	0.38	0.80
Panama	1,219.68	942.96	1,490.31	1,162.42	873.78	1,449.46	0.01	-0.01	0.02
Papua New Guinea	2,264.66	1,616.65	2,883.15	2,960.40	2,318.62	3,577.05	0.79	0.93	0.73
Paraguay	1,213.14	1,064.12	1,358.76	1,556.23	1,161.68	1,938.91	0.37	0.23	0.45
Peru	1,130.86	968.38	1,293.39	784.58	642.99	926.38	0.43	0.34	0.51
Philippines	1,596.99	1,112.02	2,071.87	2,256.75	1,559.46	2,941.18	0.95	0.94	0.96
Poland	6,169.59	4,429.58	7,999.60	3,742.68	2,742.59	4,818.99	-0.30	-0.30	-0.30
Portugal	2,717.42	2,100.95	3,380.42	1,576.77	1,231.13	1,945.35	-0.30	-0.32	-0.29
Puerto Rico	2,828.92	2,124.45	3,577.92	2,099.50	1,727.97	2,500.86	-0.20	-0.21	-0.19
Qatar	1,275.81	986.34	1,418.72	639.71	366.71	741.79	-0.31	-0.49	-0.27
Romania	5,118.96	4,125.62	6,140.87	5,249.78	4,133.79	6,437.62	0.08	0.07	0.08
Russia	6,063.18	5,147.21	7,103.34	7,244.83	5,584.66	9,194.47	0.11	0.05	0.15
Rwanda	1,004.09	824.52	1,190.02	670.76	542.96	809.77	0.56	0.54	0.55
Saint Lucia	1,874.41	1,536.74	2,222.18	1,544.06	1,302.82	1,794.54	-0.03	0.00	-0.04
Saint Vincent and the Grenadines	2,071.94	2,056.07	2,087.97	2,453.90	2,140.98	2,761.06	0.20	0.12	0.27
Samoa	1,912.57	1,235.18	2,536.97	2,067.58	1,829.40	2,291.23	0.26	0.51	0.18
Sao Tome and Principe	996.33	1,041.61	950.22	927.41	1,061.69	792.11	0.66	0.78	0.48
Saudi Arabia	1,164.25	1,011.64	1,284.28	1,233.25	746.8	1,606.95	0.85	0.40	1.07
Senegal	805.58	664.61	947.89	904.96	802.24	1,011.45	1.18	1.31	1.04

Serbia	3,838.05	3,055.70	4,641.42	4,162.91	3,405.35	4,957.46	0.06	0.07	0.05
Seychelles	2,587.18	1,730.71	3,447.35	2,026.47	1,388.76	2,647.29	-0.13	-0.14	-0.12
Sierra Leone	976.7	666.71	1,293.10	1,097.57	1,053.27	1,142.78	1.01	1.70	0.64
Singapore	2,203.74	1,573.02	2,818.03	1,541.73	1,036.64	2,064.84	-0.19	-0.27	-0.13
Slovakia	6,334.81	4,821.72	7,919.39	4,039.46	3,332.76	4,785.16	-0.24	-0.22	-0.24
Slovenia	2,939.09	2,252.24	3,667.40	2,147.15	1,718.39	2,583.63	-0.14	-0.14	-0.14
Solomon Islands	2,722.73	1,908.26	3,493.92	3,238.88	2,310.80	4,138.27	0.55	0.56	0.56
Somalia	1,151.45	899.33	1,408.07	991.9	830.72	1,154.58	0.28	0.37	0.22
South Africa	1,398.24	1,531.65	1,261.73	1,542.31	1,410.85	1,678.42	0.05	-0.12	0.26
South Korea	1,714.90	1,407.51	2,020.93	850.33	680.79	1,021.92	-0.43	-0.45	-0.41
South Sudan	917.41	771.34	1,064.86	990.93	923.44	1,058.22	0.72	0.90	0.58
Spain	2,500.24	1,809.56	3,219.81	1,493.63	1,103.01	1,894.35	-0.29	-0.32	-0.26
Sri Lanka	2,398.88	1,463.53	3,316.20	2,223.68	1,459.81	3,044.80	0.30	0.33	0.32
Sudan	3,004.58	2,518.93	3,486.42	2,436.87	1,943.48	2,926.65	0.58	0.52	0.60
Suriname	2,131.10	1,677.03	2,570.66	1,952.33	1,430.66	2,472.07	0.05	0.02	0.06
Swaziland	985.66	830.56	1,159.11	1,731.10	1,479.17	1,988.34	0.28	0.24	0.27
Sweden	4,716.01	3,347.73	6,117.48	2,351.19	1,808.82	2,894.64	-0.40	-0.38	-0.41
Switzerland	2,923.19	2,126.13	3,757.92	1,578.23	1,154.40	2,011.61	-0.33	-0.36	-0.30
Syria	2,716.11	2,251.02	3,166.28	2,515.96	1,951.20	3,066.94	-0.23	-0.06	-0.32
Taiwan	1,362.12	807.25	1,877.19	1,230.14	839.75	1,621.85	-0.11	0.00	-0.14
Tajikistan	2,547.56	2,317.52	2,780.34	2,107.96	1,643.69	2,561.43	0.62	0.42	0.77
Tanzania	923	794.11	1,054.17	844.06	710.42	979.34	0.74	0.69	0.73
Thailand	1,657.66	1,149.21	2,175.48	1,634.45	1,087.95	2,196.68	-0.05	-0.04	-0.06
The Bahamas	1,902.21	1,471.51	2,338.92	1,977.39	1,536.65	2,437.03	0.14	0.10	0.18
The Gambia	790.77	765.73	815.45	669.45	552.69	788.52	0.58	0.43	0.71
Timor-Leste	1,272.33	1,119.47	1,421.34	1,238.93	1,151.86	1,323.31	1.54	1.72	1.39
Togo	959.27	893.33	1,026.92	1,088.88	779.31	1,405.77	0.71	0.39	0.94
Tonga	1,701.24	1,137.34	2,251.45	2,056.55	1,204.59	2,903.88	0.33	0.23	0.36
Trinidad and Tobago	3,028.29	2,447.79	3,613.08	3,262.41	2,381.88	4,166.34	0.02	-0.03	0.05
Tunisia	1,130.82	828.37	1,429.60	1,422.06	940.48	1,914.96	0.69	0.59	0.72
Turkey	3,153.46	1,958.37	4,377.88	1,513.60	982.58	2,063.40	-0.09	-0.06	-0.10
Turkmenistan	3,789.00	3,216.60	4,377.44	4,099.27	3,051.09	5,183.24	0.81	0.68	0.89
Uganda	558.6	480.12	638.1	592.88	423.58	762.33	1.15	0.82	1.34
Ukraine	7,221.78	6,673.97	7,859.26	10,674.89	9,261.36	12,344.46	0.37	0.34	0.40
United Arab Emirates	1,625.77	949.23	1,977.15	1,952.15	653.27	2,425.56	0.50	-0.02	0.49
United Kingdom	4,916.01	3,531.15	6,380.87	2,015.25	1,389.56	2,656.58	-0.49	-0.53	-0.47
United States	3,665.36	2,780.01	4,596.46	2,406.44	1,804.85	3,028.37	-0.29	-0.33	-0.26
Uruguay	3,307.22	2,376.18	4,295.61	1,940.25	1,468.92	2,445.17	-0.34	-0.33	-0.34
Uzbekistan	2,973.84	2,707.12	3,246.45	3,486.08	2,785.58	4,209.58	0.57	0.44	0.68
Vanuatu	3,015.43	1,840.00	4,126.88	3,642.00	2,460.57	4,794.13	0.38	0.49	0.35
Venezuela	1,646.03	1,266.51	2,019.62	1,912.57	1,293.64	2,536.77	0.13	0.09	0.13
Vietnam	1,129.28	1,094.36	1,165.28	1,204.76	1,011.69	1,401.98	0.55	0.42	0.66
Virgin Islands, U.S.	2,701.30	2,045.63	3,395.81	4,599.15	3,310.31	6,015.73	0.27	0.26	0.28
Yemen	3,166.43	3,171.16	3,161.62	2,688.71	2,520.45	2,853.51	0.79	0.70	0.88
Zambia	859.29	972.65	744.01	1,313.28	986.45	1,641.03	1.56	0.77	2.52
Zimbabwe	590.52	691.72	488.47	704.16	673.61	735.6	0.04	-0.16	0.31
**Ischemic stroke**									
Afghanistan	899.92	1,248.63	567.96	1,043.83	1,381.64	726.64	0.91	0.90	1.02
Albania	276.5	269.93	282.75	440.82	396.39	485.96	0.79	0.69	0.88
Algeria	364.22	400.47	328.86	485.22	527.68	443.35	0.90	0.86	0.94
American Samoa	366.65	267.34	460.65	258.95	247.01	270.84	-0.14	-0.03	-0.21
Andorra	421.44	383.19	461.77	345.53	289.04	404.39	-0.18	-0.23	-0.13
Angola	453.53	542.5	362.36	326.33	412.91	238.61	0.46	0.48	0.39
Antigua and Barbuda	883.37	927.04	836.66	433.21	428.95	437.86	-0.46	-0.50	-0.41
Argentina	957.98	840.46	1,080.13	397.55	378.47	417.46	-0.51	-0.49	-0.53
Armenia	775.2	845.54	700.66	1,017.67	964.37	1,079.07	0.46	0.28	0.69
Australia	465.62	466.06	465.18	286.25	289.86	282.63	-0.29	-0.31	-0.27
Austria	1,069.57	1,188.05	940.47	403.38	421.57	384.28	-0.55	-0.58	-0.51
Azerbaijan	443.56	450.86	435.93	437.98	394.73	481.75	0.49	0.33	0.65
Bahrain	198.86	238.95	169.38	139.44	163.08	124.96	0.02	-0.05	0.09
Bangladesh	516.26	589.03	446.72	755.8	737.84	773.41	2.40	2.15	2.75

Barbados	971.75	988.79	953.38	716.03	740.55	689.39	-0.30	-0.31	-0.30
Belarus	1,880.33	2,067.66	1,668.15	2,303.33	2,238.52	2,378.52	0.06	0.01	0.15
Belgium	813	876.17	746.9	427.17	435.3	418.71	-0.40	-0.44	-0.35
Belize	378.54	374.47	382.54	278.68	242.22	315.36	-0.12	-0.19	-0.06
Benin	342.59	407.29	273.76	373.77	421.7	325.66	1.08	0.96	1.24
Bermuda	568.22	525.59	611.4	320.27	283.95	357.28	-0.28	-0.35	-0.23
Bhutan	280.83	293.68	268.9	279.95	243.64	311.22	1.24	1.02	1.41
Bolivia	460.69	517	404.77	405.47	433.85	377.19	0.90	0.82	1.00
Bosnia and Herzegovina	806.18	862.22	750.07	1,520.89	1,463.36	1,578.95	0.92	0.66	1.20
Botswana	312.81	371.59	252.65	422.09	468.11	376.02	0.05	0.00	0.14
Brazil	359.74	313.11	407.12	303.8	255.03	354.19	0.06	0.02	0.10
Brunei	225.48	214.48	235.33	239.11	220.85	256.29	0.08	0.04	0.11
Bulgaria	2,009.11	1,922.91	2,097.75	2,288.97	2,237.75	2,342.72	0.11	0.11	0.11
Burkina Faso	248.83	293.77	201.8	204.47	253.65	154.66	0.53	0.57	0.44
Burundi	777.66	924.14	626.05	326.59	373.9	278.26	-0.06	-0.08	-0.02
Cambodia	392.96	441.59	340.86	416.46	411.57	421.61	1.59	1.34	1.95
Cameroon	366.29	433.55	298.18	391.57	434.51	348.67	0.42	0.33	0.53
Canada	444.51	435.91	453.25	346.33	341.33	351.39	-0.19	-0.22	-0.16
Cape Verde	553.32	565.81	539.51	459.34	496.54	421.13	0.28	0.37	0.20
Central African Republic	735.38	838.88	628.37	721.09	812.6	627.15	0.20	0.14	0.22
Chad	328.54	403.36	252.62	330.23	386.41	274.19	0.46	0.38	0.56
Chile	733.88	680.81	788.38	460.41	447.61	473.55	-0.28	-0.29	-0.27
China	682.91	636.24	727.18	891.41	680.79	1,089.81	0.94	0.74	1.08
Colombia	245.08	244.11	246.06	169.74	165.23	174.38	-0.08	-0.16	0.00
Comoros	448.77	542.27	355.88	255.21	307.53	203.84	0.23	0.23	0.24
Congo	662.1	814.19	509.22	474.12	685.11	263.45	0.02	0.06	-0.18
Costa Rica	226.48	223.01	229.9	165.4	160.44	170.35	-0.23	-0.25	-0.22
Cote d'Ivoire	376.31	406.1	348.86	398.2	422.34	374.89	0.50	0.48	0.48
Croatia	1,463.04	1,485.47	1,439.21	1,312.41	1,363.51	1,257.57	-0.03	-0.05	-0.02
Cuba	637.49	626.79	648.04	739.17	712.65	765.43	0.11	0.09	0.13
Cyprus	565.89	509.57	623.64	278.12	231.06	328.42	-0.43	-0.47	-0.39
Czech Republic	2,001.74	2,097.02	1,900.74	964.27	1,009.82	917.26	-0.40	-0.42	-0.38
Democratic Republic of the Congo	363.29	491.33	232.08	352.4	422.31	282.13	0.58	0.40	0.98
Denmark	772.11	772.15	772.07	446.54	423.63	469.82	-0.28	-0.34	-0.23
Djibouti	307.8	314.08	301.59	397.77	405.47	390.13	0.78	0.80	0.73
Dominica	514.61	575.07	453.7	525.61	512.68	538.74	-0.08	-0.12	-0.01
Dominican Republic	367.83	337.9	397.37	432.11	401.8	462.62	0.58	0.63	0.54
Ecuador	346.85	313.63	379.7	285.11	253.55	316.68	0.15	0.15	0.15
Egypt	609.24	645.55	573.18	450.24	457.09	443.54	0.28	0.28	0.29
El Salvador	363.2	335.84	391.86	186.64	175.89	198.79	-0.24	-0.23	-0.25
Equatorial Guinea	849.31	1,044.81	662.26	293.04	342.67	245.85	-0.34	-0.37	-0.28
Eritrea	383.96	478.71	288.01	362.07	470.73	253.8	0.66	0.66	0.56
Estonia	2,649.29	2,887.81	2,377.86	1,200.20	1,286.79	1,098.54	-0.42	-0.47	-0.38
Ethiopia	514.07	552.33	475.57	319.46	351.75	287.07	0.95	0.95	0.90
Federated States of Micronesia	643.86	534.81	748.24	589.9	489.86	685.18	0.23	0.23	0.24
Fiji	569.52	510.33	626.88	591.6	558.5	623.6	-0.10	-0.08	-0.11
Finland	1,055.05	1,154.54	949.5	599.15	609.74	588.23	-0.35	-0.41	-0.27
France	550.01	554.7	545.08	359.98	352.15	368.31	-0.27	-0.32	-0.23
Gabon	670.75	738.69	599.97	428.37	540.53	318.74	-0.04	0.06	-0.19
Georgia	1,281.05	1,206.91	1,362.82	1,491.82	1,232.92	1,775.02	0.18	0.10	0.25
Germany	1,033.51	1,156.69	901.17	572.74	576.59	568.76	-0.38	-0.44	-0.29
Ghana	450.2	723.12	185.9	495.19	680.88	307.63	0.85	0.64	1.64
Greece	1,152.59	1,280.80	1,021.08	862.5	952.03	768.55	-0.24	-0.25	-0.23
Greenland	370.84	308.55	424.74	331.44	232.38	417.33	0.25	0.07	0.35
Grenada	1,106.40	1,222.83	986.92	681.36	733.66	629.39	-0.33	-0.33	-0.31
Guam	395.97	359.42	427.9	650.76	600.71	699.41	0.22	0.23	0.20
Guatemala	179.95	178.72	181.19	168.12	173.36	162.65	0.71	0.84	0.60
Guinea	398.91	558.03	236.23	426.88	534.74	319.53	1.01	0.83	1.47
Guinea-Bissau	489.14	616.29	358.93	495.08	608.83	379.92	0.50	0.45	0.58
Guyana	1,008.86	881.88	1,139.96	778.69	681	875.43	-0.12	-0.10	-0.14
Haiti	1,103.23	1,406.33	791.21	778.22	952.91	599.72	0.36	0.28	0.48

Honduras	385.42	380.05	390.76	435.74	423.25	448.22	0.77	0.69	0.85
Hungary	2,244.55	2,198.83	2,294.01	1,266.97	1,267.89	1,265.95	-0.28	-0.31	-0.25
Iceland	424.67	421.1	428.22	247.13	215.59	279.34	-0.32	-0.40	-0.25
India	499.1	481.61	515.38	516.79	442.09	586.21	0.78	0.72	0.81
Indonesia	455.24	503.31	407.58	650.35	674.17	626.88	1.21	1.15	1.30
Iran	428.37	374.48	480.31	461.13	378.55	542.47	1.06	1.05	1.05
Iraq	664.23	732.5	597.2	549.47	563.98	535.3	0.03	0.04	0.04
Ireland	506.71	540	473.12	236.68	226.4	247.27	-0.43	-0.48	-0.36
Israel	455.23	455.69	454.77	230.12	224.97	235.36	-0.39	-0.41	-0.37
Italy	906.88	908.74	904.9	538.1	564.88	509.64	-0.35	-0.36	-0.35
Jamaica	829.25	907.4	749.11	713.69	735.53	691.68	-0.18	-0.20	-0.15
Japan	812.5	748.89	878.4	729.15	629.24	834.37	-0.20	-0.24	-0.17
Jordan	524.58	591.17	464.32	293.53	277.14	309.13	-0.16	-0.27	-0.03
Kazakhstan	1,245.92	1,346.96	1,138.55	1,313.40	1,176.05	1,460.56	0.15	0.00	0.34
Kenya	197.05	222.12	171.8	183.74	188.78	178.71	0.29	0.20	0.42
Kiribati	1,041.37	745.92	1,342.98	1,123.76	685.7	1,573.10	0.53	0.41	0.55
Kuwait	135.32	133.94	136.52	180.69	152.65	202.57	0.65	0.33	0.93
Kyrgyzstan	1,094.96	1,144.38	1,043.41	893.56	717.41	1,073.46	0.15	-0.06	0.40
Laos	532.95	620.87	444.91	376.92	383.04	370.75	0.96	0.78	1.23
Latvia	2,701.75	2,971.39	2,391.16	2,233.27	2,413.48	2,023.41	-0.04	-0.08	0.01
Lebanon	494.92	663.74	321.46	328.65	462.63	196.01	0.16	0.08	0.18
Lesotho	447.42	470.3	423.2	675.5	780.27	568.61	-0.05	0.00	-0.13
Liberia	337.38	428.07	246.17	320.69	411.62	231.23	1.46	1.34	1.55
Libya	251.9	284.46	222.75	382.75	398.93	366.75	0.61	0.64	0.61
Lithuania	1,437.22	1,543.91	1,318.48	1,591.46	1,640.50	1,534.44	0.11	0.08	0.15
Luxembourg	1,027.94	1,096.44	956.64	300.68	312.28	289.04	-0.58	-0.62	-0.54
Macedonia	1,396.43	1,342.98	1,449.26	1,667.07	1,608.59	1,726.07	0.29	0.29	0.28
Madagascar	521.75	583.74	459.57	488.11	557.87	417.91	0.75	0.81	0.64
Malawi	267.39	340.04	192.2	229.82	256.76	202.79	0.86	0.67	1.22
Malaysia	299.25	292.65	305.64	318.87	270.15	368.5	0.13	0.05	0.20
Maldives	212.77	172.75	250.77	146.08	101.19	190.72	0.84	0.65	0.95
Mali	469.59	647.08	288.24	286.75	403.9	171.93	0.08	0.09	0.06
Malta	510.46	490.45	531.19	364.08	357.01	371.2	-0.28	-0.27	-0.30
Marshall Islands	519.02	405.04	628.01	540.17	438.4	638.46	0.25	0.28	0.23
Mauritania	391.69	504.23	278.47	279.2	377.03	182.71	0.30	0.32	0.22
Mauritius	555.35	481.07	629.94	432.22	383.84	481.8	-0.24	-0.24	-0.24
Mexico	186.85	186.13	187.57	180.55	178.19	182.93	0.27	0.23	0.31
Moldova	1,459.30	1,539.65	1,371.15	1,295.37	1,248.26	1,346.30	0.08	0.01	0.17
Mongolia	223.64	266.22	180.05	330.94	295.38	367.22	1.17	0.89	1.66
Montenegro	376.28	410.86	341.39	599.76	586.46	613.37	0.54	0.42	0.69
Morocco	463.67	521.5	405.62	527.54	589.42	464.21	0.71	0.69	0.72
Mozambique	400.34	412.63	386.95	445.21	452.18	437.92	1.03	0.95	1.06
Myanmar	604.9	576.09	634.88	622.04	514.57	734.45	0.81	0.69	0.91
Namibia	403.63	452.78	352.35	294.77	292.05	297.63	-0.15	-0.23	-0.04
Nepal	332.15	333.71	330.56	382.27	321.44	446.96	1.63	1.36	1.88
Netherlands	549.97	568.21	531.31	368.28	359.82	376.89	-0.27	-0.33	-0.20
New Zealand	460.68	451.88	469.76	302.45	306.02	298.74	-0.21	-0.23	-0.20
Nicaragua	189.51	180.31	198.86	166.94	157.19	176.98	0.57	0.58	0.57
Niger	277.81	336.96	216.66	289.8	317.65	262.4	1.43	1.19	1.81
Nigeria	321.78	403.62	241.57	193.15	210.88	176.08	0.09	-0.03	0.30
North Korea	569.05	613.59	521.86	1,131.97	1,096.22	1,169.32	1.11	0.98	1.29
Northern Mariana Islands	252.08	219.9	281.01	157.36	100.18	214.95	-0.22	-0.38	-0.11
Norway	817.53	822.97	811.98	343.13	356.94	329.49	-0.45	-0.47	-0.44
Oman	57.52	65.88	50.85	103.5	114.45	97.95	2.06	2.14	2.21
Pakistan	460.91	402.17	515.41	522.62	430.99	609.37	0.71	0.70	0.70
Palestine	148.89	165.86	132.34	244.67	226.04	262.78	1.49	1.18	1.90
Panama	365.37	324.8	405.05	268.79	231.53	305.85	-0.22	-0.24	-0.21
Papua New Guinea	717.15	739.4	695.92	705.75	776.69	637.58	0.35	0.41	0.27
Paraguay	254.37	253.65	255.08	246.31	224.71	267.26	0.04	0.00	0.07
Peru	345.66	335.9	355.42	169.54	162.91	176.18	0.01	-0.02	0.04
Philippines	202.08	182.6	221.16	336.15	301.05	370.61	1.30	1.28	1.31

Poland	1,166.75	1,287.63	1,039.63	928.98	918.34	940.43	-0.08	-0.19	0.06
Portugal	1,825.22	1,802.61	1,849.53	758.95	753.71	764.53	-0.50	-0.51	-0.49
Puerto Rico	399.05	368.07	431.98	322.35	327.81	316.45	-0.13	-0.13	-0.15
Qatar	141.01	164.88	129.23	81.25	90.64	77.74	-0.20	-0.25	-0.16
Romania	1,695.39	1,734.25	1,655.41	1,922.16	1,864.99	1,983.01	0.19	0.15	0.23
Russia	2,665.94	3,029.52	2,253.07	2,595.79	2,632.71	2,552.43	-0.10	-0.16	0.01
Rwanda	518.1	612.47	420.39	276.82	302.88	248.47	0.25	0.16	0.35
Saint Lucia	936.93	991.15	881.09	648.06	694.36	599.99	-0.18	-0.17	-0.19
Saint Vincent and the Grenadines	673.8	809.18	537.07	611.46	638.37	585.05	-0.08	-0.15	0.04
Samoa	501.89	392.53	602.69	431.22	411.97	449.29	0.00	0.07	-0.03
Sao Tome and Principe	491.07	603.98	376.07	440.44	562.5	317.46	0.60	0.63	0.50
Saudi Arabia	231.83	237.3	227.53	227.81	200.11	249.08	0.72	0.60	0.81
Senegal	335.21	391.39	278.49	366.4	454.47	275.12	1.13	1.23	0.90
Serbia	1,708.80	1,779.34	1,636.37	2,147.74	2,214.36	2,077.87	0.23	0.20	0.25
Seychelles	514.13	469.78	558.68	343.33	297.64	387.81	-0.26	-0.32	-0.21
Sierra Leone	334.08	321.48	346.95	401.96	482.37	319.89	1.15	1.57	0.71
Singapore	540.11	546.3	534.07	384.92	393.89	375.63	-0.18	-0.20	-0.17
Slovakia	1,145.97	1,141.55	1,150.60	886.42	855.49	919.06	-0.07	-0.16	0.00
Slovenia	1,301.62	1,314.91	1,287.53	672.16	674.39	669.88	-0.39	-0.42	-0.37
Solomon Islands	765.41	706.82	820.89	712.41	675.19	748.47	0.21	0.23	0.20
Somalia	602.35	685.04	518.18	414.79	465.33	363.77	0.03	0.01	0.05
South Africa	478.06	615.87	337.05	488.4	557.41	416.95	-0.03	-0.14	0.17
South Korea	1,022.14	1,014.23	1,030.01	660.68	615.01	706.91	-0.26	-0.32	-0.20
South Sudan	375.59	461.61	288.76	364.06	447.19	281.18	0.55	0.55	0.57
Spain	840.16	879.3	799.37	360.52	378.83	341.75	-0.49	-0.52	-0.46
Sri Lanka	214.73	172.37	256.28	519.3	447.92	596.04	2.41	2.48	2.34
Sudan	686.25	763.35	609.75	609.01	631.8	586.39	0.73	0.64	0.83
Suriname	623	628.95	617.24	638.29	618.08	658.42	0.17	0.17	0.17
Swaziland	336.91	378.86	290	495.33	565.21	423.98	0.08	0.05	0.10
Sweden	836.06	832.6	839.61	493.69	504.02	483.35	-0.29	-0.31	-0.28
Switzerland	596.94	597.63	596.21	289.38	291.54	287.17	-0.40	-0.43	-0.37
Syria	594.17	576.09	611.67	439.27	411.9	465.97	-0.38	-0.23	-0.47
Taiwan	580.69	469.15	684.22	434.02	361.75	506.53	-0.26	-0.26	-0.26
Tajikistan	558.09	565.32	550.78	421.84	386.04	456.8	0.48	0.37	0.59
Tanzania	298.66	342.85	253.69	271.95	287.57	256.14	0.73	0.59	0.89
Thailand	215.86	211.99	219.81	407.2	349.45	466.61	0.83	0.68	0.98
The Bahamas	528.01	531.98	523.99	494.65	509.22	479.46	0.03	0.01	0.04
The Gambia	294.22	366.71	222.77	243.94	267.68	219.75	0.54	0.44	0.75
Timor-Leste	237.98	273.78	203.09	294.21	342.85	247.08	2.22	2.32	2.13
Togo	375.84	467.7	281.61	353.3	377.36	328.68	0.42	0.28	0.66
Tonga	401.82	443.46	361.2	416.03	421.91	410.19	0.14	0.10	0.19
Trinidad and Tobago	791.21	761.95	820.69	625.01	554.24	697.66	-0.25	-0.27	-0.23
Tunisia	499.19	437.42	560.22	549.43	479.44	621.06	0.48	0.54	0.43
Turkey	355.26	410.68	298.49	324.25	319.18	329.51	0.74	0.46	1.12
Turkmenistan	146.87	170.31	122.78	160.52	169.06	151.68	0.83	0.76	0.97
Uganda	365.01	427.6	301.6	256.62	252.73	260.52	0.43	0.23	0.70
Ukraine	2,508.82	2,810.97	2,157.20	2,124.03	2,213.15	2,018.76	-0.22	-0.24	-0.17
United Arab Emirates	317.27	346.26	302.22	294.57	245.28	312.54	0.16	0.01	0.26
United Kingdom	911.02	978.47	839.67	469.99	483.69	455.95	-0.36	-0.41	-0.31
United States	502.81	515.61	489.35	383.21	383.23	383.19	-0.18	-0.24	-0.12
Uruguay	1,272.45	1,231.62	1,315.79	833.47	895.57	766.95	-0.27	-0.22	-0.32
Uzbekistan	658.47	638.75	678.62	660.18	554.55	769.28	0.34	0.21	0.47
Vanuatu	838.17	712.46	957.03	801.61	750.2	851.74	0.09	0.18	0.03
Venezuela	314.92	304.63	325.05	220.38	203.68	237.22	-0.32	-0.29	-0.34
Vietnam	527.83	528.32	527.31	647.99	589.62	707.61	0.78	0.72	0.86
Virgin Islands, U.S.	371.78	335.65	410.05	620.64	532.34	717.69	0.25	0.24	0.26
Yemen	584.26	720.45	445.57	592.05	701.32	485.03	1.14	1.09	1.27
Zambia	260.6	358.49	161.05	307.3	330.95	283.59	0.98	0.61	1.82
Zimbabwe	167.49	213.94	120.66	185.07	206.08	163.45	-0.03	-0.17	0.18
**Other cardiovascular and circulatory diseases**									
Afghanistan	383.84	522.23	252.11	522.74	728.38	329.64	1.24	1.39	1.07

Albania	351.82	292.88	407.96	557.78	457.74	659.43	0.78	0.80	0.76
Algeria	409.66	453.36	367.04	314.65	358.48	271.43	0.10	0.12	0.06
American Samoa	195.91	108.51	278.65	215.27	183.54	246.84	0.34	0.79	0.19
Andorra	231.4	218.49	245.01	253.93	230.74	278.09	0.10	0.08	0.12
Angola	597.55	624.94	569.48	432	483.16	380.17	0.46	0.49	0.41
Antigua and Barbuda	252.55	220.32	287.03	392.82	333.21	457.83	0.72	0.65	0.79
Argentina	277.79	273.75	281.98	354.25	367.04	340.91	0.51	0.53	0.48
Armenia	34.68	35.84	33.45	71.68	74.33	68.62	1.31	1.33	1.25
Australia	176.68	173.15	180.23	187.93	186.13	189.74	0.23	0.19	0.27
Austria	415.61	471.38	354.86	316.27	351.06	279.76	-0.10	-0.13	-0.05
Azerbaijan	61.27	57.95	64.73	99.74	81.42	118.27	1.45	1.14	1.73
Bahrain	171.63	175.66	168.66	98.89	109.87	92.17	-0.17	-0.13	-0.19
Bangladesh	15.3	15.71	14.92	20.5	18.71	22.26	2.11	1.99	2.23
Barbados	240.33	245.02	235.28	511.2	532.47	488.07	1.02	1.00	1.03
Belarus	56.62	63.26	49.09	64.85	65.06	64.6	-0.01	-0.04	0.06
Belgium	349.38	371.59	326.14	417.04	426.32	407.38	0.36	0.28	0.45
Belize	212.45	187.83	236.65	325.55	287.58	363.74	0.83	0.93	0.75
Benin	597.7	646.8	545.45	425.45	413.1	437.85	0.37	0.23	0.53
Bermuda	275.46	245.83	305.47	336.13	276.28	397.13	0.55	0.35	0.72
Bhutan	17.3	17.36	17.24	24.25	20.44	27.53	2.14	1.87	2.33
Bolivia	697.05	688.45	705.58	319.91	288.6	351.11	-0.01	-0.09	0.07
Bosnia and Herzegovina	460.93	417.13	504.78	367.28	277.91	457.48	-0.19	-0.35	-0.05
Botswana	128.9	157.05	100.08	160.52	188.13	132.9	-0.03	-0.04	0.00
Brazil	325.58	287.8	363.96	331.59	302.96	361.19	0.28	0.32	0.25
Brunei	101.01	98.01	103.69	110.65	102.15	118.65	0.12	0.06	0.17
Bulgaria	707.28	546.46	872.64	886.69	699.39	1,083.27	0.22	0.22	0.23
Burkina Faso	666.16	763.65	564.12	483.36	531.51	434.58	0.35	0.27	0.45
Burundi	1,138.71	1,140.12	1,137.25	454.18	445.68	462.86	-0.11	-0.11	-0.10
Cambodia	340.75	312.49	371.02	219.47	189.23	251.22	0.57	0.52	0.61
Cameroon	664.18	727.37	600.2	491.83	485.52	498.13	-0.01	-0.10	0.09
Canada	192.87	197.94	187.71	212.75	217.51	207.92	0.15	0.09	0.21
Cape Verde	895.8	802.16	999.35	590.93	658.66	521.39	0.02	0.29	-0.20
Central African Republic	627.96	662.76	591.98	646.46	663.43	629.04	0.26	0.18	0.29
Chad	526.26	625.75	425.33	437.87	472.69	403.14	0.22	0.10	0.38
Chile	71.29	69.68	72.94	138.4	144.84	131.79	1.23	1.24	1.19
China	415.91	400.17	430.84	206.95	174.7	237.33	-0.26	-0.29	-0.24
Colombia	226.95	221.9	232.06	268.17	279.72	256.27	0.57	0.56	0.56
Comoros	802.83	764.96	840.46	405.22	397.91	412.4	0.09	0.13	0.06
Congo	544.02	592.74	495.06	442.65	566.09	319.39	0.16	0.20	0.03
Costa Rica	115.62	114.58	116.64	216.03	209.23	222.81	0.96	0.89	1.02
Cote d'Ivoire	590.8	609.35	573.7	455.55	444.09	466.62	0.09	0.05	0.13
Croatia	777.36	719.8	838.52	457.65	428.73	488.69	-0.37	-0.39	-0.35
Cuba	84.26	79.7	88.76	232.58	198.48	266.35	1.65	1.38	1.89
Cyprus	357.29	356.82	357.78	260.11	249.39	271.56	-0.15	-0.18	-0.13
Czech Republic	540.36	538.88	541.93	565	553.74	576.62	0.30	0.23	0.36
Democratic Republic of the Congo	369.92	431.68	306.64	347.96	383.78	311.95	0.53	0.44	0.66
Denmark	468.34	438.58	498.96	315.02	296.71	333.63	-0.17	-0.18	-0.16
Djibouti	670.01	559.01	779.7	668.79	542.28	794.17	0.37	0.36	0.36
Dominica	187.83	201.02	174.55	423.17	413.21	433.29	1.04	1.04	1.08
Dominican Republic	222.15	188.19	255.66	237.13	208.73	265.71	0.44	0.52	0.38
Ecuador	130.07	113.8	146.17	152.23	128.02	176.44	0.64	0.60	0.66
Egypt	881.83	840.42	922.97	556	479.9	630.53	0.09	0.03	0.14
El Salvador	217.79	205.01	231.17	225.42	231.5	218.56	0.54	0.67	0.39
Equatorial Guinea	740.01	789.73	692.44	438.15	468.42	409.37	0.14	0.13	0.15
Eritrea	701.99	713.19	690.65	555.94	614.77	497.33	0.39	0.46	0.28
Estonia	88.64	88.36	88.95	225.14	220.59	230.48	2.24	1.98	2.50
Ethiopia	882.54	878.79	886.32	380.23	379.44	381.02	0.35	0.33	0.35
Federated States of Micronesia	356.56	304.89	406.03	346.58	332.52	359.97	0.30	0.46	0.20
Fiji	405.71	357.05	452.85	711.94	702.74	720.83	0.53	0.65	0.43
Finland	186.58	201.71	170.53	307.25	308.4	306.07	0.89	0.70	1.11
France	567.05	514.72	622.24	512.42	458.89	569.42	0.00	-0.04	0.04

Gabon	605.39	609.91	600.68	436.38	483.4	390.42	0.08	0.15	-0.01
Georgia	65.47	61.45	69.9	300.02	195.56	414.28	3.63	2.43	4.70
Germany	299.45	326.55	270.35	498.58	506.2	490.71	0.87	0.75	1.04
Ghana	292.74	209.79	373.08	288.18	233.29	343.62	0.67	0.99	0.49
Greece	220.59	228.92	212.05	174.81	180.82	168.49	-0.20	-0.21	-0.19
Greenland	381.8	265.84	482.16	423.12	308.46	522.53	0.55	0.65	0.49
Grenada	233.78	211.87	256.26	411.03	362.87	458.89	0.93	0.92	0.93
Guam	153.14	145.17	160.11	302.08	302.96	301.22	0.46	0.54	0.39
Guatemala	135.45	120.96	150.15	139.11	124.82	154.02	0.88	0.96	0.82
Guinea	679.44	798.21	558.02	473.17	475.69	470.67	0.31	0.15	0.56
Guinea-Bissau	759.98	876.75	640.39	583.27	623.32	542.73	0.16	0.07	0.29
Guyana	193.02	171.64	215.09	342.07	307.27	376.54	1.01	1.08	0.96
Haiti	414.68	459.71	368.32	355.13	381.21	328.49	0.65	0.56	0.74
Honduras	132.65	129.71	135.58	109.85	98.72	120.98	0.30	0.16	0.44
Hungary	590.83	558.66	625.63	407.89	387.08	430.82	-0.12	-0.18	-0.06
Iceland	196.84	199.14	194.57	191.77	175.12	208.76	0.13	0.04	0.23
India	17.06	17.8	16.38	29.43	26.29	32.35	1.96	1.77	2.14
Indonesia	179.61	155.01	204.01	184.5	166.97	201.78	0.59	0.72	0.48
Iran	1,310.89	1,050.43	1,561.89	692.01	514.23	867.13	0.01	-0.01	0.01
Iraq	633.05	596.36	669.07	467.76	432.64	502.01	-0.08	-0.02	-0.13
Ireland	202.55	206.64	198.42	166.96	157.01	177.2	0.01	-0.06	0.09
Israel	89.04	92.74	85.33	165.11	175.04	155.01	1.24	1.25	1.22
Italy	212.21	212.89	211.5	245.23	248.88	241.35	0.27	0.21	0.32
Jamaica	207.16	189.48	225.3	314.4	300.93	327.97	0.45	0.57	0.34
Japan	164.37	148.17	181.16	238.6	230.9	246.71	0.29	0.41	0.19
Jordan	229.68	270.28	192.95	165.5	180.26	151.46	0.08	0.04	0.14
Kazakhstan	100.47	81.89	120.22	317.07	181.54	462.3	2.44	1.53	3.03
Kenya	297.06	309.13	284.9	287.22	274.16	300.28	0.34	0.26	0.43
Kiribati	368.12	289.89	447.97	513.12	495.27	531.42	0.97	1.62	0.57
Kuwait	94.98	105.47	85.8	115.32	124.82	107.9	0.50	0.38	0.63
Kyrgyzstan	66.09	73.08	58.8	103	86	120.36	1.20	0.75	1.78
Laos	447.82	425.07	470.6	239.46	201.37	277.92	0.48	0.37	0.58
Latvia	75.15	87.05	61.44	172.92	149.38	200.33	1.67	0.94	2.88
Lebanon	323.21	333.3	312.83	245.02	287.66	202.82	0.32	0.33	0.25
Lesotho	161.77	193.63	128.03	235.31	273.23	196.62	-0.09	-0.15	-0.02
Liberia	644.06	741.08	546.5	352.83	387.43	318.8	0.42	0.28	0.59
Libya	558.61	552.77	563.84	365.65	359.12	372.11	-0.31	-0.24	-0.36
Lithuania	98.11	102.82	92.87	292.54	238.52	355.36	2.00	1.36	2.79
Luxembourg	691.78	686.1	697.69	457.34	449.62	465.09	-0.06	-0.12	0.01
Macedonia	445.68	380.42	510.17	348.81	273.55	424.74	-0.16	-0.23	-0.10
Madagascar	980.23	916.02	1,044.65	785.89	712.01	860.24	0.50	0.47	0.49
Malawi	546.33	601.12	489.62	367.51	353.59	381.49	0.46	0.31	0.64
Malaysia	378.96	351.74	405.38	352.71	334.63	371.13	-0.02	0.08	-0.09
Maldives	507.55	424.56	586.37	270.87	192.95	348.35	0.43	0.27	0.52
Mali	701.84	892.91	506.62	394.68	470.56	320.3	0.00	-0.07	0.13
Malta	229.25	216.58	242.36	248.55	234.38	262.83	0.09	0.08	0.09
Marshall Islands	348.64	300	395.15	465.07	503.07	428.37	0.61	0.99	0.32
Mauritania	518.63	599.8	436.97	330.05	377.01	283.73	0.17	0.13	0.22
Mauritius	138.85	106.89	170.95	165.4	150	181.18	0.17	0.34	0.05
Mexico	160.38	142.47	178.48	163.64	143.6	183.89	0.34	0.29	0.38
Moldova	66.42	59.5	74	99.64	75.01	126.27	0.82	0.57	1.03
Mongolia	219.03	159.71	279.75	114.74	79.67	150.52	-0.23	-0.15	-0.30
Montenegro	272.65	247.56	297.95	312.79	283.14	343.15	0.11	0.14	0.08
Morocco	967.92	897.89	1,038.22	579.91	527.11	633.94	-0.10	-0.12	-0.08
Mozambique	653.82	621.47	689.09	637.46	571.74	706.2	0.79	0.66	0.88
Myanmar	324.15	305.76	343.28	284.4	278.91	290.14	0.54	0.72	0.39
Namibia	157.16	184.66	128.46	118.62	134.56	101.83	-0.12	-0.13	-0.10
Nepal	14.09	13.19	15.01	18.77	15.33	22.42	2.04	1.85	2.18
Netherlands	328.92	344.37	313.13	368.13	361.34	375.03	0.22	0.10	0.34
New Zealand	186.45	173.64	199.65	182.85	171.92	194.19	0.17	0.12	0.23
Nicaragua	120.42	121.72	119.09	138.67	143.73	133.46	1.05	1.13	0.97

Niger	555.36	628.14	480.13	316.11	341.31	291.32	0.33	0.27	0.42
Nigeria	514.13	611.57	418.65	302.25	324.69	280.63	0.07	-0.01	0.20
North Korea	337.72	362.53	311.44	287.1	315.48	257.45	-0.09	-0.03	-0.14
Northern Mariana Islands	177.13	168.29	185.08	172.52	144.52	200.72	0.22	0.16	0.27
Norway	352.15	345.28	359.17	257.24	261.63	252.9	-0.05	-0.08	-0.02
Oman	3,094.79	2,934.15	3,222.94	1,278.70	1,020.90	1,409.42	-0.30	-0.37	-0.27
Pakistan	18.65	13.78	23.17	31.37	25.16	37.26	1.53	1.89	1.31
Palestine	279.31	274.2	284.3	246.45	236.16	256.45	0.34	0.37	0.32
Panama	104.3	98.2	110.26	188.42	176.28	200.49	0.92	0.92	0.91
Papua New Guinea	398.97	397.2	400.65	402.19	420.98	384.13	0.38	0.42	0.34
Paraguay	163.04	136.69	188.78	217.14	187.12	246.24	0.42	0.55	0.33
Peru	142.67	109.1	176.24	159.53	128.65	190.46	1.30	1.37	1.27
Philippines	35.2	31.9	38.42	81.62	72.81	90.28	2.21	2.16	2.24
Poland	441.45	325.85	563.04	333.73	267.99	404.48	-0.13	-0.07	-0.16
Portugal	293.44	274.37	313.95	303.05	299.2	307.16	0.24	0.27	0.21
Puerto Rico	212.24	207.92	216.83	270.7	253.82	288.93	0.37	0.19	0.54
Qatar	198.16	221.15	186.81	129.07	133.28	127.49	-0.10	-0.18	-0.05
Romania	404.25	273.58	538.68	345.14	206.26	492.96	-0.11	-0.20	-0.06
Russia	90.24	87.35	93.51	238.27	201.12	281.91	1.46	1.24	1.69
Rwanda	770.43	836.53	701.99	392.95	399.73	385.57	0.20	0.13	0.26
Saint Lucia	240.79	232.66	249.17	423.22	402.55	444.68	1.08	1.04	1.11
Saint Vincent and the Grenadines	274.04	287.68	260.26	531.76	478.99	583.56	0.97	0.80	1.15
Samoa	255.13	195.45	310.14	301.82	324.84	280.2	0.38	0.70	0.18
Sao Tome and Principe	816.47	1,056.75	571.74	595.4	768.11	421.39	0.31	0.29	0.33
Saudi Arabia	209.9	216.62	204.61	201.58	200.33	202.54	0.68	0.74	0.64
Senegal	425.35	518.19	331.62	301.62	363.2	237.77	0.39	0.37	0.39
Serbia	345.64	310.18	382.05	466.09	405.66	529.47	0.32	0.26	0.36
Seychelles	446.72	333.64	560.28	352.35	272.71	429.88	-0.13	-0.13	-0.12
Sierra Leone	625.54	648.45	602.17	439.89	472.85	406.25	0.26	0.25	0.26
Singapore	62.98	69.77	56.36	77.49	86.04	68.64	0.42	0.37	0.44
Slovakia	638.55	553.2	727.94	454.27	422.47	487.82	-0.15	-0.14	-0.16
Slovenia	344.76	337.83	352.1	392.45	395.91	388.92	0.33	0.31	0.35
Solomon Islands	354.94	315.97	391.83	413.82	410.7	416.83	0.52	0.67	0.40
Somalia	932.15	998.59	864.53	624.07	669.53	578.18	-0.01	-0.01	-0.01
South Africa	198.62	237.66	158.66	238.43	284.7	190.52	0.14	0.14	0.13
South Korea	106.57	109.37	103.78	120.49	126.81	114.09	0.30	0.31	0.29
South Sudan	795.84	857.68	733.42	591.69	633.87	549.64	0.19	0.18	0.20
Spain	368.55	338.03	400.35	330.89	321.95	340.07	0.07	0.07	0.07
Sri Lanka	734.28	512.12	952.16	279.05	208.29	355.1	-0.47	-0.46	-0.46
Sudan	607.72	617.25	598.26	472.62	457.92	487.22	0.51	0.47	0.56
Suriname	185.19	171.99	197.97	295.92	268.48	323.25	0.83	0.86	0.80
Swaziland	159.45	186.54	129.16	215.83	249.8	181.14	-0.01	-0.06	0.05
Sweden	397.29	384.3	410.59	301.2	291.09	311.34	-0.09	-0.13	-0.05
Switzerland	594.57	558.9	631.92	348.17	337.59	358.98	-0.27	-0.29	-0.26
Syria	194.04	171.32	216.03	138.88	124.31	153.09	-0.41	-0.22	-0.51
Taiwan	224.17	210.06	237.26	229.27	258.42	200.03	0.01	0.18	-0.16
Tajikistan	28.13	30.35	25.89	30.27	26.12	34.32	1.10	0.73	1.54
Tanzania	528.27	545.99	510.23	408.84	397.05	420.77	0.48	0.40	0.55
Thailand	396.94	304.35	491.23	405.55	359.95	452.47	-0.01	0.20	-0.14
The Bahamas	219.46	213.28	225.73	399.68	402.48	396.76	1.01	1.00	0.99
The Gambia	451.06	505.88	397.03	303.52	296.8	310.37	0.25	0.15	0.38
Timor-Leste	277.86	271.43	284.12	173.9	168.1	179.53	0.62	0.63	0.61
Togo	541.33	623.69	456.84	397.62	391.78	403.59	0.11	0.01	0.26
Tonga	46.19	36.54	55.6	59	51.44	66.51	0.41	0.63	0.26
Trinidad and Tobago	87.43	93.69	81.13	266.41	266.36	266.46	1.90	1.85	1.99
Tunisia	456.28	450.88	461.61	380.44	367.64	393.53	0.12	0.14	0.10
Turkey	465.3	438.3	492.96	343.09	348.22	337.77	0.40	0.49	0.31
Turkmenistan	54.24	57.38	51	55.01	52.36	57.76	0.70	0.62	0.81
Uganda	576.9	626.3	526.85	454.91	414.09	495.76	0.60	0.38	0.85
Ukraine	56.01	56.9	54.98	70.1	64	77.32	0.16	0.08	0.25
United Arab Emirates	51.9	46.48	54.71	61.7	48.42	66.54	0.49	0.49	0.48

United Kingdom	315.1	322.56	307.21	326.16	323.99	328.37	0.28	0.20	0.36
United States	309.54	302.07	317.4	307.41	302.69	312.29	0.07	0.03	0.10
Uruguay	227.48	210.68	245.32	390.82	401.16	379.75	0.93	1.05	0.79
Uzbekistan	20.63	20.49	20.77	37.37	30.4	44.58	1.43	1.07	1.78
Vanuatu	305.22	235.56	371.09	354.18	306.29	400.88	0.32	0.45	0.25
Venezuela	74.81	77.86	71.8	138.59	137.71	139.48	0.79	0.88	0.75
Vietnam	176.59	155.29	198.54	192.69	180.24	205.41	0.58	0.78	0.43
Virgin Islands, U.S.	240.39	212.71	269.7	570.81	484.56	665.61	0.78	0.78	0.78
Yemen	630.75	699.8	560.43	417.93	459.78	376.95	0.40	0.41	0.40
Zambia	538.28	627.59	447.46	532.13	453.76	610.73	0.66	0.27	1.18
Zimbabwe	246.32	302.4	189.77	290.64	310.88	269.8	0.04	-0.10	0.25
**Peripheral artery disease**									
Afghanistan	2.04	2.02	2.06	2.45	2.5	2.4	0.98	1.14	0.85
Albania	5.42	5.71	5.15	12.63	12.63	12.64	1.62	1.55	1.68
Algeria	3.43	3.5	3.36	6.56	6.46	6.66	1.73	1.61	1.85
American Samoa	4.77	3.99	5.5	12.26	4.94	19.55	2.13	0.31	3.79
Andorra	40.99	31.85	50.64	57.8	45.89	70.21	0.42	0.48	0.37
Angola	3.21	3.53	2.89	3.88	4.17	3.58	1.49	1.35	1.65
Antigua and Barbuda	12.48	11.22	13.84	18.24	15.27	21.49	0.62	0.49	0.74
Argentina	12.58	11.26	13.96	15.06	15.18	14.93	0.42	0.54	0.31
Armenia	6.11	7.01	5.16	12.96	14.27	11.45	1.36	1.29	1.43
Australia	38.87	34.34	43.43	47.21	42.82	51.61	0.41	0.38	0.44
Austria	37.84	34.75	41.2	77.77	71.1	84.78	1.45	1.40	1.49
Azerbaijan	4.6	5.49	3.66	7.32	7.95	6.68	1.39	1.20	1.73
Bahrain	5.96	3.64	7.67	12.41	9.73	14.06	2.02	2.74	1.72
Bangladesh	2.81	2.67	2.94	4.53	4.42	4.63	2.74	3.15	2.41
Barbados	43.14	53.88	31.56	78.11	99.16	55.22	0.72	0.69	0.71
Belarus	11.91	14.29	9.22	32.74	29.56	36.43	1.38	0.94	2.19
Belgium	17	17.62	16.36	34.89	32.02	37.87	1.34	1.04	1.69
Belize	3.55	2.84	4.25	4.43	3.59	5.28	0.49	0.59	0.42
Benin	3.63	4.03	3.21	3.51	3.9	3.11	0.88	0.90	0.89
Bermuda	20.08	17.44	22.75	31.19	32.65	29.72	0.97	1.24	0.73
Bhutan	3.16	3	3.3	6.07	5.58	6.48	3.31	3.52	3.10
Bolivia	2.54	2.58	2.5	4.07	4.11	4.02	2.46	2.45	2.47
Bosnia and Herzegovina	8.28	8.85	7.72	19.78	20.35	19.21	1.42	1.25	1.60
Botswana	7.25	6.78	7.73	16.55	13.31	19.8	0.77	0.56	0.94
Brazil	8.37	7.31	9.44	23.01	20.97	25.12	2.47	2.60	2.36
Brunei	3.62	2.85	4.32	6.88	5.62	8.07	0.94	1.00	0.91
Bulgaria	13.15	13.85	12.43	25.87	26.74	24.96	0.92	0.85	0.99
Burkina Faso	3.57	3.87	3.25	3.17	3.8	2.53	0.66	0.82	0.48
Burundi	4.79	4.63	4.97	3.43	3.4	3.47	0.62	0.69	0.56
Cambodia	3.72	4.02	3.39	5.81	6.51	5.07	2.81	3.06	2.57
Cameroon	3.93	4.08	3.79	3.94	4.24	3.63	0.35	0.42	0.28
Canada	34.53	29.3	39.85	43.08	41.28	44.9	0.30	0.40	0.23
Cape Verde	5.9	6.79	4.92	8.15	10.67	5.57	1.14	1.46	0.75
Central African Republic	4.79	5.09	4.47	4.99	5.35	4.62	0.29	0.29	0.30
Chad	3.35	3.54	3.15	2.89	3.14	2.64	0.27	0.31	0.24
Chile	11.4	10.43	12.39	32.77	30.42	35.18	2.31	2.15	2.45
China	5.65	6.06	5.27	11.71	11.93	11.51	2.07	2.20	2.02
Colombia	3.46	3.49	3.42	6.37	6.71	6.01	1.46	1.38	1.48
Comoros	4.82	5.21	4.43	4.25	4.55	3.97	0.91	0.89	0.94
Congo	5.89	6.19	5.59	6.16	7.1	5.22	0.49	0.46	0.50
Costa Rica	3.84	3.47	4.22	8.49	7.69	9.28	1.31	1.30	1.32
Cote d'Ivoire	3.42	3.21	3.61	3.91	3.62	4.18	0.63	0.64	0.63
Croatia	12.48	14.32	10.52	77.3	71.97	83.02	5.71	4.21	7.85
Cuba	18.79	17.78	19.78	65.43	58.52	72.27	2.34	2.14	2.51
Cyprus	20.35	19.53	21.19	21.69	20.67	22.78	0.24	0.24	0.24
Czech Republic	15.3	16.35	14.18	46.86	41.92	51.95	2.81	2.09	3.70
Democratic Republic of the Congo	3.44	3.96	2.91	3.3	3.68	2.93	0.56	0.50	0.65
Denmark	63.69	62.55	64.86	51.56	53.65	49.44	0.00	0.04	-0.04
Djibouti	4.13	4.4	3.86	7.19	7.06	7.33	1.44	1.30	1.63

Dominica	8.33	6.27	10.4	21.99	16.99	27.07	1.39	1.69	1.18
Dominican Republic	2.57	2.5	2.65	5.16	5.1	5.22	1.70	1.80	1.61
Ecuador	2.28	2.28	2.28	4.23	4.28	4.18	1.59	1.67	1.52
Egypt	4.69	5.01	4.37	5.93	6.29	5.58	1.19	1.26	1.13
El Salvador	2.13	2.16	2.1	4.26	4.25	4.28	1.97	1.91	2.00
Equatorial Guinea	5.7	6.18	5.25	8.08	7.2	8.91	1.71	1.23	2.26
Eritrea	2.32	2.71	1.92	3.38	3.95	2.82	1.61	1.56	1.70
Estonia	11.62	14.5	8.34	34.81	36.55	32.78	2.83	2.01	4.32
Ethiopia	3.77	3.62	3.92	4.38	4.46	4.31	2.67	2.85	2.52
Federated States of Micronesia	4.86	4.6	5.11	8.23	7.49	8.94	1.28	1.19	1.36
Fiji	3.6	3.58	3.62	8.47	9.65	7.32	1.05	1.26	0.82
Finland	38.08	38.81	37.3	72.71	75.01	70.34	1.19	1.15	1.22
France	15.93	17.33	14.46	29.13	27.85	30.49	1.03	0.73	1.41
Gabon	11.26	10.62	11.94	10.89	11.68	10.11	0.45	0.61	0.31
Georgia	9.99	11.67	8.15	21.49	20.77	22.28	1.17	0.92	1.63
Germany	25.46	26.34	24.52	105.5	89.2	122.35	3.67	2.82	4.63
Ghana	3.2	3.47	2.94	4.02	4.2	3.83	1.15	1.18	1.15
Greece	17.11	17.22	17	20.48	21.22	19.7	0.21	0.24	0.18
Greenland	18.8	15.29	21.84	40.38	31.27	48.29	2.00	1.92	2.04
Grenada	14.66	12.1	17.29	22.89	21.7	24.07	0.71	1.01	0.50
Guam	5.06	5.16	4.98	34.7	19.68	49.31	4.11	1.82	6.33
Guatemala	1.73	1.61	1.85	2.59	2.62	2.56	1.75	2.09	1.47
Guinea	3.82	4.02	3.62	3.68	3.89	3.46	0.82	0.88	0.78
Guinea-Bissau	4.22	4.47	3.97	3.95	4.22	3.67	0.45	0.45	0.45
Guyana	4.15	2.02	6.35	7.64	6.61	8.66	1.09	2.81	0.53
Haiti	3.39	3.1	3.68	4.46	4.41	4.51	1.54	1.70	1.40
Honduras	1.82	1.83	1.8	3.25	3.15	3.34	1.81	1.63	2.00
Hungary	23.39	19.92	27.14	71.13	58.78	84.73	2.90	2.53	3.25
Iceland	14.15	16.24	12.09	19.04	20.48	17.56	0.56	0.49	0.66
India	3.79	3.65	3.93	6.64	6.18	7.06	2.00	2.17	1.85
Indonesia	5.18	5.39	4.96	7.91	8.27	7.55	1.36	1.46	1.29
Iran	3.24	2.93	3.54	5.53	4.94	6.11	2.26	2.42	2.14
Iraq	4.02	3.84	4.2	3.67	3.63	3.71	0.14	0.28	0.03
Ireland	49.15	40.88	57.49	33.35	30.05	36.74	-0.17	-0.09	-0.22
Israel	21.26	17.29	25.25	40.84	35.39	46.38	1.32	1.45	1.24
Italy	30.95	24.22	38.1	46.17	44.35	48.09	0.64	0.90	0.46
Jamaica	17.07	14.79	19.41	35.67	34.37	36.99	0.99	1.30	0.75
Japan	12.74	13.77	11.67	29.17	30.67	27.6	1.03	1.01	1.07
Jordan	3.19	3.12	3.26	3.69	3.65	3.73	0.74	0.82	0.67
Kazakhstan	9.66	10.31	8.97	13.23	12.38	14.14	0.49	0.37	0.65
Kenya	3.4	3.47	3.33	3.93	4.11	3.74	0.60	0.68	0.53
Kiribati	4.48	4.93	4.01	7.07	6.75	7.4	1.24	1.11	1.45
Kuwait	1.76	1.4	2.07	2.4	2.18	2.57	0.69	0.82	0.61
Kyrgyzstan	10.05	9.75	10.35	10.12	7.93	12.37	0.42	0.21	0.62
Laos	4.96	5.31	4.61	5.98	6.28	5.66	2.33	2.41	2.28
Latvia	12.19	14.83	9.16	43.19	42.51	43.97	3.12	2.24	4.73
Lebanon	6.13	5.7	6.58	8.74	8.44	9.03	1.48	1.29	1.66
Lesotho	10.51	9.52	11.56	17.4	16.26	18.56	0.04	0.04	0.04
Liberia	3.41	3.43	3.38	3.61	3.84	3.38	1.75	1.75	1.74
Libya	3.23	3.19	3.26	5.36	5.31	5.41	0.76	0.95	0.62
Lithuania	12.32	14.42	9.98	61.57	45.78	79.93	4.04	2.23	6.95
Luxembourg	22.43	21.24	23.67	25.46	24.31	26.61	0.62	0.54	0.70
Macedonia	7.44	7.57	7.3	14.29	14.81	13.75	1.07	1.11	1.03
Madagascar	4.91	4.57	5.25	4.89	4.94	4.83	0.88	1.09	0.70
Malawi	3.5	3.77	3.22	4.37	4.68	4.06	1.72	1.78	1.68
Malaysia	5.02	5	5.03	8.79	8.25	9.35	0.86	0.88	0.86
Maldives	5.1	4.74	5.45	7.8	7.81	7.78	3.08	3.62	2.66
Mali	4.03	4.17	3.88	3.1	3.5	2.69	0.37	0.48	0.25
Malta	21.53	18.79	24.36	45.84	43.04	48.67	1.14	1.30	1.01
Marshall Islands	3.73	3.38	4.06	10.97	8.76	13.11	2.54	2.08	2.93
Mauritania	3.7	4.08	3.31	4.02	4.59	3.47	1.01	1.03	0.99

Mauritius	7.14	7.67	6.61	16.75	17.19	16.3	1.30	1.15	1.45
Mexico	5.47	5.6	5.35	7.55	7.94	7.16	0.82	0.82	0.80
Moldova	8.09	9.18	6.9	27.3	22.04	32.99	3.11	1.99	4.71
Mongolia	5.06	4.57	5.57	6.57	5.4	7.76	0.90	1.01	0.82
Montenegro	10.89	12.18	9.58	18.58	19.52	17.62	0.65	0.60	0.73
Morocco	4.27	3.98	4.55	7.26	6.78	7.75	1.56	1.56	1.57
Mozambique	4.13	4.43	3.8	5.02	5.19	4.84	1.25	1.15	1.38
Myanmar	5.88	6.38	5.36	8.88	9.63	8.08	1.66	1.86	1.50
Namibia	9.28	8.2	10.41	11.97	10.44	13.6	0.51	0.53	0.49
Nepal	3.45	3.78	3.11	5.91	5.66	6.18	2.92	2.67	3.24
Netherlands	18.52	17.99	19.06	58.4	51.72	65.18	2.44	2.03	2.84
New Zealand	35.86	31.02	40.84	35.51	35.39	35.64	0.18	0.29	0.10
Nicaragua	1.75	1.82	1.69	3.17	3.27	3.07	2.22	2.24	2.21
Niger	2.72	2.81	2.62	3.07	3.09	3.05	1.64	1.58	1.73
Nigeria	3.38	3.57	3.19	3.49	3.69	3.3	0.88	0.92	0.85
North Korea	4.76	6.36	3.06	9.19	11.73	6.53	1.05	1.04	1.19
Northern Mariana Islands	3.47	3.31	3.61	11.19	10.23	12.15	3.06	3.21	2.94
Norway	16.4	16.21	16.59	32.19	30.85	33.51	1.57	1.32	1.81
Oman	2.23	2.55	1.97	3.17	3.36	3.07	1.42	1.37	1.59
Pakistan	4.14	3.57	4.66	5.79	5.23	6.32	1.11	1.32	0.95
Palestine	2.22	2.34	2.11	3.14	3.2	3.09	1.15	1.18	1.15
Panama	9.51	7.47	11.5	12.84	11.79	13.88	0.43	0.69	0.26
Papua New Guinea	2.81	2.8	2.83	4.66	4.29	5.01	1.27	1.07	1.47
Paraguay	2.9	3.07	2.73	7.09	6.48	7.69	1.62	1.39	1.88
Peru	2.33	2.35	2.31	4.13	4.23	4.03	2.65	2.62	2.67
Philippines	4.41	4.62	4.2	7.74	7.55	7.92	1.42	1.26	1.60
Poland	59.56	37.6	82.65	74.07	57.44	91.96	0.44	0.74	0.30
Portugal	30.08	26.43	34	62.85	55.62	70.57	1.52	1.46	1.57
Puerto Rico	16.34	15.82	16.89	36.96	37.99	35.86	1.43	1.35	1.45
Qatar	2.23	2.05	2.32	2.14	1.9	2.23	0.32	0.26	0.34
Romania	30.42	19.9	41.25	49.77	34.63	65.89	0.71	0.85	0.65
Russia	10.22	13.22	6.81	79.57	64.68	97.05	6.27	3.78	11.76
Rwanda	3.35	3.48	3.22	4.02	4.28	3.74	1.83	1.94	1.70
Saint Lucia	14.24	14.42	14.06	28.36	33.12	23.41	1.36	1.71	0.97
Saint Vincent and the Grenadines	8.5	14.09	2.85	13.3	11.93	14.64	0.58	-0.09	3.94
Samoa	5.42	5.33	5.51	11.69	10.88	12.46	1.51	1.08	1.93
Sao Tome and Principe	5.78	6.03	5.51	4.59	5.27	3.9	0.43	0.56	0.30
Saudi Arabia	2.69	2.96	2.47	3.54	3.63	3.47	1.30	1.31	1.31
Senegal	3.37	3.53	3.22	3.56	3.98	3.13	1.10	1.26	0.95
Serbia	12.41	12.28	12.53	23.76	24.18	23.31	0.87	0.90	0.83
Seychelles	10.34	11.65	9.03	13.4	14.86	11.97	0.44	0.36	0.52
Sierra Leone	3.68	3.51	3.85	3.43	3.5	3.35	0.67	0.72	0.63
Singapore	7.6	8.65	6.57	26.11	28	24.16	2.98	2.62	3.38
Slovakia	11.6	11.97	11.21	27.66	26.16	29.24	1.86	1.47	2.28
Slovenia	12.08	13.69	10.38	21.46	23.51	19.38	1.08	0.93	1.27
Solomon Islands	3.4	2.91	3.86	5.54	4.39	6.65	1.13	0.95	1.27
Somalia	4.69	5	4.37	3.96	4.2	3.72	0.27	0.26	0.28
South Africa	21.58	18.52	24.72	39.93	31.44	48.71	0.76	0.62	0.86
South Korea	7.18	5.88	8.48	14.13	14.38	13.88	1.26	1.75	0.91
South Sudan	4.22	4.37	4.07	4.95	5.05	4.86	0.93	0.91	0.96
Spain	21.71	20.34	23.14	40.9	38.12	43.76	1.25	1.10	1.39
Sri Lanka	7.74	7.22	8.25	13.79	13.76	13.83	1.52	1.56	1.42
Sudan	2.67	2.64	2.7	3.25	3.23	3.28	1.37	1.43	1.33
Suriname	3.04	2.44	3.62	4.76	4.17	5.36	0.79	1.04	0.63
Swaziland	8.93	6.8	11.31	16.52	13.71	19.4	0.36	0.43	0.29
Sweden	35.36	35.93	34.77	41.7	42.41	40.99	0.42	0.35	0.48
Switzerland	39.16	34.14	44.41	34.48	30.93	38.11	0.09	0.06	0.13
Syria	3.3	3.1	3.5	4.81	4.76	4.86	0.21	0.65	-0.03
Taiwan	6.41	6.35	6.46	13.07	14.01	12.12	1.02	1.12	0.89
Tajikistan	4.08	4.51	3.63	3.81	3.59	4.03	0.83	0.59	1.13
Tanzania	4.23	4.29	4.17	5.04	4.89	5.2	1.29	1.23	1.37

Thailand	6.42	6.72	6.12	15.3	15.96	14.62	1.32	1.42	1.24
The Bahamas	11.68	9.15	14.24	25.73	26.47	24.97	1.43	2.06	0.99
The Gambia	3.06	2.83	3.28	2.96	2.87	3.04	0.79	0.98	0.63
Timor-Leste	2.54	2.67	2.41	6.23	6.55	5.91	5.39	5.51	5.29
Togo	3.22	3.45	3	3.24	3.49	2.98	0.53	0.64	0.44
Tonga	6.08	6.2	5.96	11.2	10.97	11.43	1.03	1.05	1.02
Trinidad and Tobago	10.52	8.99	12.07	20.44	21.59	19.26	0.85	1.41	0.45
Tunisia	5.13	4.61	5.65	8.98	8.63	9.34	1.35	1.63	1.13
Turkey	13.75	10.07	17.53	17.04	13.8	20.4	1.35	1.57	1.23
Turkmenistan	4.53	5.09	3.96	7.61	6.72	8.53	1.81	1.34	2.44
Uganda	3.65	3.57	3.72	3.7	3.56	3.84	1.07	1.10	1.06
Ukraine	13.75	16.55	10.48	29.56	28.81	30.45	0.99	0.68	1.60
United Arab Emirates	1.62	1.61	1.62	2.18	1.47	2.44	0.69	0.30	0.84
United Kingdom	64.98	61.29	68.89	55.83	58.91	52.67	0.06	0.15	-0.03
United States	31.48	30.25	32.78	46.07	44.38	47.82	0.57	0.51	0.63
Uruguay	14.84	14.07	15.66	16.27	18.05	14.36	0.23	0.38	0.06
Uzbekistan	4.31	5.02	3.59	5.65	6.05	5.23	0.75	0.68	0.89
Vanuatu	4.7	3.88	5.47	6.53	5.54	7.49	0.59	0.60	0.59
Venezuela	4.09	4.4	3.78	6.39	7.14	5.64	0.51	0.73	0.34
Vietnam	7.3	8.52	6.04	10.65	12.4	8.85	1.12	1.25	1.04
Virgin Islands, U.S.	9.3	5.35	13.47	46.94	60.38	32.17	2.78	7.85	0.72
Yemen	2.35	2.55	2.14	2.78	2.81	2.76	1.52	1.37	1.71
Zambia	3.78	4.11	3.44	4.73	4.81	4.64	1.11	1.08	1.18
Zimbabwe	4.01	4.14	3.87	4.16	4.18	4.14	-0.08	-0.11	-0.05

eFigure 1a.

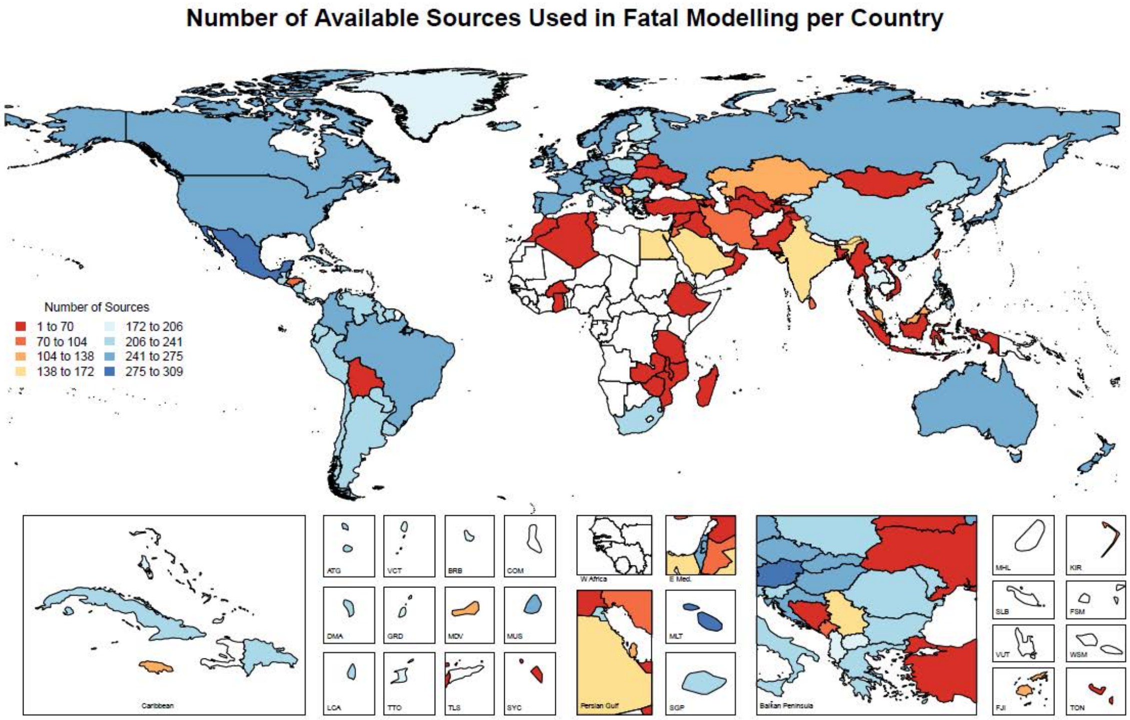

eFigure 1b.

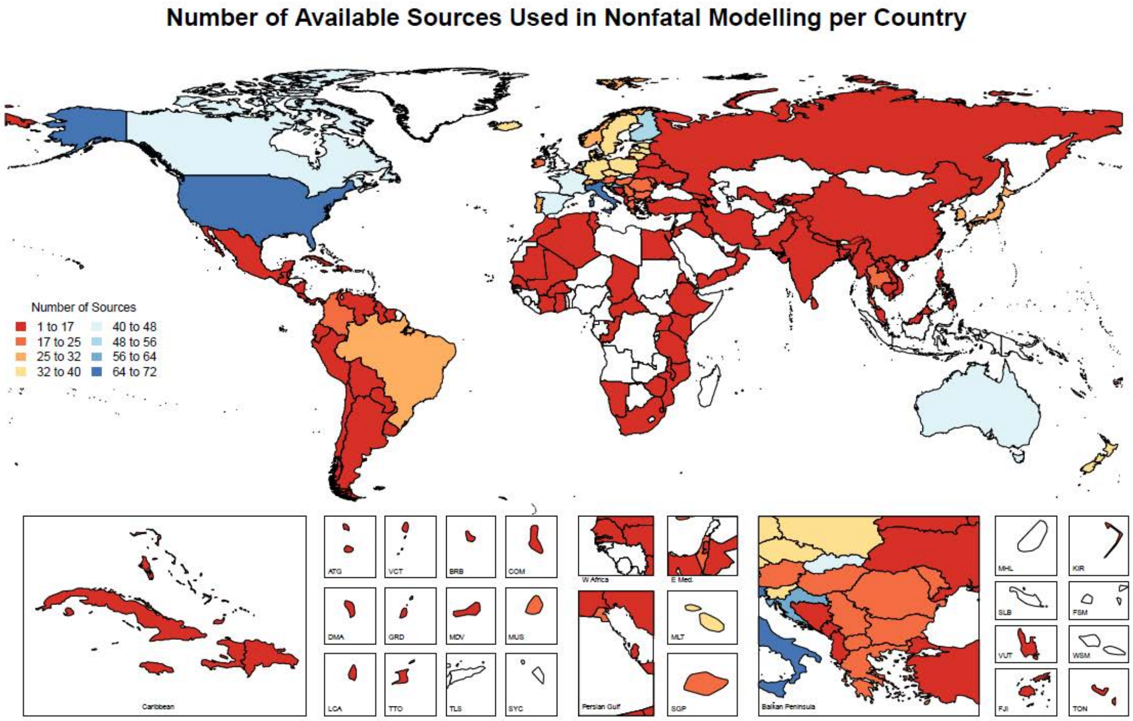

eFigure 2a.

All Cardiovascular Disease: Age−Standardized Median Percent Change in Prevalent Cases per 100,000 Persons, Both Sexes, 1990−2015

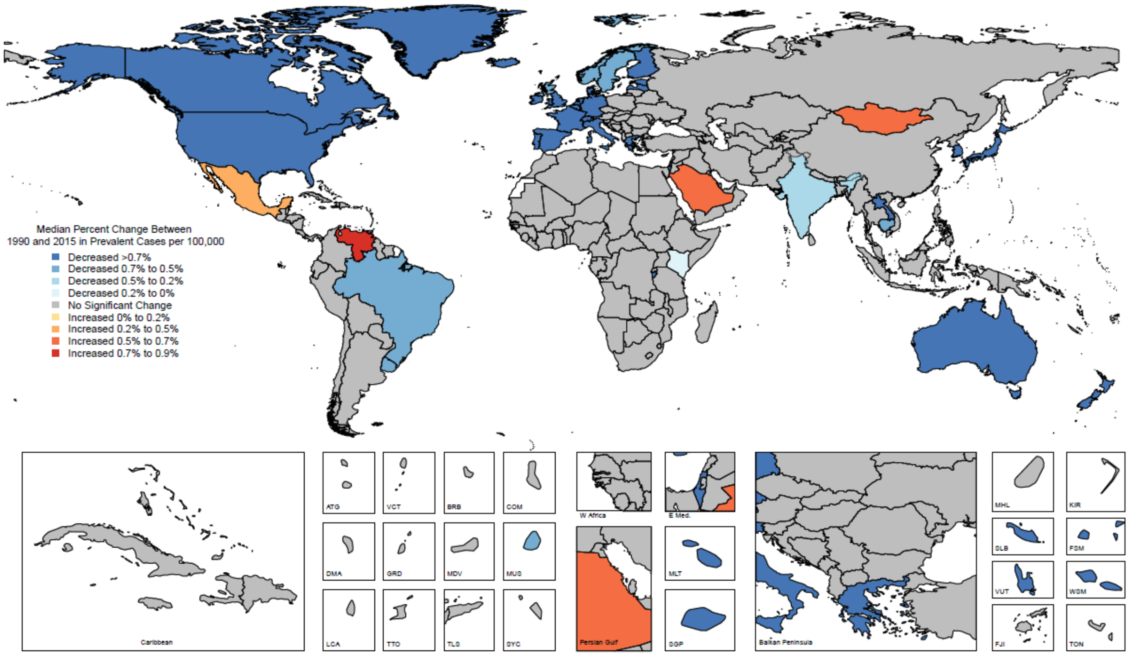

eFigure 2b.

All Cardiovascular Disease: Age−Standardized Median Percent Change in Deaths per 100,000 Persons, Both Sexes, 1990−2015

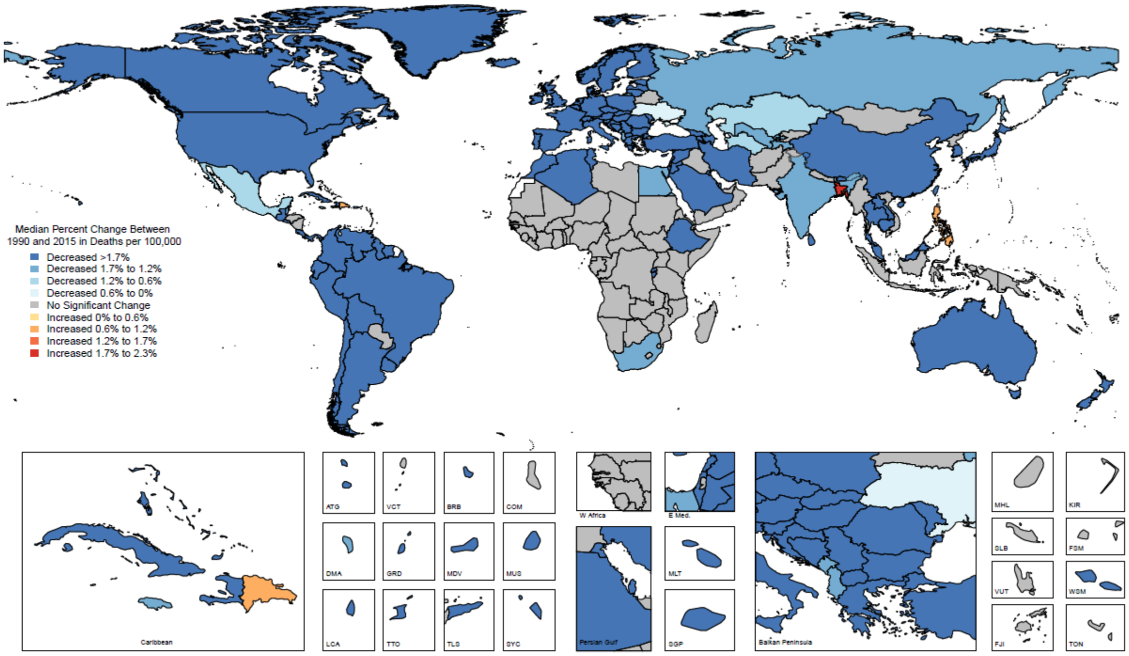

eFigure 3. Rankings of Cardiovascular Disease Subcauses by Age-Standardized DALYs, Both Sexes, 2015

	Global	Central Asia	Eastern Europe	Central Europe	Western Europe	High-income North America	High-income Asia Pacific	Australasia	Southern Latin America	Andean Latin America	Central Latin America	Tropical Latin America	Caribbean	Oceania	East Asia	Southeast Asia	South Asia	North Africa and Middle East	Central Sub-Saharan Africa	Eastern Sub-Saharan Africa	Southern Sub-Saharan Africa	Western Sub-Saharan Africa
Ischemic heart disease	1	1	1	1	1	1	1	1	1	1	1	1	1	1	1	1	1	1	1	1	1	1
Hemorrhagic and other stroke	2	2	3	3	2	3	2	2	2	2	2	2	2	2	2	2	2	2	2	2	2	2
Ischemic stroke	3	3	2	2	3	2	3	3	3	3	3	3	3	3	3	3	3	3	3	4	3	3
Hypertensive heart disease	4	4	5	6	7	5	7	9	5	5	5	6	4	4	4	4	4	5	4	5	4	5
Other cardiovascular and circulatory diseases	5	6	6	5	4	4	4	4	4	4	4	4	5	5	5	5	8	4	5	3	5	4
Rheumatic heart disease	6	5	9	9	10	9	9	10	8	8	10	9	7	6	6	6	5	7	6	6	7	6
Cardiomyopathy and myocarditis	7	7	4	4	6	6	6	5	6	6	6	5	6	7	7	7	7	6	7	7	6	7
Atrial fibrillation and flutter	8	8	7	7	5	7	8	6	9	7	7	8	9	10	8	8	6	9	10	10	11	9
Aortic aneurysm	9	9	8	8	8	8	5	7	7	10	8	7	8	9	9	10	9	10	9	9	8	10
Endocarditis	10	10	11	11	11	11	10	11	10	9	9	10	10	8	11	9	10	8	8	8	10	8
Peripheral artery disease	11	11	10	10	9	10	11	8	11	11	11	11	11	11	10	11	11	11	11	11	9	11

eFigure 4a. Prevalence of CVD level 3 and 4 causes in 2015, by age categories

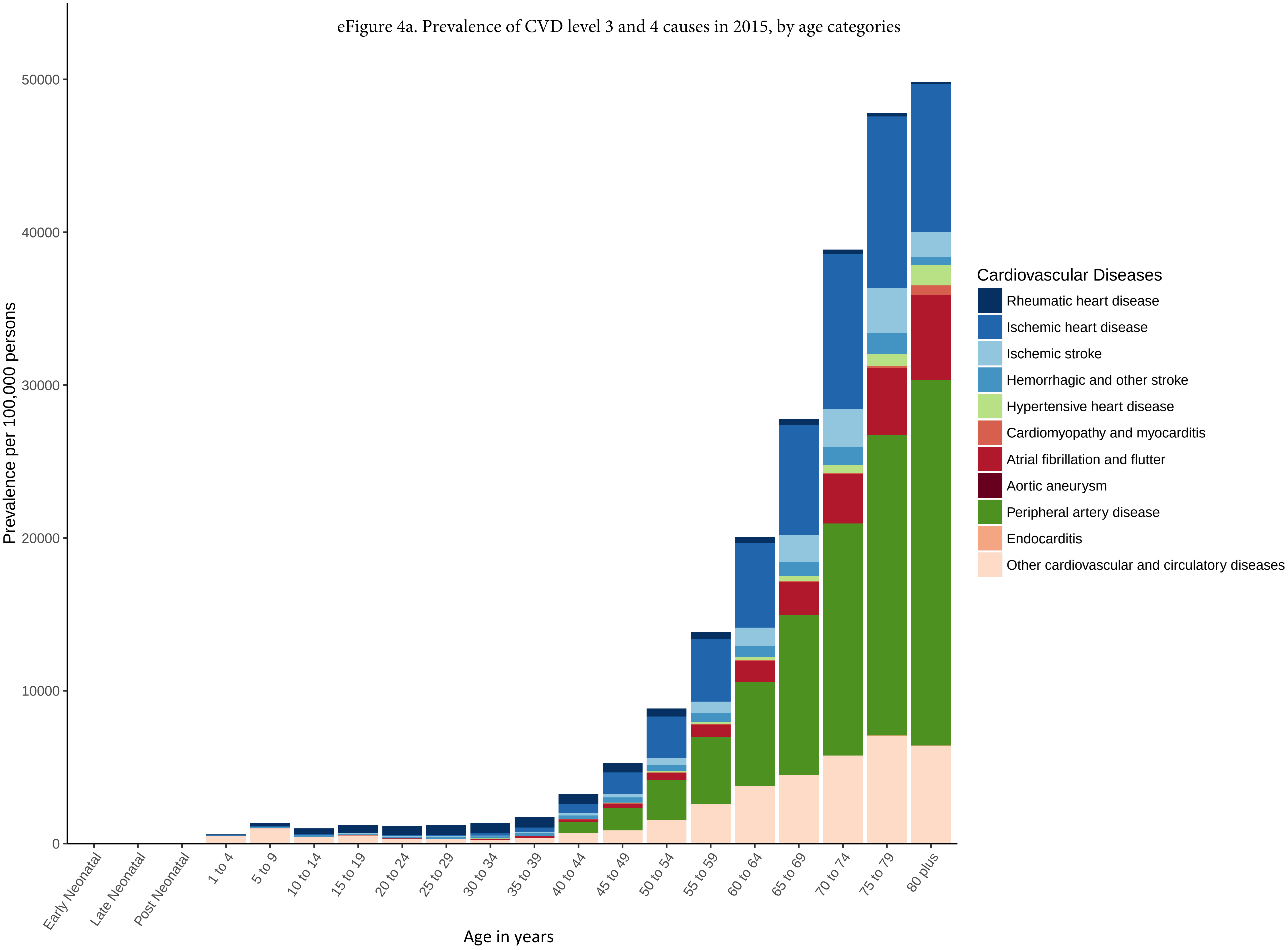

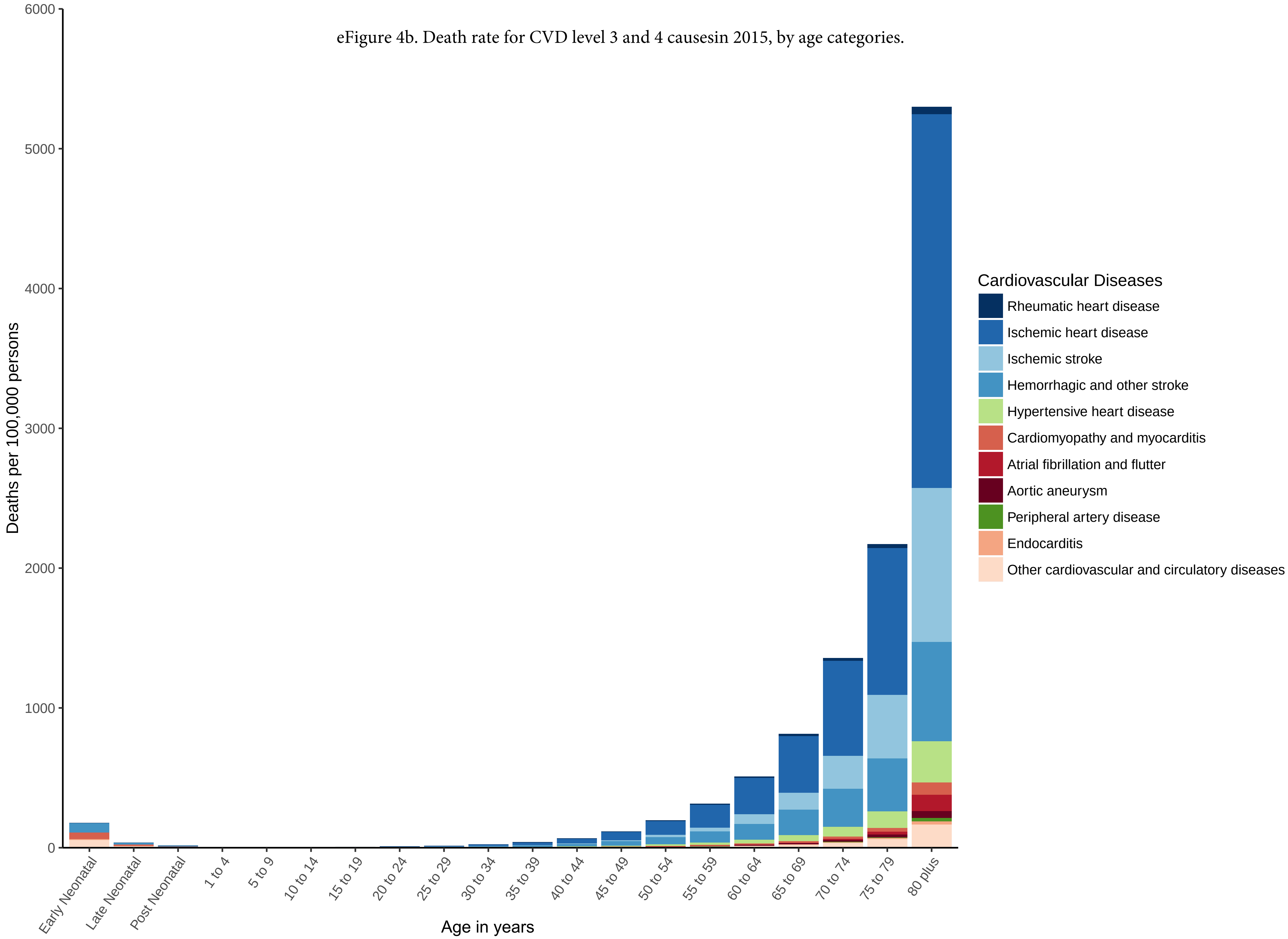
eFigure 4b. Death rate for CVD level 3 and 4 causesin 2015, by age categories.
Deaths per 100,000 persons
0
1000
2000
3000
4000
5000
6000
Early Neonatal
Late Neonatal
Post Neonatal
1 to 4
5 to 9
10 to 14
15 to 19
20 to 24
25 to 29
30 to 34
35 to 39
40 to 44
45 to 49
50 to 54
55 to 59
60 to 64
65 to 69
70 to 74
75 to 79
80 plus
Age in years
Cardiovascular Diseases
Rheumatic heart disease
Ischemic heart disease
Ischemic stroke
Hemorrhagic and other stroke
Hypertensive heart disease
Cardiomyopathy and myocarditis
Atrial fibrillation and flutter
Aortic aneurysm
Peripheral artery disease
Endocarditis
Other cardiovascular and circulatory diseases

eFigure 5a.

Age−Standardized Prevalence of Ischemic Heart Disease per 100,000 Persons in 2015, Both Sexes

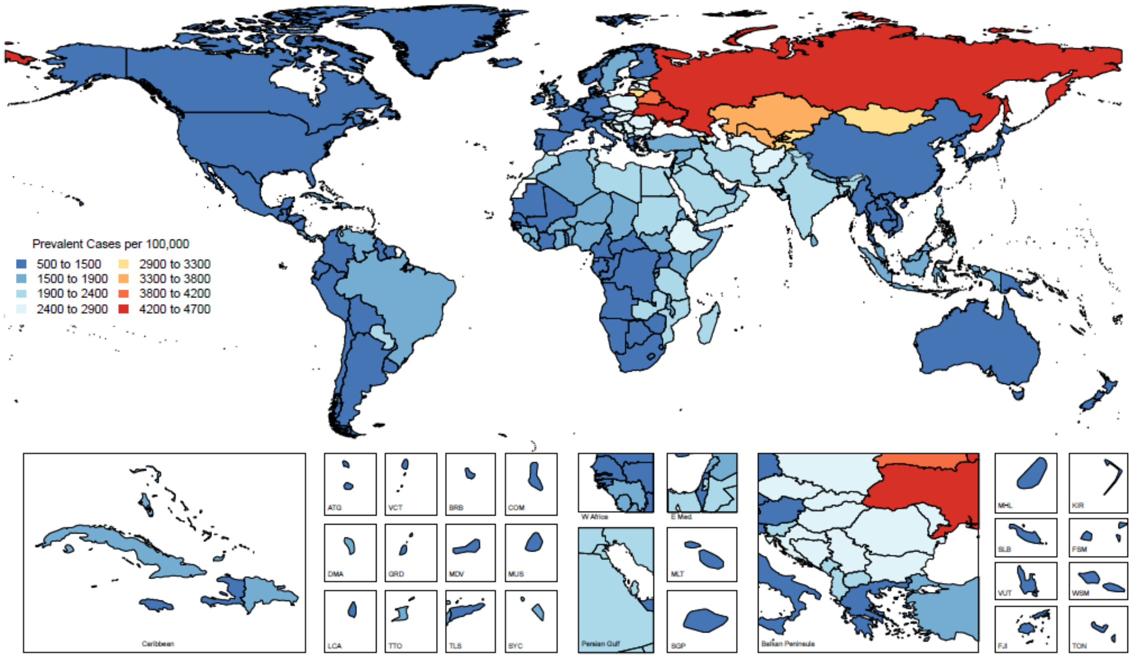

eFigure 5b.

Age−Standardized Prevalence of Hypertensive Heart Disease per 100,000 Persons in 2015, Both Sexes

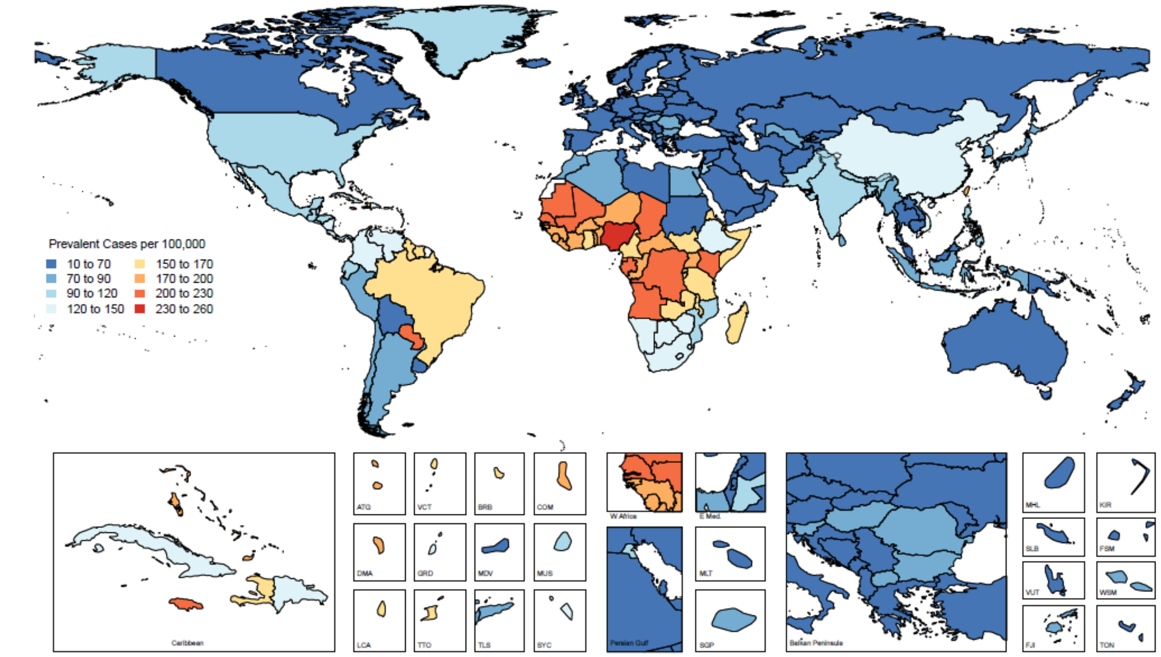

eFigure 5c.

Age−Standardized Prevalence of Ischemic Stroke per 100,000 Persons in 2015, Both Sexes

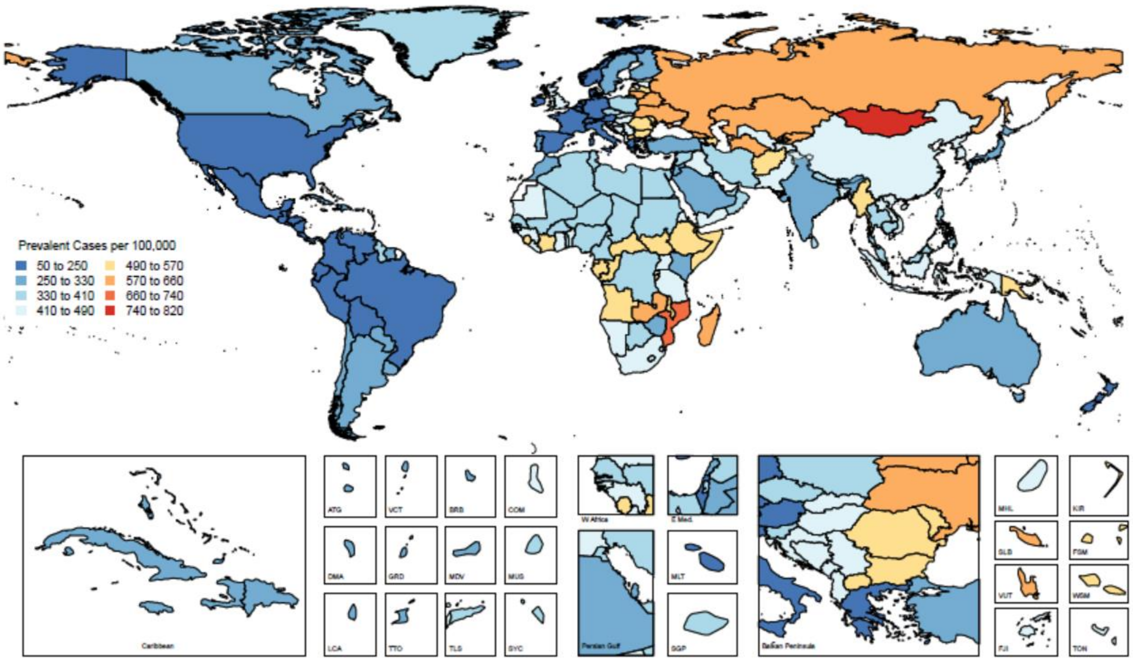

eFigure 5d.

Age−Standardized Prevalence of Hemorrhagic Stroke per 100,000 Persons in 2015, Both Sexes

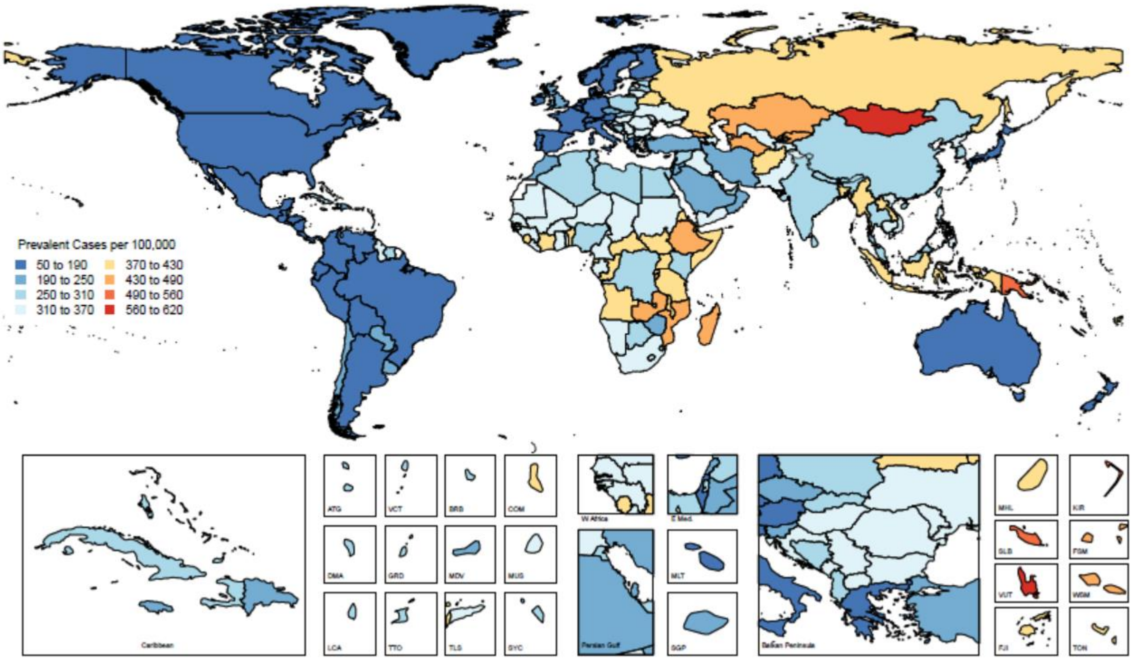

eFigure 6a.

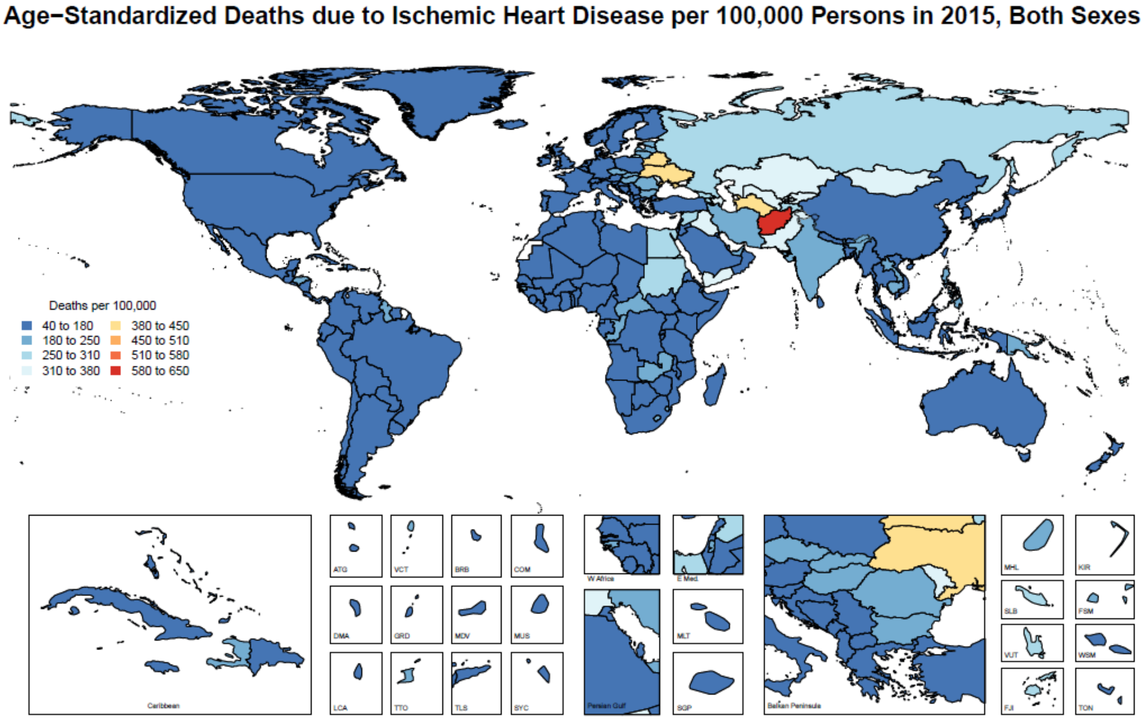

eFigure 6b.

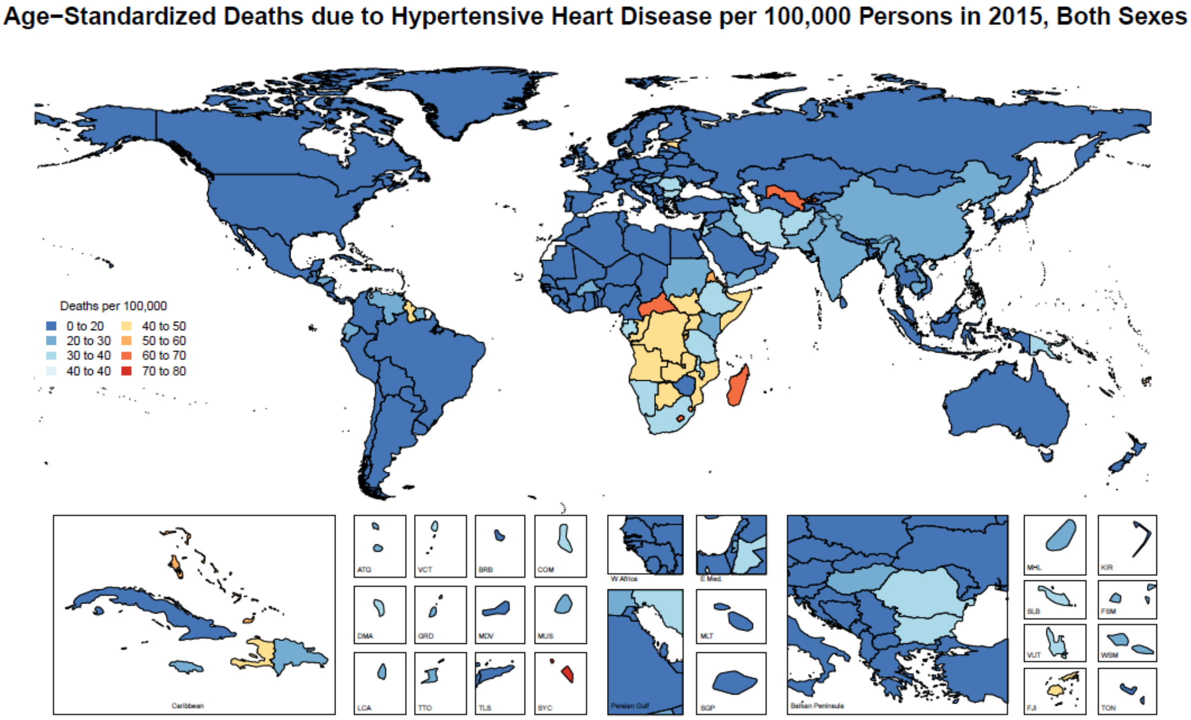

eFigure 6c.

Age−Standardized Deaths due to Ischemic Stroke per 100,000 Persons in 2015, Both Sexes

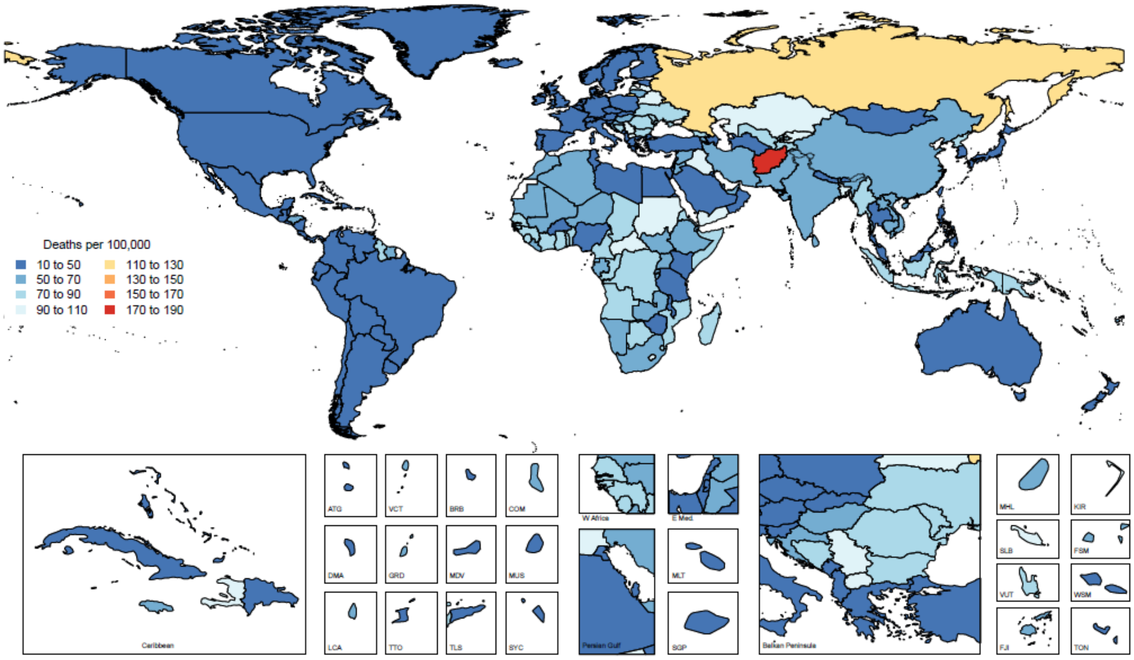

eFigure 6d.

Age−Standardized Deaths due to Hemorrhagic Stroke per 100,000 Persons in 2015, Both Sexes

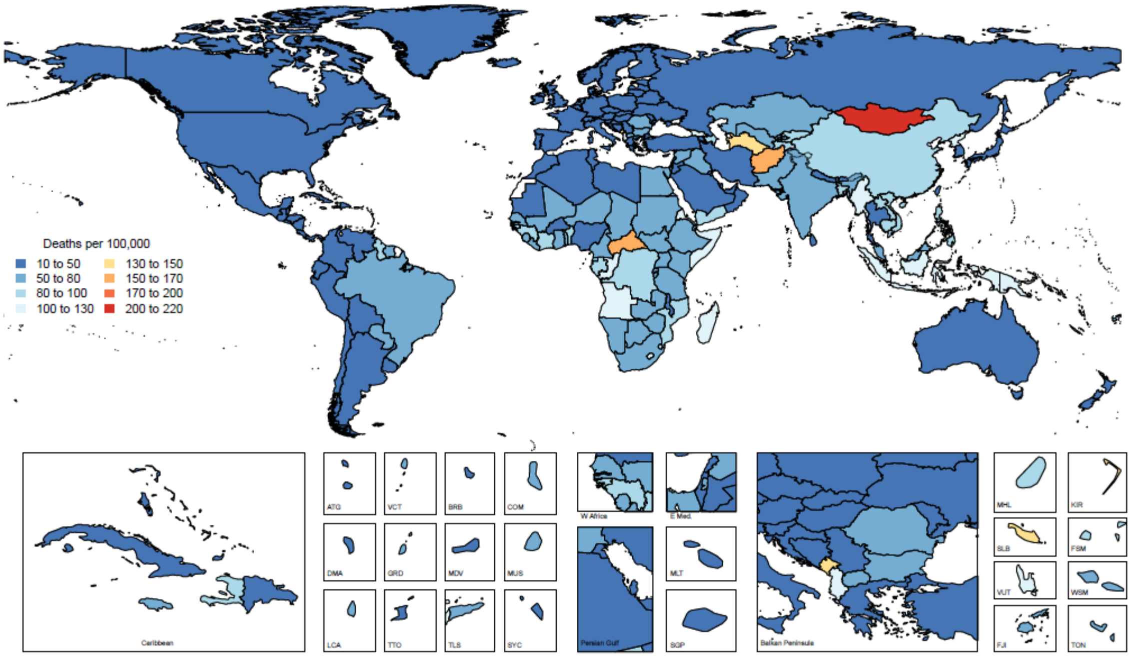

eFigure 7a.

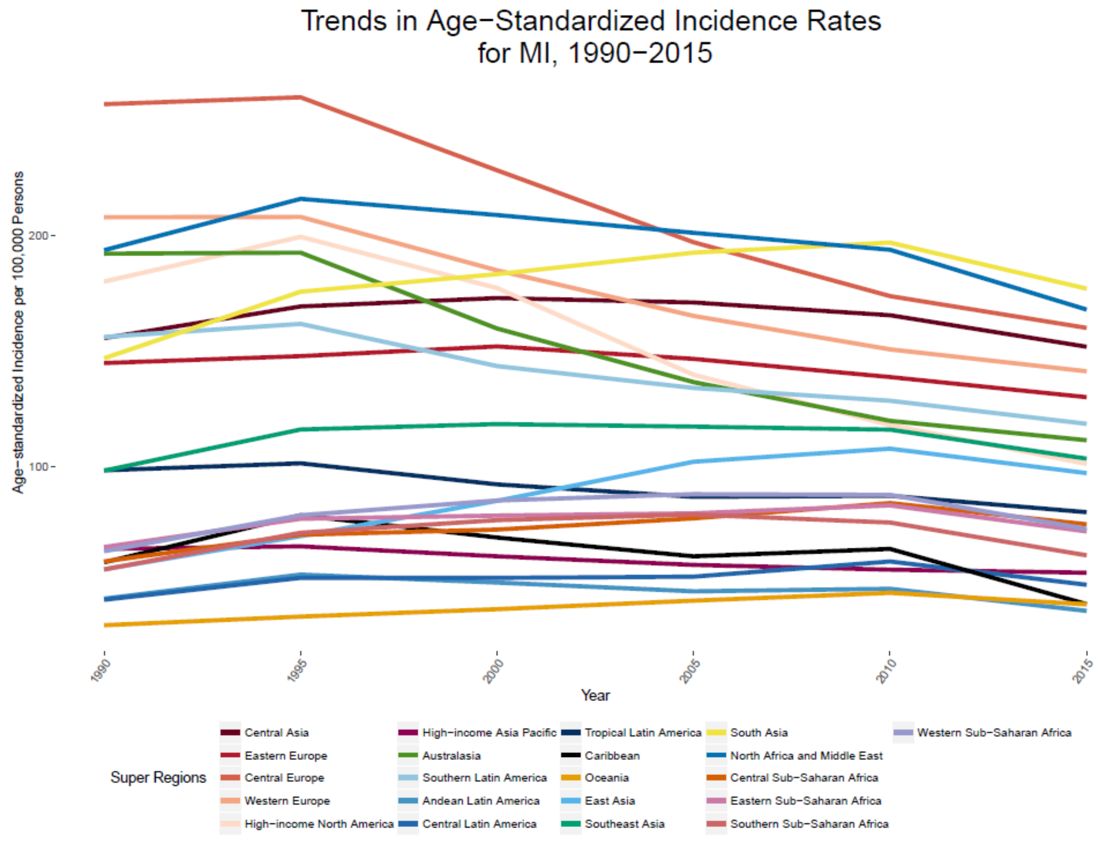

eFigure 7b.

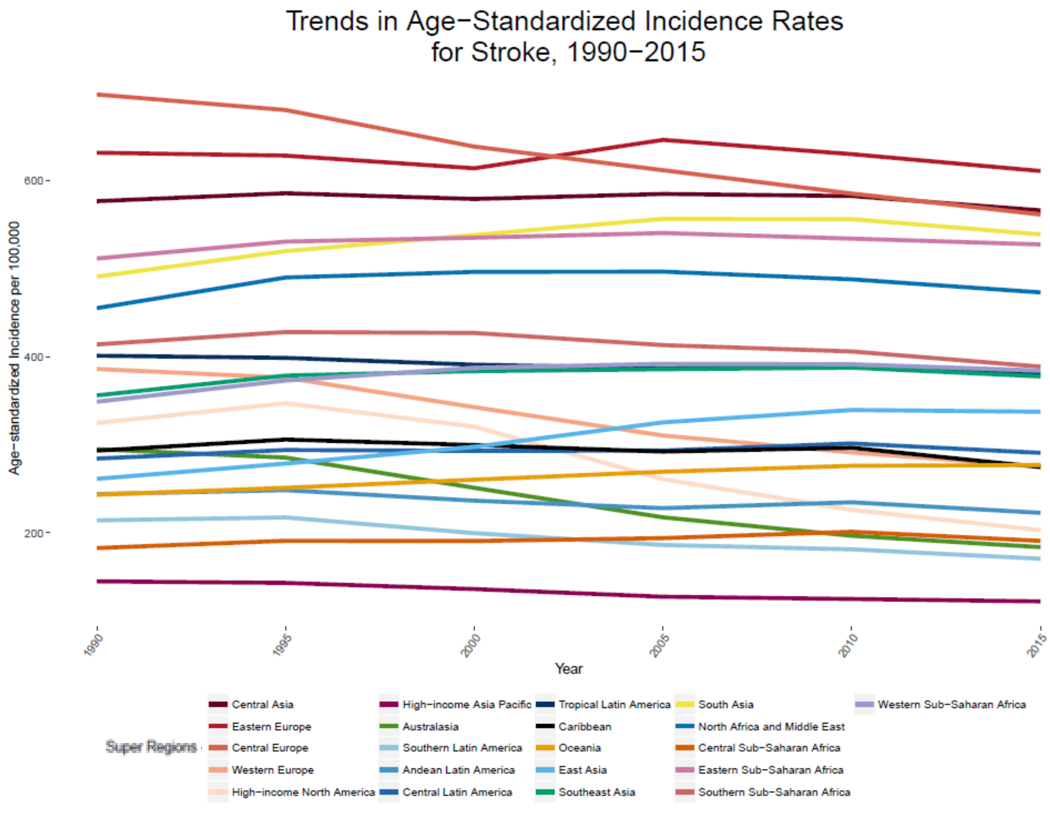

eFigure 8a.

Age−Standardized Prevalence of All Cardiovascular Disease per 100,000 Persons in 2015, Females

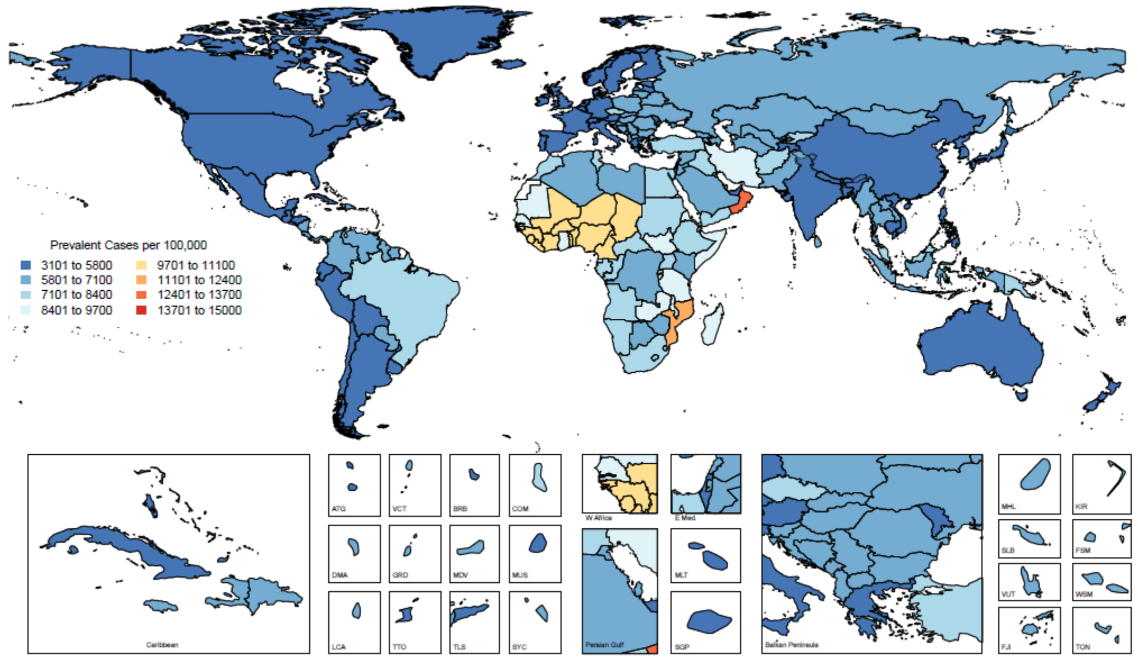

eFigure 8b.

Age−Standardized All Cardiovascular Disease, Deaths per 100,000 Persons in 2015, Females

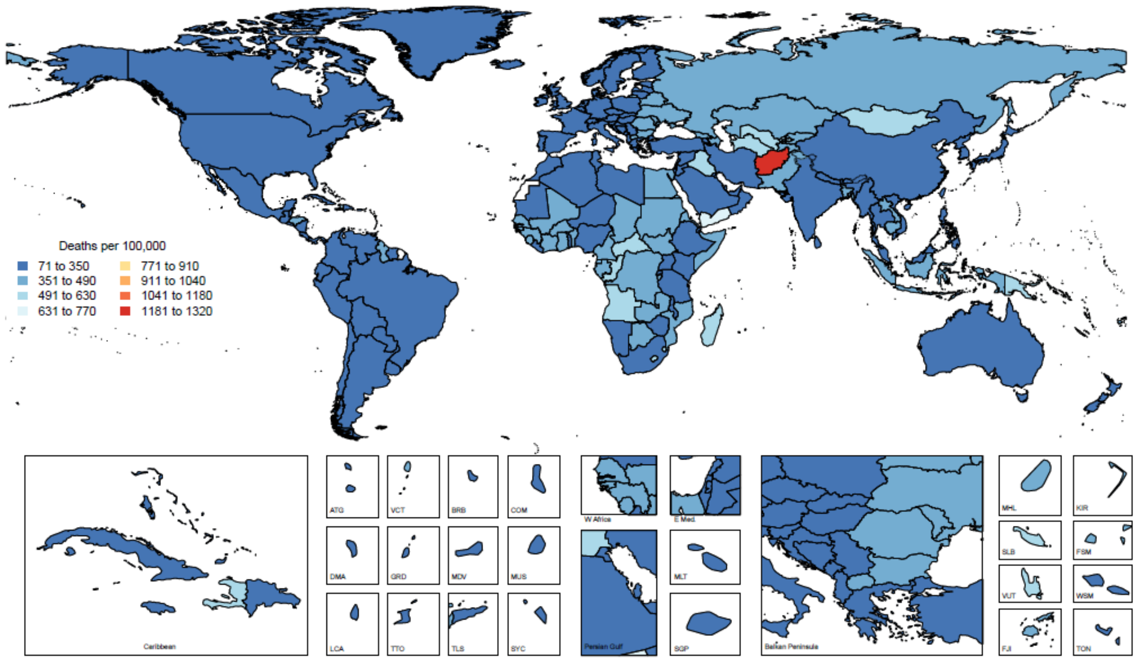

eFigure 9a.

Age−Standardized Prevalence of All Cardiovascular Disease per 100,000 Persons in 2015, Males

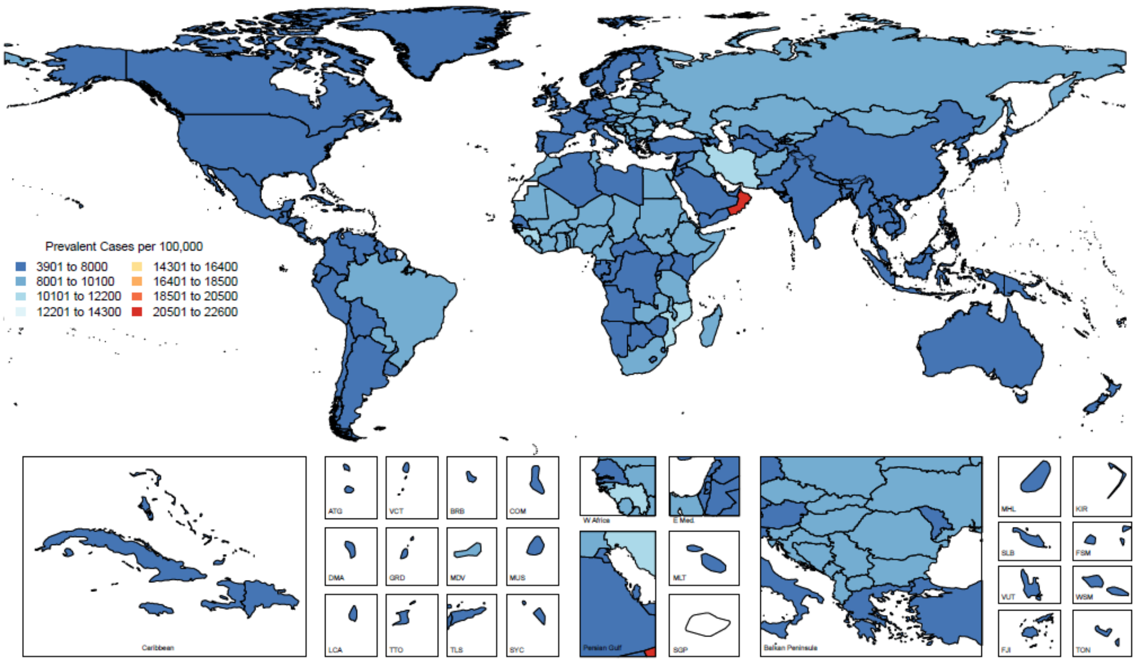

eFigure 9b.

Age−Standardized All Cardiovascular Disease, Deaths per 100,000 Persons in 2015, Males

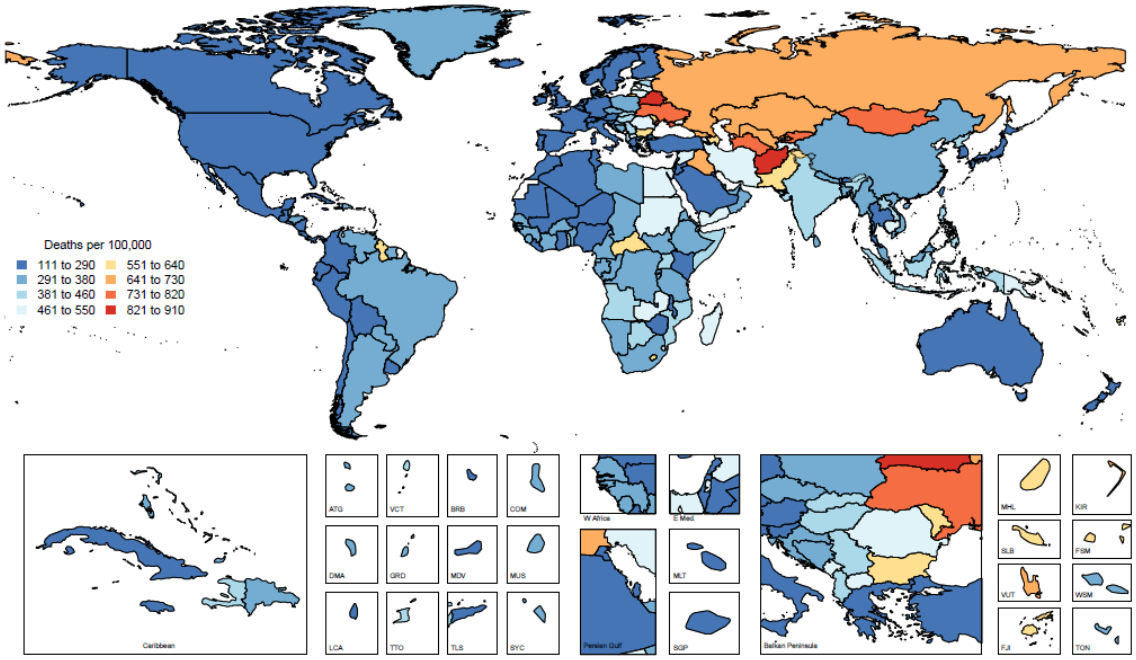

Supplement: Online Tables 1–4 and Online Figures 1–9 [file mmc1.pdf]
