# Supplementary material for: Global, Regional, and National Burden of Cardiovascular Diseases for 10 Causes, 1990 to 2015
Source: J Am Coll Cardiol. 2017 Jul 4;70(1):1–25. doi: 10.1016/j.jacc.2017.04.052 (PMC5491406; doi:10.1016/j.jacc.2017.04.052)

Methods Appendix to Global, Regional, and National Burden of Cardiovascular Diseases for 10 Causes, 1990–2015

This appendix provides further methodological detail and supplemental figures and tables. The appendix is organized into broad sections following the structure of the main paper.

Supplementary results for incidence, prevalence, and years of life lived with disability are presented in a

separate on-line file.

Contents

[Methods: Tables & Figures 4](#_Toc482280800)

[Supplementary Results: Tables & Figures 5](#_Toc482280801)

[Section 1. GBD Overview 7](#_Toc482280802)

[1.1 GATHER statement 7](#_Toc482280803)

[1.2 Geographies estimated and Socio-demographic Index 7](#_Toc482280804)

[1.3 GBD Cause List 8](#_Toc482280805)

[1.4 Data Sources 8](#_Toc482280806)

[1.5 Sources for Estimation of Mortality: Vital Registration, Sample Registration Systems, and Disease Surveillance Points 8](#_Toc482280807)

[1.6 Household Recall of Death 8](#_Toc482280808)

[1.7 Adult Population Estimates 8](#_Toc482280809)

[1.8 Noise Reduction 9](#_Toc482280810)

[1.9 Regress garbage codes versus non‐garbage 9](#_Toc482280811)

[Section 2. Causes of death modeling methods 10](#_Toc482280812)

[2.1 Overview of method 10](#_Toc482280813)

[2.2 Model pool development 11](#_Toc482280814)

[2.3 Testing model pool on 15% sample 11](#_Toc482280815)

[2.4 Ensemble development 11](#_Toc482280816)

[2.5 Testing ensembles 12](#_Toc482280817)

[2.6 Final estimation 12](#_Toc482280818)

[2.7 Covariates used for each CODEm model plus CODEm model hyper parameters 12](#_Toc482280819)

[2.8 Fit statistics for CODEm models 12](#_Toc482280820)

[2.9 Cause specific modeling methods for CVD Causes 12](#_Toc482280821)

[Cardiovascular Diseases 13](#_Toc482280822)

[Rheumatic Heart Disease 14](#_Toc482280823)

[Ischemic Heart Disease 15](#_Toc482280824)

[Cerebrovascular Disease 16](#_Toc482280825)

[Ischemic Stroke 17](#_Toc482280826)

[Hemorrhagic and Other Stroke 18](#_Toc482280827)

[Hypertensive Heart Disease 19](#_Toc482280828)

[Cardiomyopathy and Myocarditis 20](#_Toc482280829)

[Atrial Fibrillation 21](#_Toc482280830)

[Aortic Aneurysm 23](#_Toc482280831)

[Peripheral Artery Disease 24](#_Toc482280832)

[Endocarditis 25](#_Toc482280833)

[Other Cardiovascular and Circulatory Diseases 26](#_Toc482280834)

[Section 3. Nonfatal modeling methods 27](#_Toc482280835)

[Rheumatic Heart Disease 27](#_Toc482280836)

[Ischemic Heart Disease 32](#_Toc482280837)

[Cerebrovascular Disease, Ischemic Stroke & Hemorrhagic Stroke 37](#_Toc482280838)

[Acute Myocarditis 43](#_Toc482280839)

[Atrial Fibrillation and Flutter 45](#_Toc482280840)

[Peripheral Artery Disease 48](#_Toc482280841)

[Acute Endocarditis 51](#_Toc482280842)

[Other Cardiovascular Diseases 53](#_Toc482280843)

[References 54](#_Toc482280844)

# Methods: Tables & Figures

Appendix Table 1. GBD 2015 cause and sequela hierarchy with four levels of causes (1-4) and two levels of sequelae (5-6) for Cardiovascular Diseases

Appendix Table 2. Causes of death source list by geography, source type, and study site, with years, age range, and sample size

Appendix Table 3. GBD 2015 Citations Sorted by CVD Cause

Appendix Table 4. GBD 2015 Geography Hierarchy with Levels

Appendix Table 5. Socio-demographic Index (SDI) groupings by geography, based on 2015 values

Appendix Table 6. GBD 2015 CODem model covariates by CVD Cause

Appendix Table 7. CODEm predictive validity results by CVD cause, sex, age, and location

Appendix Table 8. Count of CVD literature data included for GBD 2015

Appendix Table 9. GBD 2015 nonfatal model covariates by CVD Cause

Appendix Table 10. List of International Classification of Diseases (ICD) codes mapped to the Global Burden of Disease cause list for Cardiovascular Diseases (fatal)

Appendix Table 11. Guidelines for Accurate and Transparent Health Estimates Reporting (GATHER) 18-item checklist with description of compliance and location of information for this GBD 2015 Cardiovascular Diseases publication

Appendix Figure 1. Increase in mortality in CVD after redistributing garbage codes by different years and ICD coding system

# Supplementary Results: Tables & Figures (see separate file)

Tables:

eTable 1. Global and Regional YLLs for all CVD causes, total number and age-standardized rate, by sex and

total, for 2015

eTable 2. Global and Regional YLDs for all CVD causes, total number and age-standardized rate, by sex

and total, for 2015

eTable 3a. Prevalence of CVD Causes for Years 1990-2000, with Upper and Lower Uncertainty Bounds

eTable 3b. Prevalence of CVD Causes for Years 2005-2015, with Upper and Lower Uncertainty Bounds

eTable 4a. All-ages Prevalence for men and women separately and combined, for all CVD level 3 and 4

subcauses, by country in 1990 and 2015

eTable 4b. All-ages Mortality rate for men and women separately and combined, for all CVD level 3 and 4

subcauses, by country in 1990 and 2015

eTable 4c. All-ages DALY rate for men and women separately and combined, for all CVD level 3 and 4

subcauses, by country in 1990 and 2015

Figures:

eFigure 1a. Global country map, CVD data availability in GBD modeling, by sources for fatal modelling

eFigure 1b. Global country map, CVD data availability in GBD modeling, by sources for nonfatal modelling

eFigure 2a. Global country map, percentage change in age-standardized prevalence of CVD, 1990-2015

eFigure 2b. Global country map, percentage change in age-standardized death rate of CVD, 1990-2015

eFigure 3. Ranking of age-standardized DALYs per 100,000, both sexes combined, for CVD level 3 and 4

causes globally and for 21 GDB world regions.

eFigure 4a. Prevalence of CVD level 3 and 4 causes in 2015, by age categories

eFigure 4b. Death rate for CVD level 3 and 4 causes in 2015, by age categories.

eFigure 5a. Age-Standardized Prevalence of IHD per 100,000 persons in 2015, Both Sexes

eFigure 5b. Age-Standardized Prevalence of HHD per 100,000 persons in 2015, Both Sexes

eFigure 5c. Age-Standardized Prevalence of Ischemic Stroke per 100,000 persons in 2015, Both Sexes

eFigure 5d. Age-Standardized Prevalence of Hemorrhagic Stroke per 100,000 persons in 2015, Both Sexes

eFigure 6a. Age-Standardized Deaths due to IHD per 100,000 persons in 2015, Both Sexes

eFigure 6b. Age-Standardized Deaths due to HHD per 100,000 persons in 2015, Both Sexes

eFigure 6c. Age-Standardized Deaths due to Ischemic Stroke per 100,000 persons in 2015, Both Sexes

eFigure 6d. Age-Standardized Deaths due to Hemorrhagic Stroke per 100,000 persons in 2015, Both

Sexes

eFigure 7a. Trends in Age-Standarized Incidence Rates for MI, 1990 – 2015

eFigure 7b. Trends in Age-Standarized Incidence Rates for Stroke, 1990 - 2015

eFigure 8a. Age-Standardized Prevelance of All Cardiovascular Disease per 100,000 persons in 2015,

Female

eFigure 8b. Age-Standardized All Cardiovascular Disease, Deaths per 100,000 persons in 2015, Female

eFigure 9a. Age-Standardized Prevelance of All Cardiovascular Disease per 100,000 persons in 2015, Male

eFigure 9b. Age-Standardized All Cardiovascular Disease, Deaths per 100,000 persons in 2015, Male

# Section 1. GBD Overview

## GATHER statement

This study is in compliance with the Guidelines for Accurate and Transparent Health Estimates Reporting (GATHER) recommendations. The GBD 2015 capstone papers and their respective supplementary documents contain the general methods, data sources, model selection information, performance and limitation information for the GBD 2015 analyses including detailed GATHER documentation^1,2^. Appendix Table 11 contains GATHER compliance information for this publication.

## Geographies estimated and Socio-demographic Index

Consistent with the protocol of the Global Burden of Disease Study 2015, we estimated incidence and prevalence of cardiovascular diseases from 1980-2015 for 195 countries and territories, for both sexes, and 15 five-year age groups (age group 10-14 through age group 80+). Additionally, geographies were aggregated into categories consistent with previously published GBD location hierarchies (Appendix Table 4). Estimates based on aggregation of locations were also computed by level of development using the Socio-demographic Index (SDI) methodology. Full details of the development and use of SDI levels were previously published^1,2^. Appendix Table 5 lists the GBD 2015 geographies by SDI level.

The Socio-Demographic Index (SDI) was calculated using the Human Development Index (HDI) methodology, wherein an index value was determined for each covariate input (log lag dependentincome per capita (LDI), average educational attainment in the population over age 15, and total fertility rate (TFR)):


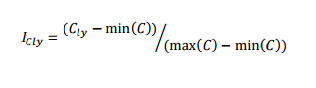


Where 𝐼𝑐𝑙𝑦 – the index for covariate C, location l, and year y – is equal to the difference between the value of that covariate in that location-year and the minimum observed value of the covariate in any location over the time interval divided by the observed range. The Socio-Demographic Index is then the geometric mean of these three indices.

For LDI and TFR, we noted diminishing gains in life expectancy at birth and 5q0 at the higher and lower terminals, respectively. Due to the significance of these values in indexing, we aimed to identify the point at which increasing income or reducing fertility no longer resulted in improved child mortality or life expectancy. We tested various restrictions, and found that capping LDI at 60,000 and setting a TFR floor at 1 resulted in improved correlations with resultant health indicators.

We further aimed to validate the use of SDI by regressing it in a variety of forms against life expectancy at birth, 5q0, 35q15, and 20q50. We found that SDI generally is as capable of predicting these demographic indicators as the previous SDS, and also as the inputs. We also found that in incorporating year, we did not substantially reduce the coefficients for SDI.

## GBD Cause List

The GBD cause List is organized into four levels, consisting of a hierarchy that is mutually exclusive and collectively exhaustive. Details on the overall GBD cause list have been documented elsewhere^1,2^. In brief, Level 1 for all CVD causes is Non-Communicable Diseases. Level 2 is the overarching Cardiovascular Diseases grouping. Disaggregation into level 3 and 4 is done to capture the finest level of detail for causes. Lastly, level 5 and 6 contains the sequalae of the CVD diseases. Appendix Table 1 contains the cause and sequelae list for Cardiovascular Diseases.

## Data Sources

A complete list of sources used in the GBD 2015 analyses is available from the GBD 2015 Data Input Sources Tool (<http://ghdx.healthdata.org/gbd-2015/data-input-sources>).

## Sources for Estimation of Mortality: Vital Registration, Sample Registration Systems, and Disease Surveillance Points

We endeavored to include all available data from vital registration systems as inputs in our all‐cause and cause-specific mortality estimation process. To achieve this, we utilized a number of multi‐country vital registration sources, including the WHO Mortality Database, the Human Mortality Database, United Nations Demographic Yearbooks and OECD databases. These multi‐country sources are regularly updated in our systems when new data are added. Beyond multi‐country sources, for all ongoing national VR systems (for example, the USA National Vital Statistics System), where possible, we cataloged all data sources from each system. Some countries that do not have a well‐performing VR system implement sample registration systems that are incomplete by design. We made use of these data, paying close attention to the proper weighting of sampled data and consistency with other representative sources. We have systematically extracted data from the Sample Registration System Statistical Report series published by the Registrar General of India. For the Disease Surveillance Points system of China, we obtained both national and provincial level DSP data through a data usage agreement with the Chinese Center for Disease Control and Prevention. Census data are systematically extracted from Demographic Yearbook series, Integrated Public Use Microdata Series (IPUMS), and statistical reports from the national statistical bureaus.

## Household Recall of Death

Household recall is ascertained from large survey series. A survey series must include a module asking about the number of deaths of household members within a given recall time period, along with a list of household members who have not died over this period of time. In addition, these survey series must be considered nationally representative, include survey weights (if applicable), and include the sex and age (either current or at death) of all household members.

## Adult Population Estimates

To calculate adult mortality rate using household death recall, age specific populations in age group 15 to 59 from the corresponding survey or census sources are used. This is also true in calculating adult mortality rate using reported deaths from Sample Registration System from India and Disease Surveillance Point system from China. For data from vital registration systems, we currently have two major sources for population in the corresponding age groups:

1. Population estimates from the World Population Prospect 2015 Revision by the United Nations

Population Division. This provides majority of the population estimates used in GBD2015.

2. For the 37 countries covered by the Human Mortality Database, we use population exposure

from this source instead of WPP2015.

For subnational locations, interpolation and extrapolation based on rate of change are used together with age specific population from censuses. Raking is applied to ensure consistency between subnational and national populations.

## Noise Reduction

To deal with problems of zero counts in vital registration, verbal autopsy, cancer registries, or sibling histories for a given age group in a given year, we use a Bayesian noise reduction algorithm. For this algorithm, we assume a normal prior and a normal data likelihood. We estimate the normal prior for a given country series of data by estimating a negative binomial for the fraction of deaths in each age group due to each respective cause with dummy variables for age and year. With two notable exceptions (detailed below), these regressions are country‐specific, so borrowing strength over age is only within a data type in a country. The variance of the prior, $\tau^{2}$, is estimated from the negative binomial regression, taking into account the variance‐covariance matrix of the regression coefficients. For the data variance, we use the Wilson approximation which provides an estimate of $\sigma^{2}$ even in cases with a zero count of cause‐specific deaths. The posterior estimate for each data point is:

$$Mean=\left( \frac{\tau^{2}}{\tau^{2}+\sigma^{2}}X+\frac{\sigma^{2}}{\tau^{2}+\sigma^{2}}\mu\right)$$

$$Variance=\left( \frac{\tau^{2}\sigma^{2}}{\tau^{2}+\sigma^{2}} \right)$$

Where X is the mean of the data and μ is the mean of the prior. This approach to noise reduction avoids the problem that zero counts in an ln rates model or a logit cause fraction model will be dropped from the regression and lead to upward bias in the estimates. This is particularly important in two settings: high‐income countries with small numbers of cause‐specific deaths, and in the analysis of sibling history data where for any given age group in any given year the number of deaths reported in the survey that are pregnancy‐related or the number of deaths from all causes in that age group may be small.

Regarding the exceptions to the regression, the first is that country‐years with populations under 1 million are pooled with the region data in order to prevent overdispersion and provide a stronger signal. Additionally, verbal autopsy data diverge from the above description in two ways. First, all data for a given super‐region are pooled together and a study dummy variable is added, allowing for different studies and surveillance sites to borrow strength from one another within a super‐region. Second, unless the data are part of a time series (e.g., Matlab HDSS), there is no year component to the regression.

## Regress garbage codes versus non‐garbage

As in GBD 2013, the statistical analysis used to determine proportions for garbage code redistribution for ill‐defined cancer sites, ill‐defined external causes of injury, unspecified stroke, heart failure, hypertension, and atherosclerosis was based on the approach outlined by Ahern et al.^3^ For each redistribution package, we defined the “universe” of data as all deaths coded to either the package’s garbage codes or the package’s redistribution targets for each country, year, age, and sex. We then ran a regression based on the following equation, separately for each target group and sex:

TGcrt = a + f31Garcrt + f32Agecrt Garcrt + 8r Garcrt + yr + Ect

TGcrt = percentage of deaths within the given garbage code’s universe which were coded to a given target group, by country

Garcrt = percentage of deaths within the given garbage code’s universe which were coded to a given set of garbage codes

a = constant

f31 = slope coefficient describing the association between Garcrt and Gcrt

f32 = slope coefficient describing the association between the interaction Agecrt Garcrt and Gcrt

yr = region specific random intercept (or super region if the random effect on region is not significant) 8r = region specific random slope (or super region if the random effect on region is not significant)

Ect = standard error, normally distributed and calculated by bootstrapping

This regression was adjusted from GBD2013 to include fixed effects on the interaction of garbage and age to ensure smooth age patterns. We made this decision after investigating diagnostic visualizations that showed unlikely gaps between proportions assigned to different age groups.

Once proportions were produced for each country, sex, age, and target group, certain adjustments were made to conform our packages to the best medical evidence available. In some cases, we implemented restrictions on the proportions that the regressions could yield. For example, we did not allow any redistribution onto Chagas disease outside of Latin America and the Caribbean, or suicide under the age of 15. In other cases, we capped the proportion for some targets to the level that would be produced from proportional redistribution; for example, hemoglobinopathies and hemolytic anemias were restricted to the level of proportional redistribution in the redistribution of left heart failure.

Occasionally, further adjustments were made on a case‐by‐case basis per country, age, sex, and target group to suppress the impact of outliers based on existing epidemiological evidence and expert judgment

# Section 2. Causes of death modeling methods

Mortality estimates for cardiovascular diseases were generated using CODEm.

## 2.1 Overview of method

CODEm is a framework for modeling most cause-specific death rates in the GBD using five core principles: 1) Identify and use all the available data in the modeling process. Though data may vary in quality it all contains some signal of the true epidemiological process. 2) Develop a diverse set of plausible models to use for estimation. That is, build a number of models capturing well-documented associations to make estimates. 3) Assess the predictive validity of each plausible individual model and of an ensemble of models created from the pool of plausible models. 4) Choose the models and ensemble model with the best performance in the out-of-sample predictive validity tests.

For some causes, separate models were run for diﬀerent age ranges when there was reason to believe that the relation between covariates and death rates might be diﬀerent in diﬀerent age ranges, for example, in children compared with adults. Separate models are developed for countries with extensive, complete, and representative VR for every cause such that uncertainty can better reflect the more complete vital registration in these locations.

## 2.2 Model pool development

As many factors covary with a particular cause of death, a large range of plausible statistical models are developed for each cause. For the CODEm framework, four families of statistical models are developed using covariates (see 2x2 table in Foreman et al).^4^ These are mixed effects linear models of the natural log of the death rate, mixed effects linear models of the logit of the cause fraction, spatiotemporal Gaussian process regression (ST-GPR) models of the log of the death rate, and ST-GPR of the logit of the cause fraction. All plausible relationships between covariates and relevant cause are identified, and all possible permutations of selected covariates are tested in linear models where the logit cause fraction or log death rate is the response variable. Because we test all permutations of covariates, multicollinearity between covariates may produce implausible signs on coefficients or unstable coefficients. All models where the sign on the coefficient is in the direction expected based on the literature and where the coefficient is statistically significant at p <0.05 are retained. We run covariate selection for both cause fractions and death rates and then create both mixed effects only and ST models for each set of covariates. For a detailed explanation of the covariate selection algorithm see Foreman et al 2012.^4^

## 2.3 Testing model pool on 15% sample

The performance of all component models and ensembles is evaluated using out-of-sample predictive validity tests. Thirty percent of the data are excluded from the initial model ﬁts, and half of that (15% of total) is used to evaluate and rank component models and then build ensembles. Data are held out from the analysis using the pattern of missingness for each cause in the cause of death database. Out-of-sample predictive validity testing is repeated until stable model results have been obtained. The out-of-sample performance tests include the root mean squared error of the log of the cause-speciﬁc death rate, the direction of the trend in the prediction compared to the data, and the validity of the 95% UI. For every model, we show the in-sample root mean squared error of the log

death rates (RMSE) and the out-of-sample performance in the 15% of data not used in the model building process.

## 2.4 Ensemble development

After component models are ranked on their out-of-sample predictive validity they are weighted based on their ranking and each component model contributes a portion to the final estimate. How much each submodel contributes is a function of its relative ranking as well as the value of psi chosen, which dictates that distribution of rankings (see Foreman et al 2012 for the details of psi distribution).^4^

## 2.5 Testing ensembles

Using the second half of the holdout data (15% of total), the differently weighted ensembles and different values of psi are tested using the same predictive validity metrics as the component models. For every model, we show the in-sample root mean squared error of the log death rates (RMSE) and the out-of-sample performance in the 15% of data not used in the model building process. The ensemble with the best average trend and RMSE is chosen as the final ensemble weighting scheme.

## 2.6 Final estimation

After a model weighting scheme has been chosen, each model contributes a number of draws proportional to its weight such that 1,000 draws are created. The mean of the draws is used as the final estimate for the CODEm process and 95% UI are created from the 0.025 and 0.975 quantiles of the draws. The ﬁnal assessment of ensemble model performance is the validity of the UIs; ideally, the 95% UI for a model would capture 95% of the data out-of-sample. Higher coverage suggests that UIs are too large and lower than 95% suggest UIs are too narrow.

## 2.7 Covariates used for each CODEm model plus CODEm model hyper parameters

For details on covariates used for CODEm models, see Appendix Table 6. CODEm covariates used, level of covariate, and expected direction of covariate by CVD cause, sex, age, and location.

## 2.8 Fit statistics for CODEm models

For details on fit statistics for CVD CODEm models, see Appendix Table 7. CODEm predictive validity results by cause, sex, age, and location.

## 2.9 Cause specific modeling methods for CVD Causes

Specific methodologies for mortality estimates for cardiovascular diseases were also described in the GBD 2015 Causes of Death Capstone paper and have been appended here for ease of access^1^. Appendix Table 10 includes a List of International Classification of Diseases (ICD) codes mapped to specific cardiovascular diseases for use in generating fatal estimates.

Flow chart legend

The legend for all flowcharts describing the modeling strategy for cause of death estimates is below.

## Cardiovascular Diseases

Input data

Vital registration, verbal autopsy, and surveillance data were used to model this cause. We outliered non-representative subnational verbal autopsies in a number of Indian states. We also outliered verbal autopsy data sources that were implausibly low in all age groups and ICD8 and ICD9 BTL data points that were inconsistent with the rest of the data and created implausible time trends.

Modeling strategy

We used a standard CODEm approach to model deaths from cardiovascular diseases. We have included two new variables, Socio-Demographic Index and the SEV scalar for rheumatic heart disease, as possible covariates for selection in the ensemble modeling process. Otherwise, there have been no substantive changes from the approach used in GBD 2013.

## Rheumatic Heart Disease

Input data

Vital registration and surveillance data were used to model rheumatic heart disease. We outliered ICD8 and ICD9 BTL data points which were inconsistent with the rest of the data and created implausible time trends. We also outliered data points which were too high after the redistribution process in a number of age groups.

Modeling strategy

We used a standard CODEm approach to model deaths from rheumatic heart disease. We have included two new variables, Socio-Demographic Index and the SEV scalar for rheumatic heart disease, as possible covariates for selection in the ensemble modeling process. Otherwise, there have been no substantive changes from the approach used in GBD 2013.

## Ischemic Heart Disease

Input data

Vital registration, verbal autopsy, and surveillance data were used to model ischemic heart disease. We outliered verbal autopsy data in countries and subnational locations where high-quality vital registration data were also available. We also outliered non-representative subnational verbal autopsy data points, ICD8 and ICD9 BTL data points which were inconsistent with the rest of the data and created implausible time trends, and data in a number of Indian states identified by experts as poor-quality.

Modeling strategy

We used a standard CODEm approach to model deaths from ischemic heart disease. We have included two new variables, Socio-Demographic Index and the SEV scalar for ischemic heart disease, as possible covariates for selection in the ensemble modeling process. Otherwise, there have been no substantive changes from the approach used in GBD 2013.

## Cerebrovascular Disease

Input data

Verbal autopsy and vital registration data were used to model this cause. We outliered non-representative subnational verbal autopsy data points. We reassigned deaths from verbal autopsy reports for cerebrovascular disease to the parent cardiovascular disease for both sexes for those under 20 years of age. We also outliered ICD8, ICD9 BTL, and ICD10 Tabulated data points which were inconsistent with the rest of the data and created implausible time trends. Data points from sources which were implausibly low in all age groups and data points that were causing the regional estimates to be improbably high were outliered.

Modeling strategy

We used a standard CODEm approach to model deaths from cerebrovascular disease. We have included two new variables, Socio-Demographic Index and the SEV scalar for cerebrovascular disease, as possible covariates for selection in the ensemble modeling process. Otherwise, there have been no substantive changes from the approach used in GBD 2013.

## Ischemic Stroke

Input data

Vital registration and surveillance data were used to model ischemic stroke. We reassigned deaths from verbal autopsy reports for ischemic stroke to the parent cardiovascular disease for both sexes for those under 20 years of age. We outliered ICD8 data points which were inconsistent with the rest of the data and created implausible time trends.

Modeling strategy

We used a standard CODEm approach to model deaths from ischemic stroke. We have included two new variables, Socio-Demographic Index and the SEV scalar for ischemic stroke, as possible covariates for selection in the ensemble modeling process. Otherwise, there have been no substantive changes from the approach used in GBD 2013.

## Hemorrhagic and Other Stroke

Input data

Vital registration and surveillance data were used to model hemorrhagic and other stroke. We reassigned deaths from verbal autopsy reports for hemorrhagic and other stroke to the parent cardiovascular disease for both sexes for those under 20 years of age. We outliered ICD8 data points which were inconsistent with the rest of the data and created implausible time trends.

Modeling strategy

We used a standard CODEm approach to model deaths from hemorrhagic and other stroke. squeWe have included two new variables, Socio-Demographic Index and the SEV scalar for hemorrhagic and other stroke, as possible covariates for selection in the ensemble modeling process. Otherwise, there have been no substantive changes from the approach used in GBD 2013.

## Hypertensive Heart Disease

Input data

Vital registration and surveillance data were used to model hypertensive heart disease. We outliered ICD9 BTL data points, which were inconsistent with the rest of the data and created implausible time trends.

Modeling strategy

We used a standard CODEm approach to model deaths from hypertensive heart disease. We have included two new variables, Socio-Demographic Index and the SEV scalar for hypertensive heart disease, as possible covariates for selection in the ensemble modeling process. Otherwise, there have been no substantive changes from the approach used in GBD 2013.

## Cardiomyopathy and Myocarditis

Input data

Vital registration and surveillance data were used to model cardiomyopathy and myocarditis. We outliered data points in Central Asia and Central and Eastern Europe due to implausibly high values which we attributed to variation in local coding practices. We also outliered ICD8 data points in countries where they were discontinuous with other data in the time series.

Modeling strategy

We used a standard CODEm approach to model deaths from cardiomyopathy and myocarditis. We have included two new variables, Socio-Demographic Index and the SEV scalar for cardiomyopathy and myocarditis, as possible covariates for selection in the ensemble modeling process. Otherwise, there have been no substantive changes from the approach used in GBD 2013. Finally, local differences in coding practices may explain some of the geographic variation that we see for deaths due to cardiomyopathy; we plan to explore this issue further in future iterations of GBD.

## Atrial Fibrillation

Input data

Vital Registration Data: We outliered ICD8 and ICD9 data points that were discontinuous from other

data in the time series and created an unlikely time trend. We also outliered data points that were

implausibly low in multiple age groups.

Modeling strategy

In order to address changes in coding practices for atrial fibrillation, we used an integrated approach

that combined DisMod-MR and CODEm models to estimate deaths from atrial fibrillation and flutter.

This approach allowed us to adjust estimates to more accurately reflect the number of deaths for which

atrial fibrillation was the true underlying cause of death. The modeling steps are illustrated in the above flowchart.

In Step 1, we estimated deaths for atrial fibrillation using a standard CODEm approach. In Step 2, we estimated prevalence rates in DisMod-MR using data from published reports of cross-sectional and cohort surveys and primary care facility data. We also used claims data covering inpatient and outpatient visits for the United States along with inpatient hospital data from 22 countries. As the inpatient hospital data only included information from the primary code for each visit, prevalence rates for these data were adjusted based on the age- and sex-specific proportions of atrial fibrillation in the primary codes vs. secondary codes in the US claims data. In Step 3, we calculated the excess mortality rate (EMR) for 2015 (defined as the cause-specific mortality rate (CSMR) estimated from CODEm divided by the prevalence rate from DisMod-MR). We then selected 27 countries based on four conditions: 1) availability of VR data; 2) prevalence rate ≥0.005; 3) CSMR ≥ 0.00002; and, 4) EMR ≥ 0.001. Using information from these countries as input data, we ran a linear mixed-effects regression of logEMR on sex, age, and location. Sex and age were treated as fixed effects for the regression, while location was considered a random effect. We then predicted age- and sex-specific EMR using the results of this regression for all non-selected countries. Countries included in the regression were assigned their directly calculated values. These EMR data points were assigned to the time period 1990–2015 and uploaded into the nonfatal database in order to be used in modeling. In Step 4, we reran DisMod-MR including the EMR estimated in Step 3 and using log-transformed lagged distributed income (LDI) as a country-level covariate. Based on information from other regressions, we set the bounds at -1.5 to -0.25.

In Steps 5 and 6, we repeated the process in Steps 3 and 4. In this iteration, we selected 31 countries

that were included in the mixed-effects regression. The criteria were: 1) availability of VR data; 2)

prevalence rate ≥ 0.004; 3) CSMR ≥ 0.00002; and, 4) EMR ≥ 0.002. The CSMR from the DisMod-MR

model in Step 6 was used as the finalized output. As DisMod-MR only generates estimates for six years

(1990, 1995, 2000, 2005, 2010, 2015), we interpolated the missing years to generate death estimates for

all years (1980–2015). These results were then uploaded into the Cause of Death database. Finally, in

Step 7, the unadjusted death estimates were run through the CodCorrect process to generate adjusted

deaths and YLLs were generated by the DALYnator using a standard reference life table.

## Aortic Aneurysm

Input data

Vital registration and surveillance data were used to model this cause. We outliered data in Oman as they were improbably high in comparison with the rest of the region. We also outliered ICD8 data that were discontinuous with the rest of the time series and created implausible time trends.

Modeling strategy

We used a standard CODEm approach to model deaths from aortic aneurysm. We have included two new variables, Socio-Demographic Index and the SEV scalar for aortic aneurysm, as possible covariates for selection in the ensemble modeling process. Otherwise, there have been no substantive changes from the approach used in GBD 2013.

## Peripheral Artery Disease

Input data

Vital registration data were used to model peripheral artery disease. We outliered all data points with <1 death in Egypt per expert review.

Modeling strategy

We used a standard CODEm approach to model deaths from peripheral artery disease. We have included two new variables, Socio-Demographic Index and the SEV scalar for peripheral artery disease, as possible covariates for selection in the ensemble modeling process. Otherwise, there have been no substantive changes from the approach used in GBD 2013.

## Endocarditis

Input data

Vital registration and surveillance data were used to model endocarditis. We outliered vital registration data in Mozambique as these were non-representative for sub-Saharan Africa and were causing regional estimates to be implausibly low. We also outliered ICD8 data that were discontinuous from the rest of the data series and created an implausible time trend.

Modeling strategy

We used a standard CODEm approach to model deaths from endocarditis. We have included two new variables, Socio-Demographic Index and the SEV scalar for endocarditis, as possible covariates for selection in the ensemble modeling process. Otherwise, there have been no substantive changes from the approach used in GBD 2013.

## Other Cardiovascular and Circulatory Diseases

Input data

Vital registration, verbal autopsy, and surveillance data were used to model other cardiovascular and circulatory diseases. We outliered ICD8 and ICD9 BTL data points that were inconsistent with the rest of the data and created implausible time trends. We also outliered ICD8 data points which were not nationally representative.

Modeling strategy

We used a standard CODEm approach to model deaths from other cardiovascular and circulatory diseases. We have included two new variables, Socio-Demographic Index and the SEV scalar for other cardiovascular and circulatory diseases, as possible covariates for selection in the ensemble modeling process. Otherwise, there have been no substantive changes from the approach used in GBD 2013.

# Section 3. Nonfatal modeling methods

Cardiovascular disease morbidity was modeled using the DisMod-MR 2.1 platform. Nonfatal estimation and modeling methods have been documented elsewhere^2^. Specific methods for cardiovascular disease estimation have been appended below for ease of access. Appendix Table 10 includes a list of International Classification of Diseases (ICD) codes used in the extraction of hospital and claims data, mapped to specific cardiovascular diseases.

Flow chart legend

The legend for all flowcharts describing the modeling strategy for nonfatal disease estimates is below.

## Rheumatic Heart Disease

Case definition

Rheumatic heart disease (RHD) was defined as a clinical diagnosis by a physician with or without confirmation using echocardiography. This case definition for echocardiographic confirmation of RHD follows the World Heart Federation criteria for echocardiographic diagnosis of rheumatic heart disease^5^.

| Criterion | Definition |
| --- | --- |
| 1. Echocardiography | Prevalent rheumatic heart disease based on echocardiographic assessment and clinical confirmation |
| 1. Clinical diagnosis | Prevalent rheumatic heart disease based on physician diagnosis |

Input data

*Model inputs*

A systematic review was performed for GBD 2013 and updated for GBD 2015. The GBD 2015 search information encompassed the following:

- Search terms: ('rheumatic heart disease' AND epidemiology[MeSH Subheading]) OR ('acute rheumatic fever' AND epidemiology[MeSH Subheading]) OR ('rheumatic fever' AND epidemiology[MeSH Subheading]) OR (RHD AND epidemiology[MeSH Subheading]) OR ('valvular heart disease' AND epidemiology[MeSH Subheading]) OR (((streptococcus OR streptococci) AND heart) AND epidemiology[MeSH Subheading]) OR (heart AND valve AND disease AND epidemiology[MeSH Subheading]) OR ('mitral valve stenosis' AND epidemiology[MeSH Subheading]) OR (('rheumatic heart disease' OR 'rheumatic fever') AND prevalence) OR (('rheumatic heart disease' OR 'rheumatic fever') AND incidence) OR (('rheumatic heart disease' OR 'rheumatic fever') AND ('standardized mortality ratio' OR SMR)) OR ('rheumatic heart disease' OR 'rheumatic fever' AND 'case fatality')
- Dates included in search: 1/1/2013 – 3/16/2015
- Number of initial hits: 2,045
- Number of sources included: 17

These differed from the GBD 2013 search terms:

- (hasabstract[text] AND Humans[Mesh] AND middle age[MeSH])) OR 21) AND ((rheumatic heart disease/epidemiology[Mesh] OR rheumatic heart disease/mortality[Mesh]) AND (prevalence[Title/Abstract] OR incidence[Title/Abstract]) AND ("2010"[Date - Publication] : "3000"[Date - Publication]) AND (hasabstract[text] AND Humans[Mesh] AND middle age[MeSH]))

The table below illustrates the number of literature studies included in GBD 2015, as well as the number of countries or subnational units and GBD world regions represented.

| Endemic model | | | |
| --- | --- | --- | --- |
|  | Prevalence | Incidence | Mortality risk |
| Studies | 77 | 0 | 0 |
| Countries/subnationals | 45 | 0 | 0 |
| GBD world regions | 12 | 0 | 0 |

| Non-endemic model | | | |
| --- | --- | --- | --- |
|  | Prevalence | Incidence | Mortality risk |
| Studies | 8 | 0 | 0 |
| Countries/subnationals | 7 | 0 | 0 |
| GBD world regions | 4 | 0 | 0 |

We did not include any non-literature-based data types other than the hospital and claims data described elsewhere. Hospital and claims data were available only for the non-endemic country model. We excluded all outpatient data, as they were implausibly low when compared with inpatient data from the same locations and claims data. We also excluded inpatient hospital data from Canada as these were implausibly low when compared with other locations in the region or super-region.

For the endemic country model, we included study-level covariates to crosswalk studies that did not include electrocardiographic confirmation of the disease diagnosis. For the non-endemic country model, we included study-level covariates for inpatient hospital data and claims data from 2000 and 2010 to adjust these data points, using as reference the data obtained from literature and claims data from 2012.

*Severity splits and disability weights*

| Severity level | Lay description | DISABILITY WEIGHT (95% CI) |
| --- | --- | --- |
| Rheumatic heart disease, not including heart failure | Has a chronic disease that requires medication every day and causes some worry but minimal interference with daily activities. | 0.049 (0.031-0.072) |

Modeling strategy

For GBD 2015 estimation, we ran two models – one for non-endemic countries and one for endemic countries. We defined endemicity based on GBD 2015 RHD mortality estimates for the year 2015. We used a threshold of 0.15/100,000 deaths in children aged 5-9; countries with less than this number of deaths in 2015 were categorized as high-income, while countries with at least this number of deaths in 2015 were categorized as low-income.

Non-endemic country model: We included hospital data, claims data, and limited literature data on prevalence. We also included CSMR from our mortality estimates of RHD. A prior of no remission was set for all ages, and excess mortality was capped at 0.1 for all ages. We included study-level covariates for inpatient data and claims data from 2000 and 2010, cross-walking them to data from the literature and claims data in 2012. We also included the natural log of lagged distributed income (lnLDI, I$ per capita) as a country-level covariate for excess mortality, with bounds of -0.5 to -0.1.

Endemic country model: We included prevalence data from surveys published in the literature. As with the high-income model, we included CSMR from our mortality estimates of RHD. A prior of no remission was set for all ages, and excess mortality was capped at 0.07, the highest observed mean excess mortality rate data point observed in this model. We also set priors of 0 on incidence for ages 0 to 1, and 50 to 100 to account for patterns of incidence in endemic countries. We used lnLDI as fixed-effect country-level covariates on prevalence and excess mortality, enforcing an inverse relationship for both. The log-transformed, age-standardized SEV scalar was also used as a fixed-effect country-level covariate on prevalence.

We then combined estimates from the endemic and non-endemic models, selecting estimates for the countries identified as non-endemic from the non-endemic model and estimates for the countries identified as endemic from the endemic model. Estimates of heart failure due to RHD were then subtracted from the estimates for RHD, giving the overall prevalence of RHD without heart failure. A description of heart failure due to RHD can be found in the Heart Failure section of the appendix. We evaluated models based on comparing estimates with input data as well as estimates from previous rounds of GBD.

The table below shows the country covariates, parameters, betas, and exponentiated betas:

| **Covariate** | **Parameter** | **beta** | **Exponentiated beta** |
| --- | --- | --- | --- |
| *Endemic model* | | | |
| LDI (I$ per capita) | prevalence | -.0815(-.2114 - -.0037) | .9217(.8094 - .9963) |
| Log-transformed age-standardized SEV scalar: RHD | prevalence | 1.086(.5452 - 1.483) | 2.962(1.725 - 4.406) |
| LDI (I$ per capita) | excess mortality rate | -.3748(-.4892 - -.2336) | .6874(.6131 - .7917) |
| *Non-endemic model* | | | |
| US Claims 2000 | prevalence | .3339(.2491 - .4236) | 1.396(1.283 - 1.527) |
| US Claims 2010 | prevalence | .6061(.5419 - .6678) | 1.833(1.719 - 1.95) |
| LDI (I$ per capita) | excess mortality rate | -.4849(-.4999 - -.465) | .6158(.6066 - .6281) |

We changed the process of selecting countries for the endemic and non-endemic country models. In previous rounds, this decision had been based on country development status and income level, and the models were referred to as low-income and high-income models. However, because our models are trying to capture differences between countries where RHD is endemic and developed countries where the disease is extremely rare, we are now using death data to identify which model should be used for estimates for each country. We set a threshold of 0.15 deaths per 100,000 in children ages 5-9 based on expert opinion. Countries with at least this number of deaths in 2015 were considered endemic for the purposes of RHD modeling, while countries with fewer than this number of deaths were considered non-endemic. Exceptions were applied when data existed to support high childhood endemicity for RHD; because of this, Kenya and Nicaragua were moved to the endemic country category despite relatively low estimates of RHD mortality in this age range. Lists of countries as categorized to endemic or non-endemic are below.

**Endemic Locations:** North Korea, Cambodia, Indonesia, Laos, Maldives, Myanmar, Philippines, Timor-Leste, Fiji, Kiribati, Marshall Islands, Federated States of Micronesia, Papua New Guinea, Samoa, Solomon Islands, Tonga, Vanuatu, Azerbaijan, Georgia, Kyrgyzstan, Turkmenistan, Uzbekistan, Albania, Belize, Dominica, Dominican Republic, Grenada, Guyana, Haiti, Jamaica, Saint Lucia, Saint Vincent and the Grenadines, Suriname, Bolivia, Nicaragua, Algeria, Egypt, Iraq, Libya, Morocco, Syria, Tunisia, Yemen, Afghanistan, Bangladesh, Bhutan, Nepal, Pakistan, Angola, Central African Republic, Congo, Democratic Republic of the Congo, Equatorial Guinea, Gabon, Burundi, Djibouti, Eritrea, Ethiopia, Madagascar, Malawi, Mauritius, Mozambique, Rwanda, Somalia, Tanzania, Uganda, Zambia, Botswana, Lesotho, Namibia, Swaziland, Zimbabwe, Benin, Burkina Faso, Cameroon, Chad, Cote d'Ivoire, The Gambia, Ghana, Guinea, Guinea-Bissau, Liberia, Mali, Mauritania, Niger, Nigeria, Sao Tome and Principe, Senegal, Sierra Leone, Togo, South Sudan, Sudan, China, India, Kenya, South Africa

**Non-endemic Locations:** Taiwan, Malaysia, Sri Lanka, Thailand, Vietnam, Armenia, Kazakhstan, Mongolia, Tajikistan, Bosnia and Herzegovina, Bulgaria, Croatia, Czech Republic, Hungary, Macedonia, Montenegro, Poland, Romania, Serbia, Slovakia, Slovenia, Belarus, Estonia, Latvia, Lithuania, Moldova, Russia, Ukraine, Brunei, South Korea, Singapore, Australia, New Zealand, Andorra, Austria, Belgium, Cyprus, Denmark, Finland, France, Germany, Greece, Iceland, Ireland, Israel, Italy, Luxembourg, Malta, Netherlands, Norway, Portugal, Spain, Switzerland, Argentina, Chile, Uruguay, Canada, Antigua and Barbuda, The Bahamas, Barbados, Cuba, Trinidad and Tobago, Ecuador, Peru, Colombia, Costa Rica, El Salvador, Guatemala, Honduras, Panama, Venezuela, Paraguay, Bahrain, Iran, Jordan, Kuwait, Lebanon, Palestine, Oman, Qatar, Turkey, United Arab Emirates, Comoros, Seychelles, Cape Verde, Japan, Sweden, United Kingdom, United States, Mexico, Brazil, Saudi Arabia, Greenland

Other than this difference, there were no substantive changes in the modeling strategy from GBD 2013.

## Ischemic Heart Disease

Case definition

1. Acute myocardial infarction (AMI): Definite and possible AMI according to the third universal definition of myocardial infarction^6^:
   1. When there is clinical evidence of myocardial necrosis in a clinical setting consistent with myocardial ischemia or
   2. Detection of a rise and/or fall of cardiac biomarker values and with at least one of the following: i) symptoms of ischemia, ii) new or presumed new ST-segment-T wave changes or new left bundle branch block, iii) development of pathological Q waves in the ECG, iv) imaging evidence of new loss of viable myocardium or new regional wall motion abnormality, or v) identification of an intracoronary thrombus by angiography or autopsy.
   3. Sudden (abrupt) unexplained cardiac death, involving cardiac arrest or no evidence of a noncoronary cause of death
   4. Prevalent AMI is condiered to last from the onset of the event to 28 days after the event and is divided into an acute phase (0-2 days) and subacute (3-28 days).
2. Chronic IHD
   1. Angina; clinically diagnosed stable exertional angina pectoris or definite angina pectoris according to the Rose Angina Questionnaire^7^, physician diagnosis, or taking nitrate medication for the relief of chest pain.
   2. Asymptomatic ischemic heart disease following myocardial infarction; survival to 28 days following incident AMI. The GBD study does not use estimates based on ECG evidence for prior MI, due to its limited specificity and sensitivity^8^.

Input data

*Model inputs*

Myocardial infarction

A systematic review was done for myocardial infarction for GBD 2015.

The dates of the search were 1/1/2009 – 2/3/2015. 38,522 studies were returned; 194 were extracted (this number includes extractions that were done for STEMI/NSTEMI models and revascularization models that are not currently part of the MI modeling process but may be in the future).

Literature data included: Myocardial infarction

|  | Prevalence | Incidence | Mortality risk |
| --- | --- | --- | --- |
| Studies | 0 | 93 | 61 |
| Countries/subnationals | 0 | 39 | 32 |
| GBD world regions | 0 | 8 | 10 |

Apart from inpatient hospital and inpatient claims data, we did not include any data from sources other than the literature for myocardial infarction. We excluded data with broad age ranges where it was impossible to obtain more granular data, as these data caused the known age pattern for increased risk of myocardial infarction to be masked in the estimates generated from DisMod.

We corrected inpatient hospital data and claims data to account for the fact that these data sources do not capture the out-of-hospital cardiac arrest deaths which are part of the universal definition of AMI. We also included a covariate to correct for the change in diagnostic criteria to include troponin measurements. This adjustment was applied to data collected before 2000. We also adjusted data points that were not specific about whether it was the first AMI for included subjects, using studies where only first events were included as the reference. We also adjusted estimates from studies that only included non-fatal cases using study-level covariates.

Angina

A systematic review was not performed for GBD 2015. Updates to systematic reviews are performed on an ongoing schedule across all GBD causes; an update for angina will be performed in the next one to two iterations.

A systematic review for angina was done for GBD 2013. The search terms for that are here: (Angina Pectoris/epidemiology[Mesh] OR Angina Pectoris/mortality[Mesh] ) AND (prevalence[Title/Abstract] OR incidence[Title/Abstract]) AND ("2010"[Date - Publication] : "3000"[Date - Publication])

Literature data included: Angina

|  | Prevalence | Incidence | Mortality risk |
| --- | --- | --- | --- |
| Studies | 72 | 0 | 7 |
| Countries/subnationals | 73 | 0 | 7 |
| GBD world regions | 20 | 0 | 5 |

We included survey data (including NHANES and World Health Study questionnaires) which included the RAQ items. Prevalence of angina was calculated using the standard algorithm to determine whether the RAQ was positive or negative.

We excluded data with broad age ranges where it was impossible to obtain more granular data, as these data caused the known age pattern for increased risk of angina to be masked in the estimates generated from DisMod.

We included sex- and age group-specific covariates to adjust prevalence data points obtained from the RAQ using the claims data as the reference since the RAQ has been shown to be neither sensitive nor specific.

*Severity split inputs*

Acute myocardial infarction was split into two severity levels by length of time since the event – days 1 and 2 versus days 3 through 28. Disability weights were established for these two severities using the standard approach for GBD 2015.

Angina was split into mild, moderate, and severe groups using information from the Medical Expenditure Panel Survey (MEPS). Disability weights were established for these severities using the standard approach for GBD 2015.

Acute myocardial infarction

| Severity level | Lay description | DISABILITY WEIGHT (95% CI) |
| --- | --- | --- |
| Acute myocardial infarction, days 1-2 | Has severe chest pain that becomes worse with any physical activity. The person feels nauseated, short of breath, and very anxious. | 0.432 (0.288-0.579) |
| Acute myocardial infarction, days 3-28 | Gets short of breath after heavy physical activity, and tires easily, but has no problems when at rest. The person has to take medication every day and has some anxiety. | 0.074 (0.049-0.105) |

Angina pectoris

| Severity level | Lay description | DISABILITY WEIGHT (95% CI) |
| --- | --- | --- |
| Mild angina | Has chest pain that occurs with strenuous physical activity, such as running or lifting heavy objects. After a brief rest, the pain goes away. | 0.033 (0.02-0.052) |
| Moderate angina | Has chest pain that occurs with moderate physical activity, such as walking uphill or more than half a kilometer (around a quarter-mile) on level ground. After a brief rest, the pain goes away. | 0.08 (0.052-0.113) |
| Severe angina | Has chest pain that occurs with minimal physical activity, such as walking only a short distance. After a brief rest, the pain goes away. The person avoids most physical activities because of the pain. | 0.167 (0.11-0.24) |

Modeling strategy

Myocardial infarction

- - We first calculated custom cause-specific mortality estimates using data from cause of death data prior to garbage code redistribution, generating age-sex-country specific proportions of IHD deaths that were due to AMI (acute IHD) vs those due to other causes of IHD (chronic IHD). Estimates of this proportion for all locations were then generated using a DisMod proportion-only model. This proportion was multiplied by post-CodCorrect (final GBD estimtes) IHD deaths to generate CSMR estimates for AMI, even though GBD reports only deaths for all IHD taken together. These data were then used, along with incidence and excess mortality data, in a DisMod model to estimate the prevalence and incidence of myocardial infarction due to ischemic heart disease.
  - These estimates were split into prevalence and incidence estimates for days 1-2 and days 3-28 post event. Disability weights were assigned to each of these two groupings.
  - We set a value prior of one month for remission (11/13) from the AMI health state. We also set a value prior for the maximum excess mortality rate of 10 for all ages. We included lnLDI as a fixed-effect country-level covariate on excess mortality, forcing an inverse relationship.

| Study covariate | Parameter | Beta | Exponentiated beta |
| --- | --- | --- | --- |
| Diagnostic blood sample (troponin) | incidence | -.4432(-.4543 to -.44) | .6419 (.6349 to .644) |
| Hospital data | incidence | -1.5e-04  (-4.4e-04 to -4.1e-05) | .9999(.9996 to 1) |
| First ever MI | incidence | -.002(-.009 to -5.1e-05) | .998(.9911 to .9999) |
| Non fatal MI | incidence | -8.9e-04(-.0024 to -2.3e-04) | .9991(.9976 to .9998) |
| LDI (I$ per capita) | excess mortality rate | -.1005(-.1027 to -.1) | .9044(.9024 to .9048) |

Asymptomatic ischemic heart disease

- - Excess mortality estimates from the myocardial infarction model were used to generate data of the incidence of surviving 28 days post-event.
  - We used these data, along with the estimates of CSMR due to chronic IHD (the other part of the proportion described in step 1) and excess mortality data in a DisMod model to estimate the prevalence of persons with IHD following myocardial infarction. This estimate included subjects with angina and heart failure; a proportion of this prevalence was removed in order to avoid double counting based on evidence from the literature (2). The result of this step generates estimates of asymptomatic ischemic heart disease following myocardial infarction.
  - We set a value prior of 0 for remission for all ages.

| Study covariate | Parameter | Beta | Exponentiated beta |
| --- | --- | --- | --- |
| Log-transformed age-standardized SEV scalar: IHD | incidence | .9319(.9187 to .9452) | 2.539(2.506 to 2.573) |

Angina

- - We used prevalence data from the literature and USA claims databases, along with data on mortality risk to estimate the prevalence and incidence of angina for all locations.
  - The proportion of mild, moderate, and severe angina was determined by the standard approach for severity splitting for GBD 2015.
  - We included a value prior of 0 for remission for all ages. We also included a value prior of 1 for excess mortality for all ages.
  - We included age- and sex-specific study-level covariates to adjust data points based on RAQ, using data points from the claims database as the reference.
  - We also included the log-transformed, age-standardized SEV scalar for IHD as a fixed effect country-level covariate.

| Study covariate | Parameter | Beta | Exponentiated beta |
| --- | --- | --- | --- |
| RAQ, female, less than 50 | prevalence | 2.435(2.326 - 2.497) | 11.42(10.24 - 12.15) |
| RAQ, male, less than 50 | prevalence | .9454(.9349 - .9499) | 2.574(2.547 - 2.585) |
| RAQ, female, 50 to 64 | prevalence | 1.484(1.447 - 1.5) | 4.411(4.25 - 4.482) |
| RAQ, male, 50 to 64 | prevalence | .9897(.9606 - .9997) | 2.69(2.613 - 2.717) |
| RAQ, female, 65 plus | prevalence | .2929(.2719 - .2998) | 1.34(1.312 - 1.35) |
| RAQ, male, 65 plus | prevalence | .2891(.2582 - .2997) | 1.335(1.295 - 1.349) |
| Log-transformed age-standardized SEV scalar: IHD | prevalence | 1.238(1.209 - 1.249) | 3.449(3.35 - 3.487) |

Apart from inclusion of hospital data and claims data, there have been no substantive changes in the modeling strategy for myocardial infarction, asymptomatic ischemic heart disease following myocardial infarction, and angina from GBD 2013.

## Cerebrovascular Disease, Ischemic Stroke & Hemorrhagic Stroke

Case definition

Stroke was defined according to WHO criteria – rapidly developing clinical signs of focal (at times global) disturbance of cerebral function lasting more than 24 hours or leading to death with no apparent cause other than that of vascular origin^9^. Data on transient ischemic attack (TIA) were not included.

#### Acute stroke: Stroke cases are considered acute from the data of incidence of a first ever stroke through day 28 following the event.

#### Chronic stroke: Stroke cases are considered chronic beginning 28 days following the occurrence of an event. Chronic stroke includes the sequelae of an acute stroke AND all recurrent stroke events. GBD 2015 adopts this broader definition of chronic stroke than prior iterations in order to model acute strokes using only first-ever incident events.

*Ischemic stroke:* Incident ischemic stroke is defined as the occurrence of first-ever ischemic stroke, based on clinical diagnosis by a physician using diagnostic imaging. Ischemic strokes are considered to include all vascular events leading to limited blood flow to brain tissue, with resulting infarction, including atherosclerotic and thromboembolic strokes but excluding strokes in which the underlying cause is intracranial hemorrhage.

*Hemorrhagic or other strokes:* This cause includes all non-ischemic strokes of a vascular cause including subarachnoid and stroke due to intracranial hemorrhage.

Input data

*Model inputs*

A systematic review was not performed for GBD 2015. Updates to systematic reviews are performed on an ongoing schedule across all GBD causes; an update for cerebrovascular disease will be performed in the next iteration.

A systematic review of the literature was performed in GBD 2013

- - - Search terms:
      - (stroke[Mesh]) AND (prevalence[Title/Abstract] OR incidence[Title/Abstract]) AND ("2010"[Date - Publication] : "3000"[Date - Publication]) AND (hasabstract[text] AND Humans[Mesh] AND middle age[MeSH]))
      - (hasabstract[text] AND Humans[Mesh] AND middle age[MeSH])) OR 21) AND ((hemorrhagic stroke/epidemiology[Mesh] OR hemorrhagic stroke/mortality[Mesh]) AND (prevalence[Title/Abstract] OR incidence[Title/Abstract]) AND ("2010"[Date - Publication] : "3000"[Date - Publication]) AND (hasabstract[text] AND Humans[Mesh] AND middle age[MeSH]))

The tables below indicates the number of literature studies included in GBD 2015, as well as the number of countries or subnational units and GBD world regions represented.

*Cerebrovascular disease*

|  | Prevalence | Incidence | Mortality risk |
| --- | --- | --- | --- |
| Studies | 53 | 0 | 8 |
| Countries/subnationals | 50 | 0 | 4 |
| GBD world regions | 14 | 0 | 2 |

*Ischemic stroke*

|  | Prevalence | Incidence | Mortality risk |
| --- | --- | --- | --- |
| Studies | 0 | 71 | 45 |
| Countries/subnationals | 0 | 59 | 48 |
| GBD world regions | 0 | 12 | 17 |

*Hemorrhagic or other stroke*

|  | Prevalence | Incidence | Mortality risk |
| --- | --- | --- | --- |
| Studies | 0 | 71 | 34 |
| Countries/subnationals | 0 | 59 | 43 |
| GBD world regions | 0 | 12 | 11 |

In addition to inpatient hospital data, we included unpublished stroke registry data for acute ischemic and acute hemorrhagic strokes. We include survey data for chronic cerebrovascular disease. These surveys were identified based on expert opinion and review of major survey series focused on world health that included questions regarding self-reported history of stroke.

We included crosswalks to adjust data for first and recurrent strokes combined, using data for first strokes only as reference. We also included crosswalks for ischemic and hemorrhagic strokes combined (all stroke), using as reference studies with subtype-specific information.

*Severity split inputs*

The standard GBD approach using MEPS data was used to determine severity splits for stroke. The table below illustrates the severity level, lay description, and disability weights for GBD 2015.

| Severity level | Lay description | DISABILITY WEIGHT (95% CI) |
| --- | --- | --- |
| Stroke, long-term consequences, mild | has some difficulty in moving around and some weakness in one hand, but is able to walk without help. | 0.019 (0.01-0.032) |
| Stroke, long-term consequences, moderate | has some difficulty in moving around, and in using the hands for lifting and holding things, dressing, and grooming. | 0.07 (0.046-0.099) |
| Stroke, long-term consequences, moderate plus cognition problems | has some difficulty in moving around, in using the hands for lifting and holding things, dressing and grooming, and in speaking. The person is often forgetful and confused. | 0.316 (0.206-0.437) |
| Stroke, long-term consequences, severe | is confined to bed or a wheelchair, has difficulty speaking and depends on others for feeding, toileting, and dressing. | 0.552 (0.377-0.707) |
| Stroke, long-term consequences, severe plus cognition problems | is confined to bed or a wheelchair, depends on others for feeding, toileting, and dressing, and has difficulty speaking, thinking clearly, and remembering things. | 0.588 (0.411-0.744) |

Modeling strategy

Three general approaches were employed for all of the components of the stroke modeling process, detailed in the table below.

- Data were crosswalked from nonstandard to standard case definitions using DisMod for all models. Coefficients for these crosswalks can be found in the tables for fixed effects located below.
- A GBD Standardized Exposure Variable for stroke and a covariate for country income were used as country-level covariates for all models. Coefficients for these covariates can be found in the tables for fixed effects located below.
- DisMod MR-2.1 was set with priors related to the coefficients of variation and heterogeneity for each model. Information for these parameters can be found in the tables of model parameters located below.

*Step 1*

- - We generated estimates for first-ever acute ischemic and first-ever acute hemorrhagic stroke using data collected on stroke incidence and excess mortality. We set value priors of 11 to 13 on remission for all ages to establish a one-month duration for these acute sequelae.
  - We then calculated the incidence of surviving 28 days after an acute event for both ischemic and hemorrhagic stroke using the modeled estimates of excess mortality and incidence.
  - These survivor data were then uploaded into the chronic stroke, any type model as incidence.
  - We then ran the chronic stroke model, using the survivor incidence data, prevalence data, and excess mortality data. We set a value prior of 0 on remission for all ages.
  - Implausible or extreme outliers were dropped from these estimation results.
  - From these three models, we generated the proportions of deaths for acute ischemic, acute hemorrhagic, and chronic stroke, and split the post-CodCorrect stroke deaths generated from the GBD mortality estimates into these three parts. Thus, the proportion of deaths due to acute ischemic, acute hemorrhagic, and chronic stroke are driven by all available data on incidence, prevalence, and excess mortality data for stroke. These CSMR estimates were then uploaded into the nonfatal database and used to estimates models for Step 2.

*Step 2*

- - We re-ran the first-ever acute ischemic and first-ever acute hemorrhagic models with CSMR as derived from CodCorrect and epidemiologic data as described above. Twenty-eight-day survivorship was recalculated from these models and uploaded into the chronic stroke, any type model with CSMR. As for acute models, this chronic model usises CSMR as derived from CodCorrect and epidemiologic data as described above.
  - Implausible or extreme outliers were dropped from these estimation results.
  - We then split the overall chronic stroke model into chronic hemorrhagic stroke and chronic ischemic stroke based on the ratio of 28-day survivorship in the acute ischemic and acute hemorrhagic models. The assumption built into this step is that the ratio of prevalent cases of chronic stroke matches that ratio of chronic stroke survivor cases at 29 days following an incident stroke.

Models were evaluated based on expert opinion, comparison with previous iterations, and model fit.

As described above, in GBD 2015 we are no longer directly estimating first and recurrent stroke combined. This decision was made in consultation with GBD Stroke experts and reflects the fact that standard data reporting for stroke registries is for first-ever stroke. The majority of stroke incidence data available to GBD is for first-ever stroke.

The table below indicates the covariates used by cause in the estimation process, as well as the beta and exponentiated beta values.

Step 1:

| Cause | Variable name | Measure | beta | Exponentiated beta |
| --- | --- | --- | --- | --- |
| Chronic stroke; any type | Log-transformed age-standardized SEV scalar: Stroke | prevalence | .7833(.7512 to .8785) | 2.189(2.12 to- 2.407) |
| Chronic stroke; any type | LDI (I$ per capita) | excess mortality rate | -.1792(-.1819 to -.1769) | .836(.8337 to .8379) |
| First ever acute hemorrhagic stroke | Hospital data | incidence | .5278(.5223 to .5298) | 1.695(1.686 to 1.699) |
| First ever acute hemorrhagic stroke | Any stroke | incidence | 1.359(1.313 to 1.388) | 3.892(3.717 to 4.007) |
| First ever acute hemorrhagic stroke | First-ever acute stroke, ischemic or hemorrhagic | incidence | .4925(.4163 to .5291) | 1.636(1.516 to 1.697) |
| First ever acute hemorrhagic stroke | Log-transformed age-standardized SEV scalar: hemorrhagic stroke | incidence | 1.243(1.227 to 1.25) | 3.468(3.411 to 3.49) |
| First ever acute hemorrhagic stroke | Any stroke | excess mortality rate | -.4216(-.5741 to -.2617) | .656(.5632 to .7698) |
| First ever acute hemorrhagic stroke | First-ever acute stroke, ischemic or hemorrhagic | excess mortality rate | -.1409(-.3484 to .0613) | .8685(.7058 to 1.063) |
| First ever acute ischemic stroke | Hospital data | incidence | .002(4.3e-05 to .0067) | 1.002(1 to 1.007) |
| First ever acute ischemic stroke | Any stroke | incidence | .4687(.4653 to .47) | 1.598(1.592 to 1.6) |
| First ever acute ischemic stroke | First-ever acute stroke, ischemic or hemorrhagic | incidence | .5142(.4772 to .5296) | 1.672(1.612 to 1.698) |
| First ever acute ischemic stroke | Log-transformed age-standardized SEV scalar: ischemic stroke | incidence | 1.106(1.025 to 1.186) | 3.021(2.787 to 3.274) |

Step 2:

| Cause | Variable name | Measure | beta | Exponentiated beta |
| --- | --- | --- | --- | --- |
| Chronic stroke, any type with CSMR | Log-transformed age-standardized SEV scalar: Stroke | prevalence | .8185  (.7518 - .9986) | 2.267 (2.121 - 2.714) |
| Chronic stroke, any type with CSMR | LDI (I$ per capita) | excess mortality rate | -.1879 (-.1917 - -.1845) | .8287 (.8256 - .8315) |
| First-ever acute hemorrhagic stroke with CSMR | Any stroke | incidence | 1.401 (1.4 - 1.407) | 4.06 (4.055 - 4.084) |
| First-ever acute hemorrhagic stroke with CSMR | First-ever acute stroke, ischemic or hemorrhagic | incidence | 9.8e-04 (2.2e-04 - .0049) | 1.001 (1 - 1.005) |
| First-ever acute hemorrhagic stroke with CSMR | Log-transformed SEV scalar: Hem stroke | incidence | 1.152 (1.031 - 1.243) | 3.164 (2.804 - 3.466) |
| First-ever acute hemorrhagic stroke with CSMR | Any stroke | excess mortality rate | -.5999 (-.7527 - -.4538) | .5489 (.4711 - .6352) |
| First-ever acute hemorrhagic stroke with CSMR | First-ever acute stroke, ischemic or hemorrhagic | excess mortality rate | -.2336 (-.512 - .0366) | .7917 (.5993 - 1.037) |
| First-ever acute ischemic stroke with CSMR | Any stroke | incidence | .3452 (.3401 - .3575) | 1.412 (1.405 - 1.43) |
| First-ever acute ischemic stroke with CSMR | First-ever acute stroke, ischemic or hemorrhagic | incidence | 3.4e-04 (7.3e-05 - 9.8e-04) | 1 (1 - 1.001) |
| First-ever acute ischemic stroke with CSMR | Log-transformed age-standardized SEV scalar: Ischemic stroke | incidence | 1.248 (1.24 - 1.25) | 3.483 (3.456 - 3.49) |
| First-ever acute ischemic stroke with CSMR | Any stroke | excess mortality rate | -.6897 (-.8029 - -.5741) | .5017 (.448 - .5632) |
| First-ever acute ischemic stroke with CSMR | First-ever acute stroke, ischemic or hemorrhagic | excess mortality rate | -.869 (-.9992 - -.7466) | .4194 (.3682 - .474) |

## Acute Myocarditis

Case definition

#### Myocarditis refers to a heterogenous group of diseases with variable clinical and pathological features. Acute myocarditis was defined for GBD as the acute and time-limited symptoms of myocarditis separate from its chronic heart failure-related sequelae. Heart failure due to cardiomyopathy is estimated separately in GBD (see methods for heart failure). Symptoms of acute myocarditis are nonspecific and include a flu-like or gastrointestinal syndrome, followed by anginal-type chest pain, arrhythmias, syncope, or heart failure.

Input data

*Model inputs*

The preferred data sources for acute myocarditis was hospital admission data and other health facility data identifying cases of acute myocarditis.

A systematic review was performed for GBD 2013 and updated for GBD 2015. The search terms included:

(cardiomyopathy AND epidemiology[MeSH Subheading]) OR (myocarditis AND epidemiology[MeSH Subheading]) OR (cardiomyopathy AND (incidence OR prevalence OR “case fatality”)) OR (myocarditis AND (incidence OR prevalence OR “case fatality”))

- - - Dates included in search: 1/1/2013 – 3/16/2015
    - Number of initial hits: 3,598
    - Number of sources included: 0

The GBD 2013 search terms included:

(hasabstract[text] AND Humans[Mesh] AND middle age[MeSH])) OR 21) AND ((cardiomyopathy/epidemiology[Mesh] OR cardiomyopathy/mortality[Mesh]) AND (prevalence[Title/Abstract] OR incidence[Title/Abstract]) AND ("2010"[Date - Publication] : "3000"[Date - Publication]) AND (hasabstract[text] AND Humans[Mesh] AND middle age[MeSH]))

We did not include any non-literature-based data, apart from the hospital and claims data described elsewhere. We excluded all outpatient data, as they were implausibly low when compared with inpatient data from the same locations and with claims data. We also excluded inpatient hospital data from countries (Canada, Mexico, Brazil) where the data were implausibly low when compared with other data in the region or super-region.

We included study-level covariates for inpatient hospital data and claims data from 2000 and 2010 to adjust these data points, using as reference the claims data from 2012.

*Severity splits and disability weights*

| Severity level | Lay description | DISABILITY WEIGHT (95% CI) |
| --- | --- | --- |
| Acute myocarditis | Has a fever and aches, and feels weak, which causes some difficulty with daily activities. | 0.051 (0.032-0.074) |

Modeling strategy

We used a DisMod MR-2.1 model, which included the following prior settings:

- 1) Setting a minimum of 3 and maximum of 5 on remission to establish an average duration of 3 months; and, 2) Setting excess mortality to 0 for all ages.

For GBD 2015 estimation, we included study-level covariates for inpatient hospital data and claims data from 2000 and 2010 to adjust these data points, using as reference the claims data from 2012. Other than these shifts, we have made no substantive changes in the modeling strategy from GBD 2013.

| Study covariate | Parameter | beta | Exponentiated beta |
| --- | --- | --- | --- |
| Hospital data | incidence | -1.716(-1.797 to -1.644) | .1798(.1658 to .1932) |
| All MarketScan, year 2000 | incidence | .2966(.2528 to .3362) | 1.345(1.288 to 1.4) |
| All MarketScan, year 2010 | incidence | .1523(.1139 to .1933) | 1.164(1.121 to 1.213) |
| Log-transformed age-standardized SEV scalar: CMP | incidence | .695(.5119 to 1.045) | 2.004(1.668 to 2.843) |

No other significant changes were made to the modeling approach for GBD 2015.

## Atrial Fibrillation and Flutter

Case definition

Atrial fibrillation was defined as a diagnosis with atrial fibrillation or atrial flutter by ECG findings.

Input data

*Model inputs*

A systematic review was performed for GBD 2015 with the following search terms:

(“atrial fibrillation” AND epidemiology[MeSH Subheading]) OR (“atrial flutter” AND epidemiology[MeSH Subheading]) OR (“atrial fibrillation” AND (prevalence OR incidence OR “case fatality”)) OR (“atrial flutter” AND (prevalence OR incidence OR “case fatality”)) OR (“heart atrium fibrillation” AND epidemiology[MeSH Subheading]) OR (“heart atrium fibrillation” AND (prevalence OR incidence OR “case fatality”))

The dates of the search were 1/1/2013 – 3/15/2016. There were 5,630 studies returned and, of those, 27 were extracted.

A systematic review was also performed for GBD 2013 and the search terms were:

Search terms: (hasabstract[text] AND Humans[Mesh] AND middle age[MeSH])) OR 21) AND ((atrial fibrillation/epidemiology[Mesh] OR atrial fibrillation/mortality[Mesh]) AND (prevalence[Title/Abstract] OR incidence[Title/Abstract]) AND ("2010"[Date - Publication] : "3000"[Date - Publication]) AND (hasabstract[text] AND Humans[Mesh] AND middle age[MeSH]))

The table below shows the data inputs:

|  | Prevalence | Incidence | Mortality risk |
| --- | --- | --- | --- |
| Studies | 71 | 24 | 15 |
| Countries/subnationals | 42 | 17 | 12 |
| GBD world regions | 8 | 3 | 6 |

Apart from hospital and claims data points on prevalence, no non-literature-based data were included. We excluded hospital data in certain geographies where the data were implausibly low for all years in both sexes (e.g., Canada, Mexico). We included study-level covariates to crosswalk the inpatient hospital data and the claims data from 2000 and 2010, using as reference literature data and the claims data from 2012.

*Severity splits & disability weights*

Atrial fibrillation is split into symptomatic and asymptomatic based on standard GBD proportion information. The table below includes lay descriptions and disability weights for the severity levels of atrial fibrillation:

| Severity level | Lay description | DISABILITY WEIGHT (95% CI) |
| --- | --- | --- |
| Asymptomatic | No symptoms | N/A |
| Symptomatic | Has periods of rapid and irregular heartbeats and occasional fainting | 0.224 (0.151-0.312) |

Modeling strategy

In order to address changes in coding practices for atrial fibrillation, we used an integrated approach that combined DisMod-MR and CODEm models to generate estimates for atrial fibrillation and flutter. This new approach, a major change from GBD 2013, allowed us to adjust estimates to more accurately reflect the number of deaths for which atrial fibrillation was the true underlying cause of death, thus generating better estimates of prevalence.

- In Step 1, we estimated deaths for atrial fibrillation using a standard CODEm approach.
- In Step 2, we estimated prevalence rates in DisMod-MR using data from published reports of cross-sectional and cohort surveys and primary care facility data. We also used claims data covering inpatient and outpatient visits for the United States along with inpatient hospital data from 22 countries. As the inpatient hospital data only included information from the primary code for each visit, prevalence rates for these data were adjusted based on the age- and sex-specific proportions of atrial fibrillation in the primary codes vs. secondary codes in the US claims data.
- In Step 3, we calculated the excess mortality rate (EMR) for 2015 (defined as the cause-specific mortality rate (CSMR) estimated from CODEm divided by the prevalence rate from DisMod-MR). We then selected 27 countries based on four conditions: 1) availability of VR data; 2) prevalence rate ≥ 0.005; 3) CSMR ≥ 0.00002; and, 4) EMR ≥ 0.001. Using information from these countries as input data, we ran a linear mixed-effects regression of logEMR on sex, age, and location. Sex and age were treated as fixed effects for the regression, while location was considered a random effect. We then predicted age- and sex-specific EMR using the results of this regression for all non-selected countries. Countries included in the regression were assigned their directly calculated values. These EMR data points were assigned to the time period 1990-2015 and uploaded into the Nonfatal database.
- In Step 4, we re-ran DisMod-MR including the EMR estimated in Step 3 and using log-transformed lagged distributed income (LDI) as a country-level covariate. Based on information from other regressions, we set the bounds at -1.5 to -0.25. We also included study-level covariates to cross-walk the inpatient hospital and claims data from 2000 and 2010 to the reference data, which included literature data and claims data from 2012. We included a value prior of 0 for remission for all ages. We also set a value prior of 0 for excess mortality for ages 0-30.
- In Steps 5 and 6, we repeated the process in Steps 3 and 4. In this iteration, we selected 31 countries that were included in the mixed-effects regression. The criteria were: 1) availability of VR data; 2) prevalence rate ≥ 0.004; 3) CSMR ≥ 0.00002; and, 4) EMR ≥ 0.002.

The prevalence from the DisMod-MR model in Step 6 was used as the finalized output for upload to COMO and further processing into YLDs and DALYs. Models were evaluated based on expert opinion, comparison with results from previous rounds of GBD, and model fit.

The table below includes the study covariates, parameters, betas, and exponentiated betas.

| Study covariate | Parameter | Beta | Exponentiated beta |
| --- | --- | --- | --- |
| Hospital data | prevalence | -.9192(-.9199 to -.9175) | .3988(.3986 to .3995) |
| All MarketScan, year 2000 | prevalence | -.2752(-.3101 to -.2468) | .7594(.7334 to .7813) |
| All MarketScan, year 2010 | prevalence | .14(.1105 to .1658) | 1.15(1.117 to 1.18) |
| LDI (I$ per capita) | excess mortality rate | -.25(-.2501 to -.25) | .7788(.7787 to .7788) |

No other significant changes were made to modeling strategy for GBD 2015.

## Peripheral Artery Disease

Case definition

For GBD 2015, PAD was defined as having an ankle-brachial index (ABI) <0.9. Intermittent claudication was defined clinically.

**Input data**

*Model inputs*

A systematic review was performed for peripheral vascular disease and intermittent claudication for GBD 2015. The search terms used are presented below:

('peripheral vascular disease'[TIAB] AND 'epidemiology'[Subheading]) OR ('peripheral arterial disease'[TIAB] AND 'epidemiology'[Subheading]) OR ('peripheral artery disease'[TIAB] AND 'epidemiology'[Subheading]) OR ('intermittent claudication'[TIAB] AND 'epidemiology'[Subheading]) OR ('ankle-brachial index'[TIAB] AND 'epidemiology'[Subheading]) OR ('ankle brachial index'[TIAB] AND 'epidemiology'[Subheading]) OR ('peripheral artery occlusive disease'[TIAB] AND 'epidemiology'[Subheading]) OR ('peripheral obliterative arteriopathy'[TIAB] AND 'epidemiology'[Subheading]) OR ('peripheral vascular disease'[TIAB] AND 'prevalence'[MeSH Terms]) OR ('peripheral vascular disease'[TIAB] AND 'incidence'[MeSH Terms]) OR ('peripheral vascular disease'[TIAB] AND 'case fatality'[All Fields]) OR ('symptomatic claudication'[TIAB] AND (proportion[All Fields] OR percent[All Fields]))

The search was conducted from 1/1/13 – 3/16/2015, and the number of studies returned was 1,658, of which six were extracted.

A systematic review was also performed for peripheral artery disease and intermittent claudication for GBD 2013.

The table below shows the number of literature studies included in GBD 2015, as well as the number of countries or subnational units and GBD world regions represented.

Peripheral artery disease

|  | Prevalence | Incidence | Mortality risk |
| --- | --- | --- | --- |
| Studies | 23 | 3 | 1 |
| Countries/subnationals | 18 | 2 | 1 |
| GBD world regions | 11 | 2 | 1 |

Proportion with intermittent claudication

|  | Proportion |
| --- | --- |
| Studies | 10 |
| Countries/subnationals | 6 |
| GBD world regions | 4 |

Apart from the hospital and claims data, we did not include any non-literature-based data types. We excluded hospital data that were implausibly low for all years and both sexes in certain geographies (e.g., Canada, Mexico, Brazil). We included study-level covariates for inpatient hospital data and claims data, using literature data as the reference.

*Severity splits and disability weights*

We used the proportion of intermittent claudication to split the overall prevalence of peripheral artery disease into symptomatic and asymptomatic peripheral artery disease. The table below illustrates these values:

| Severity level | Lay description | DISABILITY WEIGHT (95% CI) |
| --- | --- | --- |
| Asymptomatic | No symptoms | N/A |
| Symptomatic | Has cramping pains in the legs after walking a medium distance. The pain goes away after a short rest. | 0.014 (0.007-0.025) |

Modeling strategy

For GBD 2015, we used DisMod to model the overall prevalence of peripheral artery disease using prevalence data from literature studies, inpatient hospital data, and claims data.

For this model, we included study-level covariates on hospital data and claims data to adjust the data points, using the literature as the reference data. We also included the log-transformed, age-standardized SEV scalar for PVD as a fixed-effect, country-level covariate. We set value priors of 0 for incidence from ages 0 to 30. We also set a value prior of 0 for remission for all ages. Finally, we set a value prior of a maximum value of 0.25 on excess mortality for all ages.

The table below illustrates the study covariates, parameters, beta, and exponentiated beta values for the overall peripheral artery disease model.

| Study covariate | Parameter | beta | Exponentiated beta |
| --- | --- | --- | --- |
| Hospital data | prevalence | -1.998(-2 - -1.993) | .1356(.1353 - .1363) |
| All MarketScan, year 2000 | prevalence | -.7449(-.7498 - -.733) | .4748(.4725 - .4805) |
| All MarketScan, year 2010 | prevalence | -.371(-.3739 - -.37) | .69(.688 - .6907) |
| All MarketScan, year 2012 | prevalence | -.3016(-.3065 - -.3) | .7396(.736 - .7408) |
| Log-transformed age-standardized SEV scalar: PAD | prevalence | .7985(.7506 - .9187) | 2.222(2.118 - 2.506) |

We used DisMod MR-2.1 to model the proportion of peripheral artery disease with intermittent claudication. We set a value prior of 0 for proportion for ages 0 to 40.

To obtain final estimates for asymptomatic and symptomatic peripheral artery disease, we multiplied the prevalence model by the proportion model at the draw level to generate the prevalence of symptomatic and asymptomatic peripheral artery disease.

Models were evaluated based on expert review, comparisons with estimates from prior rounds of GBD, and assessing model fit.

Apart from using hospital and claims data in the overall prevalence model, there have been no substantive changes from GBD 2013 in terms of modeling strategy.

## Acute Endocarditis

Case definition

Our case definition for acute endocarditis was a clinical diagnosis of infective endocarditis.

Input data

*Model inputs*

Prior to the GBD 2015 estimation process, a systematic review was performed for GBD 2013 and subsequently updated in anticipation of GBD 2015. The following search terms were used:

Search terms: ((‘endocarditis’[MeSH Terms] OR ‘endocarditis’[All Fields]) AND ‘epidemiology’[Subheading]) OR ((‘endocarditis’[MeSH Terms] OR ‘endocarditis’[All Fields]) AND ((‘epidemiology’[Subheading] OR ‘epidemiology’[All Fields] OR ‘incidence’[All Fields] OR ‘incidence’[MeSH Terms]) OR (‘epidemiology’[Subheading] OR ‘epidemiology’[All Fields] OR ‘prevalence’[All Fields] OR ‘prevalence’[MeSH Terms]) OR ‘case fatality’[All Fields])) OR ((‘endocardium’[MeSH Terms] OR ‘endocardium’[All Fields]) AND inflammation[TIAB] AND ‘epidemiology’[Subheading]) OR ((‘endocardium’[MeSH Terms] OR ‘endocardium’[All Fields]) AND inflammation[TIAB] AND ((‘epidemiology’[Subheading] OR ‘epidemiology’[All Fields] OR ‘incidence’[All Fields] OR ‘incidence’[MeSH Terms]) OR (‘epidemiology’[Subheading] OR ‘epidemiology’[All Fields] OR ‘prevalence’[All Fields] OR ‘prevalence’[MeSH Terms]) OR ‘case fatality’[All Fields]))

- - - Dates included in search: 1/1/2013 – 3/16/2015
    - Number of initial hits: 1,246
    - Number of sources included: 6

The table below shows the number of literature studies included in GBD 2015, as well as the number of countries or subnational units and GBD world regions represented.

|  | Prevalence | Incidence | Mortality risk |
| --- | --- | --- | --- |
| Studies | 0 | 14 | 1 |
| Countries/subnationals | 0 | 7 | 1 |
| GBD world regions | 0 | 3 | 1 |

We did not include any non-literature-based data types, apart from the hospital and claims data described elsewhere. We excluded all outpatient data, as they were implausibly low when compared with inpatient data from the same locations and claims data. We also excluded inpatient hospital data from countries (Canada, Mexico, Brazil) where the data were implausibly low when compared with other data in the region or super-region.

We included study-level covariates for inpatient hospital data and claims data from 2000 and 2010 to adjust these data points, using as reference the data obtained from literature and claims data from 2012.

*Severity split inputs*

We used the standard GBD approach, which utilizes MEPS data to split overall estimates of endocarditis into moderate and severe categories. The table below includes the severity level, lay descriptions and DISABILITY WEIGHTs associated with acute endocarditis.

| Severity level | Lay description | DISABILITY WEIGHT (95% CI) |
| --- | --- | --- |
| Moderate | Has a fever and aches, and feels weak, which causes some difficulty with daily activities. | 0.051 (0.032-0.074) |
| Severe | Has a high fever and pain, and feels very weak, which causes great difficulty with daily activities. | 0.133 (0.088-0.19) |

Modeling strategy

For GBD 2015, we estimated acute endocarditis using a DisMod MR-2.1 Bayesian meta-regression model. We included a value prior of 0-20 for ages 0-100 to override the internal defaults of DisMod and allow the model to fit using the input remission data. We included study-level covariates on incidence for inpatient hospital data and claims data from 2000 and 2010.

For GBD 2010, we calculated data for remission from a retrospective cohort study conducted by Landman et al in the Netherlands. The recommended minimum treatment period for IEC is two weeks, which is not always met according to this study. Duration for treated patients, therefore, is more likely about three weeks instead of two because of inconsistent compliance. We then calculated remission with uncertainty, taking into account the mortality rate of patients in the study and an average duration for survivors of three weeks.

All means and uncertainty intervals were calculated by DisMod-MR 2.1. We evaluated models by comparing model fits with the data and with results from previous GBD estimation cycles. Apart from correcting an error in the specification of the remission parameter, we have made no substantive changes in the modeling strategy from GBD 2013.

The table below illustrates the applicable study covariates, parameters, betas, and exponentiated betas.

| Study covariate | Parameter | Beta | Exponentiated beta |
| --- | --- | --- | --- |
| Inpatient hospital data | incidence | .4484(.1224 to .5861) | 1.566(1.13 to 1.797) |
| Claims data 2000 | incidence | -.0127(-1.913 to 2) | .9874(.1476 to 7.389) |
| Claims data 2010 | incidence | .002(-1.964 to 1.952) | 1.002(.1403 to 7.041) |
| Log LDI | incidence | .5018(.5 to .5073) | 1.652(1.649 to 1.661) |
| Log-transformed age-standardized SEV scalar endocarditis | incidence | -.2074(-.3416 to -.1001) | .8127(.7106 to .9047) |

No other significant changes were made to the modeling strategy in 2015.

## Other Cardiovascular Diseases

This residual category was modeled by calculating the ratio of the prevalence of ICD-9 coded other cardiovascular diseases in MEPS and 2005 USA outpatient data to the prevalence of heart failure due to other cardiovascular diseases (estimated as part of the heart failure envelope). This ratio was then applied to the estimates of prevalence of heart failure due to other cardiovascular diseases for all other locations and years to generate location-, age-, sex-, and year-specific estimates for other cardiovascular diseases.

# References

1. GBD Mortality and Causes of Death Collaborators. Global, regional, and national life expectancy, all-cause and cause-specific mortality for 249 causes of death, 1980–2015: a systematic analysis for the Global Burden of Disease Study 2015. *The Lancet* 2016; 388:1459–1544.
2. GBD 2015 Disease and Injury Incidence and Prevalence Collaborators. Global, regional, and national incidence, prevalence, and years lived with disability for 310 diseases and injuries, 1990-2015: a systematic analysis for the Global Burden of Disease Study 2015. *The Lancet* 2016; 388:1603–1658
3. Ryan M Ahern, Rafael Lozano, Mohsen Naghavi, Kyle Foreman, Emmanuela Gakidou, and Christopher JL Murray. “Improving the Public Health Utility of Global Cardiovascular Mortality Data: The Rise of Ischemic Heart Disease.” Population Health Metrics 9, no. 1 (March 15, 2011): 8. doi:10.1186/1478-7954-9-8
4. Foreman KJ, Lozano R, Lopez AD, Murray CJ. Modeling causes of death: an integrated approach using CODEm. Population Health Metrics 2012; 10: 1.
5. Reményi, B. et al. Nat. Rev. Cardiol. 9, 297–309 (2012); published online 28 February 2012
6. Third Universal Definition of Myocardial Infarction

Kristian Thygesen, Joseph S. Alpert, Allan S. Jaffe, Maarten L. Simoons, Bernard R. Chaitman and Harvey D. White and the Writing Group on behalf of the Joint ESC/ACCF/AHA/WHF Task Force for the Universal Definition of Myocardial Infarction

Circulation. 2012;126:2020-2035, originally published October 15, 2012

1. Rose G, McCartney P, Reid DD. Self-administration of a questionnaire on chest pain and intermittent claudication. Br J Prev Soc Med. 1977 Mar;31(1):42–48. [[PMC free article](https://www.ncbi.nlm.nih.gov/pmc/articles/PMC478990/)] [[PubMed](https://www.ncbi.nlm.nih.gov/pubmed/856370)]
2. Heart. 2012 Nov;98(22):1660-6. doi: 10.1136/heartjnl-2012-302318. Epub 2012 Sep 2. Utility of self-reported diagnosis and electrocardiogram Q-waves for estimating myocardial infarction prevalence: an international comparison study. Moran A1, Shen A, Turner-Lloveras D, Khan A, Clays E, Palmas W, De Bacquer D.
3. Hatano S. Experience from a multicentre stroke register: a preliminary report. Bulletin of the World Health Organisation 1976;54(5):541–553

| **Appendix Table 1. GBD 2015 cause and sequela hierarchy with four levels of causes (1-4) and two levels of sequelae (5-6) for Cardiovascular Diseases** | |
| --- | --- |
| **Causes and sequelae** | **Level** |
| Cardiovascular diseases | 2 |
| Rheumatic heart disease | 3 |
| Rheumatic heart disease, without heart failure | 5 |
| Heart failure due to rheumatic heart disease | 5 |
| Mild heart failure due to rheumatic heart disease | 6 |
| Moderate heart failure due to rheumatic heart disease | 6 |
| Severe heart failure due to rheumatic heart disease | 6 |
| Ischemic heart disease | 3 |
| Myocardial infarction episodes | 5 |
| Acute myocardial infarction first 2 days | 6 |
| Acute myocardial infarction 3 to 28 days | 6 |
| Asymptomatic ischemic heart disease following myocardial infarction | 5 |
| Angina due to ischemic heart disease | 5 |
| Asymptomatic angina due to ischemic heart disease | 6 |
| Mild angina due to ischemic heart disease | 6 |
| Moderate angina due to ischemic heart disease | 6 |
| Severe angina due to ischemic heart disease | 6 |
| Heart failure due to ischemic heart disease | 5 |
| Mild heart failure due to ischemic heart disease | 6 |
| Moderate heart failure due to ischemic heart disease | 6 |
| Severe heart failure due to ischemic heart disease | 6 |
| Cerebrovascular disease | 3 |
| Ischemic stroke | 4 |
| Chronic ischemic stroke | 5 |
| Asymptomatic chronic ischemic stroke | 6 |
| Chronic ischemic stroke severity level 1 | 6 |
| Chronic ischemic stroke severity level 2 | 6 |
| Chronic ischemic stroke severity level 4 | 6 |
| Chronic ischemic stroke severity level 3 | 6 |
| Chronic ischemic stroke severity level 5 | 6 |
| Ischemic stroke episodes | 5 |
| Acute ischemic stroke severity level 1 | 6 |
| Acute ischemic stroke severity level 2 | 6 |
| Acute ischemic stroke severity level 4 | 6 |
| Acute ischemic stroke severity level 3 | 6 |
| Acute ischemic stroke severity level 5 | 6 |
| Hemorrhagic stroke | 4 |
| Chronic hemorrhagic stroke | 5 |
| Asymptomatic chronic hemorrhagic stroke | 6 |
| Chronic hemorrhagic stroke severity level 1 | 6 |
| Chronic hemorrhagic stroke severity level 2 | 6 |
| Chronic hemorrhagic stroke severity level 4 | 6 |
| Chronic hemorrhagic stroke severity level 3 | 6 |
| Chronic hemorrhagic stroke severity level 5 | 6 |
| Acute hemorrhagic stroke | 5 |
| Acute hemorrhagic stroke severity level 1 | 6 |
| Acute hemorrhagic stroke severity level 2 | 6 |
| Acute hemorrhagic stroke severity level 4 | 6 |
| Acute hemorrhagic stroke severity level 3 | 6 |
| Acute hemorrhagic stroke severity level 5 | 6 |
| Hypertensive heart disease | 3 |
| Mild heart failure due to hypertensive heart disease | 5 |
| Moderate heart failure due to hypertensive heart disease | 5 |
| Severe heart failure due to hypertensive heart disease | 5 |
| Cardiomyopathy and myocarditis | 3 |
| Acute myocarditis | 5 |
| Heart failure due to cardiomyopathy | 5 |
| Mild heart failure due to cardiomyopathy and myocarditis | 6 |
| Moderate heart failure due to cardiomyopathy and myocarditis | 6 |
| Severe heart failure due to cardiomyopathy and myocarditis | 6 |
| Atrial fibrillation and flutter | 3 |
| Asymptomatic atrial fibrillation and flutter | 5 |
| Symptomatic atrial fibrillation and flutter | 5 |
| Peripheral vascular disease | 3 |
| Asymptomatic peripheral vascular disease | 5 |
| Symptomatic claudication due to peripheral vascular disease | 5 |
| Endocarditis | 3 |
| Endocarditis episodes | 5 |
| Moderate endocarditis | 6 |
| Severe endocarditis | 6 |
| Heart failure due to endocarditis | 5 |
| Mild heart failure due to endocarditis | 6 |
| Moderate heart failure due to endocarditis | 6 |
| Severe heart failure due to endocarditis | 6 |
| Other cardiovascular and circulatory diseases | 3 |
| Heart failure due to other cardiovascular diseases | 5 |
| Mild heart failure due to other cardiovascular diseases | 6 |
| Moderate heart failure due to other cardiovascular diseases | 6 |
| Severe heart failure due to other cardiovascular diseases | 6 |
| Other cardiovascular diseases episodes | 5 |
| Asymptomatic other cardiovascular diseases | 6 |
| Mild other cardiovascular diseases | 6 |
| Moderate other cardiovascular diseases | 6 |
| Severe other cardiovascular diseases | 6 |

| **Appendix Table 2: Causes of death source list by geography, source type, and study site, with years, age range, and sample size** | | | | | |
| --- | --- | --- | --- | --- | --- |
| **Location** | **Source name** | **Source type** | **Years** | **Ages** | **Sample size** |
| Afghanistan | Afghanistan Special Demographic And Health Survey | Verbal Autopsy | 2008 | All Ages | 9,210 |
| Albania | Albania Vital Registration Causes Death | Vital Registry | 2010 | All Ages | 1,151 |
| Albania | WHO Mortality Database | Vital Registry | 1987-1989 | All Ages | 222,500 |
| Albania | WHO Mortality Database | Vital Registry | 1992-2009 | All Ages | 241,824 |
| American Samoa | United States National Vital Statistics System (NVSS) | Vital Registry | 2005-2012 | All Ages | 3,381 |
| American Samoa | United States Nvss Mortality Data | Vital Registry | 1997-2004 | All Ages | 3,085 |
| Antigua and Barbuda | WHO Mortality Database | Vital Registry | 2000-2009 | All Ages | 2,121 |
| Antigua and Barbuda | WHO Mortality Database | Vital Registry | 1983 | All Ages | 3,120 |
| Antigua and Barbuda | WHO Mortality Database | Vital Registry | 1985-1995 | All Ages | 3,989 |
| Antigua and Barbuda | WHO Mortality Database | Vital Registry | 2012-2013 | All Ages | 5,649 |
| Argentina | WHO Mortality Database | Vital Registry | 1980-2013 | All Ages | 2,573,348 |
| Armenia | WHO Mortality Database | Vital Registry | 1981-1982 | All Ages | 161,673 |
| Armenia | WHO Mortality Database | Vital Registry | 1985-2003 | All Ages | 189,361 |
| Armenia | WHO Mortality Database | Vital Registry | 2006 | All Ages | 188,434 |
| Armenia | WHO Mortality Database | Vital Registry | 2008-2012 | All Ages | 190,211 |
| Australia | Australia Vital Registration - Deaths | Vital Registry | 2005 | All Ages | 2,005,221 |
| Australia | WHO Mortality Database | Vital Registry | 1980-2004 | All Ages | 1,660,954 |
| Australia | WHO Mortality Database | Vital Registry | 2006-2011 | All Ages | 2,108,770 |
| Austria | WHO Mortality Database | Vital Registry | 1980-2014 | All Ages | 1,451,840 |
| Azerbaijan | WHO Mortality Database | Vital Registry | 1981-1982 | All Ages | 399,415 |
| Azerbaijan | WHO Mortality Database | Vital Registry | 1985-2000 | All Ages | 422,892 |
| Azerbaijan | WHO Mortality Database | Vital Registry | 2001-2004 | All Ages | 312,147 |
| Azerbaijan | WHO Mortality Database | Vital Registry | 2007 | All Ages | 776,127 |
| Bahrain | Bahrain Vital Registration - Deaths | Vital Registry | 1986 | All Ages | 37,799 |
| Bahrain | WHO Mortality Database | Vital Registry | 2000-2013 | All Ages | 29,676 |
| Bahrain | WHO Mortality Database | Vital Registry | 1998 | All Ages | 84,372 |
| Bangladesh | Demographic Surveillance System (DSS) | Verbal Autopsy | 2000 | All Ages | 1,987 |
| Bangladesh | Bangladesh Chandpur And Comilla District Verbal Autopsy Study | Verbal Autopsy | 2011-2013 | All Ages | 6,797 |
| Bangladesh | Bangladesh Matlab Health And Demographic Surveillance System | Verbal Autopsy | 1980-2010 | 20 - 70 | 301 |
| Bangladesh | Beyond Pregnancy Neglected Burden Mortality Young Women Reproductive Age Bangladesh | Verbal Autopsy | 2004 | 15 - 45 | 1,107 |
| Barbados | WHO Mortality Database | Vital Registry | 1980-1995 | All Ages | 19,750 |
| Barbados | WHO Mortality Database | Vital Registry | 2000-2012 | All Ages | 26,743 |
| Belarus | WHO Mortality Database | Vital Registry | 1981-1982 | All Ages | 753,418 |
| Belarus | WHO Mortality Database | Vital Registry | 1985-1995 | All Ages | 864,214 |
| Belarus | WHO Mortality Database | Vital Registry | 1997-2001 | All Ages | 1,360,872 |
| Belarus | WHO Mortality Database | Vital Registry | 2002-2003 | All Ages | 731,042 |
| Belarus | WHO Mortality Database | Vital Registry | 2007-2009 | All Ages | 926,397 |
| Belarus | WHO Mortality Database | Vital Registry | 2011 | All Ages | 940,557 |
| Belgium | WHO Mortality Database | Vital Registry | 1980-2012 | All Ages | 1,792,995 |
| Belize | WHO Mortality Database | Vital Registry | 1989-1991 | All Ages | 6,890 |
| Belize | WHO Mortality Database | Vital Registry | 1986-1987 | All Ages | 7,397 |
| Belize | WHO Mortality Database | Vital Registry | 1980-1984 | All Ages | 7,551 |
| Belize | WHO Mortality Database | Vital Registry | 1993-2013 | All Ages | 7,719 |
| Bermuda | WHO Mortality Database | Vital Registry | 1983-2013 | All Ages | 641 |
| Bermuda | WHO Mortality Database | Vital Registry | 1980 | All Ages | 2,550 |
| Bolivia | WHO Mortality Database | Vital Registry | 2000-2003 | All Ages | 313,199 |
| Bosnia and Herzegovina | WHO Mortality Database | Vital Registry | 1985-1991 | All Ages | 275,776 |
| Bosnia and Herzegovina | WHO Mortality Database | Vital Registry | 2011 | All Ages | 522,053 |
| Brazil | Brazil Mortality Information System (SIM) | Vital Registry | 1980-2013 | All Ages | 9,226,957 |
| Brazil | Brazil Mortality Information System (SIM) | Vital Registry | 1980-2009 | All Ages | 369,243 |
| Brazil | Early childhood mortality in a Brazilian cohort: the roles of birthweight and socioeconomic status | Verbal Autopsy | 1984 | 42740 | 29 |
| Brunei | WHO Mortality Database | Vital Registry | 1996-2013 | All Ages | 6,832 |
| Bulgaria | WHO Mortality Database | Vital Registry | 1980-2012 | All Ages | 971,504 |
| Burkina Faso | Demographic Surveillance System (DSS) | Verbal Autopsy | 2000 | All Ages | 9,489 |
| Burkina Faso | Causas de la distribución de muerte con el modelo InterVA y la codificación de médicos en un área rural de Burkina Faso | Verbal Autopsy | 2002 | All Ages | 16,167 |
| Burkina Faso | The epidemiology of pregnancy outcomes in rural Burkina Faso | Verbal Autopsy | 2004 | 15 - 50 | 828 |
| Burkina Faso | An improved method for physician-certified verbal autopsy reduces the rate of discrepancy: experiences in the Nouna Health and Demographic Surveillance Site (NHDSS), Burkina Faso | Verbal Autopsy | 2009 | All Ages | 3,685 |
| Burkina Faso | The Kaya HDSS, Burkina Faso: a platform for epidemiological studies and health programme evaluation | Verbal Autopsy | 2009 | 0 - 15 | 550 |
| Burkina Faso | Measuring the local burden of disease. A study of years of life lost in sub-Saharan Africa | Verbal Autopsy | 1998 | All Ages | 5,171 |
| Canada | WHO Mortality Database | Vital Registry | 1980-2011 | All Ages | 1,700,743 |
| Cape Verde | Deaths among women of reproductive age in Cape Verde: causes and avoidability | Verbal Autopsy | 1992 | 15 - 50 | 91 |
| Cape Verde | WHO Mortality Database | Vital Registry | 1980 | All Ages | 20,936 |
| Cape Verde | WHO Mortality Database | Vital Registry | 2011-2012 | All Ages | 35,762 |
| Chile | Chile Vital Registration - Deaths | Vital Registry | 1985 | All Ages | 1,192,583 |
| Chile | WHO Mortality Database | Vital Registry | 1986-2013 | All Ages | 687,842 |
| Chile | WHO Mortality Database | Vital Registry | 1980-1984 | All Ages | 847,942 |
| China | China National Disease Surveillance Points (DSP) | Vital Registry | 2004-2007 | All Ages | 5,672,065 |
| China | China National Disease Surveillance Points (DSP) | Vital Registry | 2004-2007 | All Ages | 805,423 |
| China | China National Disease Surveillance Points And Information System Disease Control And Prevention | Vital Registry | 2013-2014 | All Ages | 62,651,689 |
| China | China National Disease Surveillance Points And Information System Disease Control And Prevention | Vital Registry | 2013-2014 | All Ages | 9,474,980 |
| China | China Mortality Registration And Reporting System | Vital Registry | 2008-2012 | All Ages | 29,130,742 |
| China | China Mortality Registration And Reporting System | Vital Registry | 2008-2010 | All Ages | 3,432,724 |
| China | China Mortality Registration And Reporting System | Vital Registry | 2012 | All Ages | 6,595,716 |
| China | WHO Mortality Database | Vital Registry | 1980-2013 | All Ages | 256,070 |
| Colombia | Colombia Vital Registration 1980-2005 | Vital Registry | 2003 | All Ages | 2,758,778 |
| Colombia | Colombia Vital Statistics - Deaths | Vital Registry | 1980-1996 | All Ages | 1,393,897 |
| Colombia | Colombia Vital Statistics - Deaths | Vital Registry | 2008 | All Ages | 2,872,601 |
| Colombia | WHO Mortality Database | Vital Registry | 1997-2002 | All Ages | 2,288,665 |
| Colombia | WHO Mortality Database | Vital Registry | 2004-2006 | All Ages | 2,715,547 |
| Colombia | WHO Mortality Database | Vital Registry | 2009-2012 | All Ages | 2,885,011 |
| Costa Rica | WHO Mortality Database | Vital Registry | 1980-2013 | All Ages | 77,359 |
| Croatia | WHO Mortality Database | Vital Registry | 1985-2013 | All Ages | 814,916 |
| Cuba | WHO Mortality Database | Vital Registry | 1980-2013 | All Ages | 548,107 |
| Cyprus | WHO Mortality Database | Vital Registry | 1999-2000 | All Ages | 35,339 |
| Cyprus | WHO Mortality Database | Vital Registry | 2004-2012 | All Ages | 82,570 |
| Czech Republic | WHO Mortality Database | Vital Registry | 1986-2013 | All Ages | 2,062,700 |
| Denmark | WHO Mortality Database | Vital Registry | 1980-1993 | All Ages | 546,639 |
| Denmark | WHO Mortality Database | Vital Registry | 1994-2012 | All Ages | 963,908 |
| Dominica | WHO Mortality Database | Vital Registry | 1980-2013 | All Ages | 3,759 |
| Dominican Republic | WHO Mortality Database | Vital Registry | 1994-2001 | All Ages | 186,052 |
| Dominican Republic | WHO Mortality Database | Vital Registry | 1980-1992 | All Ages | 231,912 |
| Dominican Republic | WHO Mortality Database | Vital Registry | 2003-2012 | All Ages | 367,758 |
| Ecuador | WHO Mortality Database | Vital Registry | 1980-2013 | All Ages | 528,976 |
| Egypt | A comparison of two cause-of-death classification systems for deaths among women of reproductive age in Menoufia, Egypt | Verbal Autopsy | 1981 | 15 - 50 | 2,005 |
| Egypt | WHO Mortality Database | Vital Registry | 1980 | All Ages | 1,148,206 |
| Egypt | WHO Mortality Database | Vital Registry | 1991-1992 | All Ages | 1,154,150 |
| Egypt | WHO Mortality Database | Vital Registry | 1987 | All Ages | 1,364,280 |
| Egypt | WHO Mortality Database | Vital Registry | 2000-2013 | All Ages | 5,450,342 |
| El Salvador | WHO Mortality Database | Vital Registry | 1990-1993 | All Ages | 260,651 |
| El Salvador | WHO Mortality Database | Vital Registry | 1981-1984 | All Ages | 276,451 |
| El Salvador | WHO Mortality Database | Vital Registry | 1995-2012 | All Ages | 280,528 |
| England | United Kingdom England And Wales Mortality Data | Vital Registry | 1981-2012 | All Ages | 8,252,077 |
| England | United Kingdom England And Wales Mortality Statistics | Vital Registry | 2013 | All Ages | 7,514,976 |
| Estonia | WHO Mortality Database | Vital Registry | 1981-1982 | All Ages | 176,585 |
| Estonia | WHO Mortality Database | Vital Registry | 1985-1993 | All Ages | 187,655 |
| Estonia | WHO Mortality Database | Vital Registry | 1994-2012 | All Ages | 326,390 |
| Ethiopia | Emerging chronic non-communicable diseases in rural communities of Northern Ethiopia: evidence using population-based verbal autopsy method in Kilite Awlaelo surveillance site | Verbal Autopsy | 2010 | All Ages | 1,837 |
| Ethiopia | Demographic Surveillance System (DSS) | Verbal Autopsy | 2000 | All Ages | 638 |
| Ethiopia | Factors associated with place of death in Addis Ababa, Ethiopia | Verbal Autopsy | 2008 | 15 + | 4,065 |
| Ethiopia | The use of simplified verbal autopsy in identifying causes of adult death in a predominantly rural population in Ethiopia | Verbal Autopsy | 1997 | 15 - 50 | 459 |
| Fiji | WHO Mortality Database | Vital Registry | 1999 | All Ages | 32,991 |
| Fiji | WHO Mortality Database | Vital Registry | 2001-2009 | All Ages | 85,768 |
| Fiji | WHO Mortality Database | Vital Registry | 2011-2012 | All Ages | 100,421 |
| Finland | WHO Mortality Database | Vital Registry | 1980-1986 | All Ages | 443,466 |
| Finland | WHO Mortality Database | Vital Registry | 1987-2013 | All Ages | 476,474 |
| France | WHO Mortality Database | Vital Registry | 1980-2011 | All Ages | 7,977,563 |
| Georgia | WHO Mortality Database | Vital Registry | 1994-1997 | All Ages | 412,411 |
| Georgia | WHO Mortality Database | Vital Registry | 1981-1982 | All Ages | 431,971 |
| Georgia | WHO Mortality Database | Vital Registry | 1985-1992 | All Ages | 433,145 |
| Georgia | WHO Mortality Database | Vital Registry | 1998-2001 | All Ages | 616,464 |
| Georgia | WHO Mortality Database | Vital Registry | 2009-2014 | All Ages | 682,053 |
| Georgia | WHO Mortality Database | Vital Registry | 2004-2007 | All Ages | 712,866 |
| Germany | WHO Mortality Database | Vital Registry | 1980-2013 | All Ages | 8,241,317 |
| Ghana | Determinants of epidemiologic transition in rural Africa: the role of socioeconomic status and drinking water source | Verbal Autopsy | 2010 | All Ages | 1,146 |
| Ghana | Determinants of epidemiologic transition in rural Africa: the role of socioeconomic status and drinking water source | Verbal Autopsy | 2007 | All Ages | 1,233 |
| Ghana | Determinants of epidemiologic transition in rural Africa: the role of socioeconomic status and drinking water source | Verbal Autopsy | 2004 | All Ages | 1,380 |
| Ghana | Effect of vitamin A supplementation on cause-specific mortality in women of reproductive age in Ghana: a secondary analysis from the ObaapaVitA trial | Verbal Autopsy | 2004 | 15 - 45 | 954 |
| Ghana | Ghana - Accra Births and Deaths Registry - Deaths | Vital Registry | 2007 | All Ages | 91,294 |
| Ghana | Ghana - Accra Births and Deaths Registry - Deaths | Vital Registry | 2000 | All Ages | 267,864 |
| Ghana | Demographic Surveillance System (DSS) | Verbal Autopsy | 2000 | All Ages | 39,664 |
| Ghana | DHS Special Demographic and Health Survey (Special DHS) | Verbal Autopsy | 2007 | 18537 | 6,352 |
| Ghana | How many years of life could be saved if malaria were eliminated from a hyperendemic area of northern Ghana? | Verbal Autopsy | 1995 | 5 + | 2,870 |
| Ghana | Maternal mortality decline in the Kassena-Nankana district of northern Ghana | Verbal Autopsy | 2003 | 18537 | 516 |
| Greece | WHO Mortality Database | Vital Registry | 1980-2012 | All Ages | 856,500 |
| Greenland | Greenland Vital Registration - Deaths | Vital Registry | 1995-2013 | All Ages | 6,725 |
| Grenada | WHO Mortality Database | Vital Registry | 1984-1985 | All Ages | 7,035 |
| Grenada | WHO Mortality Database | Vital Registry | 1988-1996 | All Ages | 7,334 |
| Grenada | WHO Mortality Database | Vital Registry | 2001-2013 | All Ages | 9,153 |
| Guam | United States National Vital Statistics System (NVSS) | Vital Registry | 2005-2012 | All Ages | 6,853 |
| Guam | United States National Vital Statistics System (NVSS) | Vital Registry | 1994-2004 | All Ages | 8,899 |
| Guatemala | WHO Mortality Database | Vital Registry | 1984 | All Ages | 596,653 |
| Guatemala | WHO Mortality Database | Vital Registry | 1986-2013 | All Ages | 613,896 |
| Guatemala | WHO Mortality Database | Vital Registry | 1980-1981 | All Ages | 643,430 |
| Guyana | Guyana Vital Registration - Deaths | Vital Registry | 2000 | All Ages | 56,212 |
| Guyana | WHO Mortality Database | Vital Registry | 1988-1999 | All Ages | 38,443 |
| Guyana | WHO Mortality Database | Vital Registry | 1984 | All Ages | 44,970 |
| Guyana | WHO Mortality Database | Vital Registry | 2001-2011 | All Ages | 62,456 |
| Honduras | WHO Mortality Database | Vital Registry | 1987-1990 | All Ages | 143,948 |
| Honduras | WHO Mortality Database | Vital Registry | 1980-1983 | All Ages | 170,741 |
| Honduras | WHO Mortality Database | Vital Registry | 2008-2013 | All Ages | 63,041 |
| Hungary | WHO Mortality Database | Vital Registry | 1980-2013 | All Ages | 2,267,846 |
| Iceland | WHO Mortality Database | Vital Registry | 1980 | All Ages | 15,128 |
| Iceland | WHO Mortality Database | Vital Registry | 1981-2009 | All Ages | 16,344 |
| India | Adult mortality surveillance by routine health workers using a short verbal autopsy tool in rural north India | Verbal Autopsy | 2008 | 10 + | 510 |
| India | All-cause mortality and cardiovascular mortality in Kerala state of India: results from a 5-year follow-up of 161,942 rural community dwelling adults | Verbal Autopsy | 2004 | 25 + | 4,248 |
| India | Causes of death in rural adult population of North India (2002-2007), using verbal autopsy tool | Verbal Autopsy | 2004 | 15 + | 2,294 |
| India | Changing profile of disease contributing to mortality in a resettlement colony of Delhi | Verbal Autopsy | 1999 | All Ages | 495 |
| India | Chronic diseases now a leading cause of death in rural India – mortality data from the Andhra Pradesh Rural Health Initiative | Verbal Autopsy | 2003 | 0 - 80 | 1,310 |
| India | A community based cross sectional study on feasibility of lay interviewers in ascertaining causes of adult deaths by using verbal autopsy in rural Wardha | Verbal Autopsy | 2005 | 15 + | 202 |
| India | Effects on the estimated cause-specific mortality fraction of providing physician reviewers with different formats of verbal autopsy data | Verbal Autopsy | 2006 | All Ages | 8,442 |
| India | India Sample Registration System (SRS) | Verbal Autopsy | 2005 | All Ages | 385,349 |
| India | India Sample Registration System (SRS) | Verbal Autopsy | 2011 | All Ages | 543,098 |
| India | India Medical Certification of Cause of Death (MCCD) | Vital Registry | 2012-2013 | All Ages | 667,379 |
| India | India Medical Certification of Cause of Death (MCCD) | Vital Registry | 2011 | All Ages | 755,534 |
| India | India Survey of Causes of Death (SCD) | Verbal Autopsy | 2011 | All Ages | 96,679 |
| India | India Medical Certification of Cause of Death (MCCD) | Vital Registry | 1997 | All Ages | 4,483,675 |
| India | India Medical Certification of Cause of Death (MCCD) | Vital Registry | 2005-2006 | All Ages | 7,353,223 |
| India | India Medical Certification of Cause of Death (MCCD) | Vital Registry | 2008-2010 | All Ages | 9,874,058 |
| India | India Medical Certification Cause Death State Level Tabulations | Vital Registry | 1990-1996 | All Ages | 3,788,932 |
| India | India Medical Certification Cause Death State Level Tabulations | Vital Registry | 1998-2004 | All Ages | 5,379,185 |
| India | India Odisha Medical Certification Cause Death Data | Vital Registry | 2009-2013 | All Ages | 378,617 |
| India | India Study Causes Death Verbal Autopsy | Verbal Autopsy | 2003 | All Ages | 9,798 |
| India | India Survey Causes Death | Verbal Autopsy | 1980-1986 | All Ages | 79,419 |
| India | India Vital Statistics | Vital Registry | 1995 | 35 + | 40,119 |
| India | India Vital Statistics | Vital Registry | 1980-1984 | All Ages | 2,188,535 |
| India | India Vital Statistics | Vital Registry | 1986-1987 | All Ages | 3,572,408 |
| India | India Vital Statistics | Vital Registry | 1989-1993 | All Ages | 3,792,940 |
| India | Risk Mortality Among Alcohol Using Adult Males Population Based Cohort Kerala India Profile | Verbal Autopsy | 2004 | 20 + | 1,859 |
| India | Singh's verbal autopsy questionnaire for the assessment of causes of death, social autopsy, tobacco autopsy and dietary autopsy, based on medical records and interview | Verbal Autopsy | 2000 | 25 - 65 | 2,222 |
| India | Study Causes Deaths Among Insured Population North Easternv District Andhra Pradesh | Verbal Autopsy | 2009 | 0 - 45 | 142 |
| India | A study of registration of deaths at primary health centre--with special reference to verbal autopsy method | Verbal Autopsy | 1991 | All Ages | 230 |
| India | Use of verbal autopsy to determine mortality patterns in an urban slum in Kolkata, India | Verbal Autopsy | 2003 | All Ages | 265,395 |
| India | Verbal autopsy of 48 000 adult deaths attributable to medical causes in Chennai (formerly Madras), India | Verbal Autopsy | 1996 | 25 + | 47,200 |
| Indonesia | Indonesia Sample Registration System | Verbal Autopsy | 2010 | All Ages | 117,323 |
| Indonesia | Indonesia Sample Registration System | Verbal Autopsy | 2014 | All Ages | 205,160 |
| Indonesia | Reproductive Mortality in Two Developing Countries | Verbal Autopsy | 1981 | 15 - 50 | 1,215 |
| Iran | Gastroesophageal Reflux Disease and overall and Cause-specific Mortality: A Prospective Study of 50000 Individuals | Verbal Autopsy | 2008 | 40 - 45 | 3,107 |
| Iran | Iran Vital Registration - Deaths | Vital Registry | 1998-2001 | All Ages | 1,734,878 |
| Iran | Iran Vital Registration - Deaths | Vital Registry | 2007-2011 | All Ages | 2,666,206 |
| Iran | Iran Vital Registration - Deaths | Vital Registry | 1996-1997 | All Ages | 1,745,048 |
| Iran | Iran Death Registration System | Vital Registry | 2002-2006 | All Ages | 2,738,405 |
| Iraq | WHO Mortality Database | Vital Registry | 2008 | All Ages | 1,274,529 |
| Ireland | WHO Mortality Database | Vital Registry | 1980-2012 | All Ages | 485,601 |
| Israel | WHO Mortality Database | Vital Registry | 1980-2013 | All Ages | 229,559 |
| Italy | WHO Mortality Database | Vital Registry | 1980-2003 | All Ages | 8,120,634 |
| Italy | WHO Mortality Database | Vital Registry | 2006-2012 | All Ages | 8,285,378 |
| Jamaica | WHO Mortality Database | Vital Registry | 1980-1991 | All Ages | 104,487 |
| Jamaica | WHO Mortality Database | Vital Registry | 2000-2006 | All Ages | 213,136 |
| Jamaica | WHO Mortality Database | Vital Registry | 2009-2011 | All Ages | 250,102 |
| Japan | Japan Vital Registration - Deaths | Vital Registry | 1980-2013 | All Ages | 11,199,102 |
| Jordan | Jordan National Maternal Mortality Study 2008 | Verbal Autopsy | 2007 | 15 - 50 | 1,406 |
| Jordan | Jordan Vital Registration - Deaths | Vital Registry | 2004-2006 | All Ages | 245,764 |
| Jordan | Mortality and causes of death in Jordan 1995-96: assessment by verbal autopsy | Verbal Autopsy | 1995 | 0 - 80 | 947 |
| Jordan | WHO Mortality Database | Vital Registry | 2008-2011 | All Ages | 203,316 |
| Kazakhstan | WHO Mortality Database | Vital Registry | 1981-1982 | All Ages | 1,043,824 |
| Kazakhstan | WHO Mortality Database | Vital Registry | 1985-1990 | All Ages | 1,098,559 |
| Kazakhstan | WHO Mortality Database | Vital Registry | 2008-2012 | All Ages | 1,039,814 |
| Kazakhstan | WHO Mortality Database | Vital Registry | 1991-2006 | All Ages | 1,175,983 |
| Kenya | Causes of deaths using verbal autopsy among adolescents and adults in rural western Kenya | Verbal Autopsy | 2003 | 29495 | 1,240 |
| Kenya | Kenya KEMRI/CDC Health and Demographic Surveillance System | Verbal Autopsy | 2002 | 0 - 5 | 923 |
| Kenya | Demographic Surveillance System (DSS) | Verbal Autopsy | 2002-2009 | All Ages | 128 |
| Kiribati | WHO Mortality Database | Vital Registry | 1991-2001 | All Ages | 3,770 |
| Kuwait | WHO Mortality Database | Vital Registry | 1993-2013 | All Ages | 32,419 |
| Kuwait | WHO Mortality Database | Vital Registry | 1980-1987 | All Ages | 44,809 |
| Kyrgyzstan | WHO Mortality Database | Vital Registry | 1981-1982 | All Ages | 274,952 |
| Kyrgyzstan | WHO Mortality Database | Vital Registry | 1985-1999 | All Ages | 297,388 |
| Kyrgyzstan | WHO Mortality Database | Vital Registry | 2000-2013 | All Ages | 500,974 |
| Latvia | WHO Mortality Database | Vital Registry | 1980-1995 | All Ages | 301,251 |
| Latvia | WHO Mortality Database | Vital Registry | 1996-2012 | All Ages | 537,745 |
| Lebanon | Non-communicable disease mortality rates using the verbal autopsy in a cohort of middle aged and older populations in Beirut during wartime, 1983-93 | Verbal Autopsy | 1988 | 50 + | 408 |
| Libya | Libya Vital Statistics | Vital Registry | 2006-2008 | All Ages | 57,489 |
| Lithuania | WHO Mortality Database | Vital Registry | 1981-1982 | All Ages | 333,016 |
| Lithuania | WHO Mortality Database | Vital Registry | 1985-1992 | All Ages | 386,823 |
| Lithuania | WHO Mortality Database | Vital Registry | 1993-2013 | All Ages | 458,187 |
| Luxembourg | WHO Mortality Database | Vital Registry | 1980-2013 | All Ages | 56,482 |
| Macedonia | WHO Mortality Database | Vital Registry | 1991-2010 | All Ages | 223,868 |
| Madagascar | Madagascar - Antananorivo Mortality Report 1984-1995 | Vital Registry | 1984-1995 | All Ages | 33,343 |
| Malawi | Adult mortality and probable cause of death in rural northern Malawi in the era of HIV treatment | Verbal Autopsy | 2004-2008 | 15 + | 104 |
| Malawi | Declining child mortality in northern Malawi despite high rates of infection with HIV | Verbal Autopsy | 2004 | 0 - 15 | 111 |
| Malawi | Rates and causes of death in Chiradzulu District, Malawi, 2008: a key informant study | Verbal Autopsy | 2008 | All Ages | 134 |
| Malaysia | Malaysia - Peninsular Vital Statistics | Vital Registry | 1980-1982 | All Ages | 151,652 |
| Malaysia | Malaysia - Peninsular Vital Statistics - Deaths 1998 | Vital Registry | 1998 | All Ages | 577,723 |
| Malaysia | WHO Mortality Database | Vital Registry | 2000-2008 | All Ages | 747,082 |
| Malaysia | WHO Mortality Database | Vital Registry | 1997 | All Ages | 362,709 |
| Maldives | WHO Mortality Database | Vital Registry | 2000-2005 | All Ages | 7,809 |
| Maldives | WHO Mortality Database | Vital Registry | 2007-2008 | All Ages | 16,769 |
| Maldives | WHO Mortality Database | Vital Registry | 2010-2011 | All Ages | 16,883 |
| Mali | Mali - Twelve Years of Urban Mortality in the Sahel. Levels, Trends, Seasons, and Causes of Mortality in Bamako, 1974-1985 | Vital Registry | 1984 | All Ages | 37,083 |
| Mali | Mali - Twelve Years of Urban Mortality in the Sahel. Levels, Trends, Seasons, and Causes of Mortality in Bamako, 1974-1986 | Vital Registry | 1981 | All Ages | 37,602 |
| Malta | WHO Mortality Database | Vital Registry | 1980-2014 | All Ages | 51,151 |
| Mauritius | WHO Mortality Database | Vital Registry | 1980 | All Ages | 63,885 |
| Mauritius | WHO Mortality Database | Vital Registry | 1981-2014 | All Ages | 55,651 |
| Mexico | Mexico Vital Registration - Deaths | Vital Registry | 1980-2008 | All Ages | 5,259,151 |
| Mexico | Mexico Vital Registration - Deaths | Vital Registry | 2010-2014 | All Ages | 8,244,586 |
| Mexico | Mexico Vital Statistics Deaths | Vital Registry | 2009 | All Ages | 7,802,583 |
| Moldova | WHO Mortality Database | Vital Registry | 1981-1982 | All Ages | 404,483 |
| Moldova | WHO Mortality Database | Vital Registry | 1985-1990 | All Ages | 405,977 |
| Moldova | WHO Mortality Database | Vital Registry | 1991-2013 | All Ages | 452,643 |
| Mongolia | WHO Mortality Database | Vital Registry | 1994 | All Ages | 141,741 |
| Montenegro | WHO Mortality Database | Vital Registry | 2009 | All Ages | 69,858 |
| Montenegro | WHO Mortality Database | Vital Registry | 2000-2004 | All Ages | 84,122 |
| Montenegro | WHO Mortality Database | Vital Registry | 2006 | All Ages | 369,952 |
| Morocco | Morocco Cause of Death Data 2005 | Vital Registry | 2005 | All Ages | 1,815,615 |
| Morocco | WHO Mortality Database | Vital Registry | 2008-2012 | All Ages | 687,918 |
| Mozambique | Mozambique Main Causes of Reported Death Study 2001 | Vital Registry | 2001 | All Ages | 177,459 |
| Mozambique | Demographic Surveillance System (DSS) | Verbal Autopsy | 2002 | All Ages | 5,505 |
| Mozambique | Mozambique National Survey on the Causes of Death 2007-2008 | Verbal Autopsy | 2007 | All Ages | 32,603 |
| Myanmar | Cause of Death Verification Study in Myanmar | Verbal Autopsy | 2007 | All Ages | 1,735 |
| Nepal | Nepal Maternal Mortality and Morbidity Study 2008-2009 | Verbal Autopsy | 2008 | 15 - 50 | 1,497 |
| Netherlands | WHO Mortality Database | Vital Registry | 1980-2013 | All Ages | 1,791,667 |
| New Zealand | New Zealand Mortality Collection | Vital Registry | 1988-2012 | All Ages | 422,739 |
| New Zealand | WHO Mortality Database | Vital Registry | 1980-1987 | All Ages | 413,443 |
| Nicaragua | WHO Mortality Database | Vital Registry | 1988-1994 | All Ages | 118,243 |
| Nicaragua | WHO Mortality Database | Vital Registry | 1996-2013 | All Ages | 130,252 |
| Nigeria | Health & demographic surveillance system profile: the Nahuche Health and Demographic Surveillance System, Northern Nigeria (Nahuche HDSS) | Verbal Autopsy | 2012 | 0 - 20 | 2,100 |
| Nigeria | The Idikan adult mortality study | Verbal Autopsy | 1994 | 15 + | 62 |
| Northern Ireland | WHO Mortality Database | Vital Registry | 1980-2013 | All Ages | 261,636 |
| Northern Mariana Islands | United States National Vital Statistics System (NVSS) | Vital Registry | 2005-2012 | All Ages | 376 |
| Northern Mariana Islands | United States National Vital Statistics System (NVSS) | Vital Registry | 1998-2004 | All Ages | 1,812 |
| Norway | WHO Mortality Database | Vital Registry | 1980-1985 | All Ages | 412,059 |
| Norway | WHO Mortality Database | Vital Registry | 1986-2013 | All Ages | 687,223 |
| Oman | WHO Mortality Database | Vital Registry | 2009-2010 | All Ages | 47,187 |
| Pakistan | To determine the probable causes of death in an urban slum community of Pakistan among adults 18 years and above by verbal autopsy | Verbal Autopsy | 2010 | 15 + | 300 |
| Pakistan | Demographic and Health Surveys Program (DHS) | Verbal Autopsy | 2006 | 0 - 50 | 2,522 |
| Palestine | Palestine - West Bank Vital Registration - Deaths | Vital Registry | 1997-2007 | All Ages | 49,126 |
| Palestine | WHO Mortality Database | Vital Registry | 2008-2009 | All Ages | 92,351 |
| Palestine | WHO Mortality Database | Vital Registry | 2011-2014 | All Ages | 103,502 |
| Panama | WHO Mortality Database | Vital Registry | 2005 | All Ages | 202,480 |
| Panama | WHO Mortality Database | Vital Registry | 1980-1989 | All Ages | 75,574 |
| Panama | WHO Mortality Database | Vital Registry | 1996-2004 | All Ages | 105,997 |
| Panama | WHO Mortality Database | Vital Registry | 2006-2013 | All Ages | 204,469 |
| Papua New Guinea | Mortality in a rural area of Madang Province, Papua New Guinea | Verbal Autopsy | 1983 | All Ages | 407 |
| Paraguay | WHO Mortality Database | Vital Registry | 1994-2013 | All Ages | 150,644 |
| Paraguay | WHO Mortality Database | Vital Registry | 1980-1991 | All Ages | 122,166 |
| Peru | WHO Mortality Database | Vital Registry | 1994-2000 | All Ages | 806,924 |
| Peru | WHO Mortality Database | Vital Registry | 1986-1992 | All Ages | 868,639 |
| Peru | WHO Mortality Database | Vital Registry | 1980-1983 | All Ages | 971,509 |
| Peru | WHO Mortality Database | Vital Registry | 2007-2013 | All Ages | 1,274,469 |
| Philippines | Philippines Vital Registration - Deaths | Vital Registry | 2006-2007 | All Ages | 5,962,922 |
| Philippines | Philippines Vital Registration - Deaths | Vital Registry | 2009-2012 | All Ages | 6,504,826 |
| Philippines | Philippines Vital Statistics - Deaths | Vital Registry | 1980 | All Ages | 2,768,704 |
| Philippines | Philippines Vital Statistics - Deaths | Vital Registry | 1982-1991 | All Ages | 2,889,641 |
| Philippines | Philippines Vital Statistics - Deaths | Vital Registry | 2004-2005 | All Ages | 4,602,110 |
| Philippines | WHO Mortality Database | Vital Registry | 1981 | All Ages | 2,759,590 |
| Philippines | WHO Mortality Database | Vital Registry | 1992-2003 | All Ages | 3,057,294 |
| Philippines | WHO Mortality Database | Vital Registry | 2008 | All Ages | 6,279,299 |
| Poland | WHO Mortality Database | Vital Registry | 1980-1996 | All Ages | 3,073,517 |
| Poland | WHO Mortality Database | Vital Registry | 1999-2013 | All Ages | 5,980,289 |
| Portugal | WHO Mortality Database | Vital Registry | 1980-2003 | All Ages | 835,570 |
| Portugal | WHO Mortality Database | Vital Registry | 2007-2013 | All Ages | 1,635,112 |
| Qatar | Qatar Vital Statistics Annual Bulletin | Vital Registry | 1984-1985 | All Ages | 1,926 |
| Qatar | WHO Mortality Database | Vital Registry | 2006-2012 | All Ages | 24,829 |
| Qatar | WHO Mortality Database | Vital Registry | 1995 | All Ages | 13,735 |
| Qatar | WHO Mortality Database | Vital Registry | 2001 | All Ages | 17,146 |
| Qatar | WHO Mortality Database | Vital Registry | 2004-2005 | All Ages | 19,204 |
| Romania | WHO Mortality Database | Vital Registry | 1980-2012 | All Ages | 2,258,780 |
| Russia | Russia Mortality Rates by Region, Age, Sex, and Cause of Death | Vital Registry | 1989-2000 | All Ages | 17,795,611 |
| Russia | Russia Vital Registration - Deaths | Vital Registry | 2001-2014 | All Ages | 30,342,218 |
| Russia | WHO Mortality Database | Vital Registry | 1980-1988 | All Ages | 11,950,445 |
| Saint Lucia | WHO Mortality Database | Vital Registry | 1983 | All Ages | 4,520 |
| Saint Lucia | WHO Mortality Database | Vital Registry | 1986-2006 | All Ages | 8,158 |
| Saint Lucia | WHO Mortality Database | Vital Registry | 1980-1981 | All Ages | 8,313 |
| Saint Lucia | WHO Mortality Database | Vital Registry | 2008-2012 | All Ages | 15,881 |
| Saint Vincent and the Grenadines | WHO Mortality Database | Vital Registry | 1982-1987 | All Ages | 6,777 |
| Saint Vincent and the Grenadines | WHO Mortality Database | Vital Registry | 1995-2013 | All Ages | 7,243 |
| Saint Vincent and the Grenadines | WHO Mortality Database | Vital Registry | 1990 | All Ages | 9,847 |
| Sao Tome and Principe | WHO Mortality Database | Vital Registry | 1985 | All Ages | 30,819 |
| Saudi Arabia | Saudi Arabia Vital Registration - Deaths | Vital Registry | 2008-2012 | All Ages | 59,266 |
| Saudi Arabia | Saudi Arabia Vital Registration - Deaths | Vital Registry | 1999-2012 | All Ages | 42,638 |
| Scotland | WHO Mortality Database | Vital Registry | 1980-2013 | All Ages | 996,209 |
| Senegal | Adult mortality in a rural area of Senegal: Non-communicable diseases have a large impact in Mlomp | Verbal Autopsy | 1994 | 15 - 60 | 352 |
| Serbia | WHO Mortality Database | Vital Registry | 1998-2013 | All Ages | 1,570,331 |
| Seychelles | WHO Mortality Database | Vital Registry | 1985-1987 | All Ages | 760 |
| Seychelles | WHO Mortality Database | Vital Registry | 2001-2012 | All Ages | 3,751 |
| Seychelles | WHO Mortality Database | Vital Registry | 1981-1982 | All Ages | 4,219 |
| Singapore | WHO Mortality Database | Vital Registry | 1980-2014 | All Ages | 123,242 |
| Slovakia | WHO Mortality Database | Vital Registry | 2012-2014 | All Ages | 830,584 |
| Slovakia | WHO Mortality Database | Vital Registry | 1992-2010 | All Ages | 836,576 |
| Slovenia | WHO Mortality Database | Vital Registry | 1985-2010 | All Ages | 310,291 |
| South Africa | South Africa Mortality and Causes of Death | Vital Registry | 1997-2013 | All Ages | 4,168,084 |
| South Korea | WHO Mortality Database | Vital Registry | 1985-2013 | All Ages | 1,983,981 |
| Spain | WHO Mortality Database | Vital Registry | 1980-2013 | All Ages | 4,505,121 |
| Sri Lanka | Sri Lanka Vital Registration - Deaths | Vital Registry | 2009-2010 | All Ages | 1,734,077 |
| Sri Lanka | Sri Lanka Vital Statistics - Deaths | Vital Registry | 1993-1996 | All Ages | 460,847 |
| Sri Lanka | Sri Lanka Vital Statistics - Deaths | Vital Registry | 2004-2005 | All Ages | 791,020 |
| Sri Lanka | WHO Mortality Database | Vital Registry | 1997-2003 | All Ages | 780,745 |
| Sri Lanka | WHO Mortality Database | Vital Registry | 1991-1992 | All Ages | 839,285 |
| Sri Lanka | WHO Mortality Database | Vital Registry | 1980-1989 | All Ages | 857,774 |
| Sri Lanka | WHO Mortality Database | Vital Registry | 2006 | All Ages | 1,635,528 |
| Suriname | WHO Mortality Database | Vital Registry | 1995-2006 | All Ages | 21,521 |
| Suriname | WHO Mortality Database | Vital Registry | 1984-1992 | All Ages | 21,789 |
| Suriname | WHO Mortality Database | Vital Registry | 1980-1982 | All Ages | 25,565 |
| Suriname | WHO Mortality Database | Vital Registry | 2008-2012 | All Ages | 42,356 |
| Sweden | Sweden Stockholm County Vital Registration - Deaths | Vital Registry | 1990-2011 | All Ages | 253,225 |
| Sweden | Sweden Stockholm County Vital Registration - Deaths | Vital Registry | 1980-1986 | All Ages | 1,047,889 |
| Sweden | Sweden Vital Registration - Deaths | Vital Registry | 1980-1986 | All Ages | 119,753 |
| Sweden | Sweden Vital Registration - Deaths | Vital Registry | 1990-2011 | All Ages | 1,248,973 |
| Sweden | Sweden Vital Registration Neonatal And Child Deaths | Vital Registry | 1987-1989 | 0 - 1 | 587 |
| Sweden except Stockholm | Sweden Stockholm County Vital Registration - Deaths | Vital Registry | 1980-1986 | All Ages | 1,047,889 |
| Sweden except Stockholm | Sweden Vital Registration - Deaths | Vital Registry | 1990-2011 | All Ages | 1,248,973 |
| Sweden except Stockholm | Sweden Vital Registration Neonatal And Child Deaths | Vital Registry | 1987-1989 | 0 - 1 | 464 |
| Switzerland | WHO Mortality Database | Vital Registry | 1980-1994 | All Ages | 575,770 |
| Switzerland | WHO Mortality Database | Vital Registry | 1995-2013 | All Ages | 991,694 |
| Syria | Syria Vital Statistics - Deaths | Vital Registry | 2005-2007 | All Ages | 543,741 |
| Syria | WHO Mortality Database | Vital Registry | 2010 | All Ages | 970,930 |
| Syria | WHO Mortality Database | Vital Registry | 1984-1985 | All Ages | 312,762 |
| Syria | WHO Mortality Database | Vital Registry | 1980 | All Ages | 640,206 |
| Taiwan | Taiwan Annual Statistical Summary of Causes of Death | Vital Registry | 1994 | All Ages | 548,300 |
| Taiwan | Taiwan Statistics of Causes of Death | Vital Registry | 1995-2006 | All Ages | 574,804 |
| Taiwan | Taiwan Statistics of Causes of Death | Vital Registry | 2008-2012 | All Ages | 2,212,792 |
| Taiwan | Taiwan Vital Statistics - Deaths | Vital Registry | 2007 | All Ages | 2,003,716 |
| Tajikistan | WHO Mortality Database | Vital Registry | 1981-1982 | All Ages | 294,266 |
| Tajikistan | WHO Mortality Database | Vital Registry | 1985-2005 | All Ages | 297,935 |
| Tanzania | Muertes de adultos y el futuro: Un análisis causa-específico de las muertes de adultos de un estudio longitudinal en Tanzania rural 2003-2007 | Verbal Autopsy | 2005 | 15 - 30 | 933 |
| Tanzania | Patterns of malaria related mortality based on verbal autopsy in Muleba District, north-western Tanzania | Verbal Autopsy | 2001 | 0 - 70 | 337 |
| Tanzania | The Policy Implications of Tanzania's Mortality Burden | Verbal Autopsy | 2001 | All Ages | 24,807 |
| Tanzania | The Policy Implications of Tanzania's Mortality Burden | Verbal Autopsy | 1995 | All Ages | 30,637 |
| Tanzania | The Policy Implications of Tanzania's Mortality Burden | Verbal Autopsy | 1998 | All Ages | 31,009 |
| Tanzania | Risk Factors and Causes of Adult Deaths in the Ifakara Health and Demographic Surveillance System Population, 2003-2007 | Verbal Autopsy | 2005 | 15 - 60 | 440 |
| Tanzania | Demographic Surveillance System (DSS) | Verbal Autopsy | 2001 | All Ages | 7,404 |
| Tanzania | Demographic Surveillance System (DSS) | Verbal Autopsy | 2000 | All Ages | 16,383 |
| Thailand | Risk factors for a five-year death in the interASIA-south cohort | Verbal Autopsy | 2000 | 35 - 65 | 50 |
| Thailand | Thailand Burden of Disease and Injuries 1998-1999 | Verbal Autopsy | 1997-1998 | All Ages | 41,740 |
| Thailand | Thailand Public Health Statistics | Vital Registry | 2009-2011 | All Ages | 2,655,732 |
| Thailand | Thailand Verbal Autopsy Study | Verbal Autopsy | 2005 | All Ages | 58,296 |
| Thailand | WHO Mortality Database | Vital Registry | 1980-1987 | All Ages | 2,397,464 |
| Thailand | WHO Mortality Database | Vital Registry | 1990-1992 | All Ages | 2,926,209 |
| Thailand | WHO Mortality Database | Vital Registry | 1994-2000 | All Ages | 3,832,483 |
| Thailand | WHO Mortality Database | Vital Registry | 2002-2006 | All Ages | 4,503,245 |
| The Bahamas | WHO Mortality Database | Vital Registry | 1984-1985 | All Ages | 10,992 |
| The Bahamas | WHO Mortality Database | Vital Registry | 1993-2012 | All Ages | 12,557 |
| The Bahamas | WHO Mortality Database | Vital Registry | 1980-1981 | All Ages | 12,646 |
| The Bahamas | WHO Mortality Database | Vital Registry | 1987 | All Ages | 13,093 |
| Tonga | Tonga Vital Statistics - Deaths | Vital Registry | 2003 | All Ages | 19,551 |
| Trinidad and Tobago | WHO Mortality Database | Vital Registry | 1980-2009 | All Ages | 72,965 |
| Tunisia | Tunisia National Statistics on Medical Causes of Death | Vital Registry | 2006 | All Ages | 28,937 |
| Tunisia | WHO Mortality Database | Vital Registry | 2009 | All Ages | 304,678 |
| Tunisia | WHO Mortality Database | Vital Registry | 2013 | All Ages | 324,364 |
| Turkey | Identifying and verifying causes of death in Turkey: National verbal autopsy survey | Verbal Autopsy | 2003 | All Ages | 5,212 |
| Turkey | Prevalence of Asthma, Allergy, and Respiratory Symptoms in Hasançelebi/Hekimhan/Malatya in Eastern Turkey | Verbal Autopsy | 2001 | 20 + | 173 |
| Turkey | Statistical Analysis of Death Causes (2005-2010) in Villages with High Arsenic Levels in Drinking Water Supplies of Simav Plain, Turkey | Verbal Autopsy | 2011 | All Ages | 1,209 |
| Turkey | Turkey National Maternal Mortality Study 2005-2006 | Verbal Autopsy | 2005 | 15 - 50 | 6,876 |
| Turkey | Turkey Verbal Autopsy Survey | Verbal Autopsy | 2002 | All Ages | 5,293 |
| Turkey | WHO Mortality Database | Vital Registry | 2010-2013 | All Ages | 4,255,267 |
| Turkey | WHO Mortality Database | Vital Registry | 2009 | All Ages | 4,312,227 |
| Turkmenistan | WHO Mortality Database | Vital Registry | 1981-1982 | All Ages | 229,399 |
| Turkmenistan | WHO Mortality Database | Vital Registry | 1985-1990 | All Ages | 244,238 |
| Turkmenistan | WHO Mortality Database | Vital Registry | 1994-1998 | All Ages | 302,973 |
| Turkmenistan | WHO Mortality Database | Vital Registry | 2013 | All Ages | 201,374 |
| Turkmenistan | WHO Mortality Database | Vital Registry | 1991-1993 | All Ages | 223,425 |
| Uganda | Uganda Child Verbal Autopsy Study | Verbal Autopsy | 2006 | 0 - 5 | 500 |
| Ukraine | WHO Mortality Database | Vital Registry | 1981-1982 | All Ages | 4,367,109 |
| Ukraine | WHO Mortality Database | Vital Registry | 1985-2004 | All Ages | 4,901,157 |
| Ukraine | WHO Mortality Database | Vital Registry | 2005-2012 | All Ages | 5,398,528 |
| United Arab Emirates | United Arab Emirates Annual Statistical Report | Vital Registry | 2006-2007 | All Ages | 59,707 |
| United Kingdom | United Kingdom - England and Wales Mortality Statistics | Vital Registry | 1981-2012 | All Ages | 9,460,049 |
| United Kingdom | United Kingdom - England and Wales Mortality Statistics | Vital Registry | 2013 | All Ages | 9,022,213 |
| United Kingdom | WHO Mortality Database | Vital Registry | 1980-2013 | All Ages | 1,416,504 |
| United States | United States National Vital Statistics System (NVSS) | Vital Registry | 2005-2013 | All Ages | 37,856,451 |
| United States | United States National Vital Statistics System (NVSS) | Vital Registry | 1980-2004 | All Ages | 30,416,755 |
| United States | WHO Mortality Database | Vital Registry | 1980-1993 | All Ages | 199,032 |
| Uruguay | Uruguay Vital Registration - Deaths | Vital Registry | 1991 | All Ages | 476,827 |
| Uruguay | WHO Mortality Database | Vital Registry | 1980-1990 | All Ages | 348,350 |
| Uruguay | WHO Mortality Database | Vital Registry | 1993-2010 | All Ages | 373,378 |
| Uruguay | WHO Mortality Database | Vital Registry | 2012-2013 | All Ages | 586,428 |
| Uzbekistan | WHO Mortality Database | Vital Registry | 1981-1982 | All Ages | 979,674 |
| Uzbekistan | WHO Mortality Database | Vital Registry | 1985-2003 | All Ages | 1,095,234 |
| Uzbekistan | WHO Mortality Database | Vital Registry | 2004-2005 | All Ages | 1,804,012 |
| Venezuela | WHO Mortality Database | Vital Registry | 1992-1994 | All Ages | 686,704 |
| Venezuela | WHO Mortality Database | Vital Registry | 1980-1983 | All Ages | 715,504 |
| Venezuela | WHO Mortality Database | Vital Registry | 1985-1990 | All Ages | 744,100 |
| Venezuela | WHO Mortality Database | Vital Registry | 1996-2012 | All Ages | 1,392,327 |
| Vietnam | Applying verbal autopsy to determine cause of death in rural Vietnam | Verbal Autopsy | 1999 | All Ages | 568 |
| Vietnam | The causes of deaths in Chililab between 2008-2010 based on verbal autopsy method | Verbal Autopsy | 2008-2010 | All Ages | 901 |
| Vietnam | Maternal mortality in Vietnam in 1994-95 | Verbal Autopsy | 1994 | 15 - 50 | 2,842 |
| Vietnam | Mortality measures from sample-based surveillance: evidence of the epidemiological transition in Viet Nam | Verbal Autopsy | 2008-2009 | All Ages | 44,821 |
| Vietnam | Mortality patterns in Vietnam, 2006: Findings from a national verbal autopsy survey | Verbal Autopsy | 2006 | All Ages | 33,065 |
| Vietnam | Socio-economic status inequality and major causes of death in adults: a 5-year follow-up study in rural Vietnam | Verbal Autopsy | 2001 | 20 + | 1,067 |
| Vietnam | Vietnam Burden of Disease and Injury Study | Verbal Autopsy | 2008 | All Ages | 2,635,153 |
| Virgin Islands, U.S. | United States National Vital Statistics System (NVSS) | Vital Registry | 2005-2012 | All Ages | 5,209 |
| Virgin Islands, U.S. | United States National Vital Statistics System (NVSS) | Vital Registry | 1994-2004 | All Ages | 8,898 |
| Virgin Islands, U.S. | WHO Mortality Database | Vital Registry | 1980 | All Ages | 3,217 |
| Wales | United Kingdom - England and Wales Mortality Statistics | Vital Registry | 1981-2012 | All Ages | 516,422 |
| Wales | United Kingdom - England and Wales Mortality Statistics | Vital Registry | 2013 | All Ages | 500,205 |
| Zambia | Zambia Sample Vital Registration with Verbal Autopsy (SAVVY) Data | Verbal Autopsy | 2009-2010 | All Ages | 1,339 |
| Zimbabwe | WHO Mortality Database | Vital Registry | 1990 | All Ages | 343,997 |
| Zimbabwe | Zimbabwe Mortality Report | Vital Registry | 2007 | All Ages | 576,849 |

| **Appendix Table 3. GBD 2015 Citations Sorted by CVD Cause** | |
| --- | --- |
| **GBD cause name** | **Citation** |
| Atrial fibrillation and flutter | Goudevenos JA, Vakalis JN, Giogiakas V, Lathridou P, Katsouras C, Michalis LK, Sideris DA. An epidemiological study of symptomatic paroxysmal atrial fibrillation in northwest Greece. Europace. 1999; 1(4): 226-33. |
| Atrial fibrillation and flutter | Ott A, Breteler MMB, de Bruyne MC, van Harskamp F, Grobbee DE, Hofman A. Atrial Fibrillation and Dementia in a Population-Based Study: The Rotterdam Study. Stroke. 1997; 28(2): 316 -321. |
| Atrial fibrillation and flutter | Gehring J, Perz S, Stieber J, Kufner R, Keil U. Cardiovascular risk factors, ECG abnormalities and quality of life in subjects with atrial fibrillation. Soz Praventivmed. 1996; 41(3): 185-93. |
| Atrial fibrillation and flutter | Molander U, Dey DK, Sundh V, Steen B. ECG abnormalities in the elderly: prevalence, time and generation trends and association with mortality. Aging Clin Exp Res. 2003; 15(6): 488-93. |
| Atrial fibrillation and flutter | Cacciatore F, Gallo C, Ferrara N, Abete P, Paolisso G, Canonico S, Signoriello G, Terracciano C, Napoli C, Varricchio M, Rengo F. Morbidity patterns in aged population in southern Italy. A survey sampling. Arch Gen Psychiatry. 1998; 26(3): 201-13. |
| Atrial fibrillation and flutter | Nakayama T, Yokoyama T, Yoshiike N, Zaman MM, Date C, Tanaka H, Detels R. Population Attributable Fraction of Stroke Incidence in Middle-Aged and Elderly People: Contributions of Hypertension, Smoking and Atrial Fibrillation. Neuroepidemiology. 20... |
| Atrial fibrillation and flutter | Masia R, Sala J, Marrugat J, Pena A. Prevalencia de fibrilación auricular en la provincia de Girona: el Estudio REGICOR. Rev Esp Cardiol. 2001; 54(10): 1240. |
| Atrial fibrillation and flutter | Go AS, Hylek EM, Phillips KA, Chang Y, Henault LE, Selby JV, Singer DE. Prevalence of diagnosed atrial fibrillation in adults: National implications for rhythm management and stroke prevention. JAMA. 2001; 285(18): 2370 -2375. |
| Atrial fibrillation and flutter | De Bacquer D, De Backer G, Kornitzer M. Prevalences of ECG findings in large population based samples of men and women. Heart. 2000; 84(6): 625-33. |
| Atrial fibrillation and flutter | De Lusignan S, Van Vlymen J, Hague N, Thana L, Dzregah B, Chan T. Preventing stroke in people with atrial fibrillation: a cross-sectional study. J Public Health (Oxf). 2005; 27(1): 85 -92. |
| Atrial fibrillation and flutter | Miyasaka Y, Barnes ME, Gersh BJ, Cha SS, Bailey KR, Abhayaratna WP, Seward JB, Tsang TSM. Secular Trends in Incidence of Atrial Fibrillation in Olmsted County, Minnesota, 1980 to 2000, and Implications on the Projections for Future Prevalence. Circula... |
| Atrial fibrillation and flutter | DeWilde S, Carey IM, Emmas C, Richards N, Cook DG. Trends in the prevalence of diagnosed atrial fibrillation, its treatment with anticoagulation and predictors of such treatment in UK primary care. Heart. 2006; 92(8): 1064-70. |
| Atrial fibrillation and flutter | Friberg J, Scharling H, Gadsbøll N, Jensen GB. Sex-specific increase in the prevalence of atrial fibrillation (The Copenhagen City Heart Study). Am J Cardiol. 2003; 92(12): 1419-23. |
| Atrial fibrillation and flutter | Chien K-L, Su T-C, Hsu H-C, Chang W-T, Chen P-C, Chen M-F, Lee Y-T. Atrial fibrillation prevalence, incidence and risk of stroke and all-cause death among Chinese. Int J Cardiol. 2010; 139(2): 173-80. |
| Atrial fibrillation and flutter | Zhou Z, Hu D. An Epidemiological Study on the Prevalence of Atrial Fibrillation in the Chinese Population of Mainland China. J Epidemiol. 2008; 18(5): 209-16. |
| Atrial fibrillation and flutter | Lakshminarayan K, Solid CA, Collins AJ, Anderson DC, Herzog CA. Atrial fibrillation and stroke in the general Medicare population: a 10-year perspective (1992 to 2002). Stroke. 2006; 37(8): 1969-74. |
| Atrial fibrillation and flutter | Clua Espuny JL, Dalmau Llorca MR, Aguilar Martín C. [Characteristics of oral anti-coagulation treatment in high-risk chronic auricular fibrillation]. Aten Primaria. 2004; 34(8): 414-9. |
| Atrial fibrillation and flutter | Bordin P, Mazzone C, Pandullo C, Goldstein D, Scardi S. Morbidity and mortality in 229 elderly patients with nonrheumatic atrial fibrillation. A five-year follow-up. Ital Heart J. 2003; 4(8): 537-43. |
| Atrial fibrillation and flutter | Wolf PA, Benhamin EJ, Belanger AJ, Kannel WB, Levy D, D'Agostino RB. Secular trends in the prevalence of atrial fibrillation: The Framingham study. Am Heart J. 1996; 131(4): 790-5. |
| Atrial fibrillation and flutter | Guertin JR, Dorais M, Khairy P, Sauriol L, Matteau A, Poulin F, Talajic M, Roy D, LeLorier J. Atrial fibrillation: a real-life observational study in the Québec population. Can J Cardiol. 2011; 27(6): 794-9. |
| Atrial fibrillation and flutter | Ntep-Gweth M, Zimmermann M, Meiltz A, Kingue S, Ndobo P, Urban P, Bloch A. Atrial fibrillation in Africa: clinical characteristics, prognosis, and adherence to guidelines in Cameroon. Europace. 2010; 12(4): 482-7. |
| Atrial fibrillation and flutter | Sandhu RK, Bakal JA, Ezekowitz JA, McAlister FA. The epidemiology of atrial fibrillation in adults depends on locale of diagnosis. Am Heart J. 2011; 161(5): 986-992. |
| Atrial fibrillation and flutter | Salam AM, AlBinali HA, Al-Sulaiti EM, Al-Mulla AW, Singh R, Al Suwaidi J. Effect of age on treatment, trends and outcome of patients hospitalized with atrial fibrillation: insights from a 20-years registry in a Middle-Eastern country (1991-2010). Agin... |
| Atrial fibrillation and flutter | Stewart S, Hart CL, Hole DJ, McMurray JJ. A population-based study of the long-term risks associated with atrial fibrillation: 20-year follow-up of the Renfrew/Paisley study. Am J Med. 2002; 113(5): 359-64. |
| Atrial fibrillation and flutter | Lake FR, Cullen KJ, de Klerk NH, McCall MG, Rosman DL. Atrial fibrillation and mortality in an elderly population. Aust N Z J Med. 1989; 19(4): 321-6. |
| Atrial fibrillation and flutter | Krahn AD, Manfreda J, Tate RB, Mathewson FAL, Cuddy TE. The natural history of atrial fibrillation: Incidence, risk factors, and prognosis in the manitoba follow-up study. Am J Med. 1995; 98(5): 476-84. |
| Atrial fibrillation and flutter | Friberg J, Scharling H, Gadsbøll N, Truelsen T, Jensen GB. Comparison of the impact of atrial fibrillation on the risk of stroke and cardiovascular death in women versus men (The Copenhagen City Heart Study). Am J Cardiol. 2004; 94(7): 889-94. |
| Atrial fibrillation and flutter | Filippi A, Bettoncelli G, Zaninelli A. Detected atrial fibrillation in North Italy: rates, calculated stroke risk and proportion of patients receiving thrombo-prophylaxis. Fam Pract. 2000; 17(4): 337 -339. |
| Atrial fibrillation and flutter | González-Peredo R, Muñoz-Esteban C, Amado-Fernández C, Riancho JA. [Known and concealed auricular fibrillation in the population consulting in a health area]. Aten Primaria. 2007; 39(2): 106-7. |
| Atrial fibrillation and flutter | Yap KB, Ng TP, Ong HY. Low prevalence of atrial fibrillation in community-Disability Weightelling Chinese aged 55 years or older in Singapore: a population-based study. J Electrocardiol. 2008; 41(2): 94-8. |
| Atrial fibrillation and flutter | Jeong JH. Prevalence of and Risk Factors for Atrial Fibrillation in Korean Adults Older than 40 Years. J Korean Med Sci. 2005; 20(1): 26-30. |
| Atrial fibrillation and flutter | Furberg CD, Psaty BM, Manolio TA, Gardin JM, Smith VE, Rautaharju PM. Prevalence of atrial fibrillation in elderly subjects (the Cardiovascular Health Study). Am J Cardiol. 1994; 74(3): 236-41. |
| Atrial fibrillation and flutter | Lok N-S, Lau C-P. Prevalence of palpitations, cardiac arrhythmias and their associated risk factors in ambulant elderly. Int J Cardiol. 1996; 54(3): 231-6. |
| Atrial fibrillation and flutter | Martín Acicoya D, Pedrero Pérez P, Martínez García JA, González Alvaro A, Hernando López T, Herreros Hernanz I. [Prevention of thromboembolism in patients with chronic atrial fibrillation in primary health care]. Med Clin (Barc). 2004; 122(2... |
| Atrial fibrillation and flutter | Bonhorst D, Mendes M, Adragão P, De Sousa J, Primo J, Leiria E, Rocha P. Prevalence of atrial fibrillation in the Portuguese population aged 40 and over: the FAMA study. Rev Port Cardiol. 2010; 29(3): 331-50. |
| Atrial fibrillation and flutter | Claes N, Van Laethem C, Goethals M, Goethals P, Mairesse G, Schwagten B, Nuyens D, Schrooten W, Vijgen J. Prevalence of atrial fibrillation in adults participating in a large-scale voluntary screening programme in Belgium. Acta Cardiol. 2012; 67(3... |
| Atrial fibrillation and flutter | Huang C-C, Chan W-L, Luo J-C, Chen Y-C, Chen T-J, Chung C-M, Huang P-H, Lin S-J, Chen J-W, Leu H-B. Gastroesophageal reflux disease and atrial fibrillation: a nationwide population-based study. PLoS One. 2012; 7(10): e47575. |
| Atrial fibrillation and flutter | Lindhardsen J, Ahlehoff O, Gislason GH, Madsen OR, Olesen JB, Svendsen JH, Torp-Pedersen C, Hansen PR. Risk of atrial fibrillation and stroke in rheumatoid arthritis: Danish nationwide cohort study. BMJ. 2012; 344: e1257. |
| Atrial fibrillation and flutter | Mashal A, Katz A, Shvartzman P. Atrial fibrillation: a primary care cross-sectional study. Isr Med Assoc J. 2011; 13(11): 666-71. |
| Atrial fibrillation and flutter | Mathur R, Pollara E, Hull S, Schofield P, Ashworth M, Robson J. Ethnicity and stroke risk in patients with atrial fibrillation. Heart. 2013; 99(15): 1087-92. |
| Atrial fibrillation and flutter | Nielsen JB, Graff C, Pietersen A, Lind B, Struijk JJ, Olesen MS, Haunsø S, Gerds TA, Svendsen JH, Køber L, Holst AG. J-shaped association between QTc interval duration and the risk of atrial fibrillation: results from the Copenhagen ECG study. J Am ... |
| Atrial fibrillation and flutter | Ntaios G, Manios E, Synetou M, Savvari P, Vemmou A, Koromboki E, Saliaris M, Blanas K, Vemmos K. Prevalence of atrial fibrillation in Greece: the Arcadia Rural Study on Atrial Fibrillation. Acta Cardiol. 2012; 67(1): 65-9. |
| Atrial fibrillation and flutter | Oyen N, Ranthe MF, Carstensen L, Boyd HA, Olesen MS, Olesen S-P, Wohlfahrt J, Melbye M. Familial aggregation of lone atrial fibrillation in young persons. J Am Coll Cardiol. 2012; 60(10): 917-21. |
| Atrial fibrillation and flutter | Piccinocchi G, Laringe M, Guillaro B, Arpino G, Piccinocchi R, Nigro G, Calabrò P. Diagnosis and management of atrial fibrillation by primary care physicians in Italy: a retrospective, observational analysis. Clin Drug Investig. 2012; 32(11): 771... |
| Atrial fibrillation and flutter | Schmutz M, Beer-Borst S, Meiltz A, Urban P, Gaspoz J-M, Costanza MC, Morabia A, Zimmermann M. Low prevalence of atrial fibrillation in asymptomatic adults in Geneva, Switzerland. Europace. 2010; 12(4): 475-81. |
| Atrial fibrillation and flutter | Sliwa K, Carrington MJ, Klug E, Opie L, Lee G, Ball J, Stewart S. Predisposing factors and incidence of newly diagnosed atrial fibrillation in an urban African community: insights from the Heart of Soweto Study. Heart. 2010; 96(23): 1878-82. |
| Atrial fibrillation and flutter | Stefansdottir H, Aspelund T, Gudnason V, Arnar DO. Trends in the incidence and prevalence of atrial fibrillation in Iceland and future projections. Europace. 2011; 13(8): 1110-7. |
| Atrial fibrillation and flutter | Heeringa J, van der Kuip DAM, Hofman A, Kors JA, van Herpen G, Stricker BHC, Stijnen T, Lip GYH, Witteman JCM. Prevalence, incidence and lifetime risk of atrial fibrillation: the Rotterdam study. Eur Heart J. 2006; 27(8): 949-53. |
| Atrial fibrillation and flutter | Andersson P, Löndahl M, Abdon N-J, Terent A. The prevalence of atrial fibrillation in a geographically well-defined population in northern Sweden: implications for anticoagulation prophylaxis. J Intern Med. 2012; 272(2): 170-6. |
| Atrial fibrillation and flutter | Albertsen IE, Rasmussen LH, Lane DA, Overvad TF, Skjøth F, Overvad K, Lip GYH, Larsen TB. The impact of smoking on thromboembolism and mortality in patients with incident atrial fibrillation: insights from the Danish Diet, Cancer, and Health study. C... |
| Atrial fibrillation and flutter | Björck S, Palaszewski B, Friberg L, Bergfeldt L. Atrial fibrillation, stroke risk, and warfarin therapy revisited: a population-based study. Stroke. 2013; 44(11): 3103–8. |
| Atrial fibrillation and flutter | Dewhurst MJ, Adams PC, Gray WK, Dewhurst F, Orega GP, Chaote P, Walker RW. Strikingly low prevalence of atrial fibrillation in elderly Tanzanians. J Am Geriatr Soc. 2012; 60(6): 1135–40. |
| Atrial fibrillation and flutter | Baena-Díez JM, Grau M, Forés R, Fernández-Bergés D, Elosua R, Sorribes M, Félix-Redondo FJ, Segura A, Rigo F, Cabrera de León A, Sanz H, Marrugat J, Sala J, en representación del estudio DARIOS. Prevalence of atrial fibrillation and its associated... |
| Atrial fibrillation and flutter | Friberg L, Engdahl J, Frykman V, Svennberg E, Levin L-Å, Rosenqvist M. Population screening of 75- and 76-year-old men and women for silent atrial fibrillation (STROKESTOP). Europace. 2013; 15(1): 135–40. |
| Atrial fibrillation and flutter | Gómez-Doblas JJ, Muñiz J, Martin JJA, Rodríguez-Roca G, Lobos JM, Awamleh P, Permanyer-Miralda G, Chorro FJ, Anguita M, Roig E, OFRECE study collaborators. Prevalence of atrial fibrillation in Spain. OFRECE study results. Rev Esp Cardiol (Engl Ed)<... |
| Atrial fibrillation and flutter | Hung C-Y, Lin C-H, Wang K-Y, Huang J-L, Hsieh Y-C, Loh E-W, Lan T-H, Chou P, Ting C-T, Wu T-J. Dosage of statin, cardiovascular comorbidities, and risk of atrial fibrillation: a nationwide population-based cohort study. Int J Cardiol. 2013; 168(2)... |
| Atrial fibrillation and flutter | Jansen S, Frewen J, Finucane C, de Rooij SE, van der Velde N, Kenny RA. AF is associated with self-reported syncope and falls in a general population cohort. Age Ageing. 2015; 44(4): 598–603. |
| Atrial fibrillation and flutter | Larsson SC, Drca N, Jensen-Urstad M, Wolk A. Incidence of atrial fibrillation in relation to birth weight and preterm birth. Int J Cardiol. 2015; 149–52. |
| Atrial fibrillation and flutter | Long MJ, Jiang CQ, Lam TH, Xu L, Zhang WS, Lin JM, Ou JP, Cheng KK. Atrial fibrillation and obesity among older Chinese: the Guangzhou Biobank Cohort Study. Int J Cardiol. 2011; 148(1): 48–52. |
| Atrial fibrillation and flutter | Norberg J, Bäckström S, Jansson J-H, Johansson L. Estimating the prevalence of atrial fibrillation in a general population using validated electronic health data. Clin Epidemiol. 2013; 5: 475–81. |
| Atrial fibrillation and flutter | Schnabel RB, Wilde S, Wild PS, Munzel T, Blankenberg S. Atrial fibrillation: its prevalence and risk factor profile in the German general population. Dtsch Arztebl Int. 2012; 109(16): 293–9. |
| Atrial fibrillation and flutter | Renoux C, Patenaude V, Suissa S. Incidence, mortality, and sex differences of non-valvular atrial fibrillation: a population-based study. J Am Heart Assoc. 2014; 3(6): e001402. |
| Atrial fibrillation and flutter | Sun G-Z, Guo L, Wang X-Z, Song H-J, Li Z, Wang J, Sun Y-X. Prevalence of atrial fibrillation and its risk factors in rural China: a cross-sectional study. Int J Cardiol. 2015; 13–7. |
| Atrial fibrillation and flutter | Suzuki S, Otsuka T, Sagara K, Kano H, Matsuno S, Takai H, Kato Y, Uejima T, Oikawa Y, Nagashima K, Kirigaya H, Kunihara T, Yajima J, Sawada H, Aizawa T, Yamashita T. Association between smoking habits and the first-time appearance of atrial fibrillation ... |
| Atrial fibrillation and flutter | Wilke T, Groth A, Mueller S, Pfannkuche M, Verheyen F, Linder R, Maywald U, Kohlmann T, Feng Y-S, Breithardt G, Bauersachs R. Oral anticoagulation use by patients with atrial fibrillation in Germany. Adherence to guidelines, causes of anticoagulation und... |
| Atrial fibrillation and flutter | Ninios I, Bogossian H, Zarse M, Lazaridou F, Dimitriadis K, Ninios V, Lemke B, Louridas G. Prevalence, clinical correlates and treatment of permanent atrial fibrillation among the elderly: insights from the first prospective population-based study in rur... |
| Atrial fibrillation and flutter | Baber U, Howard VJ, Halperin JL, Soliman EZ, Zhang X, McClellan W, Warnock DG, Muntner P. Association of chronic kidney disease with atrial fibrillation among adults in the United States: REasons for Geographic and Racial Differences in Stroke (REGARDS) ... |
| Atrial fibrillation and flutter | Marengoni A, Qiu C, Winblad B, Fratiglioni L. Atrial fibrillation, stroke and dementia in the very old: a population-based study. Neurobiol Aging. 2011; 32(7): 1336–7. |
| Atrial fibrillation and flutter | Misialek JR, Rose KM, Everson-Rose SA, Soliman EZ, Clark CJ, Lopez FL, Alonso A. Socioeconomic status and the incidence of atrial fibrillation in whites and blacks: the Atherosclerosis Risk in Communities (ARIC) study. J Am Heart Assoc. 2014; 3(4). |
| Atrial fibrillation and flutter | Nyrnes A, Mathiesen EB, Njølstad I, Wilsgaard T, Løchen M-L. Palpitations are predictive of future atrial fibrillation. An 11-year follow-up of 22,815 men and women: the Tromsø Study. Eur J Prev Cardiol. 2013; 20(5): 729–36. |
| Atrial fibrillation and flutter | Haim M, Hoshen M, Reges O, Rabi Y, Balicer R, Leibowitz M. Prospective national study of the prevalence, incidence, management and outcome of a large contemporary cohort of patients with incident non-valvular atrial fibrillation. J Am Heart Assoc.... |
| Atrial fibrillation and flutter | Prineas RJ, Le A, Soliman EZ, Zhang Z-M, Howard VJ, Ostchega Y, Howard G, Reasons for Geographic and Racial Differences in Stroke (REGARDS) Investigators. United States national prevalence of electrocardiographic abnormalities in black and white middle-a... |
| Atrial fibrillation and flutter | Ceresne L, Upshur RE. Atrial fibrillation in a primary care practice: prevalence and management. BMC Fam Pract. 2002; 3: 11. |
| Atrial fibrillation and flutter | Önundarson PT, Thorgeirsson G, Jonmundsson E, Sigfusson N, Hardarson T. Chronic atrial fibrillation- epidemiologic features and 14 year follow-up: A case control study. Eur Heart J. 1987; 8(5): 521 -527. |
| Atrial fibrillation and flutter | Vaziri S, Larson M, Benjamin E, Levy D. Echocardiographic predictors of nonrheumatic atrial fibrillation. The Framingham Heart Study. Circulation. 1994; 89(2): 724 -730. |
| Atrial fibrillation and flutter | Kaushal SS, DasGupta DJ, Prashar BS, BharDisability Weightaj AK. Electrocardiographic manifestations of healthy residents of a tribal Himalayan village. J Assoc Physicians India. 1995; 43(1): 15-6. |
| Atrial fibrillation and flutter | Schnabel RB, Yin X, Gona P, Larson MG, Beiser AS, McManus DD, Newton-Cheh C, Lubitz SA, Magnani JW, Ellinor PT, Seshadri S, Wolf PA, Vasan RS, Benjamin EJ, Levy D. 50 year trends in atrial fibrillation prevalence, incidence, risk factors, and mortality i... |
| Atrial fibrillation and flutter | Sudlow M, Thomson R, Thwaites B, Rodgers H, Kenny RA. Prevalence of atrial fibrillation and eligibility for anticoagulants in the community. Lancet. 1998; 352(9135): 1167-71. |
| Atrial fibrillation and flutter | Candel FJ, Matesanz M, Cogolludo F, Candel I, Mora C, Bescos T, Martín M, Vila i Costa I. Prevalencia de fibrilación auricular y factores relacionados en una población del centro de Madrid. An Med Interna. 2004; 21(10): 17-22. |
| Atrial fibrillation and flutter | Habibzadeh F, Yadollahie M, Roshanipoor M, Haghighi AB. Prevalence of atrial fibrillation in a primary health care centre in Fars Province, Islamic Republic of Iran. East Mediterr Health J. 2004; 10(1-2): 147-51. |
| Atrial fibrillation and flutter | Benjamin EJ, Wolf PA, D'Agostino RB, Silbershatz H, Kannel WB, Levy D. Impact of atrial fibrillation on the risk of death: the Framingham Heart Study. Circulation. 1998; 98(10): 946-52. |
| Atrial fibrillation and flutter | Wändell P. A survey of subjects with present or previous atrial fibrillation in a Swedish community. Scand J Prim Health Care. 2001; 19(1): 20-4. |
| Atrial fibrillation and flutter | Ohsawa M, Okayama A, Okamura T, Itai K, Nakamura M, Tanno K, Kato K, Yaegashi Y, Onoda T, Sakata K, Ueshima H. Mortality risk attributable to atrial fibrillation in middle-aged and elderly people in the Japanese general population: nineteen-year follow-u... |
| Atrial fibrillation and flutter | Inoue H, Fujiki A, Origasa H, Ogawa S, Okumura K, Kubota I, Aizawa Y, Yamashita T, Atarashi H, Horie M, Ohe T, Doi Y, Shimizu A, Chishaki A, Saikawa T, Yano K, Kitabatake A, Mitamura H, Kodama I, Kamakura S. Prevalence of atrial fibrillation in the gener... |
| Atrial fibrillation and flutter | Cea-Calvo L, Redón J, Lozano JV, Fernández-Pérez C, Martí-Canales JC, Llisterri JL, González-Esteban J, Aznar J. Prevalencia de fibrilación auricular en la población española de 60 o más años de edad. Estudio PREV-ICTUS. Rev Esp Cardiol.... |
| Atrial fibrillation and flutter | Labrador García MS, Merino Segovia R, Jiménez Domínguez C, García Salvador Y, Segura Fragoso A, Hernández Lanchas C. [Prevalence of auricular fibrillation in people over 65 years of age in a health area]. Aten Primaria. 2001; 28(10): 648-51. |
| Atrial fibrillation and flutter | Hill JD, Mottram EM, Killeen PD. Study of the prevalence of atrial fibrillation in general practice patients over 65 years of age. J R Coll Gen Pract. 1987; 37(297): 172-3. |
| Atrial fibrillation and flutter | Majeed A, Moser K, Carroll K. Trends in the prevalence and management of atrial fibrillation in general practice in England and Wales, 1994-1998: analysis of data from the general practice research database. Heart. 2001; 86(3): 284 -288. |
| Atrial fibrillation and flutter | Smith JG, Newton-Cheh C, Almgren P, Struck J, Morgenthaler NG, Bergmann A, Platonov PG, Hedblad B, Engström G, Wang TJ, Melander O. Assessment of conventional cardiovascular risk factors and multiple biomarkers for the prediction of incident heart failu... |
| Atrial fibrillation and flutter | Suzuki S, Sagara K, Otsuka T, Kano H, Matsuno S, Takai H, Uejima T, Oikawa Y, Koike A, Nagashima K, Kirigaya H, Yajima J, Tanabe H, Sawada H, Aizawa T, Yamashita T. Usefulness of frequent supraventricular extrasystoles and a high CHADS2 score to predict ... |
| Atrial fibrillation and flutter | Suzuki S, Yamashita T, Ohtsuka T, Sagara K, Uejima T, Oikawa Y, Yajima J, Koike A, Nagashima K, Kirigaya H, Ogasawara K, Sawada H, Yamazaki T, Aizawa T. Body size and atrial fibrillation in Japanese outpatients. Circ J. 2010; 74(1): 66-70. |
| Atrial fibrillation and flutter | Kawabata-Yoshihara LA, Benseñor IM, Kawabata VS, Menezes PR, Scazufca M, Lotufo PA. Prevalence of electrocardiographic findings in elderly individuals: the Sao Paulo aging & health study. Arq Bras Cardiol. 2009; 93(6): 602-7. |
| Atrial fibrillation and flutter | Watanabe H, Watanabe T, Sasaki S, Nagai K, Roden DM, Aizawa Y. Close bidirectional relationship between chronic kidney disease and atrial fibrillation: the Niigata preventive medicine study. Am Heart J. 2009; 158(4): 629-36. |
| Atrial fibrillation and flutter | Upshaw CB. Reduced prevalence of atrial fibrillation in black patients compared with white patients attending an urban hospital: an electrocardiographic study. J Natl Med Assoc. 2002; 94(4): 204-8. |
| Atrial fibrillation and flutter | O'Connell JE, Gray CS. Atrial Fibrillation and Stroke Prevention in the Community. Age Ageing. 1996; 25(4): 307 -309. |
| Atrial fibrillation and flutter | Fornari LS, Calderaro D, Nassar IB, Lauretti C, Nakamura L, Bagnatori R, Ageno W, Caramelli B. Misuse of antithrombotic therapy in atrial fibrillation patients: frequent, pervasive and persistent. J Thromb Thrombolysis. 2006; 23(1): 65-71. |
| Atrial fibrillation and flutter | Iguchi Y, Kimura K, Aoki J, Kobayashi K, Terasawa Y, Sakai K, Shibazaki K. Prevalence of atrial fibrillation in community-Disability Weightelling Japanese aged 40 years or older in Japan: analysis of 41,436 non-employee residents in Kurashiki-city. Circ J. 2008;... |
| Atrial fibrillation and flutter | Lip GY, Golding DJ, Nazir M, Beevers DG, Child DL, Fletcher RI. A survey of atrial fibrillation in general practice: the West Birmingham Atrial Fibrillation Project. Br J Gen Pract. 1997; 47(418): 285-9. |
| Atrial fibrillation and flutter | Fretts AM, Mozaffarian D, Siscovick DS, Heckbert SR, McKnight B, King IB, Rimm EB, Psaty BM, Sacks FM, Song X, Spiegelman D, Lemaitre RN. Associations of plasma phospholipid and dietary alpha linolenic acid with incident atrial fibrillation in older adul... |
| Atrial fibrillation and flutter | World Health Organization Regional Office for Europe (WHO/Europe). European Hospital Morbidity Database. Copenhagen, Denmark: World Health Organization Regional Office for Europe (WHO/Europe). |
| Atrial fibrillation and flutter | Federal Ministry of Health (Austria), Statistics Austria. Austria Hospital Inpatient Discharges 1989-1992. |
| Atrial fibrillation and flutter | Federal Ministry of Health (Austria), Statistics Austria. Austria Hospital Inpatient Discharges 1993-1997. |
| Atrial fibrillation and flutter | Federal Ministry of Health (Austria), Statistics Austria. Austria Hospital Inpatient Discharges 1998-2002. |
| Atrial fibrillation and flutter | Federal Ministry of Health (Austria), Statistics Austria. Austria Hospital Inpatient Discharges 2003-2007. |
| Atrial fibrillation and flutter | Ministry of Health (Brazil). Brazil Hospital Information System 1998-2002. |
| Atrial fibrillation and flutter | Ministry of Health (Brazil). Brazil Hospital Information System 2003-2007. |
| Atrial fibrillation and flutter | Ministry of Health (Brazil). Brazil Hospital Information System 2008-2012. |
| Atrial fibrillation and flutter | Ministry of Health (Brazil). Brazil Hospital Information System 2013-2014. |
| Atrial fibrillation and flutter | National Institute of Statistics and Censuses (Ecuador). Ecuador Hospital Inpatient Discharges 1993-1997. |
| Atrial fibrillation and flutter | National Institute of Statistics and Censuses (Ecuador). Ecuador Hospital Inpatient Discharges 1998-2002. |
| Atrial fibrillation and flutter | National Institute of Statistics and Censuses (Ecuador). Ecuador Hospital Inpatient Discharges 2003-2007. |
| Atrial fibrillation and flutter | National Institute of Statistics and Censuses (Ecuador). Ecuador Hospital Inpatient Discharges 2008-2012. |
| Atrial fibrillation and flutter | Ministry of Health (Mexico). Mexico Ministry of Health Hospital Discharges 2000-2002. |
| Atrial fibrillation and flutter | Ministry of Health (Mexico). Mexico Ministry of Health Hospital Discharges 2003-2007. |
| Atrial fibrillation and flutter | Ministry of Health (Mexico). Mexico Ministry of Health Hospital Discharges 2008-2012. |
| Atrial fibrillation and flutter | National Center for Health Statistics (NCHS), Centers for Disease Control and Prevention (CDC). United States National Hospital Ambulatory Medical Care Survey 1993-1997. |
| Atrial fibrillation and flutter | Norwegian Directorate of Health. Norway Patient Register 2008-2012. |
| Atrial fibrillation and flutter | National Center for Health Statistics (NCHS), Centers for Disease Control and Prevention (CDC). United States National Hospital Ambulatory Medical Care Survey 1998-2002. |
| Atrial fibrillation and flutter | Ministry of Health (New Zealand). New Zealand National Minimum Dataset 2000-2002. |
| Atrial fibrillation and flutter | Ministry of Health (New Zealand). New Zealand National Minimum Dataset 2003-2007. |
| Atrial fibrillation and flutter | Ministry of Health (New Zealand). New Zealand National Minimum Dataset 2008-2012. |
| Atrial fibrillation and flutter | Ministry of Health (New Zealand). New Zealand National Minimum Dataset 2013-2014. |
| Atrial fibrillation and flutter | National Board of Health and Welfare (Sweden). Sweden National Patient Register 1998-2002. |
| Atrial fibrillation and flutter | National Center for Health Statistics (NCHS), Centers for Disease Control and Prevention (CDC). United States National Hospital Ambulatory Medical Care Survey 2003-2007. |
| Atrial fibrillation and flutter | National Board of Health and Welfare (Sweden). Sweden National Patient Register 2003-2007. |
| Atrial fibrillation and flutter | National Board of Health and Welfare (Sweden). Sweden National Patient Register 2008-2012. |
| Atrial fibrillation and flutter | NHS England. United Kingdom - England Hospital Episode Statistics 2003-2008. |
| Atrial fibrillation and flutter | National Center for Health Statistics (NCHS), Centers for Disease Control and Prevention (CDC). United States National Hospital Ambulatory Medical Care Survey 2008-2011. |
| Atrial fibrillation and flutter | NHS England. United Kingdom - England Hospital Episode Statistics 2001-2003. |
| Atrial fibrillation and flutter | NHS England. United Kingdom - England Hospital Episode Statistics 2008-2012. |
| Atrial fibrillation and flutter | National Center for Health Statistics (NCHS), Centers for Disease Control and Prevention (CDC). United States National Hospital Discharge Survey 1988-1992. |
| Atrial fibrillation and flutter | National Center for Health Statistics (NCHS), Centers for Disease Control and Prevention (CDC). United States National Hospital Discharge Survey 1993-1997. |
| Atrial fibrillation and flutter | National Center for Health Statistics (NCHS), Centers for Disease Control and Prevention (CDC). United States National Hospital Discharge Survey 1998-2002. |
| Atrial fibrillation and flutter | National Center for Health Statistics (NCHS), Centers for Disease Control and Prevention (CDC). United States National Hospital Discharge Survey 2003-2007. |
| Atrial fibrillation and flutter | National Center for Health Statistics (NCHS), Centers for Disease Control and Prevention (CDC). United States National Hospital Discharge Survey 2008-2010. |
| Cardiomyopathy and myocarditis | World Health Organization Regional Office for Europe (WHO/Europe). European Hospital Morbidity Database. Copenhagen, Denmark: World Health Organization Regional Office for Europe (WHO/Europe). |
| Cardiomyopathy and myocarditis | Ministry of Health (New Zealand). New Zealand National Minimum Dataset 2000-2002. |
| Cardiomyopathy and myocarditis | Ministry of Health (New Zealand). New Zealand National Minimum Dataset 2003-2007. |
| Cardiomyopathy and myocarditis | Ministry of Health (New Zealand). New Zealand National Minimum Dataset 2008-2012. |
| Cardiomyopathy and myocarditis | Ministry of Health (New Zealand). New Zealand National Minimum Dataset 2013-2014. |
| Cardiomyopathy and myocarditis | Federal Ministry of Health (Austria), Statistics Austria. Austria Hospital Inpatient Discharges 1989-1992. |
| Cardiomyopathy and myocarditis | Federal Ministry of Health (Austria), Statistics Austria. Austria Hospital Inpatient Discharges 1993-1997. |
| Cardiomyopathy and myocarditis | Federal Ministry of Health (Austria), Statistics Austria. Austria Hospital Inpatient Discharges 1998-2002. |
| Cardiomyopathy and myocarditis | Federal Ministry of Health (Austria), Statistics Austria. Austria Hospital Inpatient Discharges 2003-2007. |
| Cardiomyopathy and myocarditis | Norwegian Directorate of Health. Norway Patient Register 2008-2012. |
| Cardiomyopathy and myocarditis | National Board of Health and Welfare (Sweden). Sweden National Patient Register 1998-2002. |
| Cardiomyopathy and myocarditis | National Board of Health and Welfare (Sweden). Sweden National Patient Register 2003-2007. |
| Cardiomyopathy and myocarditis | National Board of Health and Welfare (Sweden). Sweden National Patient Register 2008-2012. |
| Cardiomyopathy and myocarditis | National Center for Health Statistics (NCHS), Centers for Disease Control and Prevention (CDC). United States National Hospital Discharge Survey 1988-1992. |
| Cardiomyopathy and myocarditis | National Center for Health Statistics (NCHS), Centers for Disease Control and Prevention (CDC). United States National Hospital Discharge Survey 1993-1997. |
| Cardiomyopathy and myocarditis | National Center for Health Statistics (NCHS), Centers for Disease Control and Prevention (CDC). United States National Hospital Ambulatory Medical Care Survey 1993-1997. |
| Cardiomyopathy and myocarditis | National Center for Health Statistics (NCHS), Centers for Disease Control and Prevention (CDC). United States National Hospital Ambulatory Medical Care Survey 1998-2002. |
| Cardiomyopathy and myocarditis | National Center for Health Statistics (NCHS), Centers for Disease Control and Prevention (CDC). United States National Hospital Discharge Survey 1998-2002. |
| Cardiomyopathy and myocarditis | National Center for Health Statistics (NCHS), Centers for Disease Control and Prevention (CDC). United States National Hospital Discharge Survey 2003-2007. |
| Cardiomyopathy and myocarditis | National Center for Health Statistics (NCHS), Centers for Disease Control and Prevention (CDC). United States National Hospital Ambulatory Medical Care Survey 2003-2007. |
| Cardiomyopathy and myocarditis | National Center for Health Statistics (NCHS), Centers for Disease Control and Prevention (CDC). United States National Hospital Discharge Survey 2008-2010. |
| Cardiomyopathy and myocarditis | National Center for Health Statistics (NCHS), Centers for Disease Control and Prevention (CDC). United States National Hospital Ambulatory Medical Care Survey 2008-2011. |
| Cardiomyopathy and myocarditis | National Institute of Statistics and Censuses (Ecuador). Ecuador Hospital Inpatient Discharges 1993-1997. |
| Cardiomyopathy and myocarditis | National Institute of Statistics and Censuses (Ecuador). Ecuador Hospital Inpatient Discharges 1998-2002. |
| Cardiomyopathy and myocarditis | National Institute of Statistics and Censuses (Ecuador). Ecuador Hospital Inpatient Discharges 2003-2007. |
| Cardiomyopathy and myocarditis | National Institute of Statistics and Censuses (Ecuador). Ecuador Hospital Inpatient Discharges 2008-2012. |
| Cardiomyopathy and myocarditis | NHS England. United Kingdom - England Hospital Episode Statistics 2001-2003. |
| Cardiomyopathy and myocarditis | NHS England. United Kingdom - England Hospital Episode Statistics 2003-2008. |
| Cardiomyopathy and myocarditis | NHS England. United Kingdom - England Hospital Episode Statistics 2008-2012. |
| Cardiomyopathy and myocarditis | Ministry of Health (Mexico). Mexico Ministry of Health Hospital Discharges 2000-2002. |
| Cardiomyopathy and myocarditis | Ministry of Health (Mexico). Mexico Ministry of Health Hospital Discharges 2003-2007. |
| Cardiomyopathy and myocarditis | Ministry of Health (Mexico). Mexico Ministry of Health Hospital Discharges 2008-2012. |
| Cardiomyopathy and myocarditis | Ministry of Health (Brazil). Brazil Hospital Information System 1998-2002. |
| Cardiomyopathy and myocarditis | Ministry of Health (Brazil). Brazil Hospital Information System 2003-2007. |
| Cardiomyopathy and myocarditis | Ministry of Health (Brazil). Brazil Hospital Information System 2008-2012. |
| Cardiomyopathy and myocarditis | Ministry of Health (Brazil). Brazil Hospital Information System 2013-2014. |
| Cerebrovascular disease | Banerjee TK, Mukherjee CS, Sarkhel A. Stroke in the urban population of Calcutta – an epidemiological study. Neuroepidemiology. 2001; 20(3): 201-7. |
| Cerebrovascular disease | Melcon CM, Melcon MO. Prevalence of stroke in an Argentine community. Neuroepidemiology. 2006; 27(2): 81-8. |
| Cerebrovascular disease | Connor MD, Thorogood M, Casserly B, Dobson C, Warlow CP. Prevalence of stroke survivors in rural South Africa: results from the Southern Africa Stroke Prevention Initiative (SASPI) Agincourt field site. Stroke. 2004; 35(3): 627-32. |
| Cerebrovascular disease | Das SK, Biswas A, Roy T, Banerjee TK, Mukherjee CS, Raut DK, Chaudhuri A. A random sample survey for prevalence of major neurological disorders in Kolkata. Indian J Med Res. 2006; 124(2): 163-72. |
| Cerebrovascular disease | Koul R, Motta A, Razdan S. Epidemiology of young strokes in rural Kashmir, India. Acta Neurol Scand. 1990; 82(1): 1-3. |
| Cerebrovascular disease | Das S, Sanyal K, Moitra A. A pilot study on neuroepidemiology in urban Bengal. Indian J Public Health. 1998; 42(2): 34-6. |
| Cerebrovascular disease | Dhamija RK, Dhamija SB. Prevalence of stroke in rural community – an overview of Indian experience. J Assoc Physicians India. 1998; 46(4): 351-4. |
| Cerebrovascular disease | Mrabet A, Attia-Romdhane N, Ben Hamida M, Gharbi N, Le Noan H, Hentati R, Ben Mansour J, Srairi I. Epidemiologic aspects of cerebrovascular accidents in Tunisia. Rev Neurol (Paris). 1990; 146(4): 297-301. |
| Cerebrovascular disease | Nicoletti A, Sofia V, Giuffrida S, Bartoloni A, Bartalesi F, Bartolo MLL, Fermo SL, Cocuzza V, Gamboa H, Salazar E, Reggio A. Prevalence of Stroke: A Door-to-Door Survey in Rural Bolivia. Stroke. 2000; 31(4): 882-5. |
| Cerebrovascular disease | Kamal AK, Itrat A, Murtaza M, Khan M, Rasheed A, Ali A, Akber A, Akber Z, Iqbal N, Shoukat S, Majeed F, Saleheen D. The burden of stroke and transient ischemic attack in Pakistan: a community-based prevalence study. BMC Neurol. 2009; 9(1): 58. |
| Cerebrovascular disease | Bonita R, Solomon N, Broad JB. Prevalence of Stroke and Stroke-Related Disability: Estimates From the Auckland Stroke Studies. Stroke. 1997; 28(10): 1898-902. |
| Cerebrovascular disease | Fuh JL, Wang SJ, Larson EB, Liu HC. Prevalence of stroke in Kinmen. Stroke. 1996; 27(8): 1338-41. |
| Cerebrovascular disease | Huang Z-S, Chiang T-L, Lee T-K. Stroke Prevalence in Taiwan: Findings From the 1994 National Health Interview Survey. Stroke. 1997; 28(8): 1579-84. |
| Cerebrovascular disease | De Jesús Llibre J, Valhuerdi A, Fernández O, Llibre JC, Porto R, López AM, Marcheco B, Moreno C. Prevalence of stroke and associated risk factors in older adults in Havana City and Matanzas Provinces, Cuba (10/66 population-based study). MEDICC Rev... |
| Cerebrovascular disease | Orlandi G, Gelli A, Fanucchi S, Tognoni G, Acerbi G, Murri L. Prevalence of stroke and transient ischaemic attack in the elderly population of an Italian rural community. Eur J Epidemiol. 2003; 18(9): 879-82. |
| Cerebrovascular disease | Venketasubramanian N, Tan LCS, Sahadevan S, Chin JJ, Krishnamoorthy ES, Hong CY, Saw SM. Prevalence of Stroke Among Chinese, Malay, and Indian Singaporeans. Stroke. 2005; 36(3): 551-6. |
| Cerebrovascular disease | Cossi M-J, Gobron C, Preux P-M, Niama D, Chabriat H, Houinato D. Stroke: prevalence and disability in Cotonou, Benin. Cerebrovasc Dis. 2012; 33(2): 166-72. |
| Cerebrovascular disease | Mohammad QD, Habib M, Hoque A, Alam B, Haque B, Hossain S, Rahman KM, Khan SU. Prevalence of stroke above forty years. Mymensingh Med J. 2011; 20(4): 640-4. |
| Cerebrovascular disease | Bermejo F, Vega S, Morales JM, Díaz J, López L, Parra D, Colmenarejo C, Gabriel R. Prevalence of stroke in two samples (rural and urban) of old people in Spain. A pilot door-to-door study carried out by health professionals. Neurologia. 1997; 12... |
| Cerebrovascular disease | Danesi M, Okubadejo N, Ojini F. Prevalence of stroke in an urban, mixed-income community in Lagos, Nigeria. Neuroepidemiology. 2007; 28(4): 216-23. |
| Cerebrovascular disease | D'Alessandro G, Gallo F, Vitaliano A, Col PD, Gorraz F, Cristofaro RD, Boaretto G. Prevalence of stroke and stroke-related disability in Valle d'Aosta, Italy. Neurol Sci. 2010; 31(2): 137-41. |
| Cerebrovascular disease | Ferri CP, Schoenborn C, Kalra L, Acosta D, Guerra M, Huang Y, Jacob KS, Rodriguez JJL, Salas A, Sosa AL, Williams JD, Liu Z, Moriyama T, Valhuerdi A, Prince MJ. Prevalence of stroke and related burden among older people living in Latin America, India and... |
| Cerebrovascular disease | Institute for Health Metrics and Evaluation (IHME).&nbsp;CoDEM estimates for standardized stroke mortality rates at the country level, derived from NID 153079. |
| Cerebrovascular disease | Numminen H, Kotila M, Waltimo O, Aho K, Kaste M. Declining Incidence and Mortality Rates of Stroke in Finland From 1972 to 1991: Results of Three Population-Based Stroke Registers. Stroke. 1996; 27(9): 1487-91. |
| Cerebrovascular disease | Vemmos KN, Bots ML, Tsibouris PK, Zis VP, Takis CE, Grobbee DE, Stamatelopoulos S. Prognosis of stroke in the south of Greece: 1 year mortality, functional outcome and its determinants: the Arcadia Stroke Registry. J Neurol Neurosurg Psychiatry. 2... |
| Cerebrovascular disease | Carolei A, Marini C, Di Napoli M, Di Gianfilippo G, Santalucia P, Baldassarre M, Giorgio De Matteis M, di Orio F. High Stroke Incidence in the Prospective Community-Based L'Aquila Registry (1994-1998): First Year's Results. Stroke. 1997; 28(12): 2... |
| Cerebrovascular disease | Di Carlo A, Inzitari D, Galati F, Baldereschi M, Giunta V, Grillo G, Furch&igrave; A, Manno V, Naso F, Vecchio A, Consoli D. A Prospective Community-Based Study of Stroke in Southern Italy: The Vibo Valentia Incidence of Stroke Study (VISS). Cerebrova... |
| Cerebrovascular disease | Sacco S, Stracci F, Cerone D, Ricci S, Carolei A. Epidemiology of stroke in Italy. Int J Stroke. 2011; 6(3): 219-27. |
| Cerebrovascular disease | Hallström B, Jönsson A-C, Nerbrand C, Norrving B, Lindgren A. Stroke Incidence and Survival in the Beginning of the 21st Century in Southern Sweden: Comparisons With the Late 20th Century and Projections Into the Future. Stroke. 2008; 39(1): 10-5. |
| Cerebrovascular disease | Lindmark A, Glader E-L, Asplund K, Norrving B, Eriksson M, Riks-Stroke Collaboration. Socioeconomic disparities in stroke case fatality--Observations from Riks-Stroke, the Swedish stroke register. Int J Stroke. 2014; 9(4): 429–36. |
| Cerebrovascular disease | Jucha R. Stroke incidence and casefatality rates in population of Krosno County. Przegl Lek. 2013; 70(4): 191-4. |
| Cerebrovascular disease | Thrift AG, Dewey HM, Macdonell RAL, McNeil JJ, Donnan GA. Stroke Incidence on the East Coast of Australia: The North East Melbourne Stroke Incidence Study (NEMESIS). Stroke. 2000; 31(9): 2087-92. |
| Cerebrovascular disease | Katzenellenbogen JM, Vos T, Somerford P, Begg S, Semmens JB, Codde JP. Excess Mortality Rates for Estimating the Non-Fatal Burden of Stroke in Western Australia: A Data Linkage Study. Cerebrovasc Dis. 2010; 30(1): 57-64. |
| Cerebrovascular disease | National Centre for Social Research and University College London. Department of Epidemiology and Public Health, Health Survey for England, 2003 [computer file]. Colchester, Essex: UK Data Archive [distributor], March 2005. SN: 5098. |
| Cerebrovascular disease | National Centre for Social Research and University College London. Department of Epidemiology and Public Health, Health Survey for England, 2005 [computer file]. Colchester, Essex: UK Data Archive [distributor], July 2007. SN: 5675. |
| Cerebrovascular disease | National Centre for Social Research and University College London. Department of Epidemiology and Public Health, Health Survey for England, 2006 [computer file]. 4th Edition. Colchester, Essex: UK Data Archive [distributor], July 20... |
| Cerebrovascular disease | National Center for Health Statistics (NCHS), Centers for Disease Control and Prevention (CDC). United States National Health and Nutrition Examination Survey 2007-2008. Hyattsville, United States: National Center for Health Statistics (NCHS), Centers... |
| Cerebrovascular disease | Centers for Disease Control and Prevention (CDC). United States Behavioral Risk Factor Surveillance System 2005. Atlanta, Georgia: CDC, US Department of Health and Human Services. |
| Cerebrovascular disease | Centers for Disease Control and Prevention (CDC). United States Behavioral Risk Factor Surveillance System 2006. Atlanta, Georgia: CDC, US Department of Health and Human Services. |
| Cerebrovascular disease | Centers for Disease Control and Prevention (CDC). United States Behavioral Risk Factor Surveillance System 2007. Atlanta, Georgia: CDC, US Department of Health and Human Services. |
| Cerebrovascular disease | Centers for Disease Control and Prevention (CDC). United States Behavioral Risk Factor Surveillance System 2008. Atlanta, Georgia: CDC, US Department of Health and Human Services. |
| Cerebrovascular disease | Centers for Disease Control and Prevention (CDC). United States Behavioral Risk Factor Surveillance System 2009. Atlanta, Georgia: CDC, US Department of Health and Human Services. |
| Cerebrovascular disease | National Center for Health Statistics (NCHS), Centers for Disease Control and Prevention (CDC). United States National Health and Nutrition Examination Survey 2005-2006. Hyattsville, United States: National Center for Health Statistics (NCHS), Centers... |
| Cerebrovascular disease | National Center for Health Statistics (NCHS), Centers for Disease Control and Prevention (CDC). United States National Health and Nutrition Examination Survey 2009-2010. Hyattsville, United States: National Center for Health Statistics (NCHS), Centers... |
| Cerebrovascular disease | National Center for Health Statistics (NCHS), Centers for Disease Control and Prevention (CDC). United States National Health and Nutrition Examination Survey 2001-2002. Hyattsville, United States: National Center for Health Statistics (NCHS), Centers... |
| Cerebrovascular disease | Centers for Disease Control and Prevention (CDC). United States Behavioral Risk Factor Surveillance System 2010. Atlanta, United States: Centers for Disease Control and Prevention (CDC). |
| Cerebrovascular disease | NatCen Social Research and University College London. Department of Epidemiology and Public Health, Health Survey for England, 2011 [computer file]. Colchester, Essex: UK Data Archive [distributor], April 2013. SN: 7260, http://dx.doi.org/10.5255/UKDA... |
| Cerebrovascular disease | Agency for Healthcare Research and Quality. United States Medical Expenditure Panel Survey 2002-2009. Rockville, United States: Agency for Healthcare Research and Quality. |
| Cerebrovascular disease | Longe AC, Osuntokun BO. Prevalence of neurological disorders in Udo, a rural community in southern Nigeria. Trop Geogr Med. 1989; 41(1): 36-40. |
| Cerebrovascular disease | Das SK, Banerjee TK, Biswas A, Roy T, Raut DK, Mukherjee CS, Chaudhuri A, Hazra A, Roy J. A Prospective Community-Based Study of Stroke in Kolkata, India. Stroke. 2007; 38(3): 906-10. |
| Cerebrovascular disease | Burke T, Venketasubramanian R. The epidemiology of stroke in the East Asian region: a literature-based review. Int J Stroke. 2006; 1(4): 208-215. |
| Cerebrovascular disease | El Gengaihy E, Wasif S, El Shazli S, Shawky A, Zaitoun A, Sarhan A, El Anwar A, El Taweel Y. Epidemiology Study of Cerebrovascular Diseases in Sharkia Governorate. Med J Cairo Univ. 1993; 61(Suppl 1): 35-51. |
| Cerebrovascular disease | Jungehülsing GJ, Müller-Nordhorn J, Nolte CH, Roll S, Rossnagel K, Reich A, Wagner A, Einhäupl KM, Willich SN, Villringer A. Prevalence of stroke and stroke symptoms: a population-based survey of 28,090 participants. Neuroepidemiology. 2008; 30... |
| Cerebrovascular disease | Díaz-Guzmán J, Bermejo-Pareja F, Benito-León J, Vega S, Gabriel R, Medrano MJ. Prevalence of stroke and transient ischemic attack in three elderly populations of central Spain. Neuroepidemiology. 2008; 30(4): 247-53. |
| Cerebrovascular disease | Lê VT, Lê TL, Nguyên TH, Nguyên HH, Dào TX, Nguyên VT, Pham MB. Strokes in South Vietnam: an epidemiologic study. Rev Neurol (Paris). 1999; 155(2): 137-40. |
| Cerebrovascular disease | Osuntokun BO, Adeuja AO, Schoenberg BS, Bademosi O, Nottidge VA, Olumide AO, Ige O, Yaria F, Bolis CL. Neurological disorders in Nigerian Africans: a community-based study. Acta Neurol Scand. 1987; 75(1): 13-21. |
| Cerebrovascular disease | Osuntokun BO, Schoenberg BS, Nottidge VA, Adeuja A, Kale O, Adeyefa A, Bademosi O, Olumide A, Oyediran ABO, Pearson CA, Bolis CL. Research Protocol for Measuring the Prevalence of Neurologic Disorders in Developing Countries. Neuroepidemiology. 19... |
| Cerebrovascular disease | Prencipe M, Ferretti C, Casini AR, Santini M, Giubilei F, Culasso F. Stroke, disability, and dementia: results of a population survey. Stroke. 1997; 28(3): 531-6. |
| Cerebrovascular disease | Saha SP, Bhattacharya S, Das SK, Maity B, Roy T, Raut DK. Epidemiological study of neurological disorders in a rural population of Eastern India. J Indian Med Assoc. 2003; 101(5): 299-304. |
| Cerebrovascular disease | Tekle-Haimanot R, Abebe M, Gebre-Mariam A, Forsgren L, Heijbel J, Holmgren G, Ekstedt J. Community-based study of neurological disorders in rural central Ethiopia. Neuroepidemiology. 1990; 9(5): 263-77. |
| Cerebrovascular disease | Viriyavejakul A, Poungvarin N, Vannasaeng S. The prevalence of stroke in urban community of Thailand. J Neurol. 1985; 232: 93. |
| Cerebrovascular disease | Walker R, McLarty D, Masuki G, Kitange H, Whiting D, Moshi A, Massawe J, Amaro R, Mhina A, Alberti K. Age specific prevalence of impairment and disability relating to hemiplegic stroke in the Hai District of northern Tanzania. J Neurol Neurosurg Psych... |
| Cerebrovascular disease | Korea Centers for Disease Control and Prevention. Korea, South National Health and Nutrition Examination Survey 2012. |
| Endocarditis | Cecchi E, Chirillo F, Castiglione A, Faggiano P, Cecconi M, Moreo A, Cialfi A, Rinaldi M, Del Ponte S, Squeri A, Corcione S, Canta F, Gaddi O, Enia F, Forno D, Costanzo P, Zuppiroli A, Ronzani G, Bologna F, Patrignani A, Belli R, Ciccone G, De Rosa FG. C... |
| Endocarditis | Walls G, McBride S, Raymond N, Read K, Coomarasamy C, Morris AJ, Chambers S, Holland D, Murdoch DR. Infective endocarditis in New Zealand: data from the International Collaboration on Endocarditis Prospective Cohort Study. N Z Med J. 2014; 127(139... |
| Endocarditis | Hajihossainlou B, Heidarnia M-A, Sharif Kashani B. Changing pattern of infective endocarditis in Iran: A 16 years survey. Pak J Med Sci. 2013; 29(1): 85–90. |
| Endocarditis | Regueiro A, Falces C, Cervera C, Del Rio A, Paré JC, Mestres CA, Castañeda X, Pericàs JM, Azqueta M, Marco F, Ninot S, Almela M, Moreno A, Miró JM, Hospital Clínic Endocarditis Study Group. Risk factors for pericardial effusion in native valve infec... |
| Endocarditis | Ternhag A, Cederström A, Törner A, Westling K. A nationwide cohort study of mortality risk and long-term prognosis in infective endocarditis in Sweden. PLoS One. 2013; 8(7): e67519. |
| Endocarditis | Fedeli U, Schievano E, Buonfrate D, Pellizzer G, Spolaore P. Increasing incidence and mortality of infective endocarditis: a population-based study through a record-linkage system. BMC Infect Dis. 2011; 11(1): 48. |
| Endocarditis | Duval X, Delahaye F, Alla F, Tattevin P, Obadia J-F, Le Moing V, Doco-Lecompte T, Celard M, Poyart C, Strady C, Chirouze C, Bes M, Cambau E, Iung B, Selton-Suty C, Hoen B. Temporal Trends in Infective Endocarditis in the Context of Prophylaxis Guideline ... |
| Endocarditis | Walpot J, Blok W, van Zwienen J, Klazen C, Amsel B. Incidence and complication rate of infective endocarditis in the Dutch region of Walcheren: a 3-year retrospective study. Acta Cardiol. 2006; 61(2): 175-81. |
| Endocarditis | van der Meer JT, Thompson J, Valkenburg HA, Michel MF. Epidemiology of bacterial endocarditis in The Netherlands. I. Patient characteristics. Arch Intern Med. 1992; 152(9): 1863-8. |
| Endocarditis | Benn M, Hagelskjaer LH, Tvede M. Infective endocarditis, 1984 through 1993: a clinical and microbiological survey. J Intern Med. 1997; 242(1): 15-22. |
| Endocarditis | Hoen B, Alla F, Selton-Suty C, Béguinot I, Bouvet A, Briançon S, Casalta JP, Danchin N, Delahaye F, Etienne J, Le Moing V, Leport C, Mainardi JL, Ruimy R,Vandenesch F; Association pour l'Etude et la Prévention de l'Endocardite Infectieus... |
| Endocarditis | Cheng A, Athan E, Appelbe A, McDonald M. The changing profile of bacterial endocarditis as seen at an Australian provincial centre. Heart Lung Circ. 2002; 11(1): 26-31. |
| Endocarditis | Cecchi E, Forno D, Imazio M, Migliardi A, Gnavi R, Dal Conte I, Trinchero R; Piemonte Infective Endocarditis Study Group. New trends in the epidemiological and clinical features of infective endocarditis: results of a multicenterpr... |
| Endocarditis | Delahaye F, Goulet V, Lacassin F, Ecochard R, Selton-Suty C, Hoen B, Etienne J, Briançon S, Leport C. Characteristics of infective endocarditis in France in 1991. A 1-year survey. Eur Heart J. 1995; 16(3): 394-401. |
| Endocarditis | Fonager K, Lindberg J, Thulstrup AM, Pedersen L, Schønheyder HC, Sørensen HT. Incidence and short-term prognosis of infective endocarditis in Denmark, 1980-1997. Scand J Infect Dis. 2003; 35(1): 27-30. |
| Endocarditis | Nolsøe C, Jensen LT, Hartzner K, Godtfredsen J. Occurrence of infectious endocarditis in Denmark. Ugeskr Laeger. 1987; 149(51): 3458-9. |
| Endocarditis | Letaief A, Boughzala E, Kaabia N, Ernez S, Abid F, Ben Chaabane T, Ben Jemaa M, Boujnah R, Chakroun M, Daoud M, Gaha R, Kafsi N, Khalfallah A, Slimane L, Zaouali M. Epidemiology of infective endocarditis in Tunisia: a 10-year multice... |
| Endocarditis | Giannitsioti E, Skiadas I, Antoniadou A, Tsiodras S, Kanavos K, Triantafyllidi H, Giamarellou H; Hellenic Endocarditis Study Group. Nosocomial vs. community-acquired infective endocarditis in Greece: changing epidemiological profile an... |
| Endocarditis | Foghsgaard J, Pedersen SA, Launbjerg J. Incidence and diagnosis of infectious endocarditis in Frederiksborg county, 1990-2000. Ugeskr Laeger. 2004; 166(25): 2446-50. |
| Endocarditis | Goulet V, Etienne J, Fleurette J, Netter R. Infectious endocarditis in France. Epidemiological characteristics. Presse Med. 1986; 15(37): 1855-8. |
| Endocarditis | World Health Organization Regional Office for Europe (WHO/Europe). European Hospital Morbidity Database. Copenhagen, Denmark: World Health Organization Regional Office for Europe (WHO/Europe). |
| Endocarditis | Ministry of Health (New Zealand). New Zealand National Minimum Dataset 2000-2002. |
| Endocarditis | Ministry of Health (New Zealand). New Zealand National Minimum Dataset 2003-2007. |
| Endocarditis | Ministry of Health (New Zealand). New Zealand National Minimum Dataset 2008-2012. |
| Endocarditis | Ministry of Health (New Zealand). New Zealand National Minimum Dataset 2013-2014. |
| Endocarditis | Federal Ministry of Health (Austria), Statistics Austria. Austria Hospital Inpatient Discharges 1989-1992. |
| Endocarditis | Federal Ministry of Health (Austria), Statistics Austria. Austria Hospital Inpatient Discharges 1993-1997. |
| Endocarditis | Federal Ministry of Health (Austria), Statistics Austria. Austria Hospital Inpatient Discharges 1998-2002. |
| Endocarditis | Federal Ministry of Health (Austria), Statistics Austria. Austria Hospital Inpatient Discharges 2003-2007. |
| Endocarditis | Norwegian Directorate of Health. Norway Patient Register 2008-2012. |
| Endocarditis | National Board of Health and Welfare (Sweden). Sweden National Patient Register 1998-2002. |
| Endocarditis | National Board of Health and Welfare (Sweden). Sweden National Patient Register 2003-2007. |
| Endocarditis | National Board of Health and Welfare (Sweden). Sweden National Patient Register 2008-2012. |
| Endocarditis | National Center for Health Statistics (NCHS), Centers for Disease Control and Prevention (CDC). United States National Hospital Discharge Survey 1988-1992. |
| Endocarditis | National Center for Health Statistics (NCHS), Centers for Disease Control and Prevention (CDC). United States National Hospital Discharge Survey 1993-1997. |
| Endocarditis | National Center for Health Statistics (NCHS), Centers for Disease Control and Prevention (CDC). United States National Hospital Discharge Survey 1998-2002. |
| Endocarditis | National Center for Health Statistics (NCHS), Centers for Disease Control and Prevention (CDC). United States National Hospital Discharge Survey 2003-2007. |
| Endocarditis | National Center for Health Statistics (NCHS), Centers for Disease Control and Prevention (CDC). United States National Hospital Discharge Survey 2008-2010. |
| Endocarditis | National Institute of Statistics and Censuses (Ecuador). Ecuador Hospital Inpatient Discharges 1993-1997. |
| Endocarditis | National Institute of Statistics and Censuses (Ecuador). Ecuador Hospital Inpatient Discharges 1998-2002. |
| Endocarditis | National Institute of Statistics and Censuses (Ecuador). Ecuador Hospital Inpatient Discharges 2003-2007. |
| Endocarditis | National Institute of Statistics and Censuses (Ecuador). Ecuador Hospital Inpatient Discharges 2008-2012. |
| Endocarditis | NHS England. United Kingdom - England Hospital Episode Statistics 2001-2003. |
| Endocarditis | NHS England. United Kingdom - England Hospital Episode Statistics 2003-2008. |
| Endocarditis | NHS England. United Kingdom - England Hospital Episode Statistics 2008-2012. |
| Endocarditis | Ministry of Health (Mexico). Mexico Ministry of Health Hospital Discharges 2000-2002. |
| Endocarditis | Ministry of Health (Mexico). Mexico Ministry of Health Hospital Discharges 2003-2007. |
| Endocarditis | Ministry of Health (Mexico). Mexico Ministry of Health Hospital Discharges 2008-2012. |
| Endocarditis | Ministry of Health (Brazil). Brazil Hospital Information System 1998-2002. |
| Endocarditis | Ministry of Health (Brazil). Brazil Hospital Information System 2003-2007. |
| Endocarditis | Ministry of Health (Brazil). Brazil Hospital Information System 2008-2012. |
| Endocarditis | Ministry of Health (Brazil). Brazil Hospital Information System 2013-2014. |
| Endocarditis | National Center for Health Statistics (NCHS), Centers for Disease Control and Prevention (CDC). United States National Hospital Ambulatory Medical Care Survey 1993-1997. |
| Endocarditis | National Center for Health Statistics (NCHS), Centers for Disease Control and Prevention (CDC). United States National Hospital Ambulatory Medical Care Survey 1998-2002. |
| Endocarditis | National Center for Health Statistics (NCHS), Centers for Disease Control and Prevention (CDC). United States National Hospital Ambulatory Medical Care Survey 2003-2007. |
| Endocarditis | National Center for Health Statistics (NCHS), Centers for Disease Control and Prevention (CDC). United States National Hospital Ambulatory Medical Care Survey 2008-2011. |
| Hemorrhagic stroke | Thrift AG, Dewey HM, Sturm JW, Srikanth VK, Gilligan AK, Gall SL, Macdonell RAL, McNeil JJ, Donnan GA. Incidence of stroke subtypes in the North East Melbourne Stroke Incidence Study (NEMESIS): differences between men and women. Neuroepidemiology.... |
| Hemorrhagic stroke | Vaartjes I, Reitsma JB, de Bruin A, Berger-van Sijl M, Bos MJ, Breteler MM, Grobbee DE, Bots ML. Nationwide incidence of first stroke and TIA in the Netherlands. Eur J Neurol. 2008; 15(12): 1315-23. |
| Hemorrhagic stroke | Zhao D, Liu J, Wang W, Zeng Z, Cheng J, Liu J, Sun J, Wu Z. Epidemiological Transition of Stroke in China Twenty-One-Year Observational Study From the Sino-MONICA-Beijing Project. Stroke. 2008; 39(6): 1668-74. |
| Hemorrhagic stroke | Minelli C, Fu Fen L, Camara Minelli DP. Stroke Incidence, Prognosis, 30-Day, and 1-Year Case Fatality Rates in Matão, Brazil. Stroke. 2007; 38(11): 2906-11. |
| Hemorrhagic stroke | Corso G, Bottacchi E, Giardini G, De la Pierre F, Meloni T, Pesenti Campagnoni M, Ponzetti C, Veronese Morosini M. Community-based study of stroke incidence in the Valley of Aosta, Italy. CARe-cerebrovascular Aosta Registry: years 2004-2005. Neuroepid... |
| Hemorrhagic stroke | Kita Y, Turin TC, Ichikawa M, Sugihara H, Morita Y, Tomioka N, Rumana N, Okayama A, Nakamura Y, Abbott RD, Ueshima H. Trend of stroke incidence in a Japanese population: Takashima stroke registry, 1990-2001. Int J Stroke. 2009; 4(4): 241-9. |
| Hemorrhagic stroke | Ashok PP, Radhakrishnan K, Sridharan R, el-Mangoush MA. Incidence and pattern of cerebrovascular diseases in Benghazi, Libya. J Neurol Neurosurg Psychiatry. 1986; 49(5): 519-23. |
| Hemorrhagic stroke | Sacco S, Marini C, Toni D, Olivieri L, Carolei A. Incidence and 10-Year Survival of Intracerebral Hemorrhage in a Population-Based Registry. Stroke. 2009; 40(2): 394-9. |
| Hemorrhagic stroke | Maheswaran R, Pearson T, Smeeton NC, Beevers SD, Campbell MJ, Wolfe CD. Outdoor air pollution and incidence of ischemic and hemorrhagic stroke: a small-area level ecological study. Stroke. 2012; 43(1): 22-7. |
| Hemorrhagic stroke | Flynn RWV, MacDonald TM, Murray GD, Ferguson C, Shah K, Doney ASF. The Tayside Stroke Cohort: exploiting advanced regional medical informatics to create a region-wide database for studying the pharmacoepidemiology of stroke. Pharmacoepidemiol Drug Saf... |
| Hemorrhagic stroke | Hilmarsson A, Kjartansson O, Olafsson E. Incidence of First Stroke A Population Study in Iceland. Stroke. 2013; 44(6): 1714-6. |
| Hemorrhagic stroke | Béjot Y, Cordonnier C, Durier J, Aboa-Eboulé C, Rouaud O, Giroud M. Intracerebral haemorrhage profiles are changing: results from the Dijon population-based study. Brain. 2013; 136(Pt 2): 658-64. |
| Hemorrhagic stroke | Howard G, Cushman M, Howard VJ, Kissela BM, Kleindorfer DO, Moy CS, Switzer J, Woo D. Risk factors for intracerebral hemorrhage: the REasons for geographic and racial differences in stroke (REGARDS) study. Stroke. 2013; 44(5): 1282–7. |
| Hemorrhagic stroke | Béjot Y, Benzenine E, Lorgis L, Zeller M, Aubé H, Giroud M, Cottin Y, Quantin C. Comparative analysis of patients with acute coronary and cerebrovascular syndromes from the national French hospitalization health care system database. Neuroepidemiolo... |
| Hemorrhagic stroke | Leyden JM, Kleinig TJ, Newbury J, Castle S, Cranefield J, Anderson CS, Crotty M, Whitford D, Jannes J, Lee A, Greenhill J. Adelaide stroke incidence study: declining stroke rates but many preventable cardioembolic strokes. Stroke. 2013; 44(5): 122... |
| Hemorrhagic stroke | Irwin J, Wright P, Reeve P. Temporal trends and clinical characteristics of spontaneous intracerebral haemorrhage in the Waikato region of New Zealand: a hospital-based analysis. N Z Med J. 2011; 124(1345): 16-25. |
| Hemorrhagic stroke | Tveiten A, Ljøstad U, Mygland A, Thomassen L, Pripp AH, Naess H. Intracerebral hemorrhage in southern Norway – a hospital-based incidence study. Eur Neurol. 2012; 67(4): 240-5. |
| Hemorrhagic stroke | Azarpazhooh MR, Etemadi MM, Donnan GA, Mokhber N, Majdi MR, Ghayour-Mobarhan M, Ghandehary K, Farzadfard MT, Kiani R, Panahandeh M, Thrift AG. Excessive Incidence of Stroke in Iran. Stroke. 2010; 41(1): e3-e10. |
| Hemorrhagic stroke | Cabral NL, Gonçalves ARR, Longo AL, Moro CHC, Costa G, Amaral CH, Fonseca L a M, Eluf-Neto J. Incidence of stroke subtypes, prognosis and prevalence of risk factors in Joinville, Brazil: a 2 year community based study. J Neurol Neurosurg Psychiatry</... |
| Hemorrhagic stroke | Carolei A, Marini C, Di Napoli M, Di Gianfilippo G, Santalucia P, Baldassarre M, Giorgio De Matteis M, di Orio F. High Stroke Incidence in the Prospective Community-Based L'Aquila Registry (1994-1998): First Year's Results. Stroke. 1997; 28(12): 2... |
| Hemorrhagic stroke | Delbari A, Salman Roghani R, Tabatabaei SS, Rahgozar M, Lokk J. Stroke epidemiology and one-month fatality among an urban population in Iran. Int J Stroke. 2011; 6(3): 195-200. |
| Hemorrhagic stroke | Di Carlo A, Inzitari D, Galati F, Baldereschi M, Giunta V, Grillo G, Furch&igrave; A, Manno V, Naso F, Vecchio A, Consoli D. A Prospective Community-Based Study of Stroke in Southern Italy: The Vibo Valentia Incidence of Stroke Study (VISS). Cerebrova... |
| Hemorrhagic stroke | Earley CJ, Kittner SJ, Feeser BR, Gardner J, Epstein A, Wozniak MA, Wityk R, Stern BJ, Price TR, Macko RF, Johnson C, Sloan MA, Buchholz D. Stroke in children and sickle-cell disease: Baltimore-Washington Cooperative Young Stroke Study. Neurology.... |
| Hemorrhagic stroke | Ellekjær H, Holmen J, Indredavik B, Terent A. Epidemiology of Stroke in Innherred, Norway, 1994 to 1996: Incidence and 30-Day Case-Fatality Rate. Stroke. 1997; 28(11): 2180-4. |
| Hemorrhagic stroke | Feigin V, Carter K, Hackett M, Barber PA, McNaughton H, Dyall L, Chen M, Anderson C. Ethnic disparities in incidence of stroke subtypes: Auckland Regional Community Stroke Study, 2002-2003. Lancet Neurol. 2006; 5(2): 130-9. |
| Hemorrhagic stroke | Giroud M, Lemesle M, Gouyon JB, Nivelon JL, Milan C, Dumas R. Cerebrovascular disease in children under 16 years of age in the city of Dijon, France: a study of incidence and clinical features from 1985 to 1993. J Clin Epidemiol. 1995; 48(11): 134... |
| Hemorrhagic stroke | Hamad A, Hamad A, Sokrab TEO, Momeni S, Mesraoua B, Lingren A. Stroke in Qatar: A one-year, hospital-based study. J Stroke Cerebrovasc Dis. 2001; 10(5): 236-41. |
| Hemorrhagic stroke | Kita Y, Okayama A, Ueshima H, Wada M, Nozaki A, Choudhary SR, Bonita R, Inamoto Y, Kasamatsu T. Stroke incidence and case fatality in Shiga, Japan 1989-1993. Int J Epidemiol. 1999; 28(6): 1059-65. |
| Hemorrhagic stroke | Kolominsky-Rabas PL, Sarti C, Heuschmann PU, Graf C, Siemonsen S, Neundoerfer B, Katalinic A, Lang E, Gassmann K-G, von Stockert TR. A Prospective Community-Based Study of Stroke in Germany-The Erlangen Stroke Project (ESPro): Incidence and Case Fatality... |
| Hemorrhagic stroke | Lauria G, Gentile M, Fassetta G, Casetta I, Agnoli F, Andreotta G, Barp C, Caneve G, Cavallaro A, Cielo R, Mongillo D, Mosca M, Olivieri P. Incidence and Prognosis of Stroke in the Belluno Province, Italy: First-Year Results of a Community-Based Study. <... |
| Hemorrhagic stroke | Lavados PM, Sacks C, Prina L, Escobar A, Tossi C, Araya F, Feuerhake W, Galvez M, Salinas R, Alvarez G. Incidence, 30-day case-fatality rate, and prognosis of stroke in Iquique, Chile: a 2-year community-based prospective study (PISCIS project). Lance... |
| Hemorrhagic stroke | Manobianca G, Zoccolella S, Petruzzellis A, Miccoli A, Logroscino G. The incidence of major stroke subtypes in Southern Italy: a population based study. Eur J Neurol. 2010; 17(9): 1148-55. |
| Hemorrhagic stroke | Thrift AG, Dewey HM, Macdonell RAL, McNeil JJ, Donnan GA. Incidence of the Major Stroke Subtypes: Initial Findings From the North East Melbourne Stroke Incidence Study (NEMESIS). Stroke. 2001; 32(8): 1732-8. |
| Hemorrhagic stroke | Tsiskaridze A, Djibuti M, van Melle G, Lomidze G, Apridonidze S, Gauarashvili I, Piechowski-Józwiak B, Shakarishvili R, Bogousslavsky J. Stroke Incidence and 30-Day Case-Fatality in a Suburb of Tbilisi: Results of the First Prospective Population-Based ... |
| Hemorrhagic stroke | Sridharan SE, Unnikrishnan JP, Sukumaran S, Sylaja PN, Nayak SD, Sarma PS, Radhakrishnan K. Incidence, Types, Risk Factors, and Outcome of Stroke in a Developing Country. Stroke. 2009; 40(4): 1212-8. |
| Hemorrhagic stroke | Abdul-Ghaffar NU, el-Sonbaty MR, el-Din Abdul-Baky MS, Marafie AA, al-Said AM. Stroke in Kuwait: a three-year prospective study. Neuroepidemiology. 1997; 16(1): 40-7. |
| Hemorrhagic stroke | al-Rajeh S, Larbi EB, Bademosi O, Awada A, Yousef A, al-Freihi H, Miniawi H. Stroke register: experience from the eastern province of Saudi Arabia. Cerebrovasc Dis. 1998; 8(2): 86-9. |
| Hemorrhagic stroke | Al Rajeh S. Stroke in the Elderly Aged 75 Years and Above. Cerebrovasc Dis. 1994; 4(6): 402-6. |
| Hemorrhagic stroke | Anderson CS, Carter KN, Hackett ML, Feigin V, Barber PA, Broad JB, Bonita R. Trends in Stroke Incidence in Auckland, New Zealand, During 1981 to 2003. Stroke. 2005; 36(10): 2087-93. |
| Hemorrhagic stroke | Appelros P, Nydevik I, Seiger &Aring;ke, Ter&eacute;nt A. High Incidence Rates of Stroke in Orebro, Sweden: Further Support for Regional Incidence Differences within Scandinavia. Cerebrovasc Dis. 2002; 14(3-4): 161-8. |
| Hemorrhagic stroke | Awada A, Russell N, Al Rajeh S, Omojola M. Non-traumatic cerebral hemorrage in Saudi Arabs: a hospital-based study of 243 cases. J Neurol Sci. 1996; 144(1-2): 198-203. |
| Hemorrhagic stroke | Awada A. Stroke in Saudi Arabian young adults: a study of 120 cases. Acta Neurol Scand. 1994; 89(5): 323-8. |
| Hemorrhagic stroke | Bejot Y, Rouaud O, Durier J, Caillier M, Marie C, Freysz M, Yeguiayan J-M, Chantegret A, Osseby G, Moreau T, Giroud M. Decrease in the Stroke Case Fatality Rates in a French Population-Based Twenty-Year Study. Cerebrovasc Dis. 2007; 24(5): 439-44. |
| Hemorrhagic stroke | Hallström B, Jönsson A-C, Nerbrand C, Norrving B, Lindgren A. Stroke Incidence and Survival in the Beginning of the 21st Century in Southern Sweden: Comparisons With the Late 20th Century and Projections Into the Future. Stroke. 2008; 39(1): 10-5. |
| Hemorrhagic stroke | Börü UT, Oztürk E, Taşdemir M, Sur H. Living alone following first-ever stroke: a prospective study in Turkey identifying the risk factors and evaluating their effects. N Z Med J. 2007; 120(1255): U2559. |
| Hemorrhagic stroke | Correia M, Silva MR, Matos I, Magalhães R, Lopes JC, Ferro JM, Silva MC. Prospective Community-Based Study of Stroke in Northern Portugal: Incidence and Case Fatality in Rural and Urban Populations. Stroke. 2004; 35(9): 2048-53. |
| Hemorrhagic stroke | Dalal PM, Malik S, Bhattacharjee M, Trivedi ND, Vairale J, Bhat P, Deshmukh S, Khandelwal K, Mathur VD. Population-based stroke survey in Mumbai, India: incidence and 28-day case fatality. Neuroepidemiology. 2008; 31(4): 254-61. |
| Hemorrhagic stroke | Manobianca G, Zoccolella S, Petruzzellis A, Miccoli A, Logroscino G. Low Incidence of Stroke in Southern Italy: A Population-Based Study. Stroke. 2008; 39(11): 2923-8. |
| Hemorrhagic stroke | Immonen-Räihä P, Mähönen M, Tuomilehto J, Salomaa V, Kaarsalo E, Narva EV, Salmi K, Sarti C, Sivenius J, Alhainen K, Torppa J. Trends in Case-Fatality of Stroke in Finland During 1983 to 1992. Stroke. 1997; 28(12): 2493-9. |
| Hemorrhagic stroke | Kumral E, Ozkaya B, Sagduyu A, Sirin H, Vardarli E, Pehlivan M. The Ege Stroke Registry: A Hospital-Based Study in the Aegean Region, Izmir, Turkey. Cerebrovasc Dis. 1998; 8(5): 278-88. |
| Hemorrhagic stroke | Vibo R, Kõrv J, Roose M. The Third Stroke Registry in Tartu, Estonia: Decline of Stroke Incidence and 28-Day Case-Fatality Rate Since 1991. Stroke. 2005; 36(12): 2544-8. |
| Hemorrhagic stroke | Terént A. Trends in Stroke Incidence and 10-Year Survival in Söderhamn, Sweden, 1975-2001. Stroke. 2003; 34(6): 1353-8. |
| Hemorrhagic stroke | Thrift AG, Dewey HM, Macdonell RAL, McNeil JJ, Donnan GA. Stroke Incidence on the East Coast of Australia: The North East Melbourne Stroke Incidence Study (NEMESIS). Stroke. 2000; 31(9): 2087-92. |
| Hemorrhagic stroke | Wolfe CDA, Giroud M, Kolominsky-Rabas P, Dundas R, Lemesle M, Heuschmann P, Rudd A. Variations in Stroke Incidence and Survival in 3 Areas of Europe. Stroke. 2000; 31(9): 2074-9. |
| Hemorrhagic stroke | Pikija S, Cvetko D, Malojcic B, Trkanjec Z, Pavlicek I, Lukic A, Kopjar A, Hajduk M, Androvic A, Bilic-Genter M, Trkulja V. A population-based prospective 24-month study of stroke: incidence and 30-day case-fatality rates of first-ever strokes in Croatia... |
| Hemorrhagic stroke | Damasceno A, Gomes J, Azevedo A, Carrilho C, Lobo V, Lopes H, Madede T, Pravinrai P, Silva-Matos C, Jalla S, Stewart S, Lunet N. An Epidemiological Study of Stroke Hospitalizations in Maputo, Mozambique A High Burden of Disease in a Resource-Poor Country... |
| Hemorrhagic stroke | Vemmos KN, Bots ML, Tsibouris PK, Zis VP, Grobbee DE, Stranjalis GS, Stamatelopoulos S. Stroke Incidence and Case Fatality in Southern Greece: The Arcadia Stroke Registry. Stroke. 1999; 30(2): 363-70. |
| Hemorrhagic stroke | Bonita R, Broad JB, Beaglehole R. Changes in stroke incidence and case-fatality in Auckland, New Zealand, 1981-91. Lancet. 1993; 342(8885): 1470-3. |
| Hemorrhagic stroke | D'Alessandro G, Bottacchi E, Di Giovanni M, Martinazzo C, Sironi L, Lia C, Carenini L, Corso G, Gerbaz V, Polillo C, Pesenti Compagnoni M. Temporal trends of stroke in Valle d'Aosta, Italy. Incidence and 30-day fatality rates. Neurol Sci. 2000; 21... |
| Hemorrhagic stroke | Mihálka L, Smolanka V, Bulecza B, Mulesa S, Bereczki D. A Population Study of Stroke in West Ukraine: Incidence, Stroke Services, and 30-Day Case Fatality. Stroke. 2001; 32(10): 2227-31. |
| Hemorrhagic stroke | Morikawa Y, Nakagawa H, Naruse Y, Nishijo M, Miura K, Tabata M, Hirokawa W, Kagamimori S, Honda M, Yoshita K, Hayashi K. Trends in Stroke Incidence and Acute Case Fatality in a Japanese Rural Area: The Oyabe Study. Stroke. 2000; 31(7): 1583-7. |
| Hemorrhagic stroke | Smadja D, Cabre P, May F, Fanon J-L, René-Corail P, Riocreux C, Charpentier J-C, Fournerie P, Saint-Vil M, Ketterlé J. ERMANCIA: Epidemiology of Stroke in Martinique, French West Indies. Stroke. 2001; 32(12): 2741-7. |
| Hemorrhagic stroke | El Zunni S, Ahmed M, Prakash PS, Hassan KM. Stroke: Incidence and pattern in Benghazi, Libya. Ann Saudi Med. 1995; 15(4): 367-9. |
| Hemorrhagic stroke | Palm F, Urbanek C, Rose S, Buggle F, Bode B, Hennerici MG, Schmieder K, Inselmann G, Reiter R, Fleischer R, Piplack K-O, Safer A, Becher H, Grau AJ. Stroke Incidence and Survival in LuDisability Weightigshafen am Rhein, Germany: the LuDisability Weightigshafen Stroke Study (LuSSt). <... |
| Hemorrhagic stroke | Kôrv J, Roose M, Kaasik A-E. Changed Incidence and Case-Fatality Rates of First-Ever Stroke Between 1970 and 1993 in Tartu, Estonia. Stroke. 1996; 27(2): 199-203. |
| Hemorrhagic stroke | Musolino R LSP. First-ever stroke incidence and 30-day case fatality in the Sicilian Aeolian archipelago, Italy. Stroke. 2005; 36(12): 2738-41. |
| Hemorrhagic stroke | Walker R, Unwin N, Mugusi F, Swai M, Aris E, Jusabani A, Kabadi G, Gray W, Lewanga M, Alberti G, Whiting D. Stroke incidence in rural and urban Tanzania: a prospective, community-based study. Lancet Neurol. 2010; 9(8): 786-92. |
| Hemorrhagic stroke | Das SK, Banerjee TK, Biswas A, Roy T, Raut DK, Mukherjee CS, Chaudhuri A, Hazra A, Roy J. A Prospective Community-Based Study of Stroke in Kolkata, India. Stroke. 2007; 38(3): 906-10. |
| Hemorrhagic stroke | Matenga J. Stroke incidence rates among black residents of Harare - a prospective community-based study. S Afr Med J. 1997; 87(5): 606-8. |
| Hemorrhagic stroke | Sweileh WM, Sawalha AF, Al-Aqad SM, Zyoud SH, Al-Jabi SW. The Epidemiology of Stroke in Northern Palestine: A 1-Year, Hospital-Based Study. J Stroke Cerebrovasc Dis. 2008; 17(6): 406-11. |
| Hemorrhagic stroke | Zahuranec DB, Brown DL, Lisabeth LD, Morgenstern LB. Is it time for a large, collaborative study of pediatric stroke?. Stroke. 2005; 36(9): 1825-9. |
| Hemorrhagic stroke | Sienkiewicz-Jarosz H, Gluszkiewicz M, Pniewski J, Niewada M, Czlonkowska A, Wolfe C, Ryglewicz D. Incidence and case fatality rates of first-ever stroke - comparison of data from two prospective population-based studies conducted in Warsaw. Neurol Neu... |
| Hemorrhagic stroke | Wawrzynczyk M, Pierzchala K, Braczkowska B, Manka-Gaca I, Kumor K, Borowski D, Grodzicka-Zawisza L, Zejda J. Estimates of stroke incidence and case fatality in Zabrze, 2005-2006. Neurol Neurochir Pol. 2011; 45(1): 3-10. |
| Hemorrhagic stroke | Wieberdink RG, Ikram MA, Hofman A, Koudstaal PJ, Breteler MMB. Trends in stroke incidence rates and stroke risk factors in Rotterdam, the Netherlands from 1990 to 2008. Eur J Epidemiol. 2012; 27(4): 287-95. |
| Hemorrhagic stroke | Zhao Y, Yao Z, D'Souza W, Zhu C, Chun H, Zhuoga C, Zhang Q, Hu X, Zhou D. An Epidemiological Survey of Stroke in Lhasa, Tibet, China. Stroke. 2010; 41(12): 2739-43. |
| Hemorrhagic stroke | Hu HH, Sheng WY, Chu FL, Lan CF, Chiang BN. Incidence of stroke in Taiwan. Stroke. 1992; 23: 1237–1241. |
| Hemorrhagic stroke | Wu GX, Wu ZS, He BL. [Epidemiological characteristics of stroke in 16 provinces of China]. Nat Med J Chin. 1994; 74: 281–283. |
| Hemorrhagic stroke | Feigin VL, Wiebers DO, Nikitin YP, O'Fallon WM, Whisnant JP. Stroke Epidemiology in Novosibirsk, Russia: A Population-Based Study. Mayo Clin Proc. 1995; 70(9): 847-52. |
| Hemorrhagic stroke | Hong Y, Bots ML, Pan X, Hofman A, Grobbee DE, Chen H. Stroke Incidence and Mortality in Rural and Urban Shanghai From 1984 Through 1991: Findings From a Community-Based Registry. Stroke. 1994; 25(6): 1165-9. |
| Hemorrhagic stroke | Islam MS, Anderson CS, Hankey GJ, Hardie K, Carter K, Broadhurst R, Jamrozik K. Trends in Incidence and Outcome of Stroke in Perth, Western Australia During 1989 to 2001. Stroke. 2008; 39(3): 776-82. |
| Hemorrhagic stroke | Jorgensen HS, Plesner AM, Hubbe P, Larsen K. Marked increase of stroke incidence in men between 1972 and 1990 in Frederiksberg, Denmark. Stroke. 1992; 23(12): 1701-4. |
| Hemorrhagic stroke | Kulesh SD, Filina NA, Frantava NM, Zhytko NL, Kastsinevich TM, Kliatskova LA, Shumskas MS, Hilz MJ, Schwab S, Kolominsky-Rabas PL. Incidence and Case-Fatality of Stroke on the East Border of the European Union The Grodno Stroke Study. Stroke. 2010... |
| Hemorrhagic stroke | Numminen H, Kotila M, Waltimo O, Aho K, Kaste M. Declining Incidence and Mortality Rates of Stroke in Finland From 1972 to 1991: Results of Three Population-Based Stroke Registers. Stroke. 1996; 27(9): 1487-91. |
| Hemorrhagic stroke | Talaei M, Sarrafzadegan N, Sadeghi M, Oveisgharan S, Marshall T, Thomas GN, Iranipour R. Incidence of cardiovascular diseases in an Iranian population: the Isfahan Cohort Study. Arch Iran Med. 2013; 16(3): 138-44. |
| Hemorrhagic stroke | World Health Organization Regional Office for Europe (WHO/Europe). European Hospital Morbidity Database. Copenhagen, Denmark: World Health Organization Regional Office for Europe (WHO/Europe). |
| Hemorrhagic stroke | Federal Ministry of Health (Austria), Statistics Austria. Austria Hospital Inpatient Discharges 1989-1992. |
| Hemorrhagic stroke | Federal Ministry of Health (Austria), Statistics Austria. Austria Hospital Inpatient Discharges 1993-1997. |
| Hemorrhagic stroke | Federal Ministry of Health (Austria), Statistics Austria. Austria Hospital Inpatient Discharges 1998-2002. |
| Hemorrhagic stroke | Federal Ministry of Health (Austria), Statistics Austria. Austria Hospital Inpatient Discharges 2003-2007. |
| Hemorrhagic stroke | Ministry of Health (Brazil). Brazil Hospital Information System 1998-2002. |
| Hemorrhagic stroke | Ministry of Health (Brazil). Brazil Hospital Information System 2003-2007. |
| Hemorrhagic stroke | Ministry of Health (Brazil). Brazil Hospital Information System 2008-2012. |
| Hemorrhagic stroke | Ministry of Health (Brazil). Brazil Hospital Information System 2013-2014. |
| Hemorrhagic stroke | National Institute of Statistics and Censuses (Ecuador). Ecuador Hospital Inpatient Discharges 1993-1997. |
| Hemorrhagic stroke | National Institute of Statistics and Censuses (Ecuador). Ecuador Hospital Inpatient Discharges 1998-2002. |
| Hemorrhagic stroke | National Institute of Statistics and Censuses (Ecuador). Ecuador Hospital Inpatient Discharges 2003-2007. |
| Hemorrhagic stroke | National Institute of Statistics and Censuses (Ecuador). Ecuador Hospital Inpatient Discharges 2008-2012. |
| Hemorrhagic stroke | Ministry of Health (Mexico). Mexico Ministry of Health Hospital Discharges 2000-2002. |
| Hemorrhagic stroke | Ministry of Health (Mexico). Mexico Ministry of Health Hospital Discharges 2003-2007. |
| Hemorrhagic stroke | Ministry of Health (Mexico). Mexico Ministry of Health Hospital Discharges 2008-2012. |
| Hemorrhagic stroke | National Center for Health Statistics (NCHS), Centers for Disease Control and Prevention (CDC). United States National Hospital Ambulatory Medical Care Survey 1993-1997. |
| Hemorrhagic stroke | Norwegian Directorate of Health. Norway Patient Register 2008-2012. |
| Hemorrhagic stroke | National Center for Health Statistics (NCHS), Centers for Disease Control and Prevention (CDC). United States National Hospital Ambulatory Medical Care Survey 1998-2002. |
| Hemorrhagic stroke | Ministry of Health (New Zealand). New Zealand National Minimum Dataset 2000-2002. |
| Hemorrhagic stroke | Ministry of Health (New Zealand). New Zealand National Minimum Dataset 2003-2007. |
| Hemorrhagic stroke | Ministry of Health (New Zealand). New Zealand National Minimum Dataset 2008-2012. |
| Hemorrhagic stroke | Ministry of Health (New Zealand). New Zealand National Minimum Dataset 2013-2014. |
| Hemorrhagic stroke | National Board of Health and Welfare (Sweden). Sweden National Patient Register 1998-2002. |
| Hemorrhagic stroke | National Center for Health Statistics (NCHS), Centers for Disease Control and Prevention (CDC). United States National Hospital Ambulatory Medical Care Survey 2003-2007. |
| Hemorrhagic stroke | National Board of Health and Welfare (Sweden). Sweden National Patient Register 2003-2007. |
| Hemorrhagic stroke | National Board of Health and Welfare (Sweden). Sweden National Patient Register 2008-2012. |
| Hemorrhagic stroke | NHS England. United Kingdom - England Hospital Episode Statistics 2003-2008. |
| Hemorrhagic stroke | National Center for Health Statistics (NCHS), Centers for Disease Control and Prevention (CDC). United States National Hospital Ambulatory Medical Care Survey 2008-2011. |
| Hemorrhagic stroke | NHS England. United Kingdom - England Hospital Episode Statistics 2001-2003. |
| Hemorrhagic stroke | NHS England. United Kingdom - England Hospital Episode Statistics 2008-2012. |
| Hemorrhagic stroke | National Center for Health Statistics (NCHS), Centers for Disease Control and Prevention (CDC). United States National Hospital Discharge Survey 1988-1992. |
| Hemorrhagic stroke | National Center for Health Statistics (NCHS), Centers for Disease Control and Prevention (CDC). United States National Hospital Discharge Survey 1993-1997. |
| Hemorrhagic stroke | National Center for Health Statistics (NCHS), Centers for Disease Control and Prevention (CDC). United States National Hospital Discharge Survey 1998-2002. |
| Hemorrhagic stroke | National Center for Health Statistics (NCHS), Centers for Disease Control and Prevention (CDC). United States National Hospital Discharge Survey 2003-2007. |
| Hemorrhagic stroke | National Center for Health Statistics (NCHS), Centers for Disease Control and Prevention (CDC). United States National Hospital Discharge Survey 2008-2010. |
| Ischemic heart disease | Kim RB, Kim B-G, Kim Y-M, Seo JW, Lim YS, Kim HS, Lee HJ, Moon JY, Kim KY, Shin J-Y, Park H-K, Song J-K, Park K-S, Jeong BG, Park CG, Shin H-Y, Kang J-W, Oh G-J, Lee Y-H, Seong I-W, Yoo W-S, Hong Y-S. Trends in the Incidence of Hospitalized Acute Myocard... |
| Ischemic heart disease | Hurt RD, Weston SA, Ebbert JO, McNallan SM, Croghan IT, Schroeder DR, Roger VL. Myocardial infarction and sudden cardiac death in Olmsted County, Minnesota, before and after smoke-free workplace laws. Arch Intern Med. 2012; 172(21): 1635-41. |
| Ischemic heart disease | Waters A-M, Trinh L, Chau T, Bourchier M, Moon L. Latest statistics on cardiovascular disease in Australia. Clin Exp Pharmacol Physiol. 2013; 40(6): 347-56. |
| Ischemic heart disease | Hata J, Ninomiya T, Hirakawa Y, Nagata M, Mukai N, Gotoh S, Fukuhara M, Ikeda F, Shikata K, Yoshida D, Yonemoto K, Kamouchi M, Kitazono T, Kiyohara Y. Secular trends in cardiovascular disease and its risk factors in Japanese: half-century data from the H... |
| Ischemic heart disease | Ishikawa S, Kayaba K, Gotoh T, Nago N, Nakamura Y, Tsutsumi A, Kajii E. Incidence of Total Stroke, Stroke Subtypes, and Myocardial Infarction in the Japanese Population: The JMS Cohort Study. J Epidemiol. 2008; 18(4): 144-50. |
| Ischemic heart disease | Kubo M, Kiyohara Y, Kato I, Tanizaki Y, Arima H, Tanaka K, Nakamura H, Okubo K, Iida M. Trends in the Incidence, Mortality, and Survival Rate of Cardiovascular Disease in a Japanese Community. Stroke. 2003; 34(10): 2349-54. |
| Ischemic heart disease | Pilote L, Lavoie F, Ho V, Eisenberg MJ. Changes in the treatment and outcomes of acute myocardial infarction in Quebec, 1988-1995. CMAJ. 2000; 163(1): 31-6. |
| Ischemic heart disease | Quan H, Cujec B, Jin Y, Johnson D. Acute myocardial infarction in Alberta: temporal changes in outcomes, 1994 to 1999. Can J Cardiol. 2004; 20(2): 213-9. |
| Ischemic heart disease | Yue H, Gu D, Wu X, Yu X, Duan X, Wang J, Zheng R, Zhou J. [A 20-year prospective study on risk factors for myocardial infarction of 5,137 men in Capital Steel and Iron Company]. Chin J Prev Med. 2004; 38(1): 43-6. |
| Ischemic heart disease | Marrugat J, Elosua R, Martí H. Epidemiology of ischaemic heart disease in Spain: estimation of the number of cases and trends from 1997 to 2005. Rev Esp Cardiol. 2002; 55(4): 337-46. |
| Ischemic heart disease | Davies AR, Grundy E, Nitsch D, Smeeth L. Constituent country inequalities in myocardial infarction incidence and case fatality in men and women in the United Kingdom, 1996-2005. J Public Health (Oxf). 2011; 33(1): 131-8. |
| Ischemic heart disease | Hailer B, Naber C, Koslowski B, van Leeuwen P, Schäfer H, Budde T, Jacksch R, Sabin G, Erbel R, Myocardial Infarction Network Essen. Gender-related differences in patients with ST-elevation myocardial infarction: results from the registry study of the S... |
| Ischemic heart disease | Hardoon SL, Whincup PH, Petersen I, Capewell S, Morris RW. Trends in longer-term survival following an acute myocardial infarction and prescribing of evidenced-based medications in primary care in the UK from 1991: a longitudinal population-based study. ... |
| Ischemic heart disease | Katzenellenbogen JM, Sanfilippo FM, Hobbs MST, Briffa TG, Ridout SC, Knuiman MW, Dimer L, Taylor KP, Thompson PL, Thompson SC. Incidence of and case fatality following acute myocardial infarction in Aboriginal and non-Aboriginal Western Australians (2000... |
| Ischemic heart disease | Laks T, Jõeste E, Pullisaar O, Mäeots E, Lapidus I, Pietilä A, Salomaa V. Trends in incidence, attack rate, and mortality of acute myocardial infarction in Estonia: The Tallinn AMI Registry 1991-2005. Ann Med. 2013; 45(2): 107-11. |
| Ischemic heart disease | Morales A, Madrazo Y, Ramírez JI, Castañeda L, Machín W, Álvarez L, Angulo R, Dueñas R, Bermúdez R. Acute myocardial infarction incidence, mortality and case fatality in Santa Clara, Cuba, 2007-2008. MEDICC Rev. 2011; 13(4): 23-9. |
| Ischemic heart disease | Krotin M, Vasiljevic Z, Zdravkovic M, Milovanovic B. Gender differences in acute coronary syndrome in Serbia before organized primary PCI network service. Med Arh. 2010; 64(2): 94-7. |
| Ischemic heart disease | Gil M, Martí H, Elosúa R, Grau M, Sala J, Masiá R, Pérez G, Roset P, Bielsa O, Vila J, Marrugat J. [Analysis of trends in myocardial infarction case-fatality, incidence and mortality rates in Girona, Spain, 1990-1999]. Rev Esp Cardiol. 2007; 6... |
| Ischemic heart disease | Koopman C, Bots ML, van Oeffelen AAM, van Dis I, Verschuren WMM, Engelfriet PM, Capewell S, Vaartjes I. Population trends and inequalities in incidence and short-term outcome of acute myocardial infarction between 1998 and 2007. Int J Cardiol. 201... |
| Ischemic heart disease | Sans S, Puigdefábregas A, Paluzie G, Monterde D, Balaguer-Vintró I. Increasing trends of acute myocardial infarction in Spain: the MONICA-Catalonia Study. Eur Heart J. 2005; 26(5): 505-15. |
| Ischemic heart disease | Bakler T, Baburin A, Teesalu R, Rahu M. Comparison of management and 30-day mortality of acute myocardial infarction in men versus women in Estonia. Acta Cardiol. 2004; 59(3): 275-81. |
| Ischemic heart disease | Broda G, Rywik S, Kurjata P. Trends in myocardial infarction incidence and fatality in Warsaw Pol-MONICA population from 1984 to 1988. Int J Angiol. 1995; 4(2): 113-6. |
| Ischemic heart disease | Chaikhouni A, Chouhan L, Pomposiello C, Banna A, Mahrous F, Thomas G, Abu Al-Hassan N, Khalifa S, Jaddan A, Bsata MW, Hajar HA. Myocardial infarction in qatar: The first 2515 patients. Clin Cardiol. 1993; 16(3): 227-30. |
| Ischemic heart disease | Dan Lundblad LH. Gender differences in trends of acute myocardial infarction events: the Northern Sweden MONICA study 1985 - 2004. BMC Cardiovasc Disord. 2008; 8: 17. |
| Ischemic heart disease | Fukiyama K, Kimura Y, Wakugami K, Muratani H. Incidence and long-term prognosis of initial stroke and acute myocardial infarction in Okinawa, Japan. Hypertens Res. 2000; 23(2): 127-35. |
| Ischemic heart disease | Gafarov VV. [20-year monitoring of acute cardiovascular diseases in population of large industrial city in West Siberia (epidemiological study)]. Ter Arkh. 2000; 72(1): 15-21. |
| Ischemic heart disease | Gottlieb S, Harpaz D, Shotan A, Boyko V, Leor J, Cohen M, Mandelzweig L, Mazouz B, Stern S, Behar S. Sex Differences in Management and Outcome After Acute Myocardial Infarction in the 1990s?: A Prospective Observational Community-Based Study. Circulat... |
| Ischemic heart disease | Gupta SP, Khetrapaul NK. Incidence of acute myocardial infarction and sudden coronary death in Rohtak City. J Assoc Physicians India. 1981; 29(5): 365-72. |
| Ischemic heart disease | Lessa I, Cortes E, Souza JA, Souza Filho J, Netto JP, Almeida FA. Epidemiology of acute myocardial infarction in Salvador, Brazil: I. Incidence, lethality, and mortality. Bull Pan Am Health Organ. 1987; 21(1): 28-37. |
| Ischemic heart disease | Kuch B, Heier M, von Scheidt W, Kling B, Hoermann A, Meisinger C. 20-year trends in clinical characteristics, therapy and short-term prognosis in acute myocardial infarction according to presenting electrocardiogram: the MONICA/KORA AMI Registry (1985-20... |
| Ischemic heart disease | Mak K-H, Chia K-S, Kark JD, Chua T, Tan C, Foong B-H, Lim Y-L, Chew S-K. Ethnic differences in acute myocardial infarction in Singapore. Eur Heart J. 2003; 24(2): 151-60. |
| Ischemic heart disease | Skodová Z, Písa Z, Berka L, Cícha Z, Cerovská J, Emrová R, Hejl Z, Hrdlicková K, Hoke M, Pikhartová J. Myocardial Infarction Register in MONICA-Czechoslovakia Centre. Acta Med Scand Suppl. 1988; 79-83. |
| Ischemic heart disease | Sznajd J, Pajak A, Magdoń M, Misiowiec P, Malczewska-Malec M, Idzior-Waluś B, Celiński A, Baczyńska E. Pol-MONICA Cracow on-going study: initial findings. Acta Med Scand Suppl. 1988; 224(S728): 106-12. |
| Ischemic heart disease | Tunstall-Pedoe H, Kuulasmaa K, Mähönen M, Tolonen H, Ruokokoski E. Contribution of trends in survival and coronary-event rates to changes in coronary heart disease mortality: 10-year results from 37 WHO MONICA Project populations. Lancet. 1999; ... |
| Ischemic heart disease | National Public Health Institute (Finland), World Health Organization (WHO). MONICA Coronary Event Registration Data Book 1980-1995. Geneva, Switzerland: World Health Organization (WHO), 2000. |
| Ischemic heart disease | Barbados National Registry for Chronic Non-Communicable Disease. Barbados National Registry for Chronic Non-Communicable Disease Annual Report 2010. |
| Ischemic heart disease | Bata IR, Gregor RD, Eastwood BJ, Wolf HK. Trends in the incidence of acute myocardial infarction between 1984 and 1993 - The Halifax County MONICA Project. Can J Cardiol. 2000; 16(5): 589-95. |
| Ischemic heart disease | Heidenreich PA, McClellan M. Trends in treatment and outcomes for acute myocardial infarction: 1975-1995. Am J Med. 2001; 110(3): 165-74. |
| Ischemic heart disease | Kinjo K, Kimura Y, Shinzato Y, Tomori M, Komine Y, Kawazoe N, Takishita S, Fukiyama K. Incidence of stroke and acute myocardial infarction in subtropical islands, Okinawa, Japan. Am J Hypertens. 1991; 4(9): 793. |
| Ischemic heart disease | Roger VL, Weston SA, Gerber Y, Killian JM, Dunlay SM, Jaffe AS, Bell MR, Kors J, Yawn BP, Jacobsen SJ. Trends in Incidence, Severity, and Outcome of Hospitalized Myocardial Infarction. Circulation. 2010; 121(7): 863-9. |
| Ischemic heart disease | Yeh RW, Sidney S, Chandra M, Sorel M, Selby JV, Go AS. Population trends in the incidence and outcomes of acute myocardial infarction. N Engl J Med. 2010; 362(23): 2155-65. |
| Ischemic heart disease | Srimahachota S, Boonyaratavej S, Kanjanavanit R, Sritara P, Krittayaphong R, Kunjara-Na-ayudhya R, Tatsanavivat P, TR ACS Group. Thai Registry in Acute Coronary Syndrome (TRACS) – an extension of Thai Acute Coronary Syndrome registry (TACS) group: lowe... |
| Ischemic heart disease | Hopstock LA, Wilsgaard T, Njølstad I, Mannsverk J, Mathiesen EB, Løchen M-L, Bønaa KH. Seasonal variation in incidence of acute myocardial infarction in a sub-Arctic population: the Tromsø Study 1974-2004. Eur J Cardiovasc Prev Rehabil. 2011; ... |
| Ischemic heart disease | McManus DD, Piacentine SM, Lessard D, Gore JM, Yarzebski J, Spencer FA, Goldberg RJ. Thirty-Year (1975 to 2005) Trends in the Incidence Rates, Clinical Features, Treatment Practices, and Short-Term Outcomes of Patients <55 Years of Age Hospitalized With ... |
| Ischemic heart disease | Medina HM, Cannon CP, Zhao X, Hernandez AF, Bhatt DL, Peterson ED, Liang L, Fonarow GC. Quality of acute myocardial infarction care and outcomes in 33,997 patients aged 80 years or older: findings from Get With The Guidelines-Coronary Artery Disease (GWT... |
| Ischemic heart disease | Polonski L, Gasior M, Gierlotka M, Osadnik T, Kalarus Z, Trusz-Gluza M, Zembala M, Wilczek K, Lekston A, Zdrojewski T, Tendera M, PL-ACS Registry Pilot Group. A comparison of ST elevation versus non-ST elevation myocardial infarction outcomes in a large ... |
| Ischemic heart disease | Salam AM, Al BHA, Singh R, Gehani A, Asaad N, Al-Qahtani A, Suwaidi JA. Atrial fibrillation in Middle Eastern Arab and South Asian patients hospitalized with acute myocardial infarction: experience from a 20-year registry in qatar (1991-2010). Acta Ca... |
| Ischemic heart disease | Saposnik G, Redelmeier DA, Lu H, Fuller-Thomson E, Lonn E, Ray JG. Myocardial infarction associated with recency of immigration to Ontario. QJM. 2010; 103(4): 253-8. |
| Ischemic heart disease | Schmidt M, Jacobsen JB, Lash TL, Botker HE, Sorensen HT. 25 year trends in first time hospitalisation for acute myocardial infarction, subsequent short and long term mortality, and the prognostic impact of sex and comorbidity: a Danish nationwide cohort ... |
| Ischemic heart disease | Hishamuddin HM, Azmi NN, Jackson N. Acute myocardial infarction survival rate and complications after streptokinase therapy in Hospital Universiti Sains Malaysia, Kelantan--a comparative study. Singapore Med J. 1993; 34(4): 316-8. |
| Ischemic heart disease | Mule MF, Szajowicz D, Kevorkián R, Cohen G, Principato MB, Higa CC. Eventos clínicos en pacientes que consultan a la guardia por dolor precordial. Rev Argent Cardiol. 1997; 65(1): 1. |
| Ischemic heart disease | Pajak A. Myocardial infarction and complications. Longitudinal observation of a population of 280,000 women and men--Project POL-MONICA Krakow. I. Genesis and objectives of the WHO MONICA Project. Przegl Lek. 1996; 53(10): 703-6. |
| Ischemic heart disease | Varlamova T, Zhukovski G, Chazova L, Britov A. Monitoring of Major Cardiovascular Diseases in Moscow, USSR. Acta Med Scand Suppl. 1988; 224(S728): 73-8. |
| Ischemic heart disease | Yoshida M, Kita Y, Nakamura Y, Nozaki A, Okayama A, Sugihara H, Kasamatsu T, Hirose K, Kinoshita M, Ueshima H. Incidence of acute myocardial infarction in Takashima, Shiga, Japan. Circ J. 2005; 69(4): 404-8. |
| Ischemic heart disease | McManus DD, Gore J, Yarzebski J, Spencer F, Lessard D, Goldberg RJ. Recent trends in the incidence, treatment, and outcomes of patients with STEMI and NSTEMI. Am J Med. 2011; 124(1): 40-47. |
| Ischemic heart disease | Peltonen M, Lundberg V, Huhtasaari F, Asplund K. Marked improvement in survival after acute myocardial infarction in middle-aged men but not in women. The Northern Sweden MONICA study 1985-94. J Intern Med. 2000; 247(5): 579–587. |
| Ischemic heart disease | Hong JS, Kang HC, Lee SH. Comparison of case fatality rates for acute myocardial infarction in weekday vs weekend admissions in South Korea. Circ J. 2010; 74(3): 496–502. |
| Ischemic heart disease | Tousek P, Tousek F, Horak D, Cervinka P, Rokyta R, Pesl L, Jarkovsky J, Widimsky P, CZECH-2 Investigators. The incidence and outcomes of acute coronary syndromes in a central European country: results of the CZECH-2 registry. Int J Cardiol. 2014; ... |
| Ischemic heart disease | Shao Y-H, Croitor SK, Moreyra AE, Wilson AC, Kostis WJ, Cosgrove NM, Kostis JB, MIDAS Study Group (MIDAS II). Comparison of hospital versus out of hospital coronary death rates in women and men. Am J Cardiol. 2010; 106(1): 26–30. |
| Ischemic heart disease | Smolina K, Wright FL, Rayner M, Goldacre MJ. Determinants of the decline in mortality from acute myocardial infarction in England between 2002 and 2010: linked national database study. BMJ. 2012; 344(jan25 2): d8059. |
| Ischemic heart disease | Amagai Y, Ishikawa S, Gotoh T, Kayaba K, Nakamura Y, Kajii E. Sleep duration and incidence of cardiovascular events in a Japanese population: the Jichi Medical School cohort study. J Epidemiol. 2010; 20(2): 106–10. |
| Ischemic heart disease | Daida H, Miyauchi K, Ogawa H, Yokoi H, Matsumoto M, Kitakaze M, Kimura T, Matsubara T, Ikari Y, Kimura K, Tsukahara K, Origasa H, Morino Y, Tsutsui H, Kobayashi M, Isshiki T, PACIFIC investigators. Management and two-year long-term clinical outcome of ac... |
| Ischemic heart disease | Hung J, Brieger DB, Amerena JV, Coverdale SG, Rankin JM, Astley CM, Soman A, Chew DP. Treatment disparities and effect on late mortality in patients with diabetes presenting with acute myocardial infarction: observations from the ACACIA registry. Med ... |
| Ischemic heart disease | Igland J, Vollset SE, Nygård OK, Sulo G, Sulo E, Ebbing M, Næss Ø, Ariansen I, Tell GS. Educational inequalities in 28 day and 1-year mortality after hospitalisation for incident acute myocardial infarction--a nationwide cohort study. Int J Cardiol... |
| Ischemic heart disease | Kartalis A, Benetos G, Androulakis A, Didagelos M, Georgiadis I, Kosti A, Zotika C, Voutas P, Smyrnioudis N, Garoufalis S, Georgiopoulos G. Trends in acute myocardial infarction incidence and relative outcomes on a certain insular Mediterranean populatio... |
| Ischemic heart disease | Egiziano G, Akhtari S, Pilote L, Daskalopoulou SS, GENESIS (GENdEr and Sex DetermInants of Cardiovascular Disease) Investigators. Sex differences in young patients with acute myocardial infarction. Diabet Med. 2013; 30(3): e108–14. |
| Ischemic heart disease | O’Brien EC, Rose KM, Suchindran CM, Stürmer T, Chang PP, Chambless L, Guild CS, Rosamond WD. Medication, reperfusion therapy and survival in a community-based setting of hospitalised myocardial infarction. Heart. 2013; 99(11): 767–73. |
| Ischemic heart disease | Ortolani P, Marino M, Melandri G, Guastaroba P, Corzani A, Berti E, Rapezzi C, De Palma R, Branzi A. Recent temporal trends for first-time hospitalization for acute myocardial infarction. Treatment patterns and clinical outcome in a large cohort study. <... |
| Ischemic heart disease | Pagidipati NJ, Huffman MD, Jeemon P, Gupta R, Negi P, Jaison TM, Sharma S, Sinha N, Mohanan P, Muralidhara BG, Bijulal S, Sivasankaran S, Puri VK, Jose J, Reddy KS, Prabhakaran D. Association between gender, process of care measures, and outcomes in ACS ... |
| Ischemic heart disease | Piva e Mattos LAL, Berwanger O, Santos ES dos, Reis HJL, Romano ER, Petriz JLF, Sousa ACS, Neuenschwander FC, Guimarães JI, Andrade JP de. Clinical outcomes at 30 days in the Brazilian Registry of Acute Coronary Syndromes (ACCEPT). Arq Bras Cardiol</... |
| Ischemic heart disease | Sulo E, Vollset SE, Nygård O, Sulo G, Igland J, Egeland GM, Ebbing M, Tell GS. Trends in 28-day and 1-year mortality rates in patients hospitalized for a first acute myocardial infarction in Norway during 2001-2009: a “Cardiovascular disease in Norway... |
| Ischemic heart disease | Trzeciak P, Gierlotka M, Gąsior M, Osadnik T, Hawranek M, Lekston A, Zembala M, Poloński L. In-hospital and 12-month outcomes after acute coronary syndrome treatment in patients aged<40 years of age (from the Polish Registry of Acute Coronary Syndromes... |
| Ischemic heart disease | Khan NA, Grubisic M, Hemmelgarn B, Humphries K, King KM, Quan H. Outcomes after acute myocardial infarction in South Asian, Chinese, and white patients. Circulation. 2010; 122(16): 1570–7. |
| Ischemic heart disease | Nguyen HL, Saczynski JS, Gore JM, Waring ME, Lessard D, Yarzebski J, Reed G, Spencer FA, Li S, Goldberg RJ. Long-term trends in short-term outcomes in acute myocardial infarction. Am J Med. 2011; 124(10): 939–46. |
| Ischemic heart disease | Mak KH, Kark JD, Chia KS, Sim LL, Foong BH, Ding ZP, Kam R, Chew SK. Ethnic variations in female vulnerability after an acute coronary event. Heart. 2004; 90(6): 621-626. |
| Ischemic heart disease | Cox JL, Bata IR, Gregor RD, Johnstone DE, Wolf HK. Trends in event rate and case fatality of patients hospitalized with myocardial infarction between 1984 and 2001. Can J Physiol Pharmacol. 2006; 84(1): 121-7. |
| Ischemic heart disease | Beaglehole R, Bonita R, Jackson R, Stewart A, Sharpe N, Fraser GE. Trends in Coronary Heart Disease Event Rates in New Zealand. Am J Epidemiol. 1984; 120(2): 225-35. |
| Ischemic heart disease | Beck JA, Meisinger C, Heier M, Kuch B, Hörmann A, Greschik C, Koenig W. Effect of blood glucose concentrations on admission in non-diabetic versus diabetic patients with first acute myocardial infarction on short- and long-term mortality (from the MONIC... |
| Ischemic heart disease | Dobson AJ, McElduff P, Heller R, Alexander H, Colley P, D'Este K. Changing Patterns of Coronary Heart Disease in the Hunter Region of New South Wales, Australia. J Clin Epidemiol. 1999; 52(8): 761-71. |
| Ischemic heart disease | Grey C, Jackson R, Wells S, Marshall R, Riddell T, Kerr AJ. Twenty-eight day and one-year case fatality after hospitalisation with an acute coronary syndrome: a nationwide data linkage study. Aust N Z J Public Health. 2014; 38: 216-220. |
| Ischemic heart disease | Koek HL, de Bruin A, Gast A, Gevers E, Kardaun JWPF, Reitsma JB, Grobbee DE, Bots ML. Incidence of first acute myocardial infarction in the Netherlands. Neth J Med. 2007; 65(11): 434-41. |
| Ischemic heart disease | Máchová L, Janout V. The incidence of acute myocardial infarction in the district Olomouc in 1994--descriptive study. Cent Eur J Public Health. 1997; 5(3): 122-6. |
| Ischemic heart disease | Ergin A, Muntner P, Sherwin R, He J. Secular trends in cardiovascular disease mortality, incidence, and case fatality rates in adults in the United States. Am J Med. 2004; 117(4): 219-27. |
| Ischemic heart disease | McGovern PG, Jacobs DR, Shahar E, Arnett DK, Folsom AR, Blackburn H, Luepker RV. Trends in Acute Coronary Heart Disease Mortality, Morbidity, and Medical Care From 1985 Through 1997?: The Minnesota Heart Survey. Circulation. 2001; 104(1): 19-24. |
| Ischemic heart disease | Skodová Z, Písa Z, Hejl Z, Wiesner E, Cícha Z, Pikhartová J, Berka L, Vorlícek J, Emrová R, Vojtísek P. Coronary events in the population of six districts of the Czech Socialist Republic. Cor Vasa. 1988; 30(5): 324-30. |
| Ischemic heart disease | Wellenius GA, Mittleman MA. Disparities in myocardial infarction case fatality rates among the elderly: the 20-year Medicare experience. Am Heart J. 2008; 156(3): 483–490. |
| Ischemic heart disease | Brophy JM. The epidemiology of acute myocardial infarction and ischemic heart disease in Canada: data from 1976 to 1991. Can J Cardiol. 1997; 13(5): 474-8. |
| Ischemic heart disease | Capewell S, Murphy NF, MacIntyre K, Frame S, Stewart S, Chalmers JWT, Boyd J, Finlayson A, Redpath A, McMurray JJV. Short-term and long-term outcomes in 133,429 emergency patients admitted with angina or myocardial infarction in Scotland, 1990-2000: popu... |
| Ischemic heart disease | Smolina K, Wright FL, Rayner M, Goldacre MJ. Incidence and 30-day case fatality for acute myocardial infarction in England in 2010: national-linked database study. Eur J Public Health. 2012; 22(6): 848-53. |
| Ischemic heart disease | Dudas K, Lappas G, Rosengren A. Long-term prognosis after hospital admission for acute myocardial infarction from 1987 to 2006. Int J Cardiol. 2012; 155(3): 400-405. |
| Ischemic heart disease | García-García C, Subirana I, Sala J, Bruguera J, Sanz G, Valle V, Arós F, Fiol M, Molina L, Serra J, Marrugat J, Elosua R. Long-Term Prognosis of First Myocardial Infarction According to the Electrocardiographic Pattern (ST Elevation Myocardial Infarc... |
| Ischemic heart disease | Vaartjes I, van Dis I, Grobbee DE, Bots ML. The dynamics of mortality in follow-up time after an acute myocardial infarction, lower extremity arterial disease and ischemic stroke. BMC Cardiovasc Disord. 2010; 10: 57. |
| Ischemic heart disease | Kuch B, Wende R, Barac M, von Scheidt W, Kling B, Greschik C, Meisinger C. Prognosis and outcomes of elderly (75-84 years) patients with acute myocardial infarction 1-2 years after the event: AMI-elderly study of the MONICA/KORA Myocardial Infarction Reg... |
| Ischemic heart disease | Meisinger C, Heier M, von Scheidt W, Kirchberger I, Hörmann A, Kuch B. Gender-Specific short and long-term mortality in diabetic versus nondiabetic patients with incident acute myocardial infarction in the reperfusion era (the MONICA/KORA Myocardial Inf... |
| Ischemic heart disease | Langørgen J, Igland J, Vollset SE, Averina M, Nordrehaug JE, Tell GS, Irgens LM, Nygård O. Short-term and long-term case fatality in 11 878 patients hospitalized with a first acute myocardial infarction, 1979-2001: the Western Norway cardiovascular reg... |
| Ischemic heart disease | Lehto H-R, Lehto S, Havulinna AS, Ketonen M, Lehtonen A, Kesaniemi YA, Airaksinen KJ, Salomaa V. Sex differences in short- and long-term case-fatality of myocardial infarction. Eur J Epidemiol. 2011; 26: 851–861. |
| Ischemic heart disease | Manderbacka K, Hetemaa T, Keskimäki I, Luukkainen P, Koskinen S, Reunanen A. Are there socioeconomic differences in myocardial infarction event rates and fatality among patients with angina pectoris?. J Epidemiol Community Health. 2006; 60(5): 44... |
| Ischemic heart disease | Marshall RJ, Milne RJ, Lynn R, Jackson R. Quantifying the effect of age on short-term and long-term case fatality in 14,000 patients with incident cases of cardiovascular diseas. Eur J Cardiovasc Prev Rehabil. 2008; 15(2): 179-184. |
| Ischemic heart disease | Arciero TJ, Jacobsen SJ, Reeder GS, Frye RL, Weston SA, Killian JM, Roger Vr Vé. Temporal trends in the incidence of coronary disease. Am J Med. 2004; 117(4): 228-33. |
| Ischemic heart disease | Baba S, Iso H, Mannami T, Sasaki S, Okada K, Konishi M. Cigarette smoking and risk of coronary heart disease incidence among middle-aged Japanese men and women: the JPHC Study Cohort I. Eur J Cardiovasc Prev Rehabil. 2006; 13(2): 207-13. |
| Ischemic heart disease | Havulinna AS, Pääkkönen R, Karvonen M, Salomaa V. Geographic Patterns of Incidence of Ischemic Stroke and Acute Myocardial Infarction in Finland During 1991-2003. Ann Epidemiol. 2008; 18(3): 206-13. |
| Ischemic heart disease | Koopman C, van Oeffelen AAM, Bots ML, Engelfriet PM, Verschuren WMM, van Rossem L, van Dis I, Capewell S, Vaartjes I. Neighbourhood socioeconomic inequalities in incidence of acute myocardial infarction: a cohort study quantifying age- and gender-spec... |
| Ischemic heart disease | Wu Y, Yao C, Wu Z, Zhang R, Zhang M, Wu G, Zhao D, Hong Z. Interim report of Sino-Monica-Beijing for the years 1985-1989. Chin Med Sci J. 1992; 7(3): 125-9. |
| Ischemic heart disease | Ngoungou EB, Aboyans V, Kouna P, Makandja R, Ecke Nzengue JE, Allogho CN, Laskar M, Preux P-M, Lacroix P. Prevalence of cardiovascular disease in Gabon: a population study. Arch Cardiovasc Dis. 2012; 105(2): 77-83. |
| Ischemic heart disease | Talaei M, Sarrafzadegan N, Sadeghi M, Oveisgharan S, Marshall T, Thomas GN, Iranipour R. Incidence of cardiovascular diseases in an Iranian population: the Isfahan Cohort Study. Arch Iran Med. 2013; 16(3): 138-44. |
| Ischemic heart disease | Ebrahim S, Sung J, Song Y-M, Ferrer RL, Lawlor DA, Smith GD. Serum cholesterol, haemorrhagic stroke, ischaemic stroke, and myocardial infarction: Korean national health system prospective cohort study. BMJ. 2006; 333(7557): 22-30. |
| Ischemic heart disease | Kucharska-Newton AM, Harald K, Rosamond WD, Rose KM, Rea TD, Salomaa V. Socioeconomic Indicators and the Risk of Acute Coronary Heart Disease Events: Comparison of Population-Based Data from the United States and Finland. Ann Epidemiol. 2011; 21(8... |
| Ischemic heart disease | Goldberg RJ, Yarzebski J, Lessard D, Gore JM. A two-decades (1975 to 1995) long experience in the incidence, in-hospital and long-term case-fatality rates of acute myocardial infarction: a community-wide perspective. J Am Coll Cardiol. 1999; 33(6)... |
| Ischemic heart disease | Lampe F, Morris R, Whincup P, Walker M, Ebrahim S, Shaper A. Is the prevalence of coronary heart disease falling in British men?&nbsp;Heart. 2001; 86(5): 499-505. |
| Ischemic heart disease | Onat A, Senocak MS, Surdum-Avci G, Ornek E. Prevalence of coronary heart disease in Turkish adults. Int J Cardiol. 1993; 39(1): 23-31. |
| Ischemic heart disease | Piscitelli P, Iolascon G, Greco M, Marinelli A, Gimigliano F, Gimigliano R, Gisonni P, Giordano A, Migliore A, Granata M, Tarantino U, Brandi ML, Baggiani A, Distante A. The occurrence of acute myocardial infarction in Italy: a five-year analysis of hosp... |
| Ischemic heart disease | Ministry of Health (Mexico). Mexico Ministry of Health Hospital Discharges 2000-2002. |
| Ischemic heart disease | Ministry of Health (Mexico). Mexico Ministry of Health Hospital Discharges 2003-2007. |
| Ischemic heart disease | Ministry of Health (Mexico). Mexico Ministry of Health Hospital Discharges 2008-2012. |
| Ischemic heart disease | Norwegian Directorate of Health. Norway Patient Register 2008-2012. |
| Ischemic heart disease | Ministry of Health (New Zealand). New Zealand National Minimum Dataset 2000-2002. |
| Ischemic heart disease | Ministry of Health (New Zealand). New Zealand National Minimum Dataset 2003-2007. |
| Ischemic heart disease | Ministry of Health (New Zealand). New Zealand National Minimum Dataset 2008-2012. |
| Ischemic heart disease | Ministry of Health (New Zealand). New Zealand National Minimum Dataset 2013-2014. |
| Ischemic heart disease | National Board of Health and Welfare (Sweden). Sweden National Patient Register 1998-2002. |
| Ischemic heart disease | National Board of Health and Welfare (Sweden). Sweden National Patient Register 2003-2007. |
| Ischemic heart disease | National Board of Health and Welfare (Sweden). Sweden National Patient Register 2008-2012. |
| Ischemic heart disease | NHS England. United Kingdom - England Hospital Episode Statistics 2003-2008. |
| Ischemic heart disease | NHS England. United Kingdom - England Hospital Episode Statistics 2001-2003. |
| Ischemic heart disease | NHS England. United Kingdom - England Hospital Episode Statistics 2008-2012. |
| Ischemic heart disease | National Center for Health Statistics (NCHS), Centers for Disease Control and Prevention (CDC). United States National Hospital Discharge Survey 1988-1992. |
| Ischemic heart disease | National Center for Health Statistics (NCHS), Centers for Disease Control and Prevention (CDC). United States National Hospital Discharge Survey 1993-1997. |
| Ischemic heart disease | National Center for Health Statistics (NCHS), Centers for Disease Control and Prevention (CDC). United States National Hospital Discharge Survey 1998-2002. |
| Ischemic heart disease | National Center for Health Statistics (NCHS), Centers for Disease Control and Prevention (CDC). United States National Hospital Discharge Survey 2003-2007. |
| Ischemic heart disease | National Center for Health Statistics (NCHS), Centers for Disease Control and Prevention (CDC). United States National Hospital Discharge Survey 2008-2010. |
| Ischemic heart disease | World Health Organization Regional Office for Europe (WHO/Europe). European Hospital Morbidity Database. Copenhagen, Denmark: World Health Organization Regional Office for Europe (WHO/Europe). |
| Ischemic heart disease | Federal Ministry of Health (Austria), Statistics Austria. Austria Hospital Inpatient Discharges 1989-1992. |
| Ischemic heart disease | Federal Ministry of Health (Austria), Statistics Austria. Austria Hospital Inpatient Discharges 1993-1997. |
| Ischemic heart disease | Federal Ministry of Health (Austria), Statistics Austria. Austria Hospital Inpatient Discharges 1998-2002. |
| Ischemic heart disease | Federal Ministry of Health (Austria), Statistics Austria. Austria Hospital Inpatient Discharges 2003-2007. |
| Ischemic heart disease | Ministry of Health (Brazil). Brazil Hospital Information System 1998-2002. |
| Ischemic heart disease | Ministry of Health (Brazil). Brazil Hospital Information System 2003-2007. |
| Ischemic heart disease | Ministry of Health (Brazil). Brazil Hospital Information System 2008-2012. |
| Ischemic heart disease | Ministry of Health (Brazil). Brazil Hospital Information System 2013-2014. |
| Ischemic heart disease | National Institute of Statistics and Censuses (Ecuador). Ecuador Hospital Inpatient Discharges 1993-1997. |
| Ischemic heart disease | National Institute of Statistics and Censuses (Ecuador). Ecuador Hospital Inpatient Discharges 1998-2002. |
| Ischemic heart disease | National Institute of Statistics and Censuses (Ecuador). Ecuador Hospital Inpatient Discharges 2003-2007. |
| Ischemic heart disease | National Institute of Statistics and Censuses (Ecuador). Ecuador Hospital Inpatient Discharges 2008-2012. |
| Ischemic heart disease | National Center for Health Statistics (NCHS), Centers for Disease Control and Prevention (CDC). United States National Hospital Ambulatory Medical Care Survey 1993-1997. |
| Ischemic heart disease | National Center for Health Statistics (NCHS), Centers for Disease Control and Prevention (CDC). United States National Hospital Ambulatory Medical Care Survey 1998-2002. |
| Ischemic heart disease | National Center for Health Statistics (NCHS), Centers for Disease Control and Prevention (CDC). United States National Hospital Ambulatory Medical Care Survey 2003-2007. |
| Ischemic heart disease | National Center for Health Statistics (NCHS), Centers for Disease Control and Prevention (CDC). United States National Hospital Ambulatory Medical Care Survey 2008-2011. |
| Ischemic heart disease | Ford ES, Giles WH, Croft JB. Prevalence of nonfatal coronary heart disease among American adults. Am Heart J. 2000; 139(3): 371-7. |
| Ischemic heart disease | Konstantinov VV, Zhukovskiĭ GS, Konstantinova OS, Timofeeva TN, Kapustina AV, Olfer'ev AM, Deev AD. [The dynamics of ischemic heart disease and the risk factors among the male population of Moscow in the 1985 to 1995 period]. Ter Arkh. 1997; 69(1... |
| Ischemic heart disease | Sarraf-Zadegan N, Sayed-Tabatabaei FA, Bashardoost N, Maleki A, Totonchi M, Habibi HR, Sotodehmaram E, Tafazoli F, Karimi A. The prevalence of coronary artery disease in an urban population in Isfahan, Iran. Acta Cardiol. 1999; 54(5): 257-63. |
| Ischemic heart disease | Mozaffarian D, Bryson CL, Spertus JA, McDonell MB, Fihn SD. Anginal symptoms consistently predict total mortality among outpatients with coronary artery disease. Am Heart J. 2003; 146(6): 1015-22. |
| Ischemic heart disease | Reklaĭtene RA, Baubinene AV, Domarkene SB, Matsiavichiute NA, Tamoshiunas AA, Iurenene KS. [Prevalence of risk factors and indicators of mortality among males 40-59 years of age with various forms of ischemic heart disease (data of a 5-year prospective ... |
| Ischemic heart disease | Iurenev AP, Lediashova GA, Lupanov VP, Rubanovich AI, Zhukova IM. Results of a 10-year prospective study of patients with ischemic heart disease. Kardiol Pol. 1990; 30(6): 47-51. |
| Ischemic heart disease | Zaman MJS, Loret de Mola C, Gilman RH, Smeeth L, Miranda JJ. The prevalence of angina symptoms and association with cardiovascular risk factors, among rural, urban and rural to urban migrant populations in Peru. BMC Cardiovasc Disord. 2010; 10: 50. |
| Ischemic heart disease | Shiue I, Hristova K. Geographic variations in prevalent cardiovascular disease subtypes: UK Understanding Society cohort, 2009-2010. Int J Cardiol. 2014; 171(3): e81-83. |
| Ischemic heart disease | Hemingway H, Shipley M, Britton A, Page M, Macfarlane P, Marmot M. Prognosis of angina with and without a diagnosis: 11 year follow up in the Whitehall II prospective cohort study. BMJ. 2003; 327(7420): 895-900. |
| Ischemic heart disease | Chow C, Cardona M, Raju PK, Iyengar S, Sukumar A, Raju R, Colman S, Madhav P, Raju R, Reddy KS, Celermajer D, Neal B. Cardiovascular disease and risk factors among 345 adults in rural India--the Andhra Pradesh Rural Health Initiative. Int J Cardiol</i... |
| Ischemic heart disease | Glader EL, Stegmayr B. Declining prevalence of angina pectoris in middle-aged men and women A population-based study within the Northern Sweden MONICA Project Multinational Monitoring of Trends and Cardiovascular Disease. J Intern Med. 1999; 246(3... |
| Ischemic heart disease | Murphy NF, Stewart S, Hart CL, MacIntyre K, Hole D, McMurray JJV. A population study of the long-term consequences of Rose angina: 20-year follow-up of the Renfrew-Paisley study. Heart. 2006; 92(12): 1739-46. |
| Ischemic heart disease | National Center for Statistics and Economic Studies (Congo, Rep.). Congo, Rep. Household Survey for Poverty Assessment 2005. |
| Ischemic heart disease | National Center for Health Statistics (NCHS), Centers for Disease Control and Prevention (CDC). United States National Health and Nutrition Examination Survey 2001-2002. Hyattsville, United States: National Center for Health Statistics (NCHS), Centers... |
| Ischemic heart disease | National Center for Health Statistics (NCHS), Centers for Disease Control and Prevention (CDC). United States National Health and Nutrition Examination Survey 2005-2006. Hyattsville, United States: National Center for Health Statistics (NCHS), Centers... |
| Ischemic heart disease | National Center for Health Statistics (NCHS), Centers for Disease Control and Prevention (CDC). United States National Health and Nutrition Examination Survey 2007-2008. Hyattsville, United States: National Center for Health Statistics (NCHS), Centers... |
| Ischemic heart disease | National Center for Health Statistics (NCHS), Centers for Disease Control and Prevention (CDC). United States National Health and Nutrition Examination Survey 2009-2010. Hyattsville, United States: National Center for Health Statistics (NCHS), Centers... |
| Ischemic heart disease | Centers for Disease Control and Prevention (CDC). United States Behavioral Risk Factor Surveillance System 2005. Atlanta, Georgia: CDC, US Department of Health and Human Services. |
| Ischemic heart disease | Centers for Disease Control and Prevention (CDC). United States Behavioral Risk Factor Surveillance System 2006. Atlanta, Georgia: CDC, US Department of Health and Human Services. |
| Ischemic heart disease | Centers for Disease Control and Prevention (CDC). United States Behavioral Risk Factor Surveillance System 2007. Atlanta, Georgia: CDC, US Department of Health and Human Services. |
| Ischemic heart disease | Centers for Disease Control and Prevention (CDC). United States Behavioral Risk Factor Surveillance System 2008. Atlanta, Georgia: CDC, US Department of Health and Human Services. |
| Ischemic heart disease | Centers for Disease Control and Prevention (CDC). United States Behavioral Risk Factor Surveillance System 2009. Atlanta, Georgia: CDC, US Department of Health and Human Services. |
| Ischemic heart disease | Centers for Disease Control and Prevention (CDC). United States Behavioral Risk Factor Surveillance System 2010. Atlanta, United States: Centers for Disease Control and Prevention (CDC). |
| Ischemic heart disease | Agency for Healthcare Research and Quality. United States Medical Expenditure Panel Survey 2002-2009. Rockville, United States: Agency for Healthcare Research and Quality. |
| Ischemic heart disease | World Health Organization (WHO). United Arab Emirates World Health Survey 2003. Geneva, Switzerland: World Health Organization (WHO), 2005. |
| Ischemic heart disease | Department of Health and Ageing (Australia), World Health Organization (WHO). Australia World Health Survey 2003. Geneva, Switzerland: World Health Organization (WHO), 2005. |
| Ischemic heart disease | World Health Organization (WHO). Burkina Faso World Health Survey 2002-2003. Geneva, Switzerland: World Health Organization (WHO), 2005. |
| Ischemic heart disease | World Health Organization (WHO). Bangladesh World Health Survey 2003. Geneva, Switzerland: World Health Organization (WHO), 2005. |
| Ischemic heart disease | World Health Organization (WHO). Bosnia and Herzegovina World Health Survey 2003. Geneva, Switzerland: World Health Organization (WHO), 2005. |
| Ischemic heart disease | Center for Scientific and Technological Information, Oswaldo Cruz Foundation and World Health Organization (WHO). Brazil World Health Survey 2003. Geneva, Switzerland: World Health Organization (WHO), 2005. |
| Ischemic heart disease | World Health Organization (WHO). China World Health Survey 2002. Geneva, Switzerland: World Health Organization (WHO), 2005. |
| Ischemic heart disease | World Health Organization (WHO). Côte d'Ivoire World Health Survey 2003. Geneva, Switzerland: World Health Organization (WHO), 2005. |
| Ischemic heart disease | World Health Organization (WHO). Congo, Rep. World Health Survey 2003. Geneva, Switzerland: World Health Organization (WHO), 2005. |
| Ischemic heart disease | World Health Organization (WHO). Comoros World Health Survey 2003. Geneva, Switzerland: World Health Organization (WHO), 2005. |
| Ischemic heart disease | World Health Organization (WHO). Czech Republic World Health Survey 2002-2003. Geneva, Switzerland: World Health Organization (WHO), 2005. |
| Ischemic heart disease | World Health Organization (WHO). Dominican Republic World Health Survey 2003. Geneva, Switzerland: World Health Organization (WHO), 2005. |
| Ischemic heart disease | World Health Organization (WHO). Ecuador World Health Survey 2003. Geneva, Switzerland: World Health Organization (WHO), 2005. |
| Ischemic heart disease | World Health Organization (WHO). Spain World Health Survey 2002-2003. Geneva, Switzerland: World Health Organization (WHO), 2005. |
| Ischemic heart disease | World Health Organization (WHO). Estonia World Health Survey 2003. Geneva, Switzerland: World Health Organization (WHO), 2005. |
| Ischemic heart disease | World Health Organization (WHO). Ethiopia World Health Survey 2003. Geneva, Switzerland: World Health Organization (WHO), 2005. |
| Ischemic heart disease | World Health Organization (WHO). Finland World Health Survey 2004. Geneva, Switzerland: World Health Organization (WHO), 2005. |
| Ischemic heart disease | World Health Organization (WHO). France World Health Survey 2003. Geneva, Switzerland: World Health Organization (WHO), 2005. |
| Ischemic heart disease | World Health Organization (WHO). Georgia World Health Survey 2003. Geneva, Switzerland: World Health Organization (WHO), 2005. |
| Ischemic heart disease | World Health Organization (WHO). Ghana World Health Survey 2003. Geneva, Switzerland: World Health Organization (WHO), 2005. |
| Ischemic heart disease | World Health Organization (WHO). Croatia World Health Survey 2003. Geneva, Switzerland: World Health Organization (WHO), 2005. |
| Ischemic heart disease | World Health Organization (WHO). Hungary World Health Survey 2003. Geneva, Switzerland: World Health Organization (WHO), 2005. |
| Ischemic heart disease | International Institute for Population Sciences (India), World Health Organization (WHO). India World Health Survey 2003. Geneva, Switzerland: World Health Organization (WHO), 2005. |
| Ischemic heart disease | World Health Organization (WHO). Ireland World Health Survey 2003. Geneva, Switzerland: World Health Organization (WHO), 2005. |
| Ischemic heart disease | World Health Organization (WHO). Israel World Health Survey 2003. Geneva, Switzerland: World Health Organization (WHO), 2005. |
| Ischemic heart disease | World Health Organization (WHO). Kenya World Health Survey 2004. Geneva, Switzerland: World Health Organization (WHO), 2005. |
| Ischemic heart disease | World Health Organization (WHO). Laos World Health Survey 2003. |
| Ischemic heart disease | World Health Organization (WHO). Sri Lanka World Health Survey 2003. Geneva, Switzerland: World Health Organization (WHO), 2005. |
| Ischemic heart disease | World Health Organization (WHO). Luxembourg World Health Survey 2003. Geneva, Switzerland: World Health Organization (WHO), 2005. |
| Ischemic heart disease | World Health Organization (WHO). Latvia World Health Survey 2003. Geneva, Switzerland: World Health Organization (WHO), 2005. |
| Ischemic heart disease | World Health Organization (WHO). Morocco World Health Survey 2003. Geneva, Switzerland: World Health Organization (WHO), 2005. |
| Ischemic heart disease | World Health Organization (WHO). Mexico World Health Survey 2002-2003. Geneva, Switzerland: World Health Organization (WHO), 2005. |
| Ischemic heart disease | World Health Organization (WHO). Mali World Health Survey 2003. Geneva, Switzerland: World Health Organization (WHO), 2005. |
| Ischemic heart disease | World Health Organization (WHO). Myanmar World Health Survey 2003. Geneva, Switzerland: World Health Organization (WHO), 2005. |
| Ischemic heart disease | World Health Organization (WHO). Mauritania World Health Survey 2003. Geneva, Switzerland: World Health Organization (WHO), 2005. |
| Ischemic heart disease | World Health Organization (WHO). Mauritius World Health Survey 2003. Geneva, Switzerland: World Health Organization (WHO), 2005. |
| Ischemic heart disease | World Health Organization (WHO). Malawi World Health Survey 2003. Geneva, Switzerland: World Health Organization (WHO), 2005. |
| Ischemic heart disease | World Health Organization (WHO). Malaysia World Health Survey 2003. Geneva, Switzerland: World Health Organization (WHO), 2005. |
| Ischemic heart disease | World Health Organization (WHO). Namibia World Health Survey 2003. Geneva, Switzerland: World Health Organization (WHO), 2005. |
| Ischemic heart disease | World Health Organization (WHO). Norway World Health Survey 2003. Geneva, Switzerland: World Health Organization (WHO), 2005. |
| Ischemic heart disease | World Health Organization (WHO). Nepal World Health Survey 2003. Geneva, Switzerland: World Health Organization (WHO), 2005. |
| Ischemic heart disease | World Health Organization (WHO). Pakistan World Health Survey 2003-2004. Geneva, Switzerland: World Health Organization (WHO), 2005. |
| Ischemic heart disease | World Health Organization (WHO). Philippines World Health Survey 2003. Geneva, Switzerland: World Health Organization (WHO), 2005. |
| Ischemic heart disease | World Health Organization (WHO). Portugal World Health Survey 2003. Geneva, Switzerland: World Health Organization (WHO), 2006. |
| Ischemic heart disease | World Health Organization (WHO). Paraguay World Health Survey 2002-2003. Geneva, Switzerland: World Health Organization (WHO), 2005. |
| Ischemic heart disease | World Health Organization (WHO). Russia World Health Survey 2003. Geneva, Switzerland: World Health Organization (WHO), 2005. |
| Ischemic heart disease | World Health Organization (WHO). Senegal World Health Survey 2003. Geneva, Switzerland: World Health Organization (WHO), 2005. |
| Ischemic heart disease | World Health Organization (WHO). Slovakia World Health Survey 2003. Geneva, Switzerland: World Health Organization (WHO), 2005. |
| Ischemic heart disease | World Health Organization (WHO). Sweden World Health Survey 2003. Geneva, Switzerland: World Health Organization (WHO), 2005. |
| Ischemic heart disease | World Health Organization (WHO). Swaziland World Health Survey 2003. Geneva, Switzerland: World Health Organization (WHO), 2005. |
| Ischemic heart disease | World Health Organization (WHO). Chad World Health Survey 2003. Geneva, Switzerland: World Health Organization (WHO), 2005. |
| Ischemic heart disease | World Health Organization (WHO). Tunisia World Health Survey 2003. Geneva, Switzerland: World Health Organization (WHO), 2005. |
| Ischemic heart disease | World Health Organization (WHO). Ukraine World Health Survey 2002-2003. Geneva, Switzerland: World Health Organization (WHO), 2005. |
| Ischemic heart disease | World Health Organization (WHO). Uruguay World Health Survey 2002-2003. Geneva, Switzerland: World Health Organization (WHO), 2005. |
| Ischemic heart disease | World Health Organization (WHO). Vietnam World Health Survey 2002-2003. Geneva, Switzerland: World Health Organization (WHO), 2005. |
| Ischemic heart disease | World Health Organization (WHO). South Africa World Health Survey 2002-2003. Geneva, Switzerland: World Health Organization (WHO), 2005. |
| Ischemic heart disease | World Health Organization (WHO). Zimbabwe World Health Survey 2003. Geneva, Switzerland: World Health Organization (WHO), 2005. |
| Ischemic heart disease | National Center for Health Statistics (NCHS), Centers for Disease Control and Prevention (CDC). United States National Health and Nutrition Examination Survey 2011-2012. Hyattsville, United States: National Center for Health Statistics (NCHS), Centers fo... |
| Ischemic heart disease | National Institute of Public Health (Mexico), World Health Organization (WHO). Mexico WHO Study on Global AGEing and Adult Health 2009-2010. Geneva, Switzerland: World Health Organization (WHO), 2011. |
| Ischemic heart disease | Lampe FC, Whincup PH, Wannamethee SG, Shaper AG, Walker M, Ebrahim S. The natural history of prevalent ischaemic heart disease in middle-aged men. Eur Heart J. 2000; 21(13): 1052-62. |
| Ischemic heart disease | Hemingway H, McCallum A, Shipley M, Manderbacka K, Martikainen P, Keskimäki I. Incidence and Prognostic Implications of Stable Angina Pectoris Among Women and Men. JAMA. 2006; 295(12): 1404-11. |
| Ischemic heart disease | Harvard University Medical School. United States National Comorbidity Survey: Replication 12-Month Prevalence Estimates 2001-2003. Boston, United States: Harvard University Medical School. |
| Ischemic heart disease | National Center for Health Statistics (NCHS), Centers for Disease Control and Prevention (CDC). United States National Health and Nutrition Examination Survey 2003-2004. Hyattsville, United States: National Center for Health Statistics (NCHS), Centers... |
| Ischemic heart disease | Cobiac LJ, Magnus A, Lim S, Barendregt JJ, Carter R, Vos T. Which interventions offer best value for money in primary prevention of cardiovascular disease?. PLoS One. 2012; 7(7): e41842. |
| Ischemic heart disease | Parikh NI, Gona P, Larson MG, Fox CS, Benjamin EJ, Murabito JM, O'Donnell CJ, Vasan RS, Levy D. Long-Term Trends in Myocardial Infarction Incidence and Case Fatality in the National Heart, Lung, and Blood Institute's Framingham Heart Study. Circulatio... |
| Ischemic heart disease | Jones DA, Rathod KS, Sekhri N, Junghans C, Gallagher S, Rothman MT, Mohiddin S, Kapur A, Knight C, Archbold A, Jain AK, Mills PG, Uppal R, Mathur A, Timmis AD, Wragg A. Case fatality rates for South Asian and Caucasian patients show no difference 2.5 yea... |
| Ischemic heart disease | Buch P, Rasmussen S, Gislason GH, Rasmussen JN, Køber L, Gadsbøll N, Stender S, Madsen M, Torp-Pedersen C, Abildstrom SZ. Temporal decline in the prognostic impact of a recurrent acute myocardial infarction 1985 to 2002. Heart. 2007; 93(2): 210... |
| Ischemic heart disease | Yusuf S, Rangarajan S, Teo K, Islam S, Li W, Liu L, Bo J, Lou Q, Lu F, Liu T, Yu L, Zhang S, Mony P, Swaminathan S, Mohan V, Gupta R, Kumar R, Vijayakumar K, Lear S, Anand S, Wielgosz A, Diaz R, Avezum A, Lopez-Jaramillo P, Lanas F, Yusoff K, Ismail N, I... |
| Ischemic stroke | Thrift AG, Dewey HM, Sturm JW, Srikanth VK, Gilligan AK, Gall SL, Macdonell RAL, McNeil JJ, Donnan GA. Incidence of stroke subtypes in the North East Melbourne Stroke Incidence Study (NEMESIS): differences between men and women. Neuroepidemiology.... |
| Ischemic stroke | Turin TC, Kita Y, Rumana N, Nakamura Y, Takashima N, Ichikawa M, Sugihara H, Morita Y, Hirose K, Okayama A, Miura K, Ueshima H. Ischemic Stroke Subtypes in a Japanese Population Takashima Stroke Registry, 1988-2004. Stroke. 2010; 41(9): 1871-6. |
| Ischemic stroke | Vaartjes I, Reitsma JB, de Bruin A, Berger-van Sijl M, Bos MJ, Breteler MM, Grobbee DE, Bots ML. Nationwide incidence of first stroke and TIA in the Netherlands. Eur J Neurol. 2008; 15(12): 1315-23. |
| Ischemic stroke | Zhao D, Liu J, Wang W, Zeng Z, Cheng J, Liu J, Sun J, Wu Z. Epidemiological Transition of Stroke in China Twenty-One-Year Observational Study From the Sino-MONICA-Beijing Project. Stroke. 2008; 39(6): 1668-74. |
| Ischemic stroke | Minelli C, Fu Fen L, Camara Minelli DP. Stroke Incidence, Prognosis, 30-Day, and 1-Year Case Fatality Rates in Matão, Brazil. Stroke. 2007; 38(11): 2906-11. |
| Ischemic stroke | Corso G, Bottacchi E, Giardini G, De la Pierre F, Meloni T, Pesenti Campagnoni M, Ponzetti C, Veronese Morosini M. Community-based study of stroke incidence in the Valley of Aosta, Italy. CARe-cerebrovascular Aosta Registry: years 2004-2005. Neuroepid... |
| Ischemic stroke | Kita Y, Turin TC, Ichikawa M, Sugihara H, Morita Y, Tomioka N, Rumana N, Okayama A, Nakamura Y, Abbott RD, Ueshima H. Trend of stroke incidence in a Japanese population: Takashima stroke registry, 1990-2001. Int J Stroke. 2009; 4(4): 241-9. |
| Ischemic stroke | Ashok PP, Radhakrishnan K, Sridharan R, el-Mangoush MA. Incidence and pattern of cerebrovascular diseases in Benghazi, Libya. J Neurol Neurosurg Psychiatry. 1986; 49(5): 519-23. |
| Ischemic stroke | Maheswaran R, Pearson T, Smeeton NC, Beevers SD, Campbell MJ, Wolfe CD. Outdoor air pollution and incidence of ischemic and hemorrhagic stroke: a small-area level ecological study. Stroke. 2012; 43(1): 22-7. |
| Ischemic stroke | Flynn RWV, MacDonald TM, Murray GD, Ferguson C, Shah K, Doney ASF. The Tayside Stroke Cohort: exploiting advanced regional medical informatics to create a region-wide database for studying the pharmacoepidemiology of stroke. Pharmacoepidemiol Drug Saf... |
| Ischemic stroke | Hilmarsson A, Kjartansson O, Olafsson E. Incidence of First Stroke A Population Study in Iceland. Stroke. 2013; 44(6): 1714-6. |
| Ischemic stroke | Béjot Y, Benzenine E, Lorgis L, Zeller M, Aubé H, Giroud M, Cottin Y, Quantin C. Comparative analysis of patients with acute coronary and cerebrovascular syndromes from the national French hospitalization health care system database. Neuroepidemiolo... |
| Ischemic stroke | Leyden JM, Kleinig TJ, Newbury J, Castle S, Cranefield J, Anderson CS, Crotty M, Whitford D, Jannes J, Lee A, Greenhill J. Adelaide stroke incidence study: declining stroke rates but many preventable cardioembolic strokes. Stroke. 2013; 44(5): 122... |
| Ischemic stroke | Alzamora MT, Sorribes M, Heras A, Vila N, Vicheto M, Forés R, Sánchez-Ojanguren J, Sancho A, the, Pera G. Ischemic stroke incidence in Santa Coloma de Gramenet (ISISCOG), Spain. A community-based study. BMC Neurol. 2008; 8(1): 5. |
| Ischemic stroke | Azarpazhooh MR, Etemadi MM, Donnan GA, Mokhber N, Majdi MR, Ghayour-Mobarhan M, Ghandehary K, Farzadfard MT, Kiani R, Panahandeh M, Thrift AG. Excessive Incidence of Stroke in Iran. Stroke. 2010; 41(1): e3-e10. |
| Ischemic stroke | Cabral NL, Gonçalves ARR, Longo AL, Moro CHC, Costa G, Amaral CH, Fonseca L a M, Eluf-Neto J. Incidence of stroke subtypes, prognosis and prevalence of risk factors in Joinville, Brazil: a 2 year community based study. J Neurol Neurosurg Psychiatry</... |
| Ischemic stroke | Carolei A, Marini C, Di Napoli M, Di Gianfilippo G, Santalucia P, Baldassarre M, Giorgio De Matteis M, di Orio F. High Stroke Incidence in the Prospective Community-Based L'Aquila Registry (1994-1998): First Year's Results. Stroke. 1997; 28(12): 2... |
| Ischemic stroke | Delbari A, Salman Roghani R, Tabatabaei SS, Rahgozar M, Lokk J. Stroke epidemiology and one-month fatality among an urban population in Iran. Int J Stroke. 2011; 6(3): 195-200. |
| Ischemic stroke | Di Carlo A, Inzitari D, Galati F, Baldereschi M, Giunta V, Grillo G, Furch&igrave; A, Manno V, Naso F, Vecchio A, Consoli D. A Prospective Community-Based Study of Stroke in Southern Italy: The Vibo Valentia Incidence of Stroke Study (VISS). Cerebrova... |
| Ischemic stroke | Earley CJ, Kittner SJ, Feeser BR, Gardner J, Epstein A, Wozniak MA, Wityk R, Stern BJ, Price TR, Macko RF, Johnson C, Sloan MA, Buchholz D. Stroke in children and sickle-cell disease: Baltimore-Washington Cooperative Young Stroke Study. Neurology.... |
| Ischemic stroke | Ellekjær H, Holmen J, Indredavik B, Terent A. Epidemiology of Stroke in Innherred, Norway, 1994 to 1996: Incidence and 30-Day Case-Fatality Rate. Stroke. 1997; 28(11): 2180-4. |
| Ischemic stroke | Feigin V, Carter K, Hackett M, Barber PA, McNaughton H, Dyall L, Chen M, Anderson C. Ethnic disparities in incidence of stroke subtypes: Auckland Regional Community Stroke Study, 2002-2003. Lancet Neurol. 2006; 5(2): 130-9. |
| Ischemic stroke | Ghandehari K, Moud ZI. Incidence and etiology of ischemic stroke in Persian young adults. Acta Neurol Scand. 2006; 113(2): 121-4. |
| Ischemic stroke | Giroud M, Lemesle M, Gouyon JB, Nivelon JL, Milan C, Dumas R. Cerebrovascular disease in children under 16 years of age in the city of Dijon, France: a study of incidence and clinical features from 1985 to 1993. J Clin Epidemiol. 1995; 48(11): 134... |
| Ischemic stroke | Hamad A, Hamad A, Sokrab TEO, Momeni S, Mesraoua B, Lingren A. Stroke in Qatar: A one-year, hospital-based study. J Stroke Cerebrovasc Dis. 2001; 10(5): 236-41. |
| Ischemic stroke | Kita Y, Okayama A, Ueshima H, Wada M, Nozaki A, Choudhary SR, Bonita R, Inamoto Y, Kasamatsu T. Stroke incidence and case fatality in Shiga, Japan 1989-1993. Int J Epidemiol. 1999; 28(6): 1059-65. |
| Ischemic stroke | Kolominsky-Rabas PL, Sarti C, Heuschmann PU, Graf C, Siemonsen S, Neundoerfer B, Katalinic A, Lang E, Gassmann K-G, von Stockert TR. A Prospective Community-Based Study of Stroke in Germany-The Erlangen Stroke Project (ESPro): Incidence and Case Fatality... |
| Ischemic stroke | Lauria G, Gentile M, Fassetta G, Casetta I, Agnoli F, Andreotta G, Barp C, Caneve G, Cavallaro A, Cielo R, Mongillo D, Mosca M, Olivieri P. Incidence and Prognosis of Stroke in the Belluno Province, Italy: First-Year Results of a Community-Based Study. <... |
| Ischemic stroke | Lavados PM, Sacks C, Prina L, Escobar A, Tossi C, Araya F, Feuerhake W, Galvez M, Salinas R, Alvarez G. Incidence, 30-day case-fatality rate, and prognosis of stroke in Iquique, Chile: a 2-year community-based prospective study (PISCIS project). Lance... |
| Ischemic stroke | Manobianca G, Zoccolella S, Petruzzellis A, Miccoli A, Logroscino G. The incidence of major stroke subtypes in Southern Italy: a population based study. Eur J Neurol. 2010; 17(9): 1148-55. |
| Ischemic stroke | Thrift AG, Dewey HM, Macdonell RAL, McNeil JJ, Donnan GA. Incidence of the Major Stroke Subtypes: Initial Findings From the North East Melbourne Stroke Incidence Study (NEMESIS). Stroke. 2001; 32(8): 1732-8. |
| Ischemic stroke | Tsiskaridze A, Djibuti M, van Melle G, Lomidze G, Apridonidze S, Gauarashvili I, Piechowski-Józwiak B, Shakarishvili R, Bogousslavsky J. Stroke Incidence and 30-Day Case-Fatality in a Suburb of Tbilisi: Results of the First Prospective Population-Based ... |
| Ischemic stroke | Al Rajeh S. Stroke in the Elderly Aged 75 Years and Above. Cerebrovasc Dis. 1994; 4(6): 402-6. |
| Ischemic stroke | Correia M, Silva MR, Matos I, Magalhães R, Lopes JC, Ferro JM, Silva MC. Prospective Community-Based Study of Stroke in Northern Portugal: Incidence and Case Fatality in Rural and Urban Populations. Stroke. 2004; 35(9): 2048-53. |
| Ischemic stroke | Islam MS, Anderson CS, Hankey GJ, Hardie K, Carter K, Broadhurst R, Jamrozik K. Trends in Incidence and Outcome of Stroke in Perth, Western Australia During 1989 to 2001. Stroke. 2008; 39(3): 776-82. |
| Ischemic stroke | Sridharan SE, Unnikrishnan JP, Sukumaran S, Sylaja PN, Nayak SD, Sarma PS, Radhakrishnan K. Incidence, Types, Risk Factors, and Outcome of Stroke in a Developing Country. Stroke. 2009; 40(4): 1212-8. |
| Ischemic stroke | Abdul-Ghaffar NU, el-Sonbaty MR, el-Din Abdul-Baky MS, Marafie AA, al-Said AM. Stroke in Kuwait: a three-year prospective study. Neuroepidemiology. 1997; 16(1): 40-7. |
| Ischemic stroke | al-Rajeh S, Larbi EB, Bademosi O, Awada A, Yousef A, al-Freihi H, Miniawi H. Stroke register: experience from the eastern province of Saudi Arabia. Cerebrovasc Dis. 1998; 8(2): 86-9. |
| Ischemic stroke | Anderson CS, Carter KN, Hackett ML, Feigin V, Barber PA, Broad JB, Bonita R. Trends in Stroke Incidence in Auckland, New Zealand, During 1981 to 2003. Stroke. 2005; 36(10): 2087-93. |
| Ischemic stroke | Appelros P, Nydevik I, Seiger &Aring;ke, Ter&eacute;nt A. High Incidence Rates of Stroke in Orebro, Sweden: Further Support for Regional Incidence Differences within Scandinavia. Cerebrovasc Dis. 2002; 14(3-4): 161-8. |
| Ischemic stroke | Awada A. Stroke in Saudi Arabian young adults: a study of 120 cases. Acta Neurol Scand. 1994; 89(5): 323-8. |
| Ischemic stroke | Bejot Y, Rouaud O, Durier J, Caillier M, Marie C, Freysz M, Yeguiayan J-M, Chantegret A, Osseby G, Moreau T, Giroud M. Decrease in the Stroke Case Fatality Rates in a French Population-Based Twenty-Year Study. Cerebrovasc Dis. 2007; 24(5): 439-44. |
| Ischemic stroke | Hallström B, Jönsson A-C, Nerbrand C, Norrving B, Lindgren A. Stroke Incidence and Survival in the Beginning of the 21st Century in Southern Sweden: Comparisons With the Late 20th Century and Projections Into the Future. Stroke. 2008; 39(1): 10-5. |
| Ischemic stroke | Börü UT, Oztürk E, Taşdemir M, Sur H. Living alone following first-ever stroke: a prospective study in Turkey identifying the risk factors and evaluating their effects. N Z Med J. 2007; 120(1255): U2559. |
| Ischemic stroke | Dalal PM, Malik S, Bhattacharjee M, Trivedi ND, Vairale J, Bhat P, Deshmukh S, Khandelwal K, Mathur VD. Population-based stroke survey in Mumbai, India: incidence and 28-day case fatality. Neuroepidemiology. 2008; 31(4): 254-61. |
| Ischemic stroke | Manobianca G, Zoccolella S, Petruzzellis A, Miccoli A, Logroscino G. Low Incidence of Stroke in Southern Italy: A Population-Based Study. Stroke. 2008; 39(11): 2923-8. |
| Ischemic stroke | Immonen-Räihä P, Mähönen M, Tuomilehto J, Salomaa V, Kaarsalo E, Narva EV, Salmi K, Sarti C, Sivenius J, Alhainen K, Torppa J. Trends in Case-Fatality of Stroke in Finland During 1983 to 1992. Stroke. 1997; 28(12): 2493-9. |
| Ischemic stroke | Kumral E, Ozkaya B, Sagduyu A, Sirin H, Vardarli E, Pehlivan M. The Ege Stroke Registry: A Hospital-Based Study in the Aegean Region, Izmir, Turkey. Cerebrovasc Dis. 1998; 8(5): 278-88. |
| Ischemic stroke | Vibo R, Kõrv J, Roose M. The Third Stroke Registry in Tartu, Estonia: Decline of Stroke Incidence and 28-Day Case-Fatality Rate Since 1991. Stroke. 2005; 36(12): 2544-8. |
| Ischemic stroke | Terént A. Trends in Stroke Incidence and 10-Year Survival in Söderhamn, Sweden, 1975-2001. Stroke. 2003; 34(6): 1353-8. |
| Ischemic stroke | Wolfe CDA, Giroud M, Kolominsky-Rabas P, Dundas R, Lemesle M, Heuschmann P, Rudd A. Variations in Stroke Incidence and Survival in 3 Areas of Europe. Stroke. 2000; 31(9): 2074-9. |
| Ischemic stroke | Al-Shammri S, Shahid Z, Ghali A, Mehndiratta MM, Swaminathan TR, Chadha G, Sharma PN, Akanji AO. Risk Factors, Subtypes and Outcome of Ischaemic Stroke in Kuwait - A Hospital-Based Study. Med Princ Pract. 2003; 12(4): 218-23. |
| Ischemic stroke | Talaei M, Sarrafzadegan N, Sadeghi M, Oveisgharan S, Marshall T, Thomas GN, Iranipour R. Incidence of cardiovascular diseases in an Iranian population: the Isfahan Cohort Study. Arch Iran Med. 2013; 16(3): 138-44. |
| Ischemic stroke | Walker R, Unwin N, Mugusi F, Swai M, Aris E, Jusabani A, Kabadi G, Gray W, Lewanga M, Alberti G, Whiting D. Stroke incidence in rural and urban Tanzania: a prospective, community-based study. Lancet Neurol. 2010; 9(8): 786-92. |
| Ischemic stroke | Bonzini M, Ferrario MM, Bertù L, Bono G, Vidale S, Veronesi G, Chambless L, Cesana GC. Temporal trends in ischemic and hemorrhagic strokes in Northern Italy: results from the cardiovascular monitoring unit in Northern Italy population-based register, 19... |
| Ischemic stroke | Palm F, Dos Santos M, Urbanek C, Greulich M, Zimmer K, Safer A, Grau AJ, Becher H. Stroke seasonality associations with subtype, etiology and laboratory results in the LuDisability Weightigshafen Stroke Study (LuSSt). Eur J Epidemiol. 2013; 28(5): 373-81. |
| Ischemic stroke | Pikija S, Cvetko D, Malojcic B, Trkanjec Z, Pavlicek I, Lukic A, Kopjar A, Hajduk M, Androvic A, Bilic-Genter M, Trkulja V. A population-based prospective 24-month study of stroke: incidence and 30-day case-fatality rates of first-ever strokes in Croatia... |
| Ischemic stroke | Cantu-Brito C, Majersik JJ, Sánchez BN, Ruano A, Becerra-Mendoza D, Wing JJ, Morgenstern LB. Door-to-Door Capture of Incident and Prevalent Stroke Cases in Durango, Mexico The Brain Attack Surveillance in Durango Study. Stroke. 2011; 42(3): 601-6. |
| Ischemic stroke | Damasceno A, Gomes J, Azevedo A, Carrilho C, Lobo V, Lopes H, Madede T, Pravinrai P, Silva-Matos C, Jalla S, Stewart S, Lunet N. An Epidemiological Study of Stroke Hospitalizations in Maputo, Mozambique A High Burden of Disease in a Resource-Poor Country... |
| Ischemic stroke | Carvalho JJF de, Alves MB, Viana GÁA, Machado CB, Santos BFC dos, Kanamura AH, Lottenberg CL, Neto MC, Silva GS. Stroke Epidemiology, Patterns of Management, and Outcomes in Fortaleza, Brazil A Hospital-Based Multicenter Prospective Study. Stroke... |
| Ischemic stroke | Delbari A, Salman Roghani R, Tabatabaei SS, Lökk J. A Stroke Study of an Urban Area of Iran: Risk Factors, Length of Stay, Case Fatality, and Discharge Destination. J Stroke Cerebrovasc Dis. 2010; 19(2): 104-9. |
| Ischemic stroke | Sienkiewicz-Jarosz H, Gluszkiewicz M, Pniewski J, Niewada M, Czlonkowska A, Wolfe C, Ryglewicz D. Incidence and case fatality rates of first-ever stroke - comparison of data from two prospective population-based studies conducted in Warsaw. Neurol Neu... |
| Ischemic stroke | Wawrzynczyk M, Pierzchala K, Braczkowska B, Manka-Gaca I, Kumor K, Borowski D, Grodzicka-Zawisza L, Zejda J. Estimates of stroke incidence and case fatality in Zabrze, 2005-2006. Neurol Neurochir Pol. 2011; 45(1): 3-10. |
| Ischemic stroke | Zhao Y, Yao Z, D'Souza W, Zhu C, Chun H, Zhuoga C, Zhang Q, Hu X, Zhou D. An Epidemiological Survey of Stroke in Lhasa, Tibet, China. Stroke. 2010; 41(12): 2739-43. |
| Ischemic stroke | Vemmos KN, Bots ML, Tsibouris PK, Zis VP, Grobbee DE, Stranjalis GS, Stamatelopoulos S. Stroke Incidence and Case Fatality in Southern Greece: The Arcadia Stroke Registry. Stroke. 1999; 30(2): 363-70. |
| Ischemic stroke | Bonita R, Broad JB, Beaglehole R. Changes in stroke incidence and case-fatality in Auckland, New Zealand, 1981-91. Lancet. 1993; 342(8885): 1470-3. |
| Ischemic stroke | D'Alessandro G, Bottacchi E, Di Giovanni M, Martinazzo C, Sironi L, Lia C, Carenini L, Corso G, Gerbaz V, Polillo C, Pesenti Compagnoni M. Temporal trends of stroke in Valle d'Aosta, Italy. Incidence and 30-day fatality rates. Neurol Sci. 2000; 21... |
| Ischemic stroke | Mihálka L, Smolanka V, Bulecza B, Mulesa S, Bereczki D. A Population Study of Stroke in West Ukraine: Incidence, Stroke Services, and 30-Day Case Fatality. Stroke. 2001; 32(10): 2227-31. |
| Ischemic stroke | Morikawa Y, Nakagawa H, Naruse Y, Nishijo M, Miura K, Tabata M, Hirokawa W, Kagamimori S, Honda M, Yoshita K, Hayashi K. Trends in Stroke Incidence and Acute Case Fatality in a Japanese Rural Area: The Oyabe Study. Stroke. 2000; 31(7): 1583-7. |
| Ischemic stroke | Smadja D, Cabre P, May F, Fanon J-L, René-Corail P, Riocreux C, Charpentier J-C, Fournerie P, Saint-Vil M, Ketterlé J. ERMANCIA: Epidemiology of Stroke in Martinique, French West Indies. Stroke. 2001; 32(12): 2741-7. |
| Ischemic stroke | Thrift AG, Dewey HM, Macdonell RAL, McNeil JJ, Donnan GA. Stroke Incidence on the East Coast of Australia: The North East Melbourne Stroke Incidence Study (NEMESIS). Stroke. 2000; 31(9): 2087-92. |
| Ischemic stroke | El Zunni S, Ahmed M, Prakash PS, Hassan KM. Stroke: Incidence and pattern in Benghazi, Libya. Ann Saudi Med. 1995; 15(4): 367-9. |
| Ischemic stroke | Palm F, Urbanek C, Rose S, Buggle F, Bode B, Hennerici MG, Schmieder K, Inselmann G, Reiter R, Fleischer R, Piplack K-O, Safer A, Becher H, Grau AJ. Stroke Incidence and Survival in LuDisability Weightigshafen am Rhein, Germany: the LuDisability Weightigshafen Stroke Study (LuSSt). <... |
| Ischemic stroke | Kôrv J, Roose M, Kaasik A-E. Changed Incidence and Case-Fatality Rates of First-Ever Stroke Between 1970 and 1993 in Tartu, Estonia. Stroke. 1996; 27(2): 199-203. |
| Ischemic stroke | Musolino R LSP. First-ever stroke incidence and 30-day case fatality in the Sicilian Aeolian archipelago, Italy. Stroke. 2005; 36(12): 2738-41. |
| Ischemic stroke | Das SK, Banerjee TK, Biswas A, Roy T, Raut DK, Mukherjee CS, Chaudhuri A, Hazra A, Roy J. A Prospective Community-Based Study of Stroke in Kolkata, India. Stroke. 2007; 38(3): 906-10. |
| Ischemic stroke | Matenga J. Stroke incidence rates among black residents of Harare - a prospective community-based study. S Afr Med J. 1997; 87(5): 606-8. |
| Ischemic stroke | Sweileh WM, Sawalha AF, Al-Aqad SM, Zyoud SH, Al-Jabi SW. The Epidemiology of Stroke in Northern Palestine: A 1-Year, Hospital-Based Study. J Stroke Cerebrovasc Dis. 2008; 17(6): 406-11. |
| Ischemic stroke | Zahuranec DB, Brown DL, Lisabeth LD, Morgenstern LB. Is it time for a large, collaborative study of pediatric stroke?. Stroke. 2005; 36(9): 1825-9. |
| Ischemic stroke | Wieberdink RG, Ikram MA, Hofman A, Koudstaal PJ, Breteler MMB. Trends in stroke incidence rates and stroke risk factors in Rotterdam, the Netherlands from 1990 to 2008. Eur J Epidemiol. 2012; 27(4): 287-95. |
| Ischemic stroke | Hu HH, Sheng WY, Chu FL, Lan CF, Chiang BN. Incidence of stroke in Taiwan. Stroke. 1992; 23: 1237–1241. |
| Ischemic stroke | Wu GX, Wu ZS, He BL. [Epidemiological characteristics of stroke in 16 provinces of China]. Nat Med J Chin. 1994; 74: 281–283. |
| Ischemic stroke | Feigin VL, Wiebers DO, Nikitin YP, O'Fallon WM, Whisnant JP. Stroke Epidemiology in Novosibirsk, Russia: A Population-Based Study. Mayo Clin Proc. 1995; 70(9): 847-52. |
| Ischemic stroke | Hong Y, Bots ML, Pan X, Hofman A, Grobbee DE, Chen H. Stroke Incidence and Mortality in Rural and Urban Shanghai From 1984 Through 1991: Findings From a Community-Based Registry. Stroke. 1994; 25(6): 1165-9. |
| Ischemic stroke | Jorgensen HS, Plesner AM, Hubbe P, Larsen K. Marked increase of stroke incidence in men between 1972 and 1990 in Frederiksberg, Denmark. Stroke. 1992; 23(12): 1701-4. |
| Ischemic stroke | Kulesh SD, Filina NA, Frantava NM, Zhytko NL, Kastsinevich TM, Kliatskova LA, Shumskas MS, Hilz MJ, Schwab S, Kolominsky-Rabas PL. Incidence and Case-Fatality of Stroke on the East Border of the European Union The Grodno Stroke Study. Stroke. 2010... |
| Ischemic stroke | Numminen H, Kotila M, Waltimo O, Aho K, Kaste M. Declining Incidence and Mortality Rates of Stroke in Finland From 1972 to 1991: Results of Three Population-Based Stroke Registers. Stroke. 1996; 27(9): 1487-91. |
| Ischemic stroke | World Health Organization Regional Office for Europe (WHO/Europe). European Hospital Morbidity Database. Copenhagen, Denmark: World Health Organization Regional Office for Europe (WHO/Europe). |
| Ischemic stroke | Federal Ministry of Health (Austria), Statistics Austria. Austria Hospital Inpatient Discharges 1989-1992. |
| Ischemic stroke | Federal Ministry of Health (Austria), Statistics Austria. Austria Hospital Inpatient Discharges 1993-1997. |
| Ischemic stroke | Federal Ministry of Health (Austria), Statistics Austria. Austria Hospital Inpatient Discharges 1998-2002. |
| Ischemic stroke | Federal Ministry of Health (Austria), Statistics Austria. Austria Hospital Inpatient Discharges 2003-2007. |
| Ischemic stroke | Ministry of Health (Brazil). Brazil Hospital Information System 1998-2002. |
| Ischemic stroke | Ministry of Health (Brazil). Brazil Hospital Information System 2003-2007. |
| Ischemic stroke | Ministry of Health (Brazil). Brazil Hospital Information System 2008-2012. |
| Ischemic stroke | Ministry of Health (Brazil). Brazil Hospital Information System 2013-2014. |
| Ischemic stroke | National Institute of Statistics and Censuses (Ecuador). Ecuador Hospital Inpatient Discharges 1993-1997. |
| Ischemic stroke | National Institute of Statistics and Censuses (Ecuador). Ecuador Hospital Inpatient Discharges 1998-2002. |
| Ischemic stroke | National Institute of Statistics and Censuses (Ecuador). Ecuador Hospital Inpatient Discharges 2003-2007. |
| Ischemic stroke | National Institute of Statistics and Censuses (Ecuador). Ecuador Hospital Inpatient Discharges 2008-2012. |
| Ischemic stroke | Ministry of Health (Mexico). Mexico Ministry of Health Hospital Discharges 2000-2002. |
| Ischemic stroke | Ministry of Health (Mexico). Mexico Ministry of Health Hospital Discharges 2003-2007. |
| Ischemic stroke | Ministry of Health (Mexico). Mexico Ministry of Health Hospital Discharges 2008-2012. |
| Ischemic stroke | National Center for Health Statistics (NCHS), Centers for Disease Control and Prevention (CDC). United States National Hospital Ambulatory Medical Care Survey 1993-1997. |
| Ischemic stroke | Norwegian Directorate of Health. Norway Patient Register 2008-2012. |
| Ischemic stroke | National Center for Health Statistics (NCHS), Centers for Disease Control and Prevention (CDC). United States National Hospital Ambulatory Medical Care Survey 1998-2002. |
| Ischemic stroke | Ministry of Health (New Zealand). New Zealand National Minimum Dataset 2000-2002. |
| Ischemic stroke | Ministry of Health (New Zealand). New Zealand National Minimum Dataset 2003-2007. |
| Ischemic stroke | Ministry of Health (New Zealand). New Zealand National Minimum Dataset 2008-2012. |
| Ischemic stroke | Ministry of Health (New Zealand). New Zealand National Minimum Dataset 2013-2014. |
| Ischemic stroke | National Board of Health and Welfare (Sweden). Sweden National Patient Register 1998-2002. |
| Ischemic stroke | National Center for Health Statistics (NCHS), Centers for Disease Control and Prevention (CDC). United States National Hospital Ambulatory Medical Care Survey 2003-2007. |
| Ischemic stroke | National Board of Health and Welfare (Sweden). Sweden National Patient Register 2003-2007. |
| Ischemic stroke | National Board of Health and Welfare (Sweden). Sweden National Patient Register 2008-2012. |
| Ischemic stroke | NHS England. United Kingdom - England Hospital Episode Statistics 2003-2008. |
| Ischemic stroke | National Center for Health Statistics (NCHS), Centers for Disease Control and Prevention (CDC). United States National Hospital Ambulatory Medical Care Survey 2008-2011. |
| Ischemic stroke | NHS England. United Kingdom - England Hospital Episode Statistics 2001-2003. |
| Ischemic stroke | NHS England. United Kingdom - England Hospital Episode Statistics 2008-2012. |
| Ischemic stroke | National Center for Health Statistics (NCHS), Centers for Disease Control and Prevention (CDC). United States National Hospital Discharge Survey 1988-1992. |
| Ischemic stroke | National Center for Health Statistics (NCHS), Centers for Disease Control and Prevention (CDC). United States National Hospital Discharge Survey 1993-1997. |
| Ischemic stroke | National Center for Health Statistics (NCHS), Centers for Disease Control and Prevention (CDC). United States National Hospital Discharge Survey 1998-2002. |
| Ischemic stroke | National Center for Health Statistics (NCHS), Centers for Disease Control and Prevention (CDC). United States National Hospital Discharge Survey 2003-2007. |
| Ischemic stroke | National Center for Health Statistics (NCHS), Centers for Disease Control and Prevention (CDC). United States National Hospital Discharge Survey 2008-2010. |
| Peripheral artery disease | Félix-Redondo FJ, Fernández-Bergés D, Grau M, Baena-Diez JM, Mostaza JM, Vila J. Prevalence and clinical characteristics of peripheral arterial disease in the study population Hermex. Rev Esp Cardiol (Engl Ed). 2012; 65(8): 726–33. |
| Peripheral artery disease | Alzamora MT, Forés R, Baena-Díez JM, Pera G, Toran P, Sorribes M, Vicheto M, Reina MD, Sancho A, Albaladejo C, Llussà J, PERART/ARTPER study group. The peripheral arterial disease study (PERART/ARTPER): prevalence and risk factors in the general popul... |
| Peripheral artery disease | Merino J, Planas A, Elosua R, de Moner A, Gasol A, Contreras C, Vidal-Barraquer F, Clarà A. Incidence and risk factors of peripheral arterial occlusive disease in a prospective cohort of 700 adult elderly men followed for 5 years. World J Surg. 2... |
| Peripheral artery disease | Murabito JM, Evans JC, Larson MG, Nieto K, Levy D, Wilson PWF. The Ankle-Brachial Index in the Elderly and Risk of Stroke, Coronary Disease, and Death: The Framingham Study. Arch Intern Med. 2003; 163(16): 1939-42. |
| Peripheral artery disease | Ostchega Y, Paulose-Ram R, Dillon CF, Gu Q, Hughes JP. Prevalence of peripheral arterial disease and risk factors in persons aged 60 and older: data from the National Health and Nutrition Examination Survey 1999-2004. J Am Geriatr Soc. 2007; 55(4)... |
| Peripheral artery disease | Savji N, Rockman CB, Skolnick AH, Guo Y, Adelman MA, Riles T, Berger JS. Association between advanced age and vascular disease in different arterial territories: a population database of over 3.6 million subjects. J Am Coll Cardiol. 2013; 61(16): ... |
| Peripheral artery disease | Murabito JM, Evans JC, Nieto K, Larson MG, Levy D, Wilson PWF. Prevalence and clinical correlates of peripheral arterial disease in the Framingham Offspring Study. Am Heart J. 2002; 143(6): 961-5. |
| Peripheral artery disease | He Y, Jiang Y, Wang J, Fan L, Li X, Hu FB. Prevalence of peripheral arterial disease and its association with smoking in a population-based study in Beijing, China. J Vasc Surg. 2006; 44(2): 333-8. |
| Peripheral artery disease | Sigvant B, Wiberg-Hedman K, Bergqvist D, Rolandsson O, Andersson B, Persson E, Wahlberg E. A population-based study of peripheral arterial disease prevalence with special focus on critical limb ischemia and sex differences. J Vasc Surg. 2007; 45(6... |
| Peripheral artery disease | Makdisse M, Pereira A da C, Brasil D de P, Borges JL, Machado-Coelho GLL, Krieger JE, Nascimento Neto RM, Chagas ACP, Hearts of Brazil Study and Peripheral Arterial Disease Committee of the Brazilian Society of Cardiology/Funcor. Prevalence and risk fact... |
| Peripheral artery disease | Sodhi HS, Shrestha SK, Rauniyar R, Rawat B. Prevalence of peripheral arterial disease by ankle-brachial index and its correlation with carotid intimal thickness and coronary risk factors in Nepalese population over the age of forty years. Kathmandu Un... |
| Peripheral artery disease | Guerchet M, Aboyans V, Mbelesso P, Mouanga AM, Salazar J, Bandzouzi B, Tabo A, Clément JP, Preux PM, Lacroix P. Epidemiology of Peripheral Artery Disease in Elder General Population of Two Cities of Central Africa: Bangui and Brazzaville. Eur J Vasc ... |
| Peripheral artery disease | Jones WS, Patel MR, Rockman CB, Guo Y, Adelman M, Riles T, Berger JS. Association of the ankle-brachial index with history of myocardial infarction and stroke. Am Heart J. 2014; 167(4): 499–505. |
| Peripheral artery disease | Widener JM. Peripheral arterial disease and disability from NHANES 2001-2004 data. J Vasc Nurs. 2011; 29(3): 104–12. |
| Peripheral artery disease | Joosten MM, Pai JK, Bertoia ML, Rimm EB, Spiegelman D, Mittleman MA, Mukamal KJ. Associations between conventional cardiovascular risk factors and risk of peripheral artery disease in men. JAMA. 2012; 308(16): 1660–7. |
| Peripheral artery disease | Wang Y, Xu Y, Li J, Wei Y, Zhao D, Hou L, Hasimu B, Yang J, Yuan H, Hu D. Characteristics of prevalence in peripheral arterial disease and correlative risk factors and comorbidities among female natural population in China. VASA. 2010; 39(4): 305... |
| Peripheral artery disease | Norman PE, Flicker L, Almeida OP, Hankey GJ, Hyde Z, Jamrozik K. Cohort Profile: The Health In Men Study (HIMS). Int J Epidemiol. 2009; 38(1): 48-52. |
| Peripheral artery disease | Eldrup N, Sillesen H, Prescott E, Nordestgaard BG. Ankle brachial index, C-reactive protein, and central augmentation index to identify individuals with severe atherosclerosis. Eur Heart J. 2006; 27(3): 316 -322. |
| Peripheral artery disease | Carbayo JA, Divisón JA, Escribano J, López-Abril J, López de Coca E, Artigao LM, Martínez E, Sanchis C, Massó J, Carrión L, Grupo de Enfermedades Vasculares de Albacete (GEVA). Using ankle-brachial index to detect peripheral arterial disease: preva... |
| Peripheral artery disease | Jeganathan VSE, Wong TY, Foster PJ, Crowston JG, Tay WT, Lim SC, Saw S-M, Tai ES, Aung T. Peripheral artery disease and glaucoma: the singapore malay eye study. Arch Ophthalmol. 2009; 127(7): 888-93. |
| Peripheral artery disease | Lamar Welch VL, Casper M, Greenlund K, Zheng Z-J, Giles W, Rith-Najarian S. Prevalence of lower extremity arterial disease defined by the ankle-brachial index among American Indians: the Inter-Tribal Heart Project. Ethn Dis. 2002; 12(1): S1-63-7. |
| Peripheral artery disease | Fowkes FGR, Thorogood M, Connor MD, Lewando-Hundt G, Tzoulaki I, Tollman SM. Distribution of a subclinical marker of cardiovascular risk, the ankle brachial index, in a rural African population: SASPI study. Eur J Cardiovasc Prev Rehabil. 2006; 13... |
| Peripheral artery disease | Hooi JD, Kester AD, Stoffers HE, Overdijk MM, van Ree JW, Knottnerus JA. Incidence of and risk factors for asymptomatic peripheral arterial occlusive disease: a longitudinal study. Am J Epidemiol. 2001; 153(7): 666-72. |
| Peripheral artery disease | Amudha K, Chee KH, Tan KS, Tan CT, Lang CC. Prevalence of peripheral artery disease in urban high-risk Malaysian patients. Int J Clin Pract. 2003; 57(5): 369-72. |
| Peripheral artery disease | Chuang S-Y, Chen C-H, Cheng C-M, Chou P. Combined use of brachial-ankle pulse wave velocity and ankle-brachial index for fast assessment of arteriosclerosis and atherosclerosis in a community. Int J Cardiol. 2005; 98(1): 99-105. |
| Peripheral artery disease | Kweon S-S, Shin M-H, Park K-S, Nam H-S, Jeong S-K, Ryu S-Y, Chung E-K, Choi J-S. Distribution of the ankle-brachial index and associated cardiovascular risk factors in a population of middle-aged and elderly koreans. J Korean Med Sci. 2005; 20(3):... |
| Peripheral artery disease | Kröger K, Stang A, Kondratieva J, Moebus S, Beck E, Schmermund A, Möhlenkamp S, Dragano N, Siegrist J, Jöckel K-H, Erbel R, Heinz Nixdorf Recall Study Group. Prevalence of peripheral arterial disease - results of the Heinz Nixdorf recall study. Eur... |
| Peripheral artery disease | Wong SYS, Kwok T, Woo J, Lynn H, Griffith JF, Leung J, Tang YYN, Leung PC. Bone mineral density and the risk of peripheral arterial disease in men and women: results from Mr. and Ms Os, Hong Kong. Osteoporos Int. 2005; 16(12): 1933-8. |
| Peripheral artery disease | Ramos R, Quesada M, Solanas P, Subirana I, Sala J, Vila J, Masiá R, Cerezo C, Elosua R, Grau M, Cordón F, Juvinyà D, Fitó M, Isabel Covas M, Clarà A, Angel Muñoz M, Marrugat J, REGICOR Investigators. Prevalence of symptomatic and asymptomatic perip... |
| Peripheral artery disease | Olindo S, Cabre P, Deschamps R, Chatot-Henry C, René-Corail P, Fournerie P, Saint-Vil M, May F, Smadja D. Acute stroke in the very elderly: epidemiological features, stroke subtypes, management, and outcome in Martinique, French West Indies. Stroke</... |
| Peripheral artery disease | Vigilance JE, Reid HL, Richards-George P. Peripheral occlusive arterial disease in diabetic clinic attendees. West Indian Med J. 1999; 48(3): 143-6. |
| Peripheral artery disease | Simon S, Stephenson S, Whyte K, Stubbs M, Vickers IE, Smikle MF, Gilbert DT, Barton EN. Prevalence of chronic renal failure in the diabetic population at the University Hospital of the West Indies. West Indian Med J. 2004; 53(2): 85-8. |
| Peripheral artery disease | Feringa HHH, Bax JJ, Hoeks S, van Waning VH, Elhendy A, Karagiannis S, Vidakovic R, Schouten O, Boersma E, Poldermans D. A prognostic risk index for long-term mortality in patients with peripheral arterial disease. Arch Intern Med. 2007; 167(22): ... |
| Peripheral artery disease | World Health Organization Regional Office for Europe (WHO/Europe). European Hospital Morbidity Database. Copenhagen, Denmark: World Health Organization Regional Office for Europe (WHO/Europe). |
| Peripheral artery disease | Federal Ministry of Health (Austria), Statistics Austria. Austria Hospital Inpatient Discharges 1989-1992. |
| Peripheral artery disease | Federal Ministry of Health (Austria), Statistics Austria. Austria Hospital Inpatient Discharges 1993-1997. |
| Peripheral artery disease | Federal Ministry of Health (Austria), Statistics Austria. Austria Hospital Inpatient Discharges 1998-2002. |
| Peripheral artery disease | Federal Ministry of Health (Austria), Statistics Austria. Austria Hospital Inpatient Discharges 2003-2007. |
| Peripheral artery disease | Ministry of Health (Brazil). Brazil Hospital Information System 1998-2002. |
| Peripheral artery disease | Ministry of Health (Brazil). Brazil Hospital Information System 2003-2007. |
| Peripheral artery disease | Ministry of Health (Brazil). Brazil Hospital Information System 2008-2012. |
| Peripheral artery disease | Ministry of Health (Brazil). Brazil Hospital Information System 2013-2014. |
| Peripheral artery disease | National Institute of Statistics and Censuses (Ecuador). Ecuador Hospital Inpatient Discharges 1993-1997. |
| Peripheral artery disease | National Institute of Statistics and Censuses (Ecuador). Ecuador Hospital Inpatient Discharges 1998-2002. |
| Peripheral artery disease | National Institute of Statistics and Censuses (Ecuador). Ecuador Hospital Inpatient Discharges 2003-2007. |
| Peripheral artery disease | National Institute of Statistics and Censuses (Ecuador). Ecuador Hospital Inpatient Discharges 2008-2012. |
| Peripheral artery disease | Ministry of Health (Mexico). Mexico Ministry of Health Hospital Discharges 2000-2002. |
| Peripheral artery disease | Ministry of Health (Mexico). Mexico Ministry of Health Hospital Discharges 2003-2007. |
| Peripheral artery disease | Ministry of Health (Mexico). Mexico Ministry of Health Hospital Discharges 2008-2012. |
| Peripheral artery disease | National Center for Health Statistics (NCHS), Centers for Disease Control and Prevention (CDC). United States National Hospital Ambulatory Medical Care Survey 1993-1997. |
| Peripheral artery disease | Norwegian Directorate of Health. Norway Patient Register 2008-2012. |
| Peripheral artery disease | National Center for Health Statistics (NCHS), Centers for Disease Control and Prevention (CDC). United States National Hospital Ambulatory Medical Care Survey 1998-2002. |
| Peripheral artery disease | Ministry of Health (New Zealand). New Zealand National Minimum Dataset 2000-2002. |
| Peripheral artery disease | Ministry of Health (New Zealand). New Zealand National Minimum Dataset 2003-2007. |
| Peripheral artery disease | Ministry of Health (New Zealand). New Zealand National Minimum Dataset 2008-2012. |
| Peripheral artery disease | Ministry of Health (New Zealand). New Zealand National Minimum Dataset 2013-2014. |
| Peripheral artery disease | National Board of Health and Welfare (Sweden). Sweden National Patient Register 1998-2002. |
| Peripheral artery disease | National Center for Health Statistics (NCHS), Centers for Disease Control and Prevention (CDC). United States National Hospital Ambulatory Medical Care Survey 2003-2007. |
| Peripheral artery disease | National Board of Health and Welfare (Sweden). Sweden National Patient Register 2003-2007. |
| Peripheral artery disease | National Board of Health and Welfare (Sweden). Sweden National Patient Register 2008-2012. |
| Peripheral artery disease | NHS England. United Kingdom - England Hospital Episode Statistics 2003-2008. |
| Peripheral artery disease | National Center for Health Statistics (NCHS), Centers for Disease Control and Prevention (CDC). United States National Hospital Ambulatory Medical Care Survey 2008-2011. |
| Peripheral artery disease | NHS England. United Kingdom - England Hospital Episode Statistics 2001-2003. |
| Peripheral artery disease | NHS England. United Kingdom - England Hospital Episode Statistics 2008-2012. |
| Peripheral artery disease | National Center for Health Statistics (NCHS), Centers for Disease Control and Prevention (CDC). United States National Hospital Discharge Survey 1988-1992. |
| Peripheral artery disease | National Center for Health Statistics (NCHS), Centers for Disease Control and Prevention (CDC). United States National Hospital Discharge Survey 1993-1997. |
| Peripheral artery disease | National Center for Health Statistics (NCHS), Centers for Disease Control and Prevention (CDC). United States National Hospital Discharge Survey 1998-2002. |
| Peripheral artery disease | National Center for Health Statistics (NCHS), Centers for Disease Control and Prevention (CDC). United States National Hospital Discharge Survey 2003-2007. |
| Peripheral artery disease | National Center for Health Statistics (NCHS), Centers for Disease Control and Prevention (CDC). United States National Hospital Discharge Survey 2008-2010. |
| Peripheral artery disease | Wattanakit K, Williams JE, Schreiner PJ, Hirsch AT, Folsom AR. Association of anger proneness, depression and low social support with peripheral arterial disease: the Atherosclerosis Risk in Communities Study. Vasc Med. 2005; 10(3): 199-206. |
| Rheumatic heart disease | Agarwal AK, Yunus M, Ahmad J, Khan A. Rheumatic heart disease in India. J R Soc Promot Health. 1995; 115(5): 303-9. |
| Rheumatic heart disease | Boedhi-Darmojo R. The pattern of cardiovascular disease in Indonesia. World Health Stat Q. 1993; 46(2): 119-24. |
| Rheumatic heart disease | Folomeeva OM, Benevolenskaia LI. Rheumatism in the Russian Federation: statistic and reality. Vestn Ross Akad Med Nauk. 1996; 11: 21-4. |
| Rheumatic heart disease | Alves Meira ZM, de Castilho SR, Lins Barros MV, Maria Vitarelli A, Diniz Capanema F, Moreira NS, Moreira Camargos PA, Coelho Mota CC. Prevalence of rheumatic fever in children from a public high school in Belo Horizonte. Arq Bras Cardiol. 1995; 65... |
| Rheumatic heart disease | Carapetis JR, Currie BJ. Clinical epidemiology of rheumatic fever and rheumatic heart disease in tropical Australia. Adv Exp Med Biol. 1997; 418: 233-6. |
| Rheumatic heart disease | Carapetis JR, Wolff DR, Currie BJ. Acute rheumatic fever and rheumatic heart disease in the top end of Australia's Northern Territory. Med J Aust. 1996; 164(3): 146-9. |
| Rheumatic heart disease | Carapetis JR, Currie BJ, Mathews JD. Cumulative incidence of rheumatic fever in an endemic region: a guide to the susceptibility of the population?. Epidemiol Infect. 2000; 124(2): 239-44. |
| Rheumatic heart disease | Krylov MYu, Srámek J, Annenkova IP, Alekseeva LI, Myakotkin VA, Shokh BP, Eshchina AS. Rheumatic fever and streptococcal infections in an isolated population group in eastern Siberia. J Hyg Epidemiol Microbiol Immunol. 1990; 34(1): 45-51. |
| Rheumatic heart disease | Marijon E, Ou P, Celermajer DS, Ferreira B, Mocumbi AO, Jani D, Paquet C, Jacob S, Sidi D, Jouven X. Prevalence of rheumatic heart disease detected by echocardiographic screening. N Engl J Med. 2007; 357(5): 470-6. |
| Rheumatic heart disease | Nordet P, Lopez R, Dueñas A, Sarmiento L. Prevention and control of rheumatic fever and rheumatic heart disease: the Cuban experience (1986-1996-2002). Cardiovasc J Afr. 2008; 19(3): 135-40. |
| Rheumatic heart disease | Vashistha VM, Kalra A, Kalra K, Jain VK. Prevalence of rheumatic heart disease in school children. Indian Pediatr. 1993; 30(1): 53-6. |
| Rheumatic heart disease | Viali S. Rheumatic fever and rheumatic heart disease in Samoa. Pac Health Dialog. 2006; 13(2): 31-8. |
| Rheumatic heart disease | Reeves BM, Kado J, Brook M. High prevalence of rheumatic heart disease in Fiji detected by echocardiography screening. J Paediatr Child Health. 2011; 47(7): 473-8. |
| Rheumatic heart disease | Webb RH, Wilson NJ, Lennon DR, Wilson EM, Nicholson RW, Gentles TL, O'Donnell CP, Stirling JW, Zeng I, Trenholme AA. Optimising echocardiographic screening for rheumatic heart disease in New Zealand: not all valve disease is rheumatic. Cardiol Young</... |
| Rheumatic heart disease | Tapia PJ, Suarez EI, Lanza VHZ, Balderrama MH. Contribution to the Epidemiological Study of Rheumatic Fever / Rheumatic Heart Disease (RF/RHD). Review of School District V. Villa Fatima, La Paz, Bolivia. La Paz, Bolivia: Ministry of Health (Bolivia), ... |
| Rheumatic heart disease | Rossi E, Felici AR, Banteyrga L. Subclinical rheumatic heart disease in an Eritrean high-school population, detected by echocardiography. J Heart Valve Dis. 2014; 23(2.0): 235-9. |
| Rheumatic heart disease | Ba-Saddik IA, Munibari AA, Al-Naqeeb MS, Parry CM, Hart CA, Cuevas LE, Coulter JB. Prevalence of rheumatic heart disease among school-children in Aden, Yemen. Ann Trop Paediatr. 2011; 31(1.0): 37-46. |
| Rheumatic heart disease | Beaton A, Okello E, Lwabi P, Mondo C, McCarter R, Sable C. Echocardiography screening for rheumatic heart disease in Ugandan schoolchildren. Circulation. 2012; 125(25): 3127-32. |
| Rheumatic heart disease | Beaton A, Lu JC, Aliku T, Dean P, Gaur L, Weinberg J, Godown J, Lwabi P, Mirembe G, Okello E, Reese A, Shrestha-Astudillo A, Bradley-Hewitt T, Scheel J, Webb C, McCarter R, Ensing G, Sable C. The utility of handheld echocardiography for early rheumatic h... |
| Rheumatic heart disease | BharDisability Weightaj R, Kandoria A, Marwah R, Vaidya P, Singh B, Dhiman P, Sharma A. Prevalence of rheumatic fever and rheumatic heart disease in rural population of Himachal--a population based study. J Assoc Physicians India. 2012; 13-4. |
| Rheumatic heart disease | Kane A, Mirabel M, Tour‚ K, P‚rier MC, Fazaa S, Tafflet M, Karam N, Zourak I, Diagne D, Mbaye A, Kane M, Diack B, Jouven X, Marijon E. Echocardiographic screening for rheumatic heart disease: age matters. Int J Cardiol. 2013; 168(2.0): 888-91. |
| Rheumatic heart disease | Rama Kumari N, Bhaskara Raju I, Patnaik AN, Barik R, Singh A, Pushpanjali A, Laxmi V, Satya Ramakrishna L. Prevalence of rheumatic and congenital heart disease in school children of Andhra Pradesh, South India. J Cardiovasc Dis Res. 2013; 4(1.0): ... |
| Rheumatic heart disease | Mirabel M, Celermajer DS, Ferreira B, Tafflet M, Perier MC, Karam N, Mocumbi AO, Jani DN, Sidi D, Jouven X, Marijon E. Screening for rheumatic heart disease: evaluation of a simplified echocardiography-based approach. Eur Heart J Cardiovasc Imaging</i... |
| Rheumatic heart disease | Lu JC, Sable C, Ensing GJ, Webb C, Scheel J, Aliku T, Lwabi P, Godown J, Beaton A. Simplified Rheumatic Heart Disease Screening Criteria for Handheld Echocardiography. J Am Soc Echocardiogr. 2015; nan. |
| Rheumatic heart disease | Negi PC, Kanwar A, Chauhan R, Asotra S, Thakur JS, BharDisability Weightaj AK. Epidemiological trends of RF/RHD in school children of Shimla in north India. Indian J Med Res. 2013; 137(6.0): 1121-7. |
| Rheumatic heart disease | Saxena A, Ramakrishnan S, Roy A, Seth S, Krishnan A, Misra P, Kalaivani M, Bhargava B, Flather MD, Poole-Wilson PPA. Prevalence and outcome of subclinical rheumatic heart disease in India: the RHEUMATIC (Rheumatic Heart Echo Utilisation and Monitoring Ac... |
| Rheumatic heart disease | Sriharibabu M, Himabindu Y, Kabir Z. Rheumatic heart disease in rural south India: A clinico-observational study. J Cardiovasc Dis Res. 2013; 4(1.0): 25-9. |
| Rheumatic heart disease | Htoon MT, Ngwe T, Tun N, Kyaw MM. Prevalence of Cardiovascular Diseases in Rural Area of Hmawbi and Urban Yangon City. Asia Pac J Public Health. 1992; 6(4): 188-94. |
| Rheumatic heart disease | Kermani S, Berah H. La situation epidemiologique du RAA en Algerie depuis 1990 [Algeria Epidemiological Situation on Rheumatic Fever Since 1990]. Algiers, Algeria: Ministry of Health, Population and Hospital Reform (Algeria), 2001. |
| Rheumatic heart disease | Ibrahim-Khalil S, Elhag M, Ali E, Mahgoub F, Hakiem S, Omer N, Shafie S, Mahgoub E. An epidemiological survey of rheumatic fever and rheumatic heart disease in Sahafa Town, Sudan. J Epidemiol Community Health. 1992; 46(5): 477-9. |
| Rheumatic heart disease | Abdel-Moula AM, Sherif AA, Sallam SA, Mandil AM, Kassem AS, Zaher SR. Prevalence of rheumatic heart disease among school children in Alexandria, Egypt: a prospective epidemiological study. J Egypt Public Health Assoc. 1998; 73(3-4): 233-54. |
| Rheumatic heart disease | Ahmed J, Mostafa Zaman M, Monzur Hassan MM. Prevalence of rheumatic fever and rheumatic heart disease in rural Bangladesh. Trop Doct. 2005; 35(3): 160-1. |
| Rheumatic heart disease | Anabwani GM, Bonhoeffer P. Prevalence of heart disease in school children in rural Kenya using colour-flow echocardiography. East Afr Med J. 1996; 73(4): 215-7. |
| Rheumatic heart disease | Bahadur KCM, Sharma D, Shrestha MP, Gurung S, Rajbhandari S, Malla R, Rajbhandari R, Limbu YR, Regmi SR, Koirala B. Prevalence of rheumatic and congenital heart disease in schoolchildren of Kathmandu valley in Nepal. Indian Heart J. 2003; 55(6): 6... |
| Rheumatic heart disease | Brennan RE, Patel MS. Acute rheumatic fever and rheumatic heart disease in a rural central Australian aboriginal community. Med J Aust. 1990; 153(6): 335-9. |
| Rheumatic heart disease | Carapetis JR, Hardy M, Fakakovikaetau T, Taib R, Wilkinson L, Penny DJ, Steer AC. Evaluation of a screening protocol using auscultation and portable echocardiography to detect asymptomatic rheumatic heart disease in Tongan schoolchildren. Nat Clin Pra... |
| Rheumatic heart disease | Chen X, Zhang M, Huang D, Huang M, Xiong Y, Xie M, Shou Y, Li M, Wu C, Zeng H, Li X, Zheng M. An epidemiologic investigation of acute rheumatic fever and rheumatic heart disease among students aged 5-18 in west area of Sichuan Province. J Sichuan Univ... |
| Rheumatic heart disease | Cuboni HD, Finau SA, Cuboni G. Rheumatic fever and rheumatic heart diseases in Fiji: a review from the surveillance system (1996 -2000). Pac Health Dialog. 2006; 13(2): 39-47. |
| Rheumatic heart disease | Gupta I, Gupta ML, Parihar A, Gupta CD. Epidemiology of rheumatic and congenital heart diseases in school children. J Indian Med Assoc. 1992; 90(3): 57-9. |
| Rheumatic heart disease | Hasab AA, Jaffer A, Riyami AM. Rheumatic heart disease among Omani schoolchildren. East Mediterr Health J. 1997; 3(1): 17-23. |
| Rheumatic heart disease | Jose VJ, Gomathi M. Declining prevalence of rheumatic heart disease in rural schoolchildren in India: 2001-2002. Indian Heart J. 2003; 55(2): 158-60. |
| Rheumatic heart disease | Kimbally-Kaky G, Gombet T, Voumbo Y, Ikama-Méo S, Elenga-Mbola B, Mbika-Cardorelle A, Dilou L, Ekoba J, Nkoua JL, Moyen G, Bouramoué C. Rheumatic heart disease in schoolchildren in Brazzaville. Med Trop (Mars). 2008; 68(6): 603-5. |
| Rheumatic heart disease | Longo-Mbenza B, Bayekula M, Ngiyulu R, Kintoki VE, Bikangi NF, Seghers KV, Lukoki LE, Mandundu MF, Manzanza M, Nlandu Y. Survey of rheumatic heart disease in school children of Kinshasa town. Int J Cardiol. 1998; 63(3): 287-94. |
| Rheumatic heart disease | Mukelabai K, Pobee JOM, Shilalukey-Ngoma M, Malek ANA, Pankajam MI, Mupela M. Rheumatic heart disease in a sub-saharan african city: epidemiology, prophylaxis and health education. Cardiol Trop. 2000; 26(102): 25-8. |
| Rheumatic heart disease | Neilson G, Streatfield RW, West M, Johnson S, Glavin W, Baird S. Rheumatic fever and chronic rheumatic heart disease in Yarrabah aboriginal community, north Queensland. Establishment of a prophylactic program. Med J Aust. 1993; 158(5): 316-8. |
| Rheumatic heart disease | Olguntürk R, Aydin GB, Tunaoğlu FS, Akalin N. Rheumatic heart disease prevalence among schoolchildren in Ankara, Turkey. Turk J Pediatr. 1999; 41(2): 201-6. |
| Rheumatic heart disease | Oli K, Porteous J. Prevalence of rheumatic heart disease among school children in Addis Ababa. East Afr Med J. 1999; 76(11): 601-5. |
| Rheumatic heart disease | Oli K, Porteous J. Rheumatic heart disease among school children in Addis Ababa City: awareness and adequacy of its prophylaxis. Ethiop Med J. 1999; 37(3): 155-61. |
| Rheumatic heart disease | Regmi PR, Pandey MR. Prevalence of rheumatic fever and rheumatic heart disease in school children of Kathmandu city. Indian Heart J. 1997; 49(5): 518-20. |
| Rheumatic heart disease | Rizvi SF, Khan MA, Kundi A, Marsh DR, Samad A, Pasha O. Status of rheumatic heart disease in rural Pakistan. Heart. 2004; 90(4): 394-9. |
| Rheumatic heart disease | Sadiq M, Islam K, Abid R, Latif F, Rehman AU, Waheed A, Azhar M, Khan JS. Prevalence of rheumatic heart disease in school children of urban Lahore. Heart. 2009; 95(5): 353-7. |
| Rheumatic heart disease | Shrestha UK, Bhattarai TN, Pandey MR. Prevalence of rheumatic fever and rheumatic heart disease in school children in a rural community of the hill region of Nepal. Indian Heart J. 1991; 43(1): 39-41. |
| Rheumatic heart disease | Toure S, Balde MD, Balde OD, Sow T, Toure A, Conde A, Diallo MC, Traore O, Fofana M. Enquête sur les cardiopathies en milieu scolaire et universitaire à Conakry (R. Guinée). Cardiol Trop. 1992; 18(72): 205-10. |
| Rheumatic heart disease | World Health Organization (WHO). WHO Global Programme for the Prevention of Rheumatic Fever and Rheumatic Heart Disease: Report of a Consultation to Review Progress and Develop Future Activities. Geneva, Switzerland: World Health Organization (WHO), 2000. |
| Rheumatic heart disease | Munibari AA. A Study on Rheumatic Fever and Rheumatic Heart Disease in Yemen [dissertation]. Khartoum, Sudan: University of Khartoum, 1997. |
| Rheumatic heart disease | Shoheib S, Madkour S and El Din RS Younis. Egypt Evaluation of RF/RHD Control Program Impact among Egyptian Schooldchildren 1998.&nbsp; |
| Rheumatic heart disease | Tapia MD, Sow SO, Sanogo K, Keita MM, Mason R, Rubin F, Dale JB, Kotloff KL. The prevalence of rheumatic heart disease among children in Bamako, Mali: preliminary results. Presented at: XVII Lancefield International Symposium on Streptococci and Strep... |
| Rheumatic heart disease | Steer AC, Kado J, Wilson N, Tuiketei T, Batzloff M, Waqatakirewa L, Mulholland EK, Carapetis JR. High prevalence of rheumatic heart disease by clinical and echocardiographic screening among children in Fiji. J Heart Valve Dis. 2009; 18(3): 327-36. |
| Rheumatic heart disease | Colquhoun SM, Kado JH, Remenyi B, Wilson NJ, Carapetis JR, Steer AC. Echocardiographic screening in a resource poor setting: borderline rheumatic heart disease could be a normal variant. Int J Cardiol. 2014; 173(2.0): 284-9. |
| Rheumatic heart disease | Bhaya M, Panwar S, Beniwal R, Panwar RB. High prevalence of rheumatic heart disease detected by echocardiography in school children. Echocardiography. 2010; 27(4): 448-53. |
| Rheumatic heart disease | Thakur JS, Negi PC, Ahluwalia SK, Vaidya NK. Epidemiological survey of rheumatic heart disease among school children in the Shimla Hills of northern India: prevalence and risk factors. J Epidemiol Community Health. 1996; 50(1): 62-7. |
| Rheumatic heart disease | Zhimin W, Yubao Z, Lei S, Xianliang Z, Wei Z, Li S, Hao W, Jianjun L, Detrano R, Rutai H. Prevalence of chronic rheumatic heart disease in Chinese adults. Int J Cardiol. 2006; 107(3): 356-9. |
| Rheumatic heart disease | Periwal KL, Gupta BK, Panwar RB, Khatri PC, Raja S, Gupta R. Prevalence of rheumatic heart disease in school children in Bikaner: an echocardiographic study. J Assoc Physicians India. 2006; 54: 279-82. |
| Rheumatic heart disease | Kumar P, Garhwal S, Chaudhary V. Rheumatic heart disease: a school survey in a rural area of Rajasthan. Indian Heart J. 1992; 44(4): 245-6. |
| Rheumatic heart disease | al-Sekait MA, al-Sweliem AA, Tahir M. Rheumatic heart disease in schoolchildren in western district, Saudi Arabia. J R Soc Health. 1990; 110(1): 15-6, 19. |
| Rheumatic heart disease | Paar JA, Berrios NM, Rose JD, Cáceres M, Peña R, Pérez W, Chen-Mok M, Jolles E, Dale JB. Prevalence of rheumatic heart disease in children and young adults in Nicaragua. Am J Cardiol. 2010; 105(12): 1809-14. |
| Rheumatic heart disease | Misra M, Mittal M, Singh R, Verma A, Rai R, Chandra G, Singh D, Chauhan R, Chowdhary V, Singh R, Mall A, Khan MJ, Khare S, Yadav K. Prevalence of rheumatic heart disease in school-going children of Eastern Uttar Pradesh. Indian Heart J. 2007; 59(1... |
| Rheumatic heart disease | Sliwa K, Carrington M, Mayosi BM, Zigiriadis E, Mvungi R, Stewart S. Incidence and characteristics of newly diagnosed rheumatic heart disease in Urban African adults: insights from the Heart of Soweto Study. Eur Heart J. 2010; 31(6): 719-27. |
| Rheumatic heart disease | Baroux N, Rouchon B, Huon B, Germain A, Meunier JM, D'Ortenzio E. High prevalence of rheumatic heart disease in schoolchildren detected by echocardiography screening in New Caledonia. J Paediatr Child Health. 2013; 49(2.0): 109-14. |
| Rheumatic heart disease | Mirabel M, Bacquelin R, Tafflet M, Robillard C, Huon B, Corsenac P, de Fr‚micourt I, Narayanan K, Meunier JM, No‰l B, HagŠge AA, Rouchon B, Jouven X, Marijon E. Screening for rheumatic heart disease: evaluation of a focused cardiac ultrasound approa... |
| Rheumatic heart disease | Miranda LP, Camargos PA, Torres RM, Meira ZM. Prevalence of rheumatic heart disease in a public school of Belo Horizonte. Arq Bras Cardiol. 2014; 103(2.0): 89-97. |
| Rheumatic heart disease | Breda L, Marzetti V, Gaspari S, Del Torto M, Chiarelli F, Altobelli E. Population-based study of incidence and clinical characteristics of rheumatic fever in Abruzzo, central Italy, 2000-2009. J Pediatr. 2012; 160(5.0): 832-6e1. |
| Rheumatic heart disease | Federal Ministry of Health (Austria), Statistics Austria. Austria Hospital Inpatient Discharges 1989-1992. |
| Rheumatic heart disease | Federal Ministry of Health (Austria), Statistics Austria. Austria Hospital Inpatient Discharges 1993-1997. |
| Rheumatic heart disease | Federal Ministry of Health (Austria), Statistics Austria. Austria Hospital Inpatient Discharges 1998-2002. |
| Rheumatic heart disease | World Health Organization Regional Office for Europe (WHO/Europe). European Hospital Morbidity Database. Copenhagen, Denmark: World Health Organization Regional Office for Europe (WHO/Europe). |
| Rheumatic heart disease | National Center for Health Statistics (NCHS), Centers for Disease Control and Prevention (CDC). United States National Hospital Discharge Survey 1988-1992. |
| Rheumatic heart disease | National Center for Health Statistics (NCHS), Centers for Disease Control and Prevention (CDC). United States National Hospital Discharge Survey 1993-1997. |
| Rheumatic heart disease | National Center for Health Statistics (NCHS), Centers for Disease Control and Prevention (CDC). United States National Hospital Discharge Survey 1998-2002. |
| Rheumatic heart disease | National Center for Health Statistics (NCHS), Centers for Disease Control and Prevention (CDC). United States National Hospital Discharge Survey 2003-2007. |
| Rheumatic heart disease | National Center for Health Statistics (NCHS), Centers for Disease Control and Prevention (CDC). United States National Hospital Discharge Survey 2008-2010. |
| Rheumatic heart disease | National Institute of Statistics and Censuses (Ecuador). Ecuador Hospital Inpatient Discharges 1993-1997. |
| Rheumatic heart disease | National Institute of Statistics and Censuses (Ecuador). Ecuador Hospital Inpatient Discharges 1998-2002. |
| Rheumatic heart disease | Federal Ministry of Health (Austria), Statistics Austria. Austria Hospital Inpatient Discharges 2003-2007. |
| Rheumatic heart disease | Ministry of Health (Brazil). Brazil Hospital Information System 1998-2002. |
| Rheumatic heart disease | Ministry of Health (Brazil). Brazil Hospital Information System 2003-2007. |
| Rheumatic heart disease | Ministry of Health (Brazil). Brazil Hospital Information System 2008-2012. |
| Rheumatic heart disease | Ministry of Health (Brazil). Brazil Hospital Information System 2013-2014. |
| Rheumatic heart disease | National Institute of Statistics and Censuses (Ecuador). Ecuador Hospital Inpatient Discharges 2003-2007. |
| Rheumatic heart disease | National Institute of Statistics and Censuses (Ecuador). Ecuador Hospital Inpatient Discharges 2008-2012. |
| Rheumatic heart disease | Ministry of Health (Mexico). Mexico Ministry of Health Hospital Discharges 2000-2002. |
| Rheumatic heart disease | Ministry of Health (Mexico). Mexico Ministry of Health Hospital Discharges 2003-2007. |
| Rheumatic heart disease | Ministry of Health (Mexico). Mexico Ministry of Health Hospital Discharges 2008-2012. |
| Rheumatic heart disease | National Center for Health Statistics (NCHS), Centers for Disease Control and Prevention (CDC). United States National Hospital Ambulatory Medical Care Survey 1993-1997. |
| Rheumatic heart disease | Norwegian Directorate of Health. Norway Patient Register 2008-2012. |
| Rheumatic heart disease | National Center for Health Statistics (NCHS), Centers for Disease Control and Prevention (CDC). United States National Hospital Ambulatory Medical Care Survey 1998-2002. |
| Rheumatic heart disease | Ministry of Health (New Zealand). New Zealand National Minimum Dataset 2000-2002. |
| Rheumatic heart disease | Ministry of Health (New Zealand). New Zealand National Minimum Dataset 2003-2007. |
| Rheumatic heart disease | Ministry of Health (New Zealand). New Zealand National Minimum Dataset 2008-2012. |
| Rheumatic heart disease | Ministry of Health (New Zealand). New Zealand National Minimum Dataset 2013-2014. |
| Rheumatic heart disease | National Board of Health and Welfare (Sweden). Sweden National Patient Register 1998-2002. |
| Rheumatic heart disease | National Center for Health Statistics (NCHS), Centers for Disease Control and Prevention (CDC). United States National Hospital Ambulatory Medical Care Survey 2003-2007. |
| Rheumatic heart disease | National Board of Health and Welfare (Sweden). Sweden National Patient Register 2003-2007. |
| Rheumatic heart disease | National Board of Health and Welfare (Sweden). Sweden National Patient Register 2008-2012. |
| Rheumatic heart disease | NHS England. United Kingdom - England Hospital Episode Statistics 2003-2008. |
| Rheumatic heart disease | National Center for Health Statistics (NCHS), Centers for Disease Control and Prevention (CDC). United States National Hospital Ambulatory Medical Care Survey 2008-2011. |
| Rheumatic heart disease | NHS England. United Kingdom - England Hospital Episode Statistics 2001-2003. |
| Rheumatic heart disease | NHS England. United Kingdom - England Hospital Episode Statistics 2008-2012. |

| **Appendix Table 4. GBD 2015 geography hierarchy with levels** | |
| --- | --- |
| **Location** | **Level** |
| **Global** | 0 |
| **High SDI** | 1 |
| **High-middle SDI** | 1 |
| **Middle SDI** | 1 |
| **Low-middle SDI** | 1 |
| **Low SDI** | 1 |
| **Southeast Asia, East Asia, and Oceania** | 1 |
| East Asia | 2 |
| China | 3 |
| North Korea | 3 |
| Taiwan | 3 |
| Southeast Asia | 2 |
| Cambodia | 3 |
| Indonesia | 3 |
| Laos | 3 |
| Malaysia | 3 |
| Maldives | 3 |
| Mauritius | 3 |
| Myanmar | 3 |
| Philippines | 3 |
| Sri Lanka | 3 |
| Seychelles | 3 |
| Thailand | 3 |
| Timor-Leste | 3 |
| Vietnam | 3 |
| Oceania | 2 |
| American Samoa | 3 |
| Federated States of Micronesia | 3 |
| Fiji | 3 |
| Guam | 3 |
| Kiribati | 3 |
| Marshall Islands | 3 |
| Northern Mariana Islands | 3 |
| Papua New Guinea | 3 |
| Samoa | 3 |
| Solomon Islands | 3 |
| Tonga | 3 |
| Vanuatu | 3 |
| **Central Europe, Eastern Europe, and Central Asia** | 1 |
| Central Asia | 2 |
| Armenia | 3 |
| Azerbaijan | 3 |
| Georgia | 3 |
| Kazakhstan | 3 |
| Kyrgyzstan | 3 |
| Mongolia | 3 |
| Tajikistan | 3 |
| Turkmenistan | 3 |
| Uzbekistan | 3 |
| Central Europe | 2 |
| Albania | 3 |
| Bosnia and Herzegovina | 3 |
| Bulgaria | 3 |
| Croatia | 3 |
| Czech Republic | 3 |
| Hungary | 3 |
| Macedonia | 3 |
| Montenegro | 3 |
| Poland | 3 |
| Romania | 3 |
| Serbia | 3 |
| Slovakia | 3 |
| Slovenia | 3 |
| Eastern Europe | 2 |
| Belarus | 3 |
| Estonia | 3 |
| Latvia | 3 |
| Lithuania | 3 |
| Moldova | 3 |
| Russia | 3 |
| Ukraine | 3 |
| **High-income** | 1 |
| High-income Asia Pacific | 2 |
| Brunei | 3 |
| Japan | 3 |
| South Korea | 3 |
| Singapore | 3 |
| Australasia | 2 |
| Australia | 3 |
| New Zealand | 3 |
| Western Europe | 2 |
| Andorra | 3 |
| Austria | 3 |
| Belgium | 3 |
| Cyprus | 3 |
| Denmark | 3 |
| Finland | 3 |
| France | 3 |
| Germany | 3 |
| Greece | 3 |
| Iceland | 3 |
| Ireland | 3 |
| Israel | 3 |
| Italy | 3 |
| Luxembourg | 3 |
| Malta | 3 |
| Netherlands | 3 |
| Norway | 3 |
| Portugal | 3 |
| Spain | 3 |
| Sweden | 3 |
| Switzerland | 3 |
| United Kingdom | 3 |
| England | 4 |
| Northern Ireland | 4 |
| Scotland | 4 |
| Wales | 4 |
| Southern Latin America | 2 |
| Argentina | 3 |
| Chile | 3 |
| Uruguay | 3 |
| High-income North America | 2 |
| Canada | 3 |
| Greenland | 3 |
| United States | 3 |
| **Latin America and Caribbean** | 1 |
| Caribbean | 2 |
| Antigua and Barbuda | 3 |
| The Bahamas | 3 |
| Barbados | 3 |
| Belize | 3 |
| Bermuda | 3 |
| Cuba | 3 |
| Dominica | 3 |
| Dominican Republic | 3 |
| Grenada | 3 |
| Guyana | 3 |
| Haiti | 3 |
| Jamaica | 3 |
| Puerto Rico | 3 |
| Saint Lucia | 3 |
| Saint Vincent and the Grenadines | 3 |
| Suriname | 3 |
| Trinidad and Tobago | 3 |
| Virgin Islands, U.S. | 3 |
| Andean Latin America | 2 |
| Bolivia | 3 |
| Ecuador | 3 |
| Peru | 3 |
| Central Latin America | 2 |
| Colombia | 3 |
| Costa Rica | 3 |
| El Salvador | 3 |
| Guatemala | 3 |
| Honduras | 3 |
| Mexico | 3 |
| Nicaragua | 3 |
| Panama | 3 |
| Venezuela | 3 |
| Tropical Latin America | 2 |
| Brazil | 3 |
| Paraguay | 3 |
| **North Africa and Middle East** | 1 |
| North Africa and Middle East | 2 |
| Afghanistan | 3 |
| Algeria | 3 |
| Bahrain | 3 |
| Egypt | 3 |
| Iran | 3 |
| Iraq | 3 |
| Jordan | 3 |
| Kuwait | 3 |
| Lebanon | 3 |
| Libya | 3 |
| Morocco | 3 |
| Palestine | 3 |
| Oman | 3 |
| Qatar | 3 |
| Saudi Arabia | 3 |
| Sudan | 3 |
| Syria | 3 |
| Tunisia | 3 |
| Turkey | 3 |
| United Arab Emirates | 3 |
| Yemen | 3 |
| **South Asia** | 1 |
| South Asia | 2 |
| Bangladesh | 3 |
| Bhutan | 3 |
| India | 3 |
| Nepal | 3 |
| Pakistan | 3 |
| **Sub-Saharan Africa** | 1 |
| Central Sub-Saharan Africa | 2 |
| Angola | 3 |
| Central African Republic | 3 |
| Congo | 3 |
| Democratic Republic of the Congo | 3 |
| Equatorial Guinea | 3 |
| Gabon | 3 |
| Eastern Sub-Saharan Africa | 2 |
| Burundi | 3 |
| Comoros | 3 |
| Djibouti | 3 |
| Eritrea | 3 |
| Ethiopia | 3 |
| Kenya | 3 |
| Madagascar | 3 |
| Malawi | 3 |
| Mozambique | 3 |
| Rwanda | 3 |
| Somalia | 3 |
| South Sudan | 3 |
| Tanzania | 3 |
| Uganda | 3 |
| Zambia | 3 |
| Southern Sub-Saharan Africa | 2 |
| Botswana | 3 |
| Lesotho | 3 |
| Namibia | 3 |
| South Africa | 3 |
| Swaziland | 3 |
| Zimbabwe | 3 |
| Western Sub-Saharan Africa | 2 |
| Benin | 3 |
| Burkina Faso | 3 |
| Cameroon | 3 |
| Cape Verde | 3 |
| Chad | 3 |
| Cote d'Ivoire | 3 |
| The Gambia | 3 |
| Ghana | 3 |
| Guinea | 3 |
| Guinea-Bissau | 3 |
| Liberia | 3 |
| Mali | 3 |
| Mauritania | 3 |
| Niger | 3 |
| Nigeria | 3 |
| Sao Tome and Principe | 3 |
| Senegal | 3 |
| Sierra Leone | 3 |
| Togo | 3 |

| **Appendix Table 5. Socio-demographic Index (SDI) groupings by geography, based on 2015 values** | |
| --- | --- |
| **Location** | **SDI level** |
| Andorra | High SDI |
| Antigua and Barbuda | High SDI |
| Australia | High SDI |
| Austria | High SDI |
| Belarus | High SDI |
| Belgium | High SDI |
| Bermuda | High SDI |
| Brunei | High SDI |
| Canada | High SDI |
| Cyprus | High SDI |
| Czech Republic | High SDI |
| Denmark | High SDI |
| Estonia | High SDI |
| Finland | High SDI |
| France | High SDI |
| Germany | High SDI |
| Guam | High SDI |
| Hungary | High SDI |
| Iceland | High SDI |
| Ireland | High SDI |
| Israel | High SDI |
| Italy | High SDI |
| Kuwait | High SDI |
| Latvia | High SDI |
| Lithuania | High SDI |
| Luxembourg | High SDI |
| Netherlands | High SDI |
| New Zealand | High SDI |
| Northern Mariana Islands | High SDI |
| Norway | High SDI |
| Poland | High SDI |
| Puerto Rico | High SDI |
| Russia | High SDI |
| Scotland | High SDI |
| Singapore | High SDI |
| Slovakia | High SDI |
| Slovenia | High SDI |
| South Korea | High SDI |
| Switzerland | High SDI |
| Taiwan | High SDI |
| The Bahamas | High SDI |
| Trinidad and Tobago | High SDI |
| United Arab Emirates | High SDI |
| Virgin Islands, U.S. | High SDI |
| Wales | High SDI |
| Albania | High-middle SDI |
| American Samoa | High-middle SDI |
| Argentina | High-middle SDI |
| Armenia | High-middle SDI |
| Azerbaijan | High-middle SDI |
| Bahrain | High-middle SDI |
| Barbados | High-middle SDI |
| Bosnia and Herzegovina | High-middle SDI |
| Bulgaria | High-middle SDI |
| Chile | High-middle SDI |
| Colombia | High-middle SDI |
| Costa Rica | High-middle SDI |
| Croatia | High-middle SDI |
| Cuba | High-middle SDI |
| Dominica | High-middle SDI |
| Dominican Republic | High-middle SDI |
| Ecuador | High-middle SDI |
| Fiji | High-middle SDI |
| Georgia | High-middle SDI |
| Greece | High-middle SDI |
| Greenland | High-middle SDI |
| Grenada | High-middle SDI |
| Iran | High-middle SDI |
| Jamaica | High-middle SDI |
| Jordan | High-middle SDI |
| Kazakhstan | High-middle SDI |
| Lebanon | High-middle SDI |
| Macedonia | High-middle SDI |
| Malaysia | High-middle SDI |
| Malta | High-middle SDI |
| Mauritius | High-middle SDI |
| México | High-middle SDI |
| Moldova | High-middle SDI |
| Mongolia | High-middle SDI |
| Montenegro | High-middle SDI |
| Northern Ireland | High-middle SDI |
| Oman | High-middle SDI |
| Panama | High-middle SDI |
| Peru | High-middle SDI |
| Portugal | High-middle SDI |
| Qatar | High-middle SDI |
| Romania | High-middle SDI |
| Saint Lucia | High-middle SDI |
| Saint Vincent and the Grenadines | High-middle SDI |
| Serbia | High-middle SDI |
| Seychelles | High-middle SDI |
| Spain | High-middle SDI |
| Sri Lanka | High-middle SDI |
| Suriname | High-middle SDI |
| Thailand | High-middle SDI |
| Turkey | High-middle SDI |
| Turkmenistan | High-middle SDI |
| Ukraine | High-middle SDI |
| Uruguay | High-middle SDI |
| Uzbekistan | High-middle SDI |
| Venezuela | High-middle SDI |
| Algeria | Middle SDI |
| Belize | Middle SDI |
| Bolivia | Middle SDI |
| Botswana | Middle SDI |
| Egypt | Middle SDI |
| El Salvador | Middle SDI |
| Equatorial Guinea | Middle SDI |
| Federated States of Micronesia | Middle SDI |
| Gabon | Middle SDI |
| Guyana | Middle SDI |
| Honduras | Middle SDI |
| Indonesia | Middle SDI |
| Iraq | Middle SDI |
| Kyrgyzstan | Middle SDI |
| Libya | Middle SDI |
| Maldives | Middle SDI |
| Marshall Islands | Middle SDI |
| Namibia | Middle SDI |
| Nicaragua | Middle SDI |
| North Korea | Middle SDI |
| Palestine | Middle SDI |
| Paraguay | Middle SDI |
| Philippines | Middle SDI |
| Samoa | Middle SDI |
| Swaziland | Middle SDI |
| Syria | Middle SDI |
| Tajikistan | Middle SDI |
| Tonga | Middle SDI |
| Tunisia | Middle SDI |
| Vietnam | Middle SDI |
| Angola | Low-middle SDI |
| Bangladesh | Low-middle SDI |
| Bhutan | Low-middle SDI |
| Cambodia | Low-middle SDI |
| Cameroon | Low-middle SDI |
| Cape Verde | Low-middle SDI |
| Congo | Low-middle SDI |
| Djibouti | Low-middle SDI |
| Ghana | Low-middle SDI |
| Guatemala | Low-middle SDI |
| Haiti | Low-middle SDI |
| Kiribati | Low-middle SDI |
| Laos | Low-middle SDI |
| Lesotho | Low-middle SDI |
| Morocco | Low-middle SDI |
| Myanmar | Low-middle SDI |
| Nepal | Low-middle SDI |
| Nigeria | Low-middle SDI |
| Pakistan | Low-middle SDI |
| Papua New Guinea | Low-middle SDI |
| Sao Tome and Principe | Low-middle SDI |
| Solomon Islands | Low-middle SDI |
| Sudan | Low-middle SDI |
| Tanzania | Low-middle SDI |
| Timor-Leste | Low-middle SDI |
| Vanuatu | Low-middle SDI |
| Yemen | Low-middle SDI |
| Zambia | Low-middle SDI |
| Zimbabwe | Low-middle SDI |
| Afghanistan | Low SDI |
| Benin | Low SDI |
| Burkina Faso | Low SDI |
| Burundi | Low SDI |
| Central African Republic | Low SDI |
| Chad | Low SDI |
| Comoros | Low SDI |
| Cote d'Ivoire | Low SDI |
| Democratic Republic of the Congo | Low SDI |
| Eritrea | Low SDI |
| Ethiopia | Low SDI |
| Guinea | Low SDI |
| Guinea-Bissau | Low SDI |
| Liberia | Low SDI |
| Madagascar | Low SDI |
| Malawi | Low SDI |
| Mali | Low SDI |
| Mauritania | Low SDI |
| Mozambique | Low SDI |
| Niger | Low SDI |
| Rwanda | Low SDI |
| Senegal | Low SDI |
| Sierra Leone | Low SDI |
| Somalia | Low SDI |
| South Sudan | Low SDI |
| The Gambia | Low SDI |
| Togo | Low SDI |
| Uganda | Low SDI |

| **Appendix Table 6. GBD 2015 CODem model covariates by CVD Cause** | | | |
| --- | --- | --- | --- |
| **Cause Name** | **Level** | **Covariate Name** | **Direction** |
| Cardiovascular diseases | 2 | Alcohol (liters per capita) | 0 |
| Cardiovascular diseases | 2 | Animal Fats (kcal per capita) | 1 |
| Cardiovascular diseases | 1 | Cholesterol (total, mean per capita) | 1 |
| Cardiovascular diseases | 1 | Elevation Under 100m (proportion) | 1 |
| Cardiovascular diseases | 3 | LDI (I$ per capita) | -1 |
| Cardiovascular diseases | 2 | Omega 3 adjusted(g) | -1 |
| Cardiovascular diseases | 2 | Red Meats Adjusted(g) | 1 |
| Cardiovascular diseases | 1 | Smoking Prevalence | 1 |
| Cardiovascular diseases | 3 | Sociodemographic Status | 0 |
| Cardiovascular diseases | 2 | Vegetables Adjusted(g) | -1 |
| Rheumatic heart disease | 3 | Education (years per capita) | -1 |
| Rheumatic heart disease | 3 | Health System Access 2 (unitless) | -1 |
| Rheumatic heart disease | 1 | Improved Water Source (proportion with access) | -1 |
| Rheumatic heart disease | 3 | LDI (I$ per capita) | -1 |
| Rheumatic heart disease | 1 | Log-transformed SEV scalar: RHD | 1 |
| Rheumatic heart disease | 1 | Malnutrition (proportion <2SD weight for age) | 1 |
| Rheumatic heart disease | 1 | Population Under 30 (proportion) | 1 |
| Rheumatic heart disease | 1 | Sanitation (proportion with access) | -1 |
| Rheumatic heart disease | 3 | Sociodemographic Status | -1 |
| Ischemic heart disease | 2 | Alcohol (liters per capita) | 0 |
| Ischemic heart disease | 2 | Animal Fats (kcal per capita) | 1 |
| Ischemic heart disease | 1 | Cholesterol (total, mean per capita) | 1 |
| Ischemic heart disease | 1 | Elevation Under 100m (proportion) | 1 |
| Ischemic heart disease | 3 | LDI (I$ per capita) | -1 |
| Ischemic heart disease | 1 | Log-transformed SEV scalar: IHD | 1 |
| Ischemic heart disease | 2 | Omega 3 Adjusted(g) | -1 |
| Ischemic heart disease | 3 | Pulses Legumes Adjusted(g) | -1 |
| Ischemic heart disease | 3 | Red Meats Adjusted(g) | 1 |
| Ischemic heart disease | 1 | Smoking Prevalence | 1 |
| Ischemic heart disease | 3 | Sociodemographic Status | 0 |
| Ischemic heart disease | 2 | Vegetables Adjusted(g) | -1 |
| Cerebrovascular disease | 2 | Alcohol (liters per capita) | 0 |
| Cerebrovascular disease | 1 | Cholesterol (total, mean per capita) | 1 |
| Cerebrovascular disease | 1 | Cumulative Cigarettes (5 Years) | 1 |
| Cerebrovascular disease | 3 | Diabetes Age-Standardized Prevalence (proportion) | 1 |
| Cerebrovascular disease | 3 | Education (years per capita) | -1 |
| Cerebrovascular disease | 2 | Fruits Adjusted(g) | -1 |
| Cerebrovascular disease | 3 | LDI (I$ per capita) | -1 |
| Cerebrovascular disease | 1 | Log-transformed SEV scalar: Stroke | 1 |
| Cerebrovascular disease | 1 | Mean BMI | 1 |
| Cerebrovascular disease | 3 | Red Meats Adjusted(g) | 1 |
| Cerebrovascular disease | 3 | Sociodemographic Status | 0 |
| Cerebrovascular disease | 1 | Systolic Blood Pressure (mmHg) | 1 |
| Cerebrovascular disease | 2 | Vegetables Adjusted(g) | -1 |
| Cerebrovascular disease | 3 | Whole Grains Adjusted(g) | -1 |
| Ischemic stroke | 2 | Alcohol (liters per capita) | 0 |
| Ischemic stroke | 3 | Animal Fats (kcal per capita) | 1 |
| Ischemic stroke | 1 | Cholesterol (total, mean per capita) | 1 |
| Ischemic stroke | 3 | Education (years per capita) | -1 |
| Ischemic stroke | 2 | Fruits Adjusted(g) | -1 |
| Ischemic stroke | 2 | Health System Access 2 (unitless) | -1 |
| Ischemic stroke | 3 | LDI (I$ per capita) | -1 |
| Ischemic stroke | 1 | Log-transformed SEV scalar: Isch Stroke | 1 |
| Ischemic stroke | 2 | Milk Adjusted(g) | -1 |
| Ischemic stroke | 2 | Nuts & Seeds (kcal per capita) | -1 |
| Ischemic stroke | 2 | Nuts Seeds Adjusted(g) | -1 |
| Ischemic stroke | 3 | Omega 3 Adjusted(g) | -1 |
| Ischemic stroke | 3 | PUFA Omega 3 - Seafood (kcal per capita) | -1 |
| Ischemic stroke | 2 | Pulses Legumes Adjusted(g) | -1 |
| Ischemic stroke | 1 | Smoking Prevalence | 1 |
| Ischemic stroke | 3 | Sociodemographic Status | 0 |
| Ischemic stroke | 2 | Vegetables Adjusted(g) | -1 |
| Hemorrhagic stroke | 2 | Alcohol (liters per capita) | 0 |
| Hemorrhagic stroke | 3 | Education (years per capita) | -1 |
| Hemorrhagic stroke | 3 | LDI (I$ per capita) | -1 |
| Hemorrhagic stroke | 1 | Log-transformed SEV scalar: Hem Stroke | 1 |
| Hemorrhagic stroke | 2 | Milk Adjusted(g) | -1 |
| Hemorrhagic stroke | 2 | Nuts Seeds Adjusted(g) | -1 |
| Hemorrhagic stroke | 2 | Omega 3 Adjusted(g) | -1 |
| Hemorrhagic stroke | 2 | Pufa Adjusted(percent) | -1 |
| Hemorrhagic stroke | 2 | Pulses Legumes Adjusted(g) | -1 |
| Hemorrhagic stroke | 1 | Smoking Prevalence | 1 |
| Hemorrhagic stroke | 3 | Sociodemographic Status | 0 |
| Hemorrhagic stroke | 2 | Vegetables Adjusted(g) | -1 |
| Hypertensive heart disease | 2 | Alcohol (liters per capita) | 0 |
| Hypertensive heart disease | 2 | Cumulative Cigarettes (10 Years) | 1 |
| Hypertensive heart disease | 3 | Education (years per capita) | -1 |
| Hypertensive heart disease | 3 | Elevation Under 100m (proportion) | -1 |
| Hypertensive heart disease | 2 | Energy Unadjusted(kcal) | 1 |
| Hypertensive heart disease | 2 | Indoor Air Pollution (All Cooking Fuels) | 1 |
| Hypertensive heart disease | 3 | LDI (I$ per capita) | -1 |
| Hypertensive heart disease | 2 | Milk Adjusted(g) | -1 |
| Hypertensive heart disease | 2 | Nuts Seeds Adjusted(g) | -1 |
| Hypertensive heart disease | 2 | Omega 3 Adjusted(g) | -1 |
| Hypertensive heart disease | 2 | Outdoor Air Pollution (PM2.5) | 1 |
| Hypertensive heart disease | 2 | Pulses Legumes Adjusted(g) | -1 |
| Hypertensive heart disease | 3 | Sociodemographic Status | -1 |
| Hypertensive heart disease | 1 | Systolic Blood Pressure (mmHg) | 1 |
| Hypertensive heart disease | 2 | Vegetables Adjusted(g) | -1 |
| Cardiomyopathy and myocarditis | 2 | Alcohol (liters per capita) | 0 |
| Cardiomyopathy and myocarditis | 1 | Diabetes Age-Standardized Prevalence (proportion) | 1 |
| Cardiomyopathy and myocarditis | 3 | Education (years per capita) | -1 |
| Cardiomyopathy and myocarditis | 3 | Health System Access 2 (unitless) | -1 |
| Cardiomyopathy and myocarditis | 3 | LDI (I$ per capita) | -1 |
| Cardiomyopathy and myocarditis | 1 | Log-transformed SEV scalar: CMP | 1 |
| Cardiomyopathy and myocarditis | 3 | Sociodemographic Status | 0 |
| Cardiomyopathy and myocarditis | 1 | Systolic Blood Pressure (mmHg) | 1 |
| Atrial fibrillation and flutter | 2 | Alcohol (liters per capita) | 0 |
| Atrial fibrillation and flutter | 2 | Animal Fats (kcal per capita) | 1 |
| Atrial fibrillation and flutter | 1 | Cholesterol (total, mean per capita) | 1 |
| Atrial fibrillation and flutter | 2 | Cumulative Cigarettes (10 Years) | 1 |
| Atrial fibrillation and flutter | 1 | Diabetes Age-Standardized Prevalence (proportion) | 1 |
| Atrial fibrillation and flutter | 3 | Education (years per capita) | -1 |
| Atrial fibrillation and flutter | 3 | Elevation 100 to 500m (proportion) | -1 |
| Atrial fibrillation and flutter | 3 | Elevation 500 to 1500m (proportion) | -1 |
| Atrial fibrillation and flutter | 3 | Elevation Over 1500m (proportion) | -1 |
| Atrial fibrillation and flutter | 3 | Elevation Under 100m (proportion) | -1 |
| Atrial fibrillation and flutter | 2 | Energy Unadjusted(kcal) | 1 |
| Atrial fibrillation and flutter | 2 | Fruits Adjusted(g) | -1 |
| Atrial fibrillation and flutter | 1 | Health System Access 2 (unitless) | -1 |
| Atrial fibrillation and flutter | 2 | Indoor Air Pollution (All Cooking Fuels) | 1 |
| Atrial fibrillation and flutter | 3 | LDI (I$ per capita) | -1 |
| Atrial fibrillation and flutter | 1 | Log-transformed SEV scalar: A Fib | 1 |
| Atrial fibrillation and flutter | 1 | Mean BMI | 1 |
| Atrial fibrillation and flutter | 2 | Milk Adjusted(g) | -1 |
| Atrial fibrillation and flutter | 2 | Nuts Seeds Adjusted(g) | -1 |
| Atrial fibrillation and flutter | 2 | Omega 3 Adjusted(g) | -1 |
| Atrial fibrillation and flutter | 2 | Outdoor Air Pollution (PM2.5) | 1 |
| Atrial fibrillation and flutter | 2 | Pufa Adjusted(percent) | -1 |
| Atrial fibrillation and flutter | 2 | Pulses Legumes Adjusted(g) | -1 |
| Atrial fibrillation and flutter | 2 | Red Meats Adjusted(g) | 1 |
| Atrial fibrillation and flutter | 2 | Smoking Prevalence | 1 |
| Atrial fibrillation and flutter | 3 | Sociodemographic Status | 0 |
| Atrial fibrillation and flutter | 1 | Systolic Blood Pressure (mmHg) | 1 |
| Atrial fibrillation and flutter | 1 | Tobacco (cigarettes per capita) | 1 |
| Atrial fibrillation and flutter | 2 | Vegetables Adjusted(g) | -1 |
| Atrial fibrillation and flutter | 2 | Whole Grains (kcal per capita) | -1 |
| Atrial fibrillation and flutter | 2 | Whole Grains Adjusted(g) | -1 |
| Aortic aneurysm | 2 | Alcohol (liters per capita) | 0 |
| Aortic aneurysm | 1 | Cholesterol (total, mean per capita) | 1 |
| Aortic aneurysm | 1 | Cumulative Cigarettes (5 Years) | 1 |
| Aortic aneurysm | 1 | Diabetes Age-Standardized Prevalence (proportion) | 1 |
| Aortic aneurysm | 3 | Education (years per capita) | -1 |
| Aortic aneurysm | 2 | Fruits Adjusted(g) | -1 |
| Aortic aneurysm | 1 | Health System Access 2 (unitless) | -1 |
| Aortic aneurysm | 2 | Indoor Air Pollution (All Cooking Fuels) | 1 |
| Aortic aneurysm | 3 | LDI (I$ per capita) | -1 |
| Aortic aneurysm | 1 | Log-transformed SEV scalar: Aort An | 1 |
| Aortic aneurysm | 1 | Mean BMI | 1 |
| Aortic aneurysm | 2 | Outdoor Air Pollution (PM2.5) | 1 |
| Aortic aneurysm | 2 | Red Meats Adjusted(g) | 1 |
| Aortic aneurysm | 3 | Sociodemographic Status | 0 |
| Aortic aneurysm | 1 | Systolic Blood Pressure (mmHg) | 1 |
| Aortic aneurysm | 2 | Vegetables Adjusted(g) | -1 |
| Aortic aneurysm | 2 | Whole Grains Adjusted(g) | -1 |
| Peripheral artery disease | 2 | Alcohol (liters per capita) | 0 |
| Peripheral artery disease | 1 | Cholesterol (total, mean per capita) | 1 |
| Peripheral artery disease | 2 | Cumulative Cigarettes (10 Years) | 1 |
| Peripheral artery disease | 1 | Diabetes Age-Standardized Prevalence (proportion) | 1 |
| Peripheral artery disease | 3 | Education (years per capita) | -1 |
| Peripheral artery disease | 2 | Energy Unadjusted(kcal) | 1 |
| Peripheral artery disease | 2 | Fruits Adjusted(g) | -1 |
| Peripheral artery disease | 3 | Health System Access 2 (unitless) | -1 |
| Peripheral artery disease | 2 | Indoor Air Pollution (All Cooking Fuels) | 1 |
| Peripheral artery disease | 3 | LDI (I$ per capita) | -1 |
| Peripheral artery disease | 1 | Log-transformed SEV scalar: PVD | 1 |
| Peripheral artery disease | 1 | Mean BMI | 1 |
| Peripheral artery disease | 2 | Nuts Seeds Adjusted(g) | -1 |
| Peripheral artery disease | 2 | Omega 3 Adjusted(g) | -1 |
| Peripheral artery disease | 2 | Outdoor Air Pollution (PM2.5) | 1 |
| Peripheral artery disease | 2 | Pufa Adjusted(percent) | -1 |
| Peripheral artery disease | 2 | Pulses Legumes Adjusted(g) | -1 |
| Peripheral artery disease | 2 | Red Meats Adjusted(g) | 1 |
| Peripheral artery disease | 2 | Smoking Prevalence | 1 |
| Peripheral artery disease | 3 | Sociodemographic Status | 0 |
| Peripheral artery disease | 1 | Systolic Blood Pressure (mmHg) | 1 |
| Peripheral artery disease | 1 | Tobacco (cigarettes per capita) | 1 |
| Peripheral artery disease | 2 | Vegetables Adjusted(g) | -1 |
| Peripheral artery disease | 2 | Whole Grains Adjusted(g) | -1 |
| Endocarditis | 3 | Education (years per capita) | -1 |
| Endocarditis | 3 | Health System Access 2 (unitless) | -1 |
| Endocarditis | 1 | Improved Water Source (proportion with access) | -1 |
| Endocarditis | 3 | LDI (I$ per capita) | -1 |
| Endocarditis | 1 | Log-transformed SEV scalar: Endocar | 1 |
| Endocarditis | 1 | Sanitation (proportion with access) | -1 |
| Endocarditis | 3 | Sociodemographic Status | 0 |
| Other cardiovascular and circulatory diseases | 2 | Alcohol (liters per capita) | 0 |
| Other cardiovascular and circulatory diseases | 2 | Animal Fats (kcal per capita) | 1 |
| Other cardiovascular and circulatory diseases | 1 | Cholesterol (total, mean per capita) | 1 |
| Other cardiovascular and circulatory diseases | 2 | Cumulative Cigarettes (10 Years) | 1 |
| Other cardiovascular and circulatory diseases | 1 | Diabetes Age-Standardized Prevalence (proportion) | 1 |
| Other cardiovascular and circulatory diseases | 3 | Education (years per capita) | -1 |
| Other cardiovascular and circulatory diseases | 3 | Elevation 100 to 500m (proportion) | -1 |
| Other cardiovascular and circulatory diseases | 2 | Energy Unadjusted(kcal) | -1 |
| Other cardiovascular and circulatory diseases | 2 | Fruits Adjusted(g) | -1 |
| Other cardiovascular and circulatory diseases | 3 | Health System Access 2 (unitless) | -1 |
| Other cardiovascular and circulatory diseases | 2 | Indoor Air Pollution (All Cooking Fuels) | 1 |
| Other cardiovascular and circulatory diseases | 3 | LDI (I$ per capita) | -1 |
| Other cardiovascular and circulatory diseases | 1 | Log-transformed SEV scalar: Oth Cardio | 1 |
| Other cardiovascular and circulatory diseases | 1 | Mean BMI | 1 |
| Other cardiovascular and circulatory diseases | 2 | Milk Adjusted(g) | -1 |
| Other cardiovascular and circulatory diseases | 2 | Nuts Seeds Adjusted(g) | -1 |
| Other cardiovascular and circulatory diseases | 2 | Omega 3 Adjusted(g) | -1 |
| Other cardiovascular and circulatory diseases | 2 | Outdoor Air Pollution (PM2.5) | 1 |
| Other cardiovascular and circulatory diseases | 2 | Pufa Adjusted(percent) | -1 |
| Other cardiovascular and circulatory diseases | 2 | Pulses Legumes Adjusted(g) | -1 |
| Other cardiovascular and circulatory diseases | 2 | Red Meats Adjusted(g) | 1 |
| Other cardiovascular and circulatory diseases | 2 | Smoking Prevalence | 1 |
| Other cardiovascular and circulatory diseases | 3 | Sociodemographic Status | -1 |
| Other cardiovascular and circulatory diseases | 1 | Systolic Blood Pressure (mmHg) | 1 |
| Other cardiovascular and circulatory diseases | 1 | Tobacco (cigarettes per capita) | 1 |
| Other cardiovascular and circulatory diseases | 2 | Vegetables (kcal per capita) | -1 |
| Other cardiovascular and circulatory diseases | 2 | Vegetables Adjusted(g) | -1 |
| Other cardiovascular and circulatory diseases | 2 | Whole Grains Adjusted(g) | -1 |

| **Appendix Table 7: CODEm predictive validity results by CVD cause, sex, age, and location** | | | | | | | | | |
| --- | --- | --- | --- | --- | --- | --- | --- | --- | --- |
| Predictive validity | | | | | | | | | |
| Cause | Sex | Age start | Age end | RMSE in | RMSE out | Trend in | Trend out | Coverage in | Coverage out |
| Cardiomyopathy and myocarditis [Global] | Male | 0-6 days | 80+ years | 0·250984 0·460313 0·157951 0·165789 0·999158 0·976814 | | | | | |
| Cardiomyopathy and myocarditis [Global] | Female | 0-6 days | 80+ years | 0·23834 0·505763 0·160754 0·167175 0·999059 0·979949 | | | | | |
| Aortic aneurysm [Global] | Male | 15-19 years | 80+ years | 0·182199 0·363829 0·130304 0·135468 0·999783 0·97444 | | | | | |
| Aortic aneurysm [Global] | Female | 15-19 years | 80+ years | 0·203481 0·393351 0·144398 0·145741 0·99965 0·987712 | | | | | |
| Ischemic stroke [Global] | Male | 28-364 days | 80+ years | 0·197159 0·377334 0·135414 0·141827 0·999437 0·983448 | | | | | |
| Ischemic stroke [Global] | Female | 28-364 days | 80+ years | 0·212798 0·381924 0·145229 0·149829 0·999326 0·987279 | | | | | |
| Peripheral vascular disease [Global] | Male | 40-44 years | 80+ years | 0·319626 0·665863 0·221345 0·22302 0·996146 0·962651 | | | | | |
| Peripheral vascular disease [Global] | Female | 40-44 years | 80+ years | 0·346023 0·785803 0·23743 0·244099 0·992567 0·963198 | | | | | |
| Rheumatic heart disease [Global] | Male | 1-4 years | 80+ years | 0·20831 0·449328 0·138955 0·145013 0·999067 0·982077 | | | | | |
| Rheumatic heart disease [Global] | Female | 1-4 years | 80+ years | 0·225209 0·478289 0·153975 0·155001 0·999099 0·979783 | | | | | |
| Cardiovascular diseases [Global] | Male | 0-6 days | 80+ years | 0·169307 0·322962 0·118066 0·125409 0·998178 0·961493 | | | | | |
| Cardiovascular diseases [Global] | Female | 0-6 days | 80+ years | 0·171362 0·313795 0·120172 0·122979 0·998588 0·975545 | | | | | |
| Cerebrovascular disease [Global] | Male | 0-6 days | 80+ years | 0·198954 0·378368 0·135884 0·138689 0·998958 0·973586 | | | | | |
| Cerebrovascular disease [Global] | Female | 0-6 days | 80+ years | 0·187137 0·382782 0·130865 0·134796 0·999261 0·979278 | | | | | |
| Hemorrhagic stroke [Global] | Male | 0-6 days | 80+ years | 0·207991 0·375961 0·145933 0·152469 0·999247 0·984863 | | | | | |
| Hemorrhagic stroke [Global] | Female | 0-6 days | 80+ years | 0·204726 0·372164 0·14328 0·146654 0·999392 0·988202 | | | | | |
| Hypertensive heart disease [Global] | Male | 28-364 days | 80+ years | 0·261659 0·652723 0·155297 0·159703 0·958196 0·928237 | | | | | |
| Hypertensive heart disease [Global] | Female | 28-364 days | 80+ years | 0·276519 0·618354 0·143769 0·145815 0·963531 0·943911 | | | | | |
| Ischemic heart disease [Global] | Male | 28-364 days | 80+ years | 0·212464 0·411426 0·136687 0·143346 0·998111 0·948388 | | | | | |
| Ischemic heart disease [Global] | Female | 28-364 days | 80+ years | 0·208296 0·440276 0·133356 0·139166 0·997648 0·967468 | | | | | |
| Other cardiovascular and circulatory diseases [Global] | Male | 0-6 days | 80+ years | 0·240368 0·484629 0·153867 0·157924 0·997411 0·985108 | | | | | |
| Other cardiovascular and circulatory diseases [Global] | Female | 0-6 days | 80+ years | 0·25037 0·487232 0·159746 0·165778 0·997575 0·990026 | | | | | |
| Cardiovascular diseases [Data Rich] | Male | 0-6 days | 80+ years | 0·128789 0·157006 0·0977253 0·111164 0·999528 0·998792 | | | | | |
| Cardiovascular diseases [Data Rich] | Female | 0-6 days | 80+ years | 0·13191 0·159791 0·100678 0·115697 0·999587 0·999022 | | | | | |
| Rheumatic heart disease [Data Rich] | Male | 1-4 years | 80+ years | 0·154642 0·191258 0·116127 0·131276 0·999744 0·999166 | | | | | |
| Rheumatic heart disease [Data Rich] | Female | 1-4 years | 80+ years | 0·167888 0·210923 0·127274 0·145119 0·999717 0·999285 | | | | | |
| Ischemic heart disease [Data Rich] | Male | 28-364 days | 80+ years | 0·148811 0·185673 0·106221 0·123874 0·999568 0·999002 | | | | | |
| Ischemic heart disease [Data Rich] | Female | 28-364 days | 80+ years | 0·156253 0·194679 0·112207 0·127082 0·998142 0·997551 | | | | | |
| Cerebrovascular disease [Data Rich] | Male | 0-6 days | 80+ years | 0·148468 0·180354 0·111289 0·127271 0·999843 0·999328 | | | | | |
| Cerebrovascular disease [Data Rich] | Female | 0-6 days | 80+ years | 0·14839 0·179681 0·111404 0·124484 0·999812 0·999444 | | | | | |
| Ischemic stroke [Data Rich] | Male | 28-364 days | 80+ years | 0·162974 0·208628 0·122859 0·140799 0·999829 0·999542 | | | | | |
| Ischemic stroke [Data Rich] | Female | 28-364 days | 80+ years | 0·176751 0·226582 0·138814 0·156014 0·999427 0·999145 | | | | | |
| Hemorrhagic stroke [Data Rich] | Male | 0-6 days | 80+ years | 0·159354 0·206351 0·120562 0·138704 0·999531 0·998722 | | | | | |
| Hemorrhagic stroke [Data Rich] | Female | 0-6 days | 80+ years | 0·159895 0·210713 0·121499 0·136864 0·999653 0·999104 | | | | | |
| Hypertensive heart disease [Data Rich] | Male | 28-364 days | 80+ years | 0·20642 0·305453 0·122387 0·146038 0·940738 0·94004 | | | | | |
| Hypertensive heart disease [Data Rich] | Female | 28-364 days | 80+ years | 0·18966 0·280052 0·112534 0·131474 0·947252 0·946682 | | | | | |
| Cardiomyopathy and myocarditis [Data Rich] | Male | 0-6 days | 80+ years | 0·172748 0·251127 0·12851 0·151246 0·999407 0·998112 | | | | | |
| Cardiomyopathy and myocarditis [Data Rich] | Female | 0-6 days | 80+ years | 0·168162 0·241965 0·124297 0·143526 0·99929 0·997977 | | | | | |
| Aortic aneurysm [Data Rich] | Male | 15-19 years | 80+ years | 0·154626 0·241811 0·119887 0·133369 0·999707 0·999376 | | | | | |
| Aortic aneurysm [Data Rich] | Female | 15-19 years | 80+ years | 0·165558 0·242339 0·12879 0·139556 0·999684 0·999434 | | | | | |
| Peripheral vascular disease [Data Rich] | Male | 40-44 years | 80+ years | 0·274108 0·570584 0·199772 0·206832 0·996571 0·993439 | | | | | |
| Peripheral vascular disease [Data Rich] | Female | 40-44 years | 80+ years | 0·283709 0·624997 0·20301 0·233713 0·994326 0·99139 | | | | | |
| Endocarditis [Data Rich] | Male | 0-6 days | 80+ years | 0·18127 0·330453 0·131639 0·153831 0·99991 0·999673 | | | | | |
| Endocarditis [Data Rich] | Female | 0-6 days | 80+ years | 0·172816 0·335074 0·125424 0·146561 0·999901 0·999684 | | | | | |
| Other cardiovascular and circulatory diseases [Data Rich] | Male | 0-6 days | 80+ years | 0·167965 0·205341 0·123035 0·137883 0·998134 0·99649 | | | | | |
| Other cardiovascular and circulatory diseases [Data Rich] | Female | 0-6 days | 80+ years | 0·166953 0·207281 0·121027 0·135104 0·998454 0·996831 | | | | | |

| **Appendix Table 8: Count of CVD literature data included for GBD 2015** | | | | |
| --- | --- | --- | --- | --- |
| **Model** | **Subtype** | **Prevalence** | **Incidence** | **Mortality risk** |
| Rheumatic heart disease | Low‐income model | 77 | 0 | 0 |
| Rheumatic heart disease | High‐income model | 8 | 0 | 0 |
| Ischemic heart disease | Myocardial infarction | 0 | 93 | 61 |
| Ischemic heart disease | Angina | 72 | 0 | 7 |
| Cerebrovascular disease | Cerebrovascular disease | 53 | 0 | 8 |
| Cerebrovascular disease | Ischemic Stroke | 0 | 71 | 45 |
| Cerebrovascular disease | Hemorrhagic or other stroke | 0 | 71 | 34 |
| Atrial fibrillation and flutter |  | 71 | 24 | 15 |
| Peripheral vascular disease |  | 23 | 3 | 1 |
| Acute endocarditis |  | 0 | 14 | 1 |

| **Appendix Table 9: GBD 2015 nonfatal model covariates by CVD Cause** | | | | | |  |
| --- | --- | --- | --- | --- | --- | --- |
| **Model** | **Subtype** | **Covariate** | **Parameter** | **Beta** | **Exponentiated beta** |  |
| Rheumatic heart disease | Endemic model | LDI (I$ per capita) | prevalence | ‐.0815 (‐.2114 to .0037) | .9217 (.8094 to .9963) |  |
| Rheumatic heart disease | Endemic model | Log‐transformed agestandardized SEV scalar: RHD | prevalence | 1.086 (.5452 to 1.483) | 2.962 (1.725 to 4.406) |  |
| Rheumatic heart disease | Endemic model | LDI (I$ per capita) | excess mortality rate | ‐.3748 (‐.4892 to .2336) | .6874 (.6131 to .7917) |  |
| Rheumatic heart disease | Non‐endemic model | US Claims 2000 | prevalence | .3339 (.2491 to .4236) | 1.396 (1.283 to 1.527) |  |
| Rheumatic heart disease | Non‐endemic model | US Claims 2010 | prevalence | .6061 (.5419 to .6678) | 1.833 (1.719 to 1.95) |  |
| Rheumatic heart disease | Non‐endemic model | LDI (I$ per capita) | excess mortality rate | ‐.4849 (‐.4999 to ‐.465) | .6158 (.6066 to .6281) |  |
| Ischemic heart disease | Myocardial infarction | Diagnostic blood sample (troponin) | incidence | ‐.4432 (‐.4543 to ‐.44) | .6419  (.6349 to .644) |  |
| Ischemic heart disease | Myocardial infarction | Hospital data | incidence | ‐1.5e‐04 (‐4.4e‐04 to ‐ 4.1e‐05) | .9999 (.9996 to 1) |  |
| Ischemic heart disease | Myocardial infarction | First ever MI | incidence | ‐.002 (‐.009 to ‐5.1e‐05) | .998 (.9911 to .9999) |  |
| Ischemic heart disease | Myocardial infarction | Non fatal MI | incidence | ‐8.9e‐04 (‐.0024 to ‐ 2.3e‐04) | .9991 (.9976 to .9998) |  |
| Ischemic heart disease | Myocardial infarction | LDI (I$ per capita) | excess mortality rate | ‐.1005 (‐.1027 to ‐.1) | .9044 (.9024 to .9048) |  |
| Ischemic heart disease | Asymptomatic ischemic heart disease | Log‐transformed agestandardized SEV scalar: IHD | incidence | .9319 (.9187 to .9452) | 2.539# (2.506 to 2.573) |  |
| Ischemic heart disease | Angina | RAQ, female, less than 50 | prevalence | 2.435 (2.326 to 2.497) | 11.42 (10.24 to 12.15) |  |
| Ischemic heart disease | Angina | RAQ, male, less than 50 | prevalence | .9454 (.9349 to .9499) | 2.574 (2.547 to 2.585) |  |
| Ischemic heart disease | Angina | RAQ, female, 50 to 64 | prevalence | 1.484 (1.447 to 1.5) | 4.411 (4.25 to 4.482) |  |
| Ischemic heart disease | Angina | RAQ, male, 50 to 64 | prevalence | .9897 (.9606 to .9997) | 2.69 (2.613 to 2.717) |  |
| Ischemic heart disease | Angina | RAQ, female, 65 plus | prevalence | .2929 (.2719 to .2998) | 1.34 (1.312 to 1.35) |  |
| Ischemic heart disease | Angina | RAQ, male, 65 plus | prevalence | .2891 (.2582 to .2997) | 1.335 (1.295 to 1.349) |  |
| Ischemic heart disease | Angina | Log‐transformed agestandardized SEV scalar: IHD | prevalence | 1.238 (1.209 to 1.249) | 3.449 (3.35 to 3.487) |  |
| Cerebrovascular disease, step 1 | Chronic stroke; any type | Log‐transformed agestandardized SEV scalar: Stroke | prevalence | .7833 (.7512 to .8785) | 2.189 (2.12 to 2.407) |  |
| Cerebrovascular disease, step 1 | Chronic stroke; any type | LDI (I$ per capita) | excess mortality rate | ‐.1792 (‐.1819 to ‐.1769) | .836 (.8337 to .8379) |  |
| Cerebrovascular disease, step 1 | First ever acute hemorrhagic stroke | Hospital data | incidence | .5278 (.5223 to .5298) | 1.695 (1.686 to 1.699) |  |
| Cerebrovascular disease, step 1 | First ever acute hemorrhagic stroke | Any stroke | incidence | 1.359 (1.313 to 1.388) | 3.892 (3.717 to 4.007) |  |
| Cerebrovascular disease, step 1 | First ever acute hemorrhagic stroke | First‐ever acute stroke, ischemic or hemorrhagic | incidence | .4925 (.4163 to .5291) | 1.636 (1.516 to 1.697) |  |
| Cerebrovascular disease, step 1 | First ever acute hemorrhagic stroke | Log‐transformed agestandardized SEV scalar: hemorrhagic stroke | incidence | 1.243 (1.227 to 1.25) | 3.468 (3.411 to 3.49) |  |
| Cerebrovascular disease, step 1 | First ever acute hemorrhagic stroke | Any stroke | excess mortality rate | ‐.4216 (‐.5741 to ‐.2617) | .656 (.5632 to .7698) |  |
| Cerebrovascular disease, step 1 | First ever acute hemorrhagic stroke | First‐ever acute stroke, ischemic or hemorrhagic | excess mortality rate | ‐.1409 (‐.3484 to .0613) | .8685 (.7058 to 1.063) |  |
| Cerebrovascular disease, step 1 | First ever acute ischemic stroke | Hospital data | incidence | .002 (4.3e‐05 to .0067) | 1.002 (1 to 1.007) |  |
| Cerebrovascular disease, step 1 | First ever acute ischemic stroke | Any stroke | incidence | .4687 (.4653 to .47) | 1.598 (1.592 to 1.6) |  |
| Cerebrovascular disease, step 1 | First ever acute ischemic stroke | First‐ever acute stroke, ischemic or hemorrhagic | incidence | .5142 (.4772 to .5296) | 1.672 (1.612 to 1.698) |  |
| Cerebrovascular disease, step 1 | First ever acute ischemic stroke | Log‐transformed agestandardized SEV scalar: ischemic stroke | incidence | 1.106 (1.025 to 1.186) | 3.021 (2.787 to 3.274) |  |
| Cerebrovascular disease, step 2 | Chronic stroke, any type with CSMR | Log‐transformed agestandardized SEV scalar: Stroke | prevalence | .8185  (.7518 to .9986) | 2.267  (2.121 to 2.714) |  |
| Cerebrovascular disease, step 2 | Chronic stroke, any type with CSMR | LDI (I$ per capita) | excess mortality rate | ‐.1879  (‐.1917 to ‐.1845) | .8287  (.8256 to .8315) |  |
| Cerebrovascular disease, step 2 | First‐ever acute hemorrhagic stroke with CSMR | Any stroke | incidence | 1.401  (1.4 to 1.407) | 4.06  (4.055 to 4.084) |  |
| Cerebrovascular disease, step 2 | First‐ever acute hemorrhagic stroke with CSMR | First‐ever acute stroke, ischemic or hemorrhagic | incidence | 9.8e‐04  (2.2e‐ 04 to .0049) | 1.001  (1 to 1.005) |  |
| Cerebrovascular disease, step 2 | First‐ever acute hemorrhagic stroke with CSMR | Log‐transformed SEV scalar: Hem stroke | incidence | 1.152  (1.031 to 1.243) | 3.164  (2.804 to 3.466) |  |
| Cerebrovascular disease, step 2 | First‐ever acute hemorrhagic stroke with CSMR | Any stroke | excess mortality rate | ‐.5999  (‐.7527 to ‐.4538) | .5489  (.4711 to .6352) |  |
| Cerebrovascular disease, step 2 | First‐ever acute hemorrhagic stroke with CSMR | First‐ever acute stroke, ischemic or hemorrhagic | excess mortality rate | ‐.2336  (‐.512 to .0366) | .7917  (.5993 to 1.037) |  |
| Cerebrovascular disease, step 2 | First‐ever acute ischemic stroke with CSMR | Any stroke | incidence | .3452  (.3401 to .3575) | 1.412  (1.405 to 1.43) |  |
| Cerebrovascular disease, step 2 | First‐ever acute ischemic stroke with CSMR | First‐ever acute stroke, ischemic or hemorrhagic | incidence | 3.4e‐04  (7.3e‐ 05 to 9.8e‐04) | 1  (1 to 1.001) |  |
| Cerebrovascular disease, step 2 | First‐ever acute ischemic stroke with CSMR | Log‐transformed agestandardized SEV scalar: Ischemic stroke | incidence | 1.248  (1.24 to 1.25) | 3.483  (3.456 to 3.49) |  |
| Cerebrovascular disease, step 2 | First‐ever acute ischemic stroke with CSMR | Any stroke | excess mortality rate | ‐.6897  (‐.8029 to ‐.5741) | .5017  (.448 to .5632) |  |
| Cerebrovascular disease, step 2 | First‐ever acute ischemic stroke with CSMR | First‐ever acute stroke, ischemic or hemorrhagic | excess mortality rate | ‐.869  (‐.9992 to ‐.7466) | .4194  (.3682 to .474) |  |
| Acute myocarditis |  | Hospital data | incidence | ‐1.716 (‐1.797 to ‐1.644) | .1798 (.1658 to .1932) |  |
| Acute myocarditis |  | All MarketScan, year 2000 | incidence | .2966 (.2528 to .3362) | 1.345 (1.288 to 1.4) |  |
| Acute myocarditis |  | All MarketScan, year 2010 | incidence | .1523 (.1139 to .1933) | 1.164 (1.121 to 1.213) |  |
| Acute myocarditis |  | Log‐transformed age‐ standardized SEV scalar: CMP | incidence | .695 (.5119 to 1.045) | 2.004 (1.668 to 2.843) |  |
| Atrial fibrillation and flutter |  | Hospital data | prevalence | ‐.9192 (‐.9199 to ‐.9175) | .3988 (.3986 to .3995) |  |
| Atrial fibrillation and flutter |  | All MarketScan, year 2000 | prevalence | ‐.2752 (‐.3101 to ‐.2468) | .7594 (.7334 to .7813) |  |
| Atrial fibrillation and flutter |  | All MarketScan, year 2010 | prevalence | .14 (.1105 to .1658) | 1.15 (1.117 to 1.18) |  |
| Atrial fibrillation and flutter |  | LDI (I$ per capita) | excess mortality rate | ‐.25 (‐.2501 to ‐.25) | .7788 (.7787 to .7788) |  |
| Peripheral vascular disease |  | Hospital data | prevalence | ‐1.998# (‐2 to ‐ 1.993) | .1356# (.1353 to .1363) |  |
| Peripheral vascular disease |  | All MarketScan, year 2000 | prevalence | ‐.7449# (‐ .7498 to ‐.733) | .4748# (.4725 to .4805) |  |
| Peripheral vascular disease |  | All MarketScan, year 2010 | prevalence | ‐.371# (‐.3739 to ‐.37) | .69# (.688 to .6907) |  |
| Peripheral vascular disease |  | All MarketScan, year 2012 | prevalence | ‐.3016# (‐ .3065 to ‐.3) | .7396# (.736 to .7408) |  |
| Peripheral vascular disease |  | Log‐transformed age‐standardized SEV scalar: PVD | prevalence | .7985# (.7506 to .9187) | 2.222# (2.118 to 2.506) |  |
| Acute endocarditis |  | Inpatient hospital data | incidence | .4484 (.1224 to .5861) | 1.566 (1.13 to 1.797) |  |
| Acute endocarditis |  | Claims data 2000 | incidence | ‐.0127 (‐1.913 to 2) | .9874 (.1476 to 7.389) |  |
| Acute endocarditis |  | Claims data 2010 | incidence | .002 (‐1.964 to 1.952) | 1.002 (.1403 to 7.041) |  |
| Acute endocarditis |  | Log LDI | incidence | .5018 (.5 to .5073) | 1.652 (1.649 to 1.661) |  |
| Acute endocarditis |  | Log‐transformed age‐ standardized SEV scalar endocarditis | excess mortality rate | ‐.2074 (‐.3416 to ‐.1001) | .8127 (.7106 to .9047) |  |

| **Appendix Table 10: List of International Classification of Diseases (ICD) codes mapped to the Global Burden of Disease cause list for Cardiovascular Disease Deaths** | | |
| --- | --- | --- |
| **Cause** | **ICD10** | **ICD9** |
| Cardiovascular diseases | A39.5-A39.53, B33.2-B33.24, D86.85, G45-G46.8, I01-I01.9, I02.0, I05-I09.9, I11-I11.9, I20-I25.9, I28- | 036.41-036.43, 036.6, 074.2, 074.21-074.23, 391-391.9, 392.0, 393-398.99, 402-402.91, 410-414.9, |
|  | I28.8, I30-I31.1, I31.8-I43.9, I47-I48.92, I51.0-I51.5, I60-I61.9, I62.0-I62.03, I63-I63.9, I65-I66.9, I67.0-I67.3 | 417-417.9, 420-423, 423.1-425.9, 427-427.32, 427.6-427.89, 429.0-429.1, 430-435.9, 437.0-437.2, |
|  | I67.5-I67.6, I68.0-I68.2, I69.0-I69.398, I70.2-I70.799, I71-I73.9, I77-I83.93, I86-I89.9, I91.9, I98 | 437.5-437.8, 441-443.9, 447-454.9, 456, 456.3-457.9, 459, 459.1-459.39 |
| Rheumatic heart disease | I01-I01.9, I02.0, I05-I09.9 | 391-391.9, 392.0, 393-398.99 |
| Ischemic heart disease | I20-I25.9 | 410-414.9 |
| Cerebrovascular disease | G45-G46.8, I60-I61.9, I62.0-I62.03, I63-I63.9, I65-I66.9, I67.0-I67.3, I67.5-I67.6, I68.1-I68.2, I69.0-I69.398 | 430-435.9, 437.0-437.2, 437.5-437.8 |
| Ischemic stroke | G45-G46.8, I63-I63.9, I65-I66.9, I67.2-I67.3, I67.5-I67.6, I69.3-I69.398 | 433-435.9, 437.0-437.1, 437.5-437.8 |
| Hemorrhagic stroke | I60-I61.9, I62.0-I62.03, I67.0-I67.1, I68.1-I68.2, I69.0-I69.298 | 430-432.9, 437.2 |
| Hypertensive heart disease | I11-I11.9 | 402-402.91 |
| Cardiomyopathy and myocarditis | A39.52, B33.2-B33.24, D86.85, I40-I43.9, I51.4-I51.5 | 036.43, 036.6, 074.23, 422-422.99, 425-425.9, 429.0-429.1 |
| Atrial fibrillation and flutter | I48-I48.92 | 427.3-427.32 |
| Aortic aneurysm | I71-I71.9 | 441-441.9 |
| Peripheral vascular disease | I70.2-I70.799, I73-I73.9 | 443.0-443.9 |
| Endocarditis | A39.51, I33-I33.9, I38-I39.9 | 036.42, 074.22, 421-421.9, 424.9-424.91 |
| Other cardiovascular and circulatory diseases | A39.5-A39.50, A39.53, I28-I28.8, I30-I31.1, I31.8-I32.8, I34-I37.9, I47-I47.9, I51.0-I51.3, I68.0, I72-I72.9, I77-I83.93, I86-I89.9, I91.9, I98 | 036.41, 074.2, 074.21, 417-417.9, 420-420.99, 423, 423.1-424.8, 424.99, 427-427.2, 427.6-427.89, 442-443, 447-454.9, 456, 456.3-457.9, 459, 459.1-459.39 |

**Appendix Table 11. GATHER checklist of information that should be included in reports of global health estimates, with description of compliance and location of information for Global, Regional, and National Burden of Cardiovascular Diseases for 10 Causes, 1990–2015**

| # | GATHER checklist item | Description of compliance | Reference |
| --- | --- | --- | --- |
| **Objectives and funding** | | | |
| 1 | Define the indicators, populations, and time periods for which estimates were made. | Narrative provided in paper and appendix describing indicators, definitions, and populations. | Manuscript; Methods Appendix, Section 1. GBD Overview |
| 2 | List the funding sources for the work. | Funding sources listed at end of paper. | Funding Sources |
| **Data Inputs** | | | |
| *For all data inputs from multiple sources that are synthesized as part of the study:* | | | |
| 3 | Describe how the data were identified and how the data were accessed. | Narrative description of data seeking methodology provided in previously published appendices. | 1. GBD 2015 Mortality and Causes of Death Collaborators. Global, regional, and national life expectancy, all-cause mortality, and cause-specific mortality for 249 causes of death, 1980-2015: a systematic analysis for the Global Burden of Disease Study 2015. The Lancet 2016; 388:1459–1544. 2. GBD 2015 Disease and Injury Incidence and Prevalence Collaborators. Global, regional, and national incidence, prevalence, and years lived with disability for 310 diseases and injuries, 1990-2015: a systematic analysis for the Global Burden of Disease Study 2015. The Lancet 2016; 388:1603–1658 |
| 4 | Specify the inclusion and exclusion criteria. Identify all ad-hoc exclusions. | Narrative about inclusion and exclusion criteria by data type provided in previously published appendices. | 1. GBD 2015 Mortality and Causes of Death Collaborators. Global, regional, and national life expectancy, all-cause mortality, and cause-specific mortality for 249 causes of death, 1980-2015: a systematic analysis for the Global Burden of Disease Study 2015. The Lancet 2016; 388:1459–1544. 2. GBD 2015 Disease and Injury Incidence and Prevalence Collaborators. Global, regional, and national incidence, prevalence, and years lived with disability for 310 diseases and injuries, 1990-2015: a systematic analysis for the Global Burden of Disease Study 2015. The Lancet 2016; 388:1603–1658 |
| 5 | Provide information on all included data sources and their main characteristics. For each data source used, report reference information or contact name/institution, population represented, data collection method, year(s) of data collection, sex and age range, diagnostic criteria or measurement method, and sample size, as relevant. | Interactive, online data source tool that provides metadata for data sources by component, geography, cause, risk, or impairment has been developed. | Online data tools: <http://ghdx.healthdata.org/gbd-2015/data-input-sources> |
| 6 | Identify and describe any categories of input data that have potentially important biases (e.g., based on characteristics listed in item 5). | Summary of known biases by cause included in methodological approaches sections of previously published appendices. | 1. GBD 2015 Mortality and Causes of Death Collaborators. Global, regional, and national life expectancy, all-cause mortality, and cause-specific mortality for 249 causes of death, 1980-2015: a systematic analysis for the Global Burden of Disease Study 2015. The Lancet 2016; 388:1459–1544. 2. GBD 2015 Disease and Injury Incidence and Prevalence Collaborators. Global, regional, and national incidence, prevalence, and years lived with disability for 310 diseases and injuries, 1990-2015: a systematic analysis for the Global Burden of Disease Study 2015. The Lancet 2016; 388:1603–1658 |
| *For data inputs that contribute to the analysis but were not synthesized as part of the study:* | | | |
| 7 | Describe and give sources for any other data inputs. | Included in list of all data sources provided on online data source tool. | Online data tools <http://ghdx.healthdata.org/gbd-2015/data-input-sources> |
| *For all data inputs:* | | | |
| 8 | Provide all data inputs in a file format from which data can be efficiently extracted (e.g., a spreadsheet as opposed to a PDF), including all relevant meta-data listed in item 5. For any data inputs that cannot be shared due to ethical or legal reasons, such as third-party ownership, provide a contact name or the name of the institution that retains the right to the data. | Downloads of input data will be available through online tools, including data visualization tools and data query tools. Input data not available in tools will be made available upon request. | Online data tools <http://www.healthdata.org/results/data-visualizations>; <http://ghdx.healthdata.org/>; <http://ghdx.healthdata.org/gbd-data-tool> |
| **Data analysis** | | | |
| 9 | Provide a conceptual overview of the data analysis method. A diagram may be helpful. | Flow diagrams of the overall methodological processes, as well as cause-specific modelling processes have been provided. | Methods Appendix, Section 2. Causes of death modelling methods; Methods Appendix, Section 3. Nonfatal modelling methods |
| 10 | Provide a detailed description of all steps of the analysis, including mathematical formulae. This description should cover, as relevant, data cleaning, data pre-processing, data adjustments and weighting of data sources, and mathematical or statistical model(s). | Detailed descriptions of all steps of the analysis were included in the methodological approaches sections of previously published appendices. | 1. GBD 2015 Mortality and Causes of Death Collaborators. Global, regional, and national life expectancy, all-cause mortality, and cause-specific mortality for 249 causes of death, 1980-2015: a systematic analysis for the Global Burden of Disease Study 2015. The Lancet 2016; 388:1459–1544. 2. GBD 2015 Disease and Injury Incidence and Prevalence Collaborators. Global, regional, and national incidence, prevalence, and years lived with disability for 310 diseases and injuries, 1990-2015: a systematic analysis for the Global Burden of Disease Study 2015. The Lancet 2016; 388:1603–1658 |
| 11 | Describe how candidate models were evaluated and how the final model(s) were selected. | Provided in the methodological write-ups of previously published appendices. | 1. GBD 2015 Mortality and Causes of Death Collaborators. Global, regional, and national life expectancy, all-cause mortality, and cause-specific mortality for 249 causes of death, 1980-2015: a systematic analysis for the Global Burden of Disease Study 2015. The Lancet 2016; 388:1459–1544. 2. GBD 2015 Disease and Injury Incidence and Prevalence Collaborators. Global, regional, and national incidence, prevalence, and years lived with disability for 310 diseases and injuries, 1990-2015: a systematic analysis for the Global Burden of Disease Study 2015. The Lancet 2016; 388:1603–1658 |
| 12 | Provide the results of an evaluation of model performance, if done, as well as the results of any relevant sensitivity analysis. | Provided in the methodological write-ups of previously published appendices. | 1. GBD 2015 Mortality and Causes of Death Collaborators. Global, regional, and national life expectancy, all-cause mortality, and cause-specific mortality for 249 causes of death, 1980-2015: a systematic analysis for the Global Burden of Disease Study 2015. The Lancet 2016; 388:1459–1544. 2. GBD 2015 Disease and Injury Incidence and Prevalence Collaborators. Global, regional, and national incidence, prevalence, and years lived with disability for 310 diseases and injuries, 1990-2015: a systematic analysis for the Global Burden of Disease Study 2015. The Lancet 2016; 388:1603–1658 |
| 13 | Describe methods for calculating uncertainty of the estimates. State which sources of uncertainty were, and were not, accounted for in the uncertainty analysis. | Provided in the methodological write-ups of previously published appendices. | 1. GBD 2015 Mortality and Causes of Death Collaborators. Global, regional, and national life expectancy, all-cause mortality, and cause-specific mortality for 249 causes of death, 1980-2015: a systematic analysis for the Global Burden of Disease Study 2015. The Lancet 2016; 388:1459–1544. 2. GBD 2015 Disease and Injury Incidence and Prevalence Collaborators. Global, regional, and national incidence, prevalence, and years lived with disability for 310 diseases and injuries, 1990-2015: a systematic analysis for the Global Burden of Disease Study 2015. The Lancet 2016; 388:1603–1658 |
| 14 | State how analytic or statistical source code used to generate estimates can be accessed. | Access statement provided. | http://ghdx.healthdata.org/global-burden-disease-study-2015 |
| **Results and Discussion** | | | |
| 15 | Provide published estimates in a file format from which data can be efficiently extracted. | GBD 2015 results are available through online data visualization tools, the Global Health Data Exchange, and the online data query tool (these tools are already available for GBD 2013 results). | Online data tools <http://www.healthdata.org/results/data-visualizations>; <http://ghdx.healthdata.org/>; <http://ghdx.healthdata.org/gbd-data-tool> |
| 16 | Report a quantitative measure of the uncertainty of the estimates (e.g. uncertainty intervals). | Uncertainty intervals are provided with all results. | Main text; Online data tools <http://www.healthdata.org/results/data-visualizations>; <http://ghdx.healthdata.org/>; <http://ghdx.healthdata.org/gbd-data-tool> |
| 17 | Interpret results in light of existing evidence. If updating a previous set of estimates, describe the reasons for changes in estimates. | Discussion of methodological changes between GBD rounds provided in the appendix. | Methods Appendix, Section 2. Causes of death modelling methods; Methods Appendix, Section 3. Nonfatal modelling methods |
| 18 | Discuss limitations of the estimates. Include a discussion of any modelling assumptions or data limitations that affect interpretation of the estimates. | Discussion of limitations provided in the narrative of the main paper as well as in the methodological write-ups of previously published appendices | 1. Main text, Limitations 2. GBD 2015 Mortality and Causes of Death Collaborators. Global, regional, and national life expectancy, all-cause mortality, and cause-specific mortality for 249 causes of death, 1980-2015: a systematic analysis for the Global Burden of Disease Study 2015. The Lancet 2016; 388:1459–1544. 3. GBD 2015 Disease and Injury Incidence and Prevalence Collaborators. Global, regional, and national incidence, prevalence, and years lived with disability for 310 diseases and injuries, 1990-2015: a systematic analysis for the Global Burden of Disease Study 2015. The Lancet 2016; 388:1603–1658 |

**Figure 1: Increase in mortality in CVD after redistributing garbage codes by different years and ICD coding system**


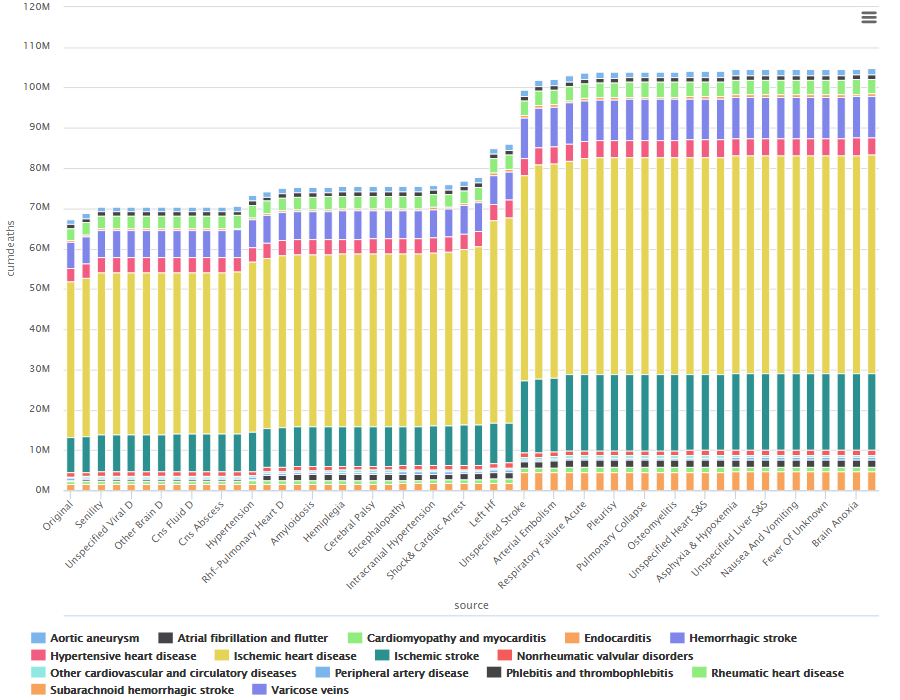

Supplement: Online Data [file mmc2.docx]
